# Targeting Acute Myelogenous Leukemia Using Potent Human Dihydroorotate Dehydrogenase Inhibitors Based on the 2-Hydroxypyrazolo[1,5-*a*]pyridine Scaffold: SAR of the Aryloxyaryl Moiety

**DOI:** 10.1021/acs.jmedchem.2c00496

**Published:** 2022-09-26

**Authors:** Stefano Sainas, Marta Giorgis, Paola Circosta, Giulio Poli, Marta Alberti, Alice Passoni, Valentina Gaidano, Agnese C. Pippione, Nicoletta Vitale, Davide Bonanni, Barbara Rolando, Alessandro Cignetti, Cristina Ramondetti, Alessia Lanno, Davide M. Ferraris, Barbara Canepa, Barbara Buccinnà, Marco Piccinini, Menico Rizzi, Giuseppe Saglio, Salam Al-Karadaghi, Donatella Boschi, Riccardo Miggiano, Tiziano Tuccinardi, Marco L. Lolli

**Affiliations:** † Department of Drug Science and Technology, University of Turin, Via P. Giuria 9, Turin 10125, Italy; ‡ Department of Clinical and Biological Sciences, University of Turin, Regione Gonzole 10, Orbassano, Turin 10043, Italy; § Molecular Biotechnology Center, University of Turin, Via Nizza 52, Turin 10126, Italy; ∥ Department of Pharmacy, University of Pisa, Via Bonanno 6, Pisa 56126, Italy; ⊥ Department of Pharmaceutical Sciences, University of Piemonte Orientale, Via G. Bovio 6, Novara 28100, Italy; # Laboratory of Mass Spectrometry, Department of Environmental Health Sciences, Istituto di Ricerche Farmacologiche Mario Negri IRCCS, Via Mario Negri 2, Milan 20156, Italy; ¶ Division of Hematology and Cell Therapy, AO Ordine Mauriziano, Largo Filippo Turati, 62, Turin 10128, Italy; ∇ Department of Molecular Biotechnology and Health Sciences, University of Turin, Via Nizza 52, Turin 10126, Italy; ○ Life Science Department, University of Modena, Via Università 4, Modena 41121, Italy; ⧫ Department of Oncology, University of Turin, Via Michelangelo 27/B, Turin 10125, Italy; †† GEM FORLAB, Via Ing. Comotto 36, Caluso, Turin, 10014, Italy; ‡‡ Department of Biochemistry and Structural Biology, Lund University, Naturvetarvägen 14, Box 124, Lund 221 00, Sweden

## Abstract

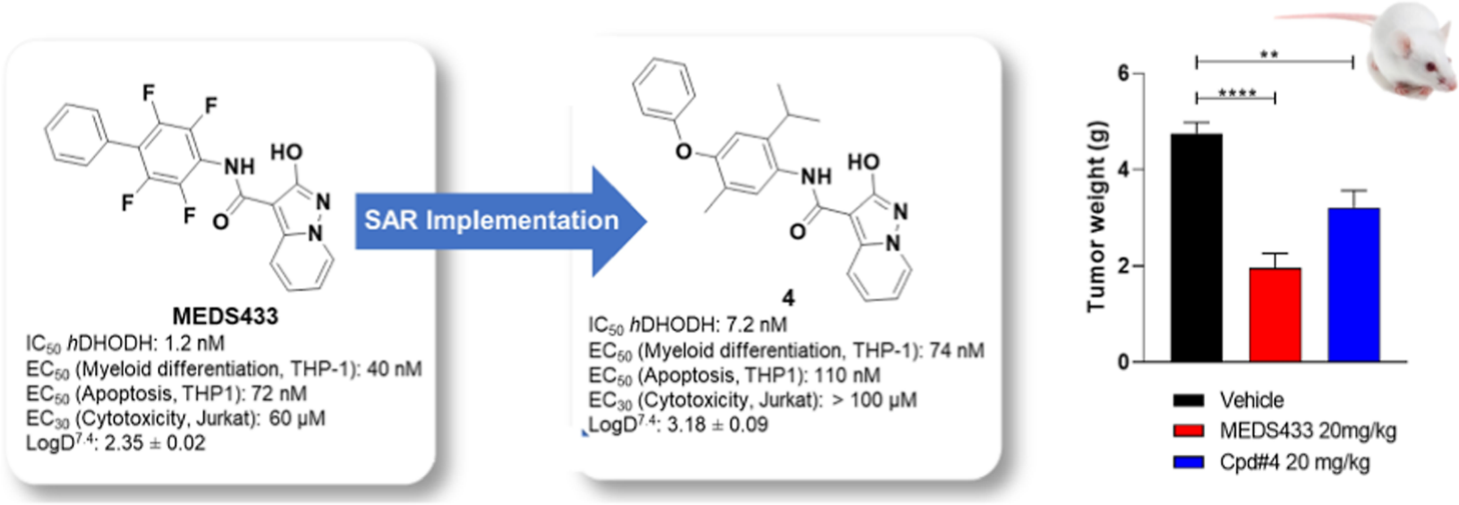

In recent years, human dihydroorotate dehydrogenase inhibitors have been associated with acute myelogenous leukemia as well as studied as potent host targeting antivirals. Starting from MEDS433 (IC_50_ 1.2 nM), we kept improving the structure–activity relationship of this class of compounds characterized by 2-hydroxypyrazolo[1,5-*a*]pyridine scaffold. Using an in silico/crystallography supported design, we identified compound **4** (IC_50_ 7.2 nM), characterized by the presence of a decorated aryloxyaryl moiety that replaced the biphenyl scaffold, with potent inhibition and pro-differentiating abilities on AML THP1 cells (EC_50_ 74 nM), superior to those of brequinar (EC_50_ 249 nM) and boosted when in combination with dipyridamole. Finally, compound **4** has an extremely low cytotoxicity on non-AML cells as well as MEDS433; it has shown a significant antileukemic activity in vivo in a xenograft mouse model of AML.

## Introduction

**Chart 1:**
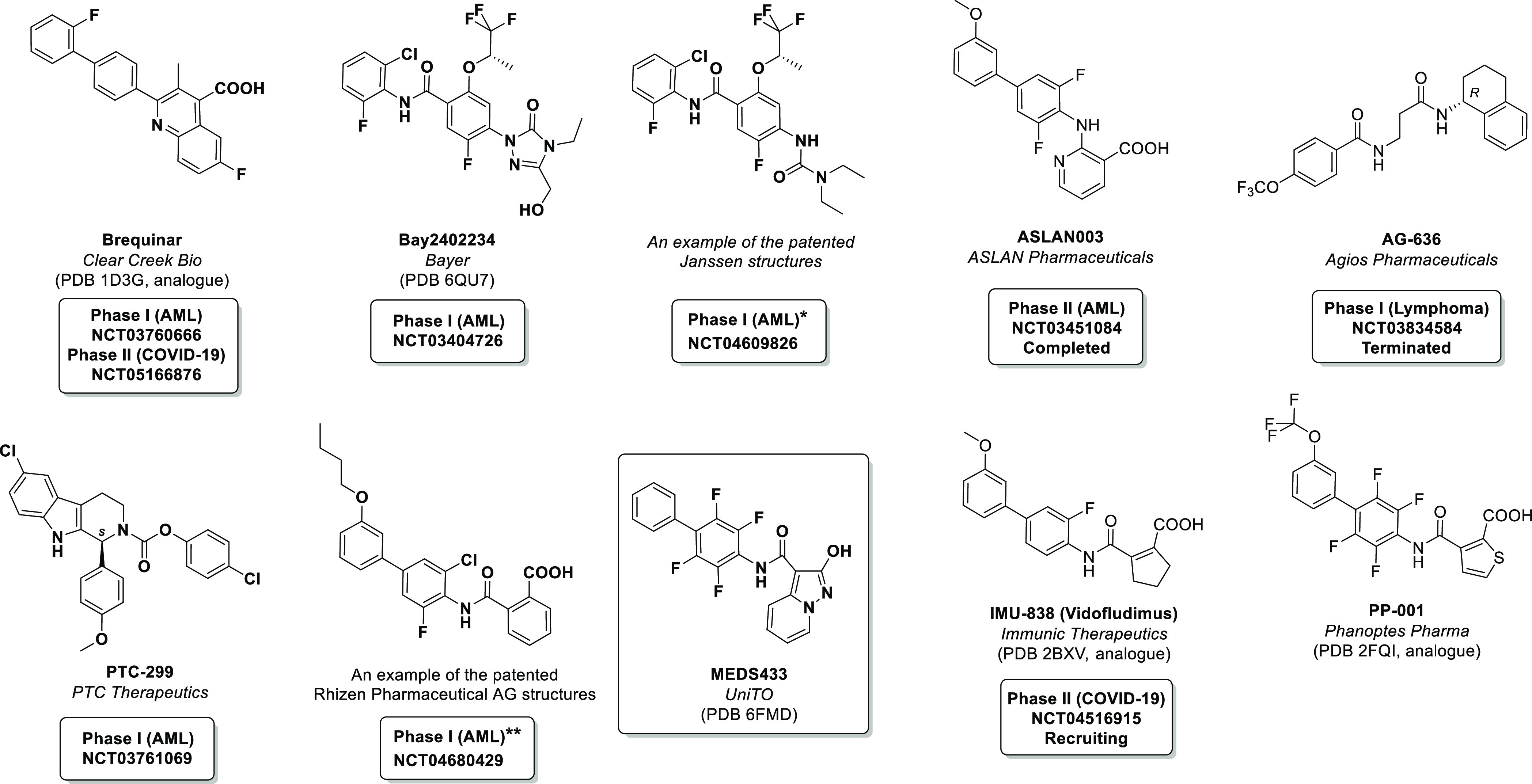
During 2020/2021, Four Major Companies Were Known to Run Phase I/II AML Clinical Trials Involving Newly Patented *h*DHODH Inhibitors As Well As The Well-Known Inhibitor Brequinara

Human dihydroorotate dehydrogenase (*h*DHODH, EC 1.3.99.11) plays a key role in the de novo pyrimidine biosynthesis. Being located in the inner mitochondrial membrane, it catalyzes the oxidation of dihydroorotate to orotate by involving the cofactor flavin mononucleotide (FMN). This latter is then regenerated by transferring electrons to ubiquinone (coenzyme Q), which is then released in the inner mitochondrial membrane, relating the *h*DHODH activity to the mitochondrial electron transport chain (ETC).^1,2^ Since the 1980s, with the development of drug-like inhibitors such as leflunomide and brequinar, *h*DHODH has been considered a validated target in diseases that involve cellular proliferation, such as autoimmune diseases and cancer.^3,4^ In recent years, this target has received renewed interest from the scientific and pharma community due to its potential as a therapeutic target in acute myeloid leukemia (AML)^5,6^ and virus replication mechanisms (Chart 1).^7,8^

a (*) Representative *h*DHODH inhibitor patented by Janssen; the structure of compound JNJ74856665 involved in the clinical trial has not been disclosed yet^22−25^ (**). An example of the patented Rhizen Pharmaceutical AG, the structure of the Rhizen Pharmaceutical AG compound RP7214 involved in the clinical trials has not been disclosed yet.^26,27^ MEDS433, an advanced preclinical candidate designed by the authors, is also included in Chart 1. Structures of other *h*DHODH inhibitors involved in several clinical trials. The PDB ID of the structures of the inhibitors in complex with *h*DHODH are shown, when available.

AML is the most common acute leukemia in adults and affects the myeloid lineage of white blood cells. It is a severe disease with a poor prognosis: typically, AML is fatal within weeks or months if left untreated, while the 5 year survival rate is approximately 25% with current therapies. Leukemic blasts are immature cells that have lost the ability to differentiate into adult white blood cells and accumulate in the bone marrow, interfering with the production of normal blood cells. The discovery^9,10^ that *h*DHODH inhibitors can promote myeloid differentiation opened new treatment scenarios for the disease. Sykes et al.^10^ were first to suggest that AML cells, unlike non-leukemic cells, may be particularly sensitive to “pyrimidine starvation”, a condition induced by *h*DHODH inhibitors by blocking the de novo biosynthesis. Although the mechanism of action of *h*DHODH inhibitors has not been fully elucidated,^3,5,6^ pyrimidine starvation seems to force AML cells to choose differentiation over self-renewal. As this approach does not depend on the presence of specific mutations, it could be applied to, possibly, all AML subtypes, potentially aligning them to the acute promyelocytic leukemia (APL) subtype. APL is currently curable in more than 90% of cases^11^ using a differentiation therapy based on all-trans-retinoic acid (ATRA), in association with a pro-apoptotic agent (chemotherapy or arsenic trioxide).^12,13^

The unpreparedness of our society to facing the COVID-19 (COronaVIrus Disease) pandemic clearly revealed the absence of effective broad-spectrum antiviral agents, therapeutically effective against severe acute respiratory syndrome coronavirus 2 (SARS-CoV-2) variants and other viruses with pandemic potential.^14^*h*DHODH inhibitors, by reducing the pyrimidine pool required for virus replication in host cells, have shown potent antiviral activity against a broad spectrum of viruses including SARS-CoV-2, thus becoming one of the most interesting therapeutic options for COVID-19.^7,8,15−21^

*h*DHODH inhibitors bind in a tunnel-like pocket, called “lipophilic patch”, which is used by ubiquinone to reach FMN during the enzymatic reaction.^4^ The tunnel, that is lipophilic at the entrance, is exposed to the inner mitochondrial membrane and becomes more polar in the inner part which approaches FMN. Here, R136 (Figure 2) of *h*DHODH often plays a key role in binding potent inhibitors characterized by the presence of an acidic moiety. It is the case of brequinar, a compound developed by Du Pont (DuP-785) in 1985^28^ that is considered to be one of the most potent *h*DHODH inhibitors discovered to date. Clear Creek Bio, who acquired it from Bristol Myers Squibb, has recently completed Phase I/II clinical trials with brequinar for the treatment of patients with relapsed/refractory AML (NCT03760666, no result released yet). The design used by Rhizen Pharmaceutical AG also involves an acidic moiety in the structure. In this case, Phase I trials (NCT04680429) on RP7214 were completed in August 2021, although also in this case no results have been released yet. The other inhibitors undergoing clinical trials are neutral compounds: PTC-299^29^ (PTC pharmaceuticals, Phase I, NCT03761069) and JNJ74856665^23,25,30^ (Janssen in Phase I, NCT04609826); both trials are in the recruiting phase. Phase I/II trials of BAY2402234^31^ (NCT03404726), supported by Bayer, which started in January 2018, were terminated at the beginning of 2021 due to the lack of adequate clinical benefits.^32^

Similarly, in July 2019, trials involving ASLAN003^33^ (NCT03451084) were terminated for the same reason,.^34^ This scenario raises an important question: why have inhibitors of the pyrimidine biosynthesis, and in particular *h*DHODH inhibitors, been unsuccessful in clinical trials?^3^ Brequinar failure in solid tumor clinical trials back in the nineties^35^ has been recently subjected to extensive analyses, suggesting that clinically relevant uridine depletion may not have occurred,^36^ preventing the tumor cells to enter a significant “pyrimidine starvation” condition.^5^ Generally speaking, this situation could be due to an insufficient potency in vivo: the inhibitor, although able to potently inhibit the *h*DHODH enzyme in vitro, could suffer cell permeability issues that limit its efficacy in vivo. Another problem could be due to an inadequate dosing: the schedule, in fact, must be carefully optimized following the drug pharmacokinetic to avoid interruptions of the “pyrimidine starvation” continuum between one administration and the following, allowing some residual *h*DHODH activity. Finally, the presence of the salvage mechanism, which allows extracellular nucleosides, including uridine, to enter into the cells through the human Equilibrative Nucleoside Transporter (*h*ENT1/2) channels, may also be considered as a possible explanation of the reduced efficacy of *h*DHODH inhibitors in an in human environment. While extracellular uridine present at a physiological concentration of 5 μM in human plasma is sufficient to allow life of cells when in the resting state,^37^ higher uridine levels are present in solid growing tumors. These high uridine levels and the subsequent lack of complete pyrimidine starvation could explain the lack of antitumor activity of brequinar observed in solid tumors.

In the past few years, our group as well as others have helped to redefine the optimal paradigm in the design of *h*DHODH inhibitors, which could translate the high potency on the isolated enzyme into potent cellular/in vivo *h*DHODH-associated activities.

In 2018, the authors discovered MEDS433^38^ (Chart 1) as a representative of a novel class of *h*DHODH inhibitors structurally based on an unusual carboxylic group hydroxyazole bioisostere,^38−42^ 2-hydroxypyrazolo[1,5-*a*]pyridine, which is involved in a key interaction with R136 in the ubiquinone binding site. As already mentioned, since the ligands have to reach the inner mitochondrial membrane where *h*DHODH is located,^43^ lipophilicity must be taken into account during ligand design. Log*D*^7.4^ above 2.5 has been considered to be essential for the translation of potent in vitro *h*DHODH enzymatic activity inhibition into a substantial effect in cells.^44^ In this sense, the more lipophilic MEDS433, the in vitro activity of which is comparable to that of brequinar (IC_50_ 1.2 nM vs 1.8 nM, respectively), was found to be superior to brequinar in cells.^38^ Unfortunately, because higher lipophilicity is usually associated with reduced solubility and adverse ADME, we and other researchers investigated alternative strategies for the improvement of the in vivo/in human efficacy of the designed *h*DHODH inhibitors. Coadministration of *h*DHODH inhibitors with ENT1/2 blockers, such as dipyridamole, to temporarily block both the de novo synthesis and the uridine salvage pathway produces an enhancement of the effect. The combination of MEDS433 and dipyridamole, indeed, has already shown to greatly boost its antileukemic activity^45^ as well as its antiviral activity.^18^ In the present work, we continued to design optimized *h*DHODH inhibitors based on the 2-hydroxypyrazolo[1,5-*a*]pyridine scaffold and to study effective ways of their optimal future application in vivo.

In the present work, we move forward from that discovery in the same two directions followed in Sainas et al.;^39^ on one hand, we continued to design optimized *h*DHODH inhibitors based on the 2-hydroxypyrazolo[1,5-*a*]pyridine scaffold exploring the structure–activity relationships (SARs) of this class of compounds in the attempt to provide analogues with better potency and drug-like profiles. On the other, we continue the investigation of MEDS433: having already observed good metabolic stability and no toxic profile when administered at doses of 10 and 25 mg/kg every 3 days for 5 weeks (Balb/c mice), in this paper, we will first describe its in vivo efficacy with that of the best compound of new series on a xenograft AML animal model, creating the proof of concepts for its future applications.

**Figure 1:**
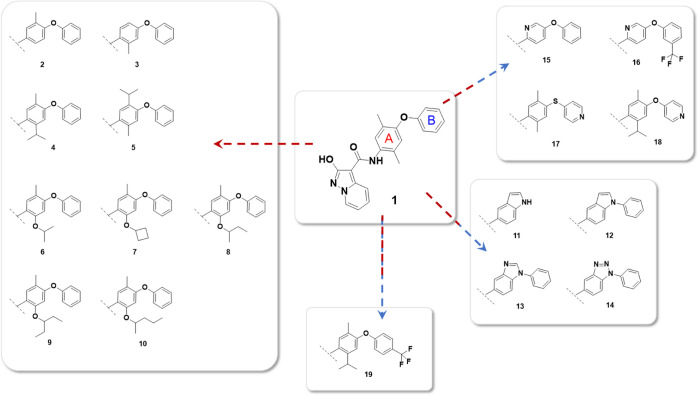
Lead compound **1** and designed compounds **2–19** involved in the SAR exploration.

Targeting *h*DHODH is a quite complex matter being the target a mitochondrial enzyme: the inhibitors must be lipophilic (log*D*^7.4^ 2.5–3 range)^44^ in order to be able to transfer optimal activity at the enzymatic level to cellular efficacy, exposing them to solubility issues. Because of these issues, as can be observed from the recent literature,^46^ it is important to keep proposing new scaffolds as a source of optimized inhibitors. The SAR of MEDS433 has already been investigated identifying the biphenyl scaffold^39,47^ as a source of effective interaction with the lipophilic *h*DHODH subsite 1. Since the MEDS433 discovery, we focused our studies on the possibilities of replacing/improving the biphenyl substructure to improve pharmacokinetics and obtain more drug-like compounds. In our earlier studies, we already investigated compound **1**, which is characterized by a substituted diarylether.^38^ Although less potent than MEDS433, compound **1** (IC_50_ = 50 nM) proved the possibility of designing inhibitors where the biphenylic moiety is replaced by a diarylether scaffold. Being characterized by a higher log*D*^7.4^, compound **1** performed in a comparable manner to MEDS433 in proliferation and immunosuppression in cellular assays, recovering the weaker efficacy in inhibiting the enzyme by an improved efficacy in reaching the target, probably due to a better membrane permeability. Moreover, compound **1** was superior to MEDS433 in terms of cytotoxicity (>100 vs 60.4 μM to observe an effect ≥30%). Due to its safer profile, we started with this compound to continue the exploration of the SAR of this series of *h*DHODH inhibitors. Using an in silico/crystallographic approach, we designed a series of compounds (Figure 1) to specifically investigate the diarylether scaffold present in **1**. Early studies suggested that the introduction of methyl substituents in ring A of compound **1** ring A could stabilize the favorite binding conformation,^38^ leading to a more optimal interaction with the protein. We investigated the substituent role on the first ring A by compounds **2–10**, while rigid compounds **11–14** were then used to investigate the possibility of making this conformation more rigid, possibly improving its binding efficacy. On the other hand, with compounds **15–18**, we investigated the potential beneficial role of the pyridine substitution of phenyl ring A or B to insert a hydrophilic center in order to increase the solubility. With compound **19**, we investigated that the possibility of placing a substituent on the para position of ring B starting from compound **4** proved to be the most potent during the enzymatic assay.

All the compounds were investigated with in silico/crystallographic approaches and evaluated for their ability to inhibit *h*DHODH and the best of them was assayed for their differentiating/proapoptotic properties, with and without dipyridamole, to boost their performance and to optimize for potential in vivo applications and compared with the clinical trial lead brequinar.

## Results and Discussion

### Inhibition of *h*DHODH and SAR

We evaluated the recombinant *h*DHODH inhibition activity of compounds **2–19** using the clinical-trial candidate brequinar, synthesized following known procedures, MEDS433 and compound **1** for comparison. In order to complete the scenario and prepare the discussion of the following cell-based studies, Log*D*^7.4^ and solubility in PBS were also measured for the most potent compounds in the series (Table 1).

**Table 1:** Enzyme Inhibition by brequinar, MEDS433^38^, and Compounds **1**−**19**, with Relative LogD^7.4^ and IC_50_ Values Shown

compound	*h*DHODH^a^ IC50 ± SE(μM)	Log D^7.4^ ± SD^b^
Brequinar^38^	0.0018 ± 0.0003	1.83 ± 0.02
MEDS433^38^	0.0012 ± 0.0002	2.35 ± 0.02
**1**	0.050 ± 0.005	2.93 ± 0.0938
**2**(38)	0.48 ± 0.03	nd
**3**	0.40 ± 0.06	2.46 ± 0.05
**4**	0.0072 ± 0.0009	3.18 ± 0.09
**5**	0.114 ± 0.011	2.91 ± 0.07
**6**	5.2 ± 0.9	nd
**7**	>10	nd
**8**	3.9 ± 0.8	nd
**9**	2.209	nd
**10**	7.0 ± 1	nd
**11**	>10	0.45 ± 0.02
**12**	2.8 ± 0.4	2.89 ± 0.05
**13**	>10	2.55 ± 0.04
**14**	7.2 ± 1.6	1.59 ± 0.03
**15**	>10	nd
**16**	>10	nd
**17**	2.2 ± 0.4	1.76 ± 0.07
**18**	0.070 ± 0.011	2.51 ± 0.07
**19**	0.018 ± 0.004	>3

a *h*DHODH, in vitro assay.

b Measured by shake flask-method; “nd” indicates that the compound was not tested in that specific assay.

In lead compound **1**, the presence of two methyl groups in the first ring is crucial for the activity. We earlier proved by molecular dynamics (MD) studies how if left unsubstituted the first ring allows free rotation of the phenyl-*O*-phenyl dihedral angle inside subsite 1, leading to the conformational variability seen along the MD trajectories.^38^ We experimentally confirmed such behavior with compounds **2** and **3**, where only one methyl is present, respectively, in position 5 (compound **2**) or in position 2 (compound **3**). Both compounds, although still active in the low nM range, lose one log digit compared to **1**.

While retaining a double substitution on the first ring, with compounds **4** and **5**, we investigated the replacement of one methyl with an isopropyl moiety as the bulkier group. In this replacement, only position 2 seems to tolerate the bulkier substituent: while **5** loses two log digits compared to **1**, compound **4** is the best compound of the series reaching an IC_50_ of 7 nM. Focusing on position 2, with compounds **6–10**, we more deeply investigate the possibility to insert bulkier groups, observing a dramatic drop-in activity, with all the IC_50_ values of all the compounds in the μM range. Moving toward a different approach, compounds **11–14** were used to investigate the possibility of creating a rigid scaffold between rings A and B, with the aim of improving the binding efficacy. Unfortunately, we observed inactivity, with all the compounds IC_50_s in the μM range. We can speculate that a certain degree of flexibility is required to navigate the lipophilic patch in order to reach subsite 2. In lead **1**, the replacement of the substituted phenyl ring A with a bioisosteric pyridine resulted in losses of activity (**15** and **16**) as well as the replacement of the oxygen that bridges the two rings with sulfur (compound **17**). Starting from **4**, the best compound of the series (IC_50_ = 7.2 nM), it can be observed that while the replacement of CH in para with the more polar bioisostere nitrogen (compound **18**) resulted into a loss of one log digit activity (IC_50_ = 70 nM), its substitution with a CF_3_ group (compound **19**) was better tolerated (IC_50_ = 18 nM). Nevertheless, the bioisosteric nitrogen replacement produced a 20-fold increased solubility of compound **18** (111 μM, Table S4) with respect to the solubility of compound **4** (<6 μM).

It can be observed that the most interesting compounds of the series (**4**, **5**, **18**, and **19**) are characterized by log*D*^7.4^s values higher than those of brequinar or MEDS433 to guarantee the superior permeability until the mitochondrial lipophilic patch. In this sense, **18** is quite interesting because it shows a potent *h*DHODH activity (IC_50_ = 70 nM), almost 6-fold higher than that of teriflunomide (IC_50_ = 388 nM),^42^ the only *h*DHODH inhibitor approved so far (multiple sclerosis), and optimal log*D*^7.4^ (2.51) and solubility in PBS (111 μM).

### Binding Mode Analysis: Molecular Modeling and Crystallographic Studies

**Figure 2:**
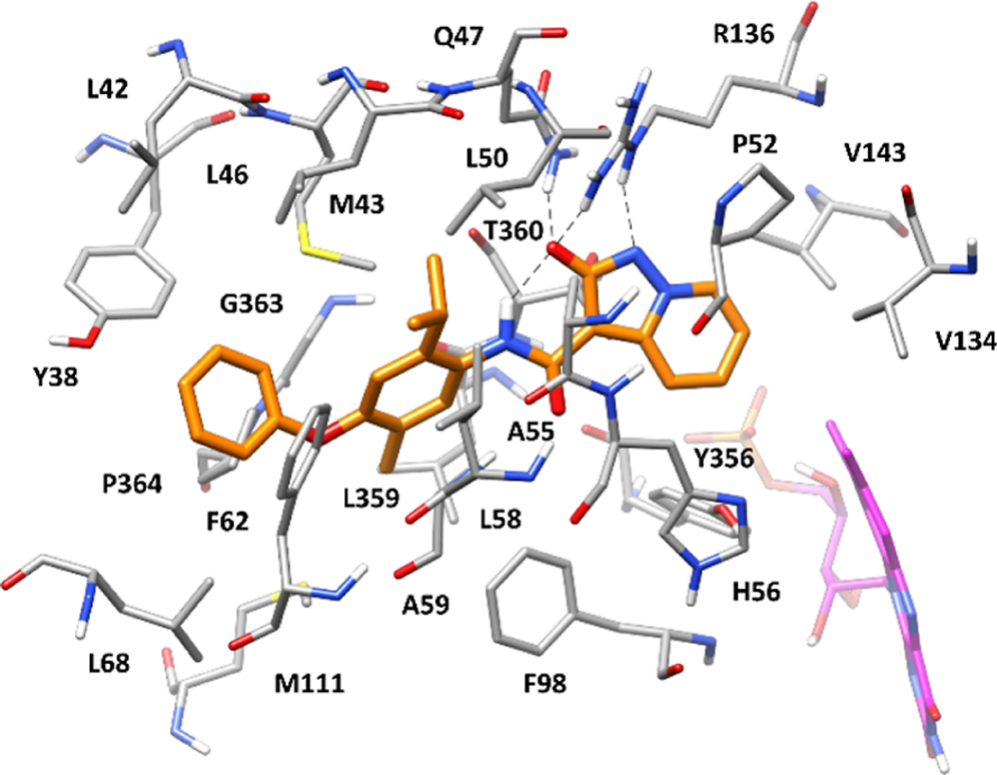
Energy-minimized average structure of compound **4** (orange) within *h*DHODH binding site (gray). Flavin mononucleotide is shown in magenta, while H-bonds are shown as black dashed lines. PDB IDs of crystal structures used as starting points for computational analysis is 6FMD.

In order to rationalize the SAR data, molecular modeling studies based on docking, molecular dynamics (MD) simulations, and binding free-energy evaluations were carried out. Compound **4**, which demonstrated to be the most potent *h*DHODH inhibitor of the series (IC_50_ 7.2 nM), was initially docked into the X-ray structure of *h*DHODH, with the aim of predicting its binding mode into the enzyme (see Material and Methods for details). The corresponding ligand–protein complex obtained by docking was then studied using a 50 ns MD simulation protocol, and this procedure was first evaluated on the reference *h*DHODH-MEDS433 crystallographic complex (PDB code 6FMD).^38^ As shown in Figure S1, both the ligand and the two cofactors (flavin mononucleotide and orotic acid) maintained their binding modes during the control MD simulation, showing an average root-mean-square deviation (rmsd) of about 0.4–0.5 Å from their position in the experimental structure. The validated MD protocol was subsequently used for studying the *h*DHODH-**4** complex obtained by docking. Figure 2 shows the predicted binding mode of the compound, which was found to be highly stable during the MD (with an average rmsd of ligand position of about 0.8 Å). As expected, the 2-hydroxypyrazolo[1,5-*a*]pyridine core of the ligand was predicted to interact with key residues through H-bonds and salt bridge interactions. In particular, the 2-hydroxypyrazolo[1,5-*a*]pyridine moiety, which is negatively charged under physiological conditions, is predicted to present a similar charge density on the oxygen and the geminal nitrogen (Figure S2), thus allowing the ligand to form a charge-assisted H-bond with the side chain of Q47, through its 2-hydroxypyrazolo[1,5-*a*]pyridine oxygen. Moreover, the ligand forms two different H-bonds with the positively charged guanidine moiety of R136 through both the geminal oxygen and nitrogen of its 2-hydroxypyrazolo[1,5-*a*]pyridine group that are maintained during almost the whole MD simulation, thus actually establishing a salt bridge interaction with R136. Additionally, a particularly stable intramolecular charge-assisted H-bond is observed between the amide NH group of the ligand and its negatively charged 2-hydroxypyrazolo[1,5-*a*]pyridine oxygen. Finally, the pyridine moiety of the core primarily forms hydrophobic interactions with P52, V134, V143, and T360. Ring A of the ligand is sandwiched between M43 and A59, while its isopropyl substituent is placed in a hydrophobic pocket mainly delimited by M43, L46, L50, A55, and L58, thus forming lipophilic interactions with these residues.

Finally, the methyl group connected to ring A makes hydrophobic contacts with F98, L359, and P364, whereas the terminal phenoxyl substituent interacts with Y38 and F62 through aromatic interactions and also makes contact with L68 and P364.

**Figure 3:**
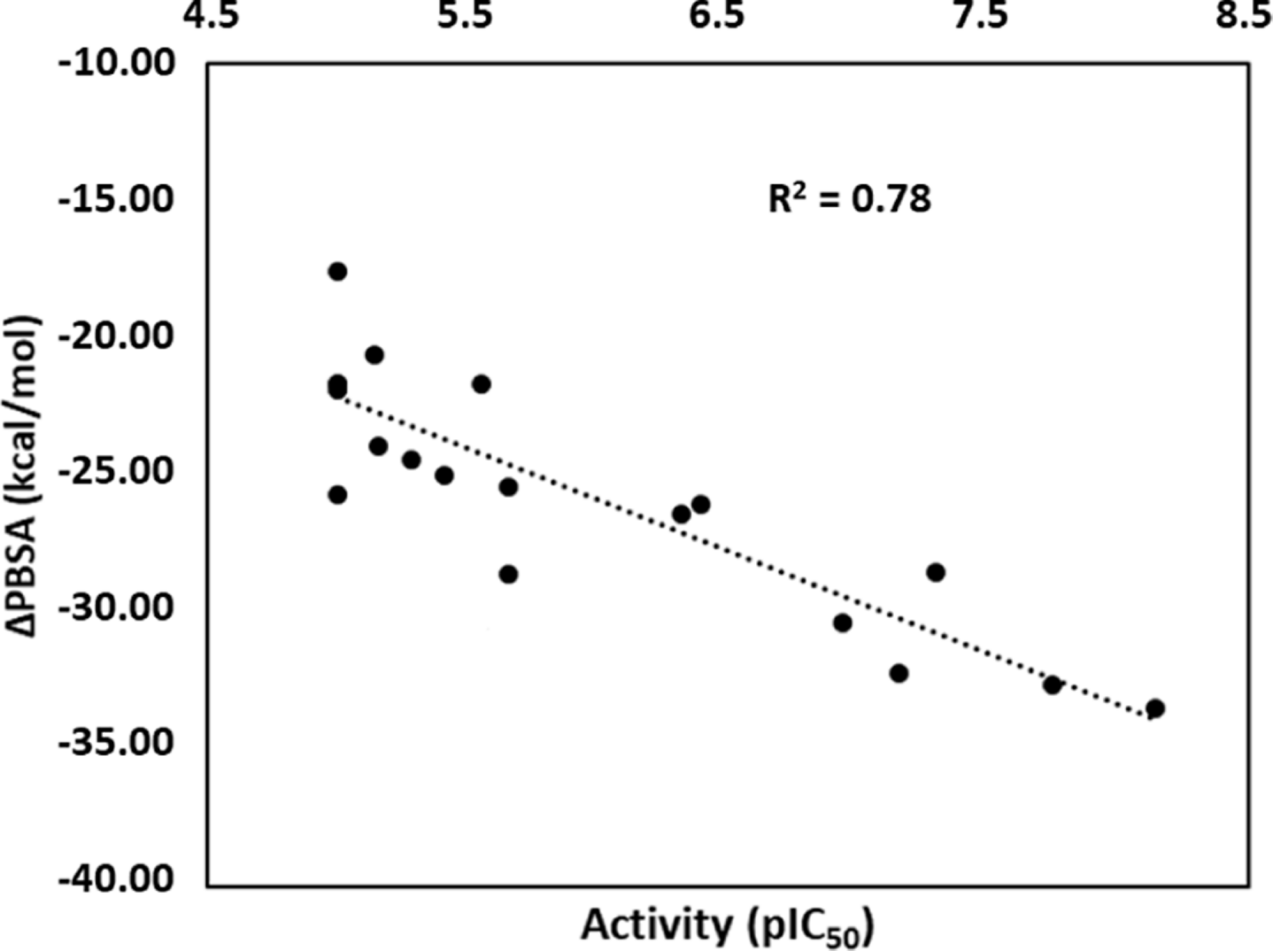
Correlation between compounds’ activity, expressed as pIC_50_, and binding energy estimated using the best MM-PBSA protocol (ε_i_ = 3), expressed in kcal/mol.

The docking/MD protocol was then applied to the whole series of 2-hydroxypyrazolo[1,5-*a*]pyridine derivatives in order to rationalize the SAR data experimentally obtained for these ligands. In addition, ligand–protein binding free-energy evaluations were also performed based on the results of the MD simulations obtained for each hDHODH-ligand complex. The predicted binding modes of the compounds, which all showed sufficiently stable conformations during the MD simulations (Table S1), thus allowing reliable energetic evaluations, were analyzed using the molecular mechanics–Poisson–Boltzmann surface area (MM-PBSA) approach (see Materials and Methods for details), seeking for a correlation between binding energies and experimental activity that could further validate the reliability of the computational protocol. Considering the high polarizability of both the ligands and the *h*DHODH binding site, various MM-PBSA protocols with different values of the internal dielectric constant (ε_int_) were evaluated with the aim of identifying the most suitable one. Figure 3 shows the correlation between compound activities and binding energies estimated using the best MM-PBSA protocol, with ε_int_ = 3, which showed a squared correlation coefficient of 0.78 (see also Tables S2 and S3).

The results confirmed the reliability of the binding modes predicted for the series of ligands and allowed possible interpretations of some of the SAR data. The highest binding energy (ΔPBSA = −33.48 kcal/mol) was predicted for compound **4**, which was the most potent ligand of the series. Our computational approach confirmed the importance of the double substitution of ring A of the ligands. In fact, most of the compounds sharing with compound **4** an alkyl-disubstituted central ring showed strong *h*DHODH inhibitory activity.

**Figure 4:**
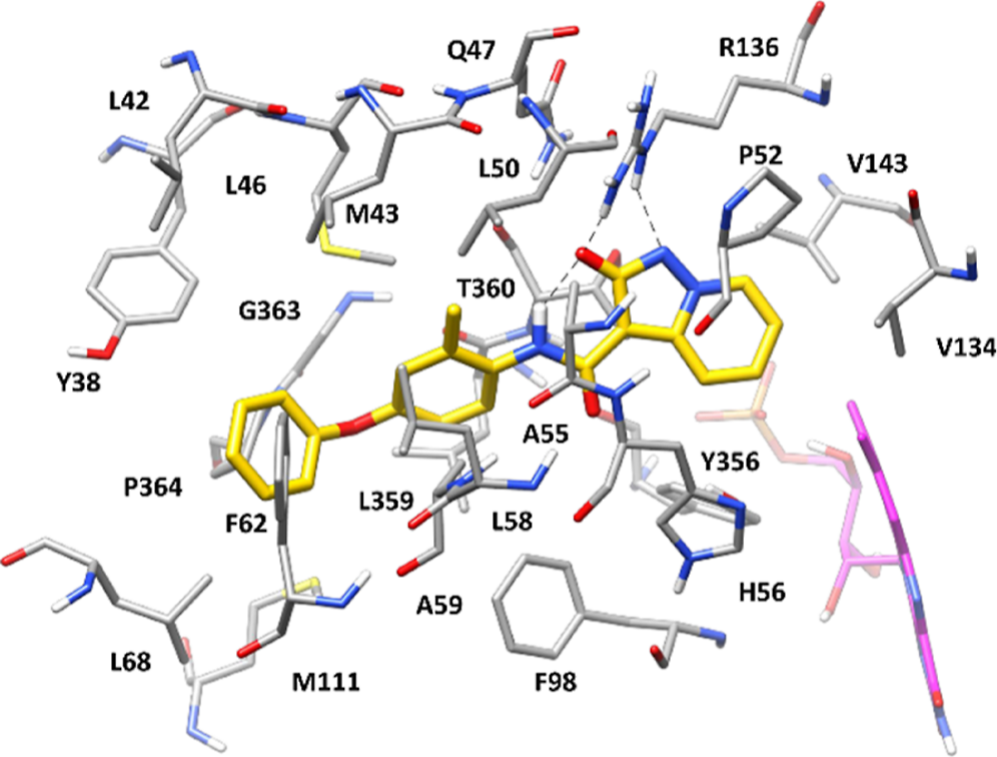
Minimized average structure of compound **3** (gold) within the *h*DHODH binding site (gray). Flavin mononucleotide is shown in magenta, while H-bonds are shown as black dashed lines. The PDB ID of crystal structures used as starting points is 6FMD.

Accordingly, these ligands were predicted to adopt a binding mode very similar to that described for compound **4**. Compounds **18** and **19**, which shared the same substitution pattern of ring A, showed similar ligand–protein interactions as compound **4**, and, accordingly, their predicted binding modes were associated with comparable values of binding free energies (Table S2). In contrast, compounds with a monoalkylated or unsubstituted central phenyl ring showed reduced potency, with IC_50_ values in the high nanomolar to micromolar range. The binding of these compounds was generally predicted to be less stable in the MD simulations, with an average ligand rmsd closer to or higher than 2.0 Å. For instance, compound **3**, with an IC_50_ value of 0.40 μM, showed an average rmsd of 2.3 Å of its position in the *h*DHODH binding site and was found to form weaker interactions with key anchoring residues of the enzyme, in particular, with Q47, which predominantly formed a H-bond with the backbone carbonyl of T360 during the MD (Figure 4). Also, considering the reduced interactions with the hydrophobic residues of the central portion of the enzyme catalytic site, such as L46, L50, L58, F98, L359, and P364, compared to compound **4**, the binding mode predicted for compound **3** may justify its lower inhibitory potency.

Nevertheless, the experimental data demonstrated that the replacement of the isopropyl group of compound **4** with bulkier alkoxy substituents in compounds **7**–**10** produced a strong decrease of activity, increasing the IC_50_ of the ligands to the micromolar range. Our molecular modeling studies suggested that these compounds adopt a different binding mode in which the central arylamide moiety of the ligands is rotated by about 180° degrees. This binding conformation may be induced by the limited size of the hydrophobic pocket constituted by M43, L46, L50, A55, and L58, in which the isopropyl group of compound **4** is predicted to be bound (Figure 2) and, most importantly, by its proximity to the highly polarized portion of the enzyme catalytic pocket in which R136 is located, which would prevent the pocket from binding too bulky and lipophilic chemical groups. Accordingly, compound **10** is predicted to interact with *h*DHODH in a conformation in which its 2-pentyloxy group is placed in the lipophilic pocket surrounded by L68, F98, M111, L359, and P364 (Figure S3), which is only partially occupied by the methyl substituent of the central phenyl ring of compound **4** (Figure 2), thus forming extensive lipophilic interactions with these residues. However, this also leads to the disruption of the ligand’s intramolecular H-bond, which negatively affects the stability of the binding conformation and results in an altered orientation of its 2-hydroxypyrazolo[1,5-*a*]pyridine core. In fact, although the negatively charged oxygen of **10** interacts with R136 for most of the MD simulation, the H-bond between this residue and the ligand pyrazolic nitrogen, as well as the interaction with Q47, is completely lost. In agreement with these results, the binding free energy estimated for compound **10** (ΔPBSA = −23.91 kcal/mol) was almost 10 kcal/mol lower than that evaluated for compound **4**. Finally, the weakest binding energy among all ligands of the series was predicted for compound **11 (**ΔPBSA = −17.57 kcal/mol). In the binding mode generated by our computational protocol, this ligand left the hydrophobic pocket constituted by M43, L46, L50, A55, and L58 substantially unoccupied and showed very poor interactions with Q47. In addition, due to its small size, compound **11** was unable to interact with the residues located at the terminal portion of the binding site, such as Y38, F62, and L68 (Figure S4).

Comparable binding modes were also observed for compounds **12–14**, characterized by the same (**12**) or similar (**13–14**) bicyclic moiety present in 11, which replaces ring A of compounds **1–10** and **15–19**. Therefore, the same considerations in the attempt to explain their low *h*DHODH inhibitory activity can be derived. The impossibility to occupy the hydrophobic pocket constituted by M43, L46, L50, A55, and L58 as well as the poor interactions established with both Q47 and the residues delimiting the second hydrophobic pocket (L68, F98, M111, L359, and P364) may justify the drop of activity compared to **4**. Moreover, although compounds **12–14** present a terminal phenyl ring, which allows potential interactions with the residues located at the terminal portion of the binding site (such as Y38, F62, and L68), its direct connection to the central bicyclic moiety may prevent the phenyl ring to assume the optimal orientation for interacting with such residues.

**Figure 5:**
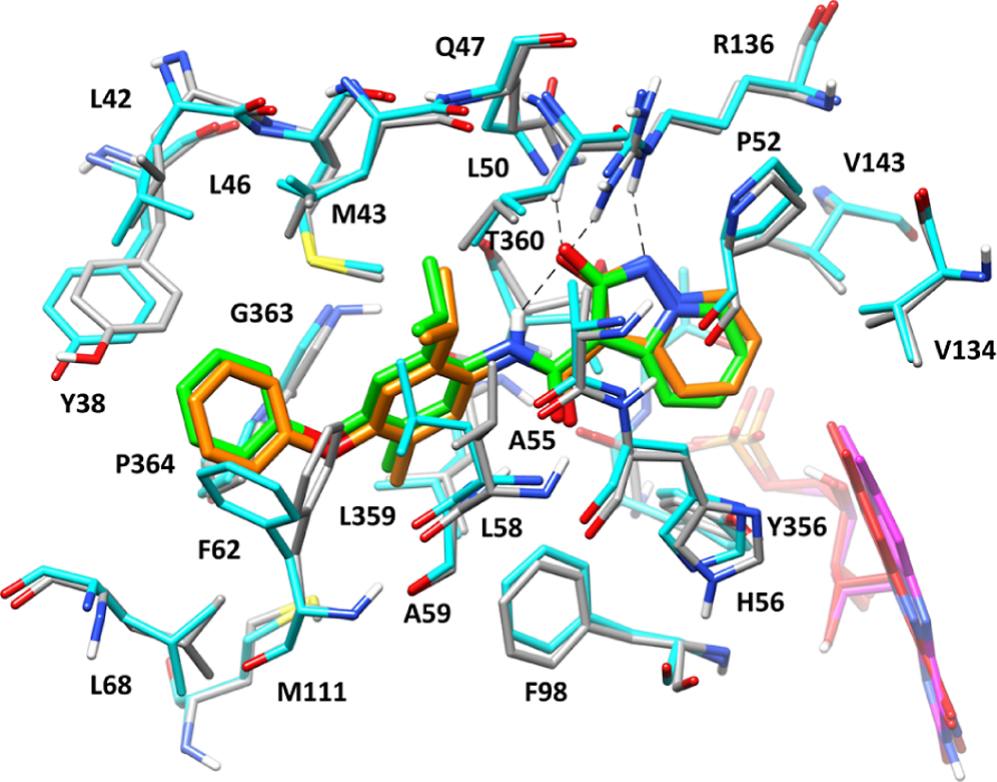
X-ray structure (PDB code 7Z6C) of *h*DHODH (cyan) in complex with compound **4** (green), superimposed with the binding mode of compound **4** (orange) within *h*DHODH (gray) predicted by molecular modelling studies. Flavin mononucleotide is shown in red and magenta in the X-ray structure and computational model, respectively.

With the aim of experimentally confirming the reliability of the molecular modeling protocol herein applied, as well as the interpretations of the SAR data based on the results of the computational studies, we determined the X-ray structure of *h*DHODH in complex with compound **4**, the most potent derivative of the series. As shown in Figure 5, the binding mode observed for compound **4** in its crystallographic complex with the enzyme was perfectly superimposable on that predicted by our computational investigation since all different structural moieties of the inhibitor were found to essentially assume the same position and orientation within the *h*DHODH binding site in both the model and the X-ray structure. Interestingly, most of the protein residues within the enzyme binding site showed the same orientation in the crystal structure and the model; in fact, all ligand–protein interactions predicted for compound **4** by our modeling studies were experimentally confirmed.

The only differences observed in the X-ray structure with respect to the modeled *h*DHODH-**4** complex, at the level of the enzyme binding site, concerned the orientation of the side chains of few solvent-exposed residues located close to the entrance of the binding site. In particular, the side chains of F62 and L58 showed an alternative spatial orientation with respect to the predicted model since they are both directed toward the hydrophobic gate formed by the side chains of Y38 and L68, instead of pointing toward L50. Nevertheless, F62 and L58 essentially maintained the interactions predicted with the terminal phenyl ring and the central isopropyl group of the ligand, respectively. In conclusion, the results of the crystallographic studies validated the binding mode predicted for compound **4** and strongly confirmed the reliability of the whole computational protocol applied on the series of new derivatives herein reported, thus also supporting the value of the structure-based interpretations of the SAR derived from the molecular modeling studies.

### Cell-Based Assays: Differentiation, Apoptosis, and Cytotoxicity

Based on their inhibitory activity on recombinant *h*DHODH in vitro, their Log*D*^7.4^, and their solubility, compounds **4**, **5**, **18**, and **19** were selected for the subsequent cellular assays and compared to brequinar, MEDS433, and **1**.

In particular, these compounds were tested for (i) their ability to induce differentiation and apoptosis on two AML cell lines (THP1 and U937) and (ii) their cytotoxicity on non-AML cells (Jurkat T-cells). Differentiation was investigated by assessing CD14 or CD11b expression in treated cells, while apoptosis was investigated with annexin V expression. The results are summarized in Table 2, while Figure S5 shows two exemplary differentiation plots (compounds **4** and **18**).

**Table 2:** Analysis of the Biological Activity (Enzymatic–Inhibitor Activity, Differentiation, Apoptosis, and Cytotoxicity) of Compounds **4**, **5**, **18**, and **19**, Compared to brequinar, MEDS433, and **1**

compound	*h*DHODH^a^ IC_50_ ± SE (nM)	differentiation EC_50_ THP1 (nM) (C.L. 95%)	apoptosis EC_50_ THP1 (nM) (C.L. 95%)	differentiation EC_50_ U937 (nM) (C.L. 95%)	apoptosis EC_50_ U937 (nM) (C.L.95%)	cytotoxicity (nM)(effect≥30% ± SD)
brequinar	1.8 ± 0.3	249	264	214	262	48000 ± 1000^38^
		(133–466)	(166–421)	(91–503)	(108–633)	
MEDS433	1.2 ± 0.2	40	72	26	40	60000 ± 1000^38^
		(21–77)	(42–124)	(6–104)	(24–68)	
**1**	50 ± 5	579	1344	755	2168	>100,000
		(170–1969)	(773–2278)	(266–2144)	(945–4974)	
**4**	7.2 ± 0.9	74	110	61	452	>100,000
		(51–108)	(60–203)	(40–153)	(130–865)	
**5**	114 ± 11	372	499	235	701	>100,000
		(140–943)	(185–1363)	(8–5655)	(156–2104)	
**18**	70 ± 11	1968	2646	2284	9820	75830 ± 10340
		(904–4652)	(1137–5912)	(1240–6243)	(2146–19620)	
**19**	18 ± 4	133	230	109	448	71320 ± 5250
		(86–206)	(143–366)	(64–188)	(309–632)	

a The differentiation and apoptotic data are expressed as EC_50_, and the cytotoxic effect was determined as the concentration that induced cytotoxicity in more than 30% of the cells.

**Figure 6:**
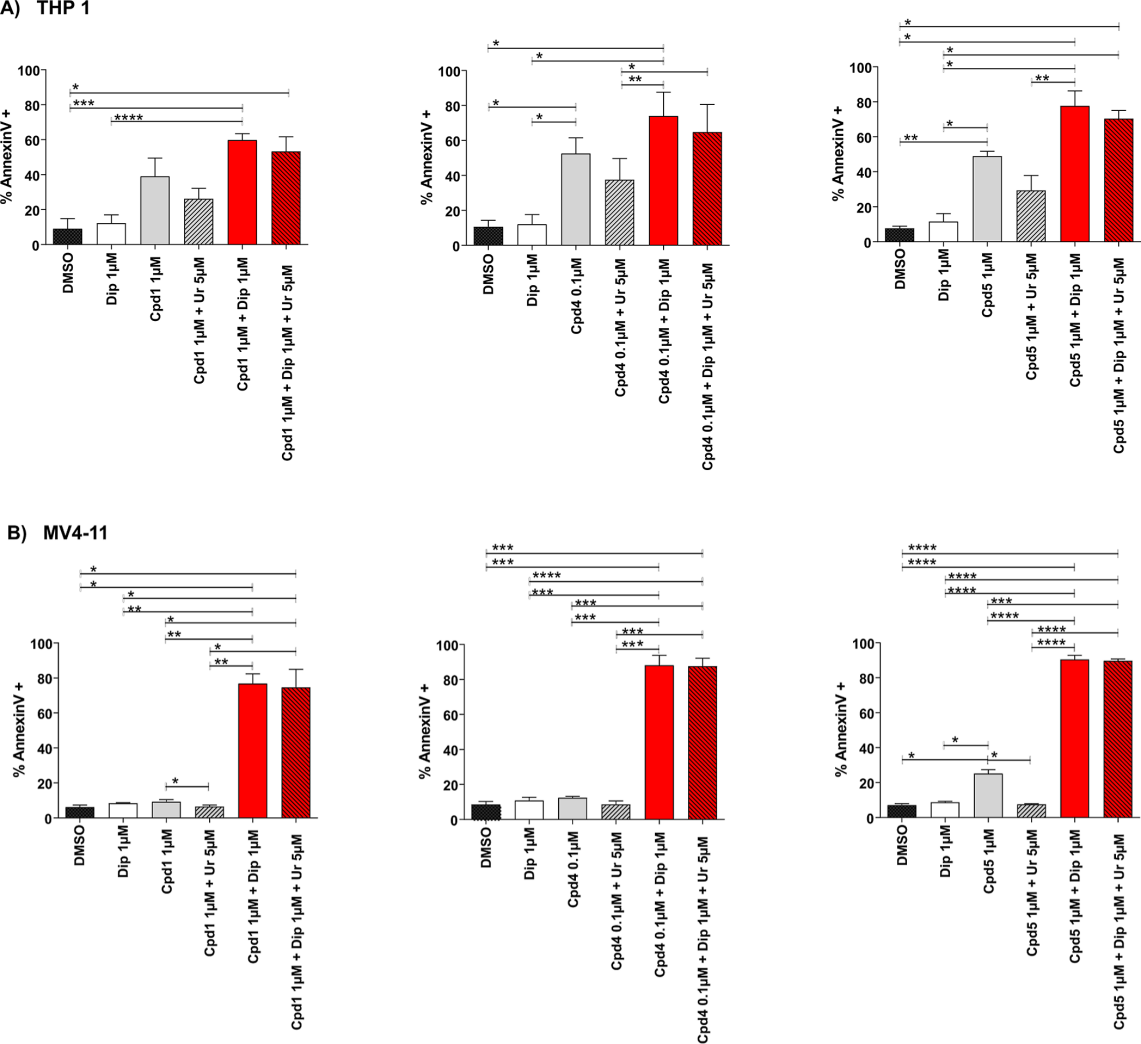
Combination of *h*DHODH inhibitors (**1**, **4**, and **5**) with dipyridamole results in synergistic effects. Analysis of the apoptotic rate induced by compounds **1**, **4**, and **5** on THP1 (*n* = 3 panel A) and MV4-11 (*n* = 3, panel B), when utilized alone or in the presence of uridine at low concentrations (5 μM). Compounds **1** and **5** were utilized at 1 μM, while **4** was utilized at 0.1 μM; apoptosis was evaluated after 3 days of treatment. DMSO: dimethyl sulfoxide. Cpd: compound. Dip: dipyridamole. Ur: uridine. Statistical significance: Anova/Tukey, *p < 0.05; **p < 0.01; ***p < 0.001; ****p < 0.0001.

As shown in Table 2, compound **4** had differentiation and apoptosis EC_50_ similar to, or better than, those of brequinar on AML cell lines, despite being 4 times less potent on the isolated enzyme. Similarly, compounds **5** and **19** had differentiation and apoptosis EC_50_ roughly comparable to that of brequinar, despite being, respectively, 63 and 10 times less potent on the isolated enzyme. This phenomenon is probably due to the higher Log*D*^7.4^ of **4**, **5**, and **19**, all approaching or exceeding 3. Compound **18**, which had been selected for its high solubility despite a medium-high Log*D*^7.4^, showed modest differentiation and apoptotic activity on AML cells, probably due to its low activity on the isolated enzyme, and for this reason, it was excluded from further analyses. In fact, as shown in Figure S5, compound **4** is able to induce a significant differentiating activity at a much lower concentration compared to compound **18**. However, it must be underlined that compound **18**, being quite soluble, could be the source of future repositioning in other *h*DHODH-related applications where the potency of the inhibitor is less compulsory than in the AML field, for example, immunosuppression. In summary, compounds **1**, **4**, and **5** showed pro-apoptotic and pro-differentiating abilities roughly comparable to those of brequinar with an extremely limited cytotoxicity on non-AML cells. Moving forward, we investigated the possibility to boost their antileukemic activity by combining **1**, **4**, and **5** with dipyridamole, an ENT1/2 blocker (Figure 6).

Dipyridamole was used at 1 μM based on previous dose finding analyses.^45^ Considering their EC_50_, compounds **1** and **5** were utilized at 1 μM, while compound **4** was utilized at 0.1 μM. At these concentrations, when used alone, the three compounds demonstrated a good pro-apoptotic activity on THP1 but limited activity on MV4-11, another AML cell line with a higher doubling time compared to THP1. Moreover, if human conditions were mimicked, that is, in the presence of physiological plasma uridine concentrations (5 μM), the antileukemic effects of these compounds were reduced or abolished (Figure 6, gray bar with line texture). When dipyridamole was added (Figure 6, red bars), the pro-apoptotic activity of the inhibitors was extremely enhanced, especially on MV4-11: in particular, the apoptotic rate increased to a minimum of 59.58 ± 4.08% (with **1** in THP1) and a maximum of 90.54 ± 2.27% (with **5** in MV4-11). More importantly, when human conditions were mimicked (with uridine 5 μM), the performances of the combinations were unaffected or just slightly reduced, predicting in vivo effectiveness of these associations (Figure 6, red bars with line texture). Finally, the synergism of this combination is not limited to apoptosis but rather extends to the differentiating effect (Figure S6). Please note that unlike other experiments, the differentiation analysis had to be performed on day 2 because on day 3, the apoptotic rate was too high and compromised the reliability of the results. For this reason, the results differ from Table 2.

It is possible to hypothesize that slowly proliferating AML cell lines, like MV4-11, would rely both on the pyrimidine salvage pathway and on the de novo synthesis, being poorly sensitive to the blocking of a single pathway but highly sensitive to the blocking of both pathways. Moreover, this combination approach could prevent a mechanism of resistance to *h*DHODH inhibitors, where cancer cells could leverage the salvage pathway to escape the *h*DHODH inhibitor-induced pyrimidine depletion. In conclusion, **1**, **4**, and **5** demonstrated to be effective against AML cell lines, especially in combination with dipyridamole, and could be characterized by a limited toxicity on non-AML cells.

### In Vivo Experiments

**Figure 7:**
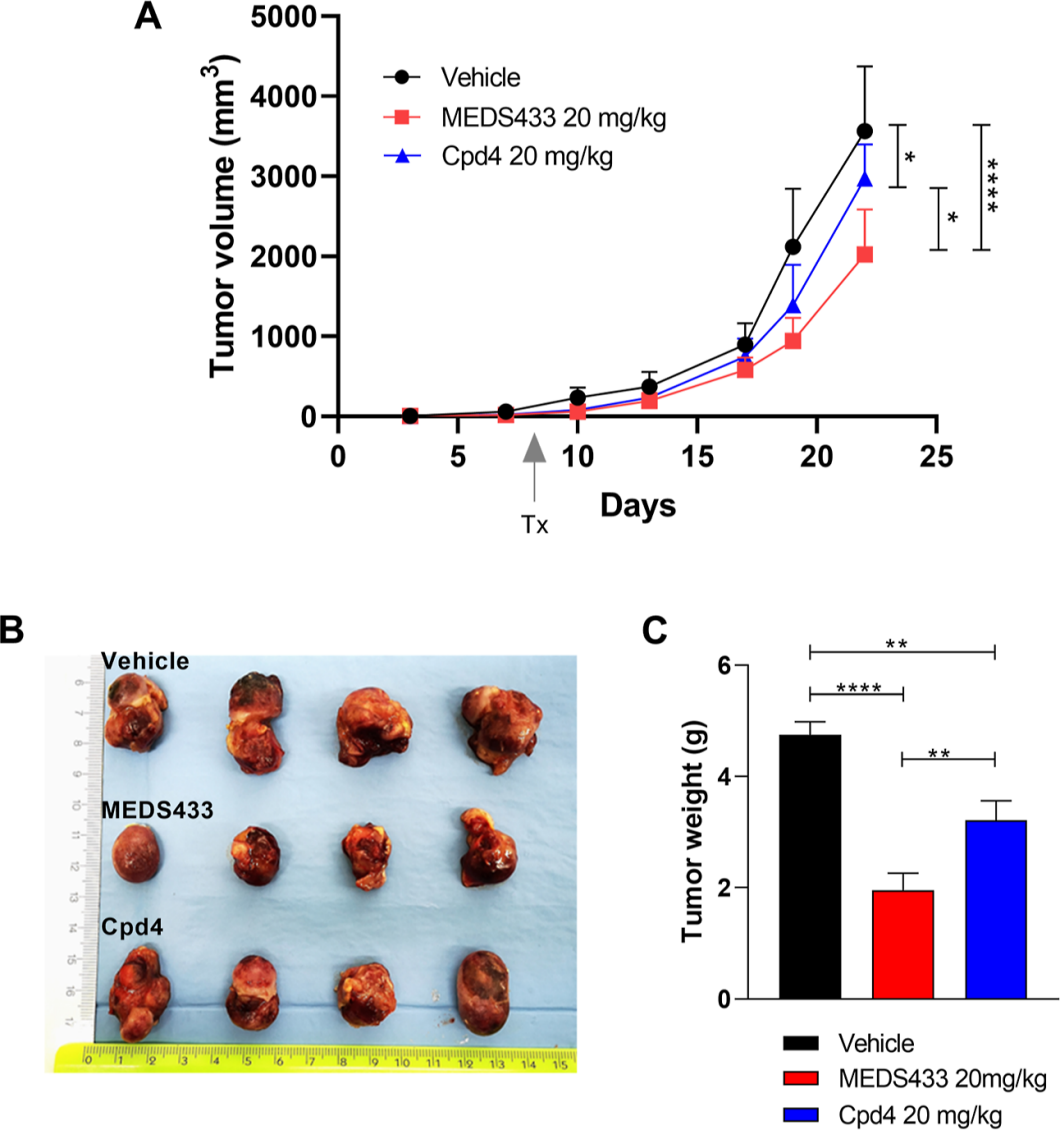
MEDS433 and compound **4** show in vivo antileukemic activity on an AML xenograft model. (A) Tumor volume measured twice weekly during the experiment. Tx indicates the beginning of treatment. (B) Macroscopic features of the excised tumors. (C) Final weight of the excised tumors. **Cpd4**: compound **4**. Statistical significance: Anova/Tukey, *p < 0.05; **p < 0.01.

MEDS433 showed a non-toxic in vivo profile when administered at doses of 10 and 25 mg/kg every 3 days for 5 weeks in Balb/c mice.^39^ Therefore, we decided to move forward testing it on a xenograft AML model where THP1 cells were injected subcutaneously in immunocompromised mice. MEDS433 was administered intraperitoneally at 20 mg/kg/die, starting from day 9; moreover, we also evaluated under the same conditions the in vivo activity of compound **4**, the best compound of this series characterized by in vitro strong antileukemic activity and a lower cytotoxicity than MEDS433. Both MEDS433 and compound **4** were able to significantly reduce the leukemic burden, in terms of both estimated tumor volume (Figure 7A) and tumor weight (Figure 7C), proving their efficacy under i.p. administration. Since MEDS433 is metabolically stable in vitro (98% of compound at 120 min), these data seem to reflect its activity at the cellular level (EC_50_ 72 nM). To complete the comparison between the two compounds, we decided to investigate the in vitro metabolic stability of **4** to better understand if its in vivo profile could be influenced by a metabolic weakness.

### In Vitro Metabolic Profile of 4

Therefore, we also characterized the in vitro metabolic profile of compound 4, being in vivo less active than MEDS433. Here, we characterize the major metabolic pathways responsible for the metabolism of compound 4 in vitro using rat-liver microsomes and therefore move the in vivo evaluation forward. The in vitro metabolic profiles of compound 4 were assessed using the following combination of methods: (C) incubation at 37 °C with active rat-liver microsomes and a regenerating system that slowly generated coenzyme units over the incubation time, leading to a better reproduction of in vivo behavior; (C1) incubation at 37 °C with heat-inactivated microsomes (via a 15 min heating cycle at 90 °C) and a regenerating system; (C2) incubation at 37 °C with microsomes without a regenerating system; and, finally, (B) incubation with the blank medium. SyGMa (Systematic Generation of potential Metabolites) software, a tool that lists predicted metabolites with associated empirical probability scores, was used to identify putative metabolites, which were then investigated by analyzing samples with liquid chromatography coupled to high-resolution mass spectrometry (HPLC-HRMS). For each series of samples (C, C1, and C2), incubation was stopped after 120 min (t 120) and intermediate samples were collected after 15, 30, and 60 min. The full-scan MS data acquired for all the samples were analyzed to find the *m*/*z* values of the predicted molecular structures. In order to exclude interfering signals, the results obtained were compared to blank samples and common background peaks were not considered. In sample C, we found for compound 4 peaks whose accurate mass data were in accordance with the monohydroxylated and dihydroxylated metabolites (Table 3). Moreover, as expected, we did not identify the same metabolites in samples C1 and C2, confirming the fundamental role of CYP450 in phase I metabolism.

**Table 3:**
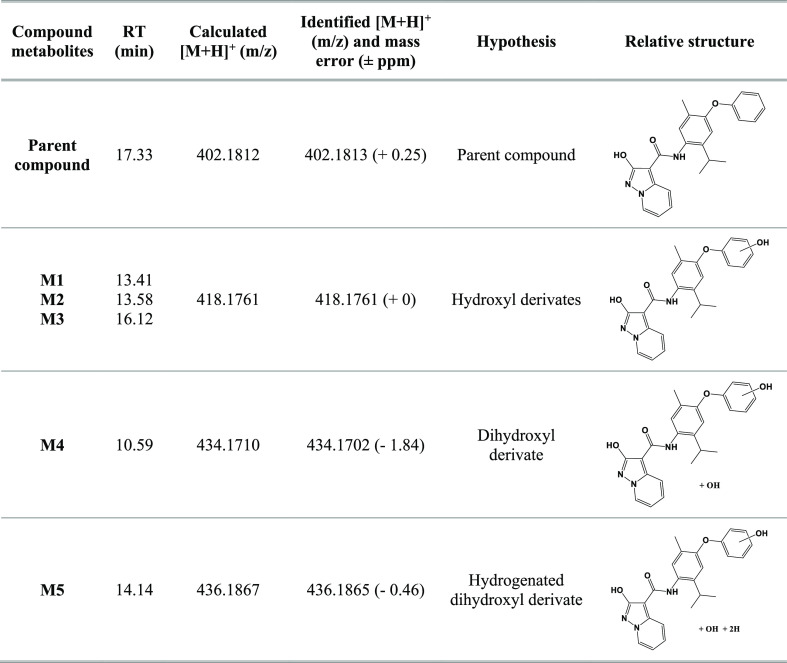
List of Metabolites of **4** with Chromatographic Retention Times, Calculated Accurate Masses (*m*/*z* M + H^+^), Identified Accurate Masses (*m*/*z* M + H^+^) in Samples, Chemical Formulas, and Structures

**Figure 8:**
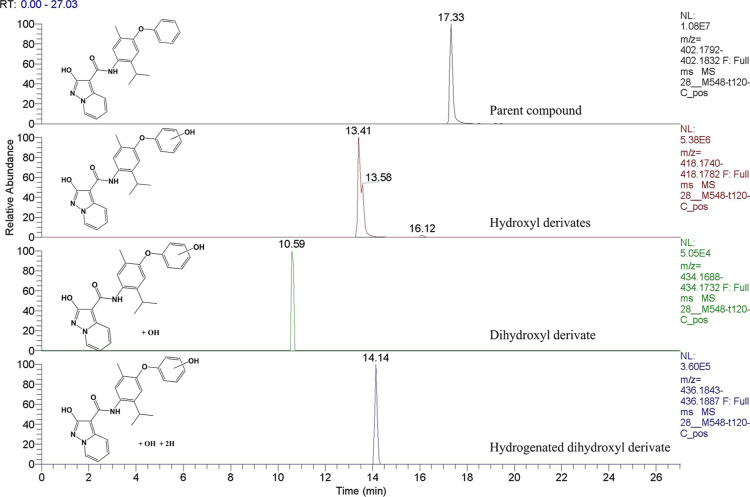
Extracted ion chromatograms of identified metabolites of **4** in sample C after incubation (time point 2 h).

We confirmed the structures of **4** and its metabolites that were found in sample C by interpreting the MS and MS2 spectra of each chromatographic run. Following the criteria proposed by Schymanski et al.,^48^ the metabolites identified are to be considered as “probable structures” (level 2b) or “tentative candidates” (level 3). Figure 8 reports extracted ion chromatograms for the putative metabolites. The different retention times of the hydroxy of **4** indicate that there may have been modifications to different parts of the molecule.

The interpretation of the fragmentation spectra, in both positive and negative ionization, shows that monohydroxylation occurs most probably on phenol ring C (Figures S9–S10). For instance, in the negative ion mode, for hydroxylated metabolites of compound **4**, we found the fragment ions at *m*/*z* 256 (rings B and C, after breaking at the CO–NH bond) but at *m*/*z* 240 for the parent compound. The MS2 spectra in the positive ion mode show that the hydroxyl cannot be on the isopropyl group because the main fragments are those originating from its loss (ion 376 for hydroxylated metabolites and 360 for the parent compound). However, it cannot be excluded that the hydroxyl can be inserted on the methyl group or another position of phenyl ring B.

The modifications involving the second hydroxylation and the hydrogenation on the hydrogenated-dihydroxylated metabolite occur most probably on the 3-hydroxypyrazol[1,5-*a*]pyridine ring A because ions 177 and 195 are present in its positive MS2 spectrum, while ion 161 is present on the parent compound and on the monohydroxylated metabolites (Figures S7–S11).

An examination of the results for compound **4**, which had undergone P-450-mediated biotransformation for an incubation period of 2 h, highlighted that compound **4** is relatively stable at short-term collection (89% of compound at 15 min), but it is not completely stable at 120 min when 53% of compound **4** was recovered. A study of compound **4** metabolic stability showed a weakness with the half-life around 120 min, inferior of those of MEDS433 (93% of the compound is still present at 120 min), and this could explain its lower efficacy in vivo compared to the lead MEDS433.

### Chemistry

#### Synthesis of the Target Compounds 2**–**20

We have already detailed the synthetic strategies used to produce the 2-aryloxypyrazolo[1,5-*a*]pyridine building blocks **20** and **31**,^38,39,49^ which are useful in the syntheses of target compounds **3–19**. Compounds **21–30** were prepared from the acyl chloride of acid **20,** obtained via treatment with oxalyl chloride and used directly after drying without further purification. Different from the MEDS433 series,^39^ the acyl chloride was allowed to react directly with the corresponding anilines **39**–**48** (the synthesis of anilines is described in the Supporting Information) without any form of activation using pyridine as a base/acyl transporter. The desired amides **21–30** were obtained in the 50–92% yield range. Moving forward, compounds **21–30** were then converted to desired target compounds **3–5, 11–16**, and **19** by applying room-pressure catalytic hydrogenation.

A similar approach was applied to the synthesis of compounds **6–10** and **17–18**, starting from acid **31** (see Scheme 2, the synthesis of anilines is described in the Supporting Information). The desired amides **32–38** were obtained in the 61–94% yield range. The projected synthetic pathway was not planned to be stereospecific, so in every precursor step, the racemic mixture was always obtained. ^1^H NMR spectra of initial compounds **34** and **36** show a peculiar pattern: due to the diastereotopic effect associated to the presence of a stereocentre, a geminal coupling between chemically non-equivalent methylene protons was observed (Figures S12–S14).

**Scheme 1:**
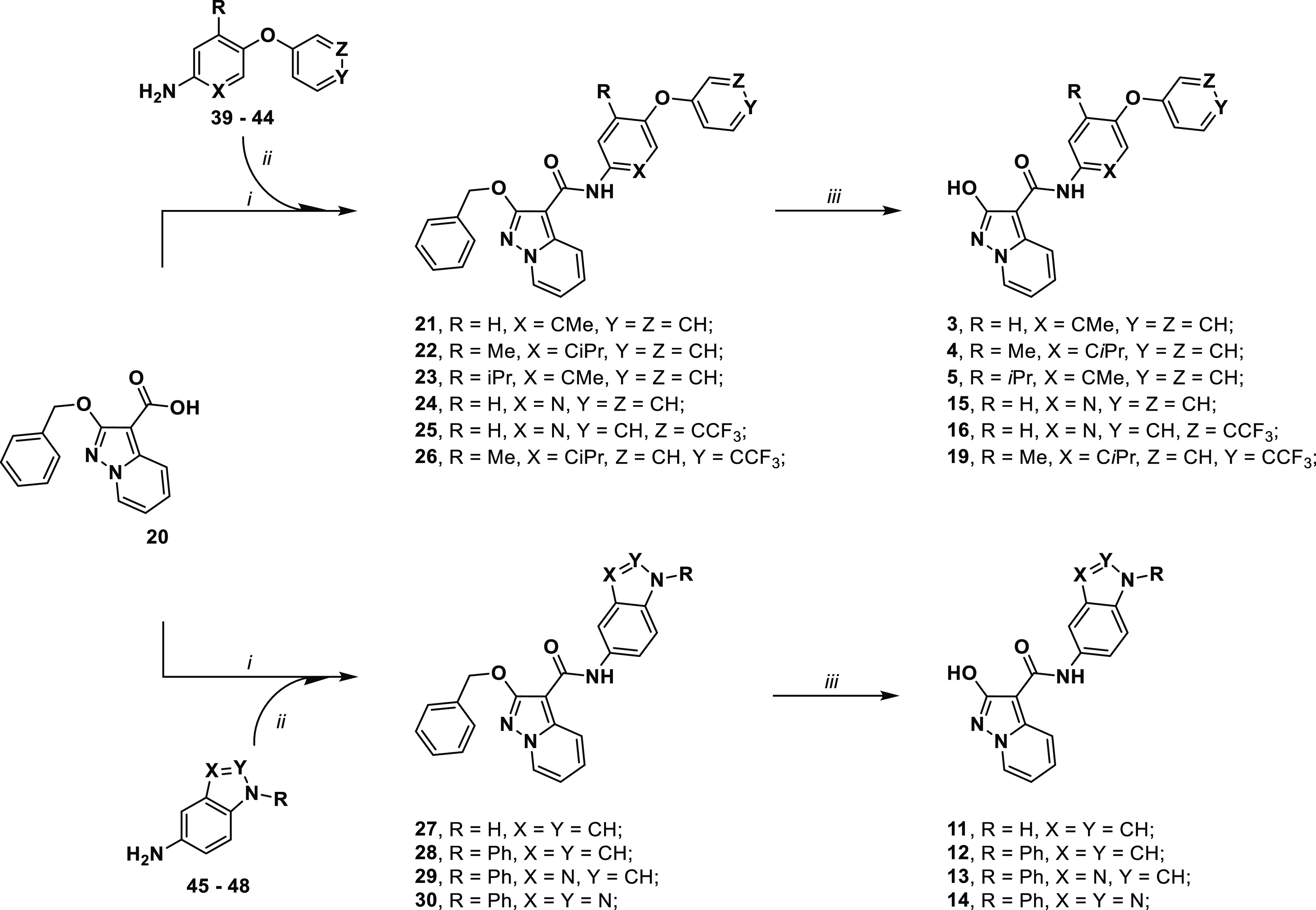
Synthetic Methodologies for the Synthesis of Targets **3–5**, **11–16**, and **19**: (i) Nitrogen Atmosphere, Oxalyl Chloride, Dry DMF, Dry THF; (ii) Dry Toluene, Dry Pyridine; Corresponding Aniline, r.t.; (iii) H_2_, Pd/C, Dry THF

**Scheme 2:**
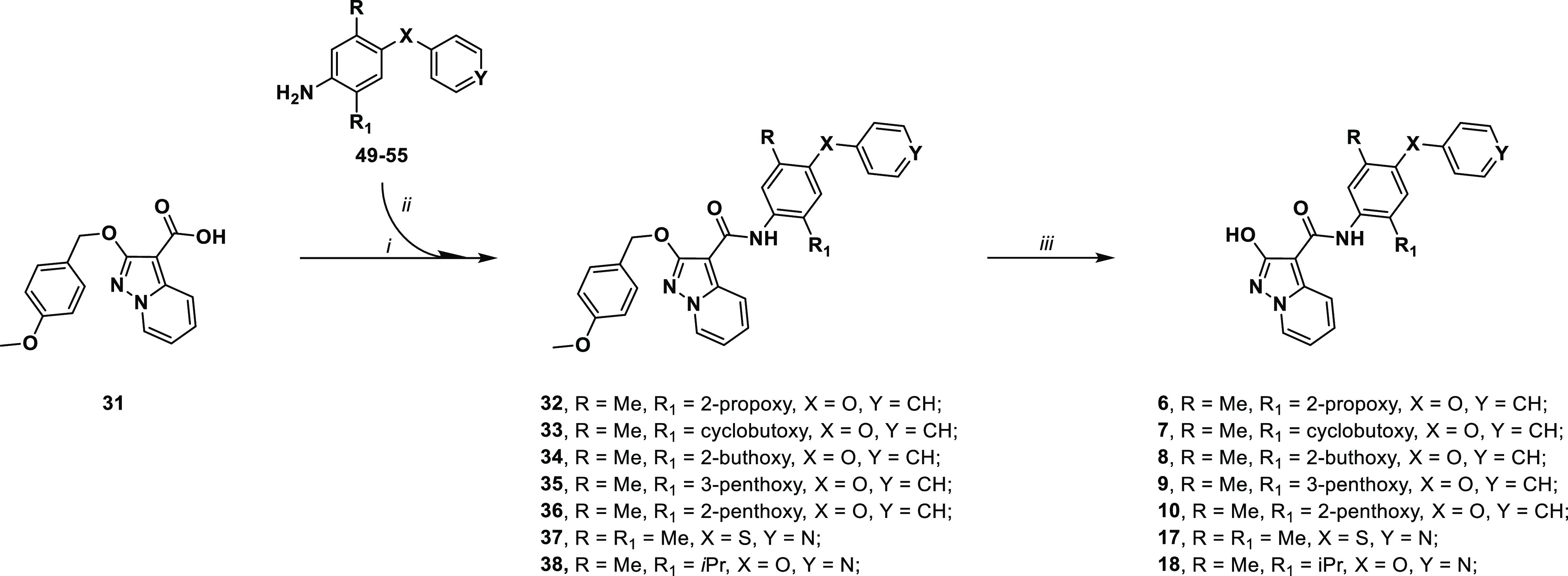
Synthetic Methodologies for the Synthesis of Targets 6–10 and 17–18: (i) Oxalyl Chloride, Dry DMF, Dry THF, Nitrogen Atmosphere; (ii) Dry Toluene, Dry Pyridine, Corresponding Aniline, r.t.; (iii) H_2_, Pd/C, Dry THF or Thioanisole, Trifluoroacetic Acid, 70 °C

Moving forward, the choice of a 4-methoxybenzyl protection the hydroxypyrazolo[1,5-*a*]pyridine moiety allowed us to investigate deprotection condition different from catalytic hydrogenation, in some occasion cause of issues. The 4-methoxybenzyl protecting group can be easily removed in acidic conditions, a protocol also applicable to molecules containing sulfur atoms and a pyridine ring, both known to poison the metal catalyst during hydrogenation. By treatment with trifluoroacetic acid (TFA) in the presence of thioanisole as a scavenger of compounds **32–38**, the desired targets **6–10** and **17**–**18** are obtained in 30–98% yield range.

## Conclusions

In this work, we keep focusing on investigating the SAR of a novel class of *h*DHODH inhibitors that are based on an unusual carboxylic group bioisostere 2-hydroxypyrazolo[1,5-*a*]pyridine. Starting from compound **1**, characterized by a diphenylether scaffold and a safety profile superior to brequinar’*s* Phase I/II, we identify compound **4**, which performed better than brequinar and better than lead **1** as a pro-differentiating and pro-apoptotic agent, recovering a weaker enzymatic potency by a higher log*D*^7.4^. While increasing its efficacy compared to **1**, it retained a better cytotoxicity profile than brequinar: CC_50_ > 100 μM for **4** versus 48 μM for brequinar. In our opinion, compound **4** is a good candidate because it presented in vivo efficacy on the xerograph model of AML. Although poor, its solubility of 6 μM is superior to that of Phase II BAY2402234 (pH 7, solubility <4 μM).^44^ A study of compound **4** metabolic stability showed a weakness with the half-life around 120 min, inferior of those of MEDS433, and this could explain its lower efficacy in vivo compared to the lead MEDS433; the in vivo activity on a xerograph model of AML was first released. Compound **4** represents a valuable new scaffold that could be a source of optimized compounds with low toxicity: the steps of removing its metabolic weakness and the increase of the solubility will be our next step in close future.

We can conclude that members of this class of *h*DHODH inhibitors as MEDS433 and **4** are characterized by a strong antileukemic activity and an optimal toxicity profile performing similar to that of other competitors that are already undergoing clinical trials. Moreover, the synergy between *h*DHODH inhibitors and hENT1/2 blockers was confirmed also with these new compounds, paving the way for in vivo applications.

## Experimental Section

### Chemistry

#### General Methods

All chemical reagents were obtained from commercial sources (Sigma Aldrich, Alfa Aesar, FluoroChem) and used without further purification. Analytical-grade solvents [acetonitrile, diisopropyl ether, diethyl ether, dichloromethane (DCM), dimethylformamide (DMF), ethanol 99.8% v/v, ethyl acetate (EtOAc), hexane, methanol (MeOH), petroleum ether b.p. 40–60 °C (petroleum ether), toluene] were used without further purification. When needed, solvents were dried over 4 Å molecular sieves. Tetrahydrofuran (THF) was distilled from Na and benzophenone under N_2_ immediately prior to use. Thin-layer chromatography (TLC) was conducted on silica gel on 5 × 20 cm plates at a 0.25 mm layer thickness. Anhydrous Na_2_SO_4_ was used as a drying agent for the organic phases. Compound purification was achieved either using flash column chromatography on silica gel (Merck Kieselgel 60, 230–400 mesh ASTM) and the eluents indicated in the procedures for each compound or using a CombiFlash Rf 200 (Teledyne Isco) with 5–200 mL/min, 200 psi (with an automatic injection valve), and RediSep Rf Silica columns (Teledyne Isco), with the eluents indicated in the procedures for each compound. Compounds synthesized in our laboratory generally varied between 90% and 99% purity.

Biological experiments were performed on compounds with a purity of at least 95%. Purity was checked using two ultra-high-performance liquid chromatography (UHPLC) analytical methods. HPLC analyses were performed on a UHPLC chromatographic system (Perkin Elmer, Flexar). The analytical columns were an UHPLC XSelect CSH Fluoro-Phenyl (3 × 75 mm, 2.5 μm particle size, Waters) and an XSelect HSS C18 column XP (3 × 75 mm, 2.5 μm particle size). Compounds were dissolved in methanol and injected through a 20 μL loop. The mobile phase consisted of methanol/water with 0.1% trifluoroacetic acid (ratio between 60/40 and 40/60, depending on the compound’s retention factor).

UHPLC retention times were obtained at flow rates of 0.5 mL/min, and the column effluent was monitored at 230, 254, 300, and 336 nm, referenced against a 400 nm wavelength. Melting points (mp) were measured on a capillary apparatus (Büchi 540). Final mp determination was achieved by placing the sample at a temperature that was 10 °C below the mp and applying a heating rate of 1 °C min^–1^. All compounds were routinely checked by ^1^H- and ^13^C NMR and mass spectrometry. The IR spectra of solid compounds were recorded by FT-IR (PerkinElmer SPECTRUM BXII, KBr dispersions) using the diffuse reflectance apparatus DRIFT ACCY. MS spectra were recorded on a Waters Micromass ZQ equipped with an ESCi source for electrospray ionization mass spectra. ^1^H- and ^13^C NMR spectra were performed on a JEOL ECZR600. The following abbreviations are used for coupling patterns: br = broad, s = singlet, d = doublet, dd = doublet of doublets, t = triplet, q = quartet, m = multiplet. Chemical shifts (δ) are given in parts per million (ppm). In this work, protons and carbons are labeled (a, b, c, d, e, f, g, h, l, m, n, o, p, q, r, and s) according to Scheme 1. Values marked with an asterisk (*, **, and ***) are interchangeable. For the final compounds **3–19**, HRMS spectra were recorded on an LTQ-Orbitrap XL Plus (Thermo Scientific, Bremen, Germany) mass spectrometer, equipped with an atmospheric pressure interface and an ESI ion source instrument. Compounds **21**, **32,** some anilines (Supporting Information), and key reactions were performed according to previously described procedures.^38,50−52^

##### General Procedure for the Synthesis of Pyrazolo[1,5-*a*]pyridine-Related Amides (**21–38**)

Oxalyl chloride (1.75 mL, 3.5 mmol) and dry DMF (7 μL) were added to a cooled (0 °C) solution of **21** (1.0 mmol) in dry THF (15 mL) under a nitrogen atmosphere. The reaction mixture was stirred for 2 h at room temperature under a nitrogen atmosphere and then concentrated under reduced pressure. The residue was dissolved in dry THF (10 mL), and the solution was again concentrated; this step was repeated three times. A solution of the appropriate aniline (**40**–**49**, 1.00 mmol) and dry pyridine (3.0 mmol) in dry toluene (5 mL) was added dropwise to the solution of the above acyl chloride in dry toluene (15 mL) kept under a nitrogen atmosphere. The resulting mixture was stirred at room temperature overnight and then cooled to room temperature and evaporated under reduced pressure.^53^ Only for compounds **21**–**23** and **26**, 0.5 M HCl (25 mL) was added after room cooling the toluene solution for quenching. The layers were resolved. The aqueous phase was further extracted with EtOAc (3 × 50 mL). The combined organic layers were washed with brine, dried over Na_2_SO_4_, and evaporated under reduced pressure. The crude material was purified using flash chromatography (details in each specific recipe).

##### 2-(Benzyloxy)-*N*-(2-methyl-4-phenoxyphenyl)pyrazolo[1,5-*a*]pyridine-3-carboxamide (**21**)

Obtained using aniline **39**. Flash chromatography eluent: petroleum ether/EtOAc 85/15 v/v. Light-yellow solid (mp 186.1–190.9 °C from diisopropyl ether). Yield 87%. ^1^H NMR (600 MHz, chloroform*-d*): δ 1.83 (s, 3H, Ar-C*H*_3_), 5.54 (s, 2H, −OC*H*_2_Ph), 6.76 (d, *J* = 2.2 Hz, 1H, *H*-*t*), 6.85–6.91 (m, 2H, aromatic protons), 6.96 (d, 2H, *J* = 8.1, aromatic protons), 7.05 (t, 1H, *J* = 7.3 Hz, *H*-*b*), 7.30 (t, 2H, *J* = 7.8 Hz, aromatic protons), 7.34–7.45 (m, 4H, aromatic protons and *H*-*c*), 7.53 (d, 2H, *J* = 6.3 Hz, aromatic protons), 8.20 (d, 1H, *J* = 8.8 Hz, *H*-*d*), 8.29–8.37 (m, 2H, aromatic protons), 8.44 (s, 1H, −NH); ^13^C NMR (151 MHz, chloroform*-d*): δ 17.6 (Ar-CH_3_), 72.6 (−O*C*H_2_Ph), 91.1 (*C-f*), 112.9 (*C-b*), 117.5 (*C-d*), 118.3, 119.0, 121.1, 122.8, 123.0, 127.6, 128.6, 129.0, 129.2, 129.4, 129.5, 129.7, 132.7, 135.4, 143.1 (*C-e*), 152.6 (*C-g*)*, 158.1 (*C-s*)*, 161.2 (*C-v*)*, 162.3 (*C-h*)*; IR (KBr) ν (cm^–1^): 3391, 3308, 3040, 2922, 2737, 1963, 1882, 1660, 1590, 1588, 1362, 1334, 1219, 1151, 1130, 1101; MS (ESI): 450 (M + 1).

##### 2-(Benzyloxy)-*N*-(2-isopropyl-5-methyl-4-phenoxyphenyl)pyrazolo[1,5-*a*]pyridine-3-carboxamide (**22**)

Obtained using aniline **40**. Flash chromatography eluent: petroleum ether/EtOAc 70/30 v/v. White solid (mp 166.2–167.7 °C; from trituration with diisopropyl ether). Yield 54%. ^1^H NMR (600 MHz, chloroform*-d*): δ 0.90 (d, 6H, *J* = 6.8 Hz, −CH(C*H*_3_)_2_), 2.16 (s, 3H, Ar-C*H*_3_), 2.72 (hept, 1H, *J* = 6.7 Hz, −C*H*(CH_3_)_2_), 5.55 (s, 2H, −OC*H*_2_Ph), 6.81 (s, 1H, H-t), 6.84 (d, 2H, *J* = 8.1 Hz, aromatic protons), 6.87 (t, 1H, *J* = 6.9 Hz, *H*-*b*), 6.98 (t, 1H, *J* = 7.3 Hz, aromatic proton), 7.22–7.28 (m, 2H, aromatic protons), 7.34–7.45 (m, 4H, aromatic protons), 7.52 (d, 2H, *J* = 6.9 Hz, aromatic protons), 8.02 (s, 1H, *H*-*q*), 8.31 (d, 1H, *J* = 6.8 Hz, *H*-*a*), 8.35 (d, 1H, *J* = 8.8 Hz, *H*-*d*), 8.48 (s, 1H, −N*H*); ^13^C NMR (151 MHz, chloroform*-d*): δ 16.1 (Ar-CH_3_), 22.7 (−CH(CH_3_)_2_), 27.8 (−CH(CH_3_)_2_), 72.5 (−O*C*H_2_Ph), 91.1 (*C-f*), 112.9 (*C-b*), 116.3, 117.9, 119.1 (*C-d*), 121.8, 126.4, 127.6 (*C–c*), 128.3, 128.6, 129.0 (*C-a*), 129.1, 129.2, 129.7, 131.5, 135.5, 138.4, 143.2 (*C-e*), 150.4 (*C-s*)*, 158.7 (*C-v*)*, 161.5 (*C-h*)**, 162.4 (*C-g*)**; IR (KBr) v (cm^–1^): 3398, 3040, 2963, 1652, 1636, 1528, 1490, 1445, 1402, 1368, 1289, 1220, 1181, 1147, 1127, 1044, 993; MS (ESI): 492 (M + 1).

##### 2-(Benzyloxy)-*N*-(5-isopropyl-2-methyl-4-phenoxyphenyl)pyrazolo[1,5-*a*]pyridine-3-carboxamide (**23**)

Obtained using aniline **41**. Flash chromatography eluent: petroleum ether/EtOAc 60/40 v/v. White solid (mp 157.5–158.9 °C; from trituration with diisopropyl ether). Yield 92%. ^1^H NMR (600 MHz, chloroform*-d*): δ 1.24 (d, 6H, *J* = 6.8 Hz, −CH(C*H*_3_)_2_), 1.77 (s, 3H, Ar-C*H*_3_), 3.20 (hept, 1H, *J* = 6.8 Hz, −C*H*(CH_3_)_2_), 5.54 (s, 2H, −OC*H*_2_Ph), 6.64 (s, 1H, *H-t*), 6.85–6.91 (m, 3H, aromatic protons and *H-b*), 7.01 (t, 1H, *J* = 7.3 Hz, *H-c*), 7.20–7.45 (m, 6H, aromatic protons), 7.52 (d, 2H, *J* = 6.4 Hz, aromatic protons), 8.29 (s, 1H, *H-q*), 8.32 (d, 1H, *J* = 6.8 Hz, *H-a*), 8.37 (d, 1H, *J* = 8.8 Hz, *H-d*), 8.47 (s, 1H, −N*H*); ^13^C NMR (151 MHz Chloroform*-d*): δ 17.1 (−*C*H(CH_3_)_2_), 23.2 (−CH(*C*H_3_)_2_), 27.4 (Ar-*C*H_3_), 72.6 (−O*C*H_2_Ph), 91.2 (*C-f*), 112.8 (*C-b*), 117.0, 119.1 (*C-d*), 120.2, 122.0, 122.1, 126.4, 127.5, 128.6, 128.9, 129.2, 129.4, 129.6, 133.6, 135.4, 138.8, 143.0 (*C-e*), 148.8 (*C-s*)*, 159.0 (*C-v*)*, 161.2 (*C-g*)**, 162.3 (*C-h*)**; IR (KBr) ν (cm^–1^): 3392, 3045, 2970, 1652, 1636, 1597, 1533, 1486, 1456, 1407, 1360, 1290, 1223, 1146, 1129, 1007, 911; MS (ESI): 492 (M + 1).

##### 2-Benzyloxy-*N*-(5-phenoxypyridin-2-yl)pyrazolo[1,5-*a*]pyridine-3-carboxamide (**24**)

Obtained using aniline **42**. Flash chromatography eluent: petroleum ether/EtOAc 80/20 v/v. White solid (mp 120.6–120.7 °C; from trituration with diisopropyl ether) Yield 87%. ^1^H NMR (600 MHz, chloroform*-d*): δ 5.55 (s, 2H, −OC*H*_2_Ph), 6.86–6.92 (m, 2H, aromatic proton and *H-b*), 7.10 (d, 2H, J = 7.9 Hz, aromatic protons), 7.17 (t, 1H, J = 7.4 Hz, *H-c*), 7.34–7.42 (m, 4H, aromatic protons), 7.45 (t, 2H, J = 7.3 Hz, aromatic protons), 7.53 (d, 2H, J = 7.2 Hz, aromatic protons), 8.01 (d, 1H, J = 2.6 Hz, aromatic proton), 8.25–8.30 (m, 2H, aromatic proton and *H-d*), 8.32 (d, 1H, J = 6.8 Hz, *H-a*), 8.64 (s, 1H, -N*H*); ^13^C NMR (151 MHz, chloroform-*d*): δ 72.4 (−O*C*H_2_Ph), 90.5 (*C-f*), 111.8, 113.1 (*C-b*), 118.8 (*C-d*), 120.7, 124.4, 127.9 (*C-c*), 128.3, 128.8 (*C-a*), 129.1, 129.2, 129.8, 131.4, 131.9, 135.6, 138.6, 143.0 (*C-e*), 155.0 (*C-s*)*, 159.6 (*C-v*)*, 161.4 (*C-g*)*, 162.3 (*C-h*)*; IR (KBr) *v* (cm^–1^): 3373, 3100, 3044, 2925, 1947, 1663, 1636, 1534, 1473, 1365, 1296, 1249, 1207, 1120, 1005. MS (ESI): 435 (M – 1).

##### 2-Benzyloxy-*N*-5-[3-(Trifluoromethyl)phenoxy]piridin-2-ylpyrazolo[1,5-*a*]pyridine-3-carboxamide (**25**)

Obtained aniline **43**. Flash chromatography eluent: petroleum ether/EtOAc 70/30 v/v). White solid (mp 133.6–135.9 °C; from trituration with diisopropyl ether). Yield 50%. ^1^H NMR (600 MHz, chloroform-*d*): δ 5.65 (s, 2H, −OC*H*_2_Ph), 6.89 (t, 1H, *J* = 6.7 Hz, *H-b*), 7.14 (dd, 1H, *J* = 8.2, 1.9 Hz, aromatic proton), 7.23 (s, 1H, aromatic proton), 7.33–7.46 (m, 7H, aromatic protons), 7.60 (d, 2H, *J* = 7.3 Hz, aromatic protons), 8.12 (d, 1H, *J* = 2.8 Hz, aromatic proton), 8.29 (d, 1H, *J* = 8.9 Hz, *H-d*), 8.31 (d, 1H, *J* = 6.9 Hz, *H-a*), 8.42 (*d*, 1H, *J* = 9.0 Hz, aromatic proton), 9.40 (s, 1H, −N*H*); ^13^C NMR (151 MHz, chloroform-*d*): δ 72.0 (−O*C*H_2_Ph), 90.7 (*C-f*), 113.1 (*C-b*), 114.6 (q, *J* = 3.8 Hz), 114.9, 118.8 (*C-d*), 119.8 (q, *J* = 3.8 Hz), 120.8, 123.8 (q, *J* = 272.0 Hz, −*C*F_3_), 127.9, 128.0, 128.6, 128.8, 128.9, 129.7, 130.6, 132.5 (q, *J* = 32.8 Hz), 135.9, 140.3, 143.2 (*C-e*), 148.3, 148.9 (*C-s*)*, 158.2 (*C-v*)*, 161.2 (*C-g*)*, 162.4 (*C-h*)*; IR (KBr) v (cm^–1^): 3373, 3069, 2924, 2853, 1666, 1634, 1538, 1449, 1328, 1287, 1163, 1130, 1012. MS (ESI): 505 (M + 1).

##### 2-(Benzyloxy)-*N*-(2-isopropyl-5-methyl-4-(4-(trifluoromethyl)phenoxy)phenyl)pyrazolo[1,5-*a*]pyridine-3-carboxamide (**26**)

Obtained using aniline **44**. Flash chromatography: eluent petroleum ether/EtOAc 85/15 v/v. White solid (186.2–187.3 °C from diisopropyl ether). Yield 95%. ^1^H NMR (600 MHz chloroform*-d*): δ 0.91 (d, 6H, *J* = 6.8 Hz, −CH(C*H*_*3*_)_2_), 2.14 (s, 3H, Ar-C*H*_*3*_), 2.73 (hept, 1H, *J* = 6.8 Hz, −C*H*(CH_3_)_2_), 5.56 (s, 2H, −OC*H*_*2*_Ph), 6.82 (s, 1H, *H-t*), 6.87–6.91 (m, 3H, aromatic protons and *H-b*), 7.36–7.46 (m, 4H, aromatic protons), 7.49–7.56 (m, 4H, aromatic protons), 8.10 (s, 1H, *H-q*), 8.33 (d, 1H, *J* = 6.9 Hz, *H-a*), 8.35 (d, 1H, *J* = 8.8 Hz, *H-d*), 8.53 (s, 1H, −N*H*); ^13^C NMR (151 MHz, chloroform-*d*): δ 16.0 (Ar-*C*H_3_), 22.7 (−CH(*C*H_3_)_2_), 27.8 (−*C*H(CH_3_)_2_), 72.6 (−O*C*H_2_Ph), 91.0 (*C-f*), 112.9 (*C-b*), 115.9, 118.2 (*C-d*), 119.0, 123.8 (q, *J* = 33.2 Hz), 124.5 (q, *J* = 270.9, −*C*F_3_), 126.4, 127.2 (q, *J* = 3.7 Hz), 127.7, 128.4, 128.7, 129.0, 129.2, 129.3, 132.3, 135.5, 138.5, 143.2 (*C-e*), 149.2 (*C-s*), 161.4 (*C-v*)*, 161.5 (*C-g*)*, 162.4 (*C-h*)*; IR (KBr) *ν* (cm^–1^): 3403, 3084, 3043, 2976, 2891, 1655, 1638, 1613, 1578, 1543, 1511, 1501, 1477, 1446, 1401, 1360, 1330, 12090, 1240, 1215, 1148, 1120, 1064, 1042, 994; MS (ESI): 560 (M + 1).

##### 2-(Benzyloxy)-*N*-(1H-indol-5-yl)pyrazolo[1,5-*a*]pyridine-3-carboxamide (**27**)

Obtained using aniline **45**. Flash chromatography eluent: petroleum ether/EtOAc 60/40 v/v. Light-brown solid (mp 225.4–228.0 °C; from trituration with diisopropyl ether). Yield 74%. ^1^H NMR (600 MHz, DMSO-*d*_6_): δ 5.60 (s, 2H, −OC*H*_2_Ph), 6.38 (s, 1H, aromatic proton), 7.03 (t, 1H, *J* = 6.9 Hz, *H-b*), 7.11 (dd, 1H, *J* = 8.6, 1.6 Hz, aromatic proton), 7.30–7.34 (m, 2H, aromatic protons), 7.41 (t, 1H, *J* = 7.4 Hz, *H-c*), 7.47 (t, 2H, *J* = 7.5 Hz, aromatic protons), 7.50–7.54 (m, 2H, aromatic protons), 7.65 (d, 2H, *J* = 7.4 Hz, aromatic protons), 7.88 (s, 1H, aromatic proton), 8.13 (d, 1H, *J* = 8.8 Hz, *H-d*), 8.69 (d, 1H, J = 6.8 Hz, *H-a*), 8.82 (s, 1H, −N*H*), 11.03 (*s*, 1H, −N*H indole*); ^13^C NMR (151 MHz, DMSO-*d*_6_): δ 71.5 (−O*C*H_2_Ph), 90.4 (*C-f*), 101.1, 110.6, 111.4, 113.1 (*C-b*), 114.8, 117.5 (*C-d*), 126.1, 127.6, 128.1, 128.2, 128.5, 128.7, 129.4, 130.6, 132.8, 136.3, 141.9 (*C-e*), 160.0 (*C-g*)*, 161.6 (*C-h*)*. MS (ESI): 383 (M + 1).

##### 2-(Benzyloxy)-*N*-(1-phenyl-1*H*-indol-5-yl)pyrazolo[1,5-*a*]pyridine-3-carboxamide (**28**)

Obtained using aniline **46**. Flash chromatography eluent: petroleum ether/EtOAc 60/40 v/v. Light-brown solid (mp 182.7–183.5 °C; from trituration with diisopropyl ether) Yield 74%. ^1^H NMR (600 MHz, chloroform-*d*): δ 5.59 (s, 2H, −OC*H*_2_Ph), 6.63 (d, 1H, *J* = 3.1 Hz, aromatic proton), 6.85 (td, 1H, *J* = 6.9, 1.4 Hz, *H-b*), 7.17 (dd, 1H, *J* = 8.8, 2.0 Hz, aromatic proton), 7.31–7.53 (m, 11H, aromatic protons and *H-c*), 7.58 (d, 2H, *J* = 7.3 Hz, aromatic protons), 8.07 (d, 1H, *J* = 1.9 Hz, aromatic proton), 8.30 (d, 1H, *J* = 6.8 Hz, *H-a*), 8.37 (d, 1H, *J* = 8.9 Hz, *H-d*), 8.78 (s, 1H, −N*H*); ^13^C NMR (151 MHz, chloroform-*d*): δ 72.2 (−O*C*H_2_Ph), 91.3 (*C-f*), 103.9, 110.7, 112.2, 112.7, 116.2, 119.0, 124.2, 126.4, 127.4, 128.2, 128.6, 128.9, 129.0, 129.75, 129.79, 132.0, 132.9, 136.0, 140.0, 143.0 (*C-e*), 161.3 (*C-g*)*, 162.2 (*C-h*)*. MS (ESI): 459 (M + 1).

##### 2-(Benzyloxy)-*N*-(1-phenyl-1*H*-benzo[d]imidazole-5-yl)pyrazolo[1,5-*a*]pyridine-3-carboxamide (**29**)

Obtained using aniline **47**. Flash chromatography eluent: DCM/MeOH 95/5 v/v. Pale-pink solid (mp 196.4–197.5 °C; from trituration with diisopropyl ether) Yield 67%. ^1^H NMR (600 MHz, chloroform-*d*): δ 5.29 (s, 1H, aromatic proton), 5.59 (s, 2H, −OC*H*_2_Ph), 6.87 (t, 1H, *J* = 6.8 Hz, *H-b*), 7.35–7.60 (m, 11H, aromatic protons), 7.63 (d, 1H, *J* = 8.7 Hz, aromatic proton), 7.94 (s, 1H, aromatic proton), 8.10 (s, 1H, aromatic proton), 8.31 (d, 1H, *J* = 6.8 Hz, *H-a*), 8.35 (d, 1H, *J* = 8.8 Hz, *H-d*), 8.84 (s, 1H, −N*H*); ^13^C NMR (151 MHz, chloroform-*d*): δ 72.3 (−O*C*H_2_Ph), 91.1 (*C-f*), 110.5, 111.4, 112.9 (*C-b*), 117.6, 119.0 (*C-d*), 124.0, 127.6, 128.1, 128.3, 128.7, 129.0, 129.1, 130.2, 130.4, 134.5, 135.9, 136.5, 142.9, 143.0 (*C-e*), 144.5, 161.4 (*C-g*)*, 162.3 (*C-h*)*. MS (ESI): 460 (M + 1).

##### 2-(Benzyloxy)-*N*-(1-phenyl-1*H*-benzo[d][1,2,3]triazol-5-yl)pyrazolo[1,5-*a*]pyridine-3-carboxamide (**30**)

Obtained using aniline **48**. Flash chromatography eluent: DCM/MeOH 95/5 v/v. Pale-brown solid (mp 199.0–199.9 °C; from trituration with diisopropyl ether) Yield 90%. ^1^H NMR (600 MHz, chloroform-*d*): δ 5.61 (s, 2H, −OC*H*_2_Ph), 7.06 (t, 1H, *J* = 6.8 Hz, *H-b*), 7.40 (t, 1H, *J* = 7.3 Hz, *H-c*), 7.47 (t, 2H, *J* = 7.5 Hz, aromatic protons), 7.53–7.72 (m, 7H, aromatic protons), 7.85–7.92 (m, 3H, aromatic protons), 8.12 (d, 1H, *J* = 8.8 Hz, *H-d*), 8.59 (s, 1H, aromatic proton), 8.70 (d, 1H, *J* = 6.8 Hz, *H-a*), 9.23 (s, 1H, −N*H*); ^13^C NMR (151 MHz, chloroform-*d*): δ 71.5 (−O*C*H_2_Ph), 90.0 (*C-f*), 107.6, 111.3(*C-b*), 113.5, 117.4 (*C-d*), 122.5, 128.1, 128.4, 128.5, 128.6, 128.7, 128.8, 129.6, 130.1, 135.8, 136.3, 136.4, 142.0 (*C-e*), 146.3, 160.6 (*C-g*)*, 161.9 (*C-h*)*. MS (ESI): 461 (M + 1).

##### General Procedure for the Synthesis of Pyrazolo[1,5-*a*]pyridine-Related Amides **32–36**

Oxalyl chloride (1.75 mL, 1.5 mmol) and dry DMF (10 μL) were added to a cooled (0 °C) solution of **31** (1.2 mmol) in dry THF (15 mL), kept under a nitrogen atmosphere. The reaction mixture was stirred for 2 h at room temperature under a nitrogen atmosphere. The solution was concentrated under reduced pressure, and the residue was dissolved in dry THF (10 mL); this step was repeated three times. A solution of the appropriate aniline (1.00 mmol) and dry pyridine (3.60 mmol) in dry toluene (5 mL) was added to the solution of the above acyl chloride in dry toluene (10 mL), kept under a nitrogen atmosphere. The resulting mixture was stirred at room temperature overnight and then quenched with 0.5 M HCl (25 mL). The layers were resolved, the aqueous phase was further extracted with EtOAc (3 × 50 mL), and the combined organic layer was dried and evaporated under reduced pressure. The crude material was purified using flash chromatography.

##### 2-(Benzyloxy)-*N*-(2-isopropoxy-5-methyl-4-phenoxyphenyl)pyrazolo[1,5-*a*]pyridine-3-carboxamide (**32**)

Obtained using aniline **49**. The crude product was purified by flash chromatography (eluent: petroleum ether/EtOAc 85/15 v/v) to afford the title compound as a white solid (melting point 126.0–126.9 °C from diisopropyl ether). Yield 90%.^1^H NMR (600 MHz, chloroform-*d*): δ 1.12 (d, 6H, *J* = 6.1 Hz, −CH(C*H*_3_)_2_), 2.17 (s, 3H, Ar-C*H*_*3*_), 4.36 (h, 1H, *J* = 6.1 Hz, −C*H*(CH_3_)_2_), 5.67 (s, 2H, −OC*H*_2_Ph), 6.55 (s, 1H, *H-t*), 6.83 (t, 1H, *J* = 6.8 Hz, *H-b*), 6.88 (d, 2H, *J* = 8.1 Hz, *H-n*), 7.0 (t, 1H, *J* = 7.3 Hz, aromatic proton), 7.25–7.41 (m, 6H, aromatic protons), 7.53 (d, 2H, *J* = 7.5 Hz, aromatic protons), 8.26 (d, 1H, *J* = 6.8 Hz, *H-a*), 8.35 (d, 1H, *J* = 8.9 Hz, *H-d*), 8.48 (s, 1H, *H-q*), 9.20 (*s*, 1H, −N*H*); ^13^C NMR (151 MHz, chloroform-*d*): δ 15.8 (Ar-*C*H_3_), 21.9 (−CH(*C*H_3_)_2_), 71.5 (−*C*H(CH_3_)_2_), 71.6 (−O*C*H_2_Ph), 91.6 (*C-f*), 106.7, 112.7 (*C-b*), 116.2, 119.0 (*C-d*), 121.8, 122.5, 122.9, 126.3, 127.4, 127.9, 128.4, 128.6, 128.8, 129.7, 136.4, 143.2 (*C-e*), 145.7, 148.6, 158.8, 161.3, 162.3. MS (ESI): 538 (M – 1).

##### *N*-(2-Cyclobutoxy-5-methyl-4-phenoxyphenyl)-2-((4-methoxybenzyl)oxy)pyrazolo[1,5-*a*]pyridine-3-carboxamide (**33**)

Obtained using aniline **50**. The crude product was purified by flash chromatography (eluent: petroleum ether/EtOAc 85/15 v/v) to afford the title compound as a sticky solid. Yield 79%.^1^H NMR (600 MHz, chloroform-*d*): δ 1.46–1.56 (m, 1H, cyclobutoxy proton), 1.59–1.68 (m, 1H, cyclobutoxy proton), 1.80–1.91 (m, 2H, cyclobutoxy proton), 2.15 (s, 3H, Ar-C*H*_*3*_), 2.18–2.25 (m, 2H, cyclobutoxy proton), 3.80 (s, 3H, −OC*H*_3_), 4.46 (p, 1H, *J* = 7.1 Hz, cyclobutoxy proton), 5.58 (s 2H, −OC*H*_2_Ph), 6.38 (s, 1H, *H-t*), 6.84 (t, 1H, *J* = 6.9 Hz, *H-b*), 6.87 (d, 2H, *J* = 8.0 Hz, aromatic protons), 6.91 (d, 2H, *J* = 8.4 Hz, *H-n*), 7.00 (t, 1H, *J* = 7.3 Hz, aromatic protons), 7.25–7.30 (m, 2H, aromatic protons), 7.35 (t, 1H, *J* = 7.9 Hz, *H-c*), 7.49 (d, 2H, *J* = 8.4 Hz, *H-m*), 8.28 (d, 1H, *J* = 6.8 Hz, *H-a*), 8.34 (d, 1H, *J* = 8.8 Hz, *H-d*), 8.44 (s, 1H, *H-q*), 9.16 (s, 1H, −N*H*); ^13^C NMR (151 MHz, chloroform-*d*): δ 13.1, 15.8, 30.5, 55.4, 71.6, 72.2 (−O*C*H_2_Ph), 91.6 (*C-f*), 105.6, 112.7 (*C-b*), 114.1, 116.3, 118.9 (*C-d*), 121.9, 122.3, 122.8, 125.3, 127.4, 128.4, 128.6, 129.7, 129.8, 143.1 (*C-e*), 145.4, 148.6, 158.6, 159.8, 161.3, 162.4. MS (ESI): 550 (M + 1).

##### *N*-(2-(Sec-butoxy)-5-methyl-4-phenoxyphenyl)-2-((4-methoxybenzyl)oxy)pyrazolo[1,5-*a*]pyridine-3-carboxamide (**34**)

Obtained using aniline **51**. The crude product was purified by flash chromatography (eluent: petroleum ether/EtOAc 85/15 v/v) to afford the title compound as a sticky solid. Yield 94%.^1^H NMR (600 MHz, chloroform-*d*): δ 0.85 (t, 3H, *J* = 7.5 Hz, −CHCH_2_C*H*_*3*_), 1.12 (d, 3H, *J* = 6.1 Hz, −CH(C*H*_*3*_)CH_2_CH_3_), 1.33–1.60 (m, 2H, −CH(CH_3_)C*H*_*2*_CH_3_), 2.16 (s, 3H, Ar-C*H*_*3*_), 3.79 (s, 3H, −OCH_3_), 4.12 (h, 1H, *J* = 6.1 Hz, −C*H*(CH_3_)CH_2_CH_3_), 5.56 (d, 1H, *J* = 12.1 Hz, −OC*H*_2_Ph), 5.59 (d, 1H, *J* = 12.1 Hz, −OC*H*_2_Ph), 6.54 (s, 1H, *H-t*), 6.83 (t, 1H, *J* = 6.8 Hz, *H-b*), 6.86–6.92 (m, 4H, aromatic protons), 7.0 (t, 1H, *J* = 7.3 Hz, aromatic proton), 7.25–7.31 (m, 2H, aromatic protons), 7.34 (t, 1H, *J* = 7.9 Hz, *H-c*), 7.47 (d, 2H, *J* = 8.5 Hz, *H-m*), 8.27 (d, 1H, *J* = 6.8 Hz, *H-a*), 8.34 (d, 1H, *J* = 8.9 Hz, *H-d*), 8.46 (s, 1H, *H-q*), 9.17 (s, 1H, −N*H*); ^13^C NMR (151 MHz, chloroform*-d*): δ 10.1 (−CH(CH_3_)CH_2_*C*H_3_) 15.8 (Ar-*C*H_3_), 19.3 (−CH(CH_3_)*C*H_2_CH_3_), 29.1 (−CH(*C*H_3_)CH_2_CH_3_), 55.4 (−O*C*H_3_), 71.5 (−*C*H(CH_3_)CH_2_CH_3_), 76.8 (−O*C*H_2_Ph), 91.6 (*C-f*), 106.7, 112.6 (*C-b*), 114.1, 116.2, 119.0 (*C-d*), 121.8, 122.4, 123.0, 126.3, 127.3, 128.4, 128.6, 129.7, 129.9, 143.2 (*C-e*), 146.0, 148.6, 158.8, 159.8, 161.4, 162.3. MS (ESI): 552 (M + 1).

##### 2-((4-Methoxybenzyl)oxy)-*N*-(5-methyl-2-(pentan-3-yloxy)-4-phenoxyphenyl)pyrazolo[1,5-*a*]pyridine-3-carboxamide (**35**)

Obtained using aniline **52**. The crude product was purified by flash chromatography (eluent: petroleum ether/EtOAc 85/15 v/v) to afford the title compound as a sticky solid. Yield 61%.^1^H NMR (600 MHz, chloroform-*d*): δ 0.84 (t, 6H, *J* = 7.4 Hz, −CH(CH_2_C*H*_*3*_)_2_, 1.43–1.54 (m, 4H, −CH(C*H*_*2*_CH_3_)_2_, 2.15 (s, 3H, Ar-C*H*_*3*_), 3.79 (s, 3H, −OCH_3_), 3.97 (p, 1H, *J* = 5.8 Hz, −C*H*(CH_2_CH_3_)_2_, 5.58 (s, 2H, −OC*H*_2_Ph), 6.54 (s, 1H, *H-t*), 6.83 (t, 1H, *J* = 6.8 Hz, *H-b*), 6.85–6.92 (m, 4H, aromatic protons), 7.0 (t, 1H, *J* = 7.3 Hz, aromatic proton), 7.25–7.31 (m, 2H, aromatic protons), 7.34 (t, 1H, *J* = 7.9 Hz, *H-c*), 7.46 (d, 2H, *J* = 8.5 Hz, *H-m*), 8.27 (d, 1H, *J* = 6.8 Hz, *H-a*), 8.34 (d, 1H, *J* = 8.9 Hz, *H-d*), 8.47 (s, 1H, *H-q*), 9.20 (s, 1H, −N*H*); ^13^C NMR (151 MHz, chloroform-*d*): δ 9.8 (−CH(CH_2_*C*H_3_)_2_) 15.8 (Ar-*C*H_3_), 26.3 (−CH*C*H_2_CH_3_)_2_), 55.4 (−OC*H*_*3*_), 71.5 (−O*C*H_2_Ph), 81.9 (−*C*HCH_2_CH_3_), 91.6 (*C-f*), 106.5, 112.6 (*C-b*), 114.1, 116.2, 119.0 (*C-d*), 121.8, 122.3, 122.9, 126.3, 127.3, 128.5, 128.6, 129.7, 129.8, 143.2 (*C*-*e*), 146.5, 148.5, 158.7, 159.8, 161.4, 162.3. MS (ESI): 566 (M + 1).

##### 2-((4-Methoxybenzyl)oxy)-*N*-(5-methyl-2-(pentan-2-yloxy)-4-phenoxyphenyl)pyrazolo[1,5-*a*]pyridine-3-carboxamide (**36**)

Obtained from **31** using aniline **53**. The crude product was purified by flash chromatography (eluent: petroleum ether/EtOAc 85:15 v/v) to afford the title compound as a sticky solid. Yield 89%.^1^H NMR (600 MHz, chloroform-*d*): δ 0.82 (t, 3H, *J* = 6.9 Hz, −CH_2_CH_2_C*H*_*3*_), 1.11 (d, 2H, *J* = 6.0 Hz, −CH(C*H*_*3*_)CH_2_CH_2_CH_3_)1.21–1.40 (m, 3H, −CH(CH_3_)C*H*_*2*_C*H*_*2*_CH_3_), 1.47–1.57 (m, 1H, −CH(CH_3_)C*H*_*2*_CH_2_CH_3_)_,_ 2.16 (s, 3H, Ar-C*H*_*3*_), 3.79 (s, 3H, −OC*H*_*3*_), 4.16–4.23 (m, 1H, −C*H*(CH_3_)CH_2_CH_2_CH_3_), 5.56 (d, 1H, *J* = 12.1 Hz, -OC*H*_2_Ph), 5.60 (d, 1H, *J* = 12.1 Hz, −OC*H*_2_Ph), 6.54 (s, 1H, *H-t*), 6.83 (t, 1H, *J* = 6.7 Hz, *H-b*), 6.85–6.92 (m, 4H, aromatic protons), 7.0 (t, 1H, *J* = 7.3 Hz, aromatic proton), 7.24–7.31 (m, 2H, aromatic protons), 7.34 (t, 1H, *J* = 7.9 Hz, *H-c*), 7.47 (d, 2H, *J* = 8.5 Hz, *H-m*), 8.27 (d, 1H, *J* = 6.8 Hz, *H-a*), 8.34 (d, 1H, *J* = 8.8 Hz, *H-d*), 8.47 (s, 1H, *H-q*), 9.17 (*s*, 1H, −N*H*); ^13^C NMR (151 MHz, chloroform-*d*): δ 14.1 (−CH(CH_3_)CH_2_CH_2_*C*H_3_) 15.8 (Ar-*C*H_3_), 18.9 (−CH(CH_3_)CH_2_*C*H_2_CH_3_), 19.8 (−CH(CH_3_)*C*H_2_CH_2_CH_3_), 38.4 (−CH(*C*H_3_)CH_2_CH_2_CH_3_), 55.4 (−O*C*H_3_), 71.5 (−O*C*H_2_Ph), 75.3 (−CH_3_*C*HCH_2_CH_2_CH_3_), 91.6 (*C-f*), 106.6, 112.6 (*C-b*), 114.1, 116.2, 118.9 (*C-d*), 121.8, 122.4, 122.9, 126.3, 127.3, 128.4, 128.6, 129.7, 129.8, 143.2 (*C*-*e*), 146.0, 148.6, 158.7, 159.8, 161.4, 162.3. MS (ESI): 566 (M + 1).

##### *N*-(2,5-Dimethyl-4-(pyridin-4-ylthio)phenyl)-2-((4-methoxybenzyl)oxy)pyrazolo[1,5-*a*]pyridine-3-carboxamide **(37)**

Oxalyl chloride (201 μL, 2.35 mmol, 3.6 equiv) and dry DMF (7 μL) were added to a cooled (0 °C) solution of **31** (0.783 mmol, 1.2 equiv) in dry THF (15 mL) under a nitrogen atmosphere. The reaction mixture was stirred for 2 h at room temperature under a nitrogen atmosphere. The solution was concentrated under reduced pressure, and the residue was dissolved in dry THF (10 mL); this step was repeated three times. A solution of aniline **54** (0.652 mmol, 1.0 equiv) and dry pyridine (2.347 mmol, 3.6 equiv) in dry toluene (5 mL) was added to the solution of acyl chloride under a nitrogen atmosphere. Due to the partial insolubility of **54** in dry toluene, 5 mL of dry THF was added. The resulting mixture was stirred at room temperature for 12 h and then at 70 °C overnight. The mixture was then quenched with 0.5 M HCl (25 mL). The layers were resolved, the aqueous phase was further extracted with EtOAc (3 × 50 mL), and the combined organic layer was dried and evaporated under reduced pressure. The crude material was purified using a flash chromatography eluent (from petroleum ether/EtOAc 70/30 v/v to DCM/methanol 80/20 v/v) to afford the title compound as a white solid (melting point: 205.0–205.8 °C from diisopropyl ether). Yield 80%. ^1^H NMR (600 MHz, chloroform-*d*): δ 1.77 (s, 3H, Ar-C*H*_3_), 2.34 (s, 3H, Ar-C*H*_3_), 3.84 (s, 3H, −OC*H*_*3*_), 5.48 (s, 2H, −OC*H*_*2*_Ph), 6.81 (*d*, 2H, *J* = 5.7 Hz, aromatic proton), 6.90 (*t*, 1H, *J* = 6.9 Hz, *H-b*), 6.94 (*d*, 2H, *J* = 8.4 Hz, *H-n*), 7.24 (*s*, 1H, aromatic protons), 7.40 (*t*, 1H, *J* = 7.9 Hz, *H-c*), 7.47 (*d*, 2H, *J* = 8.4 Hz, aromatic protons), 8.28 (*d*, 2H, *J* = 5.5 Hz, aromatic protons), 8.31–8.36 (m, 2H, aromatic protons), 8.51 (s, 1H, aromatic proton), 8.62 (s, 1H, −N*H*); ^13^C NMR (151 MHz chloroform-*d*): δ 16.8 (Ar-*C*H_3_), 20.7 (Ar-*C*H_3_), 55.5 (−O*C*H_2_Ph), 72.6 (−O*C*H_2_Ph), 91.1 (*C-f*), 113.1 (*C-b*), 114.3, 118.9 (*C-d*), 120.1, 121.0, 122.5, 125.3, 127.4, 127.9 (*C-a*), 128.8 (*C-c*), 131.4, 138.5, 139.4, 141.9, 143.1 (*C-e*), 149.4, 150.9, 160.5, 161.4, 162.4; MS (ESI): 511 (M + 1).

##### *N*-(2-Isopropyl-5-methyl-4-(pyridin-4-yloxy)phenyl)-2-((4-methoxybenzyl)oxy)pyrazolo[1,5-*a*]pyridine-3-carboxamide **(38)**

Oxalyl chloride (198 μL, 2.23 mmol, 3.60 equiv) and dry DMF (7 μL) were added to a cooled (0 °C) solution of **31** (0.743 mmol, 1.20 equiv) in dry THF (15 mL) under a nitrogen atmosphere. The reaction mixture was stirred for 2 h at room temperature under a nitrogen atmosphere. The solution was concentrated under reduced pressure, and the residue was dissolved in dry THF (10 mL, this step was repeated three times). The resulting acyl chloride was dissolved in dry toluene (10 mL). A solution of aniline **55** (0.619 mmol, 1.0 equiv) and dry pyridine (2.228 mmol, 3.60 equiv) in dry toluene (5 mL) was added to the solution of acyl chloride under a nitrogen atmosphere. The resulting mixture was stirred at room temperature overnight. The mixture was quenched in water (80 mL) and partially concentrated under reduce pressure. The aqueous phase was extracted with EtOAc (3 × 50 mL). The combined organic layers were dried and evaporated under reduced pressure. The crude material was purified using flash chromatography (eluent: petroleum ether/EtOAc 50:50 v/v) to afford the title compound as a white solid (melting point: 190.1–190.9 °C from diisopropyl ether). Yield 80%. ^1^H NMR (600 MHz, chloroform-*d*): δ 0.94 (d, 6H, *J* = 6.7 Hz, −CH(C*H*_*3*_)_2_), 2.11 (s, 3H, −C*H*_*3*_), 2.70 (hept, 1H, *J* = 6.7 Hz, −C*H*(CH_3_)_2_), 3.82 (s, 3H, −OC*H*_3_), 5.49 (s, 2H, −OC*H*_*2*_Ph), 6.74 (d, 2H, *J* = 4.3 Hz, aromatic protons), 6.83 (s, 1H, aromatic proton), 6.88 (t, 1H, *J* = 6.8 Hz, *H-b*), 6.95 (d, 2H, *J* = 8.4 Hz, *H-n*), 7.38 (t, 1H, *J* = 7.9 Hz, *H-c*), 7.47 (d 2H, *J* = 8.4 Hz, *H-m*), 8.13 (s, 1H, aromatic proton), 8.31–8.36 (m, 2H, aromatic protons), 8.41 (d, 2H, *J* = 4.7 Hz, aromatic protons), 8.55 (s,1H, −N*H*). ^13^C NMR (151 MHz, chloroform-*d*): δ 15.9 (Ar-*C*H_3_), 22.6 (−CH(*C*H_3_)_2_), 27.8 (−*C*H(CH_3_)_2_), 55.5 (−O*C*H_3_), 72.4 (−O*C*H_2_Ph), 91.0 (*C-f*), 111.3, 112.9 (*C-b*), 114.3, 118.3, 119.0 (*C-d*), 126.1, 127.6, 127.7 (*C-a*), 128.3, 128.7 (*C-c*), 131.1, 132.9, 138.3, 143.2 (*C-e*), 148.1, 151.4, 160.4, 161.6, 162.5, 165.2. MS (ESI): 523 (M + 1).

##### General Hydrogenation Procedure for Target Compounds **3–16** and **19**

Palladium on carbon (10% w/w Pd/C, 45 mg) was added to a solution of the appropriate amide (compounds **22**–**31** and **34**–**38**, 0.300 mmol) in dry THF (15 mL). The resulting mixture was vigorously stirred under a hydrogen atmosphere for 3 h. The suspension was filtered through celite, and the cake was washed with methanol. The filtrate was concentrated under reduced pressure, providing a solid that, when necessary, was further purified by flash chromatography.

##### 2-Hydroxy-*N*-(2-methyl-4-(p-tolyloxy)phenyl)pyrazolo[1,5-*a*]pyridine-3-carboxamide (**3**)

Obtained from **21**, flash chromatography eluent: DCM/methanol 90/10 v/v. White solid (m.p. 238.3–239.9 °C from trituration with diisopropyl ether). Yield 87%. ^1^H NMR (600 MHz DMSO-*d*_6_): δ 2.28 (*s*, 3H, Ar-C*H*_*3*_), 6.88 (dd, 1H, *J* = 8.8, 2.6 Hz, *H-r*), 6.94–7.01 (m, 4H, aromatic protons), 7.09 (t, 1H, J = 7.3 Hz, *H-b*), 7.37 (t, 2H, J = 7.9 Hz, aromatic protons), 7.47 (t, 1H, *J* = 7.9, *H-c*), 8.06 (d, 1H, *J* = 8.8 Hz, *H-q*), 8.20 (d, 1H, *J* = 8.8 Hz, *H-d*), 8.57 (d, 1H, *J* = 6.8 Hz, *H-a*), 8.93 (s, 1H, −N*H*), 12.99 (v br s, 1H, −O*H*). ^13^C NMR (151 MHz, DMSO-*d*_6_): δ 17.6 (Ar-*C*H_3_), 89.5 (*C-f*), 112.9 (*C-b*), 117.0, 117.1 (*C-d*), 117.9, 121.0, 122.2, 122.9, 127.8 (*C-c*), 129.0 (*C-a*), 129.2, 130.0, 133.1, 141.5 (*C-e*), 151.6 (*C-s*), 157.5 (*C-v*), 160.8 (*C-g*)*, 162.1 (*C-h*)*; IR (KBr) ν (cm^–1^): 3388, 3039, 2567, 1664, 1633, 1590, 1549, 1485, 1445, 1413, 1380, 1333, 1307, 1273, 1245, 1227, 1173, 1134; MS (ESI): 360 (M + 1). ESI-HRMS (*m*/*z*): [M + H]^+^calcd for C_21_H_18_N_3_O_3_, 360.1343; obsd. 360.1341.

##### 2-Hydroxy-*N*-(2-ispropyl-5-methyl-4-phenoxyphenyl)pyrazolo[1,5-*a*]pyridine-3-carboxamide (**4**)

Obtained from **22**, flash chromatography eluent: DCM/methanol 98/2 v/v. White solid (mp 244.2–247.9 °C dec.; from trituration with diisopropyl ether). Yield 91%. ^1^H NMR (600 MHz, DMSO-*d*_6_): δ 1.17 (d, 6H, *J* = 6.8 Hz, −CH(C*H*_3_)_2_), 2.10 (s, 3H, Ar-C*H*_3_), 3.10 (hept, 1H, *J* = 6.8 Hz, −C*H*(CH_3_)_2_), 6.85 (d, 2H, *J* = 8.1 Hz, aromatic protons), 6.89 (s, 1H, *H-t*), 6.98 (t, 1H, *J* = 6.9 Hz, *H-b*), 7.03 (t, 1H, *J* = 7.3 Hz, aromatic proton), 7.33 (t, 2H, *J* = 7.9 Hz, aromatic protons), 7.47 (t, 1H, *J* = 7.9 Hz, *H-c*), 8.00 (s, 1H, *H-q*), 8.06 (d, 1H, *J* = 8.8 Hz, *H-d*), 8.57 (d, 1H, *J* = 6.8 Hz, *H-a*), 8.98 (s, 1H, −N*H*), 12.95 (v br s, 1H, −O*H*). ^13^C NMR (151 MHz, DMSO-*d*_6_): δ 15.7 (Ar-*C*H_3_), 22.6 (−CH(*C*H_3_)_2_), 27.4 (−*C*H(CH_3_)_2_), 89.4 (*C-f*), 112.9 (*C-b*), 116.0, 117.1 (*C-d*), 117.5, 122.0, 125.7, 127.0, 127.8 (*C–c*), 129.0 (*C-a*), 129.9, 131.9, 138.0, 141.5 (*C-e*), 149.5, 158.0 (*C-v*), 161.0 (*C-h*)*, 162.1 (*C-g*)*. IR (KBr) *v* (cm ^–1^): 3400, 2964, 2579, 1661, 1637, 1547,1492, 1446, 1404, 1332, 1228, 1185, 1130, 887; MS (ESI): 402 (M + 1). ESI-HRMS (*m*/*z*): [M + H]^+^calcd for C_24_H_24_N_3_O_3_, 402.1812; obsd. 402.1811.

##### 2-Hydroxy-*N*-(5-isopropyl-2-methyl-4-phenoxyphenyl)pyrazolo[1,5-*a*]pyridine-3-carboxamide (**5**)

Obtained from **23**, flash chromatography eluent: DCM/methanol 95/5 v/v. White solid (m.p. 273.9–276.5 °C dec.; from trituration with diisopropyl ether). Yield 70%. ^1^H NMR (600 MHz, DMSO-*d*_6_) δ 1.16 (d, 6H, *J* = 6.8 Hz, −CH(C*H*_3_)_2_), 2.23 (s, 3H, Ar-C*H*_*3*_), 3.09 (hept, 1H, J = 6.8 Hz, −C*H*(CH_3_)_2_), 6.82 (s, 1H, *H-t*), 6.87 (d, 2H, *J* = 8.0 Hz, aromatic protons), 7.98 (t, 1H, *J* = 6.6 Hz, *H-b*), 7.03 (t, 1H, *J* = 7.2 Hz, aromatic proton), 7.33 (t, 2H, *J* = 7.7 Hz, aromatic protons), 7.47 (t, 1H, *J* = 7.8 Hz, *H-c*), 8.10 (d, 1H, *J* = 8.7 Hz, *H-d*), 8.31 (s, 1H, *H-q*), 8.58 (d, 1H, *J* = 6.6 Hz, *H-a*), 8.98 (s, 1H, -N*H*), 13.01 (v br s, 1H, −O*H*). ^13^C NMR (151 MHz, DMSO-*d*_6_): δ 17.0 (-*C*H(CH_3_)_2_), 23.0 (−CH(*C*H_3_)_2_), 26.6 (Ar-*C*H_3_), 89.5 (*C-f*), 112.9 8 (*C-b*), 116.4, 117.1 (*C-d*), 119.1, 122.1, 122.2, 126.2, 127.7 (*C-c*), 129.0 (*C-a*), 129.9, 134.2, 137.6, 141.5 (*C-e*), 147.5, 158.5, 160.8 (*C-g*)*, 162.1 (*C-h*)*. IR (KBr) *v* (cm ^–1^): 3393, 2961, 2578, 1659, 1636, 1548, 1486, 1446, 1407, 1333, 1217, 1160, 1126, 1042, 978; MS (ESI): 402 (M+1). ESI-HRMS (*m*/*z*): [M + H]^+^calcd for C_24_H_24_N_3_O_3_, 402.1812; obsd. 402.1808.

##### 2-Hydroxy-*N*-(2-isopropoxy-5-methyl-4-phenoxyphenyl)pyrazolo[1,5-*a*]pyridine-3-carboxamide (**6**)

Obtained from **32**, flash chromatography eluent: DCM/methanol 95/5 v/v. White solid (melting point: 239.3–240.6 °C dec.; from diisopropyl ether). Yield 89%. ^1^H NMR (600 MHz, chloroform-*d*): δ 1.40 (d, 6H, *J* = 6.0 Hz, −CH(C*H*_3_)_2_), 2.19 (s, 3H, Ar-C*H*_*3*_), 4.51 (h, 1H, *J* = 6.0 Hz, −C*H*(CH_3_)_2_), 6.63 (s, 1H, *H-t*), 6.88–6.92 (m, 3H, *H-b* and aromatic protons), 7.0 (t, 1H, *J* = 7.3 Hz, aromatic proton), 7.30 (t, 2H, *J* = 7.9 Hz, aromatic protons), 7.43 (t, 1H, *J* = 7.9 Hz, *H-c*), 8.27 (d, 1H, *J* = 6.8 Hz, *H-a*), 8.35 (d, 1H, *J* = 8.8 Hz, *H-d*), 8.52 (*s*, 1H, *H-q*), 9.32 (*s*, 1H, -N*H*); ^13^C NMR (151 MHz, chloroform-*d*): δ 15.9 (Ar-*C*H_3_), 22.4 (−CH(*C*H_3_)_2_), 72.0 (−*C*H(CH_3_)_2_), 91.1 (*C-f*), 107.3, 113.2 (*C-b*), 116.3, 118.8 (*C-d*), 122.0, 122.5, 123.1, 126.6, 127.4, 128.2, 129.8, 136.4, 141.9 (*C-e*), 145.4, 148.7, 158.7, 161.4, 162.8. MS (ESI): 418 (M – 1). ESI-HRMS (*m*/*z*): [M + H]^+^calcd for C_24_H_24_N_3_O_4_, 418.1761; obsd. 418.1759.

##### *N*-(2-Cyclobutoxy-5-methyl-4-phenoxyphenyl)-2-hydroxypyrazolo[1,5-*a*]pyridine-3-carboxamide (**7**)

Obtained from **33**, Flash chromatography eluent: DCM/methanol 95/5 v/v. White solid (melting point: 262.5–263.3 °C dec.; from diisopropyl ether). Yield 75%. ^1^H NMR (600 MHz, DMSO-*d*_6_): δ 1.55–1.65 (m, 1H, cyclobutoxy proton), 1.73–1.82 (m, 1H, cyclobutoxy proton), 2.00–2.14 (m, 2H, cyclobutoxy proton), 2.06 (s, 3H, Ar-C*H*_*3*_), 2.30–2.39 (m, 2H, cyclobutoxy proton), 4.70 (p, 1H, *J* = 7.0 Hz, cyclobutoxy proton), 6.53 (s, 1H, *H-t*), 6.85 (d, 2H, *J* = 8.1 Hz, aromatic proton), 6.98 (t, 1H, *J* = 6.8 Hz, *H-b*), 7.03 (t, 1H, *J* = 7.3 Hz, aromatic proton), 7.32 (t, 2H, *J* = 7.8 Hz, aromatic protons), 7.47 (t, 1H, *J* = 7.8 Hz, *H-c*), 8.09 (d, 1H, *J* = 8.7 Hz, *H-d*), 8.46 (s, 1H, *H-q*), 8.58 (d, 1H, *J* = 6.7 Hz, *H-a*), 9.62 (s, 1H, −N*H*), 12.85 (br s, 1H, −O*H*); ^13^C NMR (151 MHz, DMSO-*d*_6_): δ 12.7, 15.5, 29.8, 71.8, 89.7 (*C-f*), 105.9, 112.8 (*C-b*), 115.9, 117.0 (*C-d*), 120.8, 121.0, 122.0, 125.8, 127.7, 129.0, 129.9, 141.5 (*C-e*), 144.3, 147.4, 158.0, 160.6, 162.0. MS (ESI): 430 (M + 1). ESI-HRMS (*m*/*z*): [M + H]^+^calcd for C_25_H_24_N_3_O_4_, 430.1767; obsd. 430.1766.

##### *N*-(2-(Sec-butoxy)-5-methyl-4-phenoxyphenyl)-2-hydroxypyrazolo[1,5-*a*]pyridine-3-carboxamide (**8**)

Obtained from **34**, flash chromatography eluent: DCM/methanol 95/5 v/v. White solid. Yield 76%. ^1^H NMR (600 MHz, DMSO-*d*_6_): δ 0.91 (t, 3H, *J* = 7.4 Hz, −CHCH_2_C*H*_*3*_), 1.22 (d, 3H, *J* = 6.0 Hz, −C*H*_*3*_CH(C*H*_*3*_)CH_2_CH_3_), 1.56–1.75 (m, 2H, −CH(CH_3_)C*H*_*2*_CH_3_), 2.06 (s, 3H, Ar-C*H*_*3*_), 4.36 (h, 1H, *J* = 5.8 Hz, −C*H*(CH_3_)CH_2_CH_3_), 6.73 (s, 1H, *H-t*), 6.85 (d, 2H, *J* = 8.1 Hz, aromatic protons), 6.98 (t, 1H, *J* = 6.8 Hz, *H-b*), 7.02 (t, 1H, *J* = 7.3 Hz, aromatic proton), 7.32 (t, 2H, *J* = 7.9 Hz, aromatic protons), 7.34 (m, 1H, *H-c*), 8.09 (d, 1H, *J* = 8.8 Hz, *H-d*), 8.47 (s, 1H, *H-q*), 8.57 (d, 1H, *J* = 6.8 Hz, *H-a*), 9.63 (s, 1H, −N*H*), 12.79 (br s, 1H, −O*H*); ^13^C NMR (151 MHz, DMSO-*d*_6_): δ 9.4 (−CH(CH_3_)CH_2_*C*H_3_) 15.5 (Ar-*C*H_3_), 18.9 (−CH(CH_3_)*C*H_2_CH_3_), 28.4 (−CH(*C*H_3_)CH_2_CH_3_), 76.1 (−*C*H(CH_3_)CH_2_CH_3_), 89.7 (*C-f*), 107.0, 112.8 (*C-b*), 115.8, 117.1 (*C-d*), 120.9, 121.1, 121.9, 126.8, 127.7, 129.0, 129.9, 141.5 (*C-e*), 144.9, 147.4, 158.1, 160.6, 162.0. MS (ESI): 432 (M + 1). ESI-HRMS (*m*/*z*): [M + H]^+^calcd for C_25_H_26_N_3_O_4_, 432.1922; obsd. 432.1917.

##### 2-Hydroxy-*N*-(5-methyl-2-(pentan-3-yloxy)-4-phenoxyphenyl)pyrazolo[1,5-*a*]pyridine-3-carboxamide (**9**)

Obtained from **35**, flash chromatography eluent: DCM/methanol 95/5 v/v. White solid. Yield 30%. ^1^H NMR (600 MHz, chloroform-*d*): δ 0.96 (t, 6H, *J* = 7.4 Hz, −CHCH_2_C*H*_*3*_), 1.70–1.77 (m, 4H, −CHC*H*_*2*_CH_3_), 2.18 (s, 3H, Ar-C*H*_*3*_), 4.12 (p, 1H, J = 5.6 Hz, −C*H*CH_2_CH_3_), 6.59 (s, 1H, *H-t*), 6.89 (d, 2H, *J* = 8.1 Hz, aromatic protons), 6.93 (t, 1H, *J* = 6.7 Hz, *H-b*), 7.02 (t, 1H, *J* = 7.3 Hz, aromatic proton), 7.30 (t, 2H, *J* = 7.8 Hz, aromatic protons), 7.47 (t, 1H, *J* = 7.9 Hz, *H-c*), 8.27–8.41 (m, 2H, *H-a* and *H-d*), 8.49 (s, 1H, *H-q*), 9.25 (s, 1H, −N*H*); ^13^C NMR (151 MHz, *chloroform-d*): δ 9.7 (−CHCH_2_*C*H_3_) 15.9 (Ar-*C*H_3_), 26.2 (−CH*C*H_2_CH_3_), 81.7 (−*C*HCH_2_CH_3_), 91.0 (*C-f*), 106.5, 113.4 (*C-b*), 116.3, 118.6 (*C-d*), 122.0, 122.5, 122.6, 126.1, 127.6, 128.6, 129.8, 141.6 (*C-e*), 146.0, 148.9, 158.7, 161.4, 162.3. MS (ESI): 446 (M + 1). ESI-HRMS (*m*/*z*): [M + H]^+^calcd for C_26_H_28_N_3_O_4_, 446.2079; obsd. 446.2074.

##### 2-Hydroxy-*N*-(5-methyl-2-(pentan-2-yloxy)-4-phenoxyphenyl)pyrazolo[1,5-*a*]pyridine-3-carboxamide (**10**)

Obtained from **36**, flash chromatography eluent: DCM/methanol 95/5 v/v. White solid (melting point: 250.5–251.3 °C dec.; from diisopropyl ether). Yield 89%. ^1^H NMR (600 MHz, DMSO-*d*_6_): δ 0.84 (t, 3H, *J* = 7.3 Hz, −CH_2_CH_2_C*H*_*3*_), 1.22 (d, 2H, *J* = 6.0 Hz, −CH(C*H*_*3*_)CH_2_CH_2_CH_3_), 1.27–1.44 (m*,* 2H, −CH(CH_3_)CH_2_C*H*_*2*_CH_3_), 1.47–1.57 (m, 1H, −CH(CH_3_)C*H*_*2*_CH_2_CH_3_)_,_ 1.63–1.74 (m, 1H, −CH(CH_3_)C*H*_*2*_CH_2_CH_3_)_,_ 2.06 (s, 3H, Ar-C*H*_*3*_), 4.37–4.43 (m, 1H, −C*H*(CH_3_)CH_2_CH_2_CH_3_), 6.72 (s, 1H, *H-t*), 6.85 (d, 2H, *J* = 8.1 Hz, *aromatic protons*), 6.96 (t, 1H, *J* = 6.7 Hz, *H-b*), 7.02 (t, 1H, *J* = 7.3 Hz, *aromatic proton*), 7.32 (t, 1H, *J* = 7.8 Hz, *aromatic proton*), 7.45 (t, 1H, *J* = 7.8 Hz, *H-c*), 8.08 (d, 1H, *J* = 8.7 Hz, *H-d*), 8.47 (s, 1H, *H-q*), 8.55 (d*,* 1H, *J* = 6.7 Hz, *H-a*), 9.71 (s, 1H, −N*H*), 12.84 (s, 1H, −O*H*); ^13^C NMR (151 MHz, DMSO-*d*_6_): δ 13.9 (−CH(CH_3_)CH_2_CH_2_*C*H_3_) 15.5 (Ar-*C*H_3_), 18.1 (−CH(CH_3_)CH_2_*C*H_2_CH_3_), 19.4 (−CH(CH_3_)*C*H_2_CH_2_CH_3_), 37.8 (−CH(*C*H_3_)CH_2_CH_2_CH_3_), 74.9 (−*C*H(CH_3_)CH_2_CH_2_CH_3_), 89.7 (*C-f*), 106.9, 112.6 (*C-b*), 115.8, 116.9 (*C-d*), 120.9, 121.0, 121.9, 126.8, 127.5, 128.9, 129.9, 141.5 (C-e), 144.9, 147.4, 158.1, 160.7. MS (ESI): 446 (M + 1). ESI-HRMS (*m*/*z*): [M + H]^+^calcd for C_26_H_28_N_3_O_4_, 446.2079; obsd. 446.2078.

##### 2-Hydroxy-*N*-(1*H*-indol-5-yl)pyrazolo[1,5-*a*]pyridine-3-carboxamide (**11**)

Obtained from **27**. Flash chromatography eluent: dichloromethane/methanol 95/5 v/v). Gray solid (mp 270.7–271.4 °C dec.; from trituration with diisopropyl ether). Yield 74%. ^1^H NMR (600 MHz, DMSO-*d*_6_): δ 6.39 (s, 1H, *aromatic proton*), 6.95 (t, 1H, *J* = 6.7 Hz, *H-b*), 7.24 (dd, 1H, *J* = 8.6, 1.1 Hz, *aromatic proton*), 7.27–7.38 (m, 2H, *aromatic protons*), 7.44 (t, 1H, *J* = 7.8 Hz, *H-c*), 7.97 (d, 1H, *J* = 1.9 Hz, *aromatic proton*), 8.07 (d, 1H, *J* = 8.8 Hz, *H-d*), 8.55 (d, 1H, *J* = 6.8 Hz, *H-a*), 9.03 (s, 1H, −N*H*), 11.02 (s, 1H, −N*H indole*), 18.84 (v br s, 1H, −O*H*); ^13^C NMR (151 MHz, DMSO-*d*_6_): δ 89.6 (*C-f*), 101.1, 110.6, 111.4, 112.5 (*C-b*), 115.0, 117.1 (*C-d*), 126.0, 127.3 (*C–c*), 127.7, 128.3, 130.8 (*C-a*), 132.7, 141.4 (*C-e*), 160.8 (*C-g*)*, 162.1 (*C-h*)*. MS (ESI): 293 (M + 1). ESI-HRMS (*m*/*z*): [M + H]^+^ calcd for C_16_H_14_N_4_O_2_, 293.1033; obsd. 293.1031.

##### 2-Hydroxy-*N*-(1-phenyl-1*H*-indol-5-yl)pyrazolo[1,5-*a*]pyridine-3-carboxamide (**12**)

Obtained from **28**. Flash chromatography eluent: DCM/methanol 95/5 v/v. Pale-brown solid (mp 252.4–253.8 °C dec., from trituration with diisopropyl ether). Yield 90%. ^1^H NMR (600 MHz, DMSO-*d*_6_): δ 6.68 (d, 1H, *J* = 3.0 Hz, *aromatic proton*), 6.96 (t, 1H, *J* = 6.7 Hz, *H-b*), 7.32–7.42 (m, 2H, *aromatic protons*), 7.45 (t, 1H, *J* = 7.8 Hz, *H-c*), 7.49–7.62 (m, 5H, *aromatic protons*), 7.65 (d, 1H, *J* = 3.1 Hz, *aromatic proton*), 8.08 (d, 1H, *J* = 8.8 Hz, *H-d*), 8.13 (s, 1H, *aromatic proton*), 8.56 (d, 1H, *J* = 6.7 Hz, *H-a*), 9.15 (*s*, 1H, -N*H*), 12.95 (*s*, 1H, -O*H*); ^13^C NMR (151 MHz, DMSO-*d*_6_): *δ* 89.6 (*C-f*), 103.6, 110.5, 111.2, 112.6 (*C-b*), 115.9, 117.0 (*C-d*), 123.5, 126.3, 127.4 (*C–c*), 128.9, 129.0, 129.3 (*C-a*), 129.9, 131.7, 132.2, 139.2, 141.4 (*C-e*), 160.9, 162.2. MS (ESI): 369 (M + 1). ESI-HRMS (*m*/*z*): [M + H]^+^calcd for C_22_H_17_N_4_O_2_, 369.1346; obsd. 369.1344.

##### 2-Hydroxy-*N*-(1-phenyl-1*H*-benzo[d]imidazole-5-yl)pyrazolo[1,5-*a*]pyridine-3-*C*arboxamide (**13**)

Obtained from **29**. Flash chromatography eluent: DCM/MeOH 80/20 v/v. Pale-brown solid (mp 258.7–260.1 °C dec., from trituration with diisopropyl ether). Yield 90%. ^1^H NMR (600 MHz, DMSO6-*d*_6_): δ 6.87 (t, 1H, *J* = 6.5 Hz, *H-b*), 7.37 (t, 1H, *J* = 7.7 Hz, *H-c*), 7.46–7.54 (m, 2H, *aromatic protons*), 7.57 (d, 1H, *J* = 8.6 Hz, *aromatic proton*), 7.63 (t, 2H, *J* = 7.8 Hz, *aromatic protons*), 7.69 (d, 2H, *J* = 7.7 Hz, *aromatic protons*), 8.00 (d, 1H, *J* = 8.6 Hz, *H-d*), 8.32 (s, 1H, *aromatic proton*), 8.48 (d, 1H, *J* = 6.4 Hz, *H-a*), 8.54 (s, 1H, *aromatic proton*), 9.99 (s, 1H, −N*H*); ^13^C NMR (151 MHz, DMSO-*d*_6_): δ 89.6 (*C-f*), 109.8, 110.6, 111.8, 116.4 (*C-b*), 116.5, 123.4, 126.7, 127.6 (*C–c*), 128.4, 128.9, 130.1 134.8, 136.1, 141.4 (*C-e*), 141.5, 143.6, 144.2, 161.9. MS (ESI): 368 (M – 1). ESI-HRMS (*m*/*z*): [M + H]^+^calcd for C_21_H_16_N_5_O_2_, 370,1299; obsd. 370.1296.

##### 2-Hydroxy-*N*-(1-phenyl-1*H*-benzo[d][1,2,3]triazol-5-yl)pyrazolo[1,5-*a*]pyridine-3-carboxamide (**14**)

Obtained from **30**. Flash chromatography eluent: DCM/MeOH 95/5 v/v. Pale-yellow solid (m.p. 269.9–271.9 °C dec., from trituration with diisopropyl ether). Yield 72%. ^1^H NMR (600 MHz, DMSO-*d*_6_): δ 6.85 (t, 1H, *J* = 6.6 Hz, *H-b*), 7.35 (t, 1H, *J* = 7.7 Hz, *H-c*), 7.58 (t, 1H, *J* = 7.5 Hz, *aromatic proton*), 7.70 (t, 2H, *J* = 7.9 Hz, *aromatic protons*), 7.78 (d, 1H, *J* = 8.1 Hz, *aromatic proton*), 7.84–7.91 (m, 3H, *aromatic protons*), 7.95 (d, 1H, *J* = 8.6 Hz, *H-d*), 8.44 (d, 1H, *J* = 6.6 Hz, *H-a*), 8.71 (s, 1H, *aromatic proton*), 10.53 (s, 1H, −N*H*); ^13^C NMR (151 MHz, DMSO-*d*_6_): δ 89.5 (*C-f*), 106.8, 111.1 (*C-b*), 111.7, 116.1 (*C-d*), 122.5, 122.7, 126.6, 128.0, 128.3, 128.7, 130.1, 136.5, 136.8, 137.6, 141.5 (*C-e*), 146.5, 162.5. MS (ESI): 369 (M – 1). ESI-HRMS (*m*/*z*): [M + H]^+^calcd for C_20_H_15_N_6_O_2_, 371.1251; obsd. 371,1248.

##### 2-Hydroxy-*N*-(5-phenoxypyridin-2-yl)pyrazolo[1,5-*a*]pyridine-3-carboxamide (**15**)

Obtained from **24**, flash chromatography eluent: DCM/methanol 90/10 v/v. White solid (mp 161.1–161.9 °C dec., from trituration with diisopropyl ether). Yield 76%. ^1^H NMR (600 MHz, DMSO-*d*_6_): δ 6.75 (t, 1H, *J* = 6.7 Hz, *H-b*), 6.97 (d, 1H, *J* = 8.7 Hz, *aromatic proton*), 7.08 (d, 2H, *J* = 8.0 Hz, *aromatic protons*), 7.16 (t, 1H, *J* = 7.4 Hz, *aromatic proton*), 7.26 (t, 1H, *J* = 7.8 Hz, *aromatic proton*) 7.39 (t, 2H, *J* = 7.8 Hz, *aromatic protons*), 7.83 (d, 1H, *J* = 8.7 Hz, *H-d*), 8.21 (dd, 1H, *J* = 8.8, 2.5 Hz, *aromatic proton*), 8.33 (d, 1H, *J* = 6.6 Hz, *H-a*), 8.48 (d, 1H, *J* = 2.2 Hz, *aromatic proton*), 10.78 (s, 1H, −N*H*); ^13^C NMR (151 MHz, DMSO-*d*_6_): δ 89.3 (*C-f*), 111.1 (*C-b*), 111.7, 115.6 (*C-d*), 120.2, 123.9, 125.9 (*C–c*), 127.9, 129.7, 131.1 (*C-a*), 132.9, 137.8, 141.4 (*C-e*), 154.9, 157.6 (*C-g*), 162.8 (*C-h*). MS (ESI): 345 (M – 1). IR (KBr) v (cm^–1^): 3061, 1653, 1636, 1534, 1476, 1379, 1248, 1205, 1124, 1023. ESI-HRMS (*m*/*z*): [M + H]^+^calcd for C_19_H_15_N_4_O_3_, 347.1139; obsd. 347.1138.

##### 2-Hydroxy-*N*-5-[3-(trifluoromethyl)phenoxy]pyridin-2-ylpyrazolo[1,5-*a*]pyridine-3-carboxamide (**16**)

Obtained from **25,** flash chromatography eluent: DCM/methanol 90/10 v/v. White solid (mp 241.2–242.0 °C dec.; from trituration with diisopropyl ether). Yield 45%. ^1^H NMR (600 MHz, DMSO-*d*_6_): δ 6.59 (t, 1H, *J* = 6.7 Hz, *H-b*), 7.10 (t, 1H, *J* = 7.7 Hz, *H-c*), 7.26–7.34 (m, 2H, *aromatic protons*), 7.46 (d, 1H, *J* = 7.6 Hz, *aromatic proton*), 7.55 (dd, 1H, *J* = 9.0, 2.8 Hz, *aromatic proton*), 7.61 (t, 1H, *J* = 8.0 Hz, *aromatic proton*), 7.65 (d, 1H, *J* = 8.4 Hz, *aromatic proton*), 8.12 (d, 1H, *J* = 2.8 Hz, *aromatic proton*), 8.14 (d, 1H, *J* = 6.5 Hz, *H-a*), 8.40 (d, 1H, *J* = 9.0 Hz, *H-d*), 12.48 (s, 1H, −N*H*); ^13^C NMR (151 MHz, DMSO-*d*_6_): δ 89.0 (*C-f*), 109.8, 113.6 (q, *J* = 4.0 Hz), 114.3, 119.4 (q, *J* = 3.4 Hz), 120.9, 123.8 (q, *J* = 272.0 Hz, −*C*F_3_), 124.4, 127.0, 129.9, 130.7 (q, *J* = 32.1 Hz), 131.5, 140.1, 140.8, 141.7, 146.0, 150.7, 158.4, 163.9, 173.1. MS (ESI): 413 (M – 1). IR (KBr) v (cm^–1^): 3328, 2925, 1653, 1636, 1559, 1448, 1328, 1284, 1241, 1173, 1129, 1065. ESI-HRMS (*m*/*z*): [M + H]^+^calcd for C_20_H_14_F_3_N_4_O_3_, 415.1013; obsd. 415.1009.

##### 2-Hydroxy-*N*-(2-isopropyl-5-methyl-4-(4-(trifluoromethyl)phenoxy)phenyl)pyrazolo[1,5-*a*]pyridine-3-carboxamide (**19**)

Obtained from **26**, flash chromatography eluent: DCM/methanol 95/5 v/v. White solid (mp 249.2–249.9 °C from trituration with diisopropyl ether). Yield 98%. ^1^H NMR (600 MHz, DMSO-*d*_6_): δ 1.20 (d, 6H, *J* = 6.7 Hz, −CH(C*H*_*3*_)_2_), 2.08 (s, 3H, Ar-C*H*_*3*_), 3.08–3.16 (m, 1H, −C*H*(CH_3_)_2_), 6.95–7.03 (m, 4H, *aromatic protons*, *H-t* and *H-b*), 7.48 (t, 1H *J* = 7.8 Hz, *H-c*), 7.70 (d, 2H *J* = 8.6 Hz, *aromatic protons*), 8.07 (d, 1H, *J* = 8.8 Hz, *H-d*), 8.10 (s, 1H, *H-q*), 8.58 (d, 1H, *J* = 6.8 Hz, *H-a*), 9.05 (s, 1H, −N*H*), 13.01 (v br s, 1H, −O*H*); ^13^C NMR (151 MHz, DMSO-*d*_6_): δ 15.6 (Ar-*C*H_3_), 22.6 (−CH(*C*H_3_)_2_), 27.4 (−CH(*C*H_3_)_2_), 89.4 (*C-f),* 112.9 (*C-b*), 115.9, 117.0 (*C-d*), 118.2, 122.4 (q, *J* = 33.1 Hz), 124.4 (q, *J* = 271.1 Hz, −*CF*_*3*_), 125.5, 127.1, 127.5 (q, *J* = 3.7 Hz), 127.8 (*C–c*), 129.0 (*C-a*), 132.8, 138.1, 141.6 (*C-e*), 148.1 (*C-s*), 161.0 (*C-v*), 161.1 (*C-g*), 162.1 (*C-h*); IR (KBr) *ν* (cm^–1^): 3402, 2948, 2576, 1665, 1640, 1615, 1550, 1515, 1482, 1446, 1404, 1334, 1250, 1214, 1183, 1156, 114, 1103; MS (ESI): 470 (M+1). ESI-HRMS (*m*/*z*): [M + H]^+^ calcd for C_25_H_23_F_3_N_3_O_3_ 470.1686; obsd. 470.1685.

##### General procedure for the Synthesis of Compounds **17** and **18**

Thioanisole (10.0 equiv) was added to a solution of the respective protected amide **37** and **38** (1.0 equiv) in TFA (4 mL). The mixture was heated at 70 °C for 4 h and then cooled to r.t.. The mixture was partially concentrated, and the crude was taken up with phosphate saline buffer pH = 5, giving a suspension that was filtered. The solid so obtained was triturated with water and then with hexane to afford the title compounds in the pure form.

##### 2-Hydroxy-*N*-(2,5-dimethyl-4-(pyridin-4-ylthio)phenyl)pyrazolo[1,5-*a*]pyridine-3-carboxamide (**17**)

Obtained from **37**, flash chromatography eluent: from DCM to DCM/methanol 95/5 v/v. White solid (melting point: 250.5–251.9 °C dec.; from diisopropyl ether). Yield 80%. ^1^H NMR (600 MHz, DMSO-*d*_6_): δ 2.31 (s, 3H, Ar-C*H*_3_), 2.32 (s, 3H, Ar-C*H*_3_), 7.02 (*td*, 1H, *J* = 6.9, 1.2 Hz, *H-b*), 7.28 (d, 2H, *J* = 6.4 Hz, *aromatic protons*), 7.48–7.56 (m, 2H, *aromatic protons and H-c*), 8.08 (d, 1H, *J* = 8.8 Hz, *H-d*); 8.50 (d, 2H, *J* = 5.1 Hz, *aromatic protons*), 8.58–8.65 (m, 2H, *aromatic proton and H-a*), 9.20 (s, 1H, −N*H*); ^13^C NMR (151 MHz DMSO-*d*_6_): δ 16.6 (Ar-*C*H_3_), 20.2 (Ar-*C*H_3_), 89.4 (*C-f*), 113.2 (*C-b*), 117.0, 118.1 (*C-d*), 120.8, 121.7, 125.5, 128.2 (*C-a*), 129.1 (*C–c*), 138.1, 140.2, 140.9, 141.6, 143.8 (*C-e*), 160.9, 162.2. MS (ESI): 389 (M – 1). ESI-HRMS (*m*/*z*): [M + H]^+^calcd for C_21_H_19_N_4_O_2_S, 391.1223; obsd. 391.1222.

##### 2-Hydroxy-*N*-(2-isopropyl-5-methyl-4-(pyridin-4-yloxy)phenyl)pyrazolo[1,5-*a*]pyridine-3-carboxamide (**18**)

Obtained from **38**, flash chromatography eluent: from DCM to DCM/methanol 95/5 v/v. White solid (melting point: 226.3–226.8 °C dec.; from diisopropyl ether). Yield 74%. ^1^H NMR (600 MHz, DMSO-*d*_6_): δ 1.22 (d, 6H, *J* = 6.8 Hz, −CH(C*H*_*3*_)_2_), 2.01 (s, 3H, Ar-C*H*_*3*_), 3.14 (hept, 1H, *J* = 6.8 Hz, −C*H*(CH_3_)_2_), 6.96–7.04 (m*,* 1H, *H-b*), 7.09–7.16 (m*,* 3H, *aromatic protons*), 7.49 (t, 1H, *J* = 7.9 Hz, *H-c*), 8.07 (d, 1H, *J* = 8.8 Hz, *H-d*), 8.18 (s, 1H, *aromatic protons*), 8.59 (d, 1H, *J* = 6.8 Hz, *H-a*), 8.60–8.70 (m, 2H, *aromatic protons*), 9.10 (s,1H, −N*H*); ^13^C NMR (151 MHz, DMSO-*d*_6_): δ 15.4 (Ar-*C*H_3_), 22.5 (−CH(*C*H_3_)_2_), 27.4 (−*C*H(CH_3_)_2_), 89.4 (*C-f*), 112.3, 112.9 (*C-b*), 117.0, 118.3 (*C-d*), 125.3 (*C-a*), 127.1, 127.9 (*C–c*), 129.0, 133.7, 138.1, 141.6, (*C-e*), 146.6, 146.7, 147.7, 161.0, 162.1. MS (ESI): 403 (M+1). ESI-HRMS (*m*/*z*): [M + H]^+^calcd for C_23_H_23_N_4_O_3_, 403.1764; obsd. 403.1764.

## Materials and Methods

### Molecular Docking Studies

The X-ray structure of *h*DHODH in complex with MEDS433 (PDB code 6FMD)^38^ was downloaded from the Protein Data Bank^54^ and used for docking studies. Prior to docking, the protein loop corresponding to residues 217–225, which was unresolved in the X-ray structure, was automatically reconstructed by using Modeller software.^55^ Docking calculations were performed with GOLD software^56^ using the ChemScore fitness function. The region of interest for the docking calculations included all residues which stayed within 10 Å from the bound ligand in the reference X-ray structures. Compounds **1–19** were subjected to 100 genetic algorithm runs, in which the “allow early termination” option was deactivated, while the possibility for the ligand to flip ring corners was activated. All other settings were left as their defaults. The rmsd threshold for pose clustering was set to 2.0 Å. The best docked conformation belonging to the best cluster of solutions was considered for each ligand in each docking study.

### Molecular Dynamics Simulations

All MD simulations were carried out with AMBER 16 using ff14SB force field for the protein, while GAFF (General Amber force field) was used for the cofactors (flavin mononucleotide and orotic acid) and the different ligands, whose partial charges were calculated using the AM1-BCC method through the antechamber suite of AMBER16. The 19 ligand–protein complexes generated by docking, as well as the reference hDHODH-MEDS433 complex, were included in a parallelepiped box and solvated with a 15 Å water cap using the TIP3P explicit solvent model, while chloride ions were added for the neutralization of the systems. Prior to the MD simulations, a two-stage minimization protocol was used for each complex. A 5000 steps minimization, including 2000 steps of steepest descent (SD), followed by 3000 steps of conjugate gradient (CG), was initially performed. In this stage, a position restraint of 100 kcal/mol·Å^2^ was applied on all receptors, cofactors, and ligand heavy atoms in order to uniquely minimize the positions of the water molecules and the orientation of rotatable polar hydrogens. A second minimization stage including 5000 total steps of SD/CG algorithms was then performed applying a harmonic potential of 10 kcal/mol·Å^2^ only to the protein α-carbons, thus energy-minimizing the entire system. The energy-minimized systems were used as inputs for an MD simulation protocol adapted from previous studies,^57,58^ in which particle mesh Ewald (PME) electrostatics and periodic boundary conditions were used, with a cutoff of 10 Å for the non-bonded interactions. A time step of 2.0 fs was employed in the simulations since all bonds involving hydrogen atoms were kept fixed using SHAKE algorithm. For each complex, a constant volume MD simulation was carried out for the first 0.5 ns, during which the temperature of the system was increased from 0 to 300 K. The system was then equilibrated through 3 ns of constant-pressure simulation, which was carried out keeping the temperature at the constant value of 300 K by using a Langevin thermostat. Finally, an additional constant-pressure MD simulation stage of 46.5 ns was performed for each system for a total MD simulation time of 50 ns. In all MD stages, a harmonic potential of 10 kcal/(mol·Å^2^) was applied to the protein α-carbons. All the obtained MD trajectories were analyzed using the Cpptraj program implemented in AMBER 16.

### Binding Energy Evaluations

The evaluation of the binding free energy associated with the 19 ligand–protein complexes analyzed through MD simulations was carried out using AMBER 16, as previously described.^57^ The trajectories relative to the last 20 ns of each simulation were extracted and used for the calculation for a total of 200 snapshots (at a time interval of 100 ps) since the binding conformation of each analyzed ligand was found to be sufficiently stable in this time interval, which well represented the system along the entire MD simulation (Table S1). van der Waals electrostatic and internal interactions were calculated with the SANDER module of AMBER 16. MOLSURF program was employed to estimate the non-polar energies, while polar energies were calculated using the Poisson–Boltzmann (PB) methods with the MM-PBSA module of AMBER 16.^59,60^ A dielectric constant of 80 was used to represent the water phase in all calculations. For the gas phase, 10 different values of dielectric constant were used, ranging from 1 to 10. A total of 10 different binding free-energy evaluations were thus performed for each of the analyzed ligand–protein complexes.

### Enzymatic Assays

#### Protein Expression and Purification

BL21DE3-Gold (DE3) E. coli cells were transformed using the plasmid construct pFN2A–truncated *h*DHODH (31-395) (kindly given by Department of Oncology, University of Turin, Turin). The vector produces *h*DHODH as an *N*-terminal GST-fusion protein. Cells were grown at 37 °C in LB medium in the presence of ampicillin (0.1 mg/mL) and supplemented with 0.1 mM flavin mononucleotide (Cayman Chemical). After 20 h of growth, cells were induced with 0.8 mM isopropyl-d-thiogalactopyranoside at an OD600 of 0.5–0.7 at 28 °C for an additional 6 h. A cell pellet from 250 mL of culture was lysed in 20 mL of PBS (50 mM Na_2_HPO_4_, 50 mM NaH_2_PO_4_, 500 mM NaCl), which had been supplemented with 24 mg of lysozyme and 0.2% v/v protease inhibitor cocktail, incubated for 30 min over ice, and disrupted by sonication (total sonication time: 8 min with On/Off cycles of 10”/50″). Triton X-100 was added to the lysate to a final concentration of 1% before centrifugation at 14,000*g* for 40 min at 4 °C. The clarified supernatant was incubated with DNase I for 30 min at room temperature, supplemented with 2 mM dithiothreitol (DTT), and filtered through a 0.45 μm syringe filter as previously described by Sainas et al*.*^38^ The GST-fused enzyme was purified from the bacterial lysate using affinity chromatography on immobilized glutathione-sepharose columns (GE-HiTrap Protein G HP 1 mL). The GST tag was not cleaved for further analysis. All the reagents used in the protein expression and purification were supplied by Merck/Sigma-Aldrich if not otherwise specified.

#### *h*DHODH Inhibition Assay

The enzymatic inhibition assay was optimized for being performed on a 96-well plate and to achieve a higher throughput. For each well of the plate, a total volume of 200 μL was used: 5 μL of purified GST-*h*DHODH, 60 μL of 2,6-dichloroindophenol (DCIP) 500 μM, 20 μL of coenzyme Q10 enzyme 100 μM, 20 μL of dihydroorotate (DHO) 500 μM, and Tris–HCl pH 8 up to a final volume of 200 μL. Inhibitory activity was assessed by monitoring the reduction of DCIP, which is associated with the oxidation of dihydroorotate as catalyzed by the DHODH enzyme. The enzyme was pre-incubated for 5 min at 37 °C in Tris–HCl pH8 with coenzyme Q10, with DCIP (50 μM) and with the compounds to be tested used at different concentrations (final DMSO concentration 0.1% v/v). The reaction was initiated by the addition of DHO (500 μM), and the absorbance kinetic reduction was monitored at λ = 650 nm using a multi-plate reader (Tecan, M1000Pro). In order to assess the minimum and maximum absorbance values of the enzymatic reaction, a Min control value was obtained by measuring the absorbance without DHO. Similarly, a Max value was obtained by measuring the absorbance with DHO but no inhibitor. A blank reduction calculation was also performed by measuring the absorbance values using 180 μL of Tris–HCl and 20 μL of coenzyme Q10. The absorbance values were read every 10 s for a total read time of 10 min at 37 °C. The initial rate was measured in the first 5 min (ε = 10 400 M^–1^ cm^–1^) and an IC_50_ value was calculated^41^ using GraphPad Prism 7 software. Values are means ± SE of three independent experiments.

### Cell-Based Assays

#### Cell Lines and Drugs

The AML human cell lines THP1 (acute monocytic leukemia), MV4-11 (acute monocytic leukemia), U937 (acute pro-monocytic leukemia), and Jurkat (T-cell leukemia) were cultured in complete RPMI 1640 (Invitrogen Life Technologies, Gaithersburg, MD), supplemented with 10% heat-inactivated fetal bovine serum (FBS) and 1% penicillin/streptomycin (GIBCO, Invitrogen, Milan, Italy). Dipyridamole (Persantin, Boehringer Ingelheim, Germany) was purchased. *h*DHODH inhibitors were solubilized in DMSO (Sigma-Aldrich, Milan, Italy), and final dilutions of the drugs were made in the culture medium.

#### CFSE-Based Cytotoxic Activity Assay

Briefly, the Jurkat cell line was incubated with 1 μM carboxyfluorescein diacetate succinimidyl ester dye (CFSE, Vybrant CFDA SE cell tracer kit; Molecular Probes, Invitrogen Carlsbad, CA) at 10^7^/ml for 20 min at 37 °C. At the end of the labeling process, cells were resuspended and washed in RPMI 1640 supplemented with 1% fetal bovine serum. Cells were then resuspended in RPMI 1640 supplemented with 10% FBS and incubated for 20 min at 37 °C. Cells were centrifuged and plated (1 × 10^4^ in 200 μL of medium), with increasing concentrations of the *h*DHODH inhibitors (1 μM to 100 μM) for 3 days. Cells were harvested, and 1 μg/mL of propidium iodide was added to assign the ratio of cell death. The percentage of specific lysis was calculated in accordance with the following equation: [dead targets in sample (%) – spontaneously dead targets (%))/(100-spontaneously dead targets (%))] × 100. Spontaneous lysis was obtained by incubating cell lines in the medium supplemented with the corresponding percentage of DMSO used for the dilution of compounds. Values represent the concentration that induces significant cytotoxic effects (≥30%).

#### Annexin Assay

For the determination of EC_50_, 1 × 10^4^ THP1 or U937 cells were plated in 96-well round-bottom plates and treated with increasing doses of *h*DHODH inhibitors from 0.1 μM to 50 μM. For drug combinations, 1 × 10^4^ THP1 or MV4-11 cells were plated in 96-well round-bottom plates and treated with *h*DHODH inhibitors at 0.1 μM or 1 μM, dipyridamole 1.0 μM, and uridine (5 μM Merck, Milan, Italy) in a volume of 200 μL of medium for 3 days. After 3 days of culture, the apoptotic assay was performed using the Annexin V-FITC Kit (Miltenyi Biotec, Italy), according to the manufacturer’s instructions. The apoptotic cells were acquired on FacsVerse and analyzed using Kaluza software version 1.2 (Beckman Coulter Fullerton, CA).

#### Differentiation Assay

For the determination of EC_50_, 1 × 10^4^ THP1 or U937 cells were plated in 96-well round-bottom plates and treated with increasing doses of the *h*DHODH inhibitors from 0.1 to 50 μM. For drug combinations, 1 × 10^4^ THP1 cells were plated in 96-well round-bottom plates and treated with *h*DHODH inhibitors at 0.1 μM or 1 μM and dipyridamole 1.0 μM in a volume of 200 μL of medium for 2 days. The differentiation pathway was monitored by analyzing the expression of CD11b (APC-conjugated BD Bioscience San Jose, CA, USA) or CD14 (APC-conjugated Beckam Coulter CA, USA) via flow cytometry analysis. Cells were washed and resuspended in the staining buffer [phosphate-buffered saline (PBS), 2% bovine serum albumin, 1 mM EDTA] and incubated with antibodies at 4 °C for 45 min. Samples were acquired on an FACSVerse (BD-Biosciences San Jose CA) and dead cells were excluded from the analyses, according to the use of propidium iodide (Sigma-Aldrich, Milan, Italy). Data were processed using Kaluza software version 1.2 (Beckman Coulter Fullerton, CA).

#### Statistical Analysis

Statistical analyses were performed on Prism software, version 5.0 (GraphPad Software, San Diego, CA). Data are reported as means ± SD. For the determination of EC_50_, a nonlinear regression model was applied. For multiple comparisons, one-way ANOVA tests were performed and combined with Tukey’s tests post hoc analyses. Moreover, in this case, a p-value < 0.05 was considered significant.

For in vivo study, group comparisons were made using two-way ANOVA tests, followed by Tukey’s tests post hoc analyses. A *p*-value < 0.05 was considered significant.

*AML xenograft mouse model* THP1 (2×10^6^ cells per mouse) were resuspended in 100 μL of PBS and injected subcutaneously on the left flank of 8 week old female NOD/SCID/γ chain–/– (NSG) immunocompromised mice (*n* = 4 per group, Charles River Laboratories, Calco, Italy). Mice were randomized into control or treated groups when the volume of the masses reached approximately 0.2 cm^3^ and were palpable. Mice were treated intraperitoneally for 13 consecutive days with the vehicle (10% DMSO and 90% corn oil from Merck, Milan Italy), MEDS433 20 mg/kg, or compound 20 mg/kg. Tumor sizes were regularly measured twice weekly with a caliber, and the tumor volume was calculated as *V* = 1/2 × (length × width^2^). Mice were treated following the European guidelines and with the approval of the Italian Ministry of Health (Authorization n. 42/2020-PR). Mice were treated once daily and euthanized 24 h after the last administration. At the end of the experiment, tumors were excised to determine their weight and volume.

#### Chemophysical Profiling; Solubility Assay at pH 7.4

Solubility was assayed in PBS: 12 mM with NaCl 137 mM and KCl 2.7 mM, pH 7.4. Each solid compound (1 mg) was added to 1 mL of PBS. The samples were shaken in an orbital shaker at 25 °C for 24 h. These suspensions were filtered through a PTFE 0.45 μm filter (VWR), and the solutions were chromatographically analyzed using a Perkin Elmer UHPLC instrument, equipped with a reverse-phase (RP) C18 Phenomenex column (2.1 × 100 mm, 1.7 μm particle size). Gradient elution: the ratio of eluents A and B (0.1% trifluoroacetic acid in water and 0.1% trifluoroacetic acid in acetonitrile, respectively) changed linearly from 60% A–40% B to 0% A–100% B in 12 min, followed by 5 min in isocratic elution at 100% of eluent B and then 4 min in equilibration elution to reset the starting conditions. The flow rate was 0.5 mL/min. The standard injection volume was either 2 or 4 μL for poorly soluble compounds. The detection system was a Perkin Elmer diode-array detector. The wavelengths that were monitored for each compound were defined according to the compound’s own absorption spectrum. Solubility, expressed as μM concentration of the saturated solution, was calculated via interpolation with external calibration curves that were obtained with solutions of each compound in acetonitrile.

#### Clog *P* and log *D* (pH 7.4)

Clog *P* values were calculated using the Bio-Loom program for Windows, Version 1.5 (BioByte). The partition coefficients between *n*-octanol and PBS at pH 7.4 (log *D*^7.4^) were obtained using the shake-flask technique at room temperature. In the shake-flask experiments, 50 mM of PBS pH 7.4 was used as the aqueous phase. The organic (*n*-octanol) and aqueous phases were mutually saturated by shaking for 4 h. The compounds were solubilized in the buffered aqueous phase at the highest concentration compatible with solubility, and appropriate amounts of *n*-octanol were added. The two phases were shaken for about 20 min, by which time the partitioning equilibrium of solutes had been reached, and then centrifuged (10,000 rpm, 10 min). The concentration of the solutes was measured in the aqueous phase using a UV spectrophotometer (Varian Cary 50BIO); absorbance values (recorded for each compound at the wavelength of maximum absorption) were interpolated in calibration curves obtained using standard solutions of the compounds (*r*^2^ > 0.99). Each log *D* value is an average of at least six measurements.

### Protein Expression, Purification, and Crystallization

The Open Reading Frame of a N-terminal truncated version of *h*DHODH (Met30-Arg396) was subcloned into a pET-19b (GenScript) expression vector using NdeI/BamHI recognized sequences as restriction sites. The vector allows the inducible production of a N-terminal His-tagged protein exploiting *E. coli* BL21(DE3) (Novagen) as the expression system. By applying standard techniques, an aliquot of *E. coli* BL21(DE3) was transformed with the target construct and grown on an agar plate for 12 h at 37 °C. The bacterial culture was then inoculated in 1 L of 2xYT medium in the presence of ampicillin (50 μg/mL) and grown at 37 °C/200 rpm to OD_600_ = 0.7. The expression of the recombinant protein was induced with the addition of 0.2 mM isopropyl-1-thio-d-galactopyranoside, and cells were further incubated at 16 °C/200 rpm for 21 h. Cells were harvested by centrifugation at 6000 rpm at 4 °C for 10 min. 7 g of wet cells was resuspended in 70 mL of lysis buffer [50 mM HEPES pH 7.8, 300 mM NaCl, 10% v/v glycerol, 0.25% (w/v) UDAO] supplemented with complete EDTA-free protease inhibitor cocktail (Merck) and DNAse. Cells were lysed with a Sonics Vibra-Cell VC 130 Ultrasonic Homogenizer (Strokes: 10; Pulse: 30”; Stop: 1’; Amplitude: 45). The lysate was centrifuged at 17,000 rpm for 45 min at 4 °C (Beckman Coulter Avanti Centrifuge J-26 XP), and the cleared cell lysate was loaded onto a 2 mL Qiagen Ni-NTA Agarose column pre-equilibrated with lysis buffer. After washing the resin with 20 CV of the wash buffer (lysis buffer supplemented with 50 mM imidazole), the protein was eluted with the elution buffer (lysis buffer supplemented with 300 mM imidazole). The eluted fractions were checked on SDS-PAGE, and all positive fractions were pooled and concentrated using an Amicon 15-30000 MWCO centrifugal concentrator (Merck). The concentrated protein was loaded onto a Hiload Superdex 200 16/600 (GE Healthcare) column, pre-equilibrated with a size exclusion buffer [100 mM HEPES pH 7.0, 400 mM NaCl, 10% (v/v) glycerol, 1 mM EDTA, 0.25% (w/v) UDAO]. Size exclusion chromatography resulted in a single well-defined peak at the elution volume consistent with the monomeric structure in solution. The aliquots corresponding to the elution peak measured at 350 and 442 nm wavelengths were concentrated using an Amicon 15-30000 MWCO up to 10 mg/ml. Protein quantification was performed by Bradford assay in a calibrated system with a Savatec Onda spectrophotometer UV-21.

For crystallization assay, the purified protein was mixed with orotate (ORO) and inhibitor **4** to reach the final concentration of 2 mM for both ligands (the *h*DHODH inhibitor was added starting from stock solutions of 50 mM in 100% DMSO) and subsequently incubated at 4 °C for 1 h.

1 L of protein-inhibitor complex was mixed with 1 L of a reservoir solution consisting of 0.2 M KBr, 0.2 M KSCN, 0.1 M sodium acetate pH 5.0, 25–35% (v/v) PEG 400, and 2–5% (v/v) PGA-LM (Molecular Dimension Limited) undergoing crystallization trials by means of the sitting drop vapor diffusion method at 20 °C. After 1 week, some small needle-shaped crystals grew in drops and these were used as seeds for optimizing the crystallization process that allowed, after 3 months, to obtain single crystals suitable for X-ray diffraction under the conditions of 2 M ammonium sulfate, 100 mM sodium acetate pH 4.8, and 30% v/v glycerol. Crystals were then flash-frozen in liquid nitrogen and underwent X-ray diffraction experiments.

#### X-ray Data Collection, Structure Determination, and Refinement

X-ray diffraction data were collected at the European Synchrotron Radiation Facility (ESRF), France, on beamline ID23-2.^61^ The data were indexed using XDS program; then they were integrated and scaled to a resolution of 1.85 Å using the Aimless utilities of the CCP4 Program Suite version 7.1.018.^62^ The structure was determined by molecular replacement with phenix-PHASER^63^ using the structure of DHODH from 2PRH PDB as a search model. Manual model building was performed with Coot program,^64^ and figures were generated with PyMol.^65^

Data collection and refinement statistics are listed in the Table S6. The atomic coordinates of compound **4** in complex with *h*DHODH have been deposited in −the Protein Data Bank under accession code 7Z6C.

### In Vitro Metabolic Behavior

#### Incubation Conditions of Rat Microsomes and Sample Preparation

Rat-liver microsomes (Sprague–Dawley, male, Sigma-Aldrich; 20 mg/mL protein concentration) were incubated with the candidate compound solution (5 μM final concentration, with 1% DMSO) and TRIS buffer (0.1 M, pH = 7.4). The regenerating system, which slowly generated coenzyme units over the incubation time, leading to a better reproduction of in vivo behavior, was composed of MgCl_2_ (3.3 mM), NADP^+^ (1.3 mM), Glu6P (3.5 mM), and Glu6Pdehydrogenase (0.5 U/mL). In addition to the compound sample (“C”) which was incubated with active microsomes and the regenerating system, the drug-free matrix blank sample (B) and two other series of specimens were used to provide more information for the interpretation of experimental results:

In the “C1” control sample, the tested drug was incubated with heat-inactivated microsomes (inactivation via a 15 min heating cycle at 90 °C).
In the “C2” control sample, there was no regenerating system in the incubation medium.

The incubation time started with the addition of the microsome suspension (0.5 mg/mL). Time point t_0_ was immediately obtained, and the following samples were collected at 15, 30, 60, and 120 min in order to evaluate short-term stability and longer-term stability.

Metabolic reactions were stopped by adding 100 μL of cooled acetonitrile to the 100 μL sample of the incubation mixture. Samples were centrifuged to provoke protein precipitation, and the supernatants were immediately stocked at −80 °C, until analysis, to prevent the potential degradation of unstable products.

### Identification of Metabolites Using High-Resolution Mass Spectrometry

The products of in vitro metabolism were identified using a high-resolution mass spectrometer (LTQ Orbitrap XL, Thermo Scientific) coupled to an HPLC instrument (1200 system Agilent). All analytes were separated on an XBridge MS C18 column (100 × 2.1 mm, 3.5 μm particle size) maintained at 30 °C. The elution mixture was composed of solvent A (0.1% formic acid in water for the positive ionization mode and 10 mM ammonium acetate for the negative ionization mode) and solvent B (acetonitrile). The elution gradient was from 10 to 99% of solvent B in 24 min, held at 99% for 4 min and re-equilibrated for 4 min at 10% of solvent B. The injection volume and flow rate were 4 μL and 200 μL/min, respectively. Mass spectrometric analyses were performed in positive- and negative-ion modes using a Supporting Information source under the following conditions: a heated capillary temperature of 240 °C and a spray voltage of 4 kV (positive ions) or 2.4 kV (negative ions). Accurate mass measurements were obtained using full-scan mass spectra (resolving power *R* = 30000; mass range *m*/*z* 150–800 Da) and with data-dependent MS2 acquisition, in which the four most abundant ions of the previous full-scan spectrum were selected for fragmentation. The MS2 spectra were acquired in low resolution with an Ion Trap analyzer and allowed the identification of the main characteristic fragments for each metabolite..

## Supporting Information Available

Computational analysis for compounds **1**–**19**; solubility of compounds **1**, **4**, **5**, **17**, **18**, and **19** and p*K*_a_ of compound **11**; flow cytometry plots for compounds **4** and **18**; differentiation induced by compound **4** or **MEDS433**, alone or in combination with dipyridamole, on THP1 cells; compound **4** and its metabolite MSMS2 spectra; data collection and refinement statistics for PDBID: 7Z6C; synthesis of compounds **39–88** and characterization of ^1^H NMR and ^13^C NMR spectra for all synthetized compounds; and HRMS and UHPLC analysis of representative compounds (PDF)
Molecular formula strings (CSV)
Docking data of *h*DHODH-3 complex (PDB)
Docking data of *h*DHODH-4 complex (PDB)

## Supplementary Material

jm2c00496_si_002.csv

jm2c00496_si_003.pdb

jm2c00496_si_004.pdb

## Accession Codes

The coordinates of compound **4** in complex with human DHODH have been deposited in the Protein Data Bank (PDBID: 7Z6C), and the authors will release the atomic coordinates and experimental data upon article publication.

## Abbreviations

CFSE: carboxyfluorescein diacetate succinimidyl ester
DCIP: dichloroindophenol
DHO: dihydroorotate
DTT: dithiothreitol
ETC: electron transport chain
FMN: flavin mononucleotide
*h*DHODH: human dihydroorotate dehydrogenase
hENT1/2: human equilibrative nucleoside transporter
MD: molecular dynamics
PB: Poisson–Boltzmann
TFA: trifluoroacetic acid

## References

[ref1] Evans D. R.; Guy H. I. Mammalian Pyrimidine Biosynthesis: Fresh Insights into an Ancient Pathway. J. Biol. Chem. 2004, 279, 33035–33038. 10.1074/jbc.r400007200. 15096496

[ref2] Boschi D.; Pippione A. C.; Sainas S.; Lolli M. L. Dihydroorotate Dehydrogenase Inhibitors in Anti-infective Drug Research. Eur. J. Med. Chem. 2019, 183, 111681–111702. 10.1016/j.ejmech.2019.111681. 31557612

[ref3] Madak J. T.; Bankhead A. 3rd; Cuthbertson C. R.; Showalter H. D.; Neamati N. Revisiting the Role of Dihydroorotate Dehydrogenase as a Therapeutic Target for Cancer. Pharmacol. Ther. 2019, 195, 111–131. 10.1016/j.pharmthera.2018.10.012. 30347213

[ref4] Lolli M. L.; Sainas S.; Pippione A. C.; Giorgis M.; Boschi D.; Dosio F. Use of Human Dihydroorotate Dehydrogenase (hDHODH) Inhibitors in Autoimmune Diseases and New Perspectives in Cancer Therapy. Recent Pat Anticancer Drug Discov 2018, 13, 86–105. 10.2174/1574892812666171108124218. 29119937

[ref5] Sykes D. B. The Emergence of Dihydroorotate Dehydrogenase (DHODH) as a Therapeutic Target in Acute Myeloid Leukemia. Expert Opin Ther Targets 2018, 22, 893–898. 10.1080/14728222.2018.1536748. 30318938 PMC6457988

[ref6] Sainas S.; Pippione A. C.; Boschi D.; Gaidano V.; Circosta P.; Cignetti A.; Dosio F.; Lolli M. L. DHODH Inhibitors and Leukemia: an Emergent Interest for New Myeloid Differentiation Agents. Drugs of the Future 2018, 43, 823–834. 10.1358/dof.2018.043.11.2856492.

[ref7] Xiong R.; Zhang L.; Li S.; Sun Y.; Ding M.; Wang Y.; Zhao Y.; Wu Y.; Shang W.; Jiang X.; Shan J.; Shen Z.; Tong Y.; Xu L.; Chen Y.; Liu Y.; Zou G.; Lavillete D.; Zhao Z.; Wang R.; Zhu L.; Xiao G.; Lan K.; Li H.; Xu K. Novel and Potent Inhibitors Targeting DHODH are Broad-Spectrum Antivirals Against RNA Viruses Including Newly-Emerged Coronavirus SARS-CoV-2. Protein Cell 2020, 11, 723–739. 10.1007/s13238-020-00768-w. 32754890 PMC7402641

[ref8] Li G.; De Clercq E. Therapeutic Options for the 2019 Novel Coronavirus (2019-nCoV). Nat Rev Drug Discov 2020, 19, 149–150. 10.1038/d41573-020-00016-0. 32127666

[ref9] Lewis T. A.; Sykes D. B.; Law J. M.; Muñoz B.; Rustiguel J. K.; Nonato M. C.; Scadden D. T.; Schreiber S. L. Development of ML390: a Human DHODH Inhibitor that Induces Differentiation in Acute Myeloid Leukemia. ACS Med Chem Lett 2016, 7, 1112–1117. 10.1021/acsmedchemlett.6b00316. 27994748 PMC5150668

[ref10] Sykes D. B.; Kfoury Y. S.; Mercier F. E.; Wawer M. J.; Law J. M.; Haynes M. K.; Lewis T. A.; Schajnovitz A.; Jain E.; Lee D.; Meyer H.; Pierce K. A.; Tolliday N. J.; Waller A.; Ferrara S. J.; Eheim A. L.; Stoeckigt D.; Maxcy K. L.; Cobert J. M.; Bachand J.; Szekely B. A.; Mukherjee S.; Sklar L. A.; Kotz J. D.; Clish C. B.; Sadreyev R. I.; Clemons P. A.; Janzer A.; Schreiber S. L.; Scadden D. T. Inhibition of Dihydroorotate Dehydrogenase Overcomes Differentiation Blockade in Acute Myeloid Leukemia. Cell 2016, 167, 171–186. 10.1016/j.cell.2016.08.057. 27641501 PMC7360335

[ref11] Burnett A. K.; Russell N. H.; Hills R. K.; Bowen D.; Kell J.; Knapper S.; Morgan Y. G.; Lok J.; Grech A.; Jones G.; Khwaja A.; Friis L.; McMullin M. F.; Hunter A.; Clark R. E.; Grimwade D. Arsenic Trioxide and All-Trans Retinoic Acid Treatment for Acute Promyelocytic Leukaemia in All Risk Groups (AML17): Results of a Randomised, Controlled, Phase 3 Trial. Lancet Oncol 2015, 16, 1295–1305. 10.1016/s1470-2045(15)00193-x. 26384238

[ref12] Lo-Coco F.; Avvisati G.; Vignetti M.; Breccia M.; Gallo E.; Rambaldi A.; Paoloni F.; Fioritoni G.; Ferrara F.; Specchia G.; Cimino G.; Diverio D.; Borlenghi E.; Martinelli G.; Di Raimondo F.; Di Bona E.; Fazi P.; Peta A.; Bosi A.; Carella A. M.; Fabbiano F.; Pogliani E. M.; Petti M. C.; Amadori S.; Mandelli F.; Italian G. C. G. Front-line Treatment of Acute Promyelocytic Leukemia with AIDA Induction Followed by Risk-adapted Consolidation for Adults Younger than 61 Years: Results of the AIDA-2000 Trial of the GIMEMA Group. Blood 2010, 116, 3171–3179. 10.1182/blood-2010-03-276196. 20644121

[ref13] Platzbecker U.; Avvisati G.; Cicconi L.; Thiede C.; Paoloni F.; Vignetti M.; Ferrara F.; Divona M.; Albano F.; Efficace F.; Fazi P.; Sborgia M.; Di Bona E.; Breccia M.; Borlenghi E.; Cairoli R.; Rambaldi A.; Melillo L.; La Nasa G.; Fiedler W.; Brossart P.; Hertenstein B.; Salih H. R.; Wattad M.; Lübbert M.; Brandts C. H.; Hänel M.; Röllig C.; Schmitz N.; Link H.; Frairia C.; Pogliani E. M.; Fozza C.; D’Arco A. M.; Di Renzo N.; Cortelezzi A.; Fabbiano F.; Döhner K.; Ganser A.; Döhner H.; Amadori S.; Mandelli F.; Ehninger G.; Schlenk R. F.; Lo-Coco F. Improved Outcomes with Retinoic Acid and Arsenic Trioxide Compared with Retinoic Acid and Chemotherapy in Non-High-Risk Acute Promyelocytic Leukemia: Final Results of the Randomized Italian-German APL0406 Trial. J Clin Oncol 2017, 35, 605–612. 10.1200/jco.2016.67.1982. 27400939

[ref14] Dolgin E. The Race for Antiviral Drugs to Beat COVID - and the Next Pandemic. Nature 2021, 592, 340–343. 10.1038/d41586-021-00958-4. 33854246

[ref15] Immunic, ImmunicTherapeutics., Inc. Receives First Regulatory Approval from German Health Authority BfArM to Initiate a Phase 2 Clinical Trial (NCT04379271) of its Selective Oral DHODH Inhibitor, IMU-838, in COVID-19 Patients, 2020.

[ref16] Luban J.; Sattler R. A.; Mühlberger E.; Graci J. D.; Cao L.; Weetall M.; Trotta C.; Colacino J. M.; Bavari S.; Strambio-De-Castillia C.; Suder E. L.; Wang Y.; Soloveva V.; Cintron-Lue K.; Naryshkin N. A.; Pykett M.; Welch E. M.; O’Keefe K.; Kong R.; Goodwin E.; Jacobson A.; Paessler S.; Peltz S. W. The DHODH Inhibitor PTC299 Arrests SARS-CoV-2 Replication and Suppresses Induction of Inflammatory Cytokines. Virus Res. 2021, 292, 198246–198257. 10.1016/j.virusres.2020.198246. 33249060 PMC7690341

[ref17] Coelho A. R.; Oliveira P. J. Dihydroorotate Dehydrogenase Inhibitors in SARS-CoV-2 Infection. Eur J Clin Invest 2020, 50, e13366–e13371. 10.1111/eci.13366. 32735689 PMC7435507

[ref18] Calistri A.; Luganini A.; Mognetti B.; Elder E.; Sibille G.; Conciatori V.; Del Vecchio C.; Sainas S.; Boschi D.; Montserrat N.; Mirazimi A.; Lolli M. L.; Gribaudo G.; Parolin C. The New Generation hDHODH Inhibitor MEDS433 Hinders the In Vitro Replication of SARS-CoV-2 and Other Human Coronaviruses. Microorganisms 2021, 9, 1731–1746. 10.3390/microorganisms9081731. 34442810 PMC8398173

[ref19] Kaur H.; Sarma P.; Bhattacharyya A.; Sharma S.; Chhimpa N.; Prajapat M.; Prakash A.; Kumar S.; Singh A.; Singh R.; Avti P.; Thota P.; Medhi B. Efficacy and Safety of Dihydroorotate Dehydrogenase (DHODH) Inhibitors ″Leflunomide″ and ″Teriflunomide″ in Covid-19: a Narrative Review. Eur. J. Pharmacol. 2021, 906, 174233–174241. 10.1016/j.ejphar.2021.174233. 34111397 PMC8180448

[ref20] Luganini A.; Sibille G.; Mognetti B.; Sainas S.; Pippione A. C.; Giorgis M.; Boschi D.; Lolli M. L.; Gribaudo G. Effective Deploying of a Novel DHODH Inhibitor Against Herpes Simplex Type 1 and Type 2 Replication. Antiviral Res. 2021, 189, 105057–105067. 10.1016/j.antiviral.2021.105057. 33716051

[ref21] Hahn F.; Wangen C.; Häge S.; Peter A. S.; Dobler G.; Hurst B.; Julander J.; Fuchs J.; Ruzsics Z.; Überla K.; Jäck H. M.; Ptak R.; Muehler A.; Gröppel M.; Vitt D.; Peelen E.; Kohlhof H.; Marschall M. IMU-838, a Developmental DHODH Inhibitor in Phase II for Autoimmune Disease, Shows Anti-SARS-CoV-2 and Broad-Spectrum Antiviral Efficacy In Vitro. Viruses 2020, 12, 1394–1412. 10.3390/v12121394. PMC7762174 33291455

[ref22] Kuduk S.; Deratt L. Substituted Fluoro(trifluoropropoxy)benzamide Urea Derivatives as Dihydroorotate Dehydrogenase Inhibitors and Their Preparation, Pharmaceutical Compositions and Use in the Treatment of Diseases. WO 2,021,038,490 A1, 2021.

[ref23] Sabnis R. W. Biaryl Compounds as Dihydroorotate Dehydrogenase Inhibitors for Treating Acute Myelogenous Leukemia (AML). ACS Med Chem Lett 2022, 13, 158–159. 10.1021/acsmedchemlett.2c00017. 35178168 PMC8842110

[ref24] Cisar J.; Kuduk S.; Deratt L.; Simonnet Y. R. F. Biaryl Amide Derivatives as DHODH Inhibitors and Their Preparation, Pharmaceutical Compositions and Use in the Treatment of Diseases; WO 2,021,070,132 A1, 2021.

[ref25] Sabnis R. W. Dihydroorotate Dehydrogenase Inhibitors for Treating Acute Myelogenous Leukemia (AML). ACS Med Chem Lett 2021, 12, 170–171. 10.1021/acsmedchemlett.0c00669. 33603957 PMC7883377

[ref26] Viswanadha S.; Vakkalanka S. K. V. S. Compositions Comprising a Dihydroorotate Dehydrogenase (DHODH) Inhibitor for the Treatment of Acute Myeloid Leukemia. WO 2,021,079,273 A1, 2021.

[ref27] Muthuppalaniappan M.; Bhavar P. K.; Viswanadha S.; Vakkalanka S. K. V. S.; Merikapudi G. S. Preparation of Biphenylcarbamoylbenzoic Acid Derivatives as Dihydroorotate Dehydrogenase Inhibitors. WO 2,011,138,665 A1, 2011.

[ref28] Dexter D. L.; Hesson D. P.; Ardecky R. J.; Rao G. V.; Tippett D. L.; Dusak B. A.; Paull K. D.; Plowman J.; DeLarco B. M.; Narayanan V. L.; Forbes M. Activity of a Novel 4-Quinolinecarboxylic Acid, NSC 368390 [6-Fluoro-2-(2′-fluoro-1,1′-biphenyl-4-yl)-3-methyl-4-quinolinecarboxylic acid sodium salt], Against Experimental Tumors. Cancer Res. 1985, 45, 5563–5568. 4053030

[ref29] Cao L.; Weetall M.; Trotta C.; Cintron K.; Ma J.; Kim M. J.; Furia B.; Romfo C.; Graci J. D.; Li W.; Du J.; Sheedy J.; Hedrick J.; Risher N.; Yeh S.; Qi H.; Arasu T.; Hwang S.; Lennox W.; Kong R.; Petruska J.; Moon Y. C.; Babiak J.; Davis T. W.; Jacobson A.; Almstead N. G.; Branstrom A.; Colacino J. M.; Peltz S. W. Targeting of Hematologic Malignancies with PTC299, a Novel Potent Inhibitor of Dihydroorotate Dehydrogenase with Favorable Pharmaceutical Properties. Mol. Cancer Ther. 2019, 18, 3–16. 10.1158/1535-7163.mct-18-0863. 30352802 PMC6318026

[ref30] Sabnis R. W. Heterocyclic Compounds as Dihydroorotate Dehydrogenase Inhibitors for Treating Acute Myelogenous Leukemia (AML). ACS Med Chem Lett 2021, 12, 1641–1642. 10.1021/acsmedchemlett.1c00532. 34795852 PMC8591715

[ref31] Christian S.; Merz C.; Evans L.; Gradl S.; Seidel H.; Friberg A.; Eheim A.; Lejeune P.; Brzezinka K.; Zimmermann K.; Ferrara S.; Meyer H.; Lesche R.; Stoeckigt D.; Bauser M.; Haegebarth A.; Sykes D. B.; Scadden D. T.; Losman J. A.; Janzer A. The Novel Dihydroorotate Dehydrogenase (DHODH) Inhibitor BAY 2402234 Triggers Differentiation and is Effective in the Treatment of Myeloid Malignancies. Leukemia 2019, 33, 2403–2415. 10.1038/s41375-019-0461-5. 30940908

[ref32] Clinicaltrials.gov, 2022. (accessed June 22, 2022). https://www.clinicaltrials.gov/ct2/show/NCT03404726?term=BAY2402234&draw=2&rank=2.

[ref33] Zhou J.; Yiying Quah J.; Ng Y.; Chooi J. Y.; Hui-Min Toh S.; Lin B.; Zea Tan T.; Hosoi H.; Osato M.; Seet Q.; Ooi L. A. G.; Lindmark B.; McHale M.; Chng W. J. ASLAN003, a Potent Dihydroorotate Dehydrogenase Inhibitor for Differentiation of Acute Myeloid Leukemia. Haematologica 2020, 105, 2286–2297. 10.3324/haematol.2019.230482. 33054053 PMC7556493

[ref34] Clinicatrial.gov, 2022. (accessed June 22, 2022).https://www.clinicaltrials.gov/ct2/show/NCT03451084?term=ASLAN003&draw=2&rank=1.

[ref35] Schwartsmann G.; Dodion P.; Vermorken J. B.; ten Bokkel Huinink W. W.; Joggi J.; Winograd B.; Gall H.; Simonetti G.; van der Vijgh W. J.; van Hennik M. B.; Crespeigne N.; Pinedo H. M. Phase I Study of Brequinar Sodium (NSC 368390) in Patients with Solid Malignancies. Cancer Chemother Pharmacol 1990, 25, 345–351. 10.1007/bf00686235. 2306795

[ref36] Peters G. J. Re-evaluation of Brequinar Sodium, a Dihydroorotate Dehydrogenase Inhibitor. Nucleos Nucleot. Nucleic Acids 2018, 37, 666–678. 10.1080/15257770.2018.1508692. 30663496

[ref37] Peters G. J.; Kraal I.; Pinedo H. M. In vitro and in vivo studies on the combination of Brequinar sodium (DUP-785; NSC 368390) with 5-fluorouracil; effects of uridine. Br. J. Cancer 1992, 65, 229–233. 10.1038/bjc.1992.46. 1739622 PMC1977736

[ref38] Sainas S.; Pippione A. C.; Lupino E.; Giorgis M.; Circosta P.; Gaidano V.; Goyal P.; Bonanni D.; Rolando B.; Cignetti A.; Ducime A.; Andersson M.; Järvå M.; Friemann R.; Piccinini M.; Ramondetti C.; Buccinnà B.; Al-Karadaghi S.; Boschi D.; Saglio G.; Lolli M. L. Targeting Myeloid Differentiation Using Potent 2-Hydroxypyrazolo[1,5- a]pyridine Scaffold-Based Human Dihydroorotate Dehydrogenase Inhibitors. J. Med. Chem. 2018, 61, 6034–6055. 10.1021/acs.jmedchem.8b00373. 29939742

[ref39] Sainas S.; Giorgis M.; Circosta P.; Gaidano V.; Bonanni D.; Pippione A. C.; Bagnati R.; Passoni A.; Qiu Y.; Cojocaru C. F.; Canepa B.; Bona A.; Rolando B.; Mishina M.; Ramondetti C.; Buccinnà B.; Piccinini M.; Houshmand M.; Cignetti A.; Giraudo E.; Al-Karadaghi S.; Boschi D.; Saglio G.; Lolli M. L. Targeting Acute Myelogenous Leukemia Using Potent Human Dihydroorotate Dehydrogenase Inhibitors Based on the 2-Hydroxypyrazolo[1,5-a]pyridine Scaffold: SAR of the Biphenyl Moiety. J. Med. Chem. 2021, 64, 5404–5428. 10.1021/acs.jmedchem.0c01549. 33844533 PMC8279415

[ref40] Lolli M. L.; Giorgis M.; Tosco P.; Foti A.; Fruttero R.; Gasco A. New Inhibitors of Dihydroorotate Dehydrogenase (DHODH) Based on the 4-Hydroxy-1,2,5-oxadiazol-3-yl (hydroxyfurazanyl) Scaffold. Eur. J. Med. Chem. 2012, 49, 102–109. 10.1016/j.ejmech.2011.12.038. 22245049

[ref41] Giorgis M.; Lolli M. L.; Rolando B.; Rao A.; Tosco P.; Chaurasia S.; Marabello D.; Fruttero R.; Gasco A. 1,2,5-Oxadiazole Analogues of Leflunomide and Related Compounds. Eur. J. Med. Chem. 2011, 46, 383–392. 10.1016/j.ejmech.2010.10.029. 21109332

[ref42] Sainas S.; Pippione A. C.; Giorgis M.; Lupino E.; Goyal P.; Ramondetti C.; Buccinnà B.; Piccinini M.; Braga R. C.; Andrade C. H.; Andersson M.; Moritzer A. C.; Friemann R.; Mensa S.; Al-Karadaghi S.; Boschi D.; Lolli M. L. Design, Synthesis, Biological Evaluation and X-Ray Structural Studies of Potent Human Dihydroorotate Dehydrogenase Inhibitors Based on Hydroxylated Azole Scaffolds. Eur. J. Med. Chem. 2017, 129, 287–302. 10.1016/j.ejmech.2017.02.017. 28235702

[ref43] Baumgartner R.; Walloschek M.; Kralik M.; Gotschlich A.; Tasler S.; Mies J.; Leban J. Dual Binding Mode of a Novel Series of DHODH Inhibitors. J. Med. Chem. 2006, 49, 1239–1247. 10.1021/jm0506975. 16480261

[ref44] Gradl S. N.; Mueller T.; Ferrara S.; Sheikh S. E.; Janzer A.; Zhou H.-J.; Friberg A.; Guenther J.; Schaefer M.; Stellfeld T.; Eis K.; Kroeber M.; Nguyen D.; Merz C.; Niehues M.; Stoeckigt D.; Christian S.; Zimmermann K.; Lejeune P.; Bruening M.; Meyer H.; Puetter V.; Scadden D. T.; Sykes D. B.; Seidel H.; Eheim A.; Michels M.; Haegebarth A.; Bauser M. Abstract 2: Discovery of BAY 2402234 by Phenotypic Screening: a Human Dihydroorotate Dehydrogenase (DHODH) Inhibitor in Clinical Trials for the Treatment of Myeloid Malignancies. Cancer Res. 2019, 79, 2 10.1158/1538-7445.am2019-2.

[ref45] Gaidano V.; Houshmand M.; Vitale N.; Carra G.; Morotti A.; Tenace V.; Rapelli S.; Sainas S.; Pippione A. C.; Giorgis M.; Boschi D.; Lolli M. L.; Cilloni D.; Cignetti A.; Saglio G.; Circosta P. The Synergism Between DHODH Inhibitors and Dipyridamole Leads to Metabolic Lethality in Acute Myeloid Leukemia. Cancers 2021, 13, 1–22. 10.3390/cancers13051003. PMC7957697 33670894

[ref46] Zhang L.; Zhang J.; Wang J.; Ren C.; Tang P.; Ouyang L.; Wang Y. Recent Advances of Human Dihydroorotate Dehydrogenase Inhibitors for Cancer Therapy: Current Development and Future Perspectives. Eur. J. Med. Chem. 2022, 232, 114176–114195. 10.1016/j.ejmech.2022.114176. 35151222

[ref47] Bonanni D.; Lolli M. L.; Bajorath J. Computational Method for Structure-Based Analysis of SAR Transfer. J. Med. Chem. 2020, 63, 1388–1396. 10.1021/acs.jmedchem.9b01931. 31939664

[ref48] Schymanski E. L.; Jeon J.; Gulde R.; Fenner K.; Ruff M.; Singer H. P.; Hollender J. Identifying Small Molecules Via High Resolution Mass Spectrometry: Communicating Confidence. Environ. Sci. Technol. 2014, 48, 2097–2098. 10.1021/es5002105. 24476540

[ref49] Pippione A. C.; Sainas S.; Boschi D.; Lolli M. L. Chapter Six - Hydroxyazoles as Acid Isosteres and their Drug Design Applications—Part 2: Bicyclic Systems. Applications of Heterocycles in the Design of Drugs and Agricultural Products, Meanwell N. A., Lolli M. L., Eds.; Academic Press, 2021; Vol. 134, pp 273–311.

[ref50] Sainas S.; Pippione A. C.; Giraudo A.; Martina K.; Bosca F.; Rolando B.; Barge A.; Ducime A.; Federico A.; Grossert S. J.; White R. L.; Boschi D.; Lolli M. L. Regioselective N-Alkylation of Ethyl 4-benzyloxy-1,2,3-triazolecarboxylate: A Useful Tool for the Synthesis of Carboxylic Acid Bioisosteres. J. Heterocycl. Chem. 2018, 56, 501–519. 10.1002/jhet.3426.

[ref51] Sainas S.; Pippione A. C.; Boschi D.; Lolli M. L. Hydroxyazoles as Acid Isosteres and their Drug Design Applications—Part 1: Monocyclic Systems. Applications of Heterocycles in the Design of Drugs and Agricultural Products, Meanwell N. A., Lolli M. L., Eds.; Academic Press, 2021; Vol. 134, pp 185–272.

[ref52] Sainas S.; Temperini P.; Farnsworth J. C.; Yi F.; Møllerud S.; Jensen A. A.; Nielsen B.; Passoni A.; Kastrup J. S.; Hansen K. B.; Boschi D.; Pickering D. S.; Clausen R. P.; Lolli M. L. Use of the 4-Hydroxytriazole Moiety as a Bioisosteric Tool in the Development of Ionotropic Glutamate Receptor Ligands. J. Med. Chem. 2019, 62, 4467–4482. 10.1021/acs.jmedchem.8b01986. 30943028 PMC6508984

[ref53] Sainas S.; Dosio F.; Boschi D.; Lolli M. L. Targeting Human Onchocerciasis: Recent Advances Beyond Ivermectin. Neglected Diseases: Extensive Space for Modern Drug Discovery 2018, 51, 1–38. 10.1016/bs.armc.2018.08.001.

[ref54] Berman H. M.; Westbrook J.; Feng Z.; Gilliland G.; Bhat T. N.; Weissig H.; Shindyalov I. N.; Bourne P. E. The Protein Data Bank. Nucleic Acids Res. 2000, 28, 235 10.1093/nar/28.1.235. 10592235 PMC102472

[ref55] Webb B.; Sali A. Comparative Protein Structure Modeling Using MODELLER. Curr. Protoc. Bioinf. 2016, 54, 561–563. 10.1002/cpbi.3. PMC5031415 27322406

[ref56] Verdonk M. L.; Cole J. C.; Hartshorn M. J.; Murray C. W.; Taylor R. D. Improved Protein-Ligand Docking Using GOLD. Proteins 2003, 52, 609–623. 10.1002/prot.10465. 12910460

[ref57] Poli G.; Lapillo M.; Jha V.; Mouawad N.; Caligiuri I.; Macchia M.; Minutolo F.; Rizzolio F.; Tuccinardi T.; Granchi C. Computationally Driven Discovery of Phenyl(piperazin-1-Yl)methanone Derivatives as Reversible Monoacylglycerol Lipase (MAGL) Inhibitors. J. Enzyme Inhib. Med. Chem. 2019, 34, 589–596. 10.1080/14756366.2019.1571271. 30696302 PMC6352951

[ref58] Pini E.; Poli G.; Tuccinardi T.; Chiarelli L. R.; Mori M.; Gelain A.; Costantino L.; Villa S.; Meneghetti F.; Barlocco D. New Chromane-Based Derivatives as Inhibitors of Mycobacterium Tuberculosis Salicylate Synthase (MbtI): Preliminary Biological Evaluation and Molecular Modeling Studies. Molecules 2018, 23, 1506–1519. 10.3390/molecules23071506. PMC6099841 29933627

[ref59] Tuccinardi T.; Manetti F.; Schenone S.; Martinelli A.; Botta M. Construction and Validation of a RET TK Catalytic Domain by Homology Modeling. J. Chem. Inf. Model. 2007, 47, 644–655. 10.1021/ci6004383. 17295463

[ref60] Kollman P. A.; Massova I.; Reyes C.; Kuhn B.; Huo S.; Chong L.; Lee M.; Lee T.; Duan Y.; Wang W.; Donini O.; Cieplak P.; Srinivasan J.; Case D. A.; Cheatham T. E. 3rd. Calculating Structures and Free Energies of Complex Molecules: Combining Molecular Mechanics and Continuum Models. Acc. Chem. Res. 2000, 33, 889–897. 10.1021/ar000033j. 11123888

[ref61] Flot D.; Mairs T.; Giraud T.; Guijarro M.; Lesourd M.; Rey V.; van Brussel D.; Morawe C.; Borel C.; Hignette O.; Chavanne J.; Nurizzo D.; McSweeney S.; Mitchell E. The ID23-2 Structural Biology Microfocus Beamline at the ESRF. J. Synchrotron Radiat. 2010, 17, 107–118. 10.1107/s0909049509041168. 20029119 PMC3025444

[ref62] The CCP4 Suite: Programs for Protein Crystallography. Acta Crystallogr D Biol Crystallogr 1994, 50, 760–763. 10.1107/s0907444994003112. 15299374

[ref63] Adams P. D.; Afonine P. V.; Bunkóczi G.; Chen V. B.; Davis I. W.; Echols N.; Headd J. J.; Hung L. W.; Kapral G. J.; Grosse-Kunstleve R. W.; McCoy A. J.; Moriarty N. W.; Oeffner R.; Read R. J.; Richardson D. C.; Richardson J. S.; Terwilliger T. C.; Zwart P. H. PHENIX: a comprehensive Python-Based System for Macromolecular Structure Solution. Acta Crystallogr D Biol Crystallogr 2010, 66, 213–221. 10.1107/s0907444909052925. 20124702 PMC2815670

[ref64] Emsley P.; Cowtan K. Model-Building Tools for Molecular Graphics. Acta Crystallogr D Biol Crystallogr 2004, 60, 2126–2132. 10.1107/s0907444904019158. 15572765

[ref65] Delano W. L. The PyMOL Molecular Graphics System, 2002. http://www.pymol.org.

