# Supplementary material for: Targeting Acute Myelogenous Leukemia Using Potent Human Dihydroorotate Dehydrogenase Inhibitors Based on the 2-Hydroxypyrazolo[1,5-a]pyridine Scaffold: SAR of the Aryloxyaryl Moiety
Source: J Med Chem. 2022 Sep 26;65(19):12701–24. doi: 10.1021/acs.jmedchem.2c00496 (PMC9574863; doi:10.1021/acs.jmedchem.2c00496)
Supplement: Supplementary file 1 — jm2c00496_si_001.pdf [file jm2c00496_si_001.pdf]

Supporting Information

Targeting Acute Myelogenous Leukemia Using Potent *Human* Dihydroorotate Dehydrogenase Inhibitors Based on the 2-Hydroxypyrazolo[1,5-*a*]pyridine Scaffold: SAR of the Aryloxyaryl Moiety

Stefano Sainas,[†, #] Marta Giorgis,[†,#] Paola Circosta, [‡§] Giulio Poli,[◦] Marta Alberti, [△] Alice Passoni,[⊥] Valentina Gaidano,[œ] Agnese C. Pippione,[†] Nicoletta Vitale,[₸] Davide Bonanni,[††] Barbara Rolando,[†] Alessandro Cignetti,[œ] Cristina Ramondetti,[¥] Alessia Lanno,[⊥] Davide M. Ferraris,[△] Barbara Canepa,[▪] Barbara Buccinnà,[¥] Marco Piccinini,[¥] Menico Rizzi, [△] Giuseppe Saglio,[‡, œ] Salam Al-Karadaghi,[¡] Donatella Boschi,[†] Riccardo Miggiano, [△] Tiziano Tuccinardi[◦] and Marco L. Lolli[†*]

† *Department of Drug Science and Technology, University of Turin, Via P. Giuria 9, 10125 - Turin, Italy;*

‡ *Department of Clinical and Biological Sciences, University of Turin, Regione Gonzole 10, 10043 - Orbassano (Turin), Italy;*

§ *Molecular Biotechnology Center, University of Turin, Via Nizza 52, 10126 - Turin, Italy;*

◦ *Department of Pharmacy, University of Pisa, Via Bonanno 6, 56126 Pisa, Italy;*

△ *Department of Pharmaceutical Sciences, University of Piemonte Orientale, Via G. Bovio 6, 28100- Novara, Italy;*

⊥ *Laboratory of Mass Spectrometry, Department of Environmental Health Sciences, Istituto di Ricerche Farmacologiche Mario Negri IRCCS, Via Mario Negri 2, 20156 Milan, Italy;*

œ *Division of Hematology and Cell Therapy, AO Ordine Mauriziano, Largo Filippo Turati, 62, 10128 - Turin, Italy;*

₸ *Department of Molecular Biotechnology and Health Sciences, University of Turin, Via Nizza 52, 10126 - Turin, Italy;*

† *Life Science Department, University of Modena Via Università 4, 41121 Modena, Italy;*

¥ *Department of Oncology, University of Turin, Via Michelangelo 27/B, 10125 - Turin, Italy;*

▪ *GEM FORLAB Via Ing. Comotto 36, 10014 Caluso (Turin), Italy;*

¡ *Department of Biochemistry and Structural Biology, Lund University, Naturvetarvägen 14, Box 124, 221 00 Lund, Sweden;*

# contributed equally

AUTHOR INFORMATION

Corresponding Authors

*E-mail: marco.lolli@unito.it.

Phone: +39 0116707180.

Fax: +39 0116707162 (M.L.L.).

Table of Content

Page S3	**Figure S1.** RMSD analysis of ligand and cofactors in the reference X-ray complex
Page S3	**Table S1.** Average ligand RMSD values during the MD simulations
Page S4	**Table S2**. Activities and best correlated binding energy values predicted for compounds **1-19**
Page S4	**Table S3**. Correlation of ligand activity and binding energy obtained using different ε_{int} values
Page S5	**Figure S2.** Partial charge distribution of compound **4**
Page S5	**Figure S3.** Predicted binding mode of compound **10** into *h*DHODH
Page S5	**Figure S4.** Predicted binding mode of compound **11** into *h*DHODH
Page S6	**Table S4.** Solubility of compounds **1**, **4**, **5**, **17**, **18**, **19**
Page S6	**Table S5.** pK_a of compound **11**
Page S7	**Figure S5**. Differentiating activity of compounds **4** and **18**. Flow cytometry plots
Page S7	**Figure S6.** Differentiation induced by compound **4** or **MEDS433**, alone or in combination with dipyridamole, on THP1 cells
Pages S8 – S12	**Figure S7 – S11.** Compound **4** and its Metabolites MSMS2 spectra
Page S13	**Table S6.** Data collection and refinement statistics
Pages S14 – S15	**Figure S12 – S14.** Diastereotopic effect of Ar-methylene protons in compounds **34** and **36** compared with compound **32**
Pages S16 – S17	Synthetic methodologies for the preparation of the anilines **39** and **54**
Pages S18 – S19	Synthetic methodologies for the preparation of the anilines **42** – **43**
Pages S20 – S23	Synthetic methodologies for the preparation of the anilines **43** – **45** and **55**
Pages S24 – S28	Synthetic methodologies for the preparation of the anilines **49** – **53**
Pages S29 – S32	Synthetic methodologies for the preparation of the anilines **46** - **48**
Pages S33 – S109	^{1}H-NMR,^{13}C-NMR,
Pages S110 – S187	HRMS and UHPLC

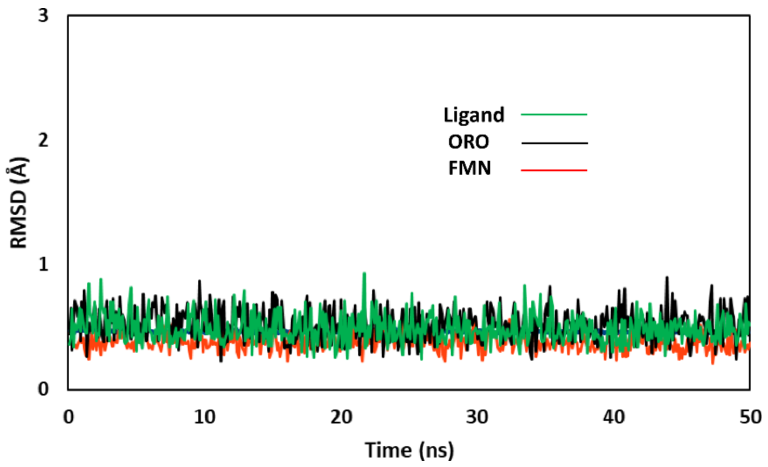

Figure S1. RMSD analysis of ligand, orotic acid (ORO) and flavin mononucleotide (FMN) disposition, with respect to the initial coordinates, during the MD study of the reference X-ray structure (PDB code 6FMD).

Compound	Mean RMSD (Å) during 50 ns	Mean RMSD (Å) during last 20 ns
**1**	0.8	0.8
**2**	1.9	1.9
**3**	2.3	2.3
**4**	0.8	0.8
**5**	1.2	1.3
**6**	1.4	1.4
**7**	1.9	1.9
**8**	2.2	2.3
**9**	2.3	2.1
**10**	2.4	2.5
**11**	2.0	2.0
**12**	1.2	1.3
**13**	1.1	1.1
**14**	1.0	1.1
**15**	1.4	1.3
**16**	2.1	1.9
**17**	1.2	1.3
**18**	0.8	0.8
**19**	1.0	1.0

Table S1. Average root-mean-square deviation (RMSD) of the disposition of each ligand during the corresponding MD simulations, with respect to its initial coordinates. The average ligand RMSD values are reported for both the whole 50 ns MD simulation and for the last 20 ns used for MM-PBSA evaluations.

Compound	Activity (pIC_{50})	ΔPBSA (kcal/mol)
**1**	7.30	-28.52
**2**	6.32	-26.37
**3**	6.40	-26.03
**4**	8.14	-33.48
**5**	6.94	-30.34
**6**	5.28	-24.44
**7**	5.00	-25.68
**8**	5.41	-25.02
**9**	5.66	-25.39
**10**	5.15	-23.91
**11**	5.00	-17.57
**12**	5.55	-21.64
**13**	5.00	-21.69
**14**	5.14	-20.61
**15**	5.00	-21.89
**16**	5.00	-21.65
**17**	5.66	-28.60
**18**	7.15	-32.17
**19**	7.74	-32.66

Table S2. Binding free energy values calculated for the 19 2-hydroxypyrazolo[1,5-a]pyridine compounds **1-19** using the MM-PBSA method with internal dielectric constant ε_{int} = 3. Values are expressed in kcal/mol. The corresponding activities of the ligands expressed as pIC_{50} values are reported.

ε_{int}	R^2
**1**	0.73
**2**	0.77
**3**	0.78
**4**	0.76
**5**	0.75
**6**	0.74
**7**	0.73
**8**	0.72
**9**	0.72
**10**	0.71

Table S3. R^2 values obtained for the correlation between the activity of compounds **1-19** expressed as pIC_{50} values and the corresponding binding free energies calculated using the MM-PBSA method with different internal dielectric constant (ε_{int}) values.

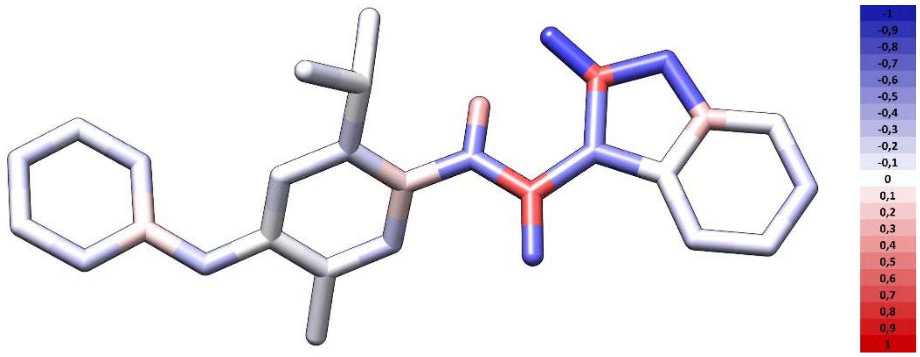

Figure S2. Partial charge distribution of compound **4** calculated based on the AM1-BCC method. The partial charge color scheme is shown on the side bar. Non-polar hydrogens are omitted for clarity.

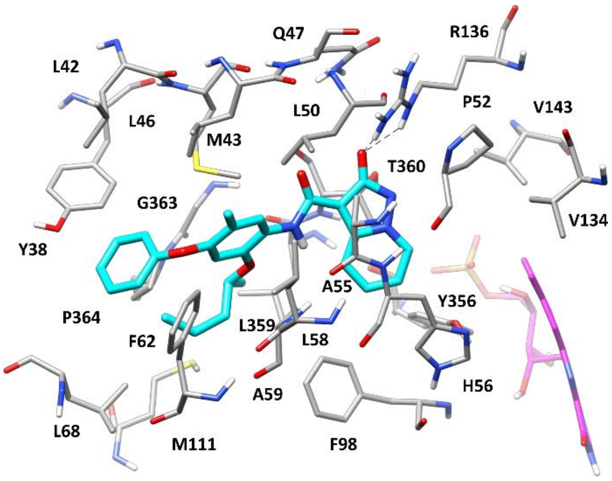

Figure S3. Minimized average structure of compound **10** (light blue) within *h*DHODH binding site (gray). Flavin mononucleotide is shown in magenta, while H-bonds are shown as black dashed lines

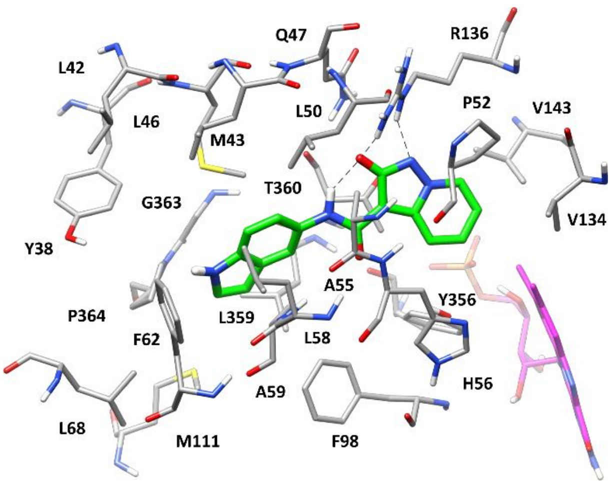

Figure S4. Minimized average structure of compound **11** (green) within *h*DHODH binding site (gray). Flavin mononucleotide is shown in magenta, while H-bonds are shown as black dashed lines

Compound	*h*DHODH[a]<br>IC_{50} ± SE (μM)	Solubility (μM)<br>in PBS
Brequinar [40]	0.0018 ± 0.0003	229
**1**	0.050 ± 0.005	< LOD
**4**	0.0072 ± 0.0009	< LOD
**5**	0.114 ± 0.011	< LOD
**15**	> 10	1850
**18**	0.070 ± 0.011	111
**19**	0.018 ± 0.004	< LOD

Table S4: Solubility in PBS of more representative compounds: **4**, **5, 15**, **18** and **19** compared to Brequinar and compound **1**. Limit of Detection (LOD) was set at 6 μM.

The pK_a for compound **11** was obtained in accordance with Albert A and Serjeant E. Ionization constants of Acids and Bases. London: Methuen and Co Ltd, **1962**.

Compound	Structure	pKa	log $D^{7.4}$ ± SD[c]
**11**	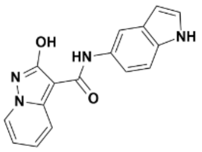	4.81 ±0.06<br>(By spectroscopic)<br><br>4.85 ±0.03<br>(Potentiometric titration)	0.45 ±0.02

Table S5. pK_a of compound **11.**

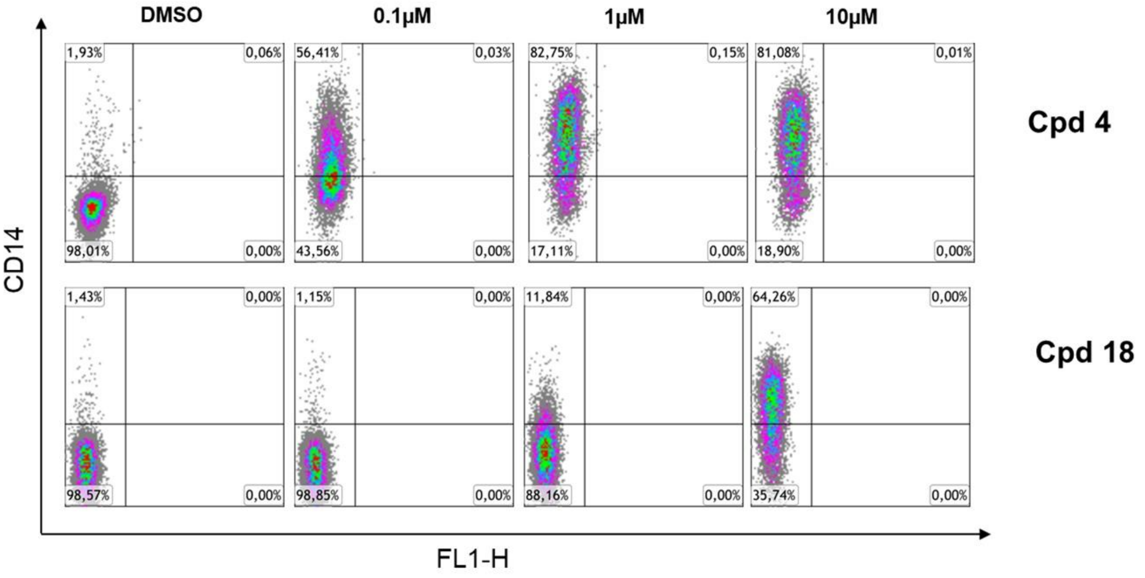

Figure S5. Differentiating activity of compounds **4** and **18**. Flow cytometry plots of 2 representative experiments on THP1 cells treated with compounds **4** (first row) and **18** (second row). THP1 differentiation was evaluated through CD14 expression.

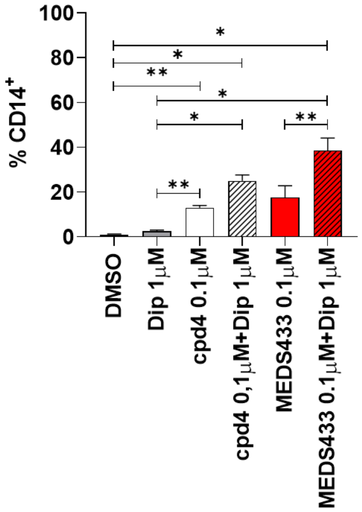

Figure S6. Differentiation induced by compound **4** or **MEDS433**, alone or in combination with dipyridamole, on THP1 cells; the differentiation analysis was performed on day 2 (n= 3). DMSO: dimethylsulfoxide. Dip: dipyridamole. Statistical significance: Anova/Tukey, * $p < 0.05$; **$p< 0.01$; ***$p< 0.001$

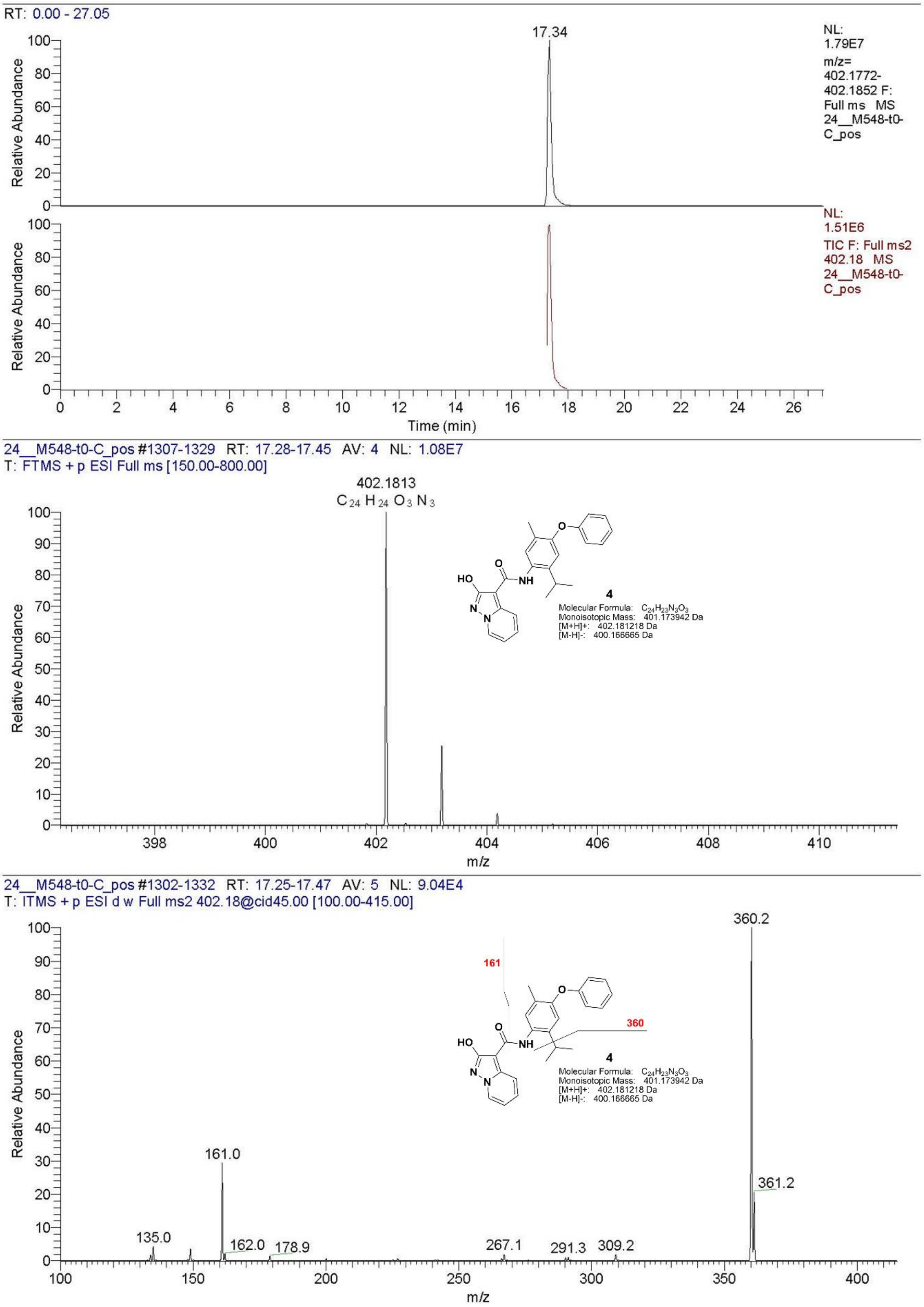

Figure S7. Positive MSMS2 spectra of compound **4**

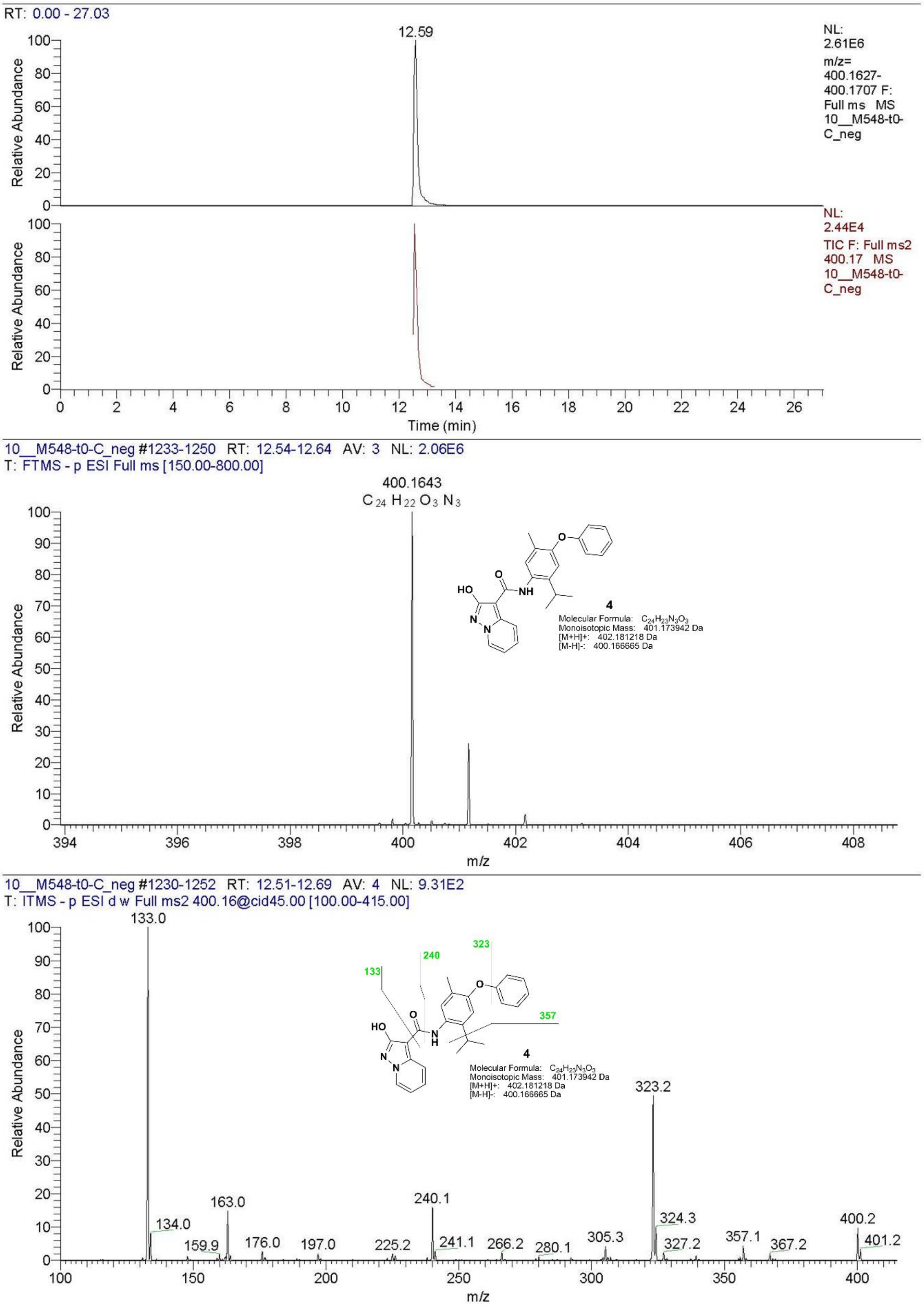

Figure S8. Negative MSMS2 spectra of compound **4**

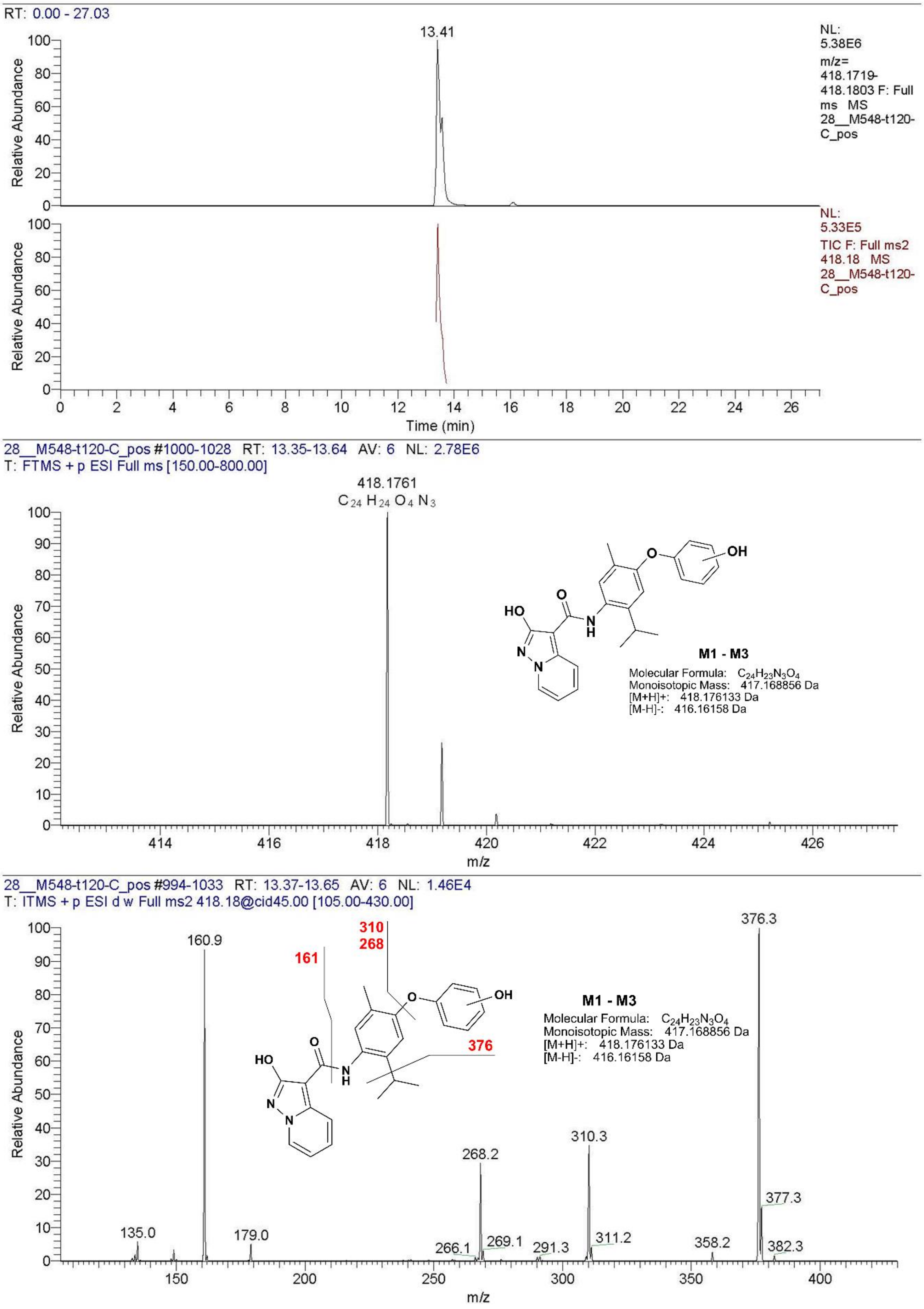

Figure S9. Positive MSMS2 spectra of metabolites **M1 – M3**

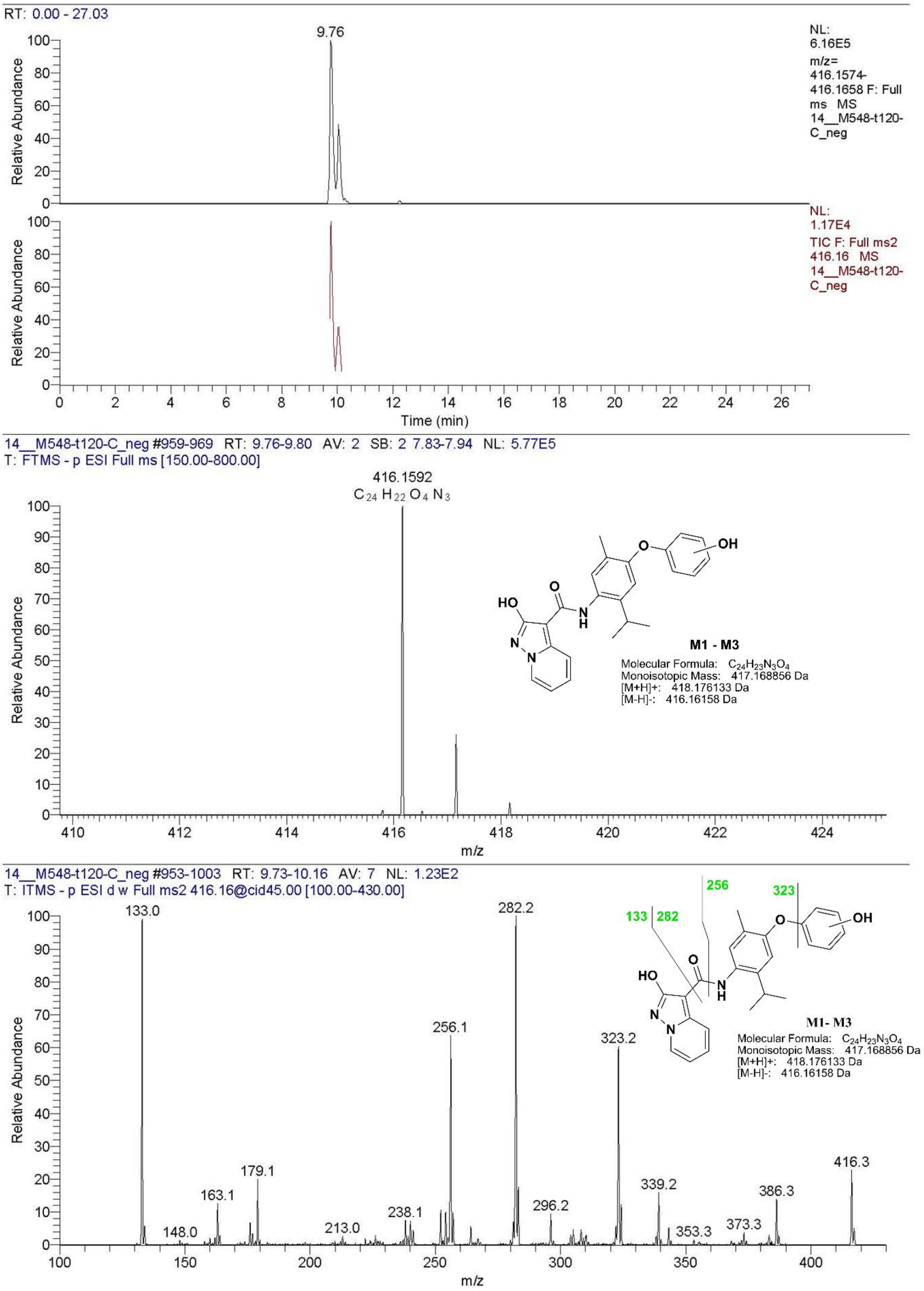

Figure S10. Negative MSMS2 spectra of metabolites **M1 – M3**

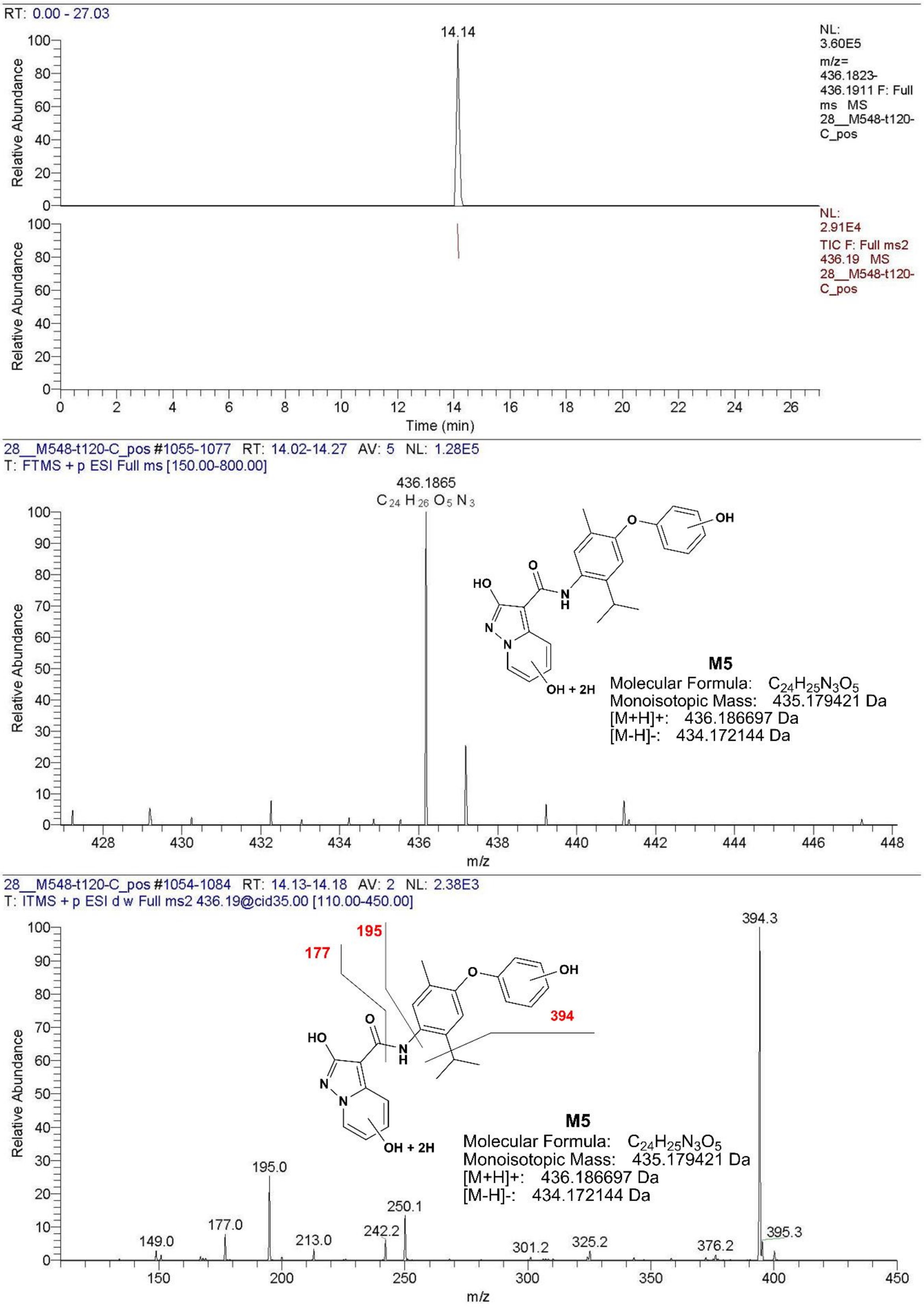

Figure S11. Positive MSMS2 spectra of metabolites **M5**

Table S6: Data collection and refinement statistics.

**dataset**	***h*DHODH: compound 4**
Data collection	
**Wavelength (Å)**	0.87313
**Resolution range (Å)**	45.55 - 1.85 (1.916-1.85)
**Unit cell constants**	
**a, b(Å)**	91.1
**c (Å)**	124.03
**α, β (°)**	90
**γ (°)**	120
**Space group**	P 32 2 1
**N° of molecules in asu**[a]	1
**N° of reflections**	334158 (50800)
**N° of unique reflections**	51326 (5071)
**R_{pim} (%)**	4.6 (30)
**R_{merge} (%)**	11.3 (76.3)
**R_{meas} (%)**	12.2 (82.3)
**I/σ**	8.8 (2.4)
**Completeness (%)**	99.74 (99.57)
**Multiplicity**	6.7 (6.8)
Refinement	
**R-factor (%)**	17.80 (24.27)
**R-free (%)**[b]	18.97 (27.55)
**N° of water molecules**	222
**N° of non-hydrogen atoms**	3129
Macromolecules	2821
Ligands	169
Solvent	213
**Average B-factor**	31.24
Macromolecules	30.62
Ligands	28.57
Solvent	40.66
**Average RMSD**[c]	
bond (Å)	0.066
angle (°)	0.94
Ramachandran statistics	
**Residues (%)**	
**in most favoured regions**	95.60
**in additional allowed regions**	4.40
**in disallowed regions**	0.00
**Rotamer outliers (%)**	0.33
**Clashscore**	4.61

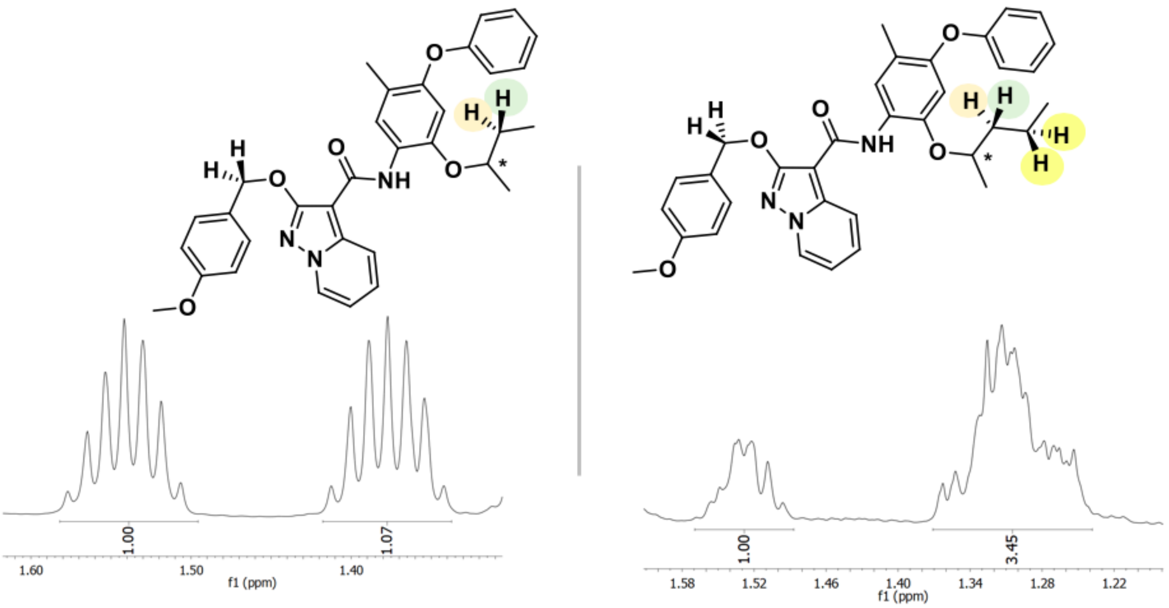

Figure S12: Resonance of methylene protons in compound **34** on the left, compound **36** on the right.

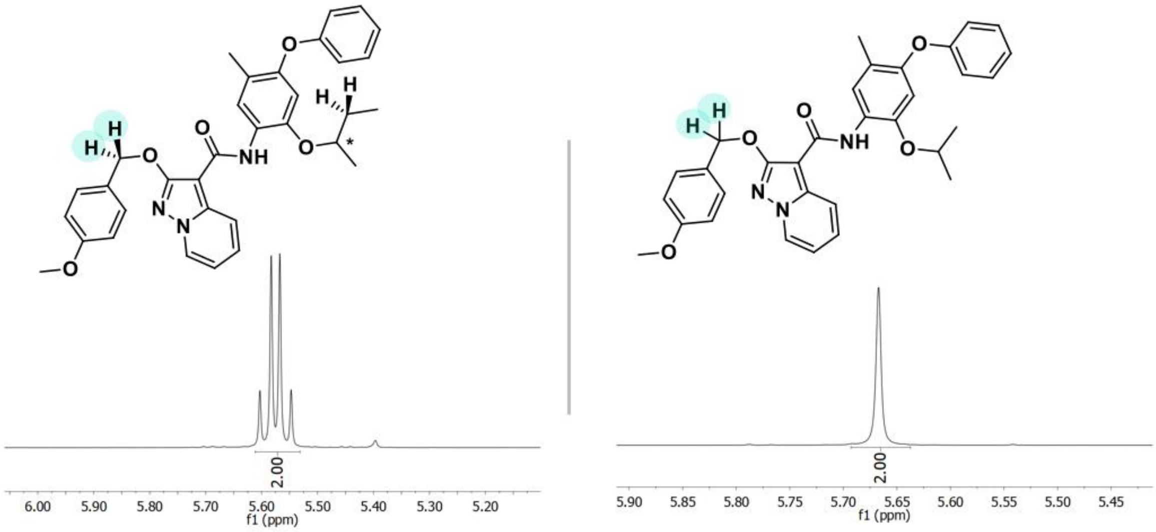

Figure S13: Resonance of Ar-methylene protons in compound **34** on the left, compound **32** on the right. They exhibit, in accordance with the expected splitting pattern, a *pseudo-quartet* which is the results of the roof effect on two distinct *doublets* and a *singlet* signal for the two non diastereotopic protons.

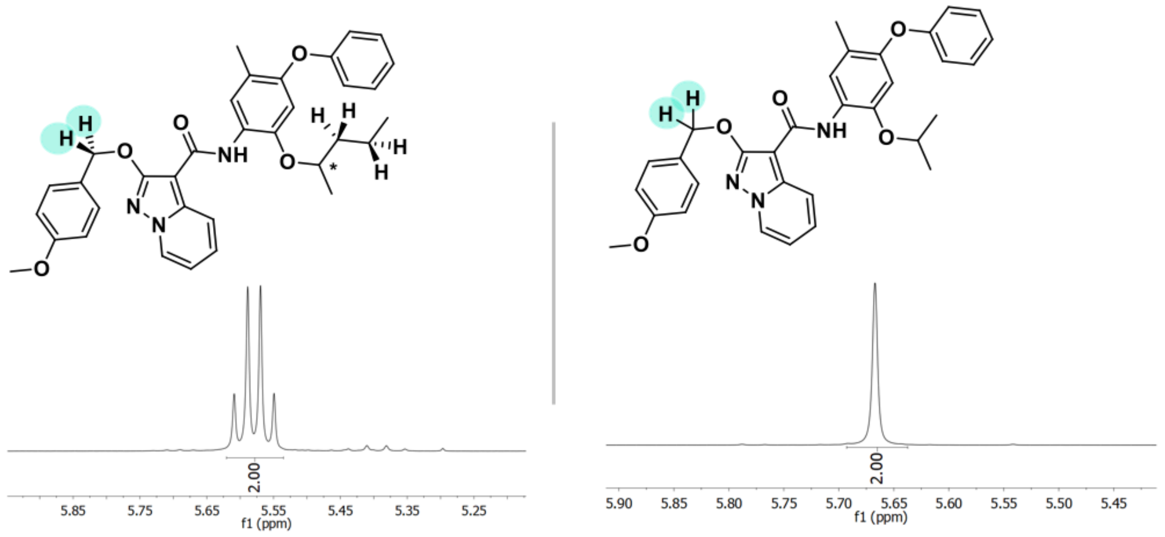

Figure S14: Resonance of Ar-methylene protons in compound **36** on the left, compound **32** on the right. They exhibit, in accordance with the expected splitting pattern, a *pseudo-quartet* which is the results of the roof effect on two distinct *doublets* and a *singlet* signal for the two non diastereotopic protons.

Synthetic methodologies for the preparation of the anilines **39 and 54**

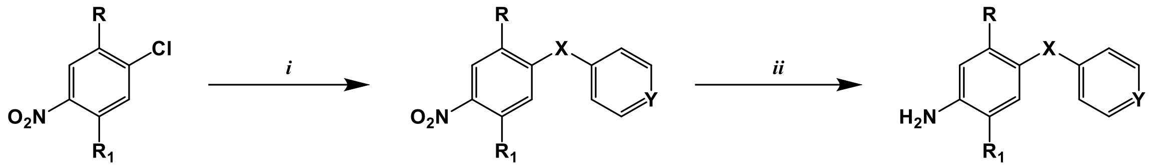

56, R = H, R_1 = Me;
57, R = R_1 = Me;

58, R = H, R_1 = Me, X = O, Y = C-H;
59, R = R_1 = Me, X = S, Y = N;

39, R = H, R_1 = Me, X = O, Y = C-H;
54, R = R_1 = Me, X = S, Y = N;

Scheme S1. Synthetic methodologies for the preparation of the anilines **39** and **54**: *i)* K_2CO_3, dry DMF; corresponding phenol, reflux; *ii)* $SnCl_2$, 37% w/w HCl, Dioxane, room temperature.

2-Methyl-1-nitro-4-phenoxybenzene (**58**). K_2CO_3 (644 mg, 4.66 mmol) was added to a solution of phenol (263 mg, 2.80 mmol) and 4-chloro-2-methyl-1-nitrobenzene (**56**, 400 mg, 2.33 mmol) in dry DMF (15 mL). The resulting solution was stirred at reflux over weekend under nitrogen atmosphere. The reaction mixture was then cooled to room temperature, quenched with water (130 mL) and the resulting mixture extracted with EtOAc (2 x 60 mL). The combined organic layers were washed with brine, dried over Na_2SO_4 and the solvent was evaporated under reduced pressure. The crude material was purified by flash chromatography (eluent: petroleum ether / EtOAc 98 / 2 v / v) to obtain a dark red oil. Yield: 56 %.^{1}H-NMR (600 MHz *Chloroform-d*): δ 2.60 (*s*, 3H, Ar-C*H3*), 6.82 – 6.87 (*m*, 2H, *H-t* and *H-r*), 7.08 (*d*, 2H, J = 7.9 Hz, *aromatic protons*), 7.24 (*t*, 1H, J = 7.4 Hz, *aromatic proton*), 7.43 (*t*, 2H, J = 7.9 Hz, *aromatic protons*), 8.05 (*d*, 1H, J = 8.8 Hz, *H-q*); ^{13}C NMR (151 MHz, *Chloroform-d*): δ 21.5 (Ar-*C*H3), 115.2 (*C-t*), 120.6 (*aromatic carbons*), 120.7 (*C-r*), 125.3 (*C-q*), 127.6 (*aromatic carbon*), 130.4 (*aromatic carbons*), 137.2 (*C-u*), 143.6 (*C-p*), 155.0 (*C-v*), 161.8 (*C-s*); MS (ESI) 230 (M+1).

4-((2,5-Dimethyl-4-nitrophenyl)thio)pyridine (**59**). K_2CO_3 (1.352 g, 2.0 eq.) was added to a solution of pyridine-4-thiol (0.543 g, 1.0 eq.) in dry DMF (20 mL); the resulting suspension was stirred at room temperature for 30 minutes, then 1-chloro-2,5-dimethyl-4-nitrobenzene (**57**, 0.998 g, 1.1 eq.) was added. The mixture was stirred overnight at 120°C, cooled to room temperature and then quenched in distilled water (150 mL). The aqueous phase was extracted using EtOAc (4 x 50 mL). The combined organic layer was washed with brine, dried on Na_2SO_4 and the solvent was evaporated under reduced pressure. The crude product was purified by flash chromatography (eluent: dichloromethane/EtOAc 50 / 50 v/v) to afford the title compound as a brown solid. Yield 58 %. ^{1}H-NMR (600 MHz, *Chloroform-d*): *δ* 2.39 (*s*, 3H, *Ar*-C*H*3), 2.55 (*s*, 3H, *Ar*-C*H*3), 6.96 (*dd*, 2H, J = 4.6, 1.6 Hz, *aromatic protons*), 7.44 (*s*, 1H, *H-t*), 7.94 (*s*, 1H, *H-q*), 8.43 (*dd*, 2H, J = 4.7, 1.4 Hz, *aromatic protons*). ^{13}C-NMR (151 MHz, *Chloroform-d*): *δ* 20.0 (*2-C*H3), 20.3 (*5-C*H3), 121.9 (*aromatic carbons*), 126.8 (*C-t*), 132.2 (*C-r*), 136.0 (*C-u*), 138.9 (*C-q*), 140.8 (*C-p*), 147.1 (*C-v*), 149.4 (*C-s*), 150.1 (*aromatic carbons*). MS (ESI) 259 (M-1).

General procedure for synthesis of compounds **39** and **54**. Tin chloride (5.0 eq.) was added to a solution of appropriate nitro derivatives (**58** – **59**, 1.0 eq.) in dioxane (10 mL) and 37 % w/w hydrochloric acid (10.0 eq.). The resulting mixture was stirred at room temperature overnight, then was neutralized with saturated aqueous $NaHCO_3$ (50 mL) then the pH was brought to 10 by adding 2M NaOH. The mixture was then extracted with EtOAc (3 x 50 mL). The combined organic layers

were dried over Na_2SO_4 and the solvent was evaporated under reduced pressure. The crude material was purified by flash chromatography (see details on each specific recipe).

2-Methyl-4-phenoxyaniline (**39**). Obtained from **58**. Flash chromatography eluent: from petroleum ether / EtOAc 98 / 2 v/v to 6 / 4 v/v. Yellow oil. Yield 78 %. ^{1}H-NMR (600 MHz, *Chloroform-d*): δ 2.16 (*s*, 3H, Ar-C*H₃*), 3.52 (*br s*,2H, -N*H₂*), 6.66 (*d*, 1H, J = 8.4 Hz, *H-q*), 6.76 (*dd*, 1H, J = 8.4, 2.7 Hz, *H-r*), 6.80 (*d*, 1H, J = 2.8 Hz, *H-t*), 6.93 (*d*, 2H, J = 8.0 Hz, *aromatic protons*), 7.01 (*t*, 1H, J = 7.4 Hz, *aromatic proton*), 7.28 (*dd*, 2H, J = 8.3, 7.6 Hz, *aromatic protons*), ^{13}C-NMR (151 MHz, *Chloroform-d*): δ 17.7 (Ar-CH_3), 116.0 (*C-u*), 117.3 (*aromatic carbons*), 118.8 (*C-q*), 122.1 (*C-t*), 122.4 (*aromatic carbon*), 124.1 (*C-r*), 129.6 (*aromatic carbons*), 141.0 (*C-p*), 148.5 (*C-s*), 159.1 (*C-v*); MS (ESI) 200 (M+1).

2,5-Dimethyl-4-(pyridin-4-ylthio)aniline **(54).** Obtained from **59**. Flash chromatography eluent: from petroleum ether/EtOAc 70 / 30 v/v to petroleum ether/EtOAc 60 / 40 v/v). Yellow solid. Yield 91 %. ^{1}H-NMR (600 MHz, *Chloroform-d*): δ 2.13 (*s*, 3H, Ar-CH_3), 2.24 (*s*, 3H, Ar-CH_3), 3.82 (*br s*, 2H, -NH_2), 6.66 (*s*, 1H, *H-q*), 6.82 (*dd*, 2H, J = 4.8, 1.4 Hz, *aromatic protons*), 7.22 (*s*, 1H, *H-t*), 8.28 (*d*, 2H, J = 5.8 Hz, *aromatic protons*). ^{13}C-NMR (151 MHz, *Chloroform-d*): δ 16.8 (*Ar-C*H_3)*, 20.3 (*Ar-C*H_3)*, 115.0 (*C-u*), 117.1 (*C-q*), 119.8 (*aromatic carbons*), 121.2 (*C-r*), 139.3 (*C-t*), 142.2 (*C-p*)*, 147.0 (*C-s*)*, 149.3 (*aromatic carbons*), 152.0 (*C-v*); MS (ESI) 231 (M+1).

Synthetic methodologies for the preparation of the anilines **42** – **43**

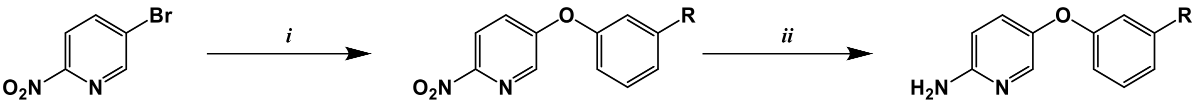

60

61, R = H;
62, R = CF_3;

42, R = H;
43, R = CF_3;

Scheme S2. Synthetic methodologies for the preparation of the anilines **42** – **43**: *i)* K_2CO_3, dry DMF; corresponding phenol, 70 °C; *ii)* $SnCl_2$, 37 % w/w HCl, Dioxane, room temperature.

General procedure for synthesis of compounds (**61** – **62**). Potassium carbonate (2.0 eq.) was added to a solution of the corresponding phenol (1.0 mmol) in dry DMF (20 mL) and the mixture was stirred at room temperature for 5 minutes. After the addition of 5-bromo-2-nitropyridine (**60**, 1.0 eq.) in DMF (20 mL) the reaction mixture was stirred overnight at 70 °C. The reaction solution was poured into water (125 mL) and extracted with ethyl acetate (3 x 50 mL). The combined organic layers were washed with brine, dried over Na_2SO_4 and the solvent evaporated under reduced pressure. The crude material was purified by flash chromatography.

2-Nitro-5-phenoxypyridine (**61**). Flash chromatography eluent: petroleum ether / EtOAc 95 / 5 v/v White solid (m.p. 91.5 – 93 °C from diisopropyl ether). Yield: 20 %. ^{1}H-NMR (600 MHz, *Chloroform-d*): δ 7.03 (*d*, 1H, J = 9.0 Hz, *H-q*), 7.17 (*d*, 2H, J = 8.0 Hz, *aromatic protons*), 7.31 (*t*, 1H, J = 7.4 Hz, *aromatic proton*), 7.46 (*t*, 2H, J = 7.9 Hz, *aromatic protons*), 8.47 (*dd*, 1H, J = 9.0, 2.6 Hz, *H-r*), 9.05 (*d*, 1H, J = 2.6 Hz, *H-t*); ^{13}C-NMR (151 MHz *Chloroform-d*): δ 111.5 (*C-t*), 121.6 (*aromatic carbons*), 126.2 (*C-r*), 130.1 (*aromatic carbons*), 135.0 (*aromatic carbon*), 140.4 (*C-p*), 145.2 (*C-q*), 152.9 (*C-v*), 167.1 (*C-s*); IR (KBr) ν (cm^{-1}): 3109, 3061, 2922, 2850, 2564, 1958, 1593, 1575, 1510, 1462, 1391, 1348, 1226, 1194, 1111; MS (ESI) 217 (M+1).

2-Nitro-5-[3-(trifluoromethyl)phenoxy]pyridine (**62**). Flash chromatography eluent: petroleum ether / EtOAc 80 / 20 v/v. Brownish solid (m.p. 89.2 – 89.8 °C from diisopropyl ether). Yield: 53 %. ^{1}H-NMR (600 MHz, DMSO-d_6): δ 7.57 (*d*, 1H, J = 7.8 Hz, *aromatic proton*), 7.62 – 7.81 (*m*, 4H, *aromatic protons*), 8.36 (*d*, 1H, J = 9.0 Hz, *aromatic proton*), 8.49 (*d*, 1H, J = 2.2 Hz, *aromatic proton*). ^{13}C-NMR (151 MHz *DMSO-d_6*): δ 116.9 (*q*, J = 3.8 Hz), 120.6, 122.2 (*q*, J = 3.8 Hz), 123.6 (*q*, J = 272.6 Hz, -CF_3), 124.1, 127.6, 131.3 (*q*, J = 32.4 Hz), 132.0, 139.2, 151.5, 154.9, 157.6. IR (KBr) ν (cm^{-1}): 3140, 3081, 2871, 1963, 1598, 1572, 1529, 1325, 1273, 1175, 1125, 1065; MS (ESI) 285 (M+1).

General procedure for synthesis of compounds **41** – **42**. Tin chloride (5.0 eq.) was added to a solution of appropriate nitro derivatives (**61** – **62**, 1.0 eq.) in dioxane (10 mL) and 37 % w/w hydrochloric acid (10.0 eq.). The resulting mixture was stirred at room temperature overnight, neutralized with saturated aqueous $NaHCO_3$ solution (50 mL) then the pH was brought to 10 with 2M NaOH. The mixture was then extracted with EtOAc (3 x 50 mL). The combined organic layers were dried over Na_2SO_4 and the solvent was evaporated under reduced pressure. The crude material was purified by flash chromatography.

5-Phenoxypyridin-2-amine (**41**). Flash chromatography eluent: petroleum ether / EtOAc 60 / 40 v/v. brownish solid (m.p. 65.7 – 68.8 °C from diisopropyl ether). Yield 88 %. ^{1}H-NMR (600 MHz, *Chloroform-d*): δ 3.57 (*br s*, 2H, -NH_2), 6.76 (*d*, 1H, J = 8.6 Hz, *aromatic proton*), 7.03 – 7.09 (*m*,

3H, *aromatic protons*), 7.11 (*t*, 1H, J = 7.4 Hz, *aromatic proton*), 7.34 (*t*, 2H, J = 7.8 Hz, *aromatic protons*), 7.72 (*d*, 1H, J = 2.8 Hz, *aromatic proton*); ^{13}C-NMR (151 MHz, *Chloroform-d*): δ 112.7, 119.9, 123.7, 126.9, 129.7, 134.3, 138.9, 155.9, 156.6. MS (ESI) 187 (M+1).

5-[3-(Trifluoromethyl)phenoxy]pyridine-2-amine (**42**). Flash chromatography eluent: petroleum ether / EtOAc 60 / 40 v/v. Brownish oil. Yield 81 %. ^{1}H -NMR (600 MHz, *Chloroform-d*): δ 4.46 (*br s*,2H, -NH_2), 6.55 (*d*, 1H, J = 8.8 Hz, *aromatic proton*), 7.09 (*dd*, 1H, J = 8.3, 2.0 Hz, *aromatic proton*), 7.15 (*s*, 1H, *aromatic proton*), 7.21 (*dd*, 1H, J = 8.8, 2.8 Hz, *aromatic proton*), 7.29 (*d*, 1H, J = 7.7, *aromatic proton*), 7.40 (*t*, 1H, J = 8.0, *aromatic proton*), 7.92 (*d*, 1H, J = 2.8, Hz, *aromatic proton*); ^{13}C-NMR (151 MHz *Chloroform-d*): δ 109.6, 113.7 (*q*, J = 3.7 Hz), 119.3 (*q*, J = 3.2 Hz), 120.0, 123.8 (*q*, J = 272.6 Hz, CF_3), 130.4, 131.1, 132.3 (*q*, J = 32.34 Hz), 140.6, 144.7, 155.8, 159.1; MS (ESI) 255 (M+1).

Synthetic methodologies for the preparation of the anilines **43** – **45** and **55**

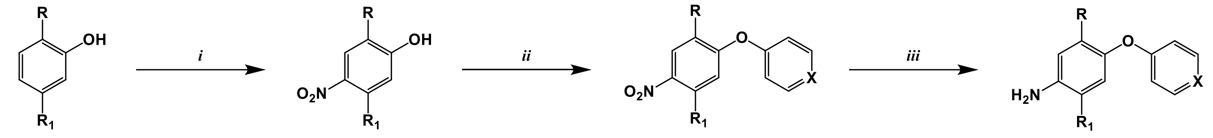

63, R = Me, R_1 = iPr;
64, R = iPr, R_1 = Me;

65, R = Me, R_1 = iPr;
66, R = iPr, R_1 = Me;

67, R = Me, R_1 = iPr, X = C-H;
68, R = iPr, R_1 = Me, X = C-H;
69, R = Me, R_1 = iPr, X = C-CF_3;
70, R = Me, R_1 = iPr, X = N;

40, R = Me, R_1 = iPr, X = C-H;
41, R = iPr, R_1 = Me, X = C-H;
44, R = Me, R_1 = iPr, X = C-CF_3;
55, R = Me, R_1 = iPr, X = N;

Scheme S3. Synthetic methodologies for the preparation of the anilines **40 – 41, 44** and **55**: *i)* CH_3COOH, HNO_3, O°C; *ii)* $Cu(OAc)_2$, Triethylamine, 1,2-dimethoxyethane, 40 °C; *iii)* $SnCl_2$, 37 % w/w HCl, Dioxane, room temperature.

General procedure for synthesis of 4-nitro thymol/carvacrol (**65** – **66**). Nitric acid (0.9 eq.) was added at 0 °C to a well stirred solution of the corresponding phenol (**63** – **64**, 1.0 eq.) in acetic acid (50 mL). The reaction mixture was slowly warmed to room temperature in 35 minutes. Then, the reaction mixture was quenched with $NaHCO_3$ until a pH of 6 and then extracted with EtOAc (3 x 100 mL). The combined organic layers were washed with brine, dried over Na_2SO_4 and the solvent evaporated under reduced pressure. The crude material was purified by flash chromatography.

5-Isopropyl-2-methyl-4-nitrophenol (**65**). Flash chromatography eluent: DCM /EtOAc 95 / 5 v/v. Yellow solid (m.p. 76.9 – 78.4 °C). Yield: 31 %. ^{1}H-NMR (600 MHz, *Chloroform-d*): δ 1.26 (*d*, 6H, J = 6.8 Hz, -CH(C*H*$_3$)$_2$), 2.25 (*s*, 3H, Ar-C*H*$_3$), 3.59 (*hept*, 1H, J = 6.8 Hz, -C*H*(CH$_3$)$_2$), 5.31 (br *s*, 1H, -O*H*), 6.80 (*s*, 1H, *H-t*), 7.71 (*s*, 1H, *H-q*); ^{13}C-NMR (151 MHz, *Chloroform-d*): δ 15.3 (Ar-*C*H$_3$), 23.7 (-CH(*C*H$_3$)$_2$), 28.5 (-*C*H(CH$_3$)$_2$), 113.3 (*C-t*), 122.5 (*C-r*), 128.1 (*C-q*), 142.2 (*C-p*), 144.3 (*C-u*), 158.1 (*C-s*); IR (KBr) ν (cm^{-1}) 3348, 2973, 1623, 1576, 1522, 1490, 1315, 1263, 1018, 787; MS (ESI) 194 (M-1).

2-Isopropyl-5-methyl-4-nitrophenol (**66**). Flash chromatography eluent: petroleum ether / DCM 60 / 40 v/v. Solid (m.p. 138.9 – 140.1 °C). Yield: 43 %. ^{1}H-NMR (600 MHz, *Chloroform-d*): δ 1.27 (*d*, 6H, J = 6.9 Hz, -CH(C*H*$_3$)$_2$), 2.57 (*s*, 3H, -C*H*$_3$), 3.20 (*hept*, 1H, J = 6.8 Hz, -C*H*(CH$_3$)$_2$), 5.76 (*s*, 1H, -O*H*), 6.68 (*s*, 1H, *H-t*), 8.00 (*s*, 1H, *H-q*); ^{13}C-NMR (151 MHz, *Chloroform-d*): δ 21.3 (-*C*H(CH$_3$)$_2$), 22.4 (-CH(*C*H$_3$)$_2$), 27.0 (Ar-*C*H$_3$), 118.9 (*C-t*), 124.8 (*C-q*), 133.6 (*C-r*), 134.6 (*C-u*), 142.3 (*C-p*), 157.4 (*C-s*); IR (KBr) ν (cm^{-1}): 3351, 2963, 1615, 1568, 1520, 1480, 1307, 1264, 1017, 758; MS (ESI) 196 (M+1).

General procedure for synthesis of compounds (**67 – 70**). Nitrothymol (**66**, 400 mg, 2.05 mmol 1.0) or nitrocarvacrol (**65**, 400 mg, 2.05 mmol) was added to a solution of $Cu(OAc)_2$ (1.5 g, 8.2 mmol) in dry dichloromethane (40 mL) under nitrogen atmosphere. After that 4 Å molecular sieves and triethylamine (622 mg, 0.9 mL, 6.15 mmol) were added, the obtained mixture was stirred for 30 minutes at room temperature. Then, the corresponding boronic acid (778 mg, 4.1 mmol) was added portion wise (0.5 mmol each 2 hours); the reaction mixture was stirred overnight at 40 °C. When no starting material was observed, the reaction mixture was quenched with 2M HCl (25 mL) and extracted with DCM (3 x 25 mL). The combined organic layers were washed with brine, dried over Na_2SO_4 and the solvent evaporated under reduced pressure. The crude material was purified by flash chromatography.

1-Isopropil-4-methyl-2-nitro-5-phenoxybenzene (**67**). Flash chromatography (eluent: *petroleum ether* / EtOAc 98 / 2 v/v) to afford the title compound as a brownish solid. Yield: 57 %. ^{1}H-NMR (600 MHz, *Chloroform-d*): δ 1.16 (*d*, 6H, J = 6.8 Hz, -CH(C*H*$_3$)$_2$), 2.29 (*s*, 3H, Ar-C*H*$_3$), 3.49 (*hept*, 1H, J = 6.8 Hz, -C*H*(CH$_3$)$_2$), 6.84 (*s*, 1H, *H-t*), 6.98 (*d*, 2H, J = 7.9 Hz, *aromatic protons*), 7.17 (*t*, 1H, J = 7.4 Hz, *aromatic proton*), 7.39 (*t*, 2H, J = 8.0 Hz, *aromatic protons*), 7.74 (*s*, 1H, *H-q*). ^{13}C-NMR (151 MHz, *Chloroform-d*): δ 15.9 (Ar-*C*H$_3$), 23.6 (-CH(*C*H$_3$)$_2$), 28.6 (-*C*H(CH$_3$)$_2$), 116.2 (*C-t*), 118.7 (*aromatic carbons*), 124.1 (*aromatic carbon*), 127.7 (*C-q*), 127.8 (*C-r*), 130.2 (*aromatic carbons*), 143.3 (*C-u*), 144.4 (*C-p*), 156.3 (*C-v*), 158.9 (*C-s*); IR (KBr) *v* (cm^{-1}): 2928, 1575, 1489, 1330, 1265, 1073, 1019, 758; MS (ESI) 272 (M+1).

1-Isopropil-4-methyl-5-nitro-2-phenoxybenzene (**68**). Flash chromatography (eluent: petroleum ether / EtOAc 95 / 5 v/v) to afford the title compound as a brownish oil. Yield: 16 %. ^{1}H-NMR (600 MHz, *Chloroform-d*): δ 1.30 (*d*, 6H, J = 6.9 Hz, -CH(C*H*$_3$)$_2$), 2.49 (*s*, 3H, Ar-C*H*$_3$), 3.38 (*hept*, 1H, J = 6.9 Hz, -C*H*(CH$_3$)$_2$), 6.61 (*s*, 1H, *H-t*), 7.02 (*d*, 2H, J = 7.9 Hz, *aromatic protons*), 7.20 (*t*, 1H, J = 7.4 Hz, *aromatic proton*), 7.41 (*t*, 2H, J = 7.8 Hz, *aromatic protons*), 8.05 (*s*, 1H, *H-q*); ^{13}C-NMR (151 MHz, *Chloroform-d*): δ 21.0 (-*C*H(CH)$_3$), 22.6 (-CH(*C*H$_3$)$_2$), 27.2 (Ar-CH$_3$), 119.7 (*aromatic carbons*), 120.4 (*C-t*), 124.5 (*aromatic carbon*), 124.6 (*C-q*), 130.3 (*aromatic carbons*), 134.0 (*C-r*), 137.9 (*C-u*), 144.0 (*C-p*), 156.1 (*C-v*), 158.7 (*C-s*). MS (ESI) 272 (M+1).

1-Isopropyl-4-methyl-2-nitro-5-(4-(trifluoromethyl)phenoxy)benzene (**69**). Flash chromatography (eluent: petroleum ether / dichloromethane 9 / 1 v/v) to afford the title compound as a pale-yellow solid (m.p. 65.4 – 67.3 °C from trituration with hexane). Yield 26 %. ^{1}H-NMR (600 MHz *Chloroform-d*): δ 1.21 (*d*, 6H, J = 6.8 Hz, -CH(C*H*$_3$)$_2$), 2.25 (*s*, 3H, Ar-C*H*$_3$), 3.48 (*hept*, 1H, J = 6.8 Hz, -C*H*(CH$_3$)$_2$), 6.96 (*s*, 1H, *H-t*), 7.01 (*d*, 2H, J = 8.6 Hz, *aromatic protons*), 7.62 (*d*, 2H, J = 8.5 Hz, *aromatic protons*), 7.74 (*s*, 1H, *H-q*); ^{13}C-NMR (151 MHz, *Chloroform-d*): δ 15.8 (Ar-*C*H$_3$), 23.6 (-CH(*C*H$_3$)$_2$), 28.6 (-*C*H(CH$_3$)$_2$), 117.5 (*aromatic carbons*), 118.2 (*C-t*), 124.1 (*q*, J = 271.7 Hz, -*C*F$_3$), 125.7 (*q*, J = 32.9 Hz, *aromatic carbons*), 127.5(*q*, J = 3.0 Hz, *aromatic carbons*), 127.7 (*C-q*), 128.8 (*C-r*), 143.4 (*C-u*), 145.6 (*C-p*), 156.9 (*C-v*), 159.4 (*C-s*); IR (KBr) *v* (cm^{-1}): 2975, 1916, 1611, 1513, 1323, 1246, 1164, 1122,1216; MS (ESI) 340 (M+1).

4-(5-Isopropyl-2-methyl-4-nitrophenoxy)pyridine **(70)**. All the reaction steps were performed under a nitrogenous atmosphere. 4Å molecular sieves were added to a suspension of copper acetate (1.75 g, 2.0 eq.), triethylamine (2.0 mL, 3.0 eq.) in 1,2-dimetoxyethane (50 mL). The suspension was stirred at room temperature for 1h then 5-isopropyl-2-methyl-4-nitrophenol (**65**, 0.940 g, 1.0 eq.) was added; the mixture was stirred at room temperature for 1h. After that, pyridin-4-yl boronic acid (2.5 eq.) was added to the mixture splitting the whole amount in 0.3 eq. every 30 minutes. The reaction mixture was heated at 90 °C overnight then was quenched in 250 mL of distilled water and the resulting mixture filtered in order to remove molecular sieves. The aqueous phase was exhaustively extracted using EtOAc; the combined organic layers were washed with brine, dried on Na_2SO_4 and the solvent was evaporated under reduced pressure. The crude product was purified by flash chromatography (eluent: DCM/EtOAc 80:20 v/v) to afford the title compound as a brown solid. Yield 19 %. ^{1}H-NMR (600 MHz, *Chloroform-d*): δ 1.23 (*d*, 6H, J = 6.8 Hz, -CH(C*H*$_3$)$_2$), 2.20 (*s*, 3H, Ar-C*H*$_{3-}$), 3.46 (*hept*, 1H, J = 6.8 Hz, -C*H*(CH$_3$)$_2$), 6.78 (*dd*, 2H, J = 4.8, 1.5 Hz, *aromatic protons*), 7.06 (*s*, 1H, *H-q*), 7.73 (*s*, 1H, *H-t*), 8.51 (*d*, 2H, J = 5.9 Hz, *aromatic protons*). ^{13}C-NMR (151 MHz, *Chloroform-d*): δ 15.7 (Ar-*C*H$_3$), 23.6 (-CH(*C*H$_3$)$_2$)28.6 (-*C*H(CH$_3$)$_2$), 118.8 (aromatic carbon), 119.7 (C-*t*), 127.8 (C-*q*),

129.5 (*C-r*), 143.4 (*C-u*), 146.4 (*C-p*), 151.9 (*aromatic carbon*), 155.3 (*C-v*), 163.7 (*C-s*). MS (ESI) 273 (M+1).

General procedure for synthesis of compounds **40 – 41**, **44** and **55**. Tin chloride (5.0 eq.) was added to a solution of appropriate nitro derivatives (**67** – **70**, 1.0 eq.) in dioxane (10 mL) and 37 % w/w hydrochloric acid (10.0 eq.). The resulting mixture was stirred at room temperature overnight, neutralized with saturated aqueous $NaHCO_3$ solution (50 mL) then the pH was brought to 10 with 2M NaOH. The mixture was then extracted with EtOAc (3 x 50 mL). The combined organic layers were dried over Na_2SO_4 and the solvent was evaporated under reduced pressure. The crude material was purified by flash chromatography.

2-Isopropyl-5-methyl-4-phenoxyaniline (**40**). Obtained from **67**. Flash chromatography eluent: petroleum ether / EtOAc 90 / 10 v/v) to afford the title compound as a red sticky oil. Yield 93 %. ^{1}H-NMR (600 MHz, *Chloroform-d*): δ 1.22 (*d*, 6H, J = 6.8 Hz, -CH(C*H*$_3$)$_2$), 2.07 (*s*, 3H, Ar-C*H*$_3$), 2.87 (*hept*, 1H, J = 6.8 Hz, -C*H*(CH$_3$)$_2$), 3.61 (*br s*, 2H, -N*H*$_2$), 6.59 (*s*, 1H, *H-q*), 6.81 (*s*, 1H, *H-t*), 6.83 (*d*, 2H, J = 8.2 Hz, *aromatic protons*), 6.97 (*t*, 1H, J = 7.3 Hz, *aromatic proton*), 7.26 (*t*, 2H, J = 7.9 Hz, *aromatic protons*). ^{13}C-NMR (151 MHz, *Chloroform-d*): δ 15.9 (-*C*H(CH$_3$)$_2$), 22.5 (-CH(*C*H$_3$)$_2$), 27.8 (Ar-*C*H$_3$), 115.7 (*aromatic carbons*), 118.5 (*C-q*), 118.9 (*aromatic carbon*), 121.3 (*C-t*), 128.7 (*C-u*), 129.6 (*aromatic carbons*), 132.2 (*C-r*), 140.2 (*C-p*), 146.0 (*C-s*), 159.4 (*C-v*); MS (ESI) 242 (M+1).

5-Isopropyl-2-methyl-4-phenoxyaniline (**41**). Obtained from **60**. Flash chromatography (eluent: petroleum ether / EtOAc 60 / 40 v/v) to afford the title compound as a brownish solid (m.p. 87.4 – 88.6 °C from trituration with diisopropyl ether). Yield 91 %. ^{1}H-NMR (600 MHz, *Chloroform-d*): δ 1.14 (*d*, 6H, J = 6.9 Hz, -CH(C*H*$_3$)$_2$), 2.10 (*s*, 3H, -C*H*$_3$), 3.09 (*hept*, 1H, J = 6.9 Hz, -C*H*(CH$_3$)$_2$), 3.51 (*s*, 2H, -N*H*$_2$), 6.64 (*s*, 1H, *H-q*), 6.68 (*s*, 1H, *H-t*), 6.86 (*d*, 2H, J = 8.2 Hz, *aromatic protons*), 6.96 (*t*, 1H, J = 7.3 Hz, *aromatic proton*), 7.25 (*t*, 2H, J = 7.8 Hz, *aromatic protons*); ^{13}C-NMR (151 MHz *Chloroform-d*): δ 17.2 (Ar-*C*H$_3$), 23.3 (-CH(*C*H$_3$)$_2$), 26.9 (-*C*H(CH$_3$)$_2$), 113.3 (*C-q*), 116.3 (*aromatic carbons*), 121.4 (*C-u*), 121.5 (*aromatic carbon*), 123.3 (*C-t*), 129.6 (*aromatic carbons*), 139.5 (*C-r*), 141.5 (*C-p*), 144.8 (*C-s*), 159.8 (*C-v*). IR (KBr) υ (cm^{-1}): 3450, 3337, 2957, 2968, 1628, 1587, 1512, 1489, 1462, 1412, 1293, 1213, 1166, 1000; MS (ESI) 242 (M+1).

2-Isopropyl-5-methyl-4-(4-(trifluoromethyl)phenoxy)aniline (**44**). Obtained from **69**. Flash chromatography (eluent: petroleum ether / EtOAc from 98 / 2 to 8 / 2 v/v) to afford the title compound as an orange solid (m.p. 76.4 - 78.7 °C from trituration with diisopropyl ether). Yield 67 %. ^{1}H-NMR (600 MHz, *Chloroform-d*): δ 1.22 (*d*, 6H, -J = 6.8 Hz, CH(C*H*$_3$)$_2$), 2.04 (*s*, 3H, Ar-C*H*$_3$), 2.88 (*hept*, 1H, J = 6.8 Hz, -C*H*(CH$_3$)$_2$), 3.68 (*br s*, 2H, -N*H*$_2$), 6.60 (*s*, 1H, *H-q*), 6.80 (*s*, 1H, *H-t*), 6.88 (*d*, 2H, J = 8.6 Hz, *aromatic protons*), 7.51 (*d*, J = 8.7 Hz, 2H, *aromatic protons*). ^{13}C-NMR (151 MHz, *Chloroform-d*): δ 15.8 (-CH(*C*H$_3$)$_2$), 22.4 (-CH(*C*H$_3$)$_2$), 27.8 (Ar-*C*H$_3$), 115.6 (*aromatic carbons*), 118.6 (*C-q*), 118.9 (*C-t*), 123.5 (*q*, J = 32.8 Hz, *aromatic carbon*), 124.6 (*q*, J = 271.7 Hz, -*C*F$_3$), 127.1 (*q*, J = 3.8, *aromatic carbons*), 128.7 (*C-u*), 132.5 (*C-r*), 140.7 (*C-p*), 145.3 (*C-s*), 162.0 (*C-v*); MS (ESI) 310 (M+1).

2-Isopropyl-5-methyl-4-(pyridin-4-yloxy)aniline **(55).** Obtained from **70**. Flash chromatography. (eluent: petroleum ether/EtOAc from 60 / 40 v/v to 50 / 50 v/v) to afford the title compound as a white solid. Yield 95 %. ^{1}H-NMR (600 MHz, *Chloroform-d*): δ 1.22 (*d*, 6H, J = 6.8 Hz, -CH(C*H*$_3$)$_2$), 2.01 (*s*, 3H, Ar-C*H*$_3$), 2.86 (*hept*, 1H, J = 6.8 Hz, -C*H*(CH$_3$)$_2$), 3.53 (*broad singlet*, 2H, -N*H*$_2$), 6.58

(s, 1H, *H-q*), 6.73 (*d*, 2H, J = 5.9 Hz, *aromatic protons*), 6.78 (*s*, 1H, *H-t*), 8.41 (*d*, 2H, J = 4.4 Hz, *aromatic protons*). ^{13}C-NMR (151 MHz, *Chloroform-d*): δ 15.7 (Ar-*C*H_3), 22.4 (-CH(*C*H_3)$_2$)), 27.8 (-*C*H(CH_3)$_2$), 111.2 (*C-q*), 118.4 (*C-t*), 118.7 (*aromatic carbons*), 128.5 (*C-u*), 132.4 (*C-r*), 141.2 (*C-p*)*, 144.2 (*C-s*)*, 151.1 (*aromatic carbons*), 165.8 (*C-v*); MS (ESI) 242 (M+1).

Synthetic methodologies for the preparation of the anilines **49** – **53.**

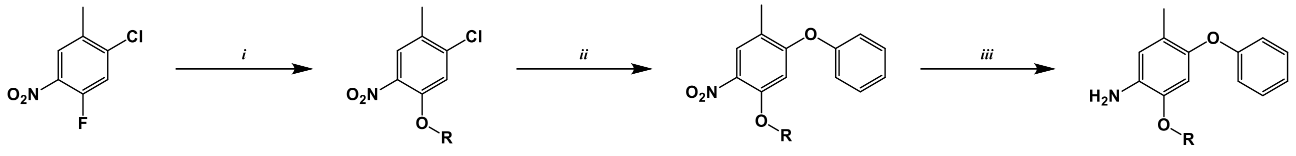

Scheme S4. Synthetic methodologies for the preparation of the anilines **49** – **53**: *i)* corresponding alcohol, Cs_2CO_3, 60 °C; *ii)* phenol, NaH, DMF, 120°C; *iii)* $SnCl_2$, 37% w/w HCl, dioxane, room temperature.

General procedure for synthesis of compounds **72** – **76**: Cs_2CO_3 (5.0 eq.) was added to a solution of 2-chloro-4-fluoro-5-nitrotoluene (**71**, 0.5 g, 2.64 mmol, 1.0 eq.) in 5 mL of the corresponding alcohol. The mixture was stirred at 60 °C for 2 hours, cooled to room temperature then concentrate under reduced pressure without reaching dryness. Water was added, and the resulting solution was extracted with EtOAc (2 x 50 mL). The organic layers were combined, dried over Na_2SO_4, concentrated, and the crude product was purified by flash chromatography.

1-Chloro-5-isopropoxy-2-methyl-4-nitrobenzene **(72).** Flash chromatography (eluent: petroleum ether / EtOAc 95:5 v/v) to afford the title compound as a white solid. Yield 80 %. ^{1}H-NMR (600 MHz, *Chloroform-d*): δ 1.39 (*d*, 6H, J = 6.1 Hz, -OCH(C*H*$_3$)$_2$), 2.33 (*s*, 3H, Ar-C*H*$_3$), 4.57-4.64 (*m*, 1H, -C*H*(CH$_3$)$_2$), 7.07 (*s*, 1H, *H-t*), 7.70 (*s*, 1H, *H-q*). ^{13}C-NMR (151 MHz, *Chloroform-d*): δ 19.2 (Ar-*C*H$_3$), 21.9 (-CH(*C*H$_3$)$_2$), 73.5 (-*C*H(CH$_3$)$_2$), 117.1 (*C-t*), 127.3 (*C-q*), 128.4 (*C-r*), 139.2 (*C-p*), 139.8(*C-s*), 150.1 (*C-u*). MS (ESI) 230 (M+1).

1-Chloro-5-cyclobutoxy-2-methyl-4-nitrobenzene **(73).** Flash chromatography (eluent: petroleum ether / EtOAc 95:5 v/v) to afford the title compound as yellowish oil. Yield 80 % ^{1}H-NMR (600 MHz, *Chloroform-d*): δ 1.66-1.76 (*m*, 1H, *cyclobutoxy proton*), 1.87-1.95 (*m*, 1H, *cyclobutoxy proton*), 2.20-2.31 (*m*, 2H, *cyclobutoxy protons*), 2.33 (*s*, 3H, Ar-C*H*$_3$), 2.45-2.53 (*m*, 2H, *cyclobutoxy protons*), 4.68 - 4.74 (*m*, 1H, -C*H*(CH$_2$)$_3$), 6.90 (*s*, 1H, *H-t*), 7.75 (*s*, 1H, *H-q*). ^{13}C-NMR (151 MHz, *Chloroform-d*): δ 13.3 (Ar-*C*H$_3$), 19.2 (-OCH(CH$_2$)$_2$(*C*H$_2$)), 30.4 (-OCH(*C*H$_2$)$_2$(CH$_2$)), 73.5 (-O*C*H(CH$_2$)$_3$), 116.0 (*C-t*), 127.5 (*C-q*), 128.3 (*C-r*), 138.0 (*C-p*), 140.1 (*C-s*), 149.8 (*C-u*).

*1-(*sec-Butoxy*)-5-chloro-4-methyl-4-nitrobenzene* **(74).** Flash chromatography (eluent: petroleum ether / EtOAc 95:5 v/v) to afford the title compound as yellowish solid. Yield 91 %. ^{1}H NMR (600 MHz, *Chloroform-d*): δ 0.99 (*t*, 3H, J = 7.5 Hz, -CH(CH$_3$)CH$_2$C*H*$_3$), 1.34 (*d*, 3H, J = 6.1 Hz, -CH(C*H*$_3$)CH$_2$CH$_3$), 1.65-1.73 (*m*, 1H, -CH(CH$_3$)C*H*$_2$CH$_3$), 1.74-1.83 (*m*, 1H, -CH(CH$_3$)C*H*$_2$CH$_3$), 2.33 (*s*, 3H, Ar-C*H*$_3$), 4.35-4.42 (*m*, 1H, -C*H*(CH$_3$)CH$_2$CH$_3$), 7.05 (*s*, 1H, *H-t*), 7.70 (*s*, 1H, *H-q*). ^{13}C NMR (151 MHz, *Chloroform-d*): δ 9.7 (-CH(CH$_3$)CH$_2$*C*H$_3$), 19.1 (Ar-*C*H$_3$), 19.2 (-CH(*C*H$_3$)CH$_2$C*H*$_3$)), 29.1 (-CH(CH$_3$)*C*H$_2$CH$_3$), 78.2 (-*C*H(CH$_3$)CH$_2$CH$_3$), 116.8 (*C-t*), 127.3 (*C-q*), 128.2 (*C-r*), 139.1 (*C-p*), 139.8 (*C-s*), 150.4 (*C-u*). MS (ESI) 244 (M+1).

1-Chloro-2-methyl-4-nitro-5-(pentan-3-yloxy)benzene **(75).** Flash chromatography (eluent: petroleum ether / EtOAc 95 / 5 v/v) to afford the title compound as a yellowish solid. Yield 79 %. ^{1}H-NMR (600 MHz, *Chloroform-d*): δ 0.97 (*t*, 6H, J = 7.4 Hz, -OCH(CH_2*CH_3*)$_2$), 1.68-1.77 (*m*, 4H, -OCH(C*H_2*CH_3)$_2$), 2.33 (*s*, 3H, Ar-C*H_3*), 4.22 (*p*, 1H, J = 5.6 Hz, -OC*H*(CH_2CH_3)$_2$), 7.03 (*s*, 1H, *H-t*), 7.69 (*s*, 1H, *H-q*). ^{13}C-NMR (151 MHz, *Chloroform-d*): δ 9.5 (-OCH(CH_2*C*H_3)$_2$), 19.2 (Ar-*C*H_3), 25.9 (-OCH(*C*H_2CH_3)$_2$), 83.1 (-O*C*H(CH_2CH_3)$_2$), 116.5 (*C-t*), 127.3 (*C-q*), 128.0 (*C-r*), 139.0 (*C-p*), 139.8 (*C-s*), 150.8 (*C-u*).

1-Chloro-2-methyl-4-nitro-5-(pentan-2-yloxy)benzene **(76).** Flash chromatography (eluent: petroleum ether / EtOAc 95 / 5 v/v) to afford the title compound as a yellowish oil. Yield 91 %. ^{1}H-NMR (600 MHz, *Chloroform-d*): δ 0.93 (*t*, 3H, J = 7.4 Hz, -CH(CH_3)CH_2CH_2C*H_3*), 1.34 (*d*, 3H, J = 6.1 Hz, -CH(CH_3)$CH_2CH_2CH_3$), 1.36 - 1.53 (*m*, 2H, -CH(CH_3)CH_2C*H_2*CH_3), 1.55 - 1.64 (*m*, 1H, -CH(CH_3)C*H_2*CH_2CH_3), 1.73 - 1.81 (*m*, 1H, -CH(CH_3)C*H_2*CH_2CH_3), 2.33 (*s*, 3H, Ar-C*H_3*), 4.42-4.48 (*m*, 1H, -C*H*(CH_3)$CH_2CH_2CH_3$), 7.05 (*s*, 1H, *H-t*), 7.70 (*s*, 1H, *H-q*). ^{13}C-NMR (151 MHz, *Chloroform-d*): δ 14.1 (-CH(CH_3)CH_2CH_2*C*H_3), 18.6 (-CH(*C*H_3)$CH_2CH_2CH_3$), 19.2 (Ar-CH_3), 19.5 (-CH(CH_3)CH_2*C*H_2CH_3), 38.4 (-CH(CH_3)*C*$H_2CH_2CH_3$), 76.8 (-*C*H(CH_3)$CH_2CH_2CH_3$), 116.6 (*C-t*), 127.3 (*C-q*), 128.1 (*C-r*), 139.0 (*C-p*), 139.8 (*C-s*), 150.4 (*C-u*); MS (ESI) 258 (M+1).

General procedure for synthesis of compounds **77 – 81**. Phenol (1.40 mmol, 1.2 eq.) was added to a suspension of 10 % w/w NaH (2.32 mmol, 1.6 eq.) in dry DMF (10 mL). The mixture was stirred for 15 minutes at room temperature under a nitrogen atmosphere then, the corresponding nitro derivate (**72 –76,** 0.3g, 1.0 eq.) was added portion wise; the resulting solution was stirred for 5 hours at 120°C under the nitrogen atmosphere. The reaction was quenched with saturated aqueous ammonium chloride solution and the resulting mixture was extracted with EtOAc (3 x 50 mL). The organic layers were combined, dried over Na_2SO_4, concentrated until a crude product that was purified by flash chromatography.

1-Isopropoxy-4-methyl-2-nitro-5-phenoxybenzene **(77).** Flash chromatography (eluent: *petroleum ether* / EtOAc 95 / 5 v/v) to afford the title compound as a yellow oil. Yield 84 %. ^{1}H-NMR (600 MHz, *Chloroform-d*): δ 1.28 (*d*, 6H, J = 6.1 Hz, -OCH(C*H_3*)$_2$), 2.25 (*s*, 3H, Ar-C*H_3*), 4.35 (*hept*, 1H, J = 6.1 Hz, -C*H*(CH_3)$_2$), 6.42 (*s*, 1H, *H-t*), 7.00 (*d*, 2H, J = 7.8 Hz, *aromatic protons*), 7.19 (*t*, 1H, J = 7.4 Hz, *aromatic proton*), 7.40 (*dd*, 2H, J = 8.2, 7.7 Hz, *aromatic protons*), 7.82 (*s*, 1H, *H-q*). ^{13}C-NMR (151 MHz, *Chloroform-d*): δ 15.4 (Ar-*C*H_3), 21.8 (-OCH(*C*H_3)$_2$), 73.1 (-O*C*H(CH_3)$_2$), 105.6 (*C-t*), 119.2 (*aromatic carbons*), 121.2 (*C-r*), 124.5 (*C-q*), 128.6 (*aromatic carbon*), 130.3 (*aromatic carbons*), 135.8 (*C-p*), 151.7 (*C-u*), 155.9 (*C-v*), 160.0 (*C-s*); MS (ESI) 288 (M+1).

1-Cyclobutoxy-4-methyl-2-nitro-5-phenoxybenzene **(78).** Flash chromatography (eluent: *petroleum ether* / EtOAc 95 / 5 v/v) to afford the title compound as a yellow oil. Yield 61 %. ^{1}H-NMR (600 MHz, *Chloroform-d*): δ 1.52-1.63 (*m*, 1H, *cyclobutoxy proton*), 1.77-1.85 (*m*, 1H, *cyclobutoxy proton*), 2.12-2.25 (*m*, 4H, *cyclobutoxy protons*), 2.26 (*s*, 3H, Ar-C*H_3*), 4.43-4.49 (*m*, 1H, -OC*H*(CH_2)$_3$), 6.20 (*s*, 1H, *H-t*), 7.01 (*d*, 2H, J = 8.0 Hz, *aromatic protons*), 7.21 (*t*, 1H, J = 7.4 Hz, *aromatic protons*), 7.41 (*dd*, 2H, J = 8.4, 7.6 Hz, *aromatic protons*), 7.87 (*s*, 1H, *H-q*). ^{13}C-NMR (151 MHz, *Chloroform-d*): δ 13.2 (*cyclobutoxy carbon*), 15.4 (Ar-*C*H_3), 30.3 (*cyclobutoxy carbon*), 73.1 (*cyclobutoxy carbon*), 103.7 (*C-t*), 119.7 (*aromatic carbons*), 120.5 (*C-r*), 124.8 (*aromatic carbon*), 128.9 (*C-q*), 130.3 (*aromatic carbons*), 134.1 (*C-p*), 151.5 (*C-u*), 155.6 (*C-v*), 160.6 (*C-s*). MS (ESI) 300 (M+1).

1-(Sec-butoxy)-4-methyl-2-nitro-5-phenoxybenzene **(79).** Flash chromatography (eluent: *petroleum ether* / EtOAc 98 / 2 v/v) to afford the title compound as a yellowish solid. Yield 43 %. ^{1}H-NMR (600 MHz, *Chloroform-d*): δ 0.90 (*t*, 3H, J = 7.4, -CH(CH$_3$)CH$_2$C*H$_3$*), 1.22 (*d*, 3H, J = 6.1, -CH(C*H$_3$*)CH$_2$CH$_3$), 1.54-1.63 (*m*, 1H, -CH(CH$_3$)C*H$_2$*CH$_3$)*, 1.65-1.74 (*m*, 1H, -CH(CH$_3$)C*H$_2$*CH$_3$)*, 2.25 (*s*, 3H, Ar-C*H$_3$*), 4.09-4.15 (*m*, 1H, -C*H*(CH$_3$)CH$_2$CH$_3$), 6.39 (*s*, 1H, *H-t*), 7.01 (*d*, 2H, J = 7.8 Hz, *aromatic protons*), 7.19 (*t*, 1H, J = 7.4 Hz, *aromatic proton*), 7.40 (*dd*, 2H, J = 8.2, 7.7 Hz, *aromatic protons*), 7.82 (*s*, 1H, *H-q*). ^{13}C NMR (151 MHz, *Chloroform-d*): δ 9.6 (-CH(CH$_3$)CH$_2$*C*H$_3$), 15.4 (Ar-*C*H$_3$), 19.0 (-CH(*C*H$_3$)CH$_2$CH$_3$), 29.0 (-CH(CH$_3$)*C*H$_2$CH$_3$), 77.7 (-*C*H(CH$_3$)CH$_2$CH$_3$), 105.1 (*C-t*), 119.2 (*aromatic carbons*), 120.8 (*C-r*), 124.5 (*aromatic carbon*), 128.7 (*C-q*), 130.3 (*aromatic carbons*), 135.5 (*C-p*), 152.0 (*C-u*), 156.0 (*C-v*), 160.1 (*C-s*). MS (ESI) 302 (M+1).

1-Methyl-5-nitro-4-(pentan-3-yloxy)-2-phenoxybenzene **(80).** Flash chromatography (eluent: *petroleum ether* / EtOAc 98 / 2 v/v) to afford the title compound as a yellowish solid. Yield 63 %. ^{1}H-NMR (600 MHz, *Chloroform-d*): δ 0.85 (*t*, 6H, J = 7.4 Hz, -OCH(CH$_2$C*H$_3$*)$_2$), 1.55-1.67 (*m*, 4H, -OCH(C*H$_2$*CH$_3$)$_2$), 2.25 (*s*, 3H, Ar-C*H$_3$*), 3.91 (*p*, 1H, J = 5.8 Hz, -OC*H*(CH$_2$CH$_3$)$_2$), 6.35 (*s*, 1H, *H-t*), 7.02 (*d*, 2H, J = 8.0 Hz, *aromatic protons*), 7.20 (*t*, 1H, J = 7.4 Hz, *aromatic proton*), 7.41 (*dd*, 2H, J = 8.3, 7.6 Hz, *aromatic protons*), 7.82 (*s*, 1H, *H-q*). ^{13}C-NMR (151 MHz, *Chloroform-d*): δ 9.5 (-OCH(CH$_2$*C*H$_3$)$_2$), 15.4 (Ar-*C*H$_3$), 26.0 (-OCH(*C*H$_2$CH$_3$)$_2$), 82.7 (-O*C*H(CH$_2$CH$_3$)$_2$), 104.5 (*C-t*), 119.4 (*aromatic carbons*), 120.4 (*C-r*), 124.6 (*aromatic carbon*), 128.7 (*C-q*), 130.3 (*aromatic carbons*), 135.2 (*C-p*), 152.4 (*C-u*), 155.8 (*C-v*), 160.2 (*C-s*); MS (ESI) 316 (M+1).

1-Methyl-5-nitro-4-(pentan-2-yloxy)-2-phenoxybenzene **(81).** Flash chromatography (eluent: *petroleum ether* / EtOAc 95 / 5 v/v) to afford the title compound as a yellow oil. Yield 94 %. ^{1}H-NMR (600 MHz, *Chloroform-d*): δ 0.85 (*t*, 3H, J = 7.4 Hz, -CH(CH$_3$)CH$_2$CH$_2$C*H$_3$*), 1.22 (*d*, 3H, J = 6.0 Hz, -CH(C*H$_3$*)CH$_2$CH$_2$CH$_3$), 1.27-1.40 (*m*, 2H, *alkylic protons*), 1.45-1.55 (*m*, 1H, *alkylic proton*), 1.64-1.71 (*m*, 1H, *alkylic proton*), 2.25 (*s*, 3H, Ar-C*H$_3$*), 4.14-4.20 (*m*, 1H, -C*H*(CH$_3$)CH$_2$CH$_2$CH$_3$), 6.38 (*s*, 1H, *H-t*), 7.01 (*d*, 2H, J = 7.9 Hz, *aromatic protons*), 7.19 (*t*, 1H, J = 7.4 Hz, *aromatic proton*), 7.40 (*t*, 2H, J = 7.9 Hz, *aromatic protons*), 7.82 (*s*, 1H, *H-q*). ^{13}C-NMR (151 MHz, *Chloroform-d*): δ 14.0 (-CH(CH$_3$)CH$_2$CH$_2$*C*H$_3$), 15.4 (Ar-*C*H$_3$), 18.6 (-CH(*C*H$_3$)CH$_2$CH$_2$CH$_3$), 19.5 (-CH(CH$_3$)CH$_2$*C*H$_2$CH$_3$), 38.2 (-CH(CH$_3$)*C*H$_2$CH$_2$CH$_3$), 76.4 (-*C*H(CH$_3$)CH$_2$CH$_2$CH$_3$), 104.9 (*C-t*), 119.3 (*aromatic carbons*), 120.7 (*C-r*), 124.5 (*aromatic carbon*), 128.7 (*C-q*), 130.3 (*aromatic carbons*), 135.4 (*C-p*), 152.0 (*C-u*), 155.9 (*C-v*), 160.1 (*C-s*); MS (ESI) 316 (M+1).

General procedure for synthesis of compounds **49 - 53**. Tin chloride (5.0 eq.) was added to a solution of corresponding nitro derivatives (**77** – **81**, 1.0 eq.) and 37 % w/w hydrochloric acid (10.0 eq.) in dioxane (10 mL). The resulting mixture was stirred at room temperature overnight, then was neutralized with saturated aqueous $NaHCO_3$ solution (50 mL) and the pH was brought to 10 with 2M NaOH. The mixture was then extracted with EtOAc (3 x 50 mL). The combined organic layers were dried over Na_2SO_4, the solvent was evaporated under reduced pressure. The crude material was purified by flash chromatography.

2-Isopropoxy-5-methyl-4-phenoxyaniline (**49**). Obtained from **77**. Flash chromatography (eluent: *petroleum ether* / EtOAc 90 / 10 v/v) to afford the title compound as a yellow oil. Yield 83 %. ^{1}H-NMR (600 MHz, *Chloroform-d*): δ 1.30 (*d*, 6H, J = 6.1 Hz, -OCH(C*H*$_3$)$_2$), 2.03 (*s*, 3H, Ar-C*H*$_3$), 3.62 (br s, 1H, -N*H*$_2$), 4.39 (*hept*, 1H, J = 6.1 Hz, -C*H*(CH$_3$)$_2$), 6.51 (*s*, 1H, *H-t*), 6.59 (*s*, 1H, *H-q*), 6.83 (*d*, 2H, J = 7.8 Hz, *aromatic protons*), 6.96 (*t*, 1H, J = 7.4 Hz, *aromatic proton*), 7.25 (*t*, 2H, J = 8.0 Hz, *aromatic protons*). ^{13}C-NMR (151 MHz, *Chloroform-d*): δ 15.5 (Ar-*C*H$_3$), 22.3 (-OCH(*C*H$_3$)$_2$), 71.1 (-O*C*H(CH$_3$)$_2$), 108.1 (*C-t*), 115.7 (*aromatic carbons*), 117.4 (*C-q*), 121.4 (*C-r*), 122.8 (*aromatic carbon*), 129.6 (*aromatic carbons*), 134.2 (*C-p*), 144.1 (*C-u*)*, 144.9 (*C-s*)*, 159.3 (*C-v*); MS (ESI) 258 (M+1).

2-Cyclobutoxy-5-methyl-4-phenoxyaniline (**50**). Obtained from **78**. Flash chromatography (eluent: *petroleum ether* / EtOAc 95 / 5 v/v) to afford the title compound as a yellowish oil. Yield 93 %. ^{1}H-NMR (600 MHz, *Chloroform-d*): δ 1.55-1.66 (*m*, 1H, *cyclobutoxy proton*), 1.77-1.85 (*m*, 1H, *cyclobutoxy proton*), 2.04 (*s*, 3H, Ar-C*H*$_3$), 2.10 - 2.20 (*m*, 2H, *cyclobutoxy protons*), 2.32-2.40 (*m*, 2H, *cyclobutoxy protons*), 4.07 (br s, 1H, -NH$_2$), 4.49-4.59 (*m*, 1H, -OC*H*(CH$_2$)$_3$), 6.36 (*s*, 1H, *H-t*), 6.66 (*s*, 1H, *H-q*), 6.83 (*d*, 2H, J = 8.1 Hz, *aromatic protons*), 6.98 (*t*, 1H, J = 7.3 Hz, *aromatic proton*), 7.26 (*t*, 2H, J = 7.9 Hz, *aromatic protons*). ^{13}C-NMR (151 MHz, *Chloroform-d*): δ 13.3 (*cyclobutoxy carbon*), 15.5 (Ar-*C*H$_3$), 30.8 (*cyclobutoxy carbon*), 72.1 (*cyclobutoxy carbon*), 106.6 (*C-t*), 115.9 (*aromatic carbons*), 117.9 (*C-q*), 121.5 (*C-r*), 122.5 (*aromatic carbon*), 129.6 (*aromatic carbons*), 132.0 (*C-p*), 144.4 (*C-u*)*, 145.6 (*C-s*)*, 159.1 (*C-v*), MS (ESI) 270 (M+1).

2-(Sec-butoxy)-5-methyl-4-phenoxyaniline (**51**). Obtained from **79**. Flash chromatography (eluent: petroleum ether / EtOAc 95 / 5 v/v) to afford the title compound as a light pink oil. Yield 77 %. ^{1}H-NMR (600 MHz, *Chloroform-d*): δ 0.95 (*t*, 3H, J = 7.5, -CH(CH$_3$)CH$_2$C*H*$_3$), 1.26 (*d*, 3H, J = 6.1 Hz, -CH(C*H*$_3$)CH$_2$CH$_3$), 1.57-1.66 (*m*, 1H, -CH(CH$_3$)C*H*$_2$CH$_3$)*, 1.70-1.78 (*m*, 1H, -CH(CH$_3$)C*H*$_2$CH$_3$)*, 2.04 (*s*, 3H, Ar-C*H*$_3$), 4.05 (*v br s*, 2H, -N*H*$_2$), 4.14 - 4.21 (*m*, 1H, -C*H*(CH$_3$)CH$_2$CH$_3$), 6.51 (*s*, 1H, *H-t*), 6.66 (*s*, 1H, *H-q*), 6.85 (*d*, 2H, J = 8.1 Hz, *aromatic protons*), 6.98 (*t*, 1H, J = 7.3 Hz, *aromatic proton*), 7.27 (*t*, 2H, J = 7.9 Hz, *aromatic protons*). ^{13}C-NMR (151 MHz, *Chloroform-d*): δ 9.9 (-CH(CH$_3$)CH$_2$*C*H$_3$), 15.5 (Ar-*C*H$_3$), 19.4 (-CH(*C*H$_3$)CH$_2$CH$_3$), 29.3 (-CH(CH$_3$)*C*H$_2$CH$_3$), 76.1 (-*C*H(CH$_3$)CH$_2$CH$_3$), 107.8 (*C-t*), 115.8 (*aromatic carbons*), 121.5 (*aromatic carbon*), 122.7 (*C-r*), 129.6 (*aromatic carbons*), 133.2 (*C-p*), 144.7 (*C-u*)*, 145.4 (*C-s*)*, 159.2 (*C-v*); MS (ESI) 272 (M+1).

5-Methyl-2-(pentan-3-yloxy)-4-phenoxyaniline (**52**). Obtained from **80**. Flash chromatography (eluent: *petroleum ether* / EtOAc 95 / 5 v/v) to afford the title compound as a brown oil. Yield 89 %. ^{1}H-NMR (600 MHz, *Chloroform-d*): δ 0.92 (*t*, 6H, J = 7.4 Hz, -OCH(CH$_2$C*H*$_3$)$_2$), 1.61-1.70 (*m*, 4H, -OCH(C*H*$_2$CH$_3$)$_2$), 2.03 (*s*, 3H, Ar-C*H*$_3$), 3.55 - 4.75 (*v br s*, 2H, -N*H*$_2$), 4.00 (*p*, 1H, J = 5.7 Hz, -OC*H*(CH$_2$CH$_3$)$_2$), 6.49 (*s*, 1H, *H-t*), 6.66 (*s*, 1H, *H-q*), 6.84 (*d*, 2H, J = 8.1 Hz, *aromatic protons*), 6.97 (*t*, 1H, J = 7.3 Hz, *aromatic proton*), 7.23 – 7.29 (*m*, 2H, *aromatic protons*). ^{13}C-NMR (151 MHz, *Chloroform-d*): δ 9.7 (-OCH(CH$_2$*C*H$_3$)$_2$), 15.5 (Ar-*C*H$_3$), 26.1 (-OCH(*C*H$_2$CH$_3$)$_2$), 81.1 (-O*C*H(CH$_2$CH$_3$)$_2$), 107.6 (*C-t*), 115.8 (*aromatic carbons*), 118.0 (*C-q*), 121.5 (*aromatic carbon*), 122.5 (*C-r*), 129.6 (*aromatic carbons*), 132.9 (*C-p*), 145.1 (*C-u*)*, 145.5 (*C-s*)*, 159.2 (*C-v*); MS (ESI) 286 (M+1).

5-Methyl-2-(pentan-2-yloxy)-4-phenoxyaniline (**53**). Obtained from **81**. Flash chromatography (eluent: *petroleum ether* / EtOAc 95 / 5 v/v) to afford the title compound as a brown oil. Yield 84 %. ^{1}H-NMR (600 MHz, *Chloroform-d*): δ 0.90 (*t*, 3H, J = 7.3 Hz, -CH(CH_3)$CH_2CH_2C\mathit{H}_3$), 1.26 (*d*, 3H, J = 6.1 Hz, -CH($C\mathit{H}_3$)$CH_2CH_2CH_3$), 1.34 - 1.48 (*m*, 2H, *alkylic protons*), 1.50 - 1.57 (*m*, 1H, *alkylic proton*), 1.69-1.76 (*m*, 1H, *alkylic proton*), 2.04 (*s*, 3H, Ar-$C\mathit{H}_3$), 3.63 - 4.58 (*v br s*, 2H, -NH_2), 4.20 - 4.27 (*m*, 1H, -C*H*(CH_3)$CH_2CH_2CH_3$), 6.51 (*s*, 1H, *H-t*), 6.69 (*s*, 1H, *H-q*), 6.85 (*d*, 2H, J = 8.1 Hz, *aromatic protons*), 6.98 (*t*, 1H, J = 7.2 Hz, *aromatic proton*), 7.27 (*m*, 2H, *aromatic protons*). ^{13}C NMR (151 MHz, *Chloroform-d*): δ 14.2 (-CH(CH_3)$CH_2CH_2\mathit{C}H_3$), 15.5 (Ar-$\mathit{C}H_3$), 18.8 (-CH($\mathit{C}H_3$)$CH_2CH_2CH_3$), 19.8 (-CH(CH_3)$CH_2\mathit{C}H_2CH_3$), 38.7 (-CH(CH_3)$\mathit{C}H_2CH_2CH_3$), 74.7 (-*C*H(CH_3)$CH_2CH_2CH_3$), 107.7 (*C-t*), 115.8 (*aromatic carbons*), 118.1 (*C-q*), 121.5 (*aromatic carbon*), 122.6 (*C-r*), 129.6 (*aromatic carbons*), 132.6 (*C-p*), 132.6 (*C-p*), 144.8 (*C-u*)*, 145.7 (*C-s*)*, 159.1 (*C-v*); MS (ESI) 286 (M+1).

Synthetic methodologies for the preparation of the anilines **47** *and* **48**

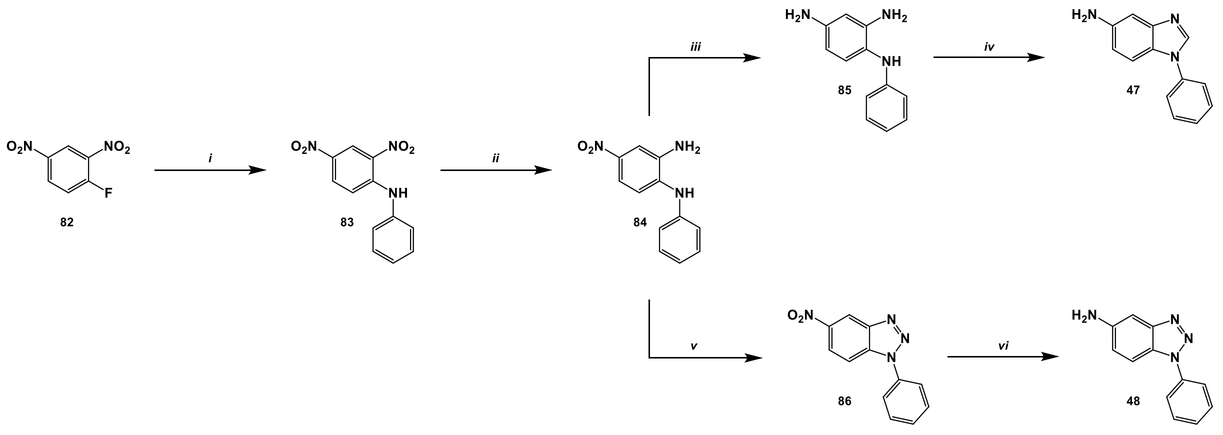

Scheme S5. Synthetic methodologies for the preparation of the anilines **47** and **48**: *i*) aniline, N,N-diisopropylethylamine, dry THF, room temperature; *ii*) $NaHCO_3$, Na_2S, MeOH, H_2O, reflux; *iii*) Pd/C, H_2, dry THF, room temperature; *iv*) formic acid, 4M HCl, reflux, *v*) isoamyl nitrite, chloroform, room temperature; *vi*) $SnCl_2$, 37 % w/w HCl, dioxane, room temperature.

2,4-Dinitro-N-phenylaniline (**83**). Aniline (0.215 µL, 2.36 mmol) and dry N,N-diisopropylethylamine (0.412 µL, 2.36 mmol) were added to a solution of 2,4-dinitrofluorobenzene (**82**, 0.400 g, 2.15 mmol) in dry THF, and stirred at r.t. overnight. The solvent was evaporated under reduced pressure and the crude material was take-up with DCM (50 mL), the resulting solution washed with 2M HCl (2 X 30 mL) and brine. The organic layer was dried over Na_2SO_4 and the solvent was evaporated under reduced pressure to give a crude solid that was recrystallized from ethanol to afford a yellow solid. Yield 97 %. ^{1}H_NMR (600 MHz, *Chloroform-d*): δ 7.17 (*d*, 1H, J = 9.5 Hz, *aromatic proton*), 7.31 (*d*, 2H, J = 7.8 Hz, *aromatic protons*), 7.39 (*t*, 1H, J = 7.5 Hz, *aromatic proton*), 7.31 (*t*, 2H, J = 7.8 Hz, *aromatic protons*), 8.17 (*dd*, 1H, J = 9.5, 2.6 Hz, *aromatic proton*), 9.18 (*d*, 1H, J = 2.6 Hz, *aromatic proton*), 9.99, (*s*, 1H.-N*H*). ^{13}C-NMR (151 MHz, *Chloroform-d*): δ 116.2, 124.2, 125.7, 127.9, 130.1, 130.4, 131.2, 136.8, 137.5, 147.3; MS (ESI) 260 (M+1)

4-Nitro-N^{1}-phenylbenzene-1,2-diamine (**84**). A solution of $NaHCO_3$ (0.525 g, 6.25 mmol) in MeOH was added to a suspension of **83** in MeOH (40 mL), the resulting mixture was stirred at room temperature for 5 minutes. In the following, a solution of Na_2S (2.92 g, 12.2mmol) in water (3.2 mL) was added dropwise over 5 minutes to the mixture, that was then stirred at reflux for 1 hour. After allowing the solution to cool to room temperature, the reaction mixture was quenched in 60 mL of water, the mixture stirred for 3 h until a red precipitate was formed. The suspension was filtered obtaining the titled compound as red solid. Yield 75 %. ^{1}H-NMR (600 MHz, *Chloroform-d*): δ 3.71 (*s*, 2H, -N*H*$_2$), 5.80 (s, 1H, -NH), 7.05 – 7.10 (m, 3H, aromatic protons), 7.15 (d, 1H, J = 8.4 Hz, *aromatic protons*), 7.35 (t, 2H, J = 7.9 Hz, aromatic protons), 7.68 – 7.72 (m, 2H, *aromatic protons*). ^{13}C-NMR (151 MHz, *Chloroform-d*): δ 112.4, 115.2, 117.3, 120.0, 123.2, 129.8, 136.2, 139.3, 141.1, 141.7; MS (ESI) 230 (M+1)

N^1-Phenylbenzene-1,2,4-triamine (**85**). Palladium on carbon (Pd/C, 40 mg, 10 % w/w) was added to a solution of **84** (0.400 g, 1.54 mmol) in dry THF (15 mL). The resulting mixture was vigorously stirred under a hydrogen atmosphere overnight. The suspension was filtered through Celite, and the cake was washed with MeOH. The filtrate was concentrated under reduced pressure. The obtained solid was further purified by flash chromatography (eluent: DCM / MeOH 9 / 1 v/v) to obtain a red solid. Yield 91 %. ^{1}H-NMR (600 MHz, *Chloroform-d*): δ 3.68 (*br s*, 4H, -2 x -NH_2), 4.97 (*s*, 1H, -N*H*), 6.11 (*dd*, 1H, J = 8.2, 2.5 Hz, *aromatic proton*), 6.15 (*d*, 1H, J = 2.5 Hz, *aromatic proton*), 6.62 (*d*, 2H, J = 7.7 Hz, *aromatic protons*), 6.75 (*t*, 1H, J = 7.3 Hz, *aromatic proton*), 6.88 (*d*, 1H, J = 8.2 Hz, *aromatic proton*), 7.17 (*dd*, 2H, J = 8.4, 7.5 Hz, *aromatic protons*). ^{13}C-NMR (151 MHz, *Chloroform-d*): δ 102.3, 106.2, 113.9, 118.3, 119.1, 129.2, 129.4, 145.0, 145.8, 147.4; MS (ESI) 200 (M+1)

5-Nitro-1-phenyl-1H-benzo[d][1,2,3]triazole (**86**). Isoamyl nitrite (2.05 mL, 15.3 mmol) was added to a solution of **84** (0.350 g, 1.53 mmol) in chloroform (20 mL); the reaction mixture was stirred at room temperature for 1 h. The solution was concentrated to afford the title compound as yellow solid and used without further purification (m.p. 169.1 – 171.1 °C). Yield 70 %. ^{1}H-NMR (600 MHz, *Chloroform-d*): δ 7.60 (*t*, 1H, J = 7.4 Hz, *aromatic proton*), 7.68 (*t*, 2H, J = 7.8 Hz, *aromatic protons*), 7.77 (*d*, 2H, J = 7.7 Hz, *aromatic protons*), 7.85 (*d*, 2H, J = 9.1 Hz, *aromatic protons*), 8.46 (*dd*, 1H, J = 9.1, 1.9 Hz, *aromatic proton*), 9.09 (*d*, 1H, J = 1.7 Hz, *aromatic proton*). ^{13}C-NMR (151 MHz, *Chloroform-d*): δ 111.1, 117.7, 123.3, 123.4, 129.8, 130.4, 135.1, 136.1, 145.1, 145.8.; MS (ESI) 241 (M+1)

1-Phenyl-1H-benzo[d]imidazol-5-amine (**47**). Formic acid (0.146 mL, 3.86 mmol) was added to a solution of **85** (0.220 g, 1.1 mmol) in 4M HCl (10 mL); the resulting reaction mixture was heated under reflux for 4h then cooled to 0 °C and treated with 6M NaOH until a pH 11 was reached. The mixture was extracted with EtOAc (3 x 75 mL), the combined organic layers were dried over Na_2SO_4 and the solvent was evaporated under reduced pressure. The crude material was purified by flash chromatography (eluent: DCM / MeOH 95 / 5 v/v), to afford the title compound as brown solid. Yield 82 %. ^{1}H-NMR (600 MHz, *Chloroform-d*): δ 3.45 (*br s*, 2H, -NH_2), 6.75 (*dd*, 1H, J = 8.6, 1.8 Hz, *aromatic proton*), 7.15 (*d*, 1H, J = 1.7 Hz, *aromatic proton*), 7.33 (*d*, 1H, J = 8.6 Hz, *aromatic proton*), 7.43 (*t*, 1H, J = 7.4 Hz, *aromatic protons*), 7.49 (*d*, 2H, J = 7.6 Hz, aromatic protons), 7.55 (*t*, 2H, J = 7.7 Hz, *aromatic protons*), 8.01 (*s*, 1H, *aromatic proton*). ^{13}C-NMR (151 MHz, *Chloroform-d*): δ 105.3, 111.0, 113.8, 123.8, 127.7, 127.8, 130.1, 130.7, 142.3, 142.7, 145.4; MS (ESI) 210 (M+1)

1-Phenyl-1H-benzo[d][1,2,3]triazol-5-amine (**48**). Tin chloride (1.09 g, 5.2 mmol) was added to a solution of **86** (0.233 g, 0.97 mmol) and 37 % w/w HCl (0.80 mL, 10.4 mmol) in dioxane (20 mL). The resulting mixture was stirred at room temperature overnight, neutralized with saturated aqueous $NaHCO_3$ solution (50 mL) then the pH was brought to 10 with 2M NaOH. The mixture was then extracted with EtOAc (3 x 50 mL). The combined organic layers were dried with brine and over Na_2SO_4 and the solvent was evaporated under reduced pressure. to afford the title compound as brown solid. Yield 99 %. ^{1}H-NMR (600 MHz, *Chloroform-d*): δ 3.88 (*br s*, 2H, -NH_2), 6.98 (*dd*, 1H, J = 8.8, 2.0 Hz, *aromatic proton*), 7.28 (*d*, 1H, J = 1.7 Hz, *aromatic proton*), 7.47 (*t*, 1H, J = 7.5 Hz, *aromatic protons*), 7.54 (*d*, 1H, J = 8.8 Hz, *aromatic proton*),7.59 (*t*, 2H, J = 7.9 Hz, aromatic protons), 7.76 (*d*, 2H, J = 7.6 Hz, *aromatic protons*). ^{13}C-NMR (151 MHz, *Chloroform-d*): δ 101.8, 111.1, 120.1, 122.7, 127.2, 128.5, 129.9, 137.3, 143.9, 148.2; MS (ESI) 211 (M+1)

Synthetic methodologies for the preparation of the anilines **46** *and* **47**

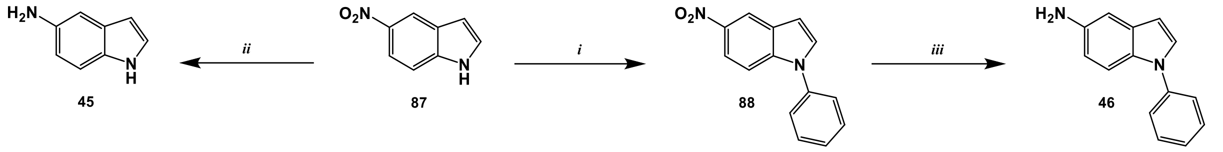

Scheme S6. Synthetic methodologies for the synthesis of the targets **45** and **48**: *i*) anhydrous $Cu(OAc)_2$, diisopropylethylamine, phenylboronic acid (0.5 eq. x 4), dry dichloromethane, room temperature; *ii*) Pd/C, H_2, dry THF, room temperature; iii) $SnCl_2$, 37% w/w HCl, dioxane, room temperature.

5-Nitro-1-phenyl-1H-indole (**88**). Anhydrous copper (II) acetate (2.80 g, 15.4 mmol) and diisopropylethylamine (2.3 mL, 15.4 mmol) were added to a solution of 5-nitroindole (**87**, 1.00 g, 6.17 mmol) in dry DCM (50 mL). The reaction mixture was stirred at 25 °C for 30 minutes, then phenylboronic acid (1.88 g, 1.54 mmol) was added to the mixture portion wise (0.5 eq. every 2 hours). In the following the reaction was stirred for 2 days at room temperature adding one additional equivalent of phenylboronic acid during the second day. The reaction mixture was quenched in 80 mL of 1M HCl and the aqueous phase was extracted with EtOAc (3 x 70 mL). The combined organic layers were dried over Na_2SO_4 and the solvent was evaporated under reduced pressure. The crude material was purified by flash chromatography (eluent: petroleum ether / EtOAc 80 / 20 v/v) to afford the title compound as yellow solid after crystallization with diisopropyl ether. Yield 62 %. ^{1}H-NMR (600 MHz, *Chloroform-d*): δ 6.86 (*d*, 1H, J = 3.2 Hz, *aromatic proton*), 7.44–7.51 (*m*, 4H, *aromatic protons*), 7.53 (*d*, 1H, J = 9.1 Hz, *aromatic proton*), 7.58 (*t*, 2H, J = 7.8 Hz, *aromatic protons*), 8.11 (*dd*, 1H, J = 9.1, 2.2 Hz, *aromatic proton*), 8.65 (*d*, 1H, J = 2.2 Hz, *aromatic proton*). ^{13}C-NMR (151 MHz, *Chloroform-d*): *δ* 105.8, 110.6, 118.1, 118.4, 124.8, 127.9, 128.6, 130.1, 131.4, 138.7, 138.8, 142.3. MS (ESI) 239 (M+1).

1H-Indol-5-amine (**45**). Palladium on carbon (Pd/C, 80 mg, 20 % w/w) was added to a solution of **87** (0.800 g, 4.93 mmol) in dry THF (25 mL). The resulting mixture was vigorously stirred under a hydrogen atmosphere at room temperature overnight. The suspension was filtered through Celite, the cake was washed with MeOH. The filtrate was concentrated under reduced pressure. The obtained solid was further purified by flash chromatography (eluent: petroleum ether / EtOAc 60 /40 v/v) to obtain a brown solid. Yield 58 %. ^{1}H-NMR (600 MHz, *Chloroform-d*): *δ* 3.51 (*br s*, 2H, -N*H₂*), 6.38 (*t*, 1H, J = 2.3 Hz, *aromatic proton*), 6.68 (*dd*, 1H, J = 8.5, 2.2 Hz, *aromatic proton*), 6.96 (*d*, 1H, J = 2.1 Hz, *aromatic proton*), 7.12 (*t*, 1H, J = 2.8 Hz, *aromatic proton*), 7.19 (*d*, 1H, J = 8.5 Hz, *aromatic proton*), 8.01 (*br s*, 1H, -N*H*). ^{13}C-NMR (151 MHz, *Chloroform-d*): *δ* 101.6, 105.7, 114.6, 113.1, 124.9, 128.9, 130.8, 139.6; MS (ESI) 133 (M+1)

1-Phenyl-1H-indol-5-amine (**46**). Tin chloride (1.893 g, 8.39 mmol) was added to a solution of **88** (0.400 g, 1.679 mmol) and 37 % w/w hydrochloric acid (1.40 mL, 16.79 mmol) in dioxane (20 mL). The resulting mixture was stirred at room temperature overnight, then was neutralized with saturated aqueous $NaHCO_3$ solution (50 mL) then the pH was brought to 10 with 2M NaOH. The mixture was then extracted with EtOAc (4 x 50 mL). The combined organic layers were dried with brine and over Na_2SO_4 and the solvent was evaporated under reduced pressure to afford the title compound as brown solid. Yield 45 %. ^{1}H-NMR (600 MHz, *Chloroform-d*): δ 3.55 (*br s*, 2H, -N*H*$_2$), 6.51 (*d*, 1H, J = 3.2, Hz, *aromatic proton*), 6.69 (*dd*, 1H, J = 8.7, 2.2 Hz, *aromatic proton*), 6.98 (*d*, 1H, J = 2.2 Hz, *aromatic proton*), 7.28 (*d*, 1H, J = 3.2 Hz, *aromatic proton*), 7.30 – 7.35 (*m*, 1H, *aromatic proton*), 7.41 (*d*, 1H, J = 8.7 Hz, *aromatic proton*), 7.47 – 7.52 (*m*, 4H, *aromatic protons*). ^{13}C-NMR (151 MHz, *Chloroform-d*): *δ* 102.7, 105.9, 111.3, 113.1, 124.0, 126.1, 128.3, 129.7, 130.5, 130.8, 140.18, 140.23; MS (ESI) 209 (M+1)

2-Benzyloxy-N-(5-phenoxypyridin-2-yl)pyrazolo[1,5-a]pyridine-3-carboxamide (**24**)

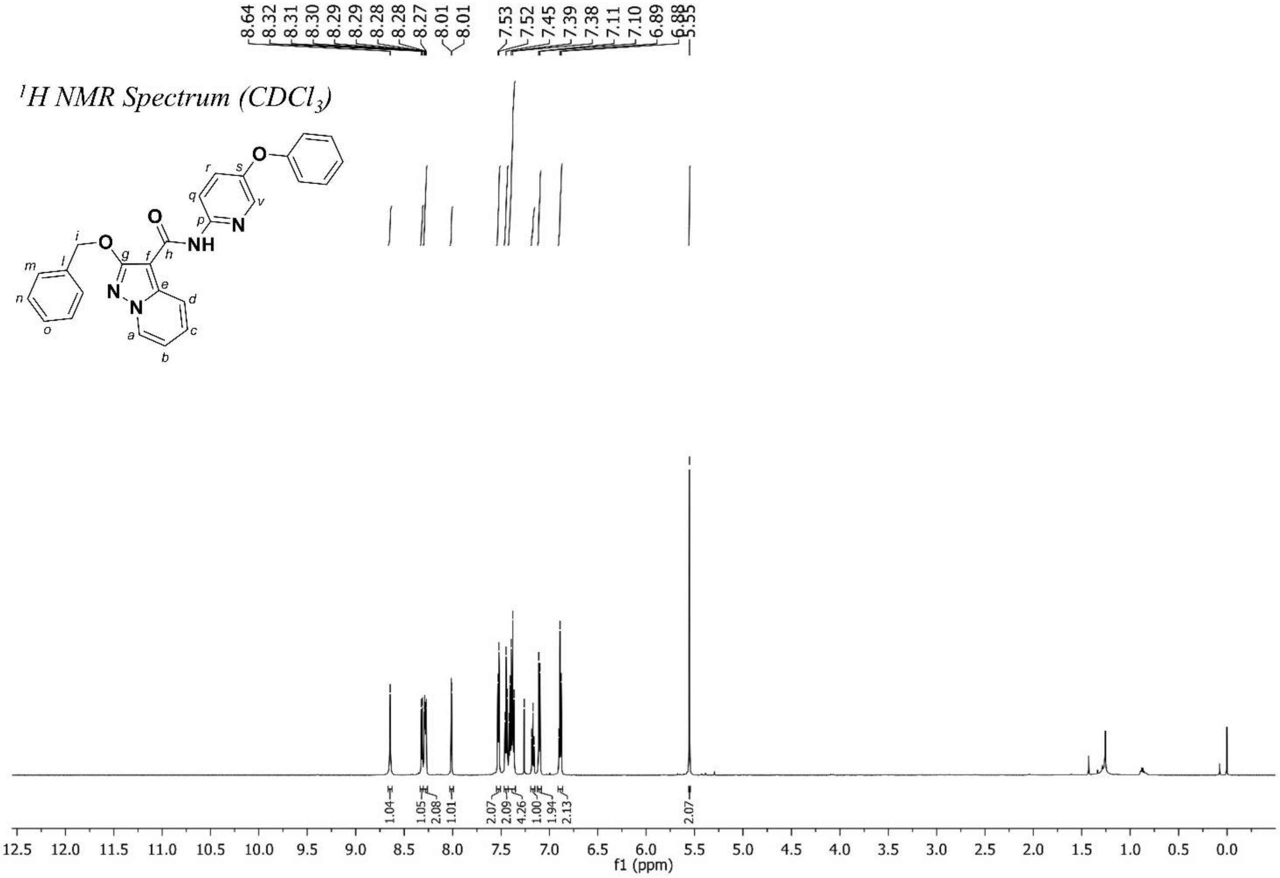

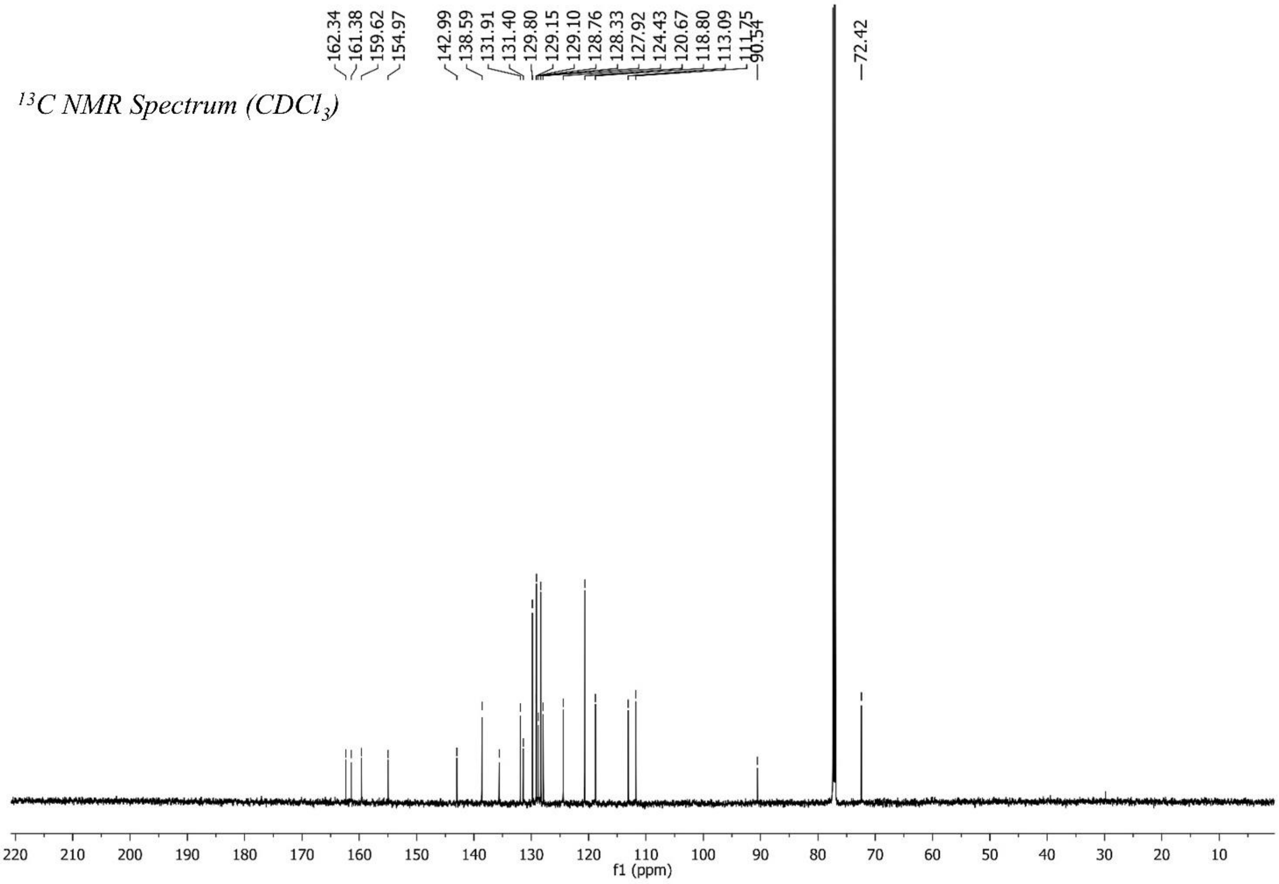

2-Benzyloxy-N-5-[3-(trifluoromethyl)phenoxy]piridin-2-ylpyrazolo[1,5-a]pyridine-3-carboxamide (**25**)

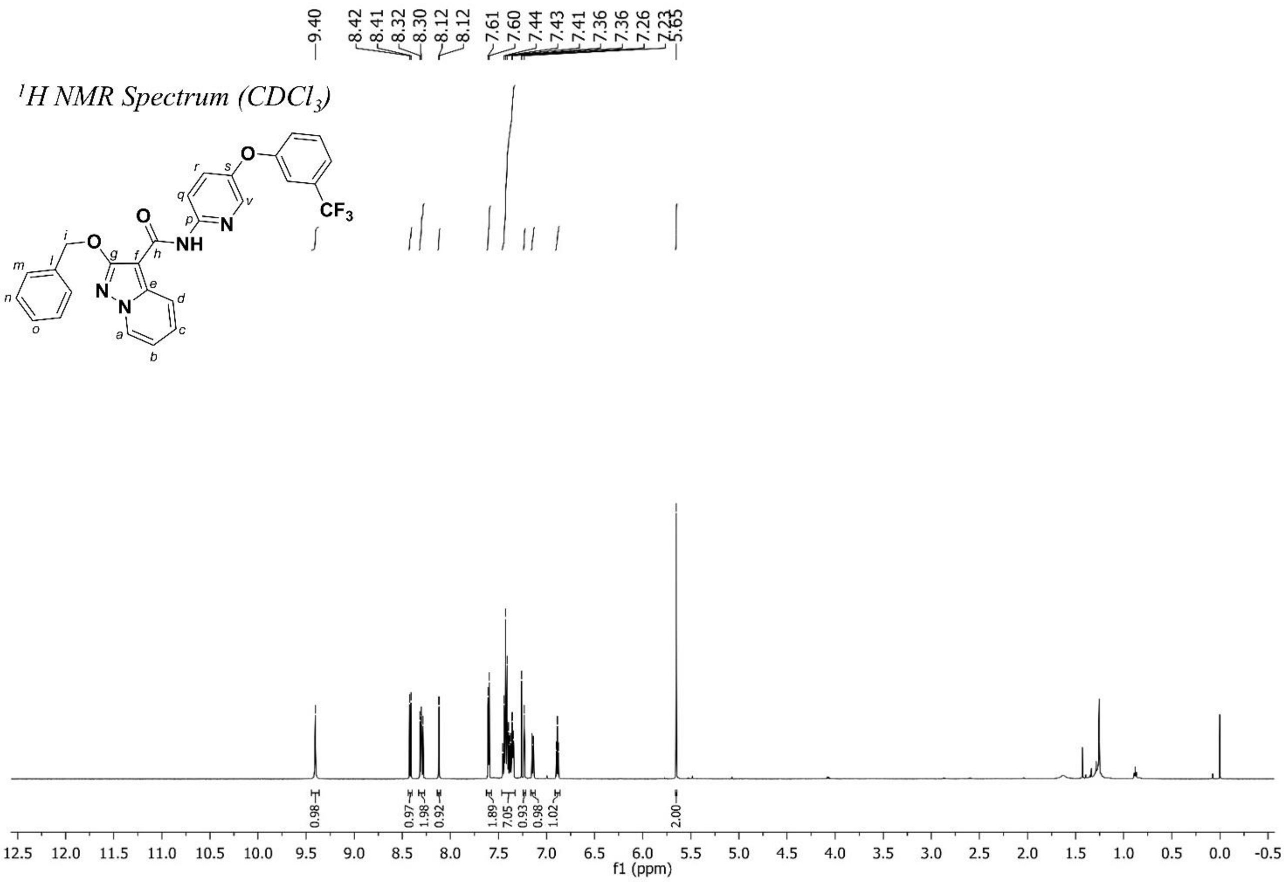

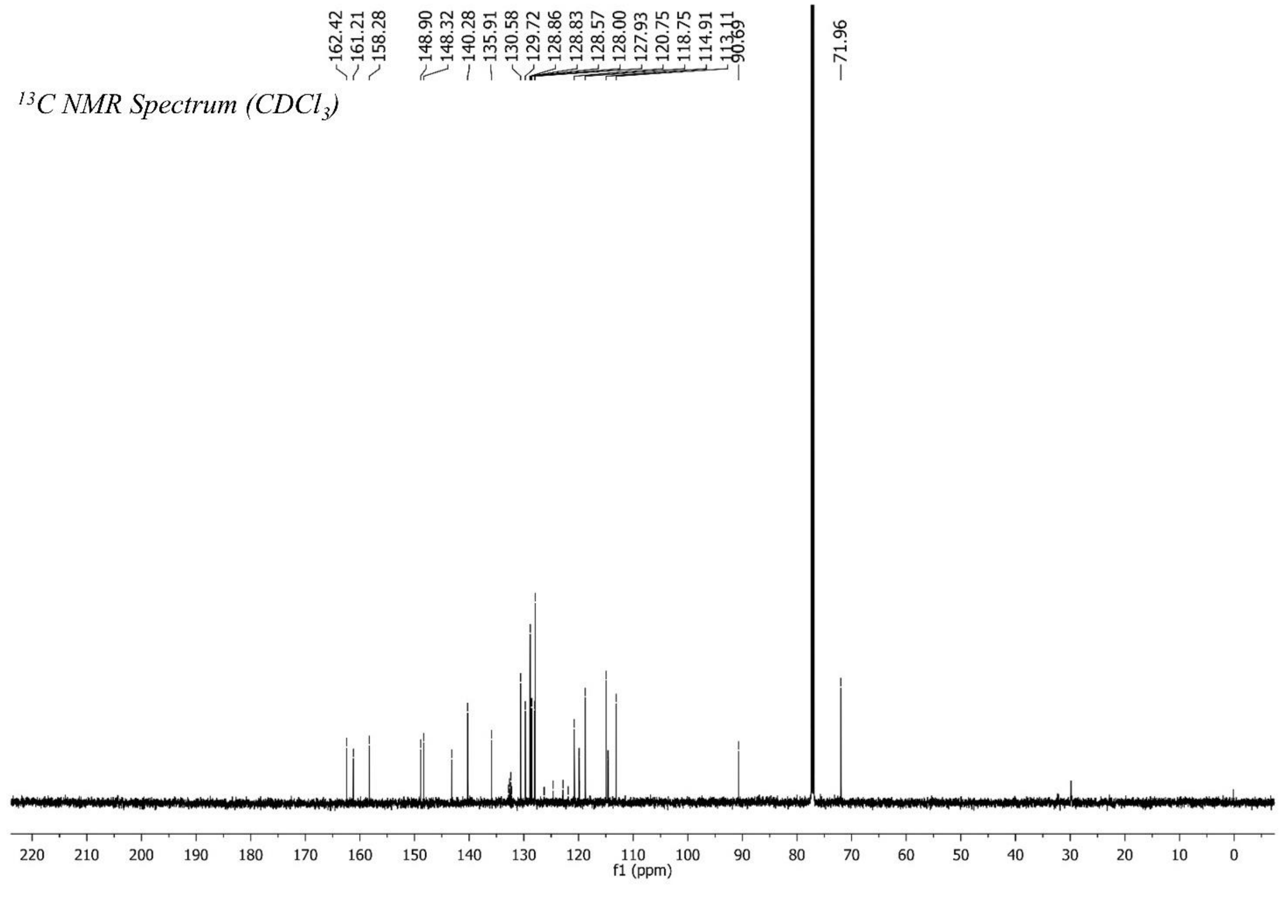

2-(Benzyloxy)-N-(2-methyl-4-phenoxyphenyl)pyrazolo[1,5-a]pyridine-3-carboxamide (**21**)

^{1}H NMR Spectrum (CDCl3)

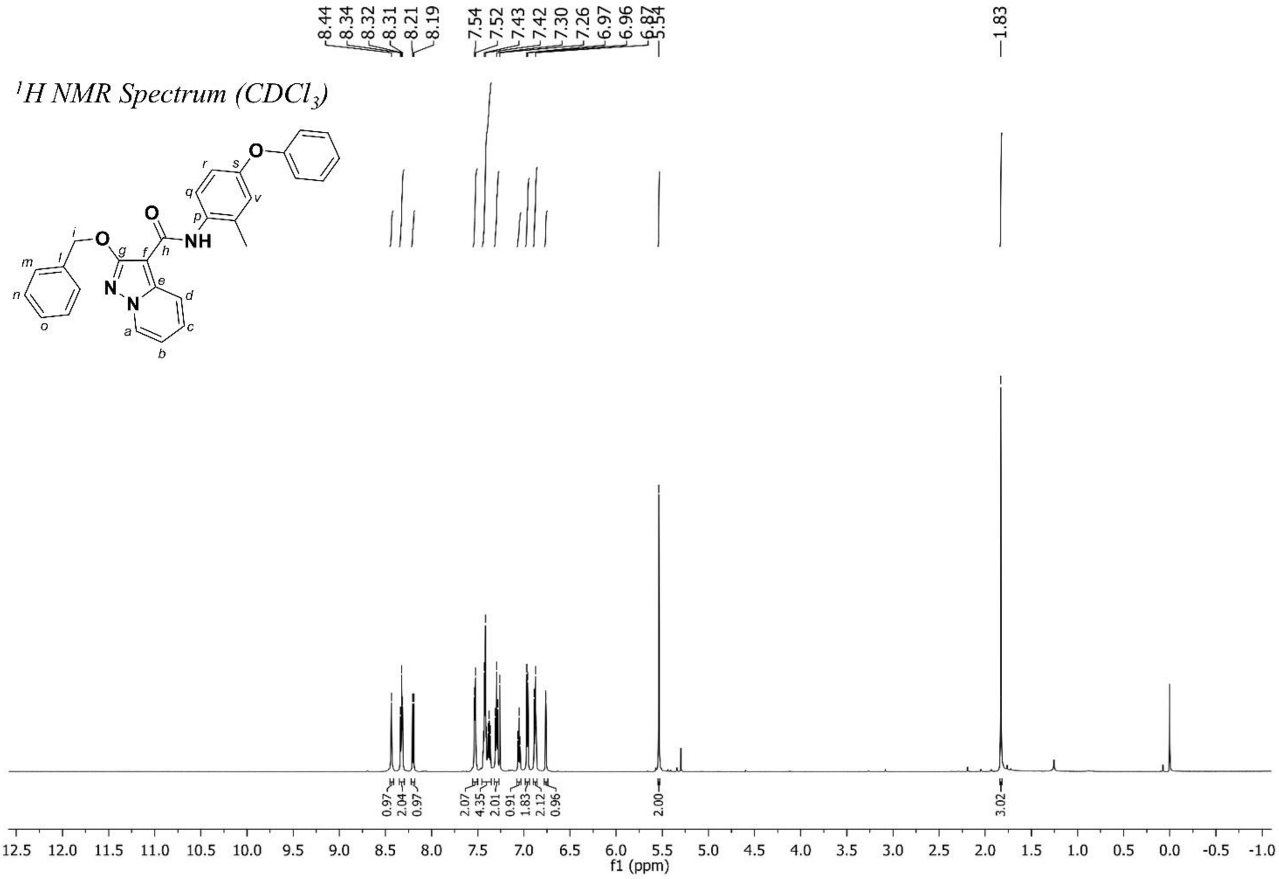

^{13}C NMR Spectrum (CDCl3)

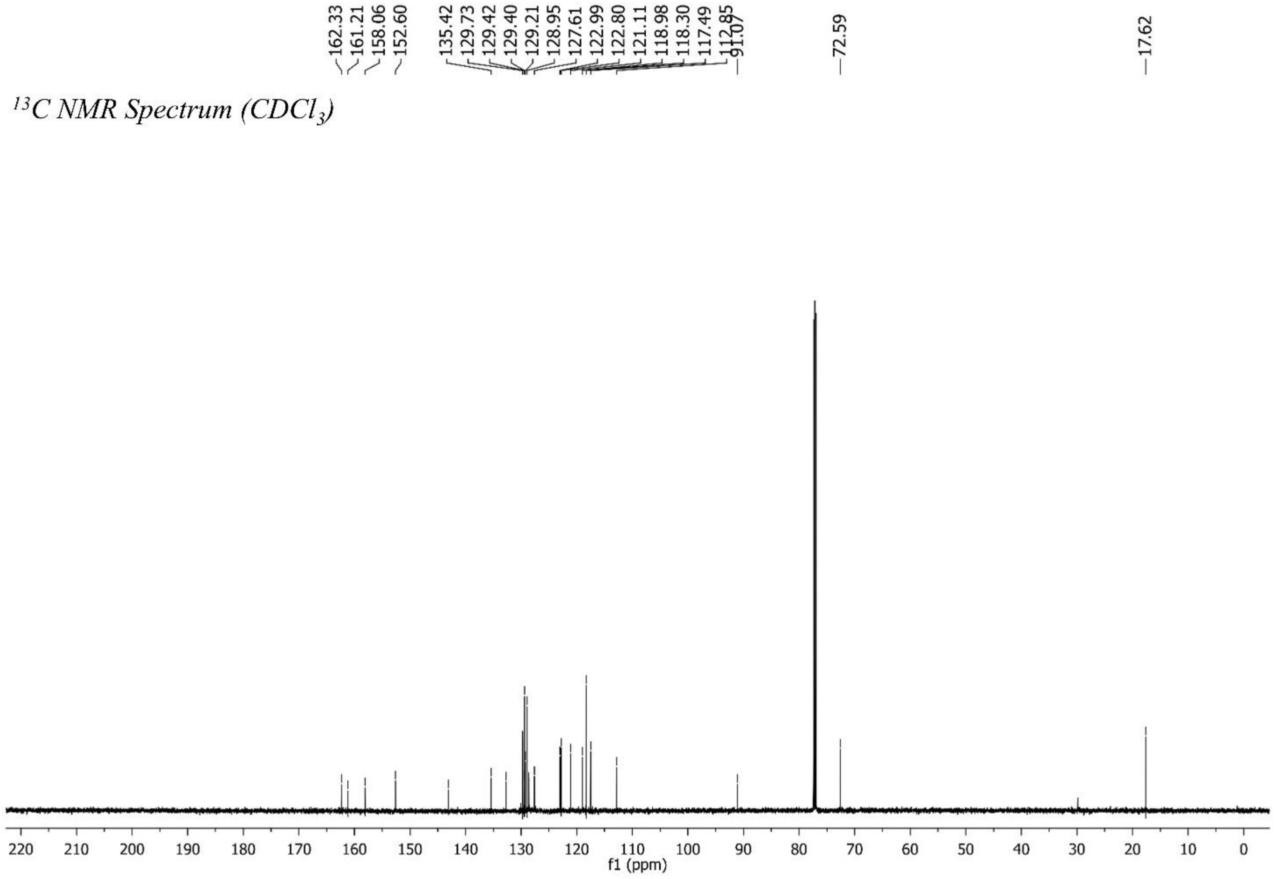

2-(Benzyloxy)-N-(2-isopropyl-5-methyl-4-phenoxyphenyl)pyrazolo[1,5-a]pyridine-3-carboxamide (**22**)

^{1}H NMR Spectrum ($CDCl_3$)

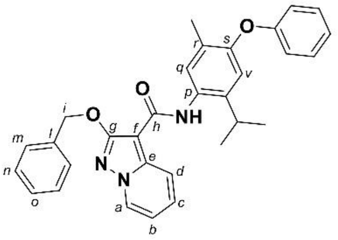

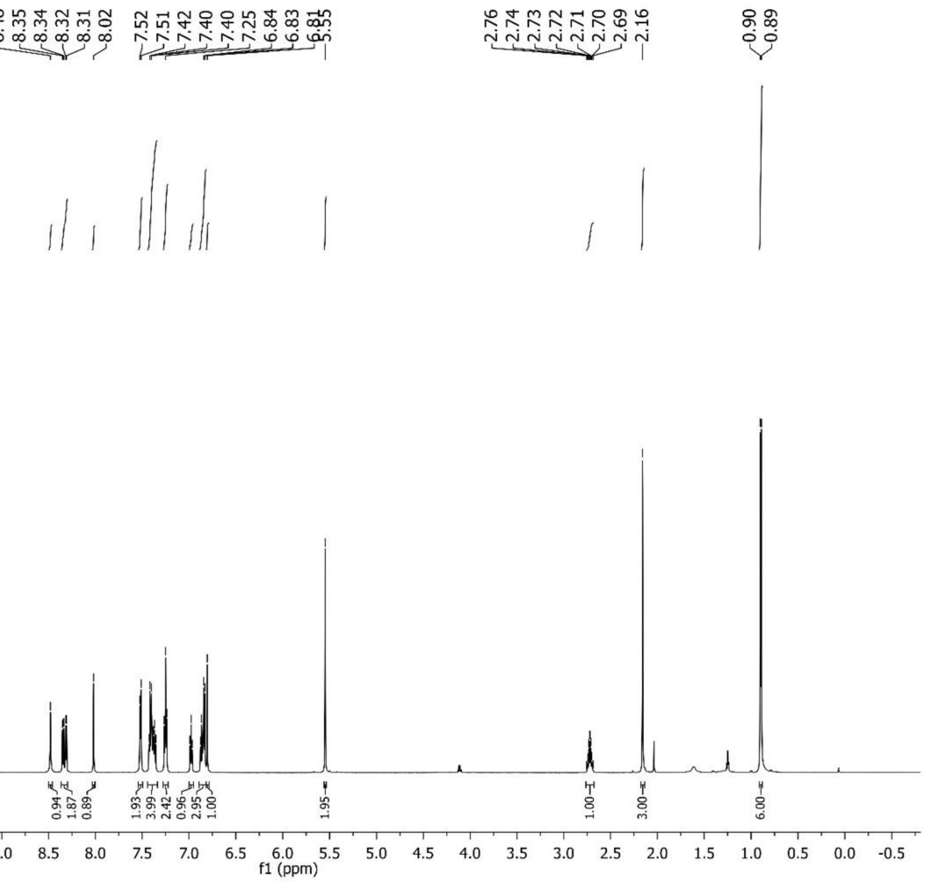

^{13}C NMR Spectrum ($CDCl_3$)

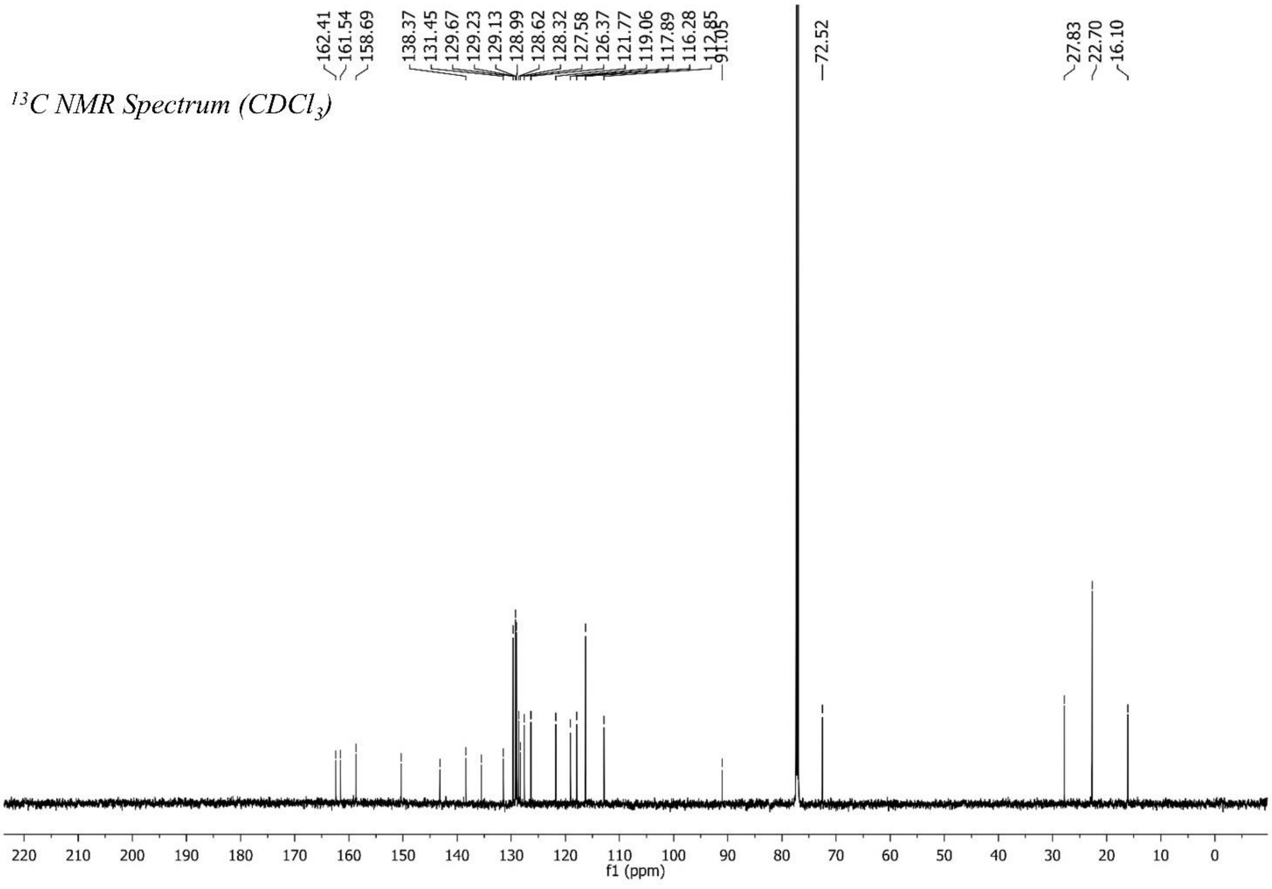

2-(Benzyloxy)-N-(5-isopropyl-2-methyl-4-phenoxyphenyl)pyrazolo[1,5-a]pyridine-3-carboxamide (**23**)

^{1}H NMR Spectrum ($CDCl_3$)

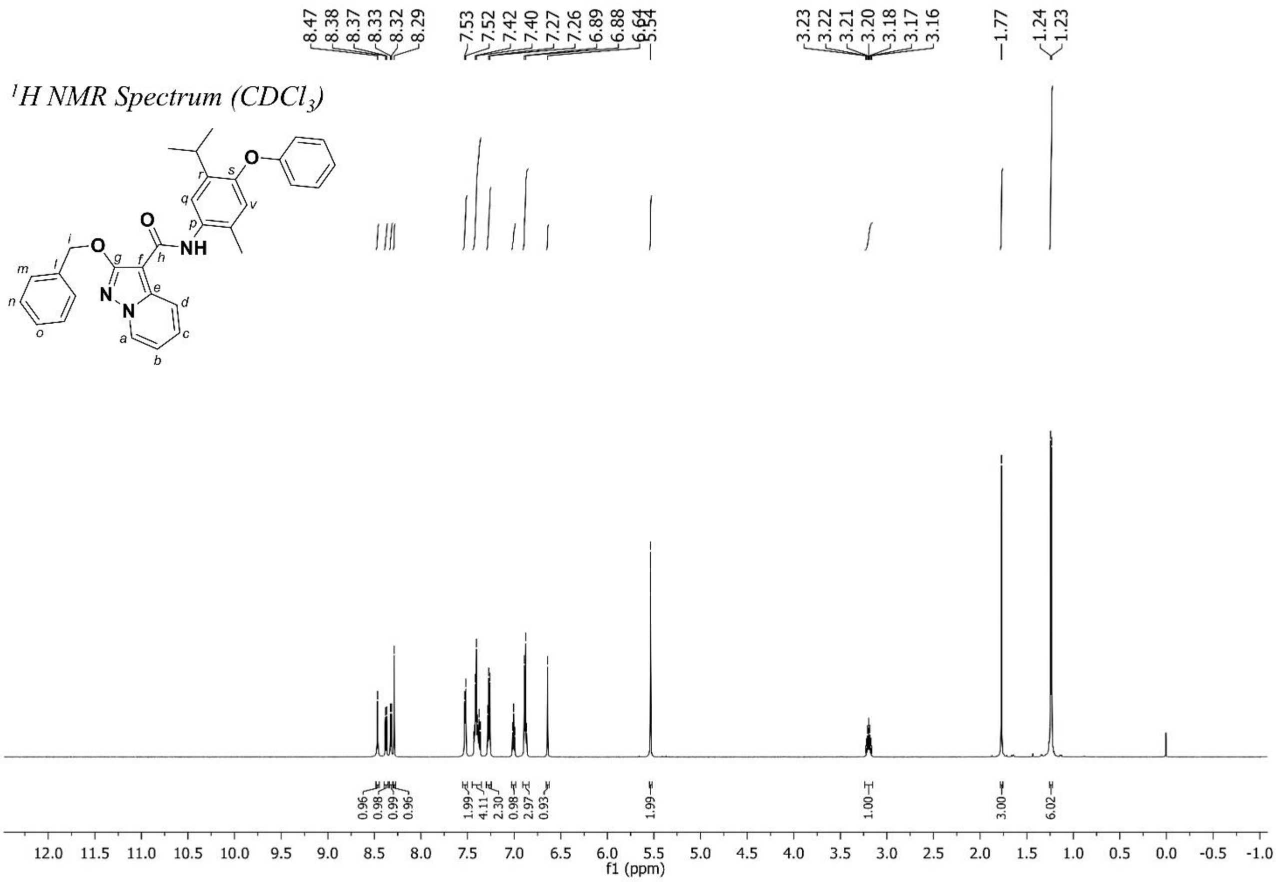

^{13}C NMR Spectrum ($CDCl_3$)

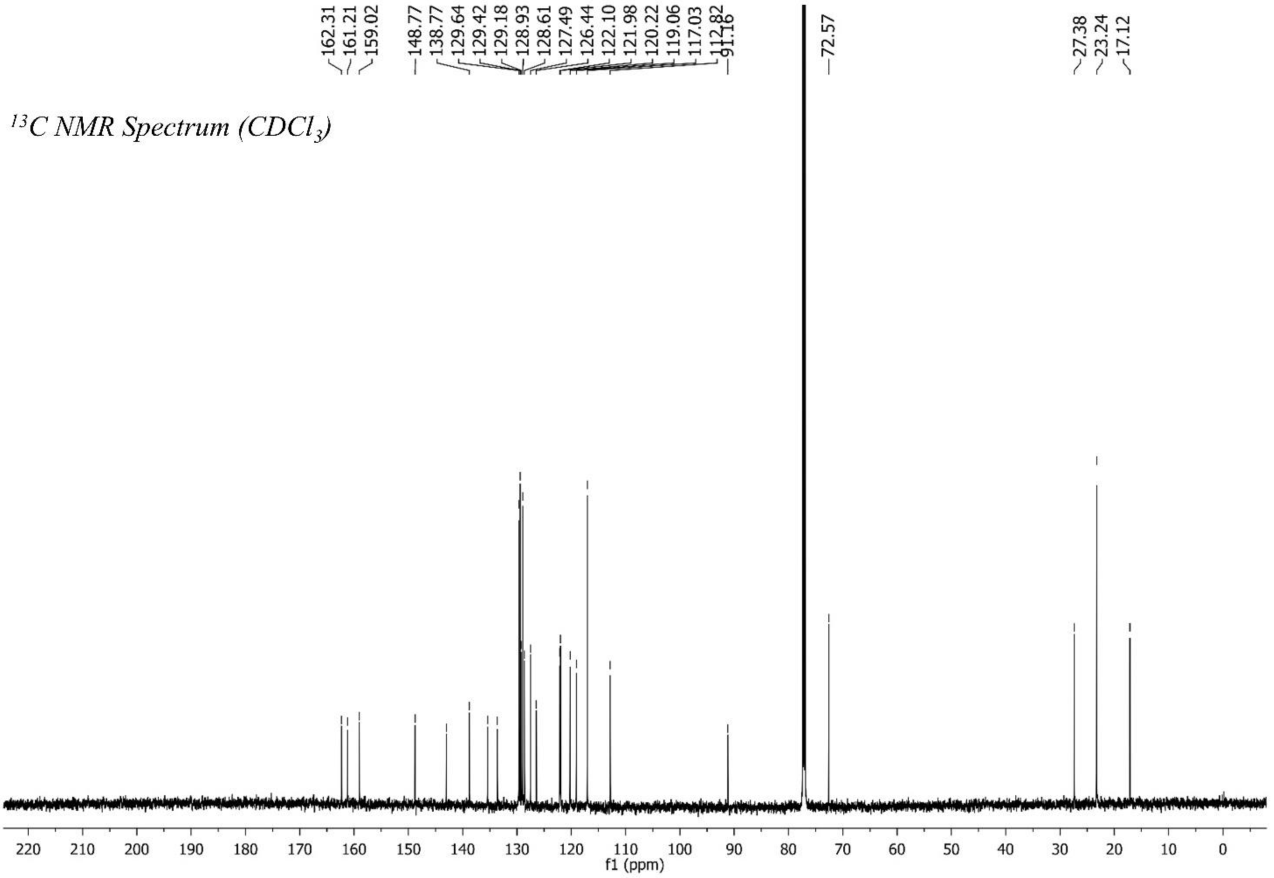

2-(Benzyloxy)-N-(2-isopropyl-5-methyl-4-(4-(trifluoromethyl)phenoxy)phenyl)pyrazolo[1,5-a]pyridine-3-carboxamide (**26**)

^{1}H NMR Spectrum ($CDCl_3$)

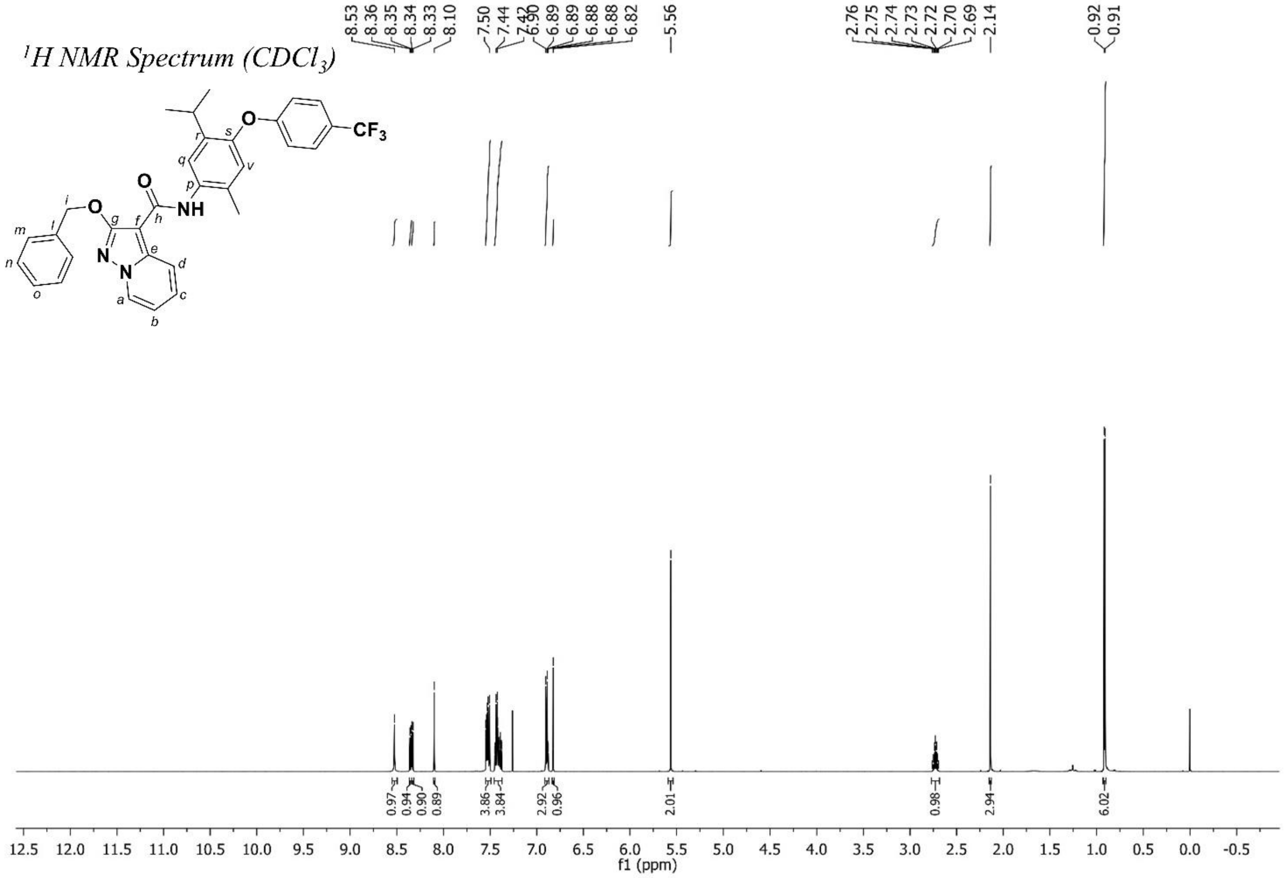

^{13}C NMR Spectrum ($CDCl_3$)

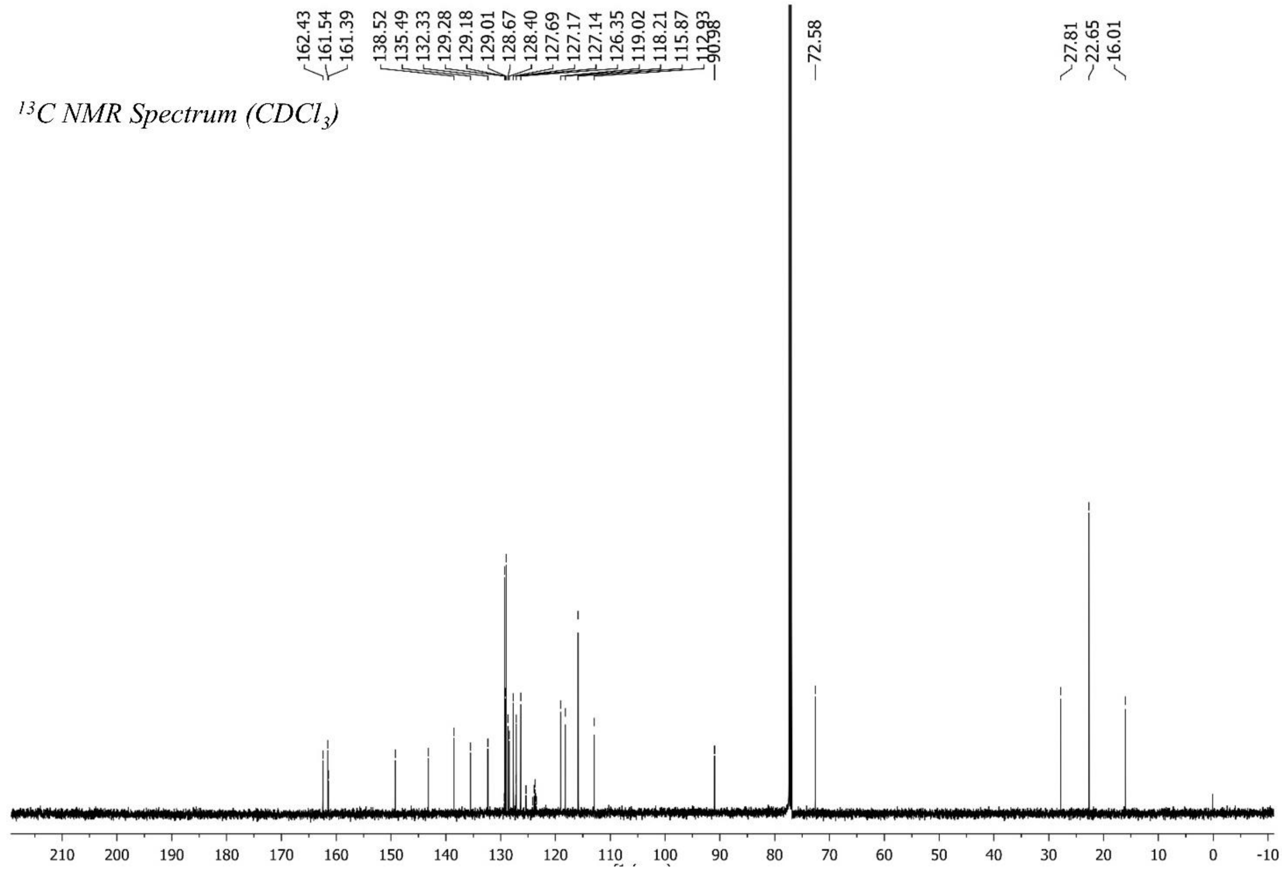

2-(Benzyloxy)-N-(1H-indol-5-yl)pyrazolo[1,5-a]pyridine-3-carboxamide (**27**)

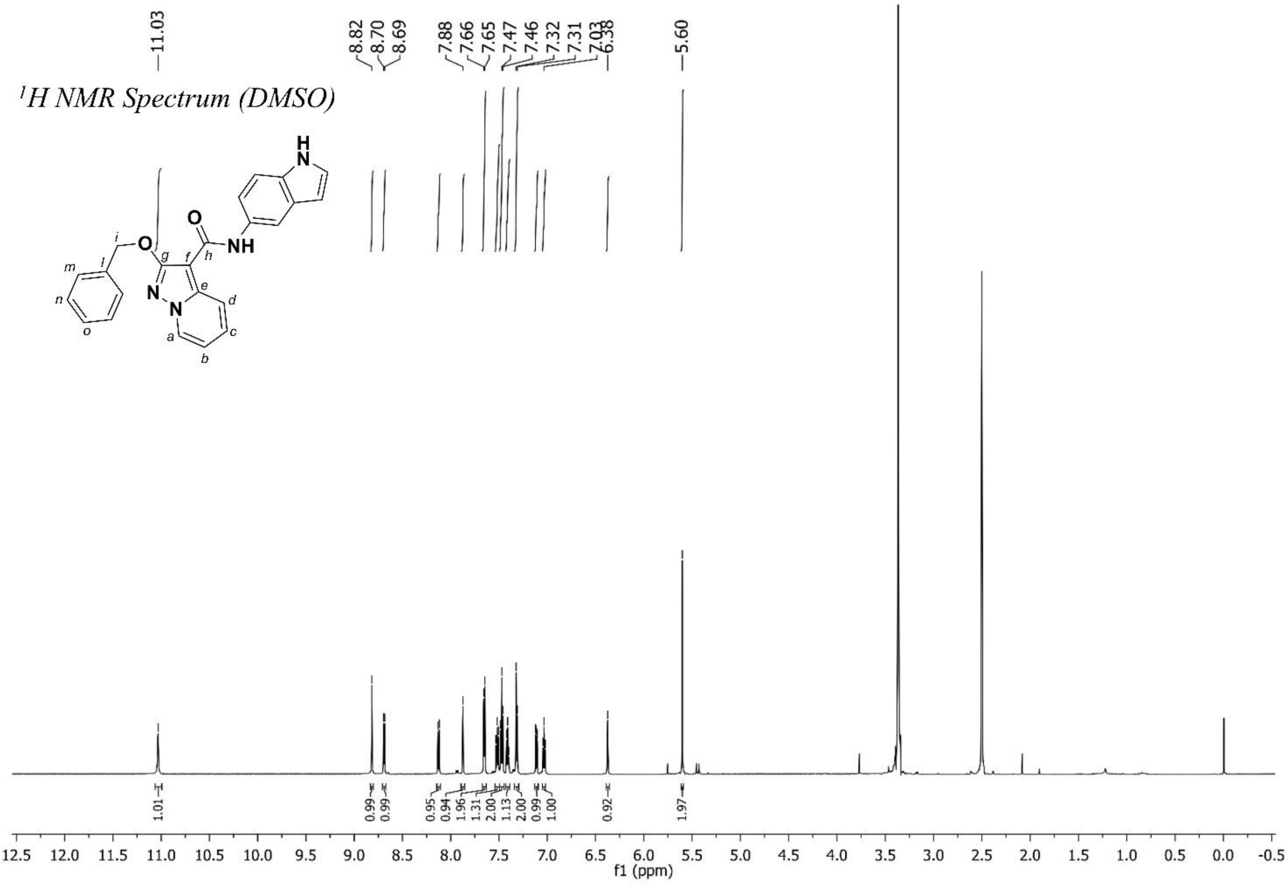

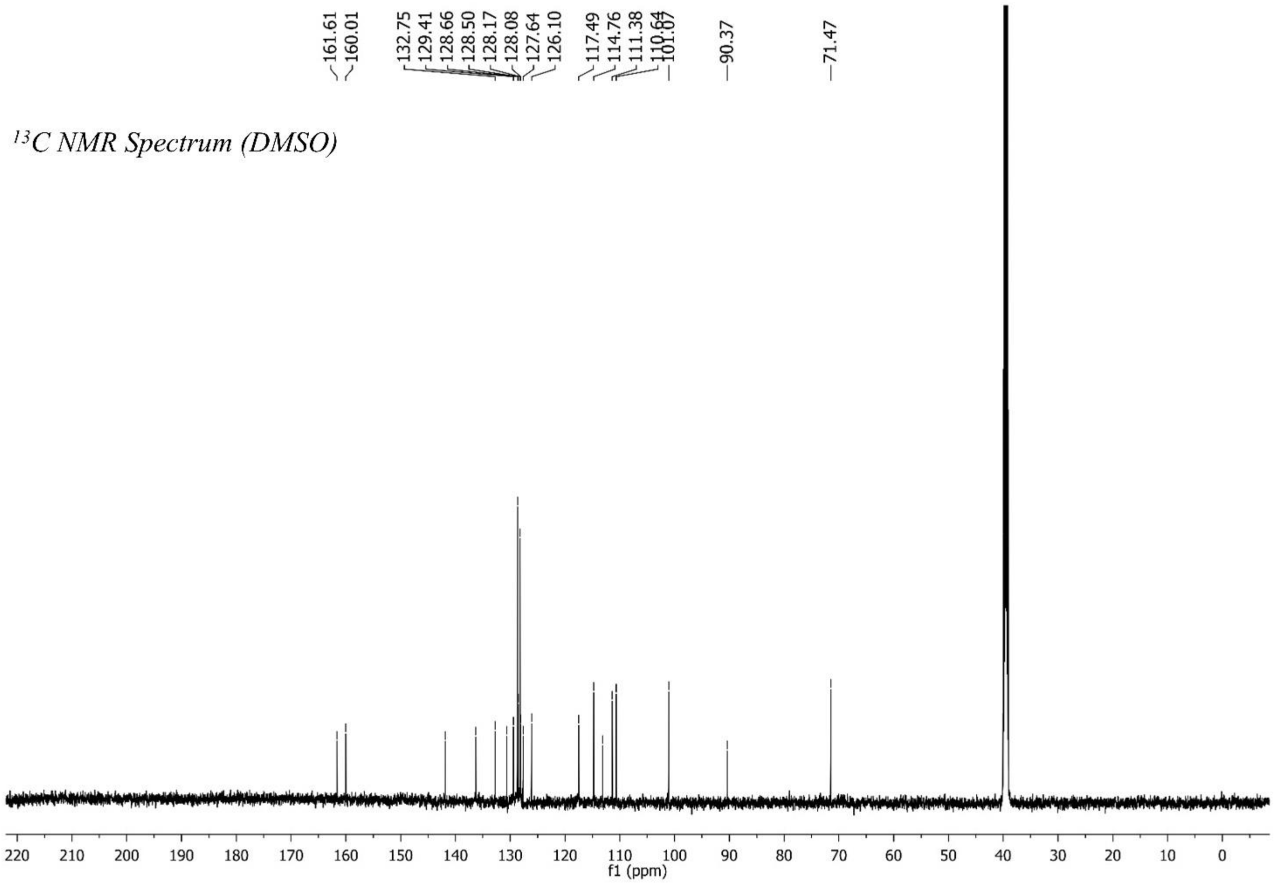

2-(Benzyloxy)-N-(1-phenyl-1H-indol-5-yl)pyrazolo[1,5-a]pyridine-3-carboxamide (**28**)

^{1}H NMR Spectrum ($CDCl_3$)

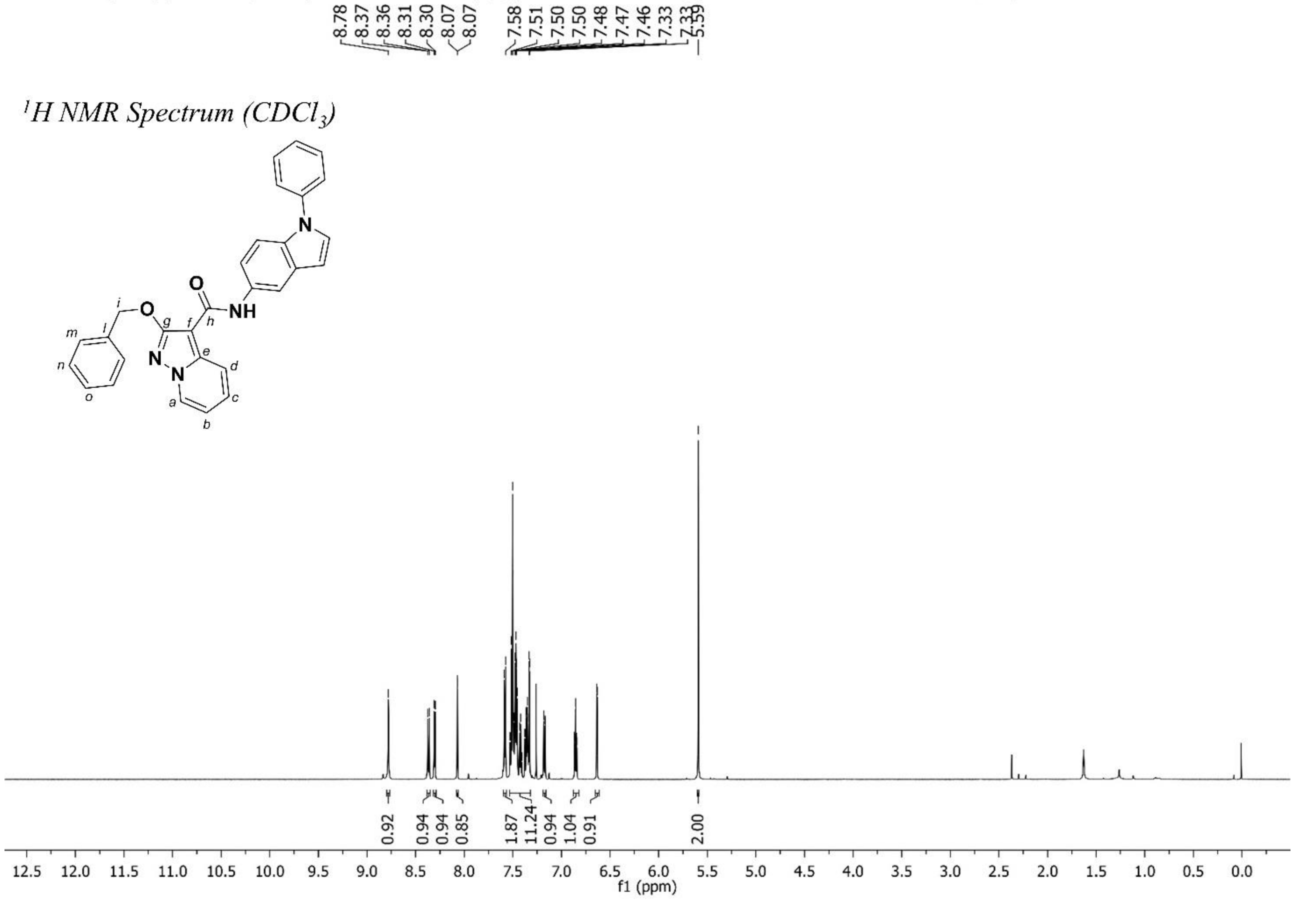

^{13}C NMR Spectrum ($CDCl_3$)

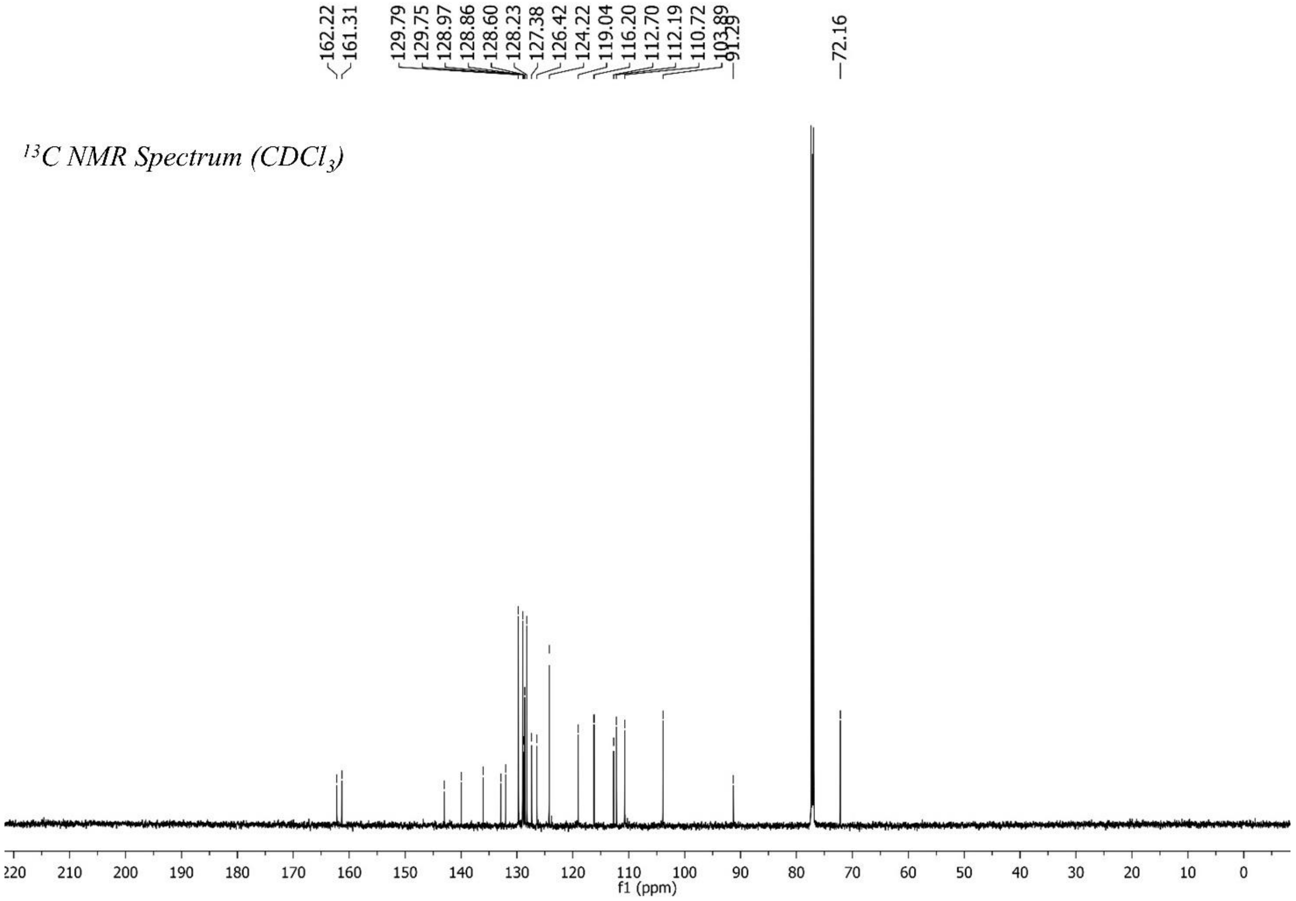

2-(Benzyloxy)-N-(1-phenyl-1H-benzo[d]imidazol-5-yl)pyrazolo[1,5-a]pyridine-3-carboxamide (**29**)

^{1}H NMR Spectrum (CDCl$_3$)

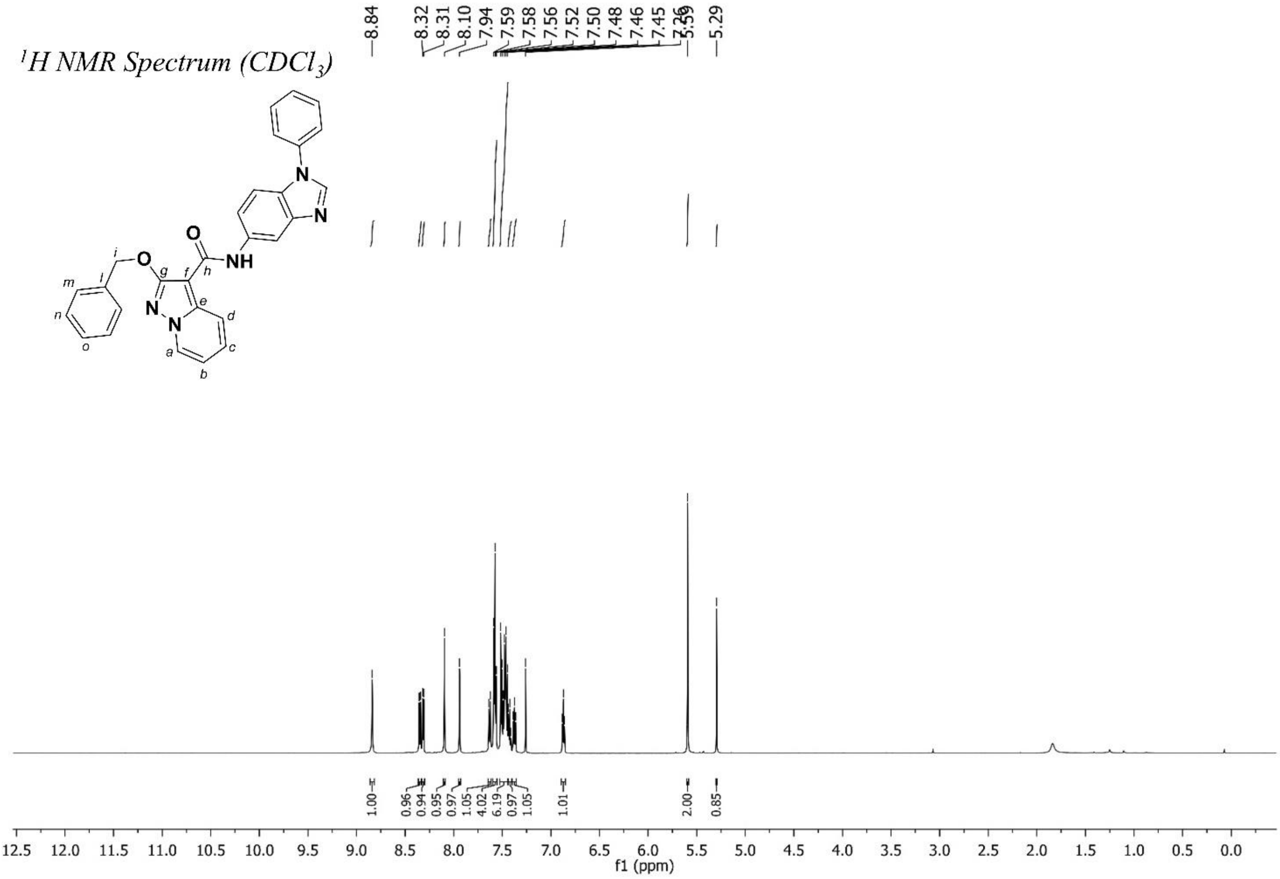

^{13}C NMR Spectrum (CDCl$_3$)

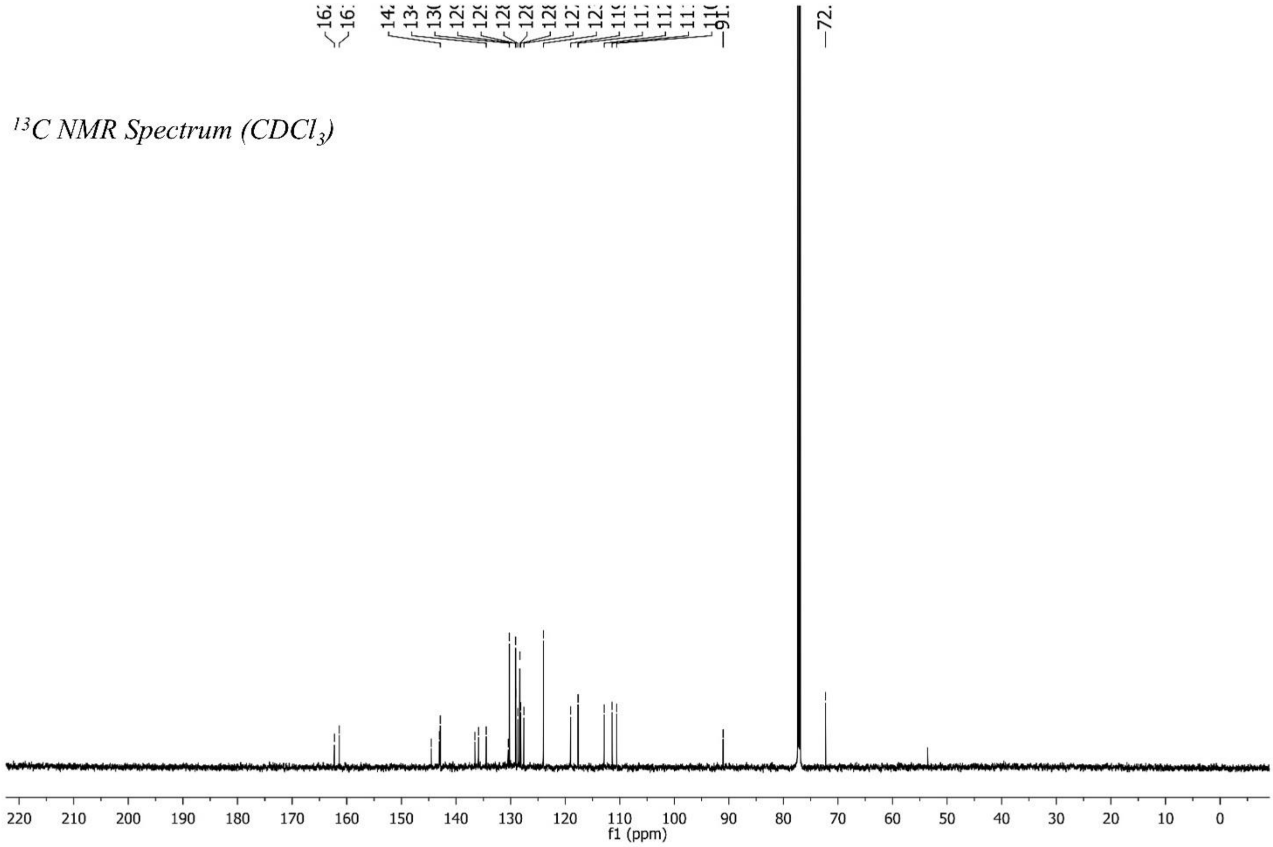

2-(Benzyloxy)-N-(1-phenyl-1H-benzo[d][1,2,3]triazol-5-yl)pyrazolo[1,5-a]pyridine-3-carboxamide (**30**)

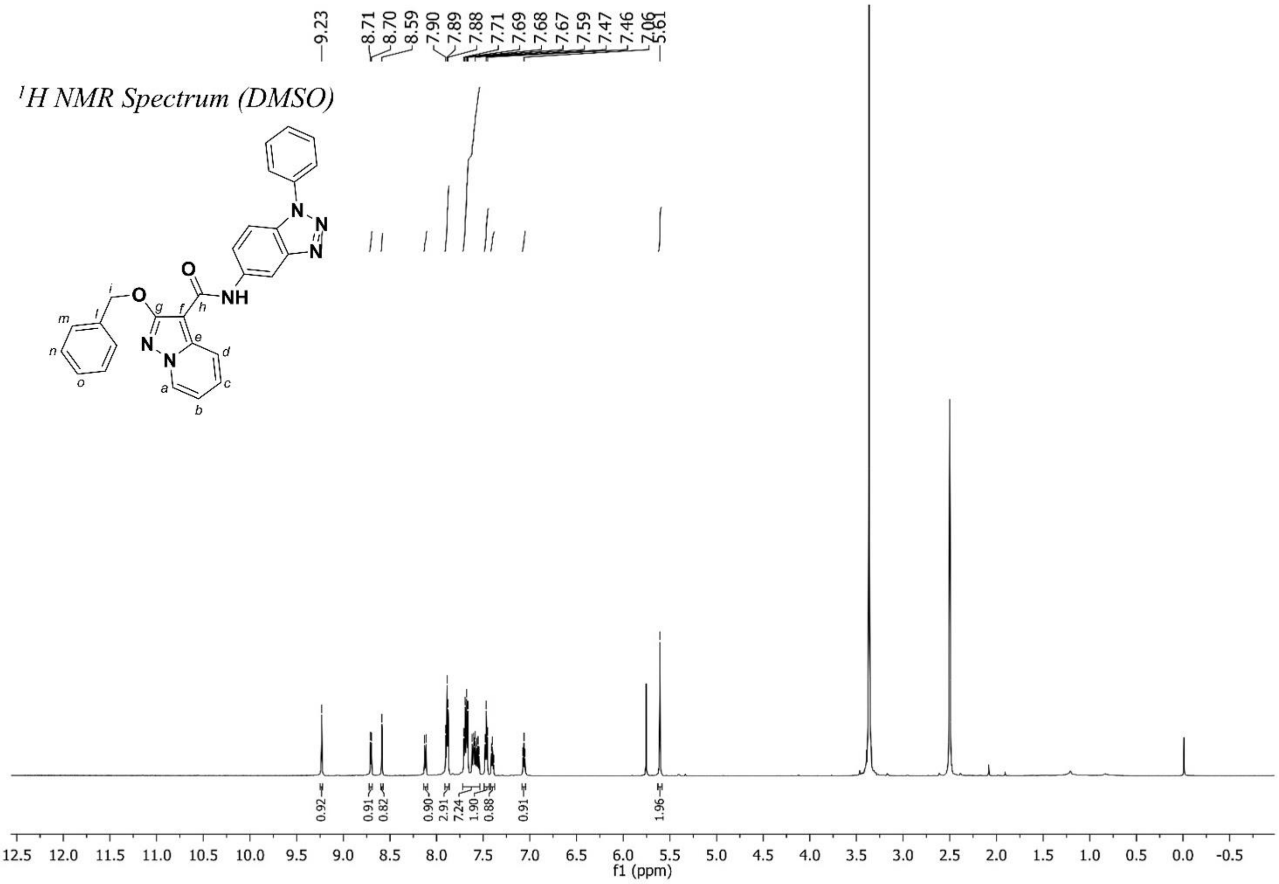

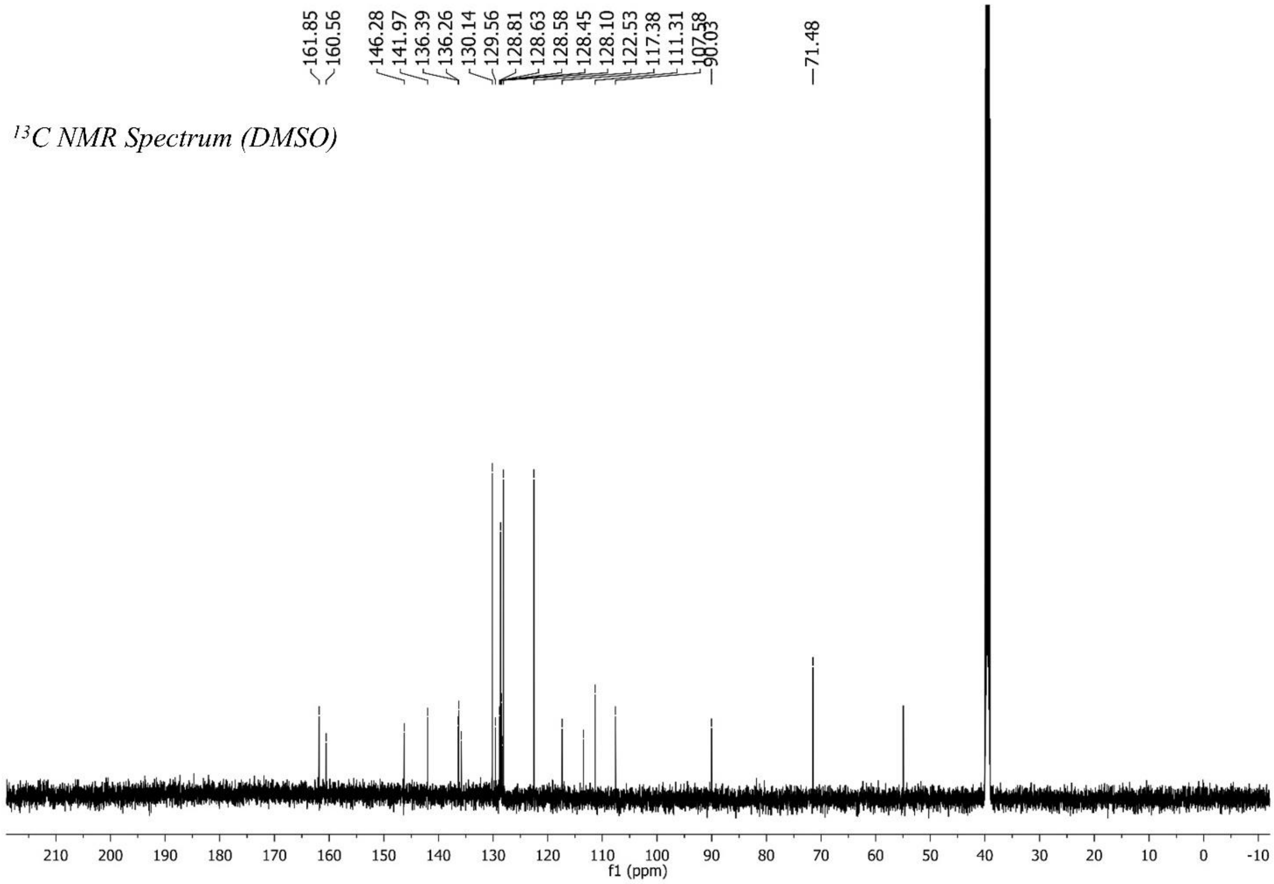

N-(2,5-dimethyl-4-(pyridin-4-ylthio)phenyl)-2-((4-methoxybenzyl)oxy)pyrazolo[1,5-a]pyridine-3-carboxamide (**37**)

^{1}H NMR Spectrum ($CDCl_3$)

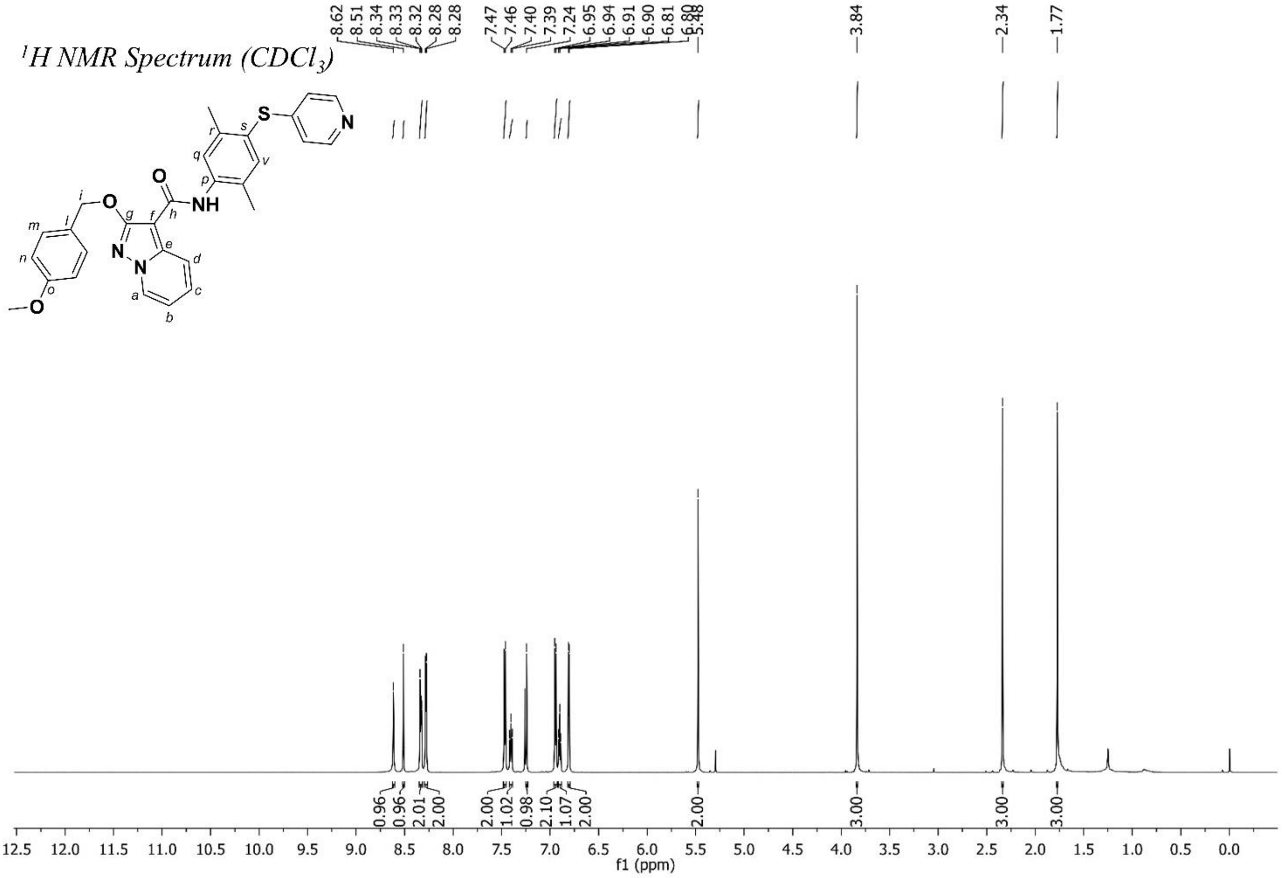

^{13}C NMR Spectrum ($CDCl_3$)

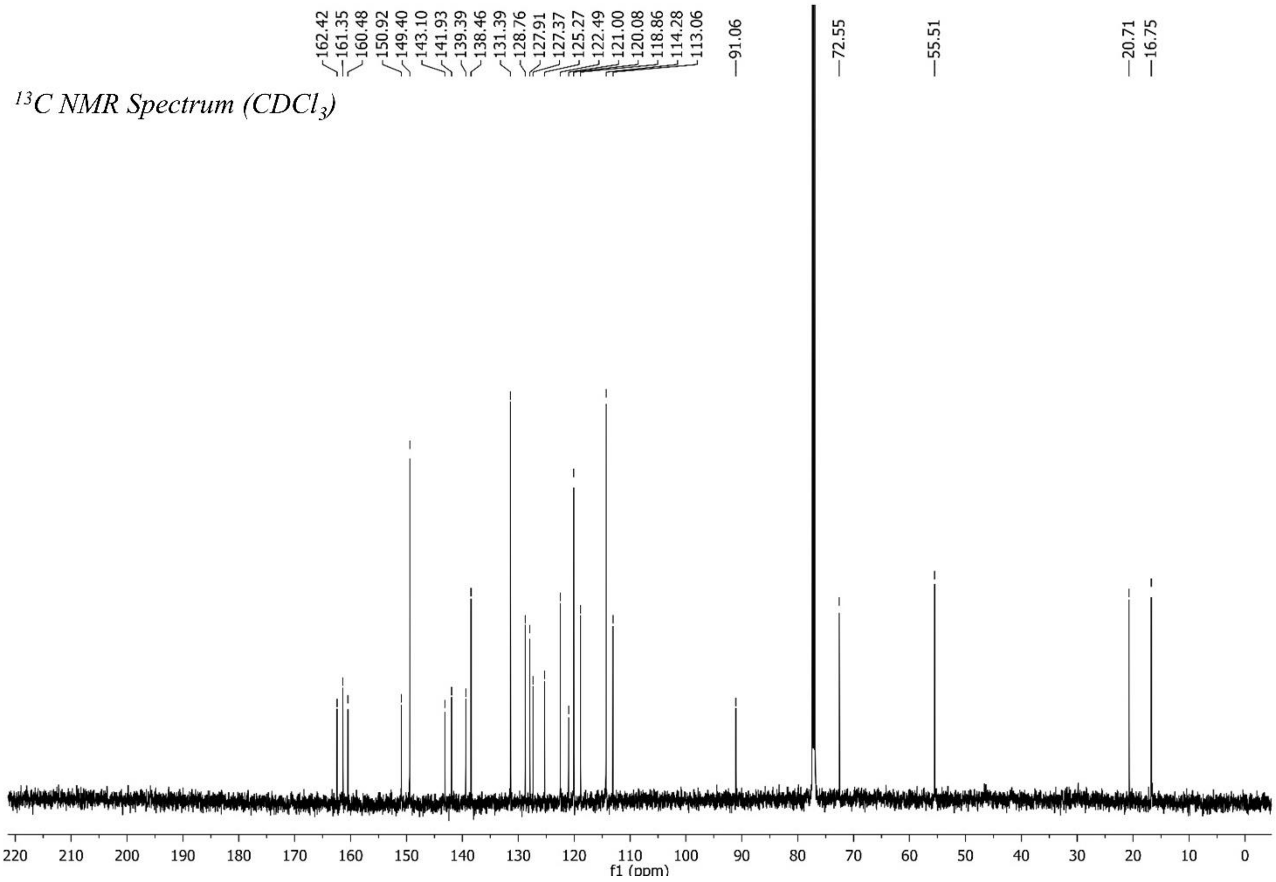

2-(Benzyloxy)-N-(2-isopropoxy-5-methyl-4-phenoxyphenyl)pyrazolo[1,5-a]pyridine-3-carboxamide (**32**)

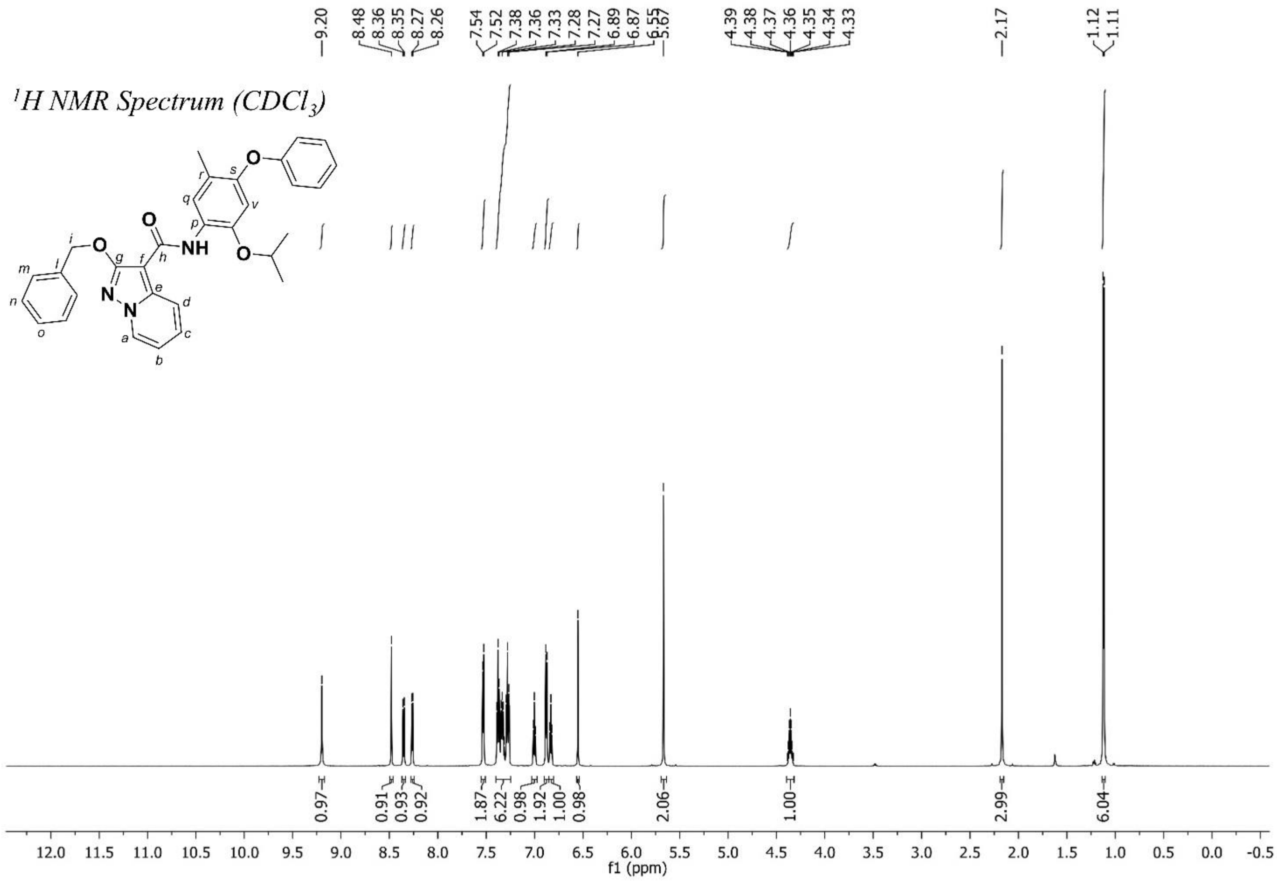

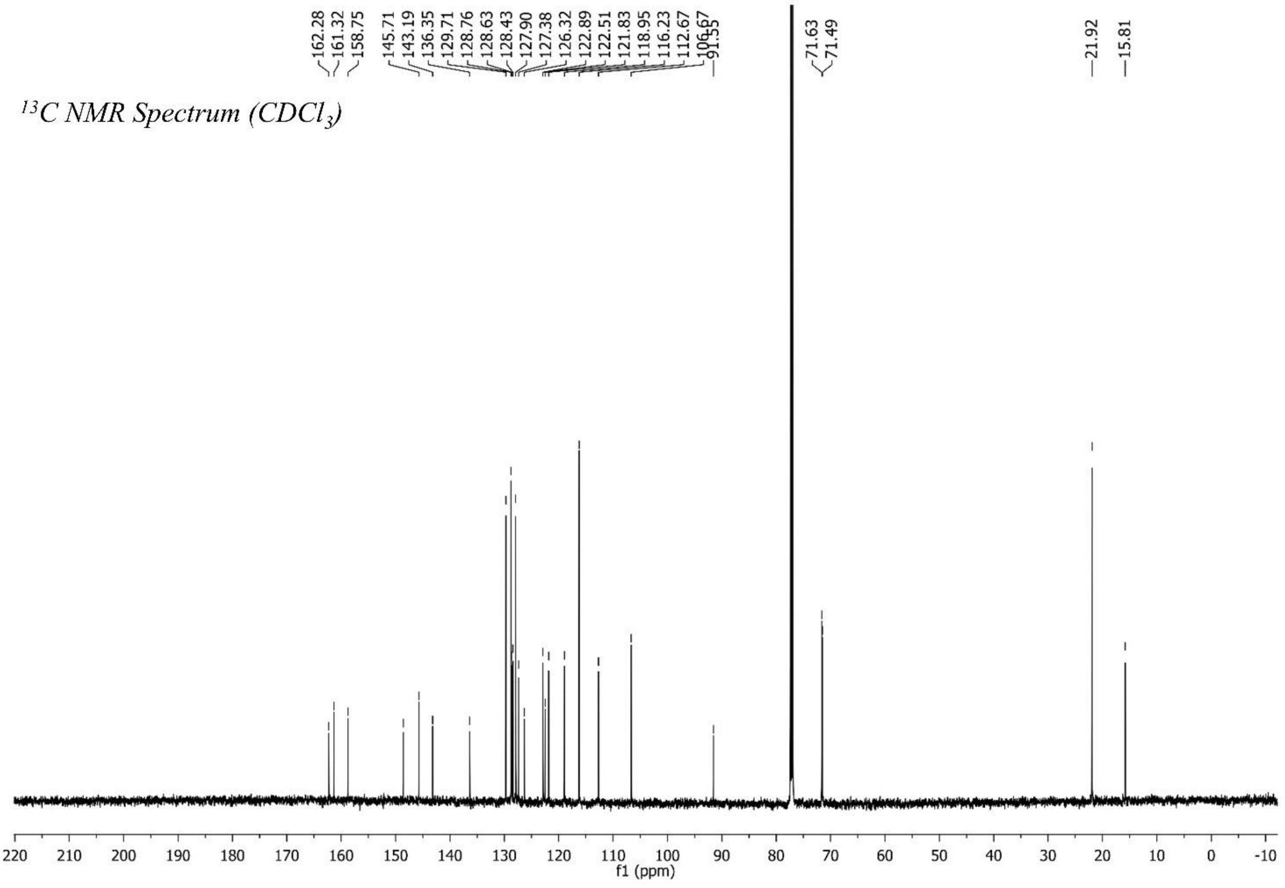

N-(2-Cyclobutoxy-5-methyl-4-phenoxyphenyl)-2-((4-methoxybenzyl)oxy)pyrazolo[1,5-a]pyridine-3-carboxamide (**33**)

^{1}H NMR Spectrum (CDCl$_3$)

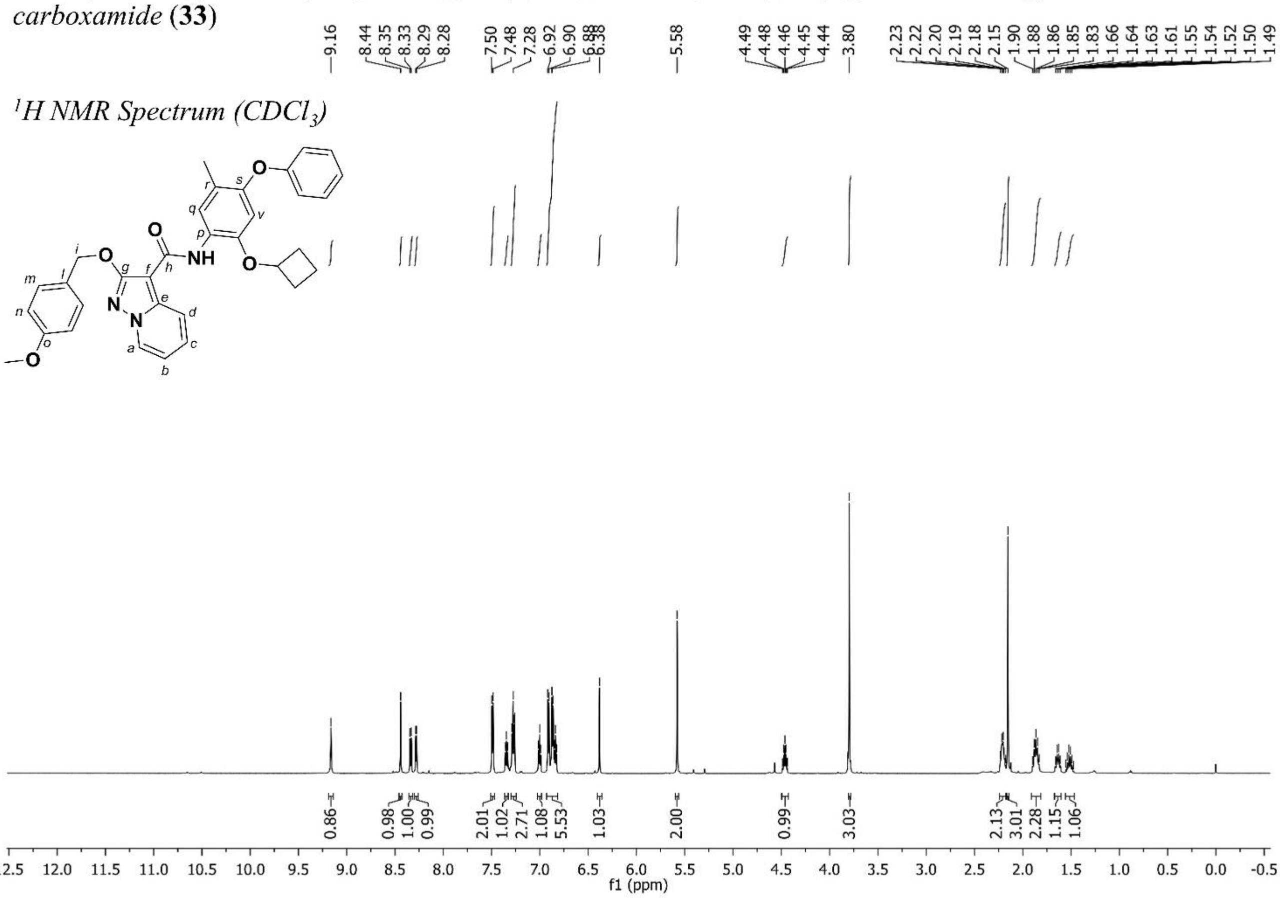

^{13}C NMR Spectrum (CDCl$_3$)

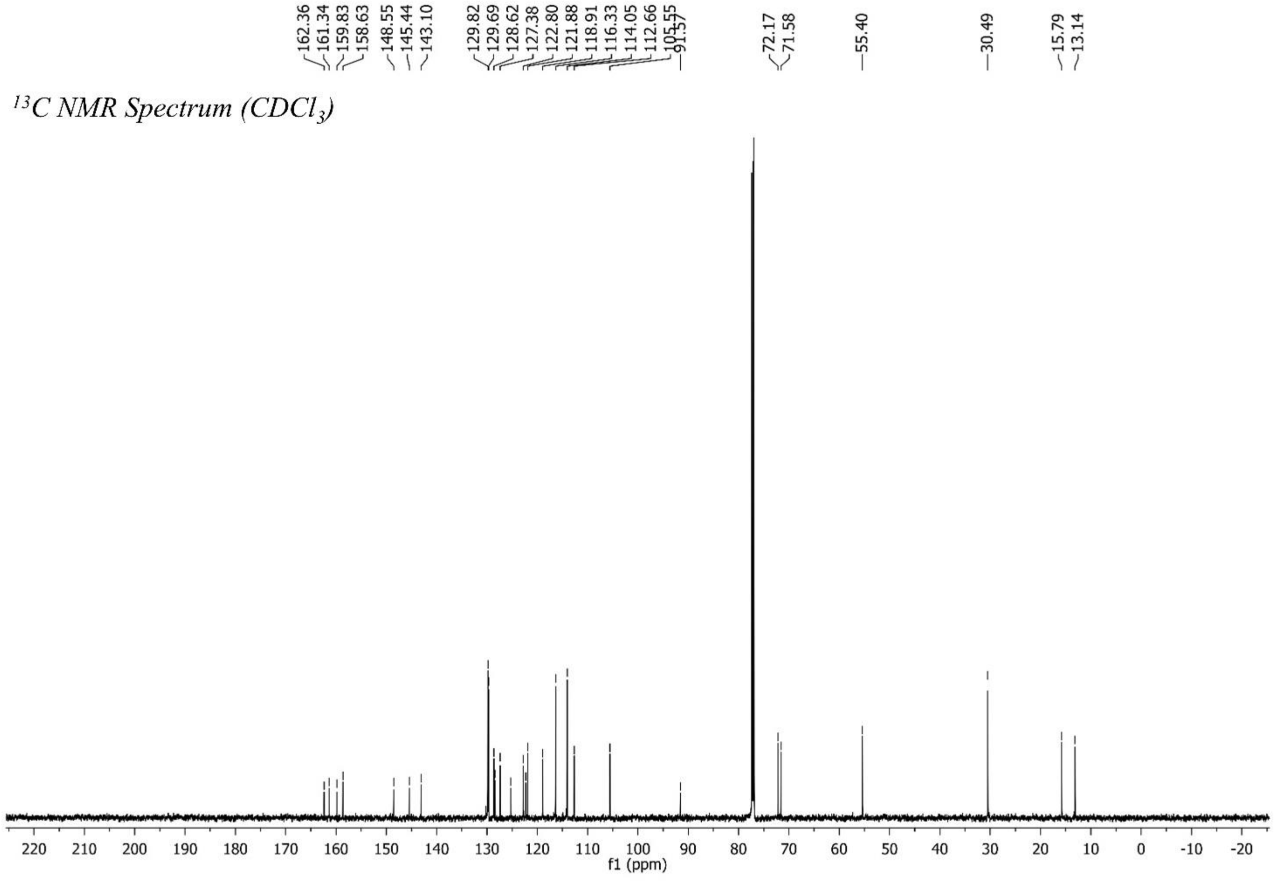

N-(2-(Sec-butoxy)-5-methyl-4-phenoxyphenyl)-2-((4-methoxybenzyl)oxy)pyrazolo[1,5-a]pyridine-3-carboxamide (**34**)

^{1}H NMR Spectrum (CDCl$_3$)

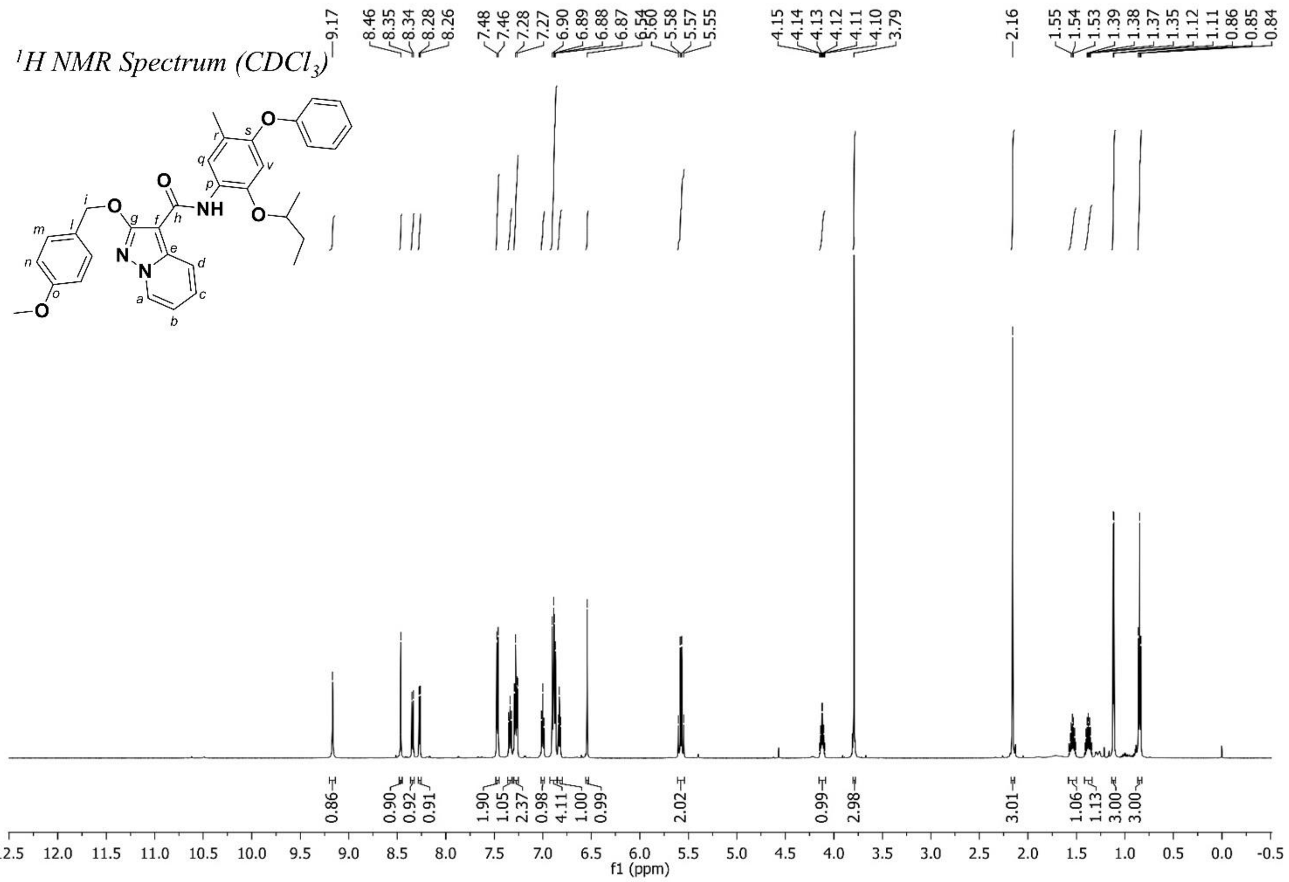

^{13}C NMR Spectrum (CDCl$_3$)

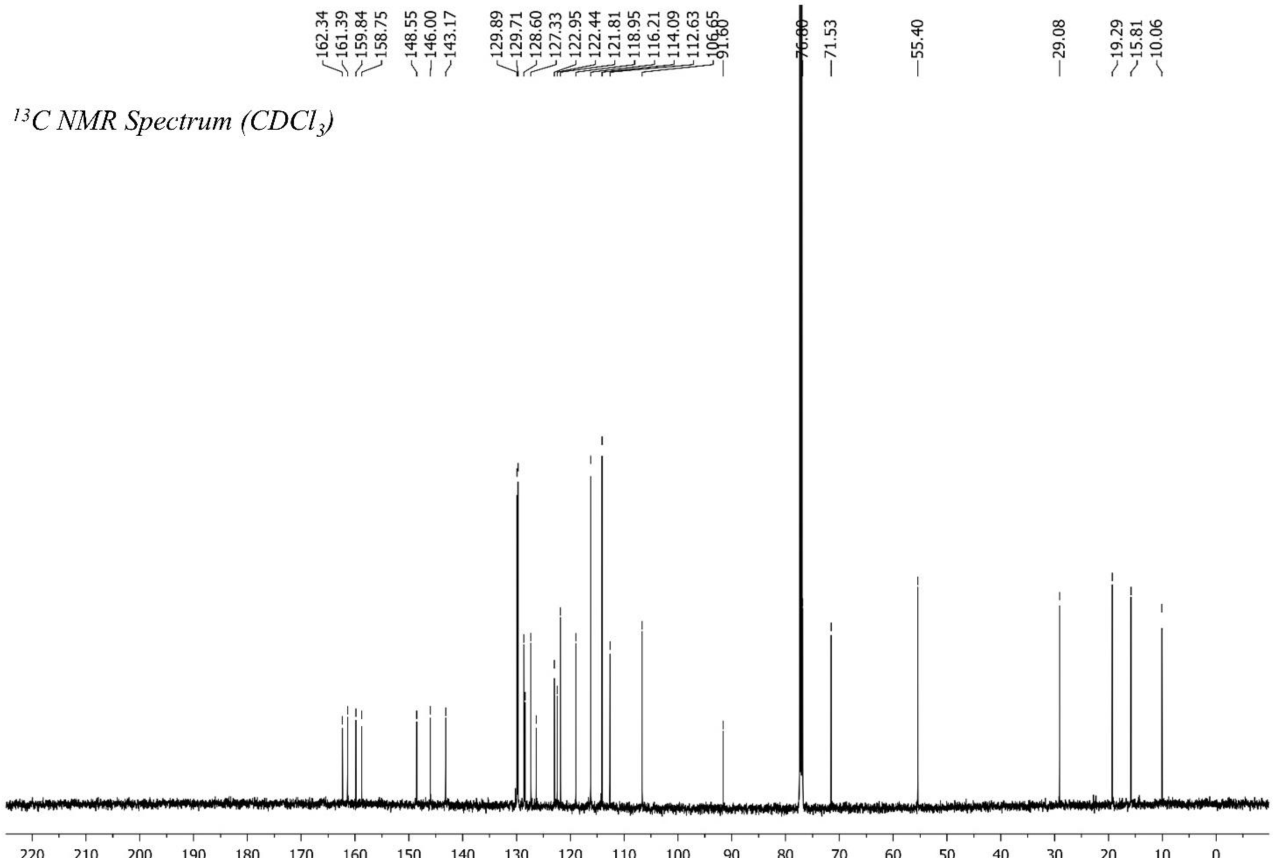

2-((4-Methoxybenzyl)oxy)-N-(5-methyl-2-(pentan-3-yloxy)-4-phenoxyphenyl)pyrazolo[1,5-a]pyridine-3-carboxamide (**35**)

^{1}H NMR Spectrum ($CDCl_3$)

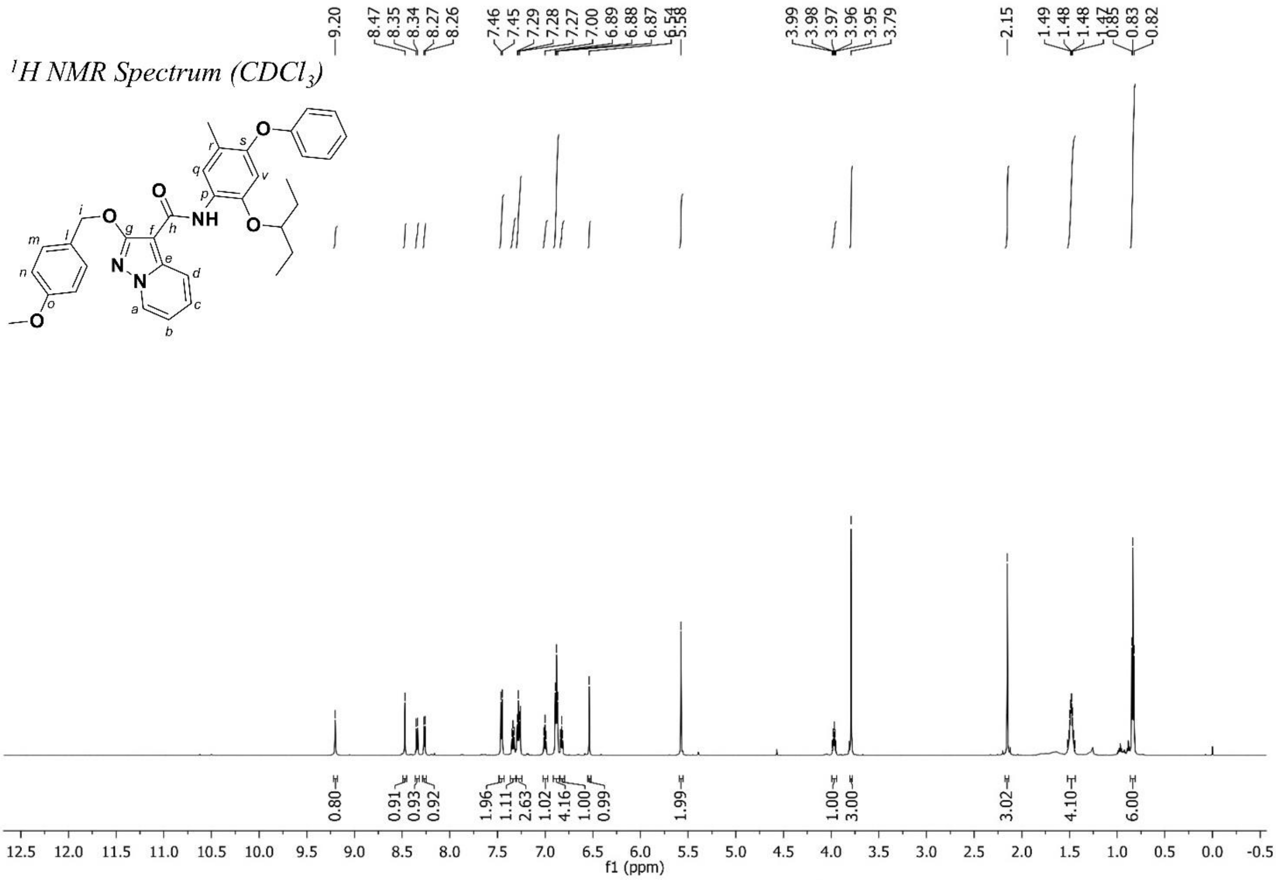

^{13}C NMR Spectrum ($CDCl_3$)

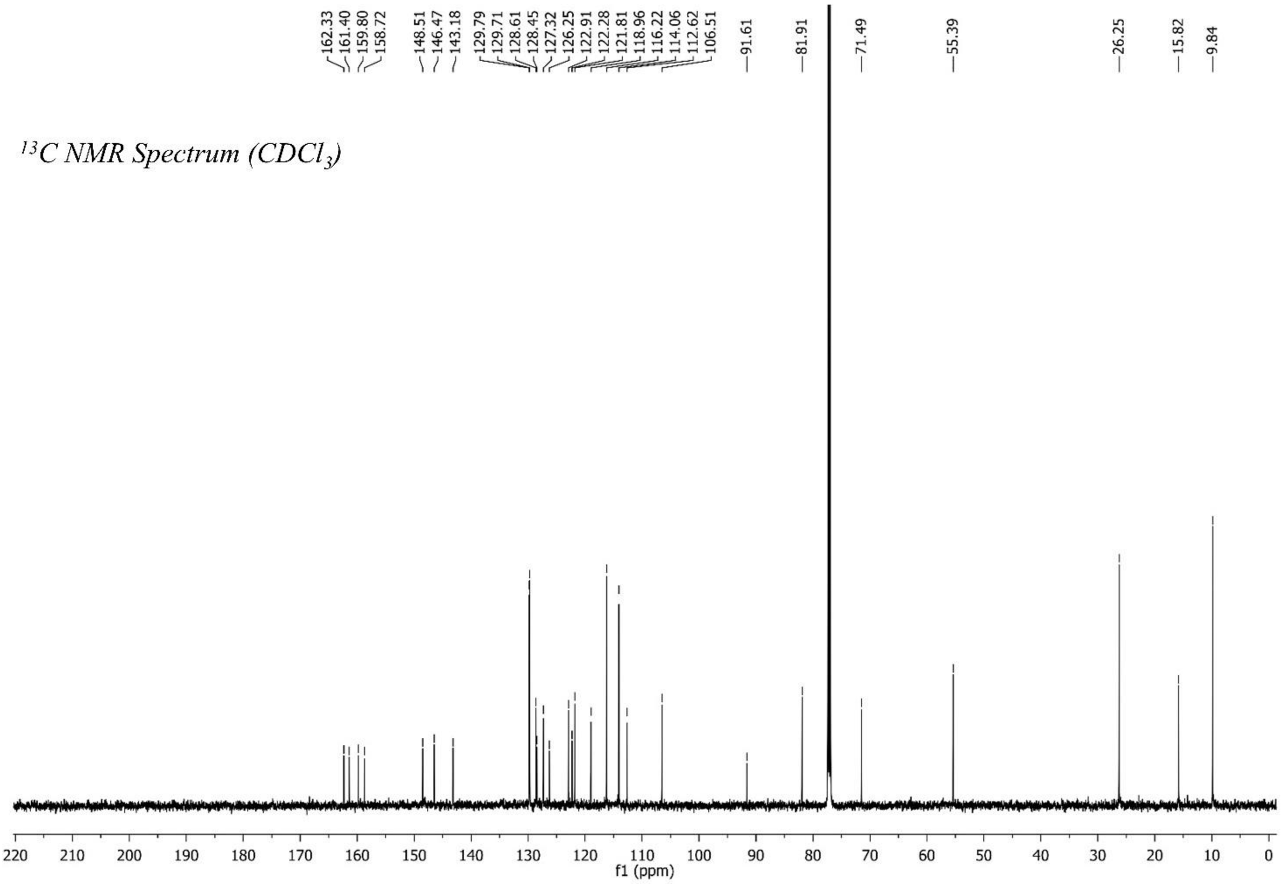

2-((4-Methoxybenzyl)oxy)-N-(5-methyl-2-(pentan-2-yloxy)-4-phenoxyphenyl)pyrazolo[1,5-a]pyridine-3-carboxamide (**36**)

^{1}H NMR Spectrum (CDCl$_3$)

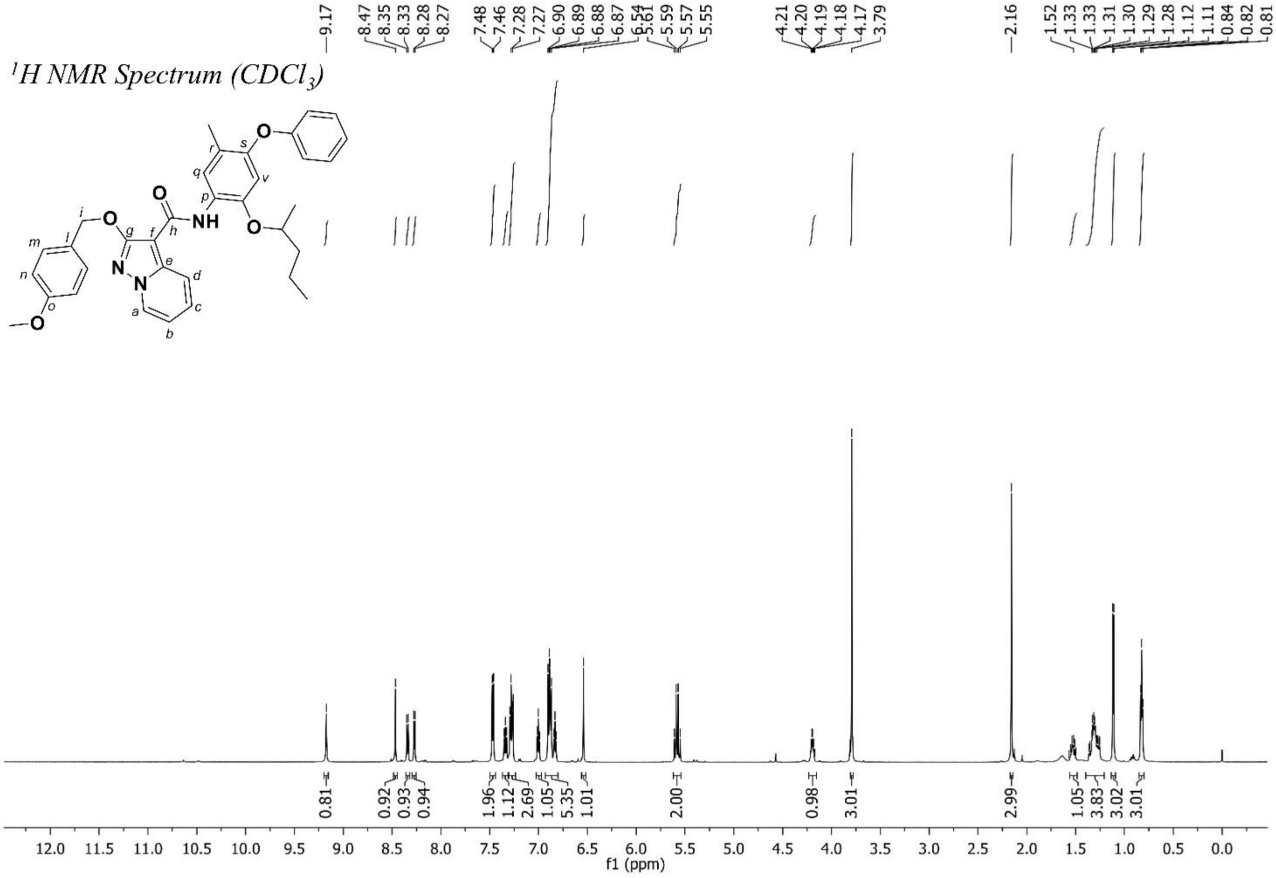

^{13}C NMR Spectrum (CDCl$_3$)

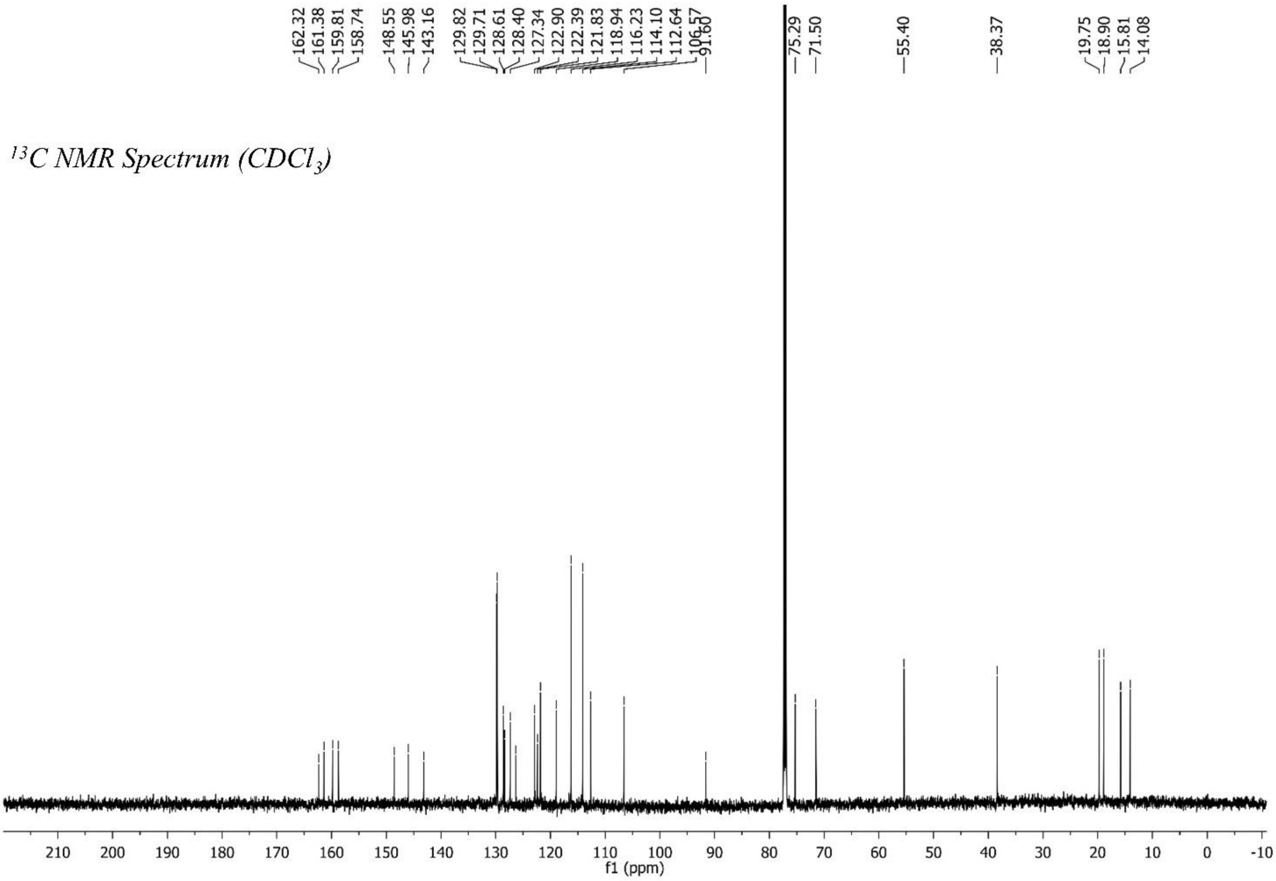

N-(2-Isopropyl-5-methyl-4-(pyridin-4-yloxy)phenyl)-2-((4-methoxybenzyl)oxy)pyrazolo[1,5-a]pyridine-3-carboxamide (**38**)

^{1}H NMR Spectrum (CDCl$_3$)

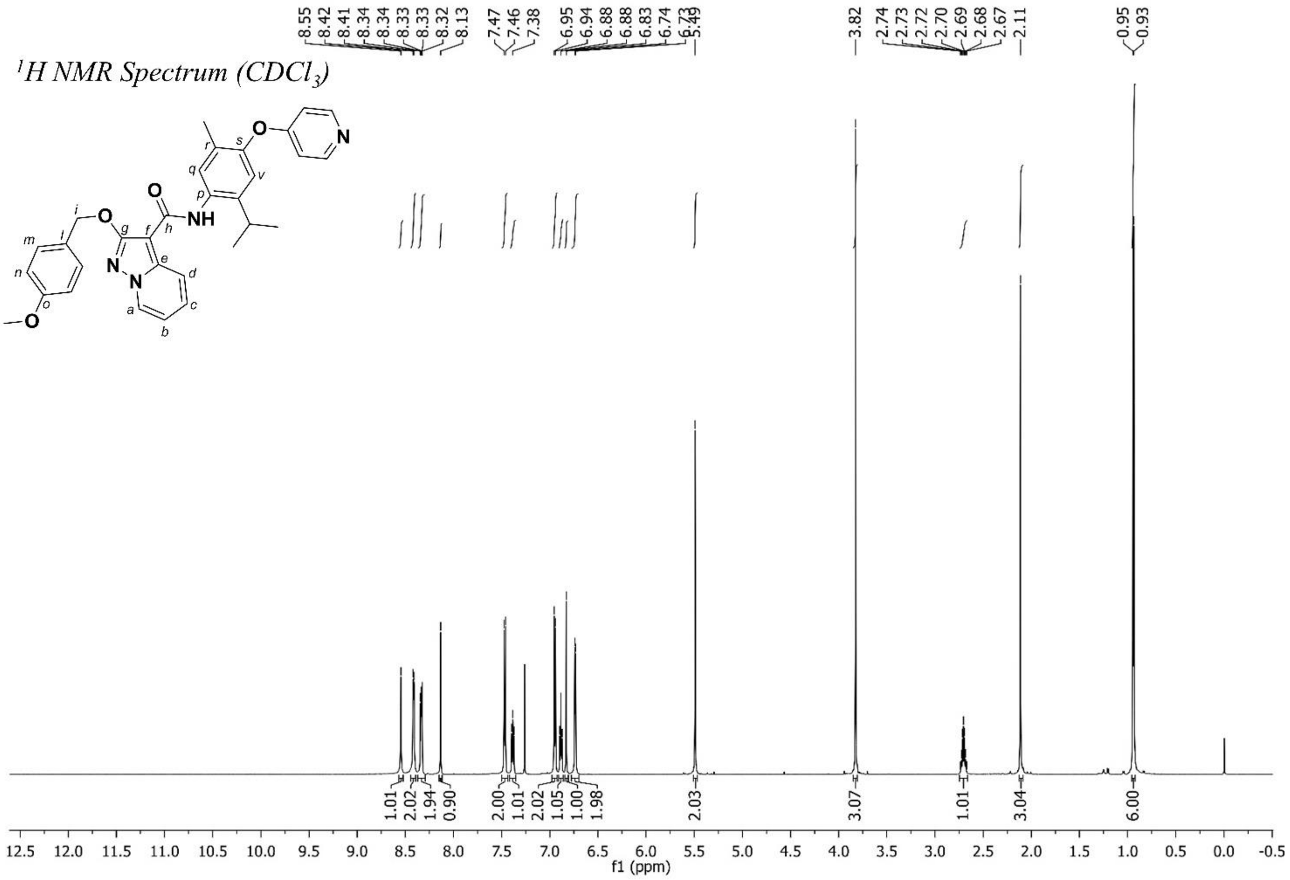

^{13}C NMR Spectrum (CDCl$_3$)

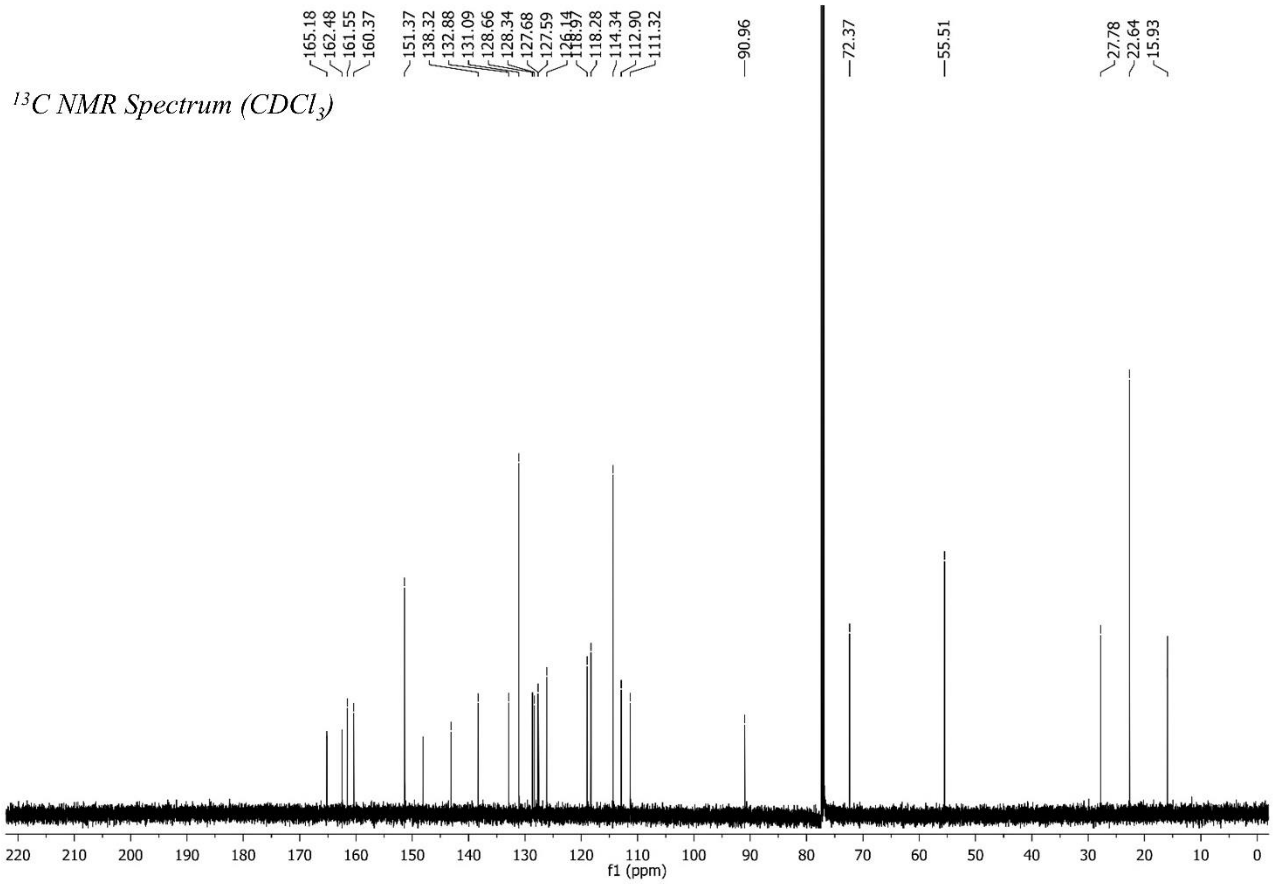

2-Hydroxy-N-(1H-indol-5-yl)pyrazolo[1,5-a]pyridine-3-carboxamide (**11**)

^{1}H NMR Spectrum (DMSO)

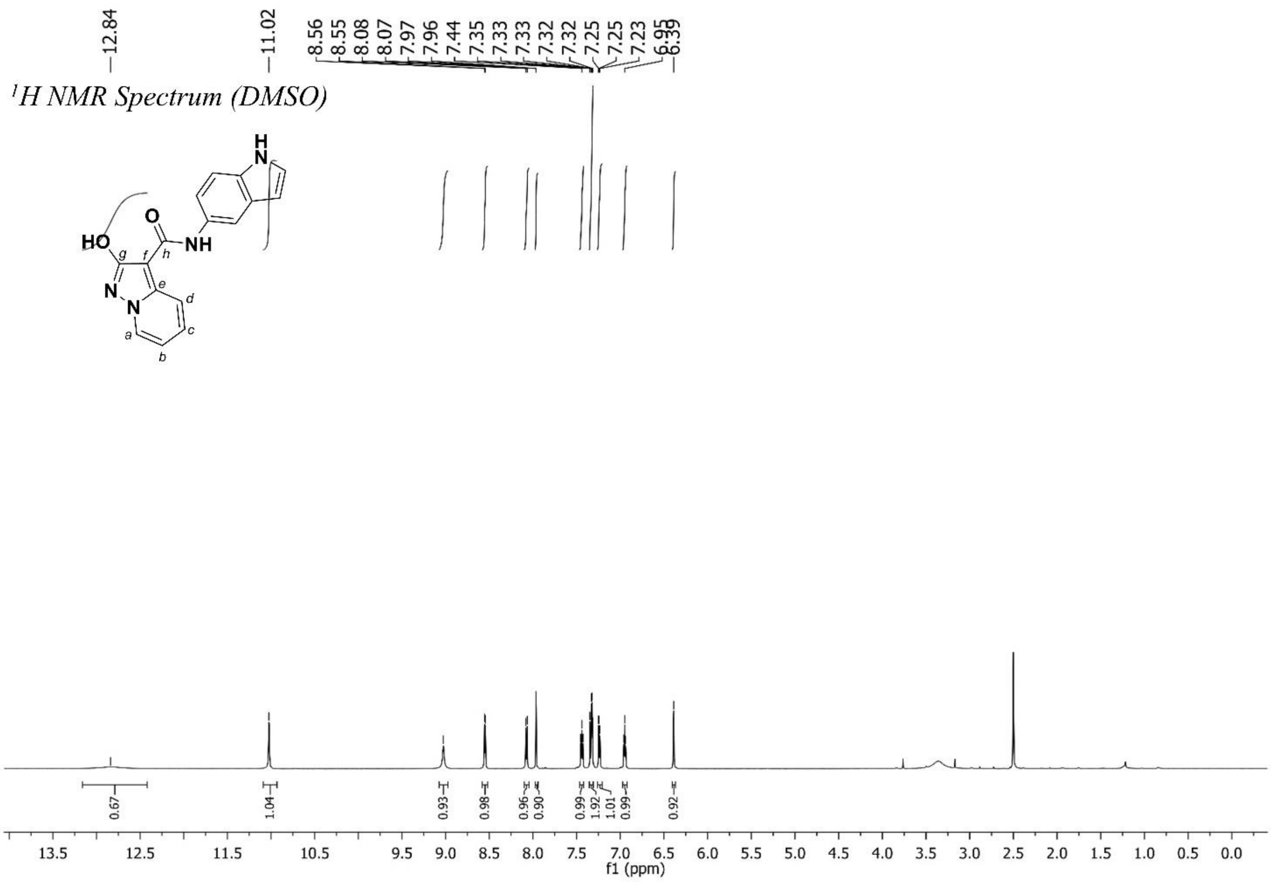

^{13}C NMR Spectrum (DMSO)

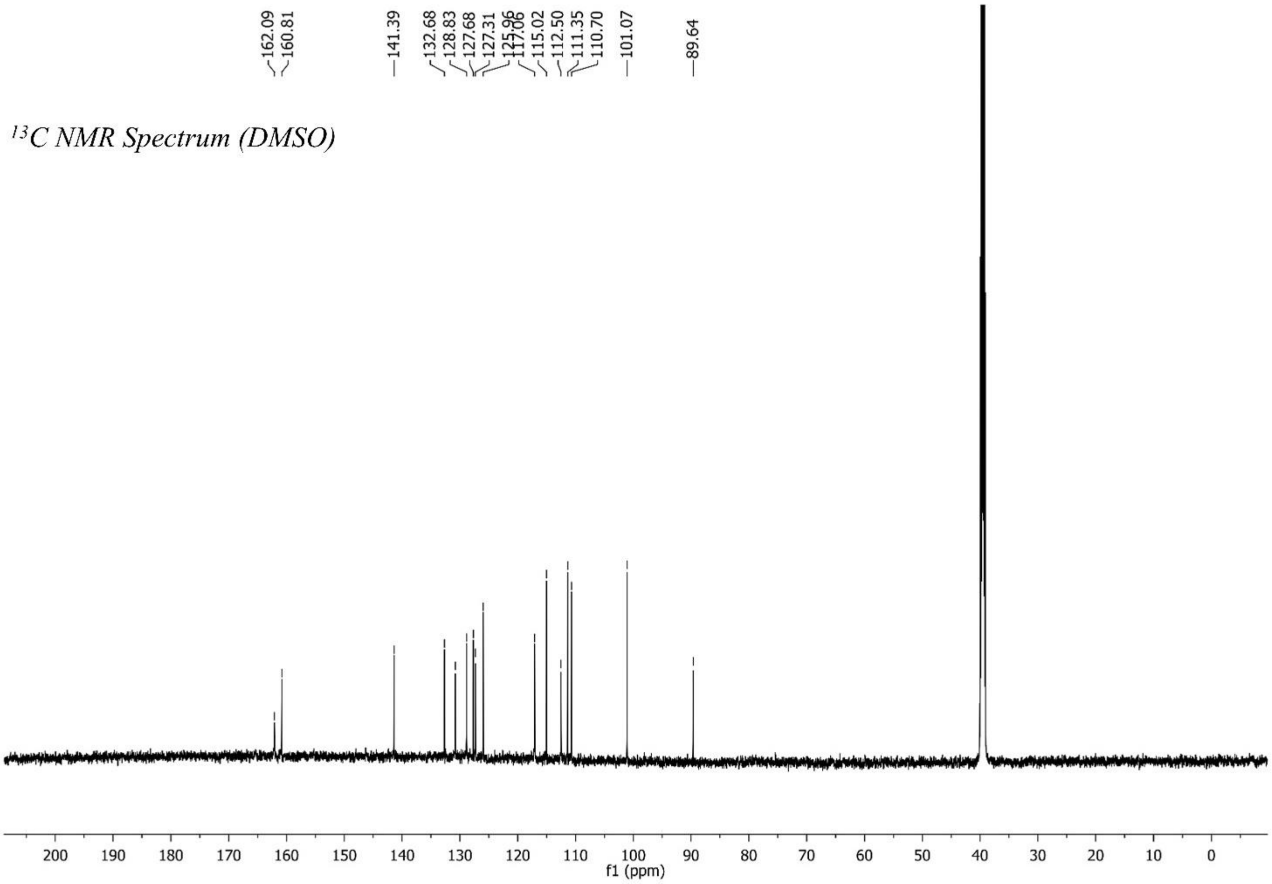

2-Hydroxy-N-(1-phenyl-1H-indol-5-yl)pyrazolo[1,5-a]pyridine-3-carboxamide (**12**)

^{1}H NMR Spectrum (DMSO)

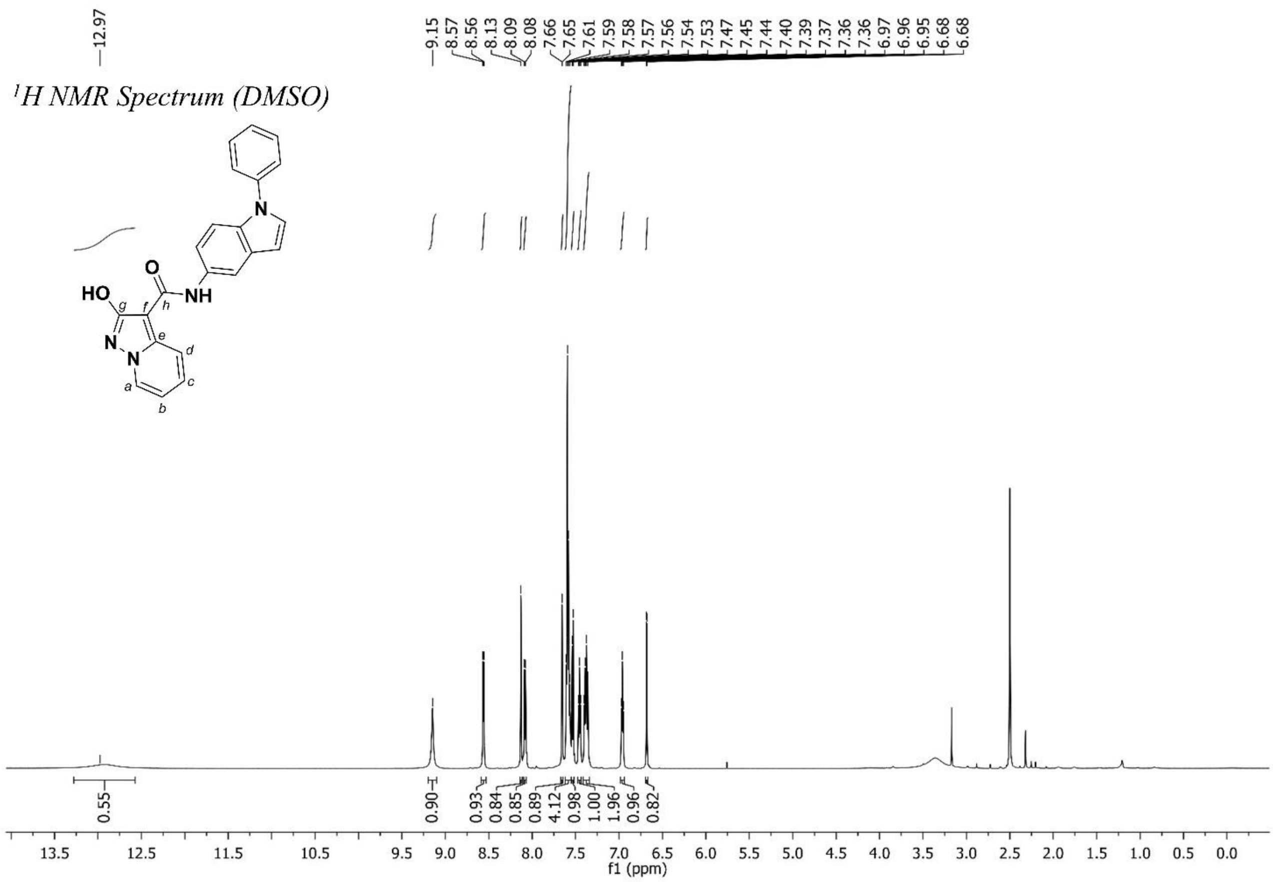

^{13}C NMR Spectrum (DMSO)

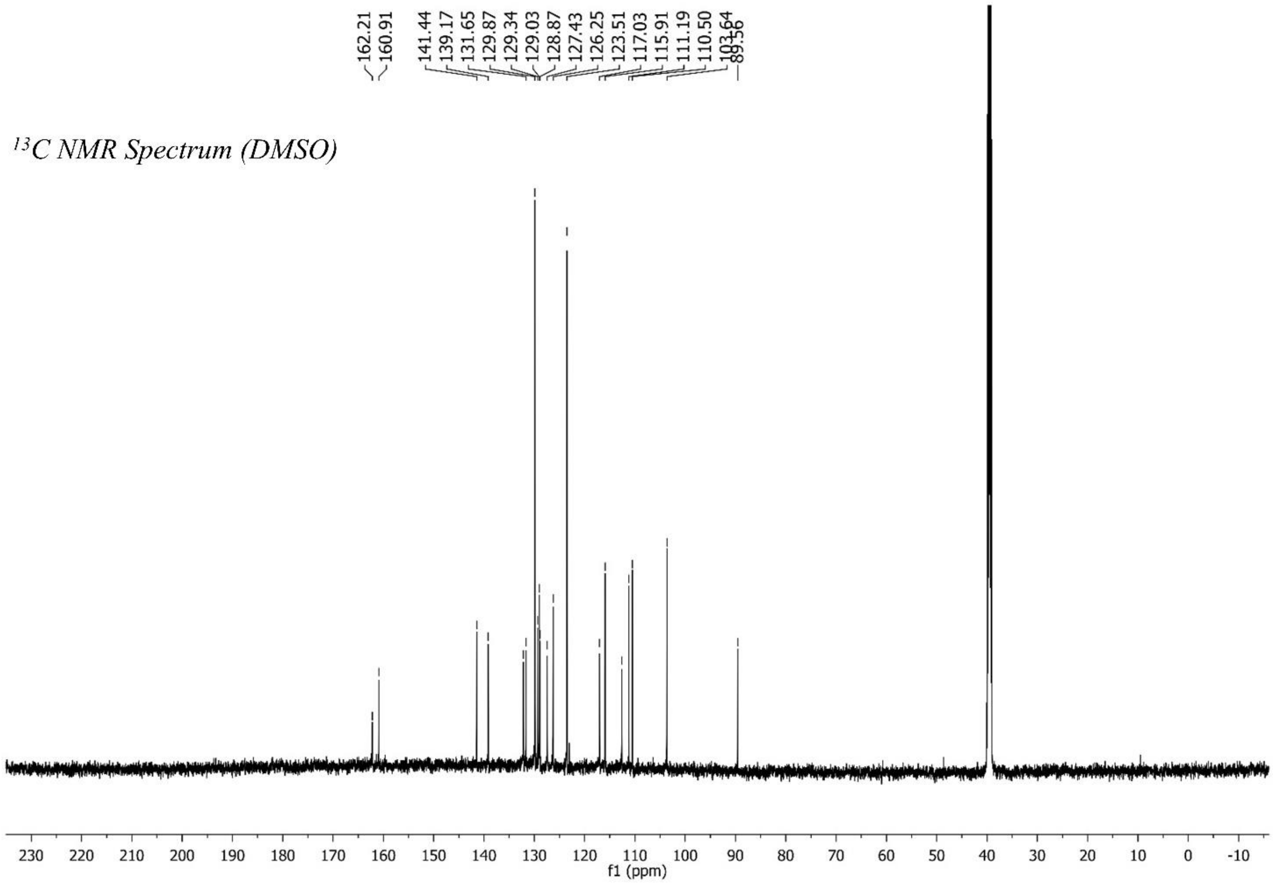

2-Hydroxy-N-(1-phenyl-1H-benzo[d]imidazol-5-yl)pyrazolo[1,5-a]pyridine-3-carboxamide (**13**)

^{1}H NMR Spectrum (DMSO)

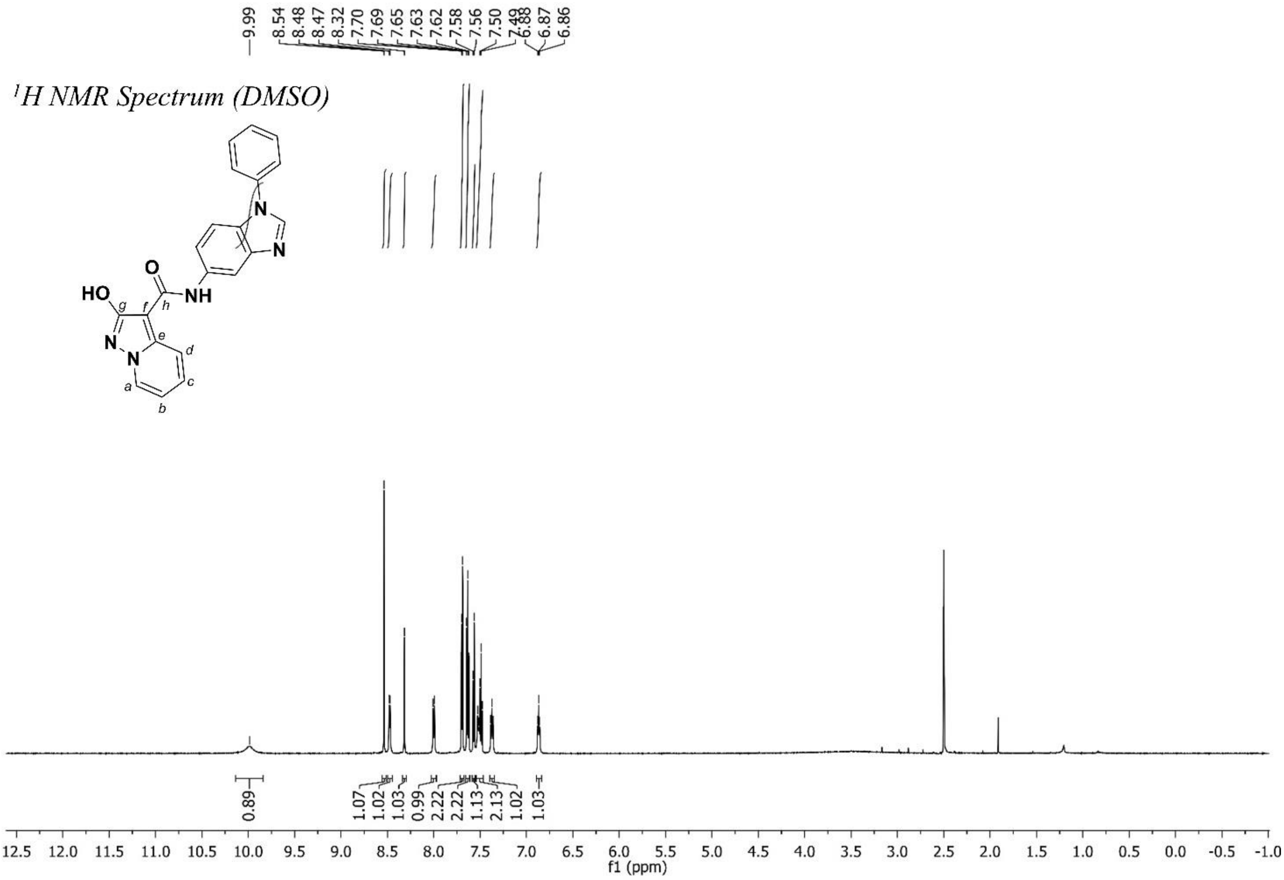

^{13}C NMR Spectrum (DMSO)

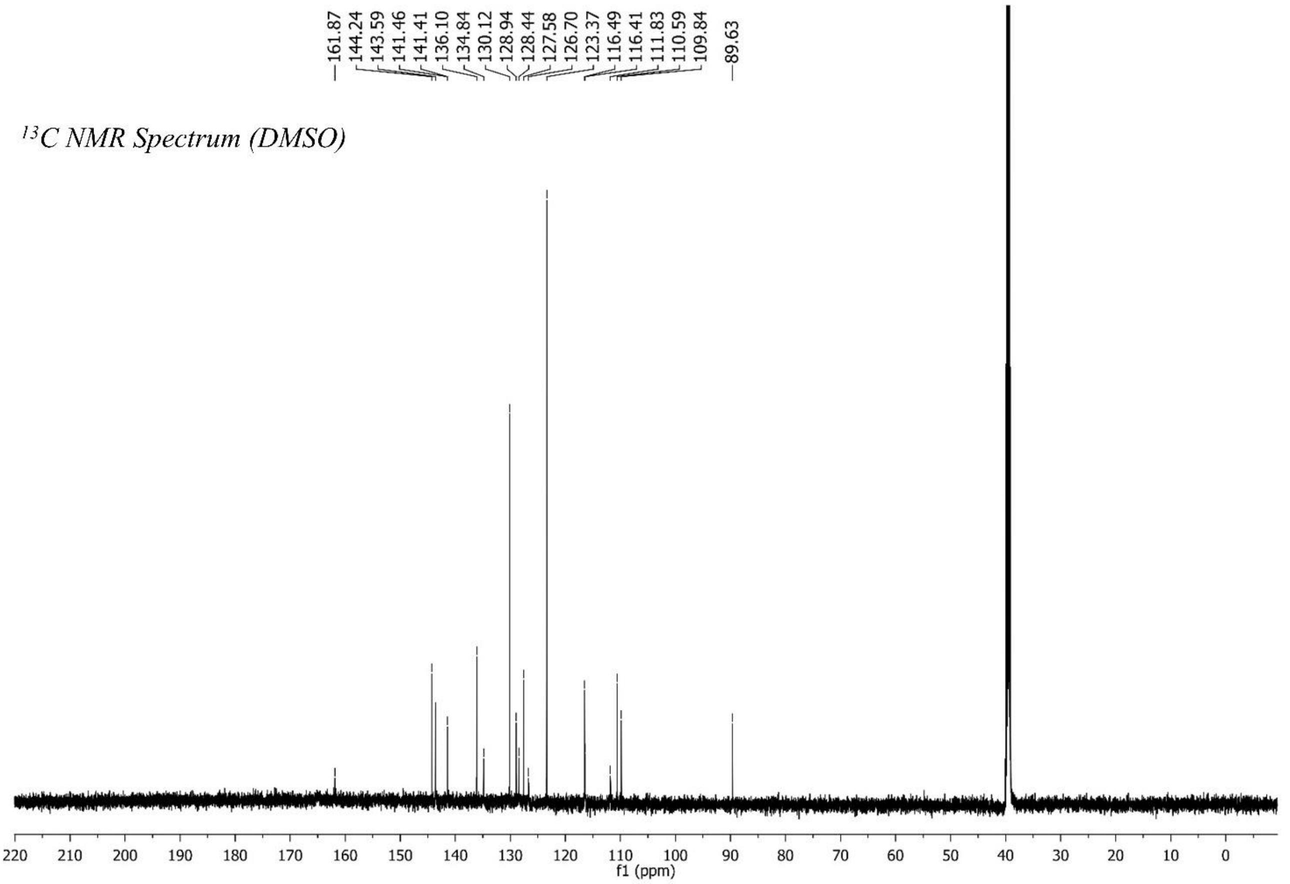

2-Hydroxy-N-(1-phenyl-1H-benzo[d][1,2,3]triazol-5-yl)pyrazolo[1,5-a]pyridine-3-carboxamide (**14**)

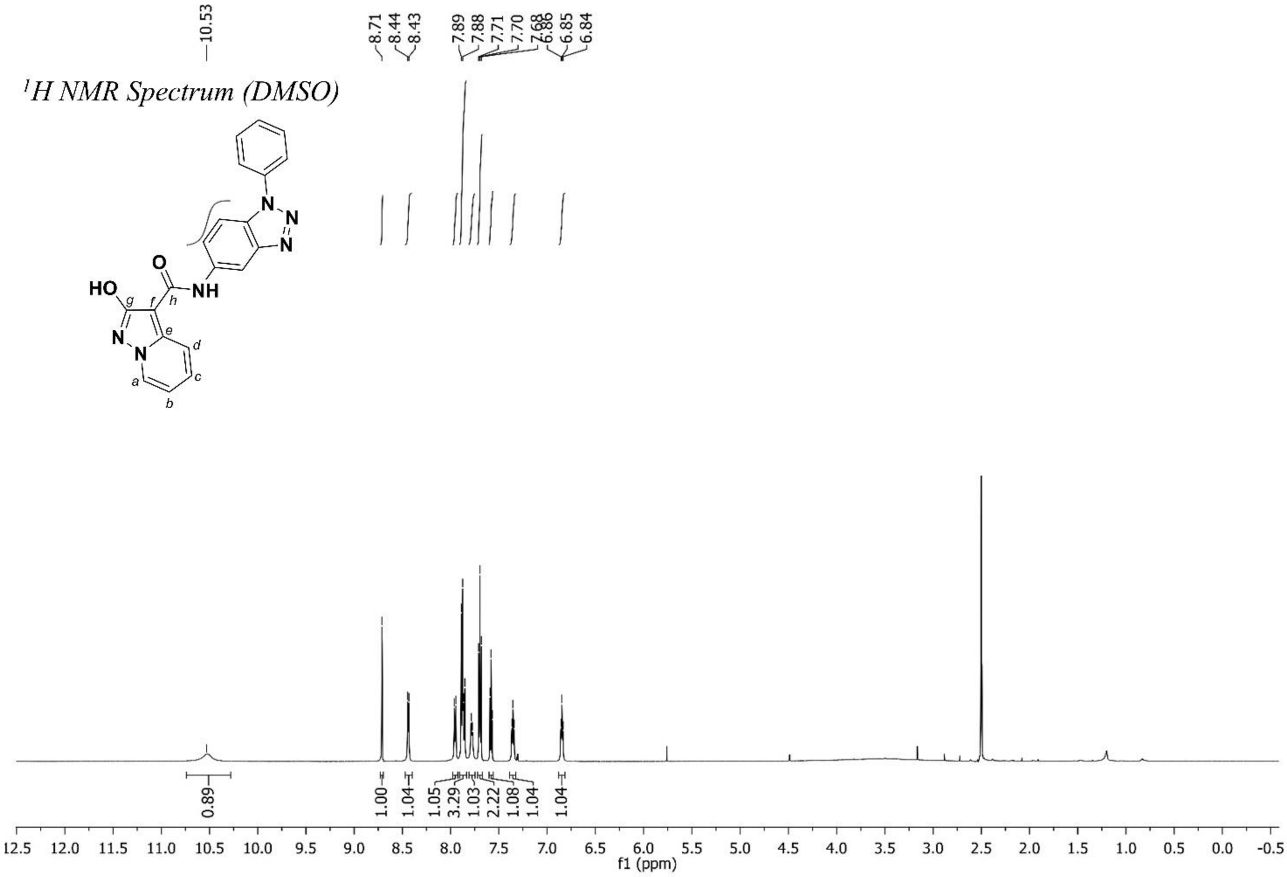

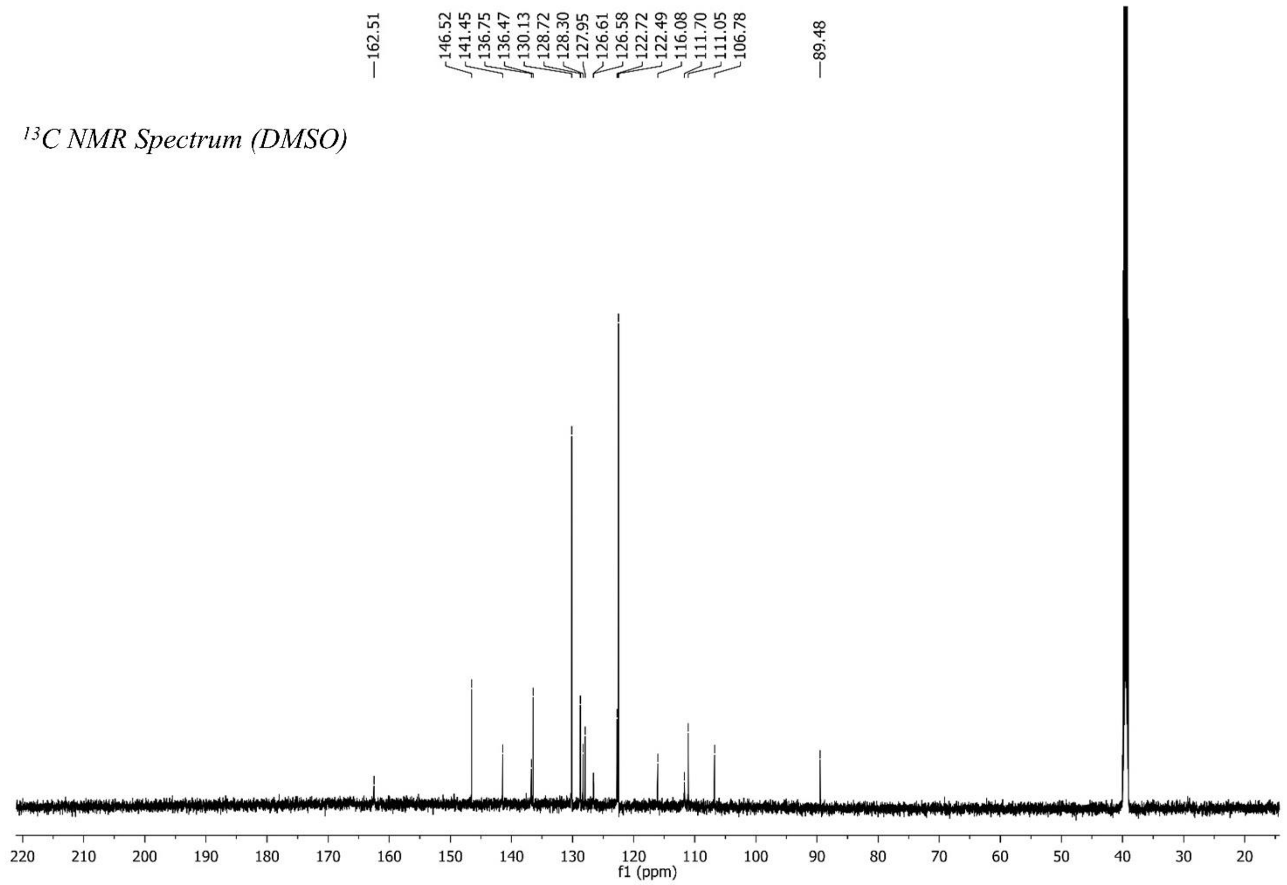

2-Hydroxy-N-(5-phenoxypyridin-2-yl)pyrazolo[1,5-a]pyridine-3-carboxamide (**15**)

^{1}H NMR Spectrum (DMSO)

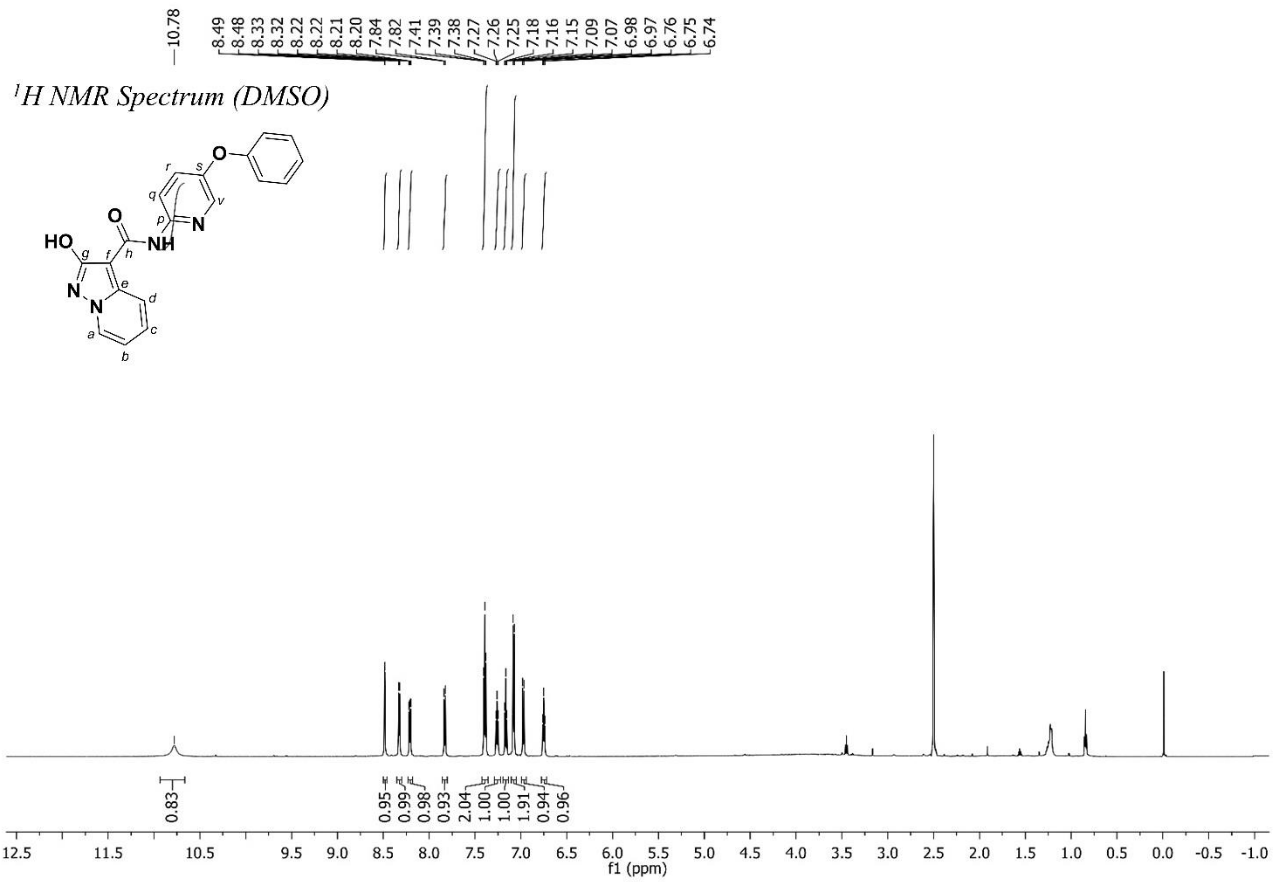

^{13}C NMR Spectrum (DMSO)

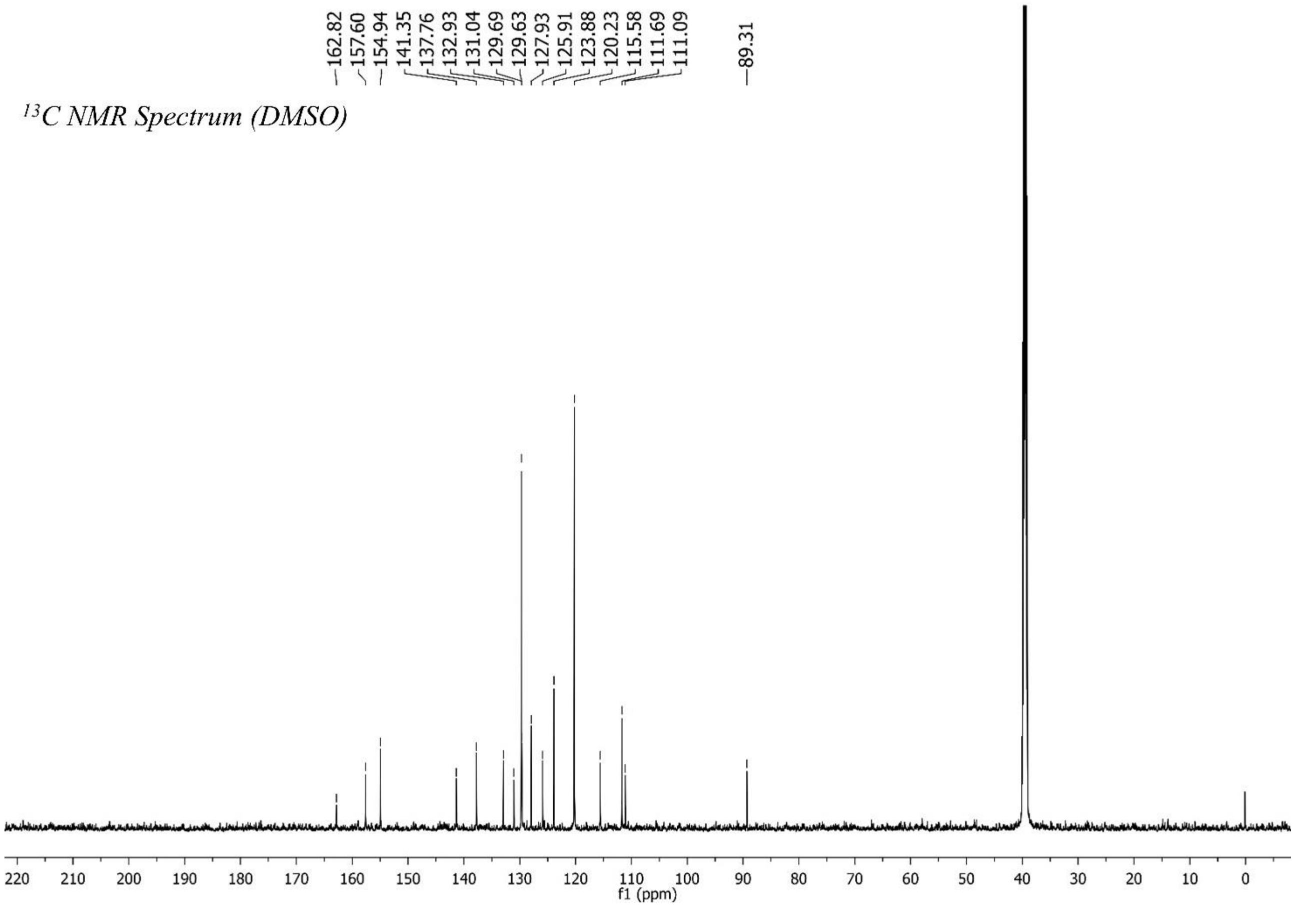

2-Hydroxy-N-5-[3-(trifluoromethyl)phenoxy]pyridin-2-ylpyrazolo[1,5-a]pyridine-3-carboxamide (**16**)

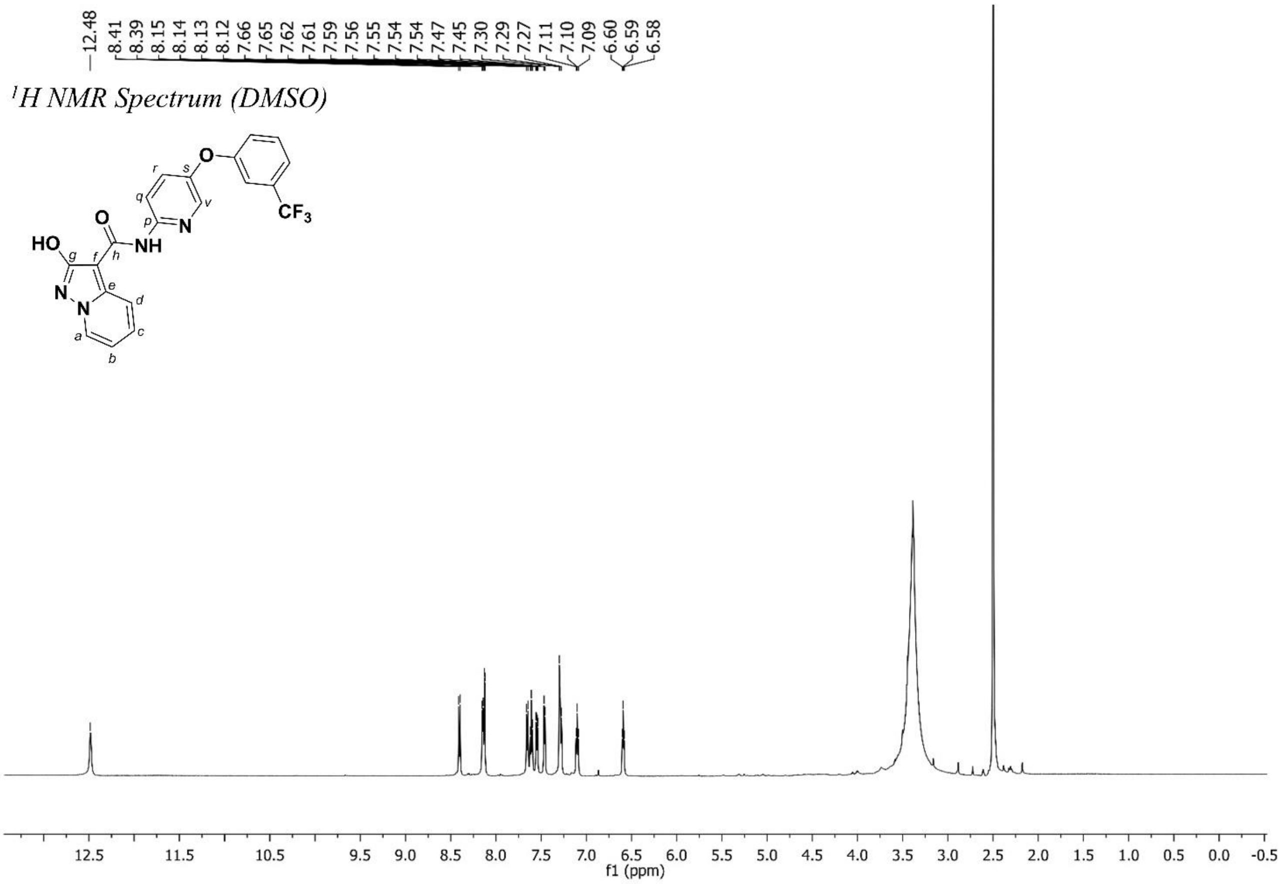

^{1}H NMR Spectrum (DMSO)

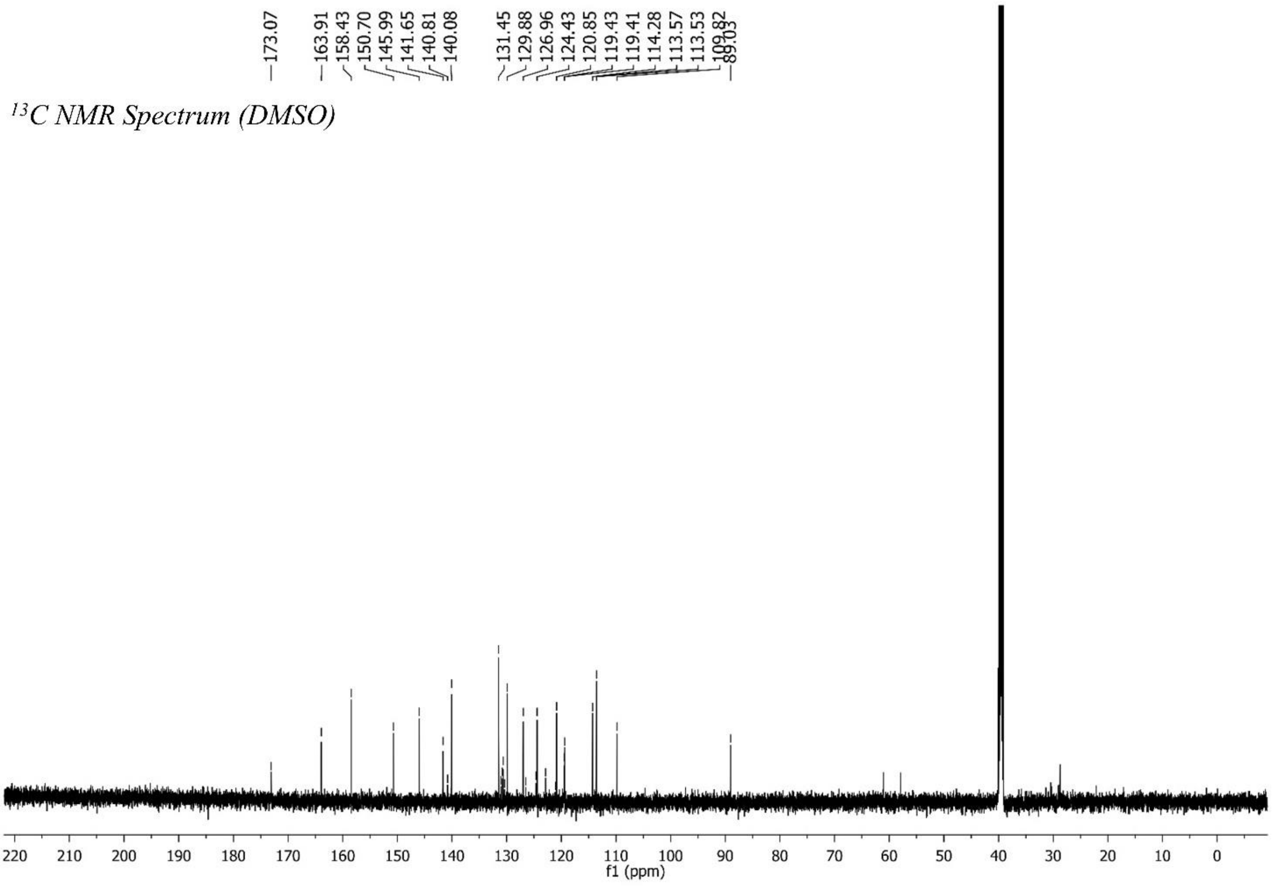

^{13}C NMR Spectrum (DMSO)

2-Hydroxy-N-(2,5-dimethyl-4-(pyridin-4-ylthio)phenyl) pyrazolo[1,5-a]pyridine-3-carboxamide (**17**)

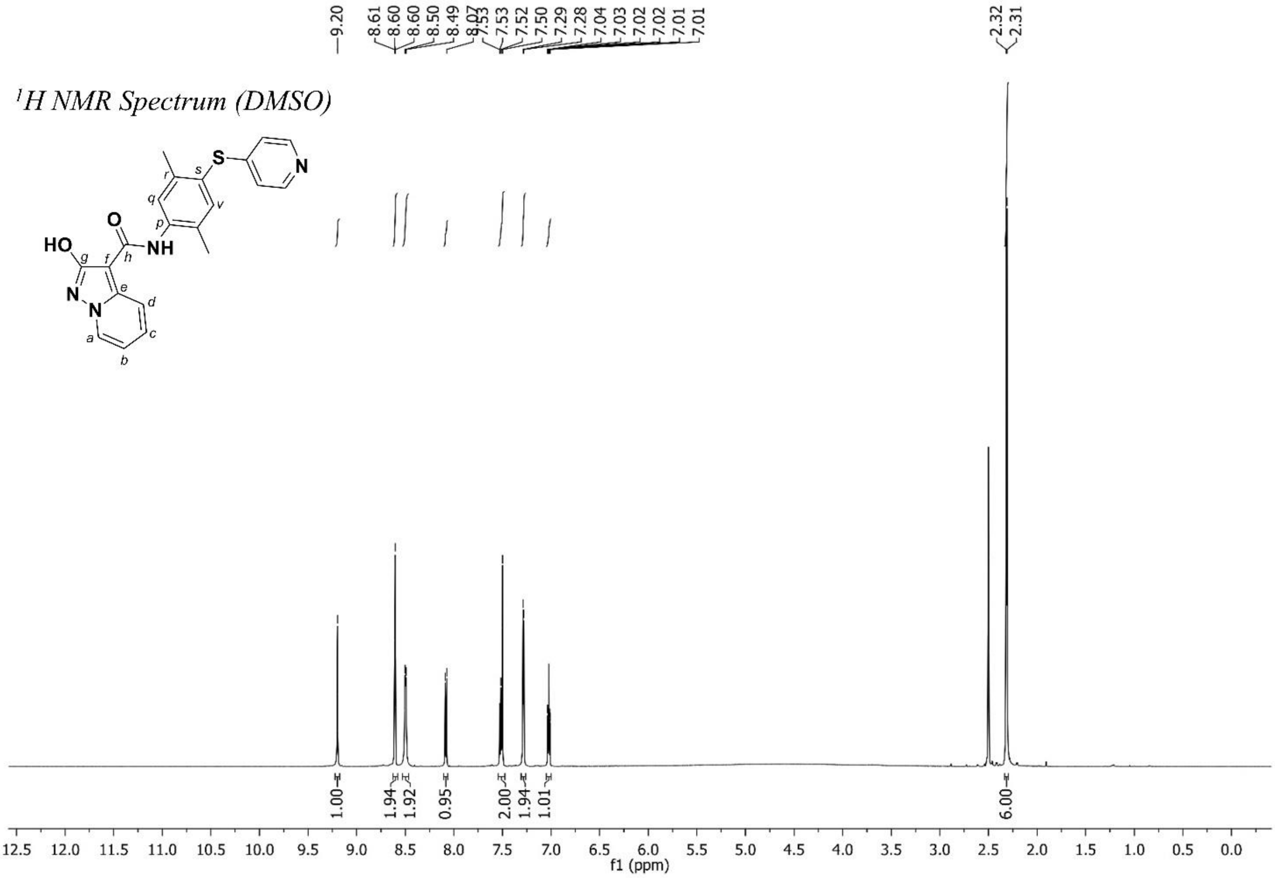

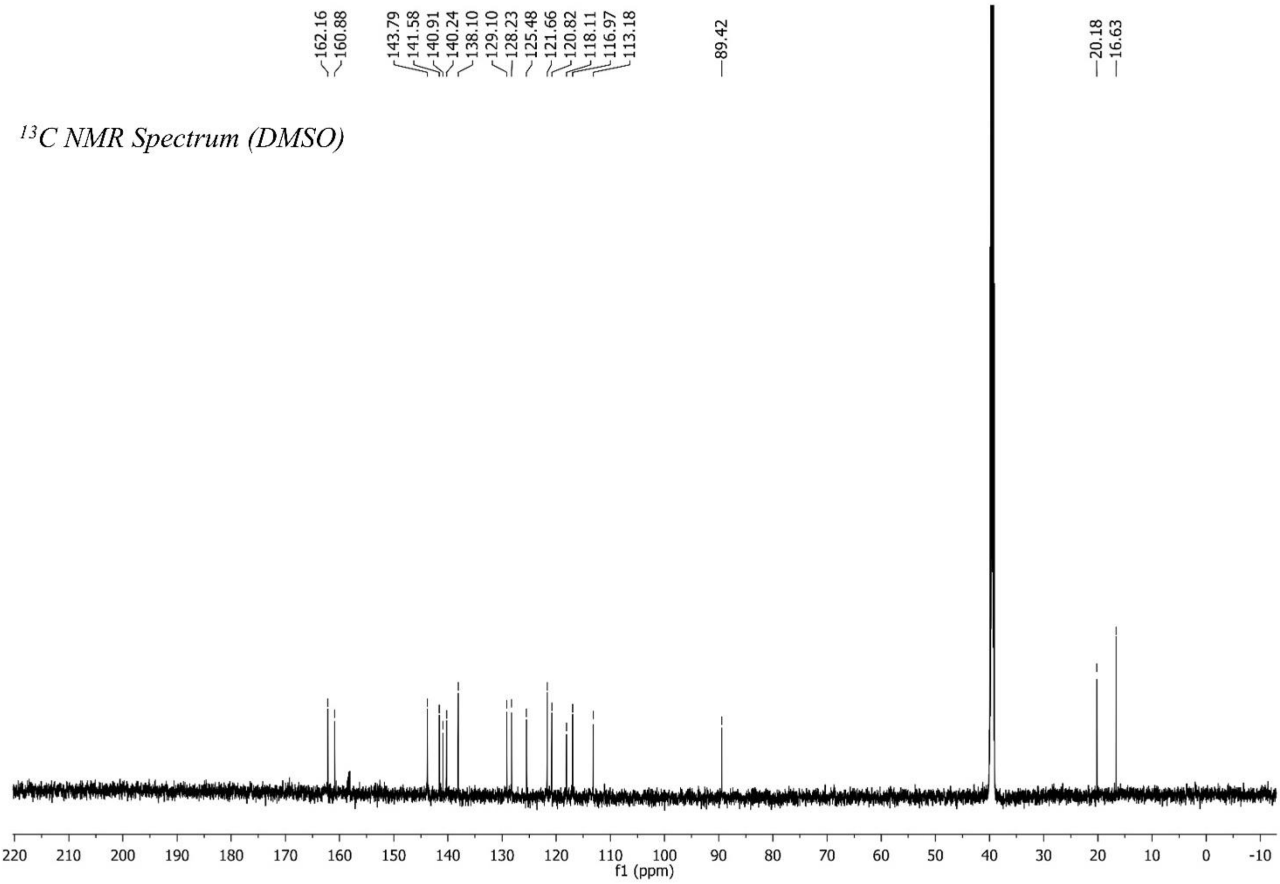

2-Hydroxy-N-(2-methyl-4-(p-tolyloxy)phenyl)pyrazolo[1,5-a]pyridine-3-carboxamide (**3**)

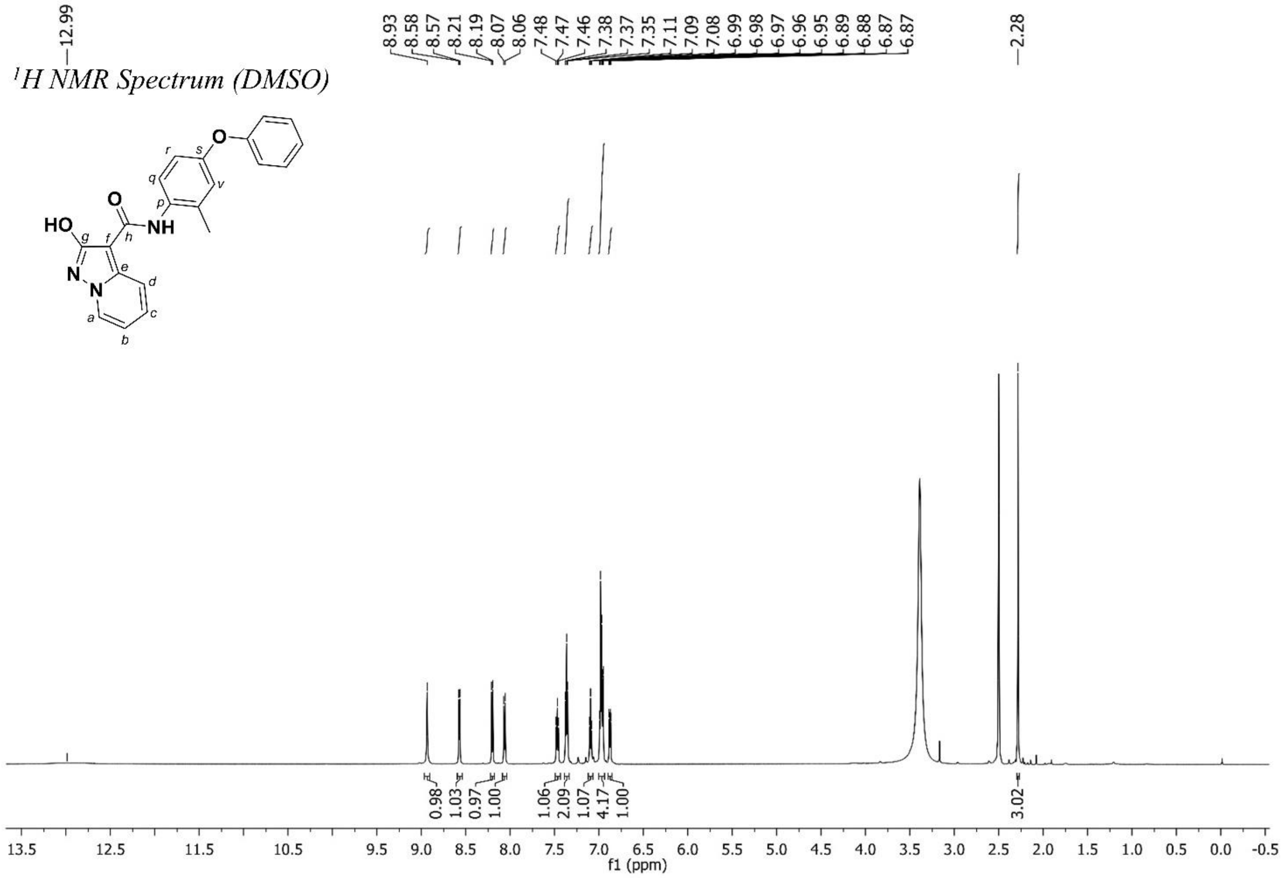

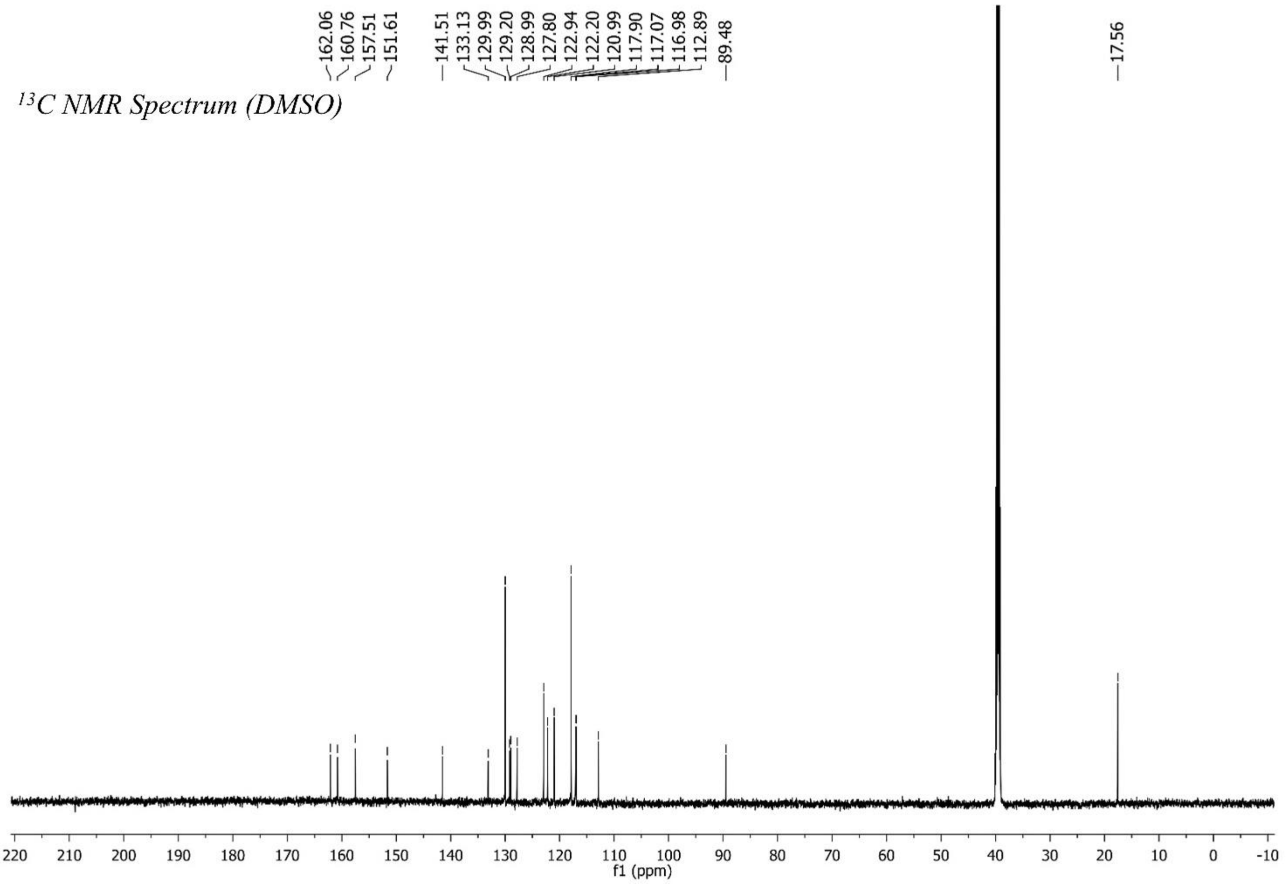

2-Hydroxy-N-(2-ispropyl-5-methyl-4-phenoxyphenyl)pyrazolo[1,5-a]pyridine-3-carboxamide (**4**)

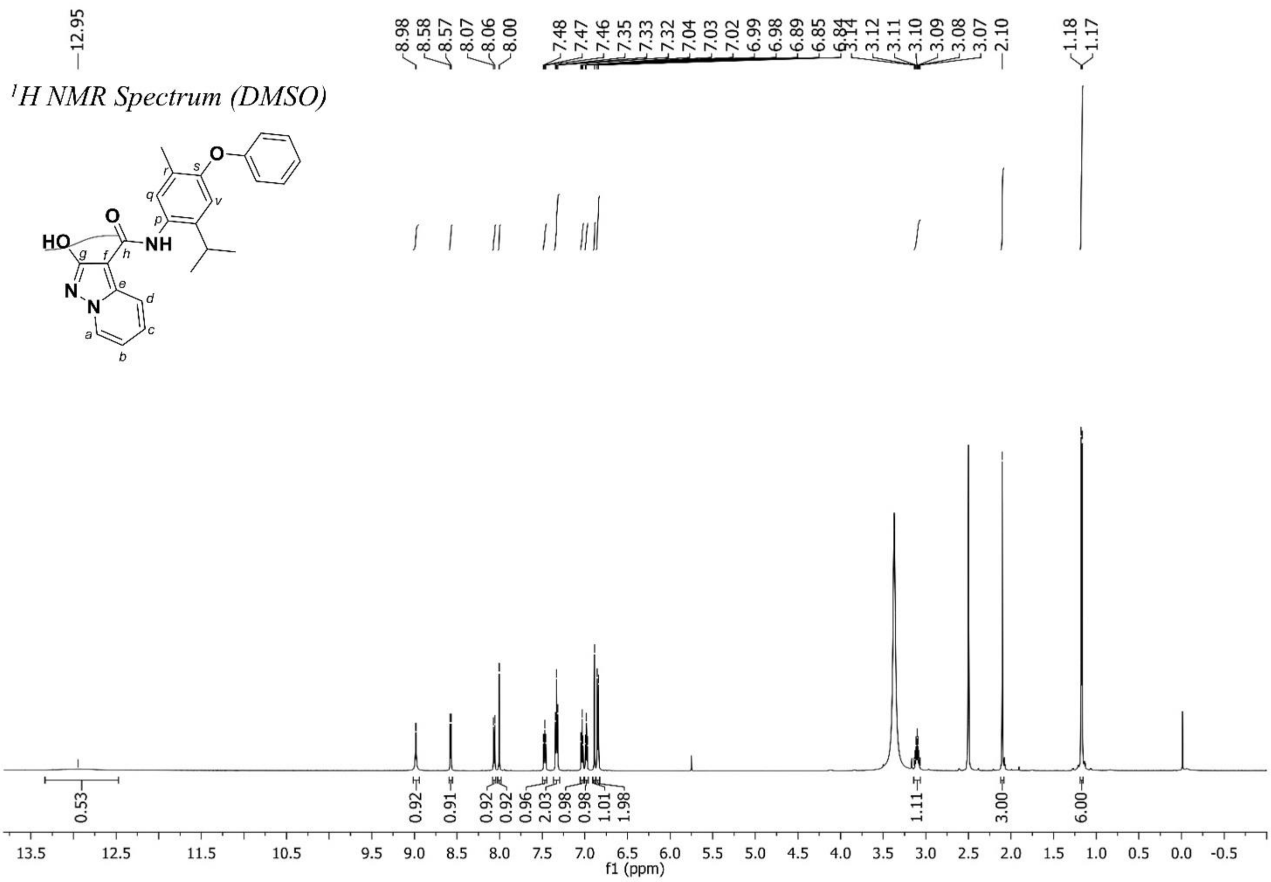

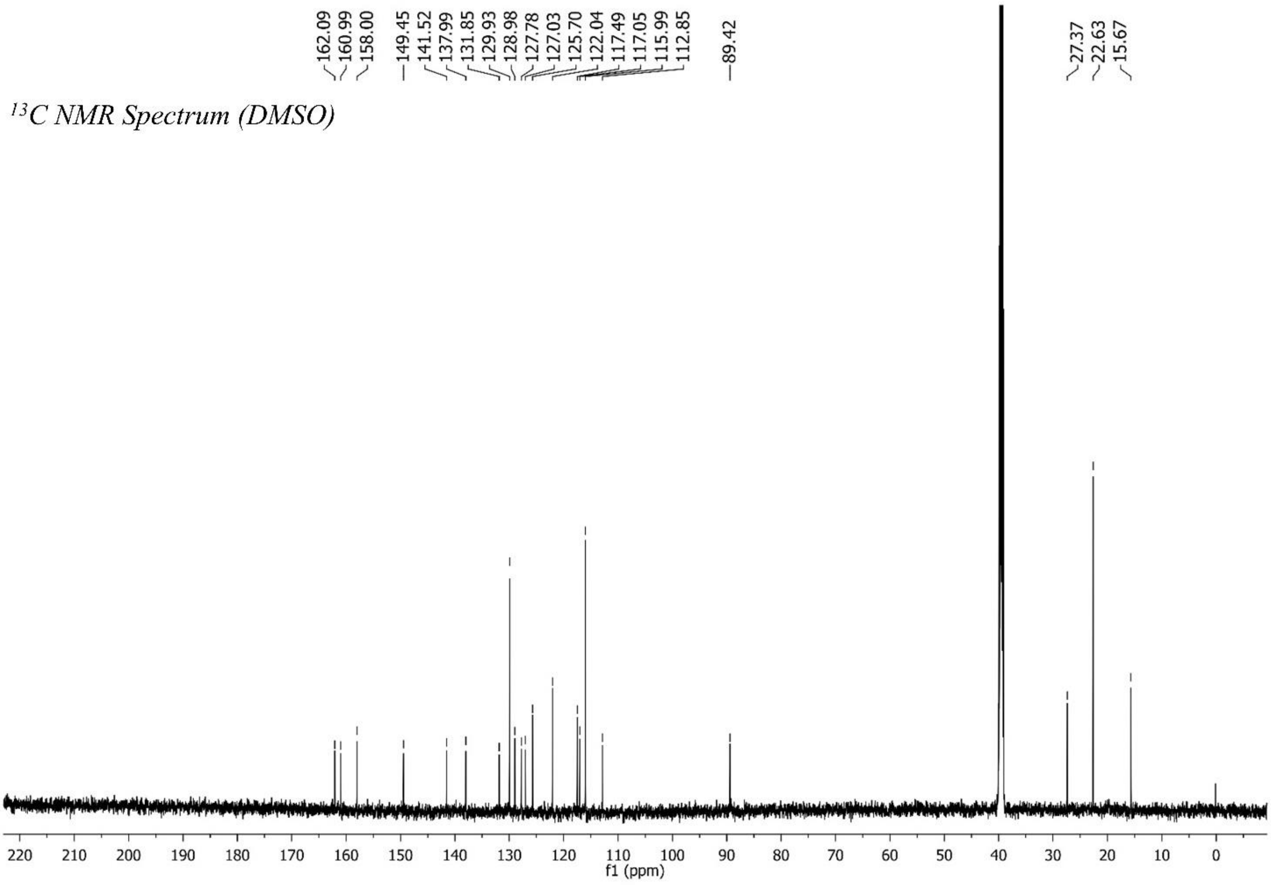

2-Hydroxy-N-(5-isopropyl-2-methyl-4-phenoxyphenyl)pyrazolo[1,5-a]pyridine-3-carboxamide (**5**)

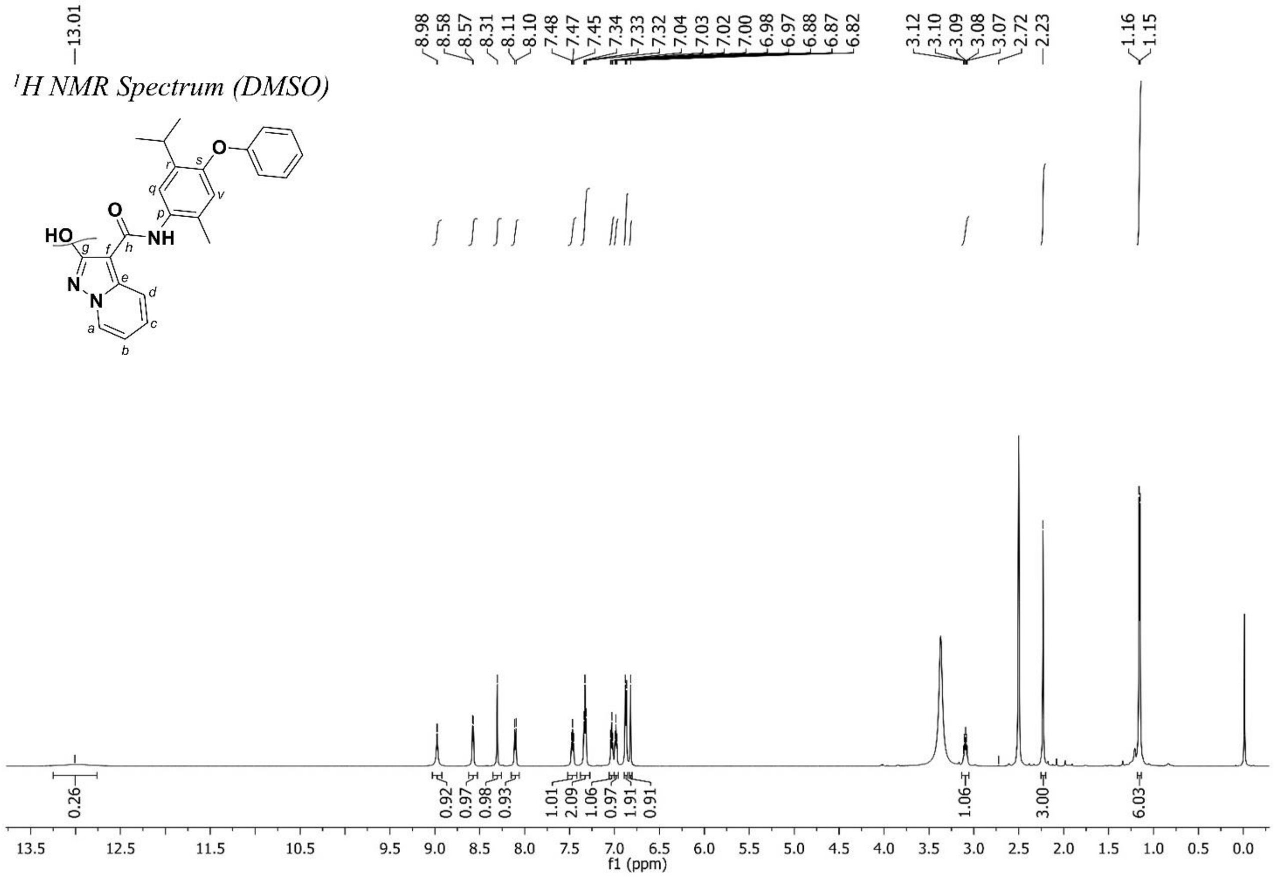

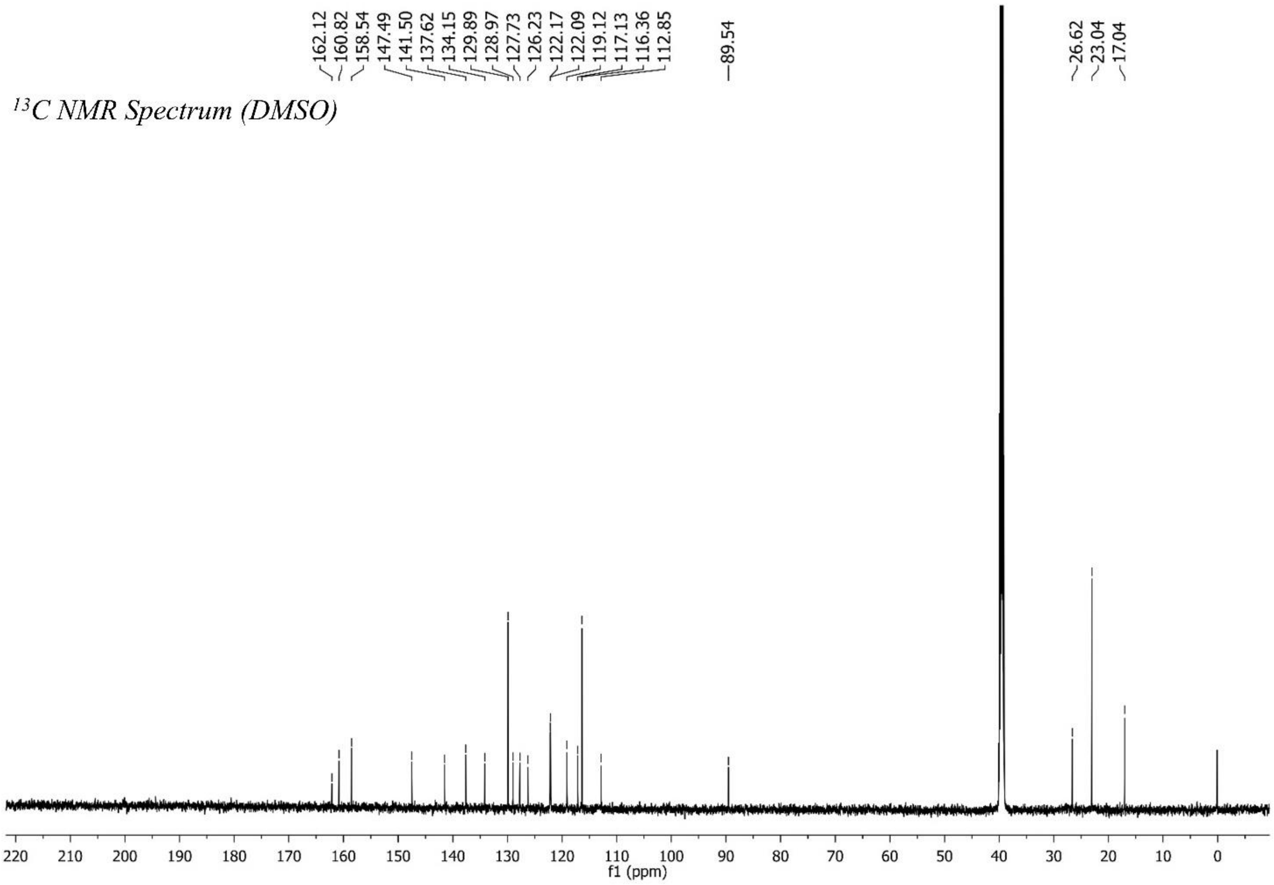

2-Hydroxy-N-(2-isopropoxy-5-methyl-4-phenoxyphenyl)pyrazolo[1,5-a]pyridine-3-carboxamide (**6**)

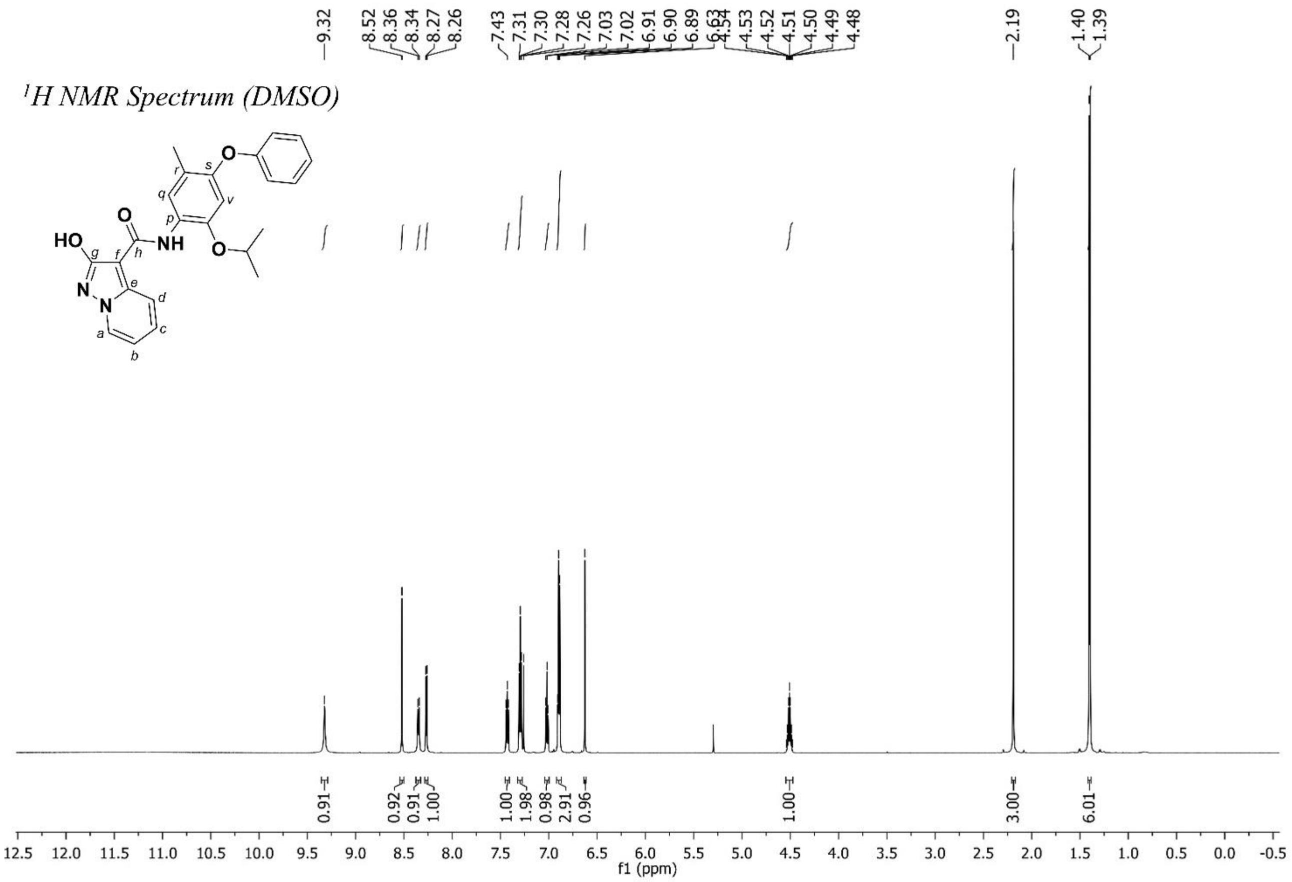

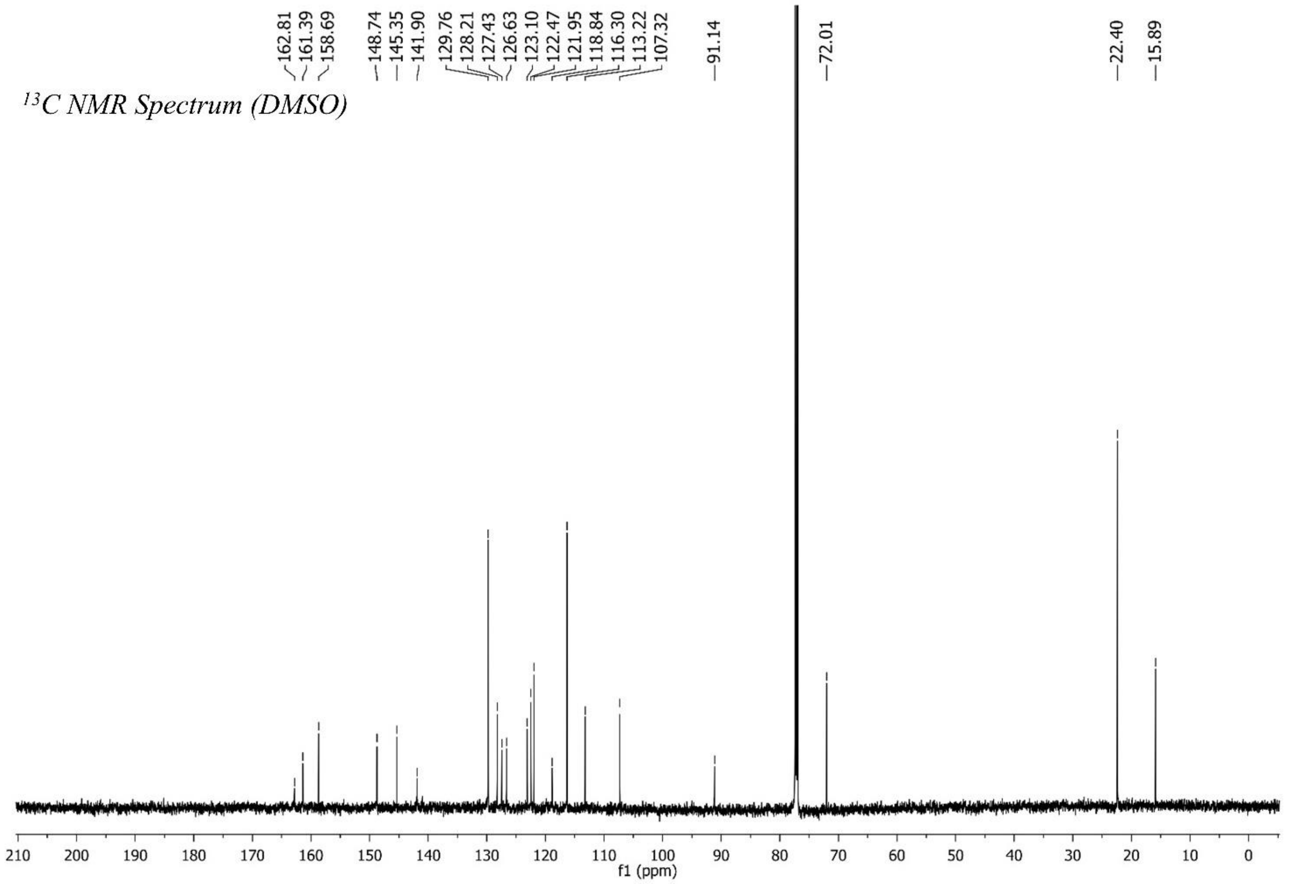

N-(2-cyclobutoxy-5-methyl-4-phenoxyphenyl)-2-hydroxypyrazolo[1,5-a]pyridine-3-carboxamide (**7**)

^{1}H NMR Spectrum (DMSO)

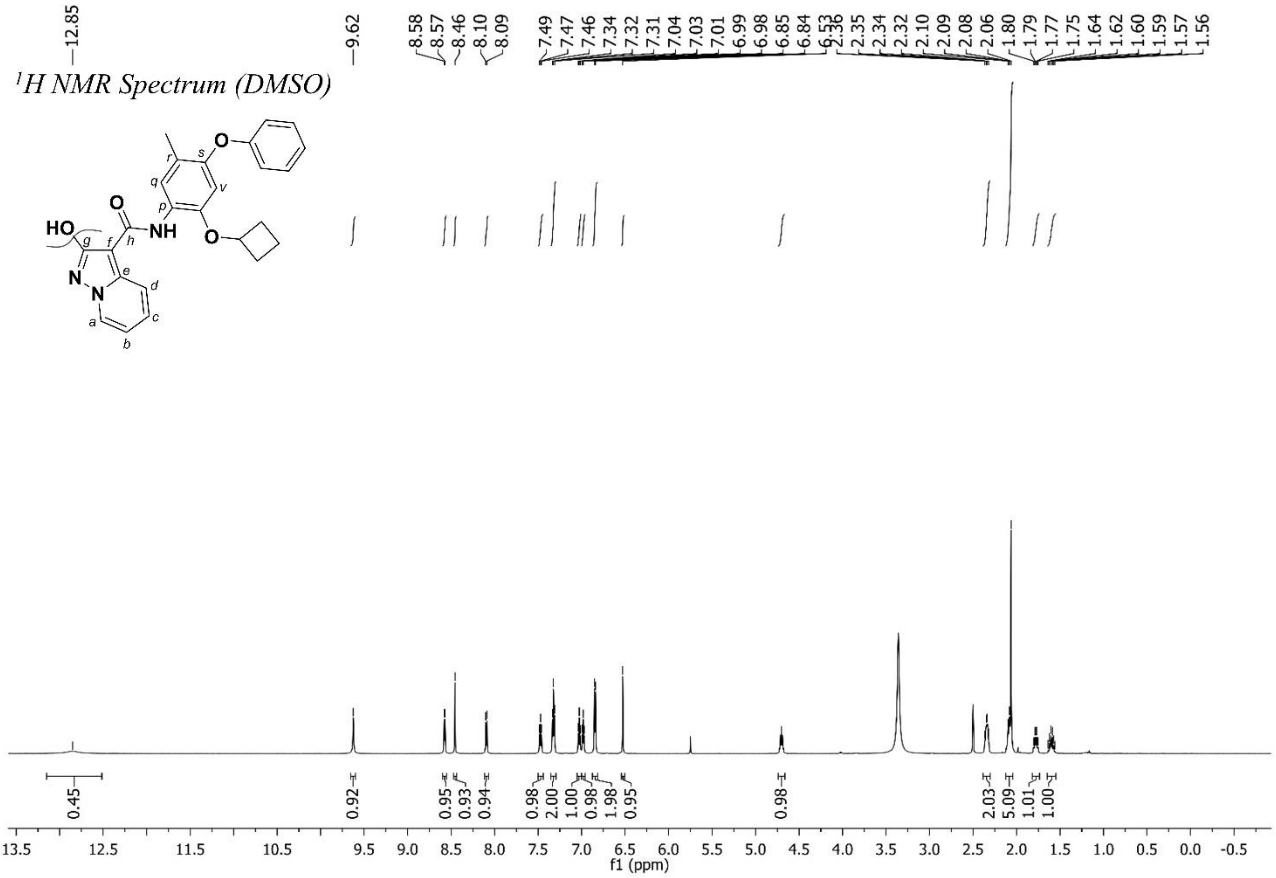

^{13}C NMR Spectrum (DMSO)

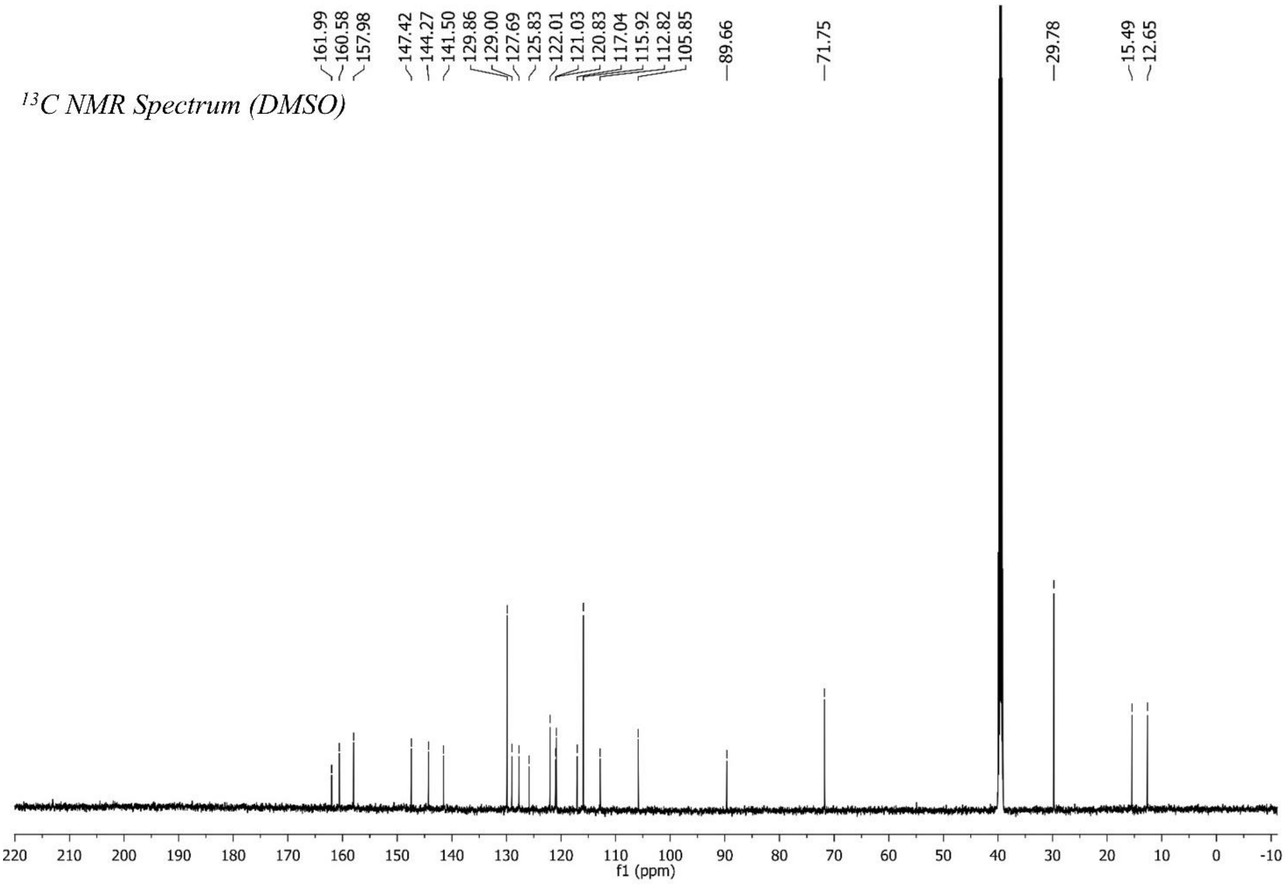

N-(2-cyclobutoxy-5-methyl-4-phenoxyphenyl)-2-hydroxypyrazolo[1,5-a]pyridine-3-carboxamide (**7**)

^{13}C-Dept NMR Spectrum (DMSO)

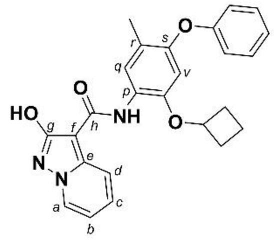

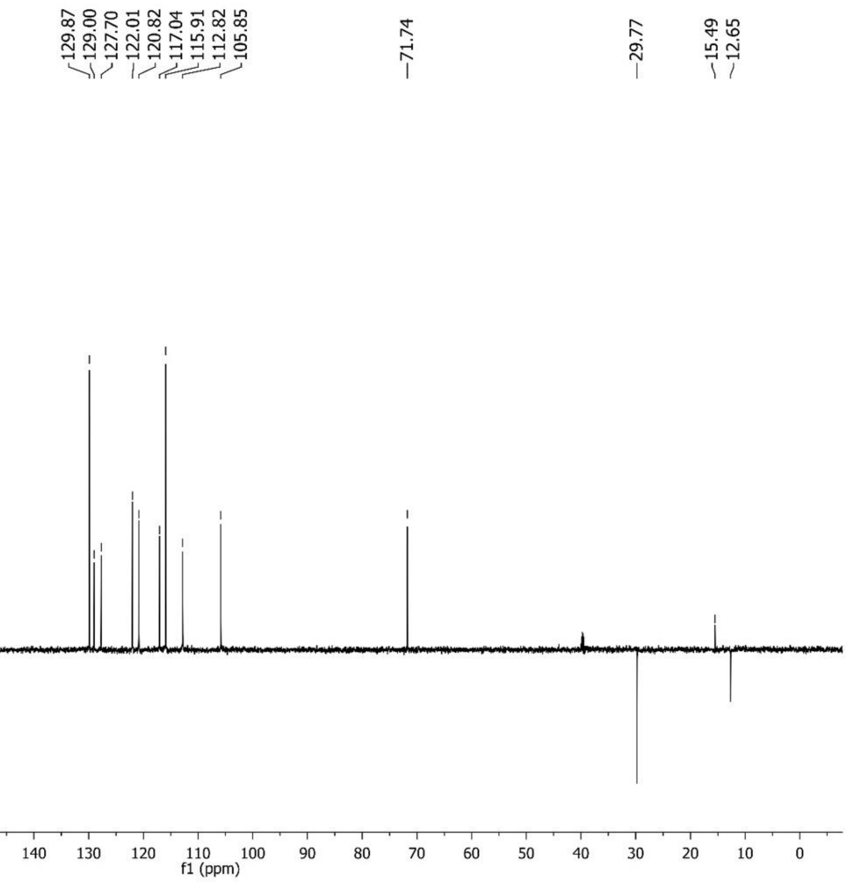

N-(2-(Sec-butoxy)-5-methyl-4-phenoxyphenyl)-2-hydroxypyrazolo[1,5-a]pyridine-3-carboxamide (**8**)

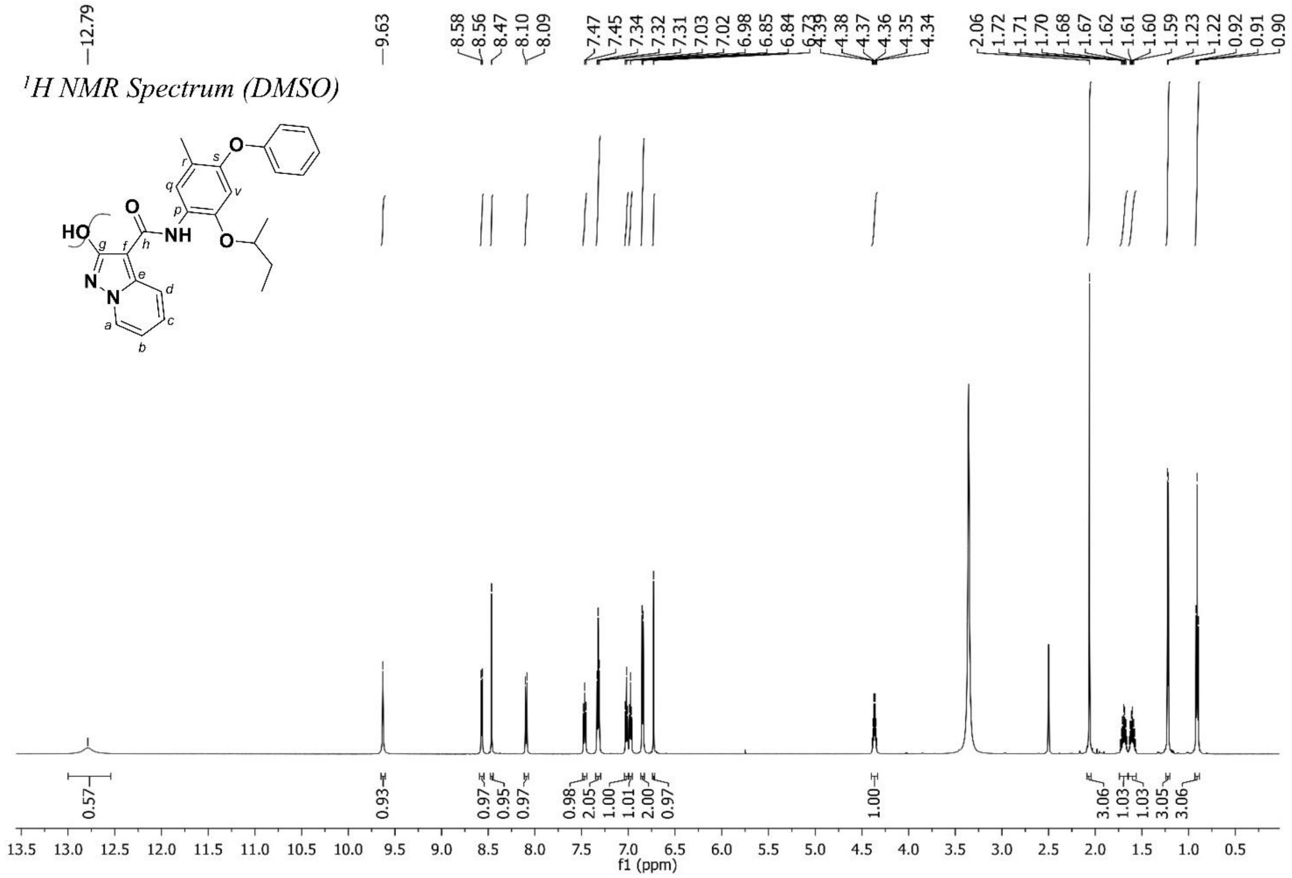

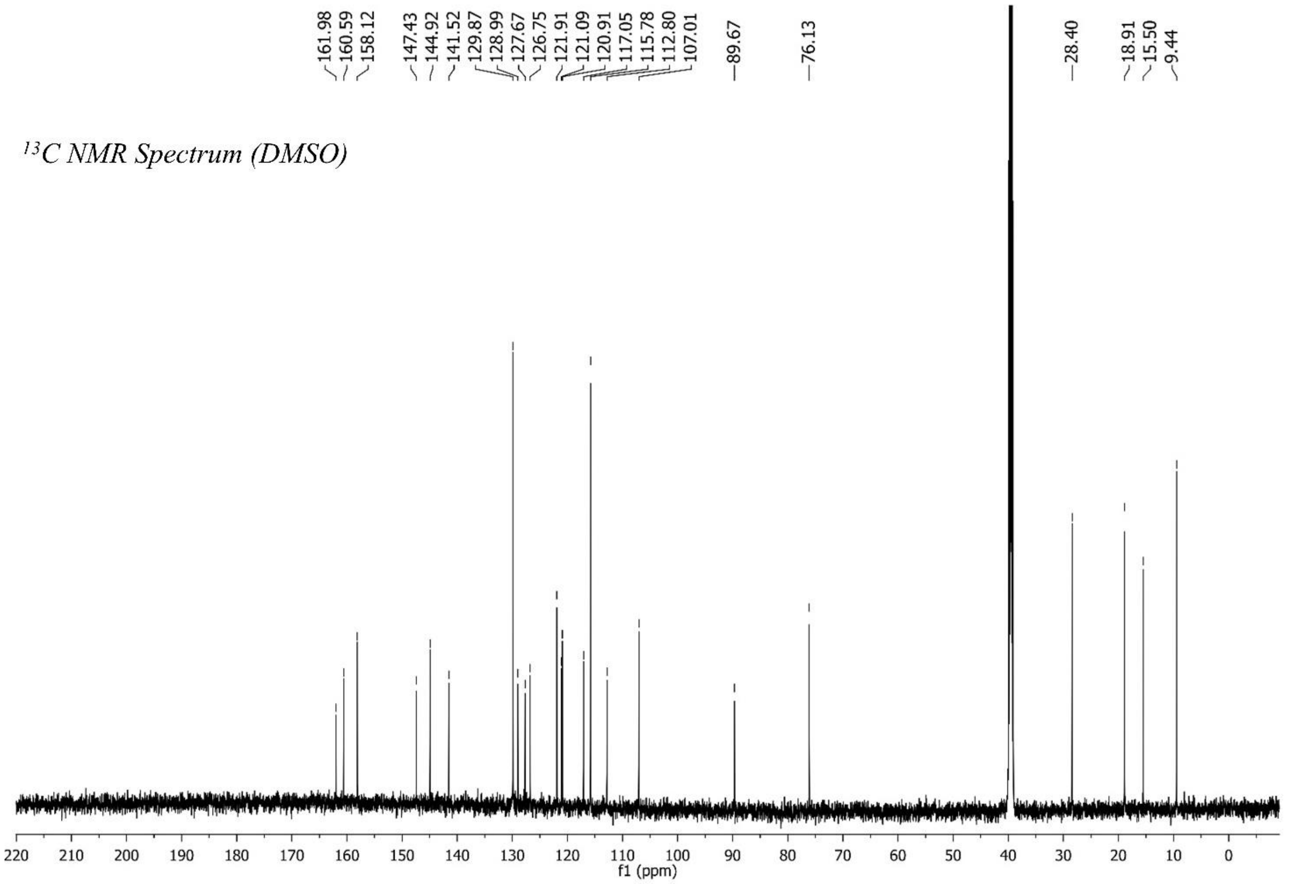

2-Hydroxy-N-(5-methyl-2-(pentan-3-yloxy)-4-phenoxyphenyl)pyrazolo[1,5-a]pyridine-3-carboxamide (**9**)

^{1}H NMR Spectrum (CDCl$_3$)

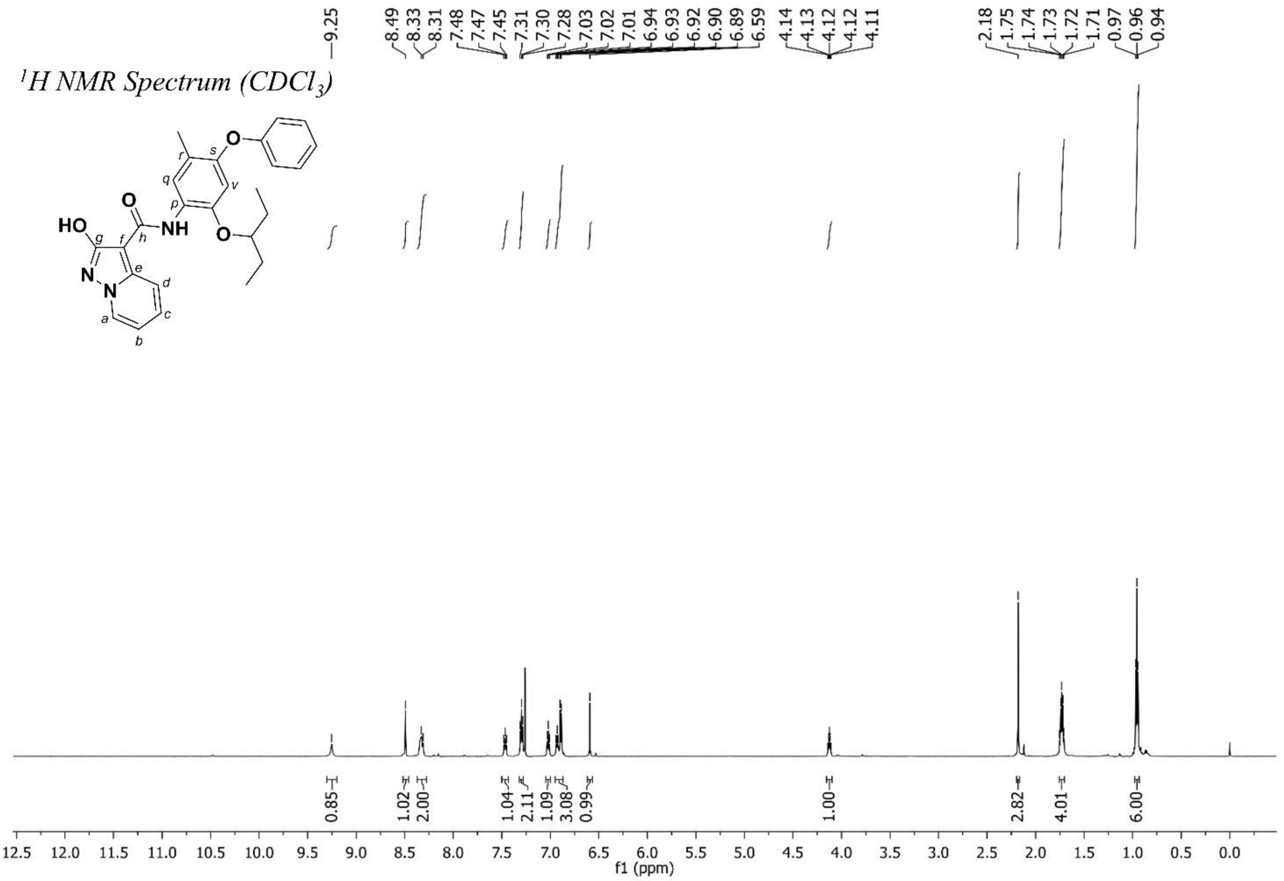

^{13}C NMR Spectrum (CDCl$_3$)

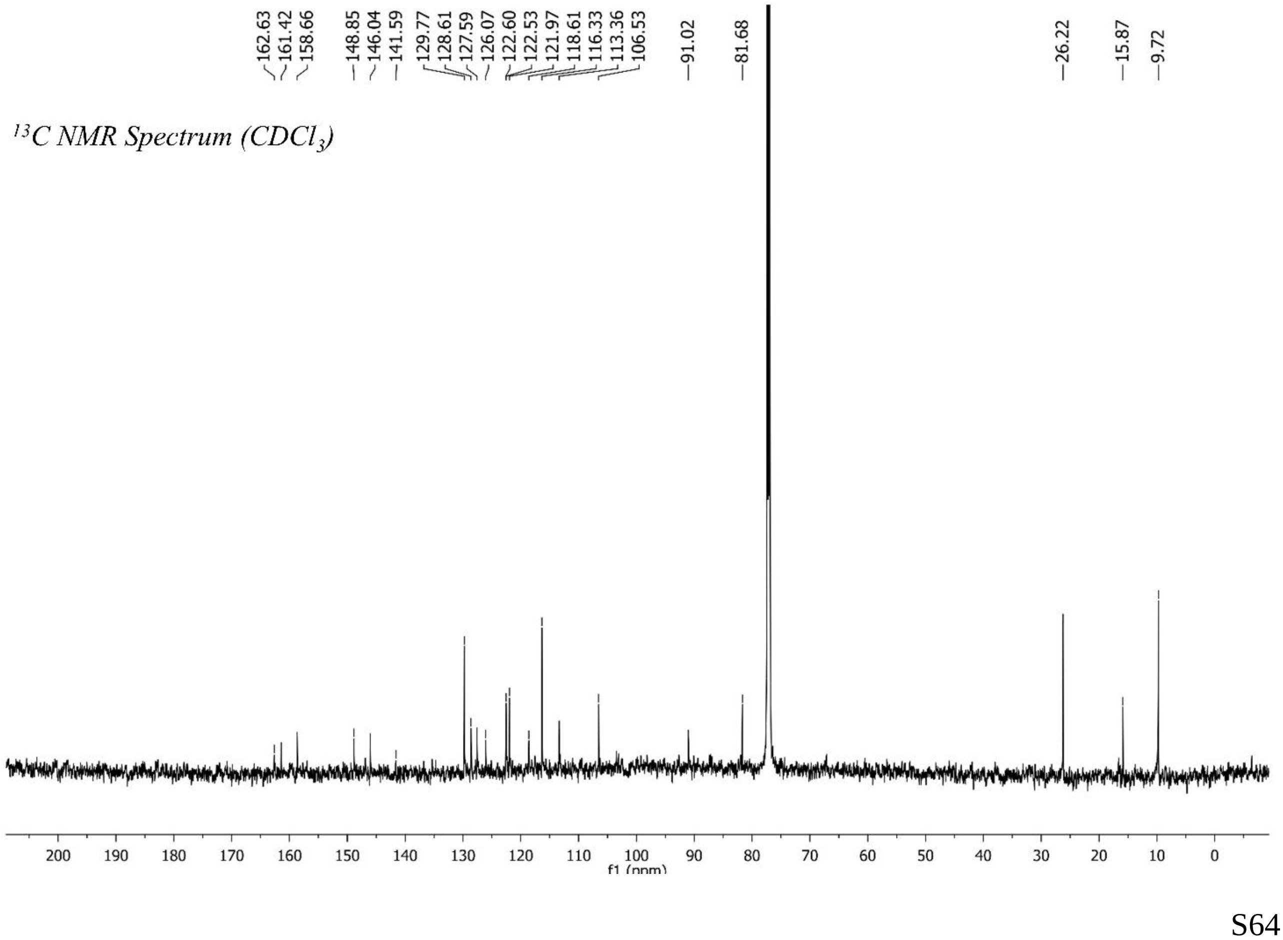

2-Hydroxy-N-(5-methyl-2-(pentan-2-yloxy)-4-phenoxyphenyl)pyrazolo[1,5-a]pyridine-3-carboxamide (**10**)

^{1}H NMR Spectrum (DMSO)

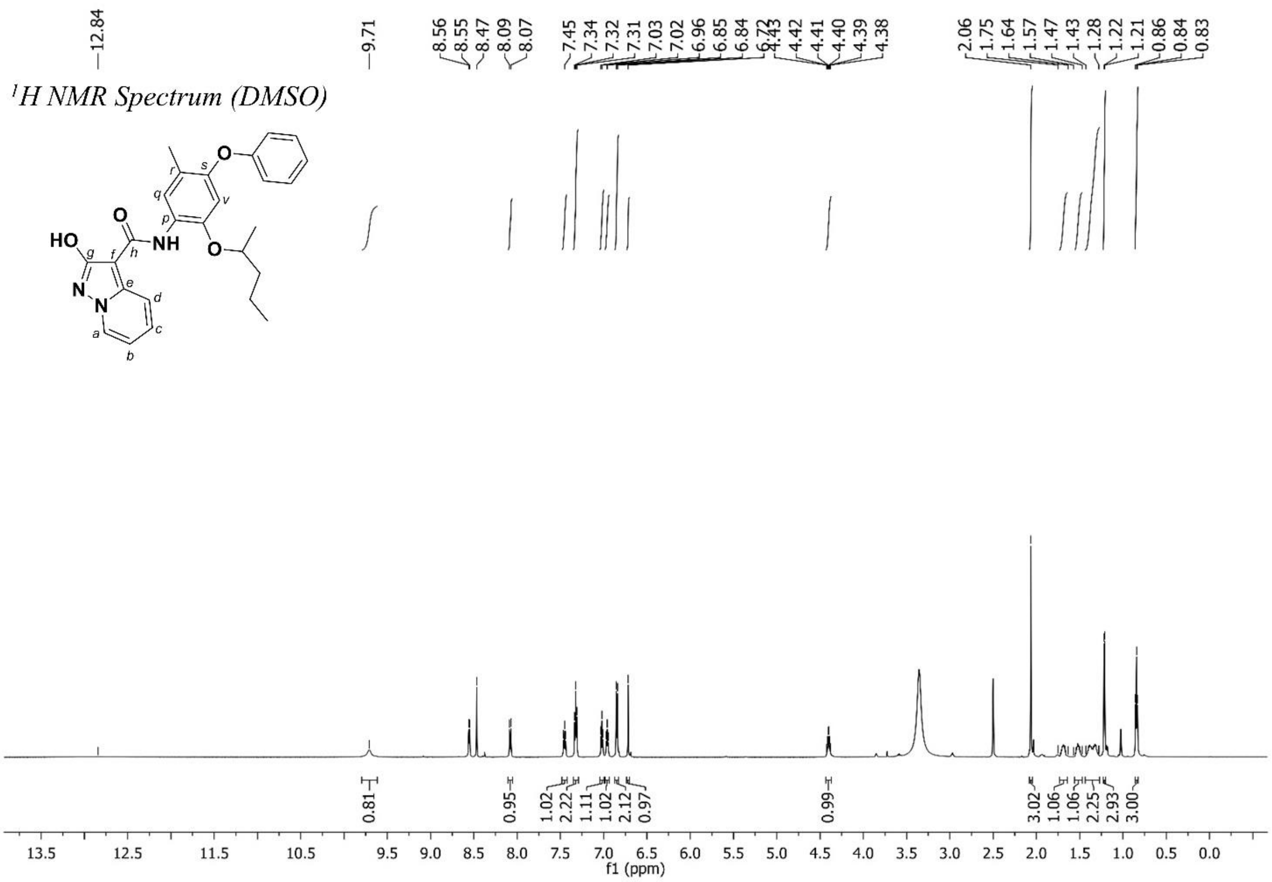

^{13}C NMR Spectrum (DMSO)

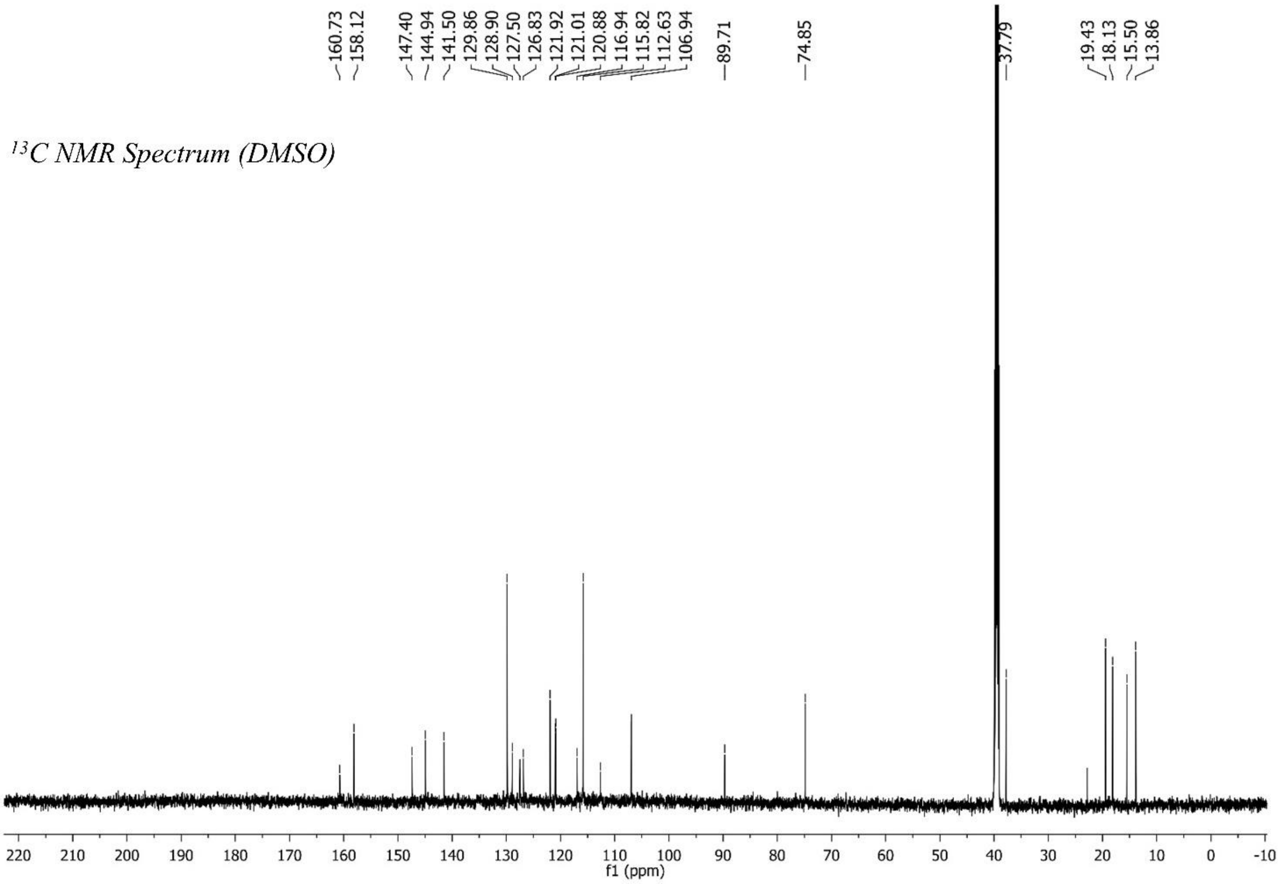

2-Hydroxy-N-(2-isopropyl-5-methyl-4-(pyridin-4-yloxy)phenyl)pyrazolo[1,5-a]pyridine-3-carboxamide (**18**)

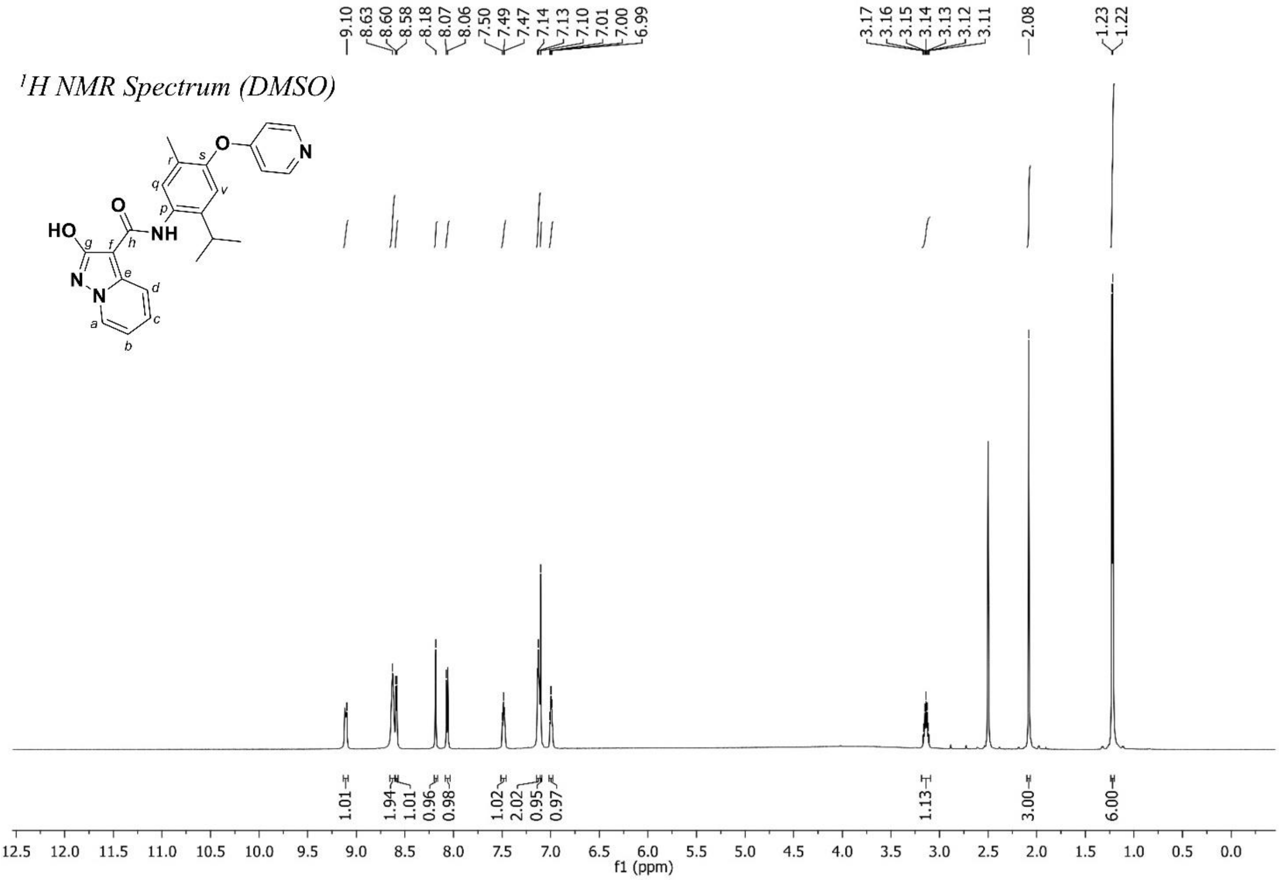

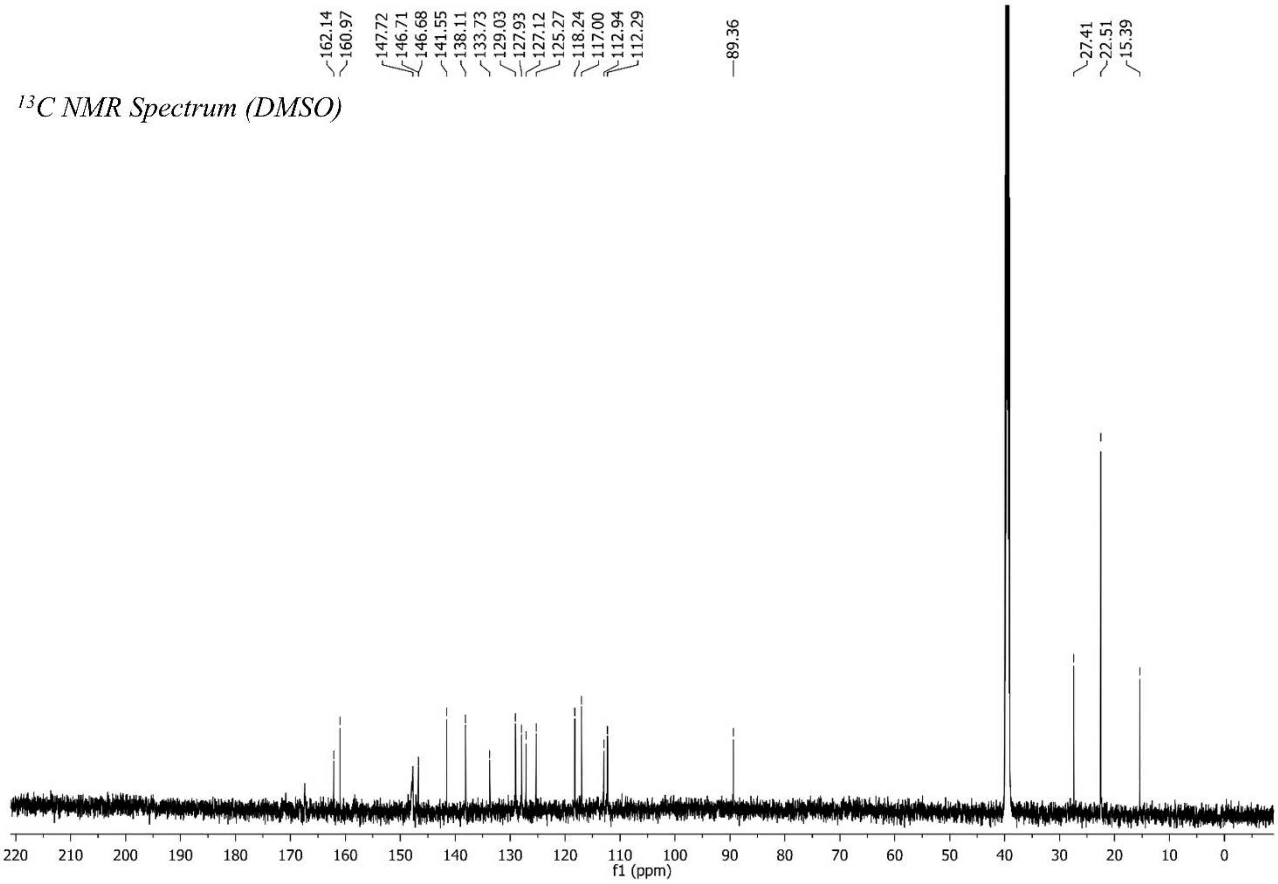

2-Hydroxy-N-(2-isopropyl-5-methyl-4-(4-(trifluoromethyl)phenoxy)phenyl)pyrazolo[1,5-a]pyridine-3-carboxamide (**19**)

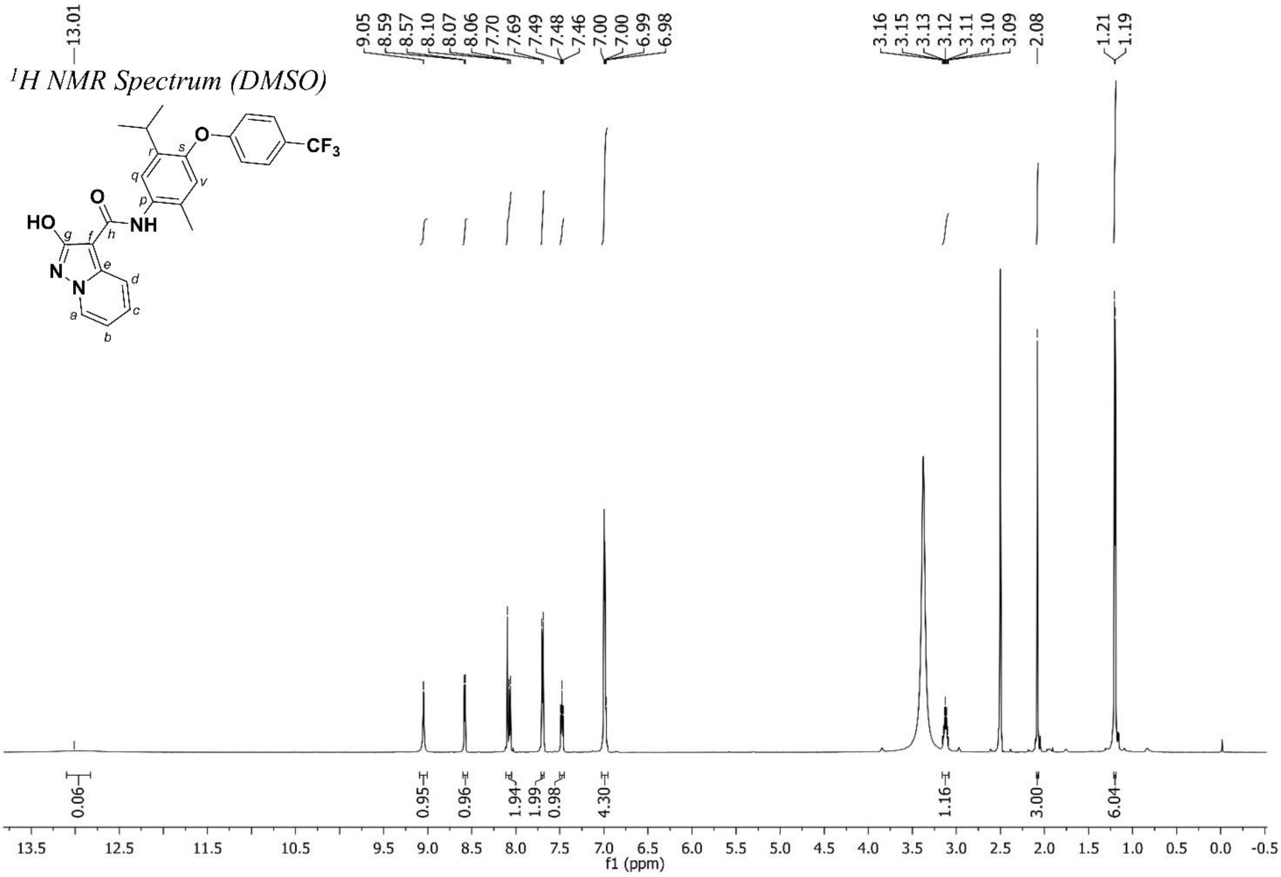

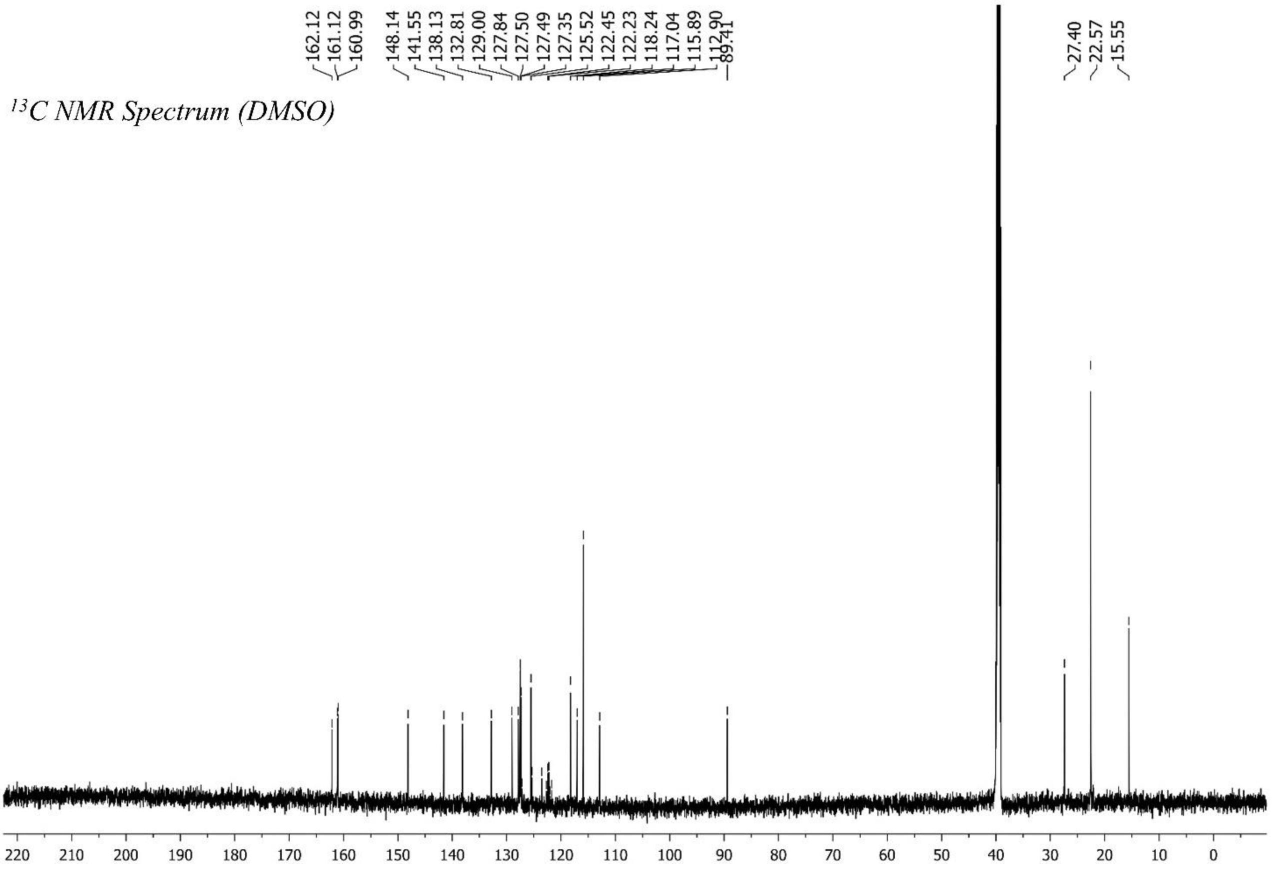

2-Methyl-1-nitro-4-phenoxybenzene (**58**)

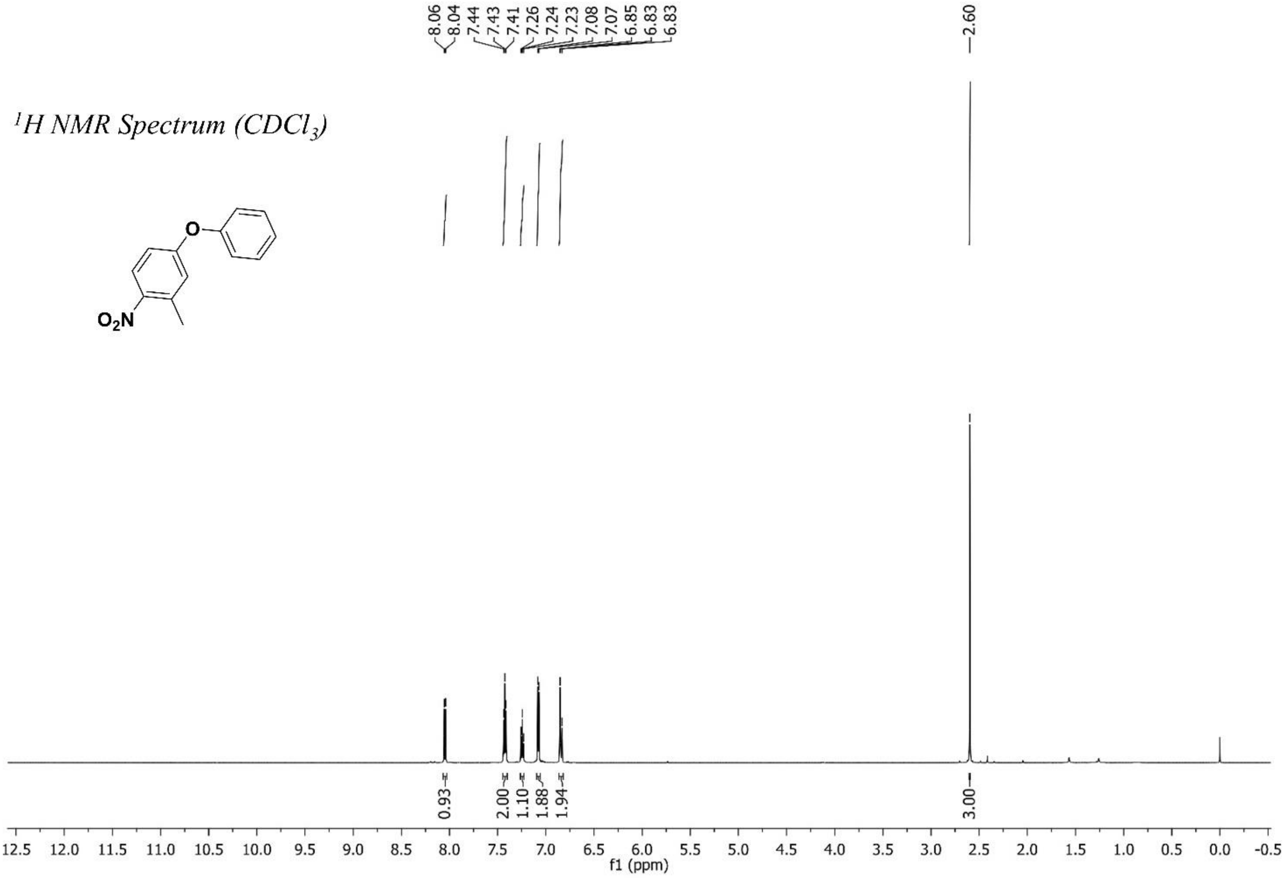

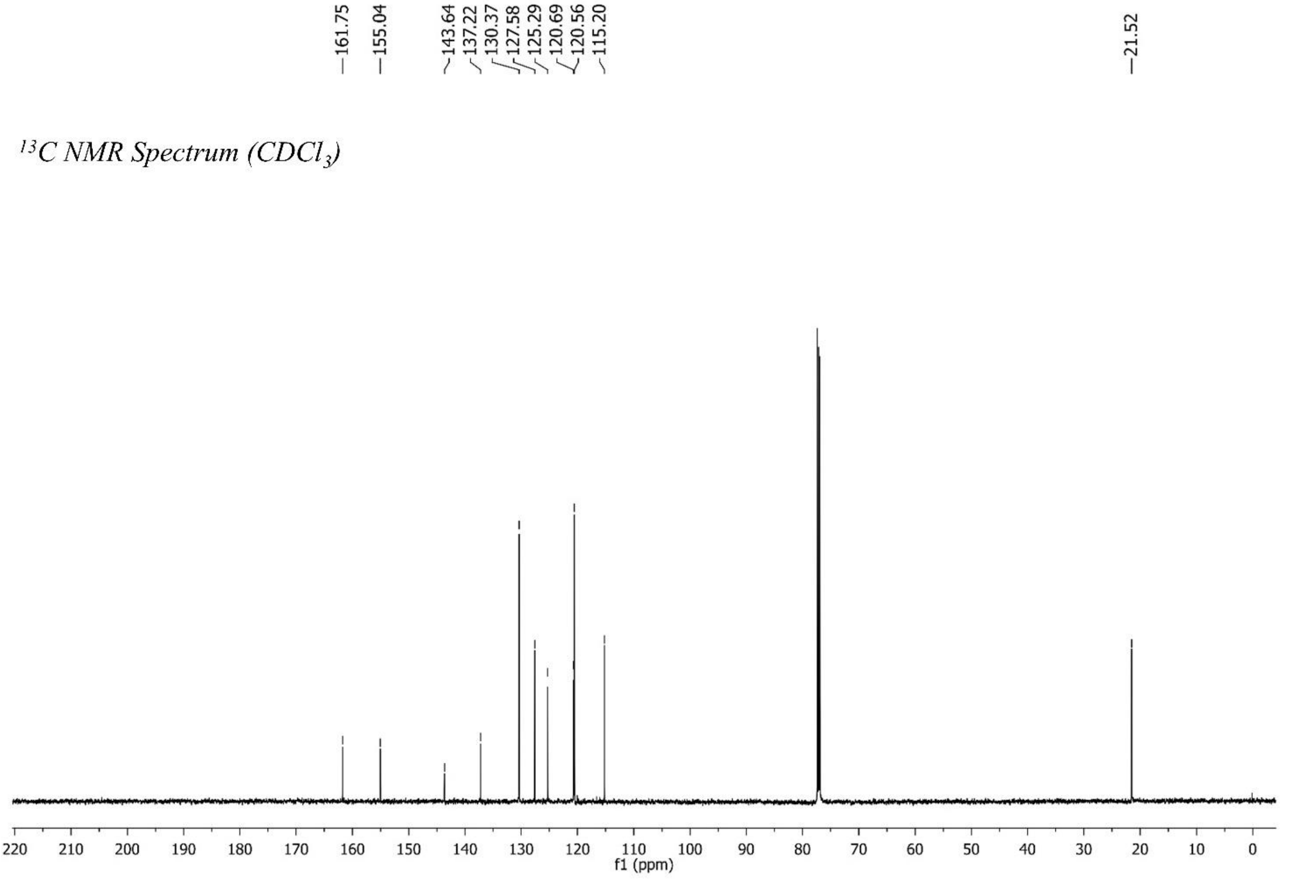

4-((2,5-Dimethyl-4-nitrophenyl)thio)pyridine (**59**)

^{1}H NMR Spectrum (CDCl$_3$)

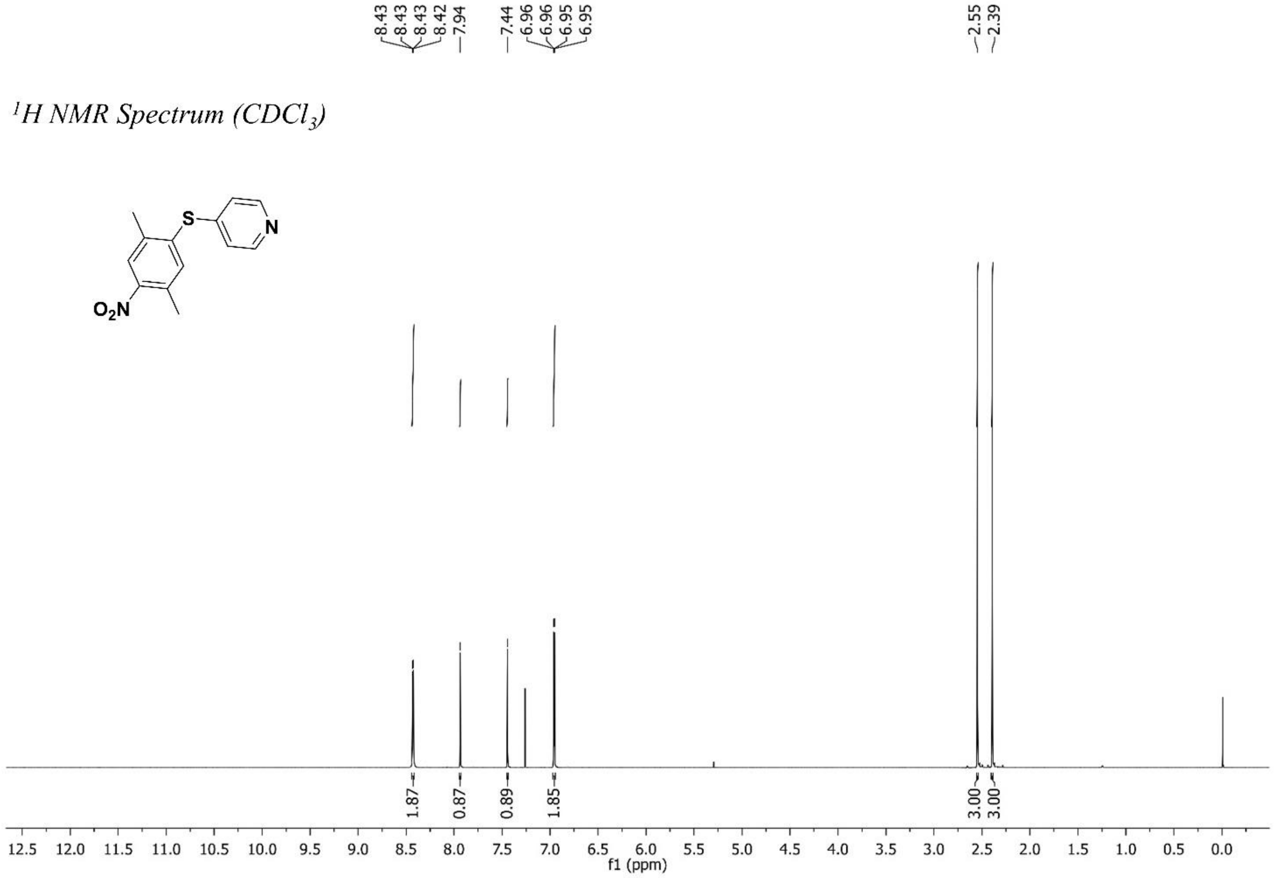

^{13}C NMR Spectrum (CDCl$_3$)

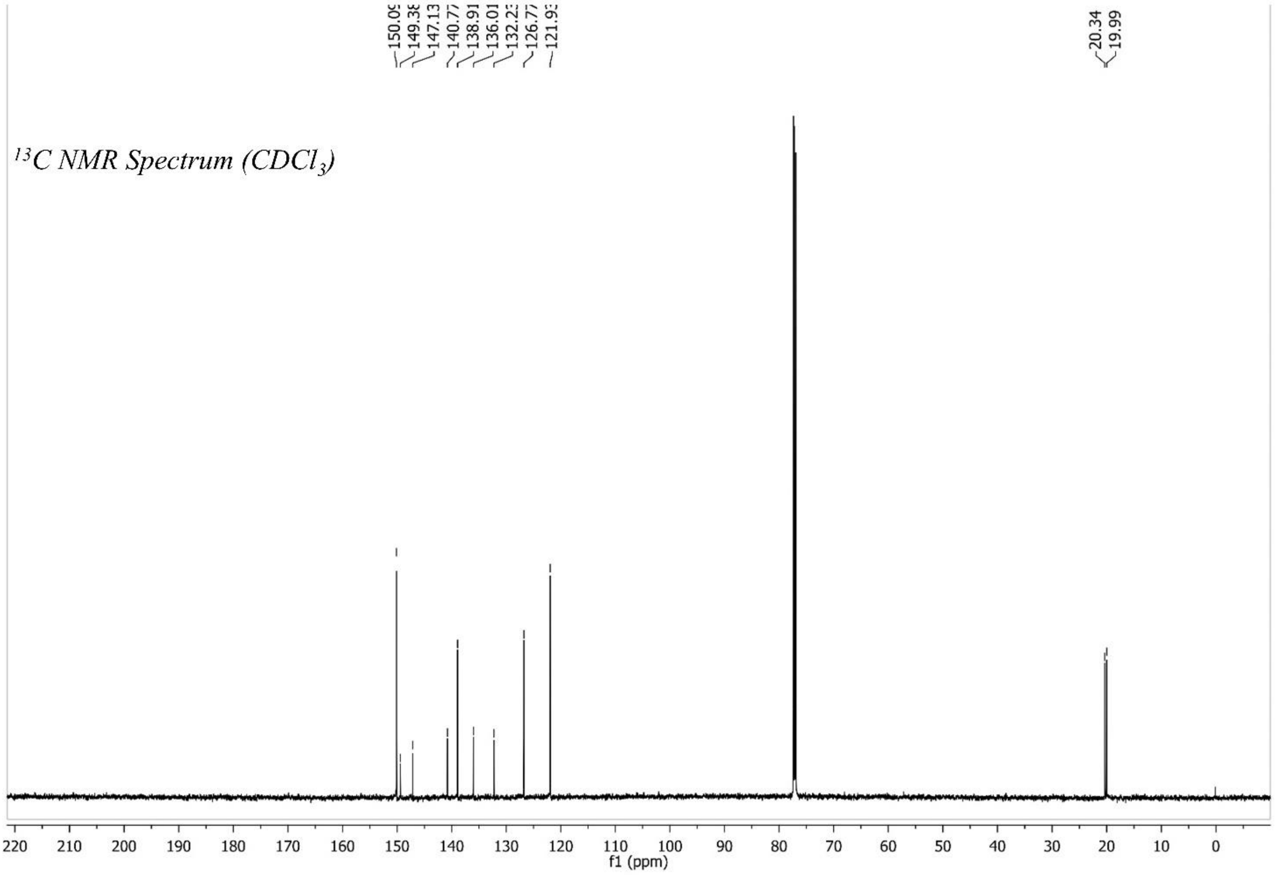

2-Methyl-4-phenoxyaniline (**39**)

^{1}H NMR Spectrum ($CDCl_3$)

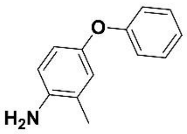

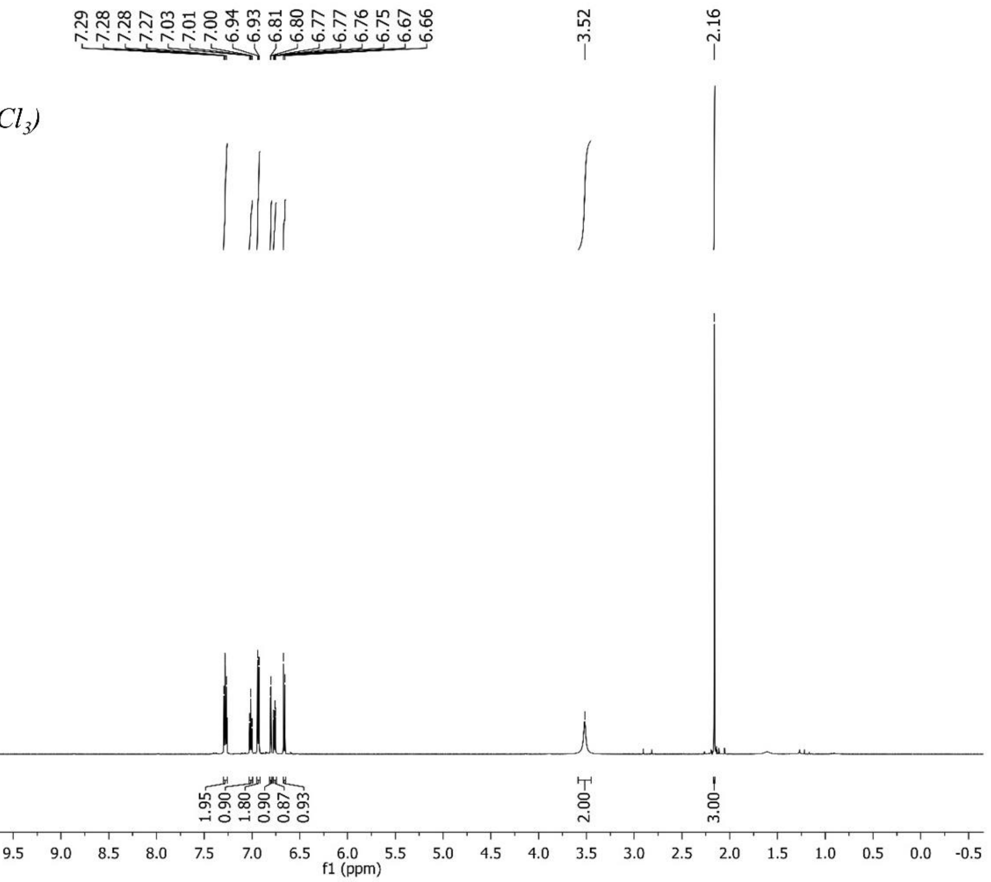

^{13}C NMR Spectrum ($CDCl_3$)

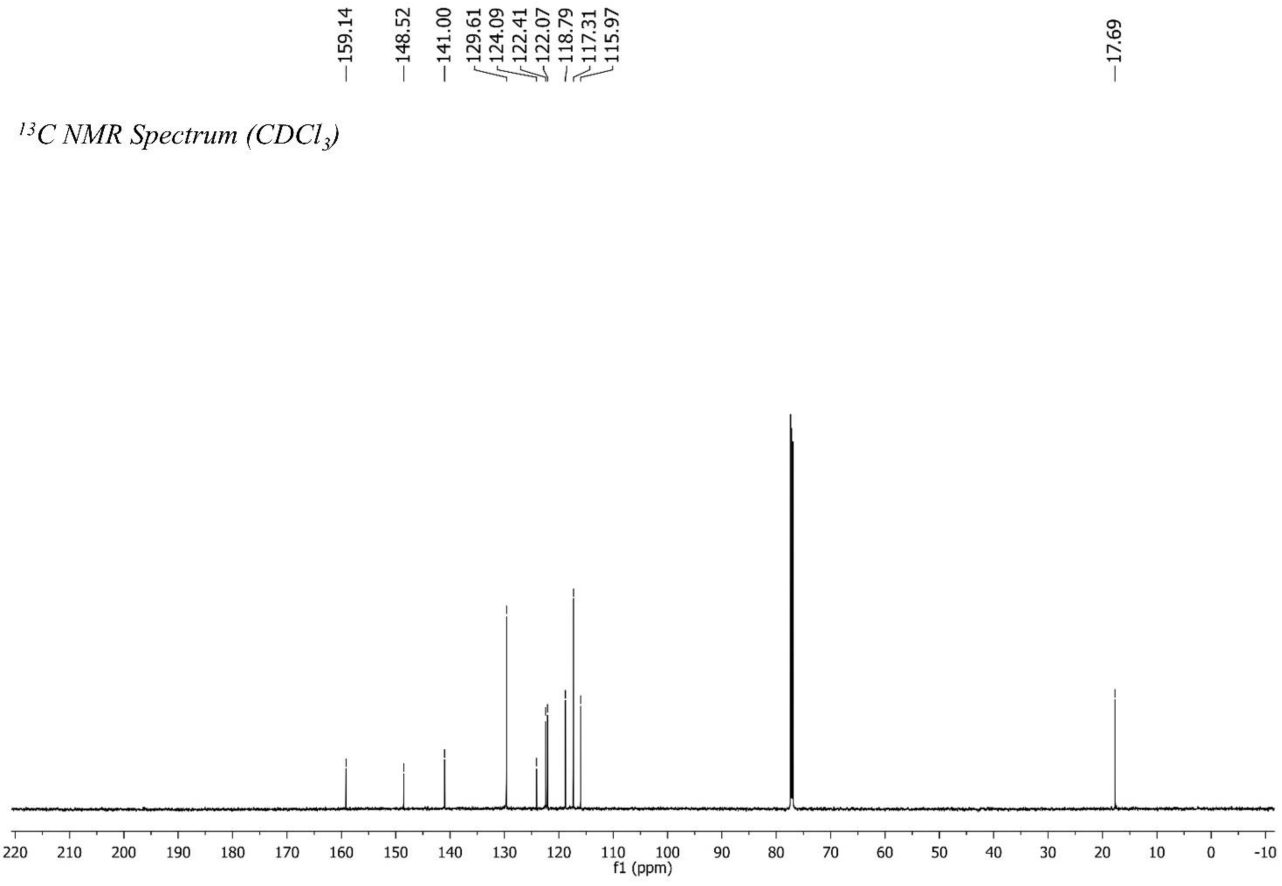

2,5-Dimethyl-4-(pyridin-4-ylthio)aniline (**54**)

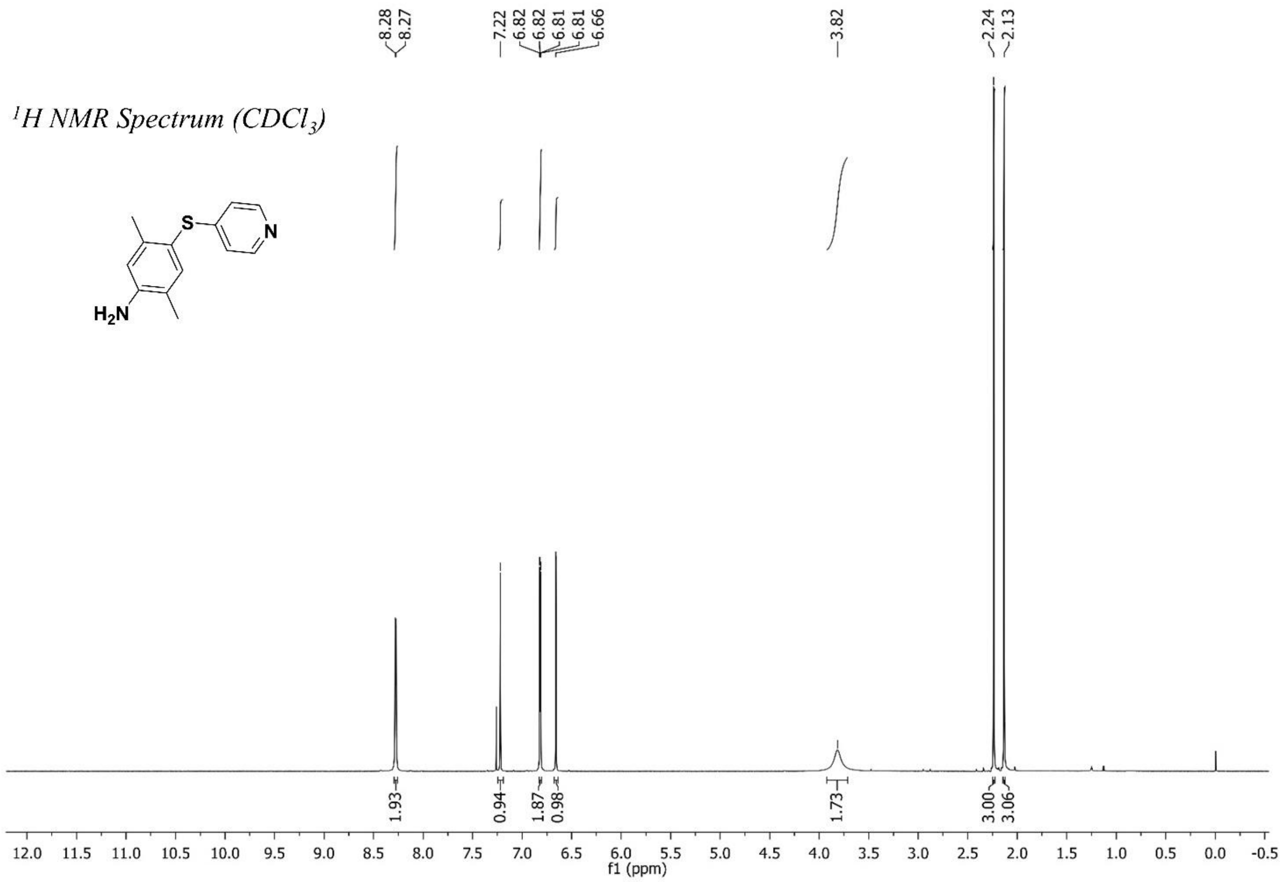

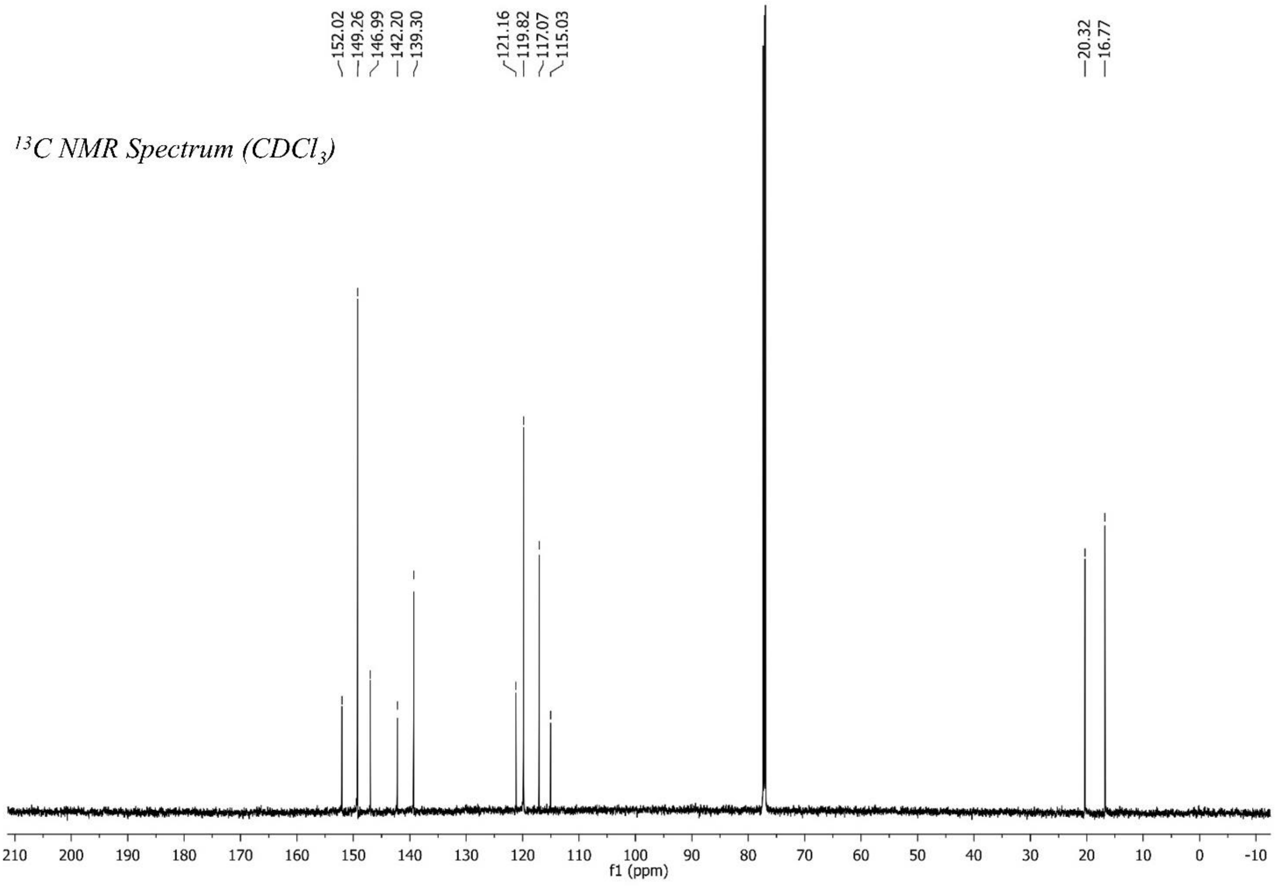

2-Nitro-5-phenoxypyridine (**61**)

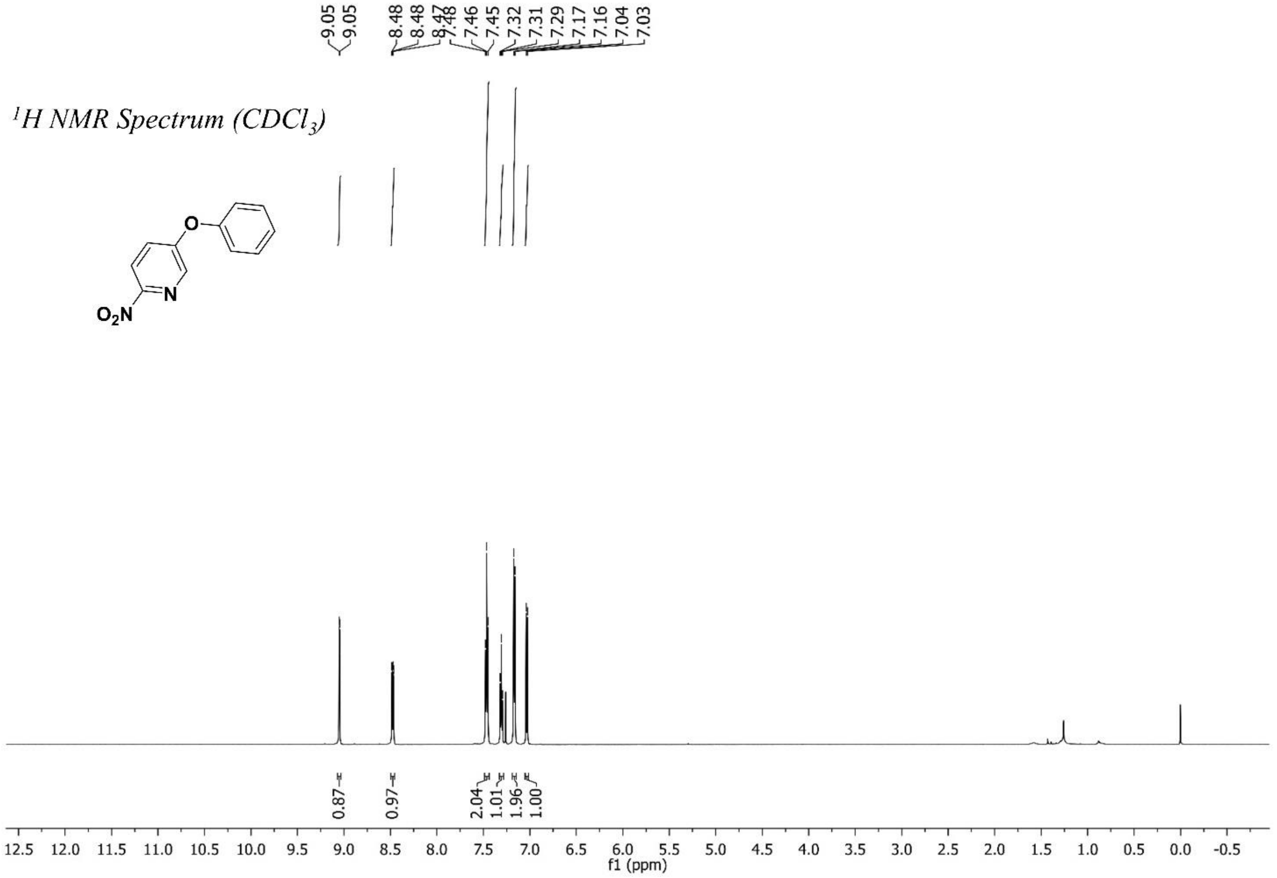

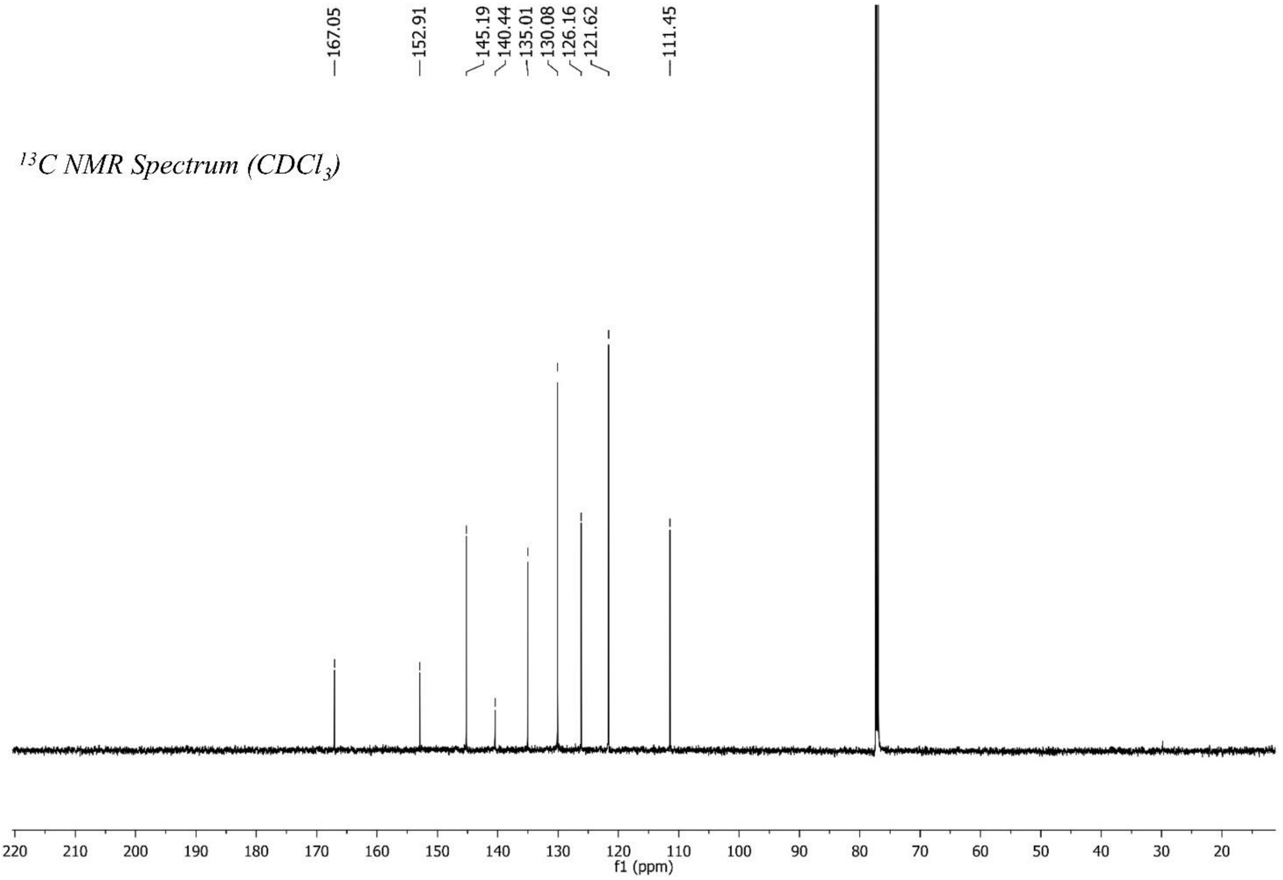

2-Nitro-5-[3-(trifluoromethyl)phenoxy]pyridine (**62**)

^{1}H NMR Spectrum (DMSO)

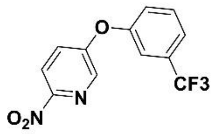

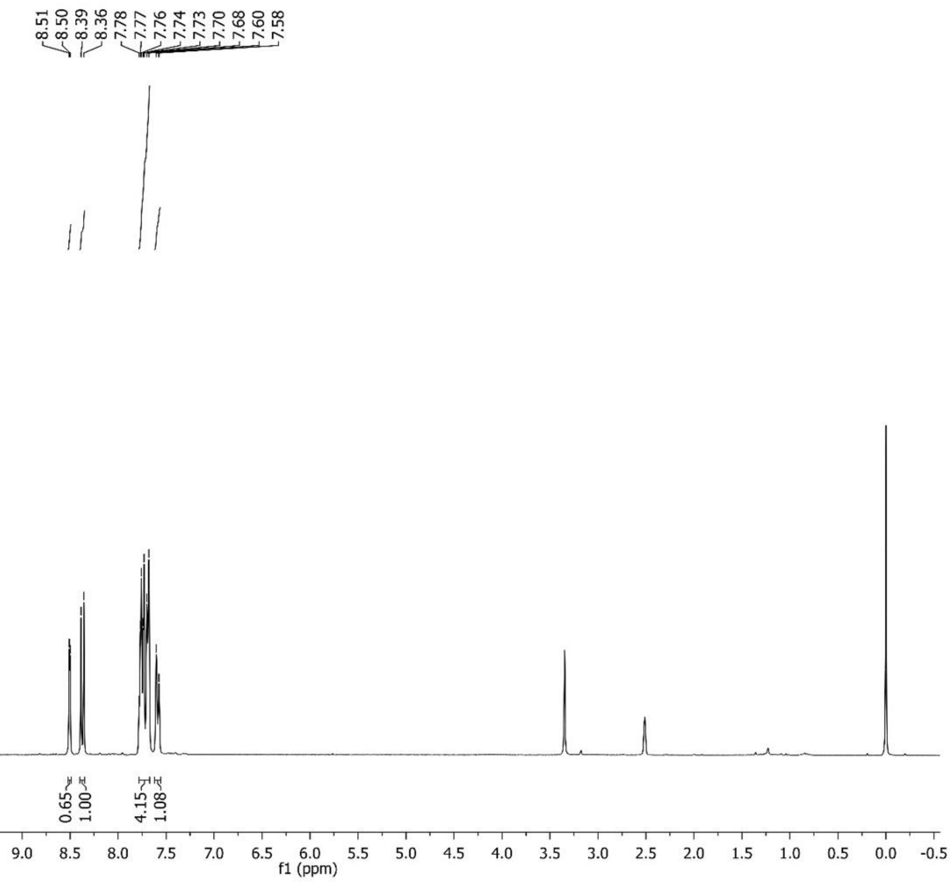

^{13}C NMR Spectrum (DMSO)

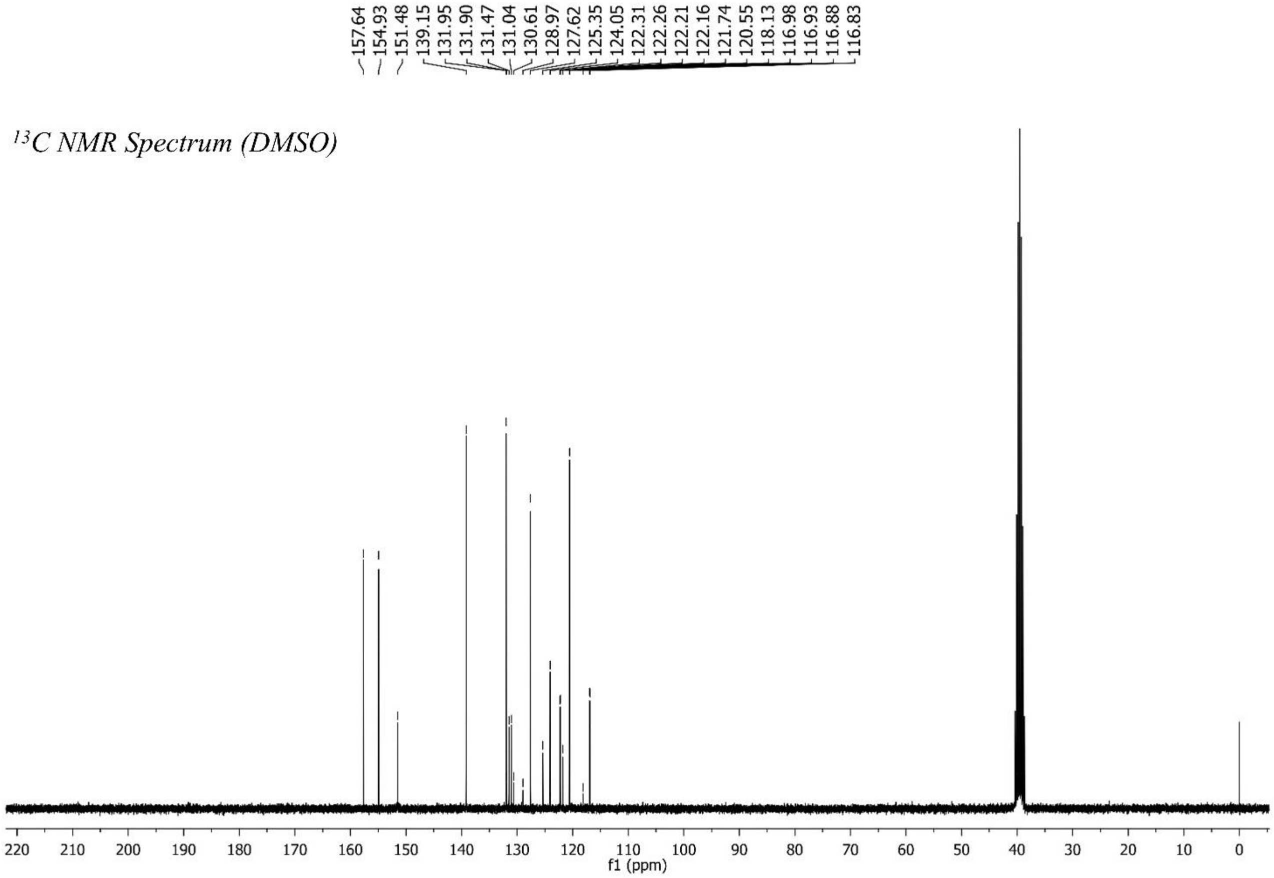

5-Phenoxypyridin-2-amine (**42**)

^{1}H NMR Spectrum ($CDCl_3$)

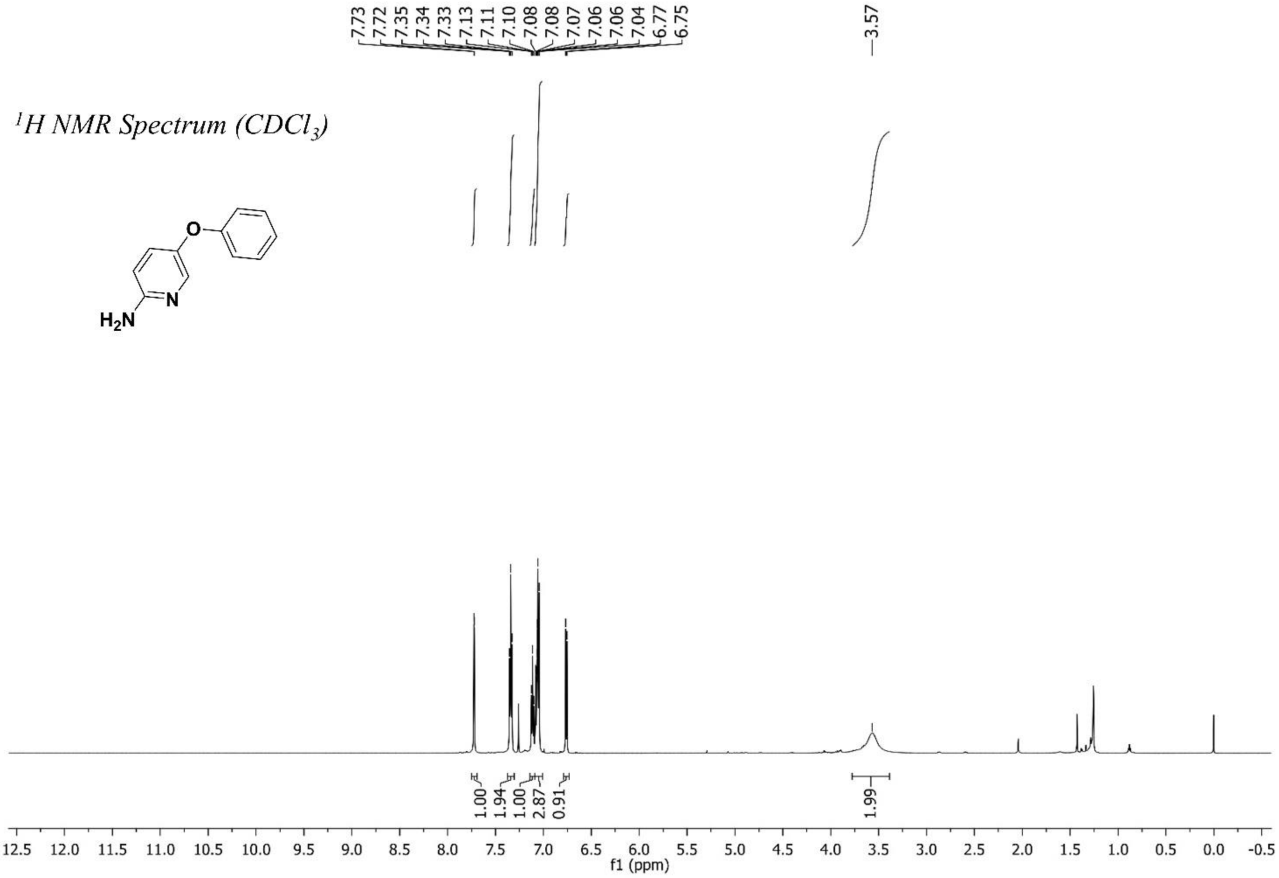

^{13}C NMR Spectrum ($CDCl_3$)

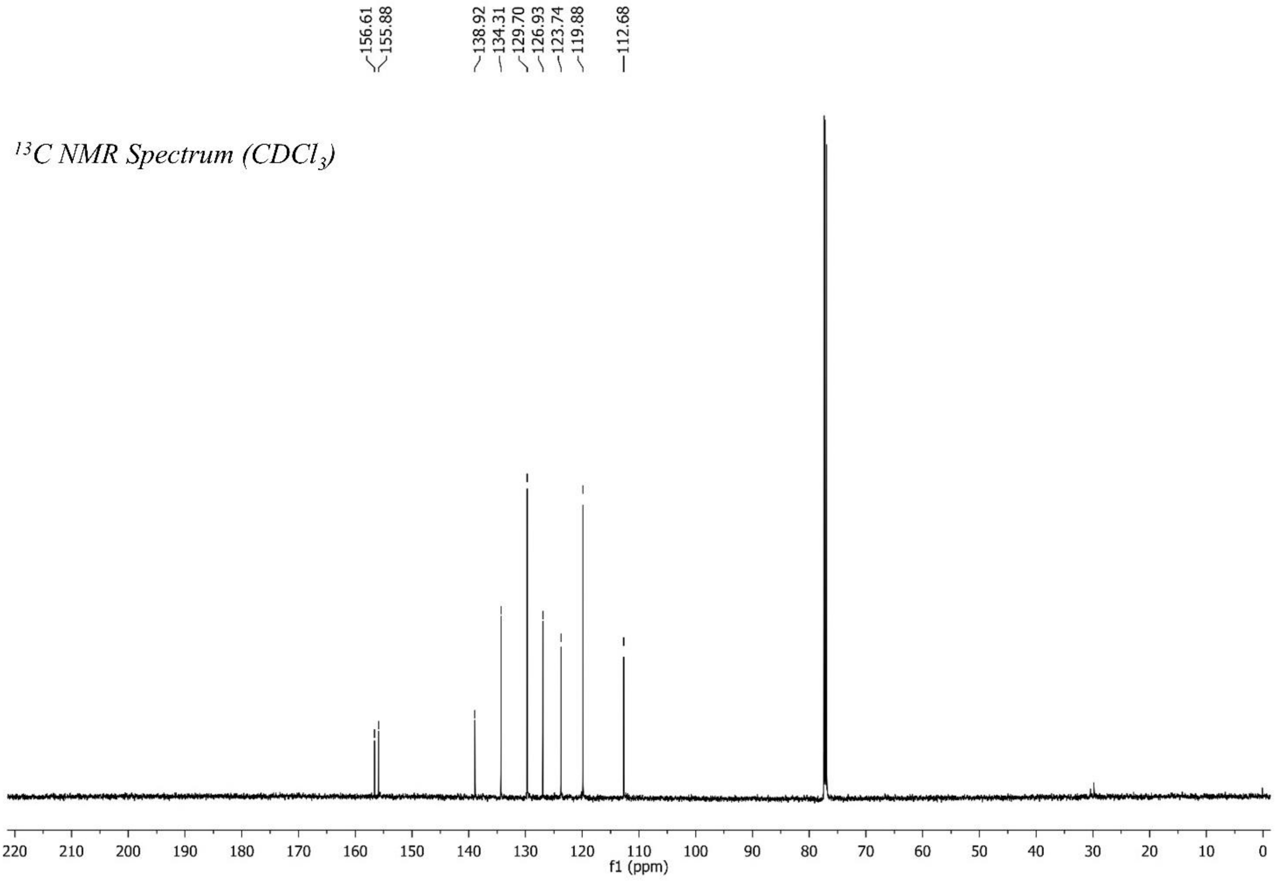

5-[3-(Trifluoromethyl)phenoxy]pyridine-2-amine (**43**)

^{1}H NMR Spectrum ($CDCl_3$)

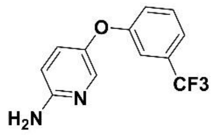

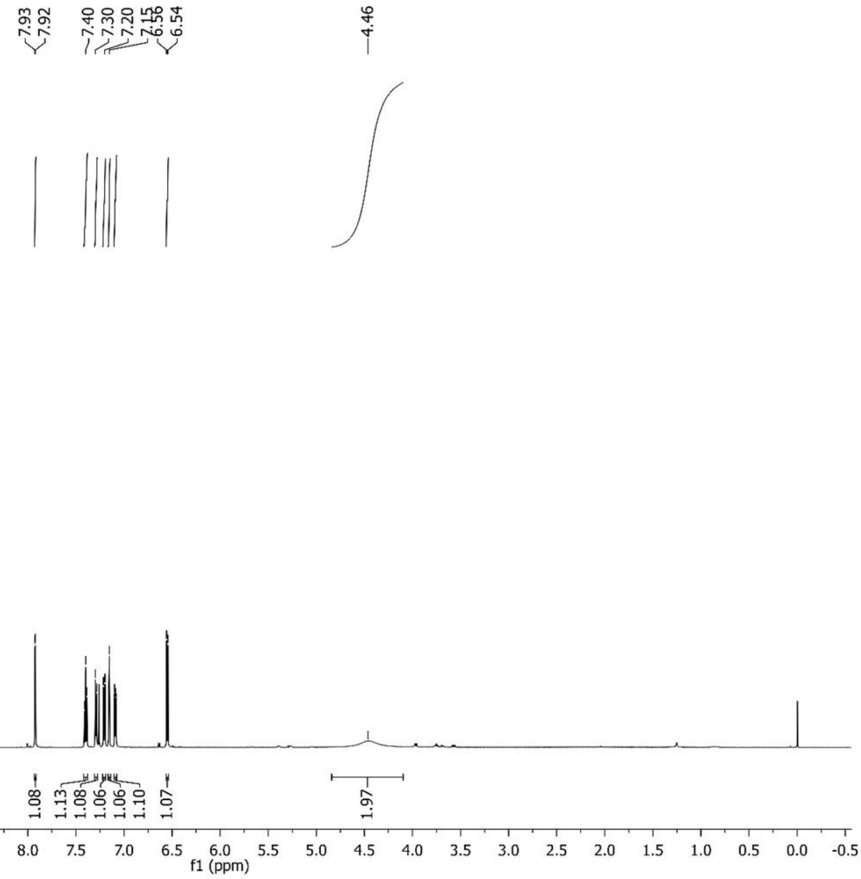

^{13}C NMR Spectrum ($CDCl_3$)

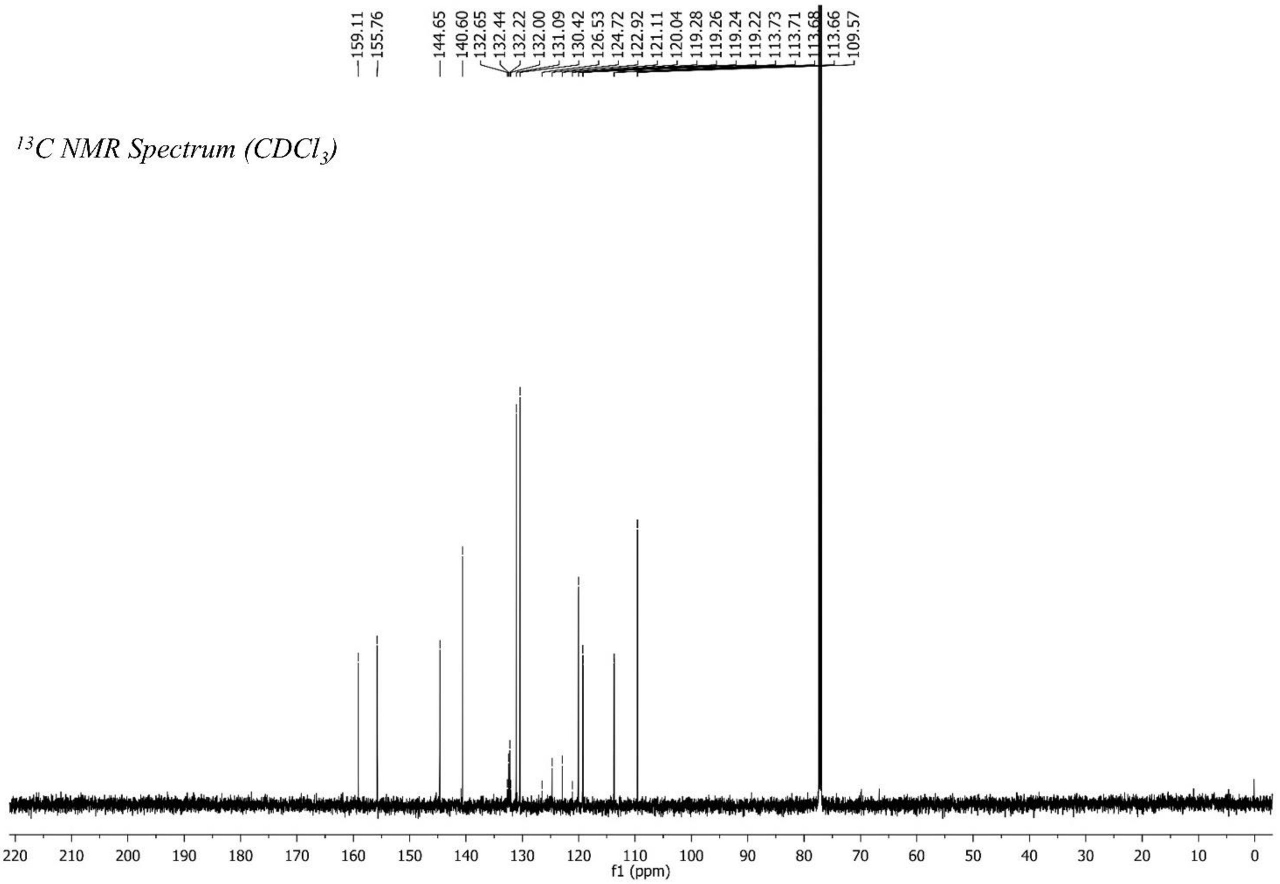

5-Isopropyl-2-methyl-4-nitrophenol (**65**)

^{1}H NMR Spectrum ($CDCl_3$)

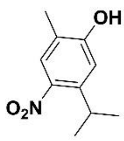

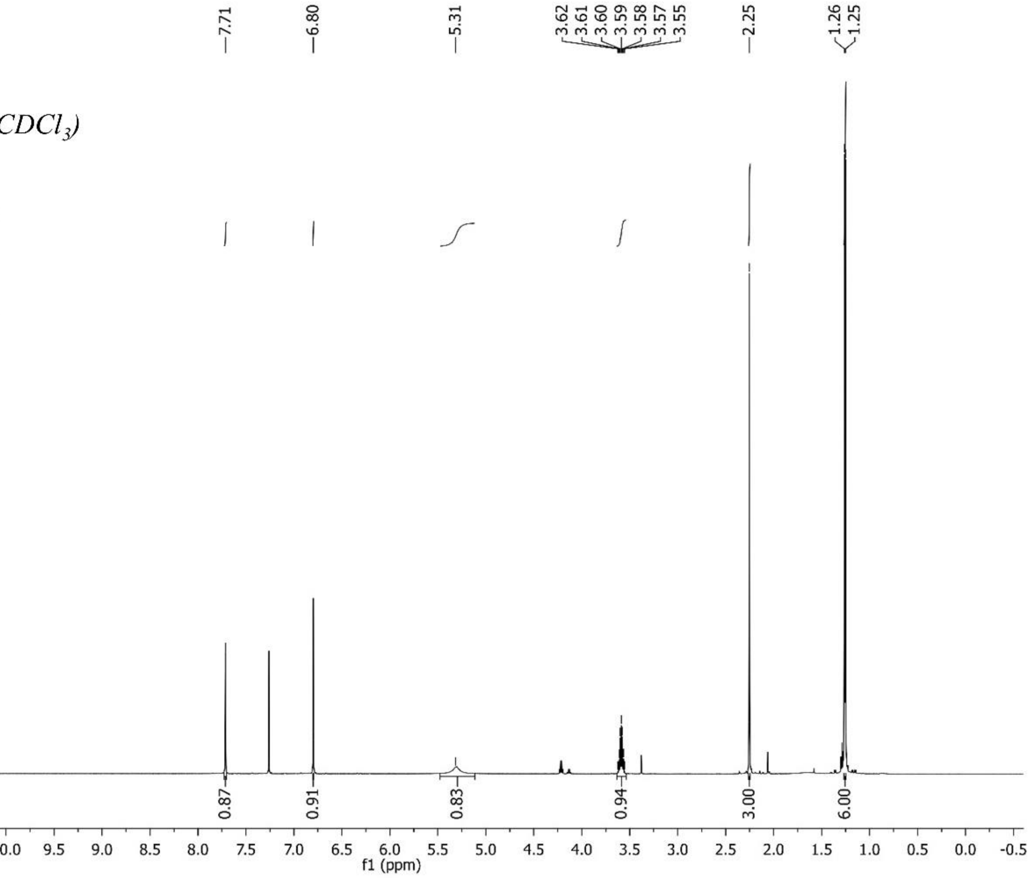

^{13}C NMR Spectrum ($CDCl_3$)

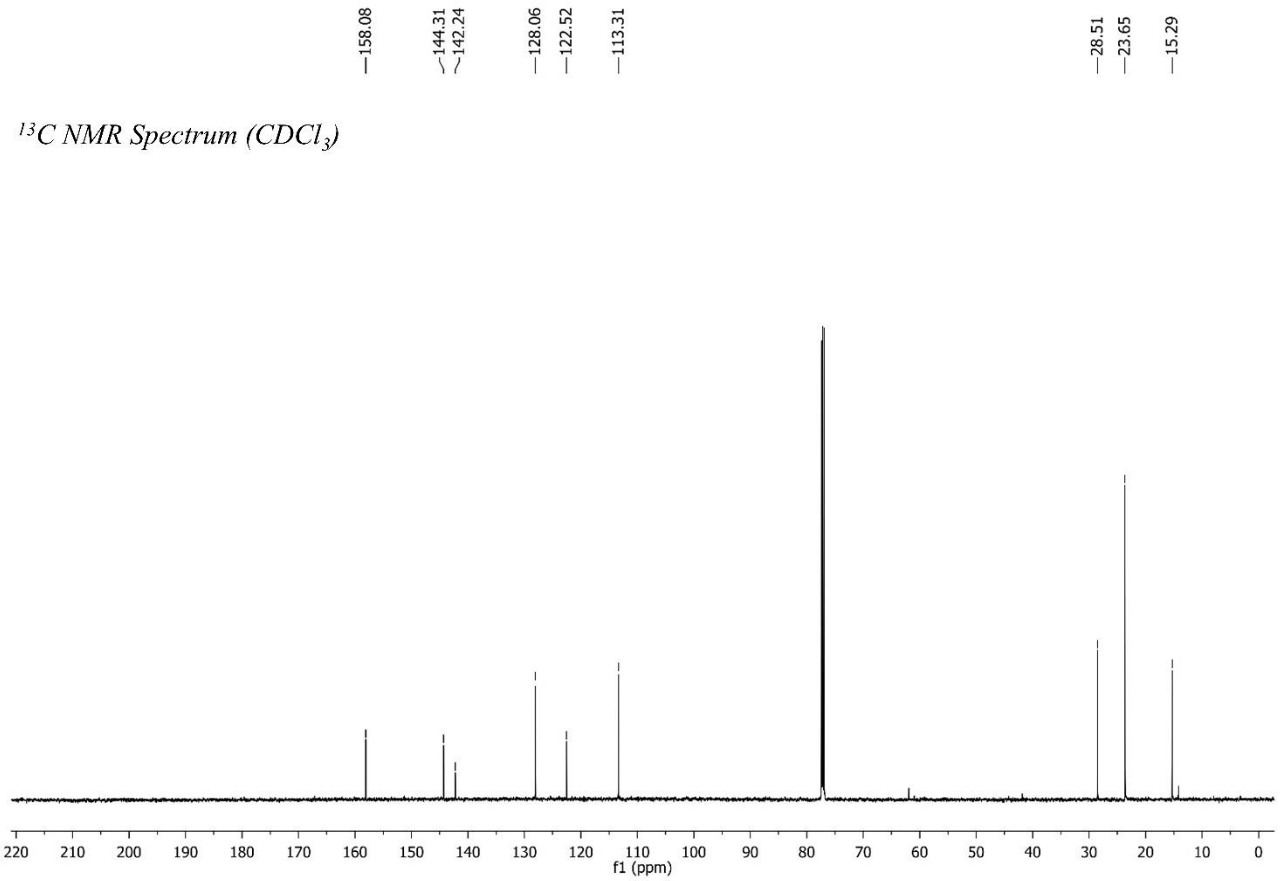

2-Isopropyl-5-methyl-4-nitrophenol (**66**)

^1H NMR Spectrum ($CDCl_3$)

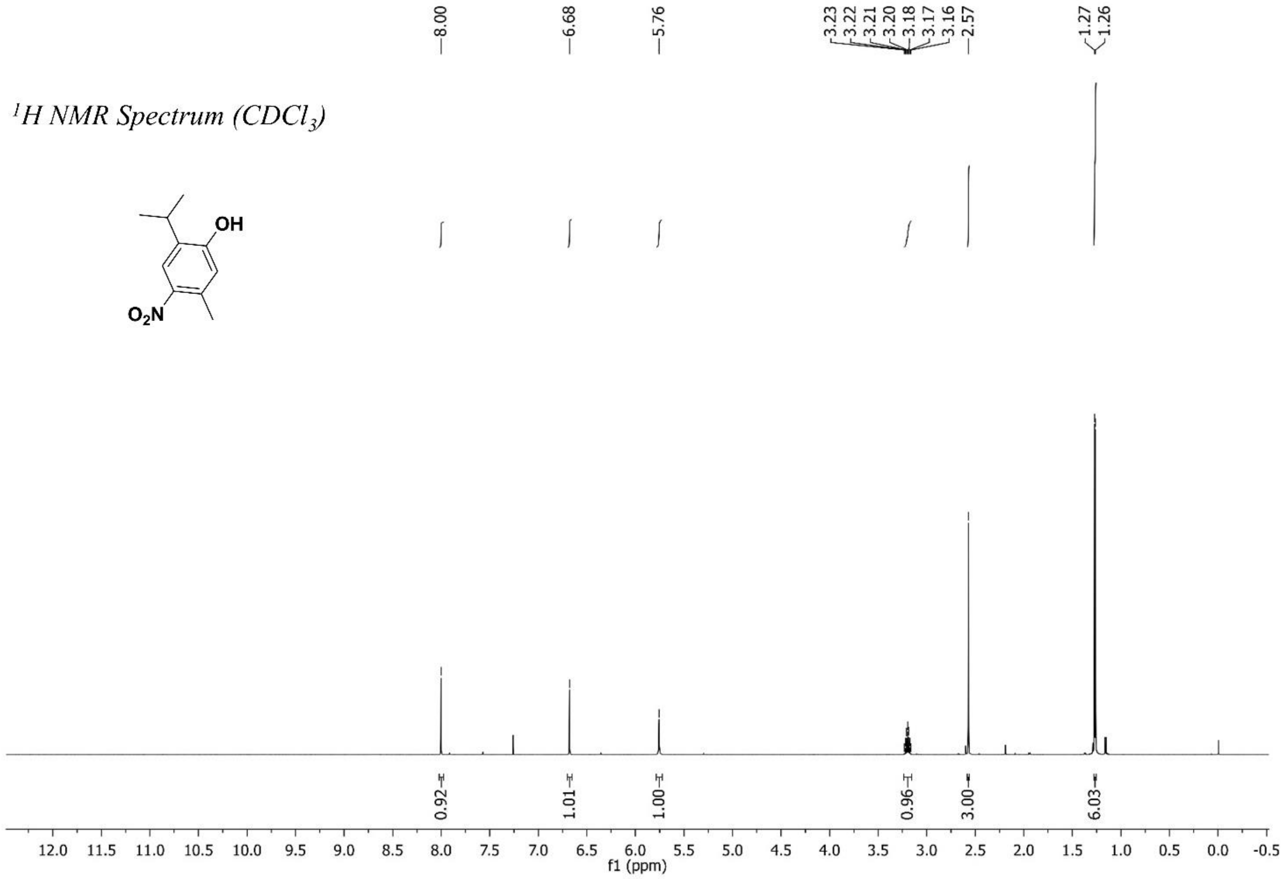

^{13}C NMR Spectrum ($CDCl_3$)

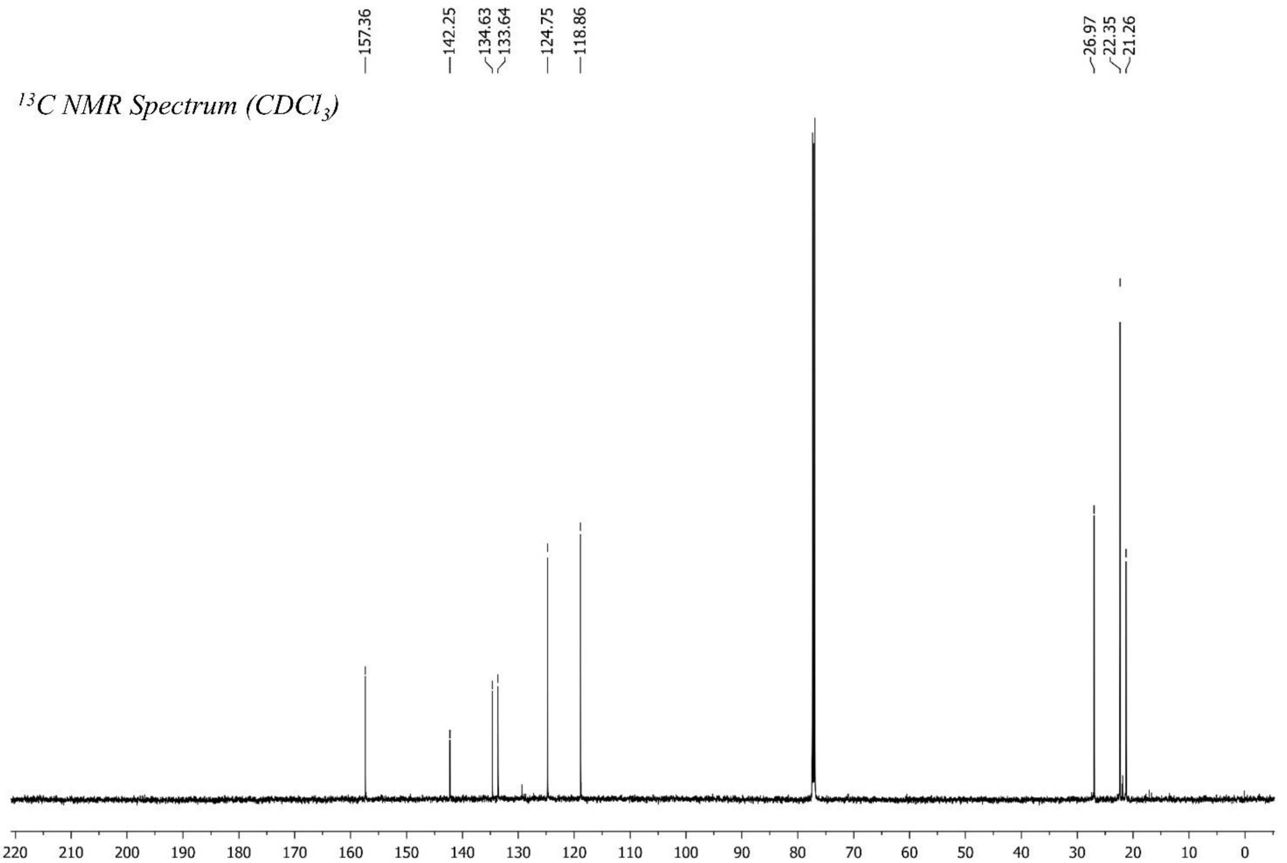

1-Isopropil-4-methyl-5-nitro-2-phenoxybenzene (**67**)

^{1}H NMR Spectrum ($CDCl_3$)

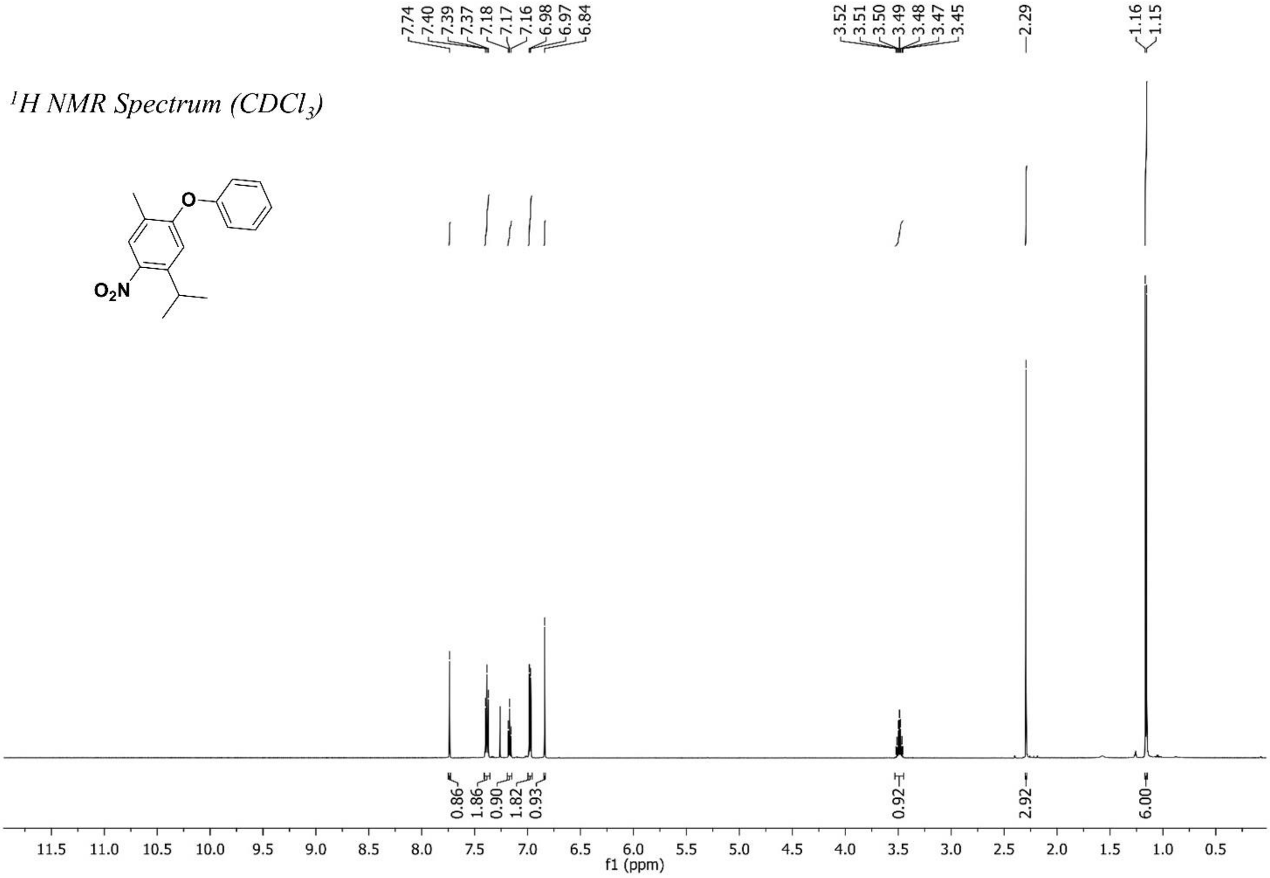

^{13}C NMR Spectrum ($CDCl_3$)

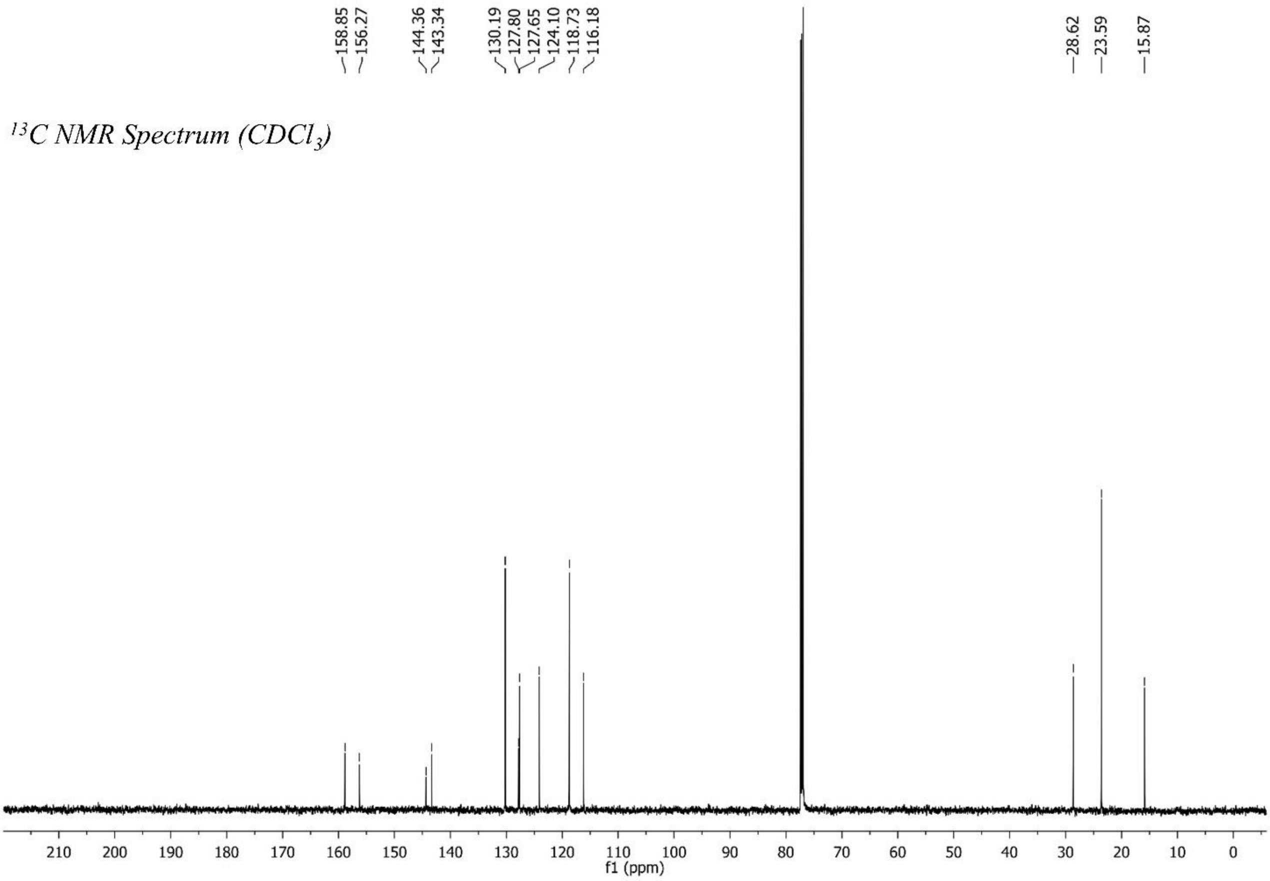

1-Isopropil-4-methyl-5-nitro-2-phenoxybenzene (**68**)

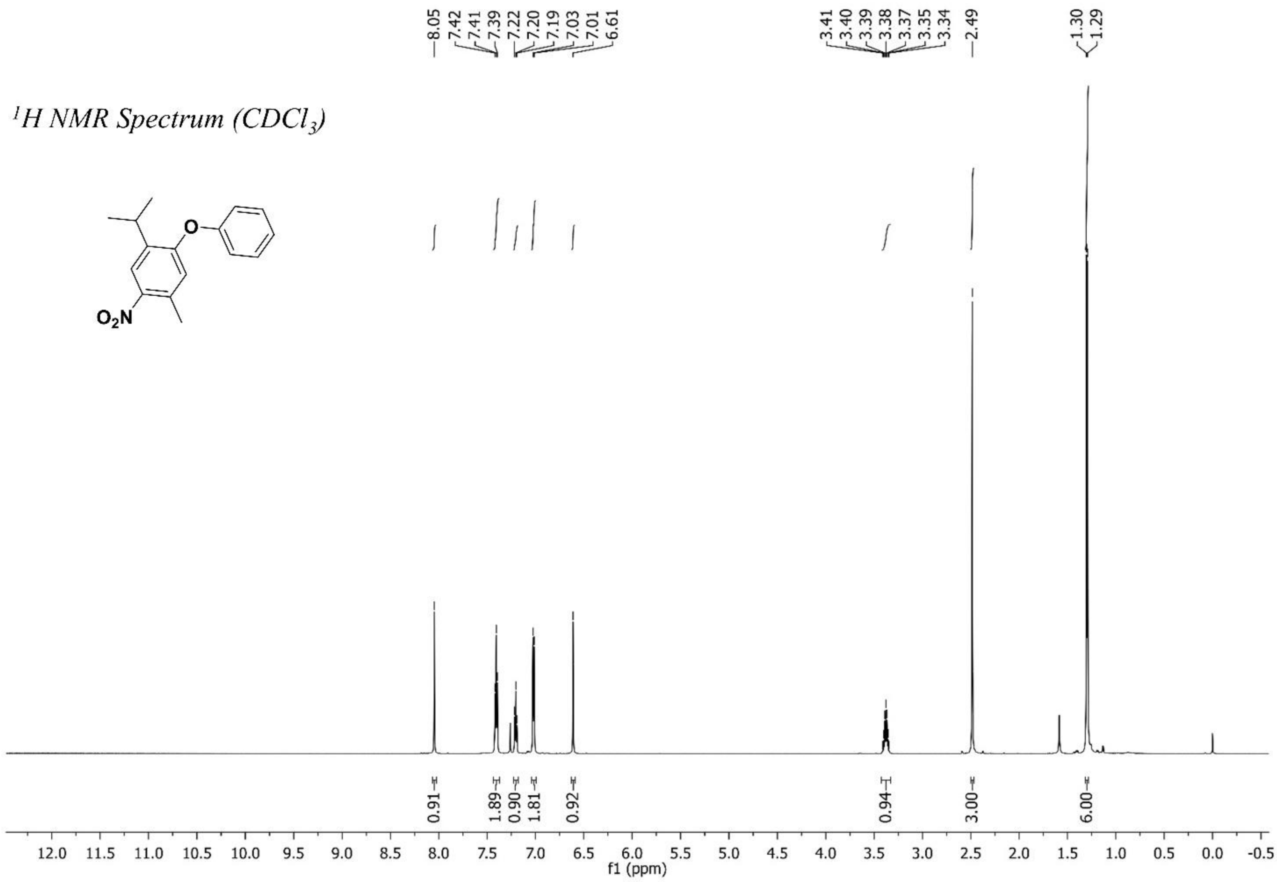

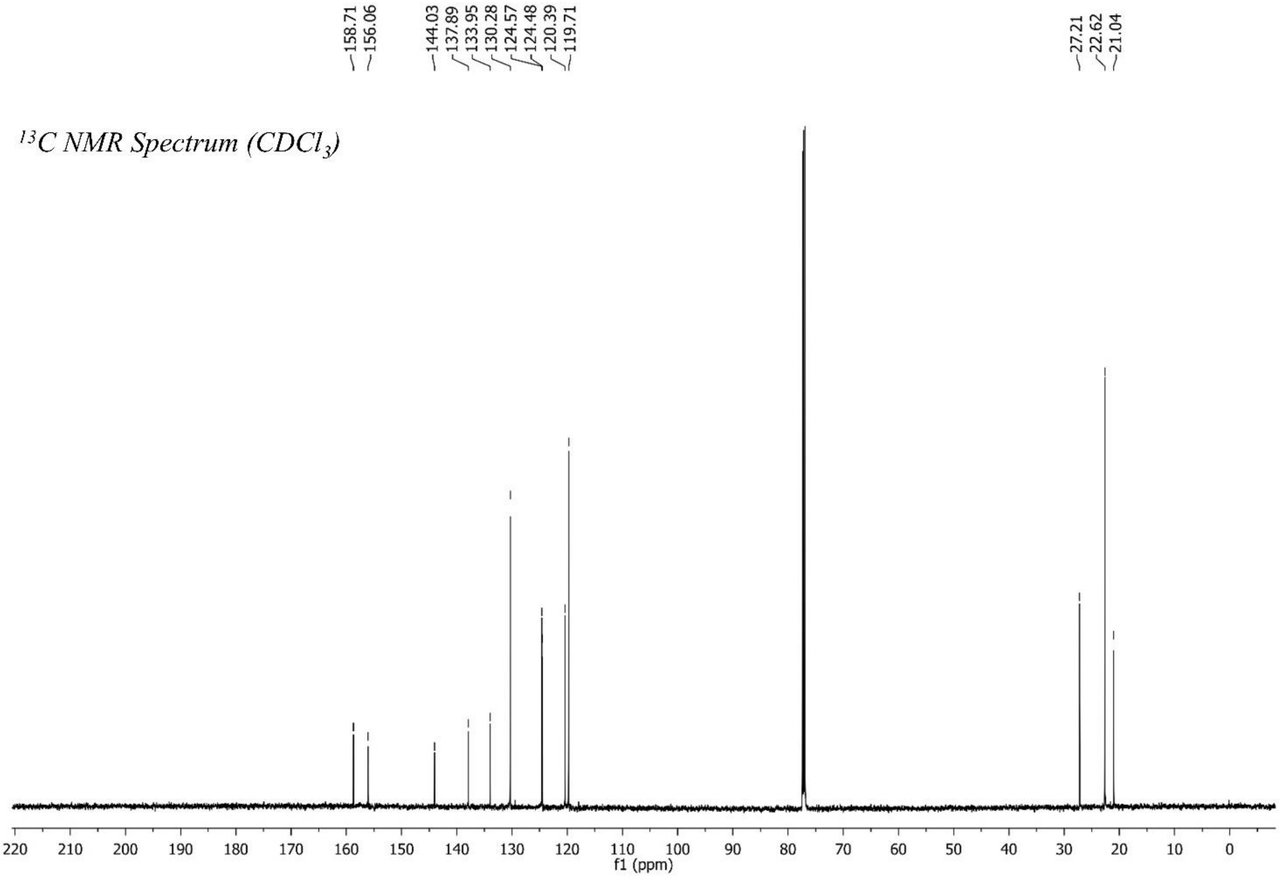

1-Isopropyl-4-methyl-2-nitro-5-(4-(trifluoromethyl)phenoxy)benzene (**69**)

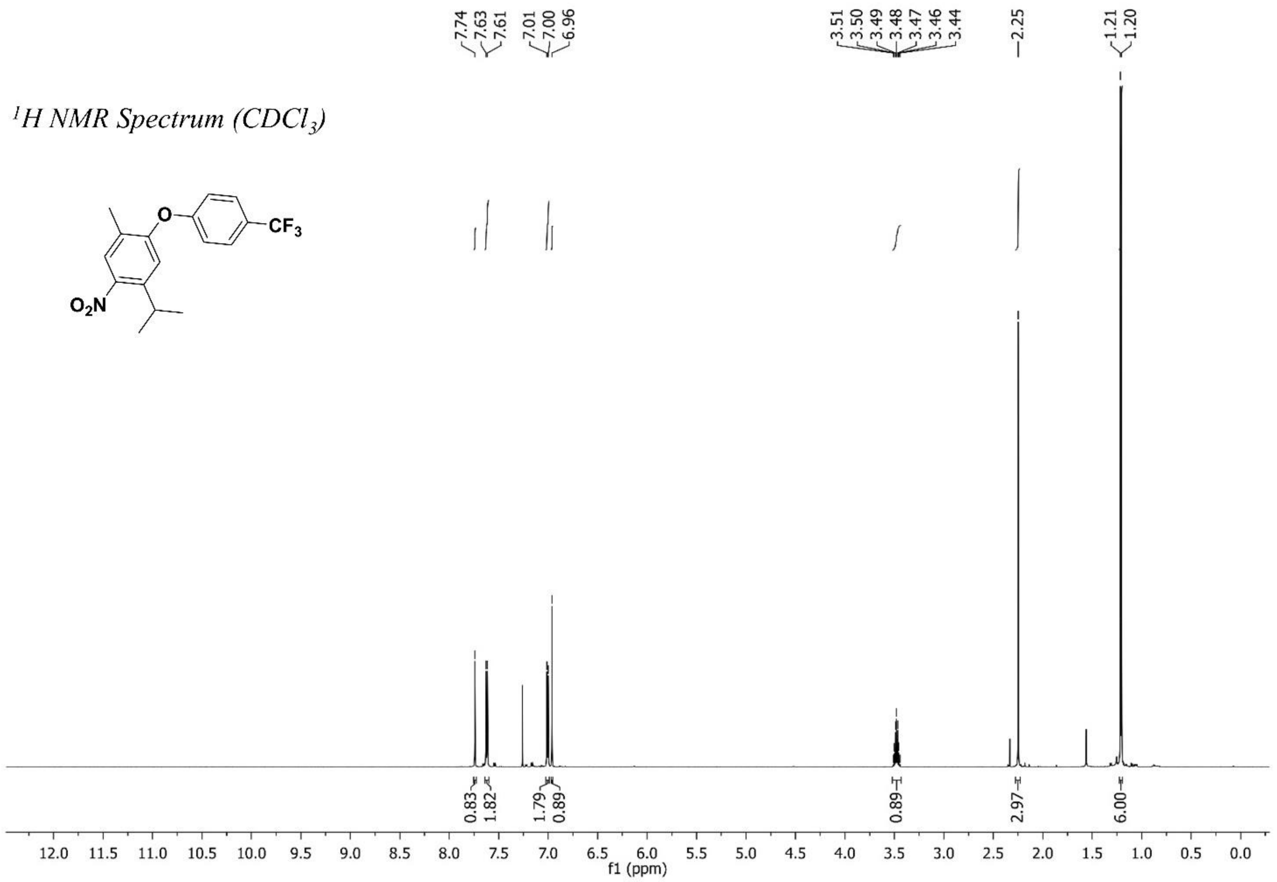

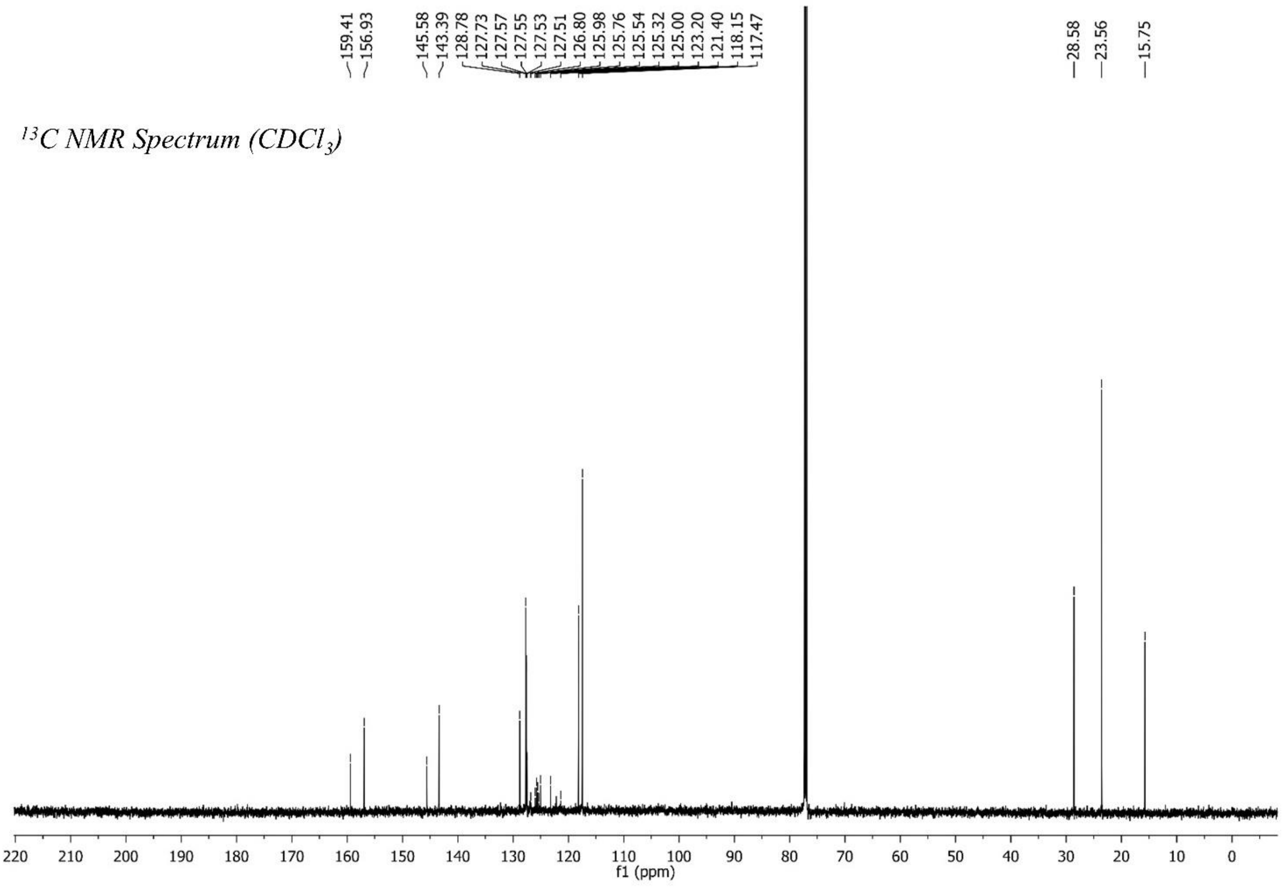

4-(5-Isopropyl-2-methyl-4-nitrophenoxy)pyridine (**70**)

^1H NMR Spectrum ($CDCl_3$)

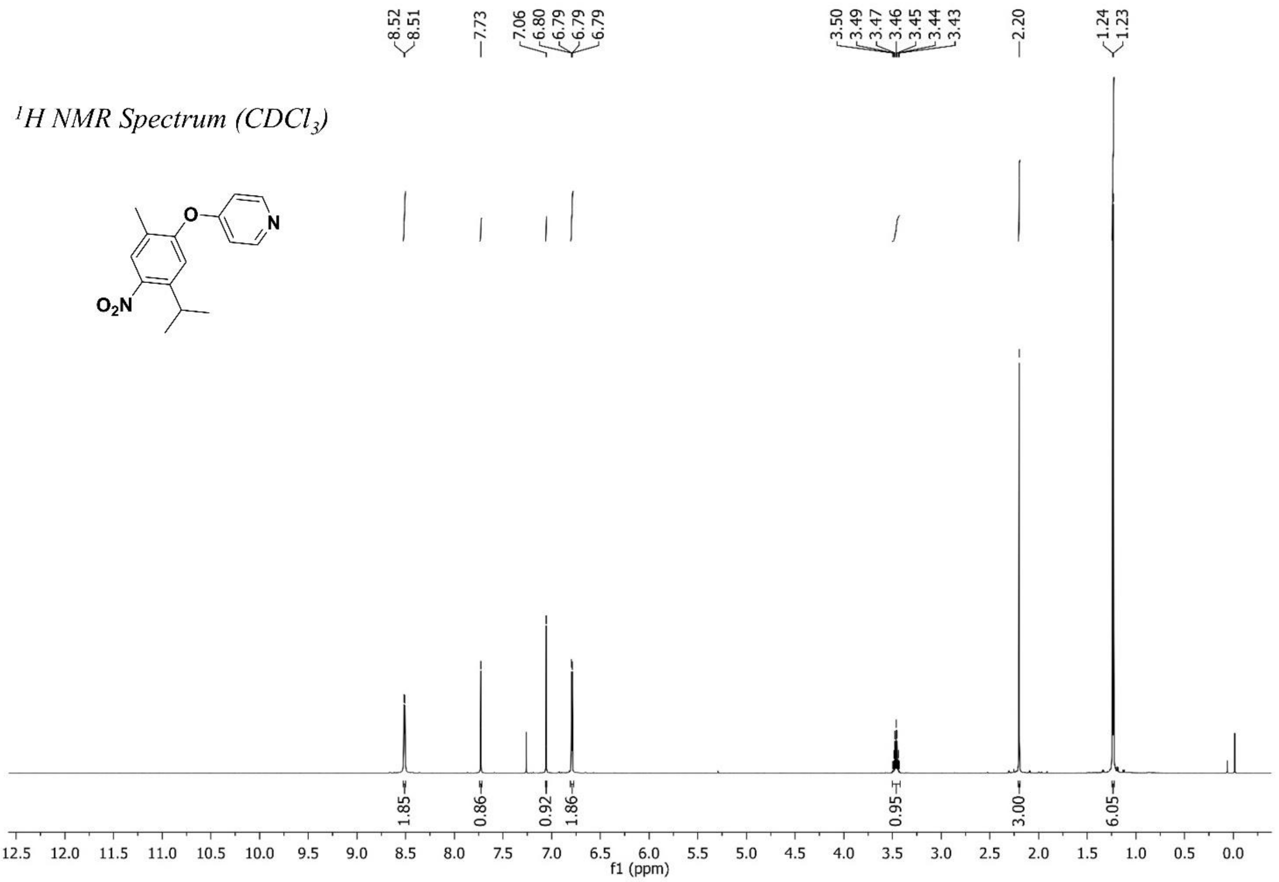

^{13}C NMR Spectrum ($CDCl_3$)

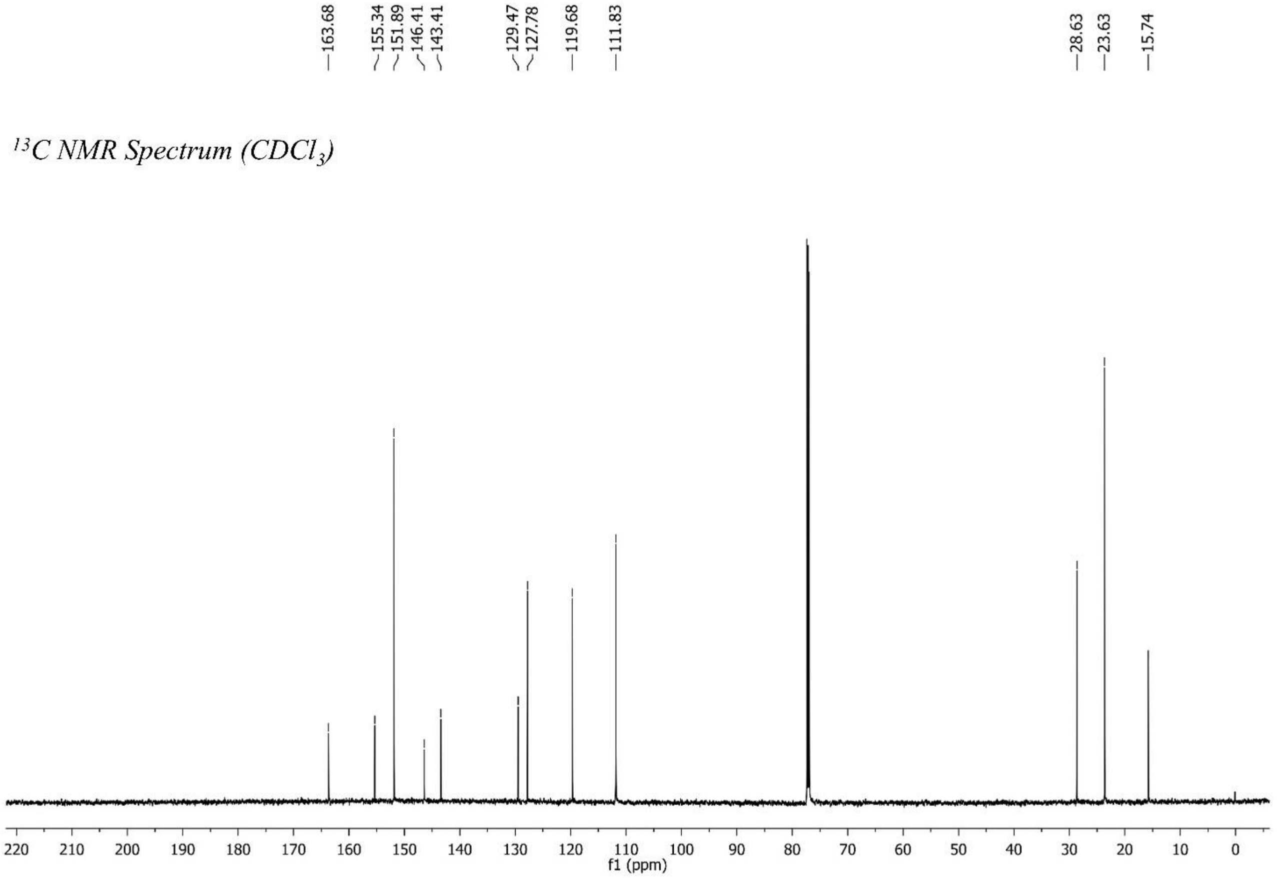

2-Isopropyl-5-methyl-4-phenoxyaniline (**40**)

^{1}H NMR Spectrum ($CDCl_3$)

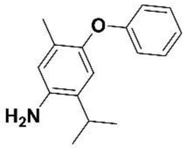

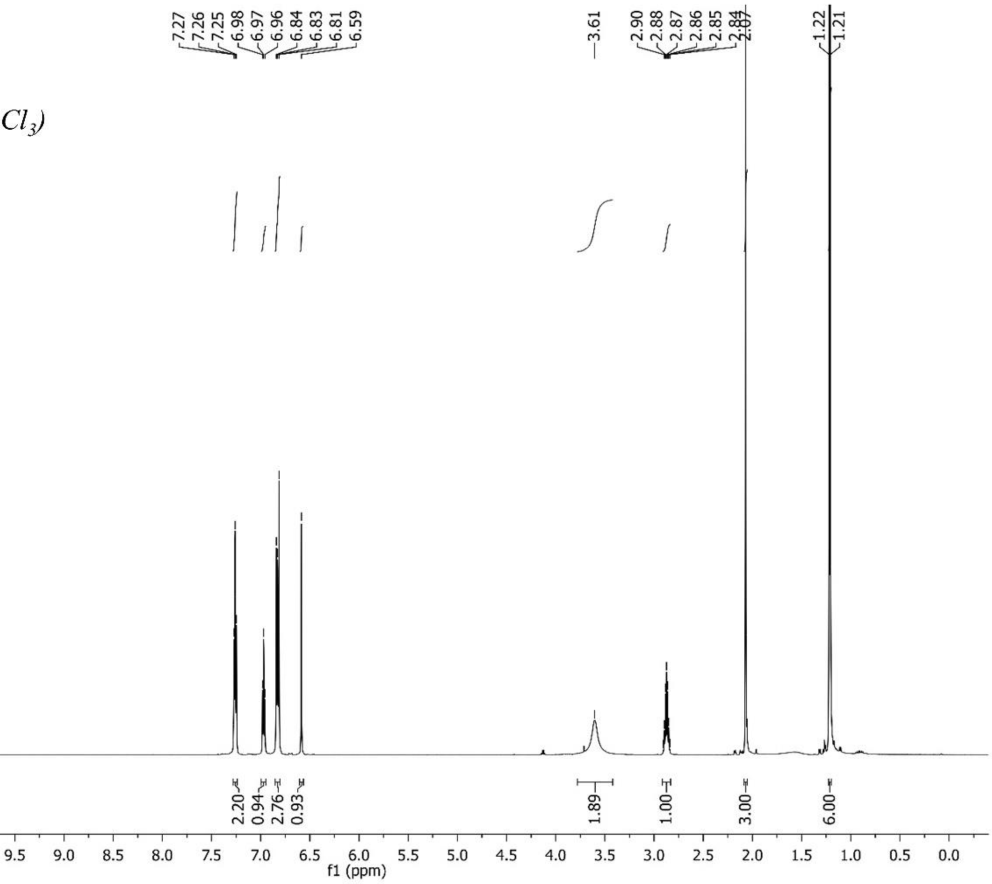

^{13}C NMR Spectrum ($CDCl_3$)

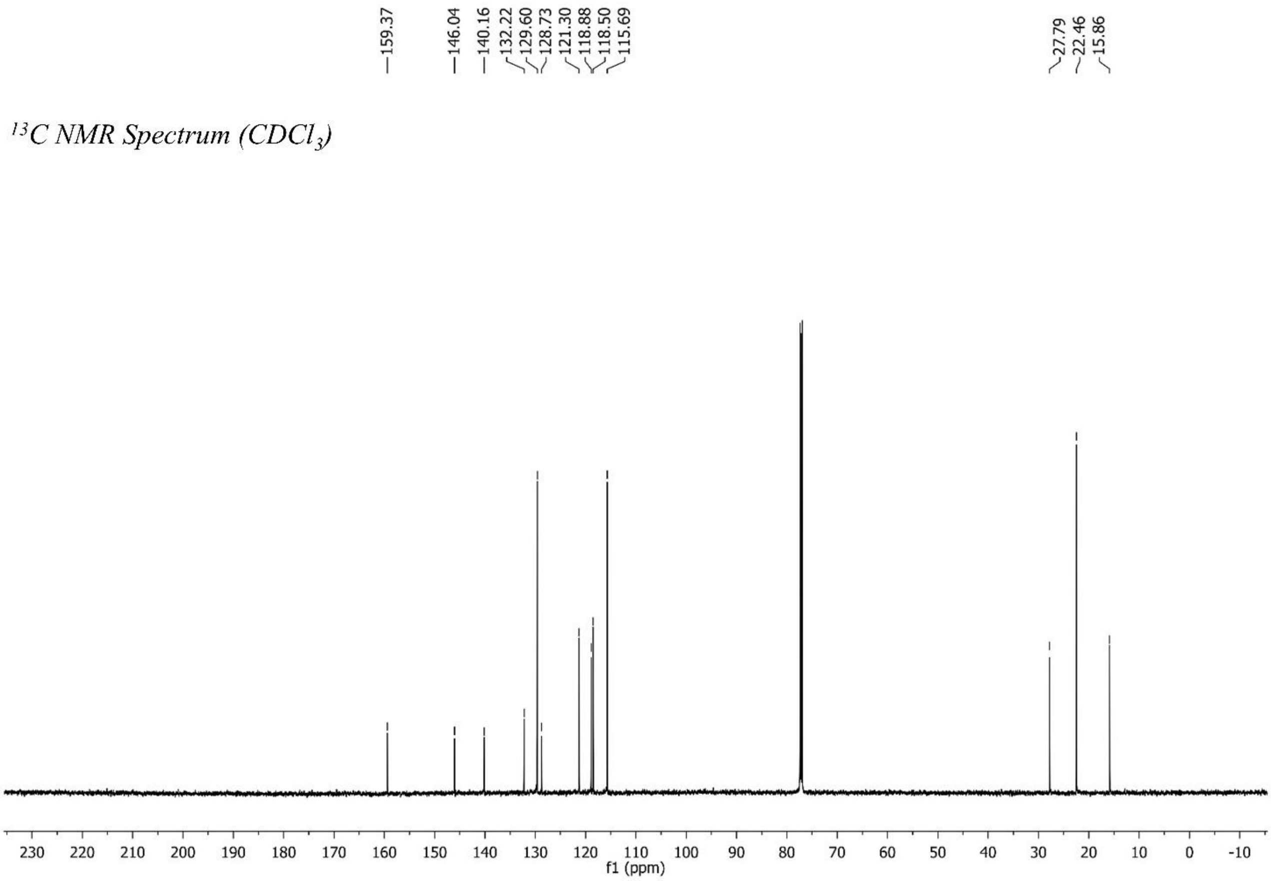

5-Isopropyl-2-methyl-4-phenoxyaniline (**41**)

^{1}H NMR Spectrum (CDCl$_3$)

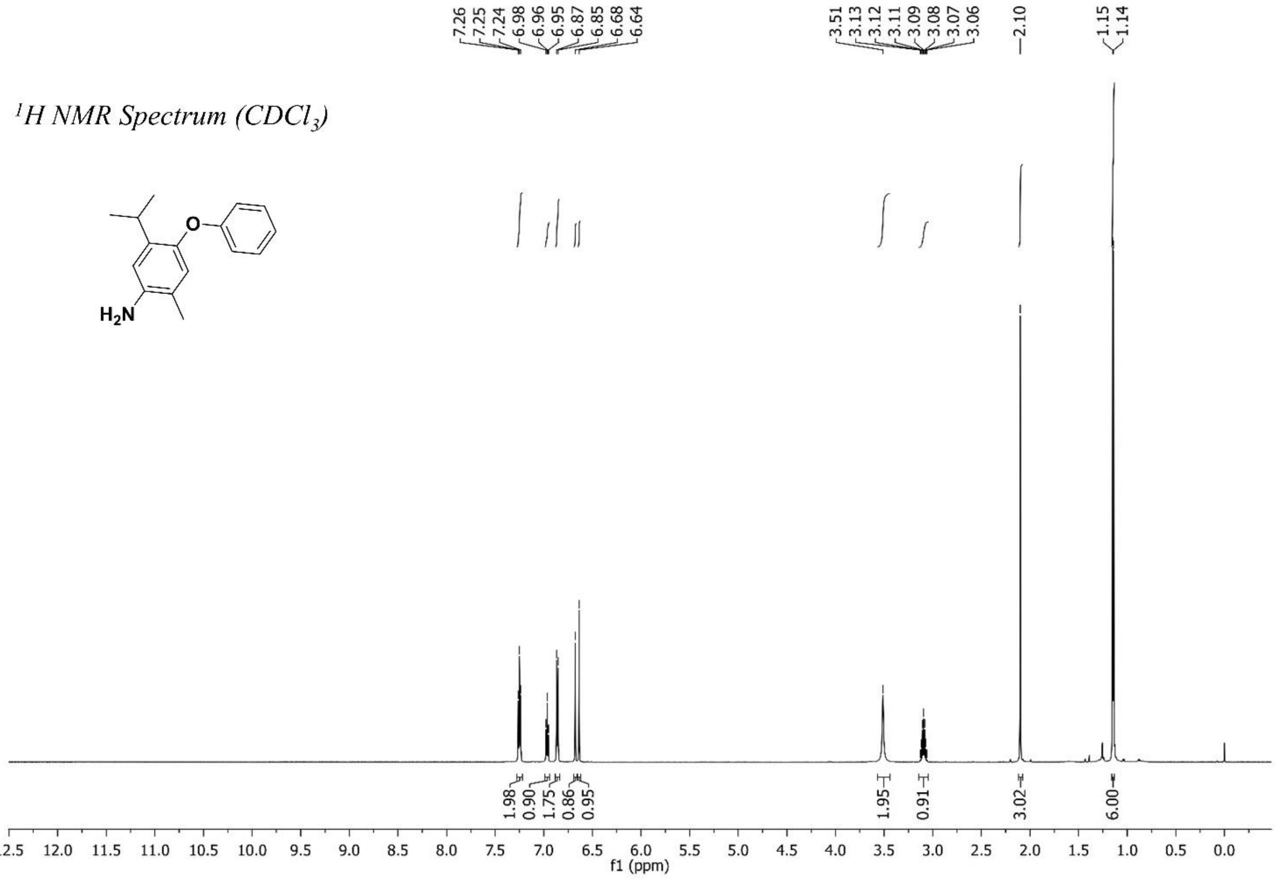

^{13}C NMR Spectrum (CDCl$_3$)

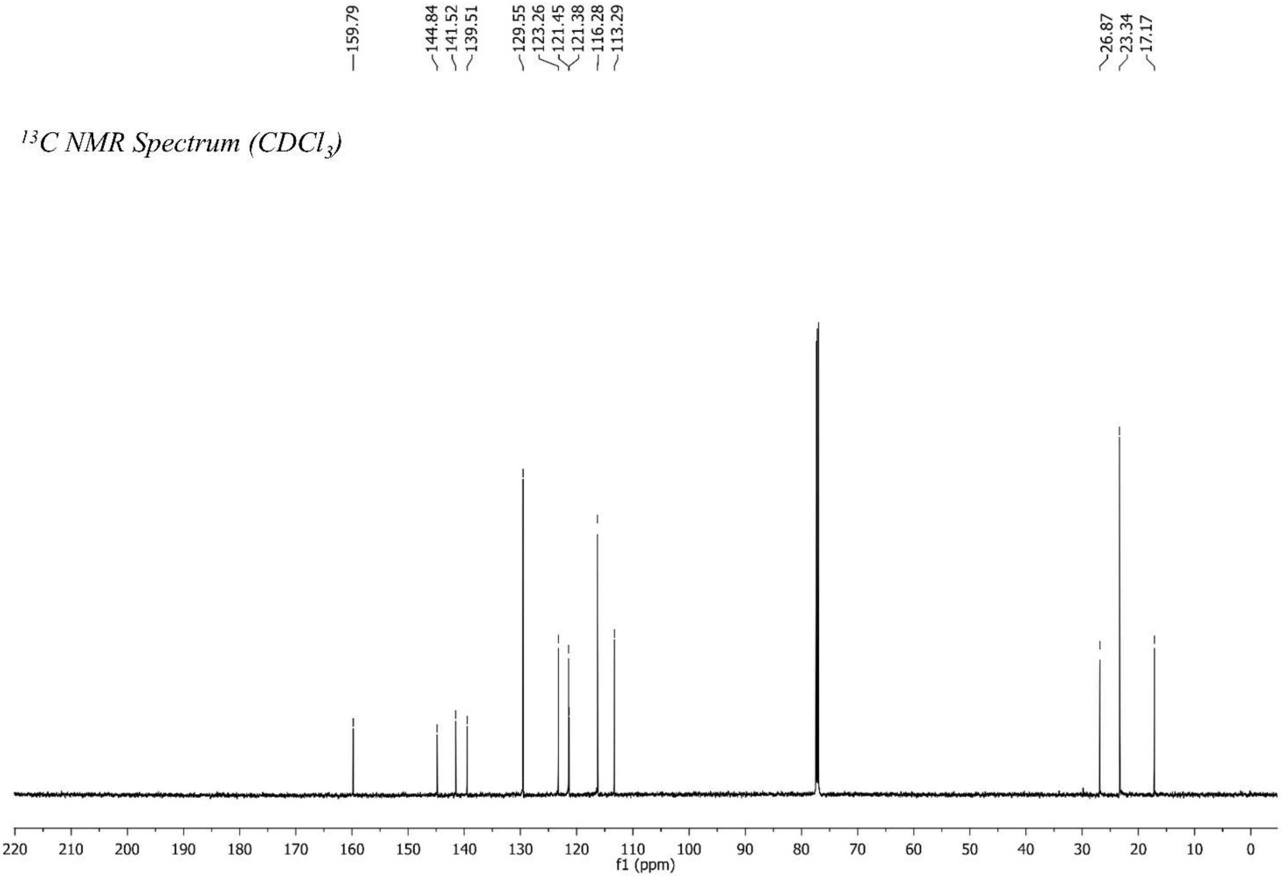

2-Isopropyl-5-methyl-4-(4-(trifluoromethyl)phenoxy)aniline (**44**)

^{1}H NMR Spectrum (CDCl$_3$)

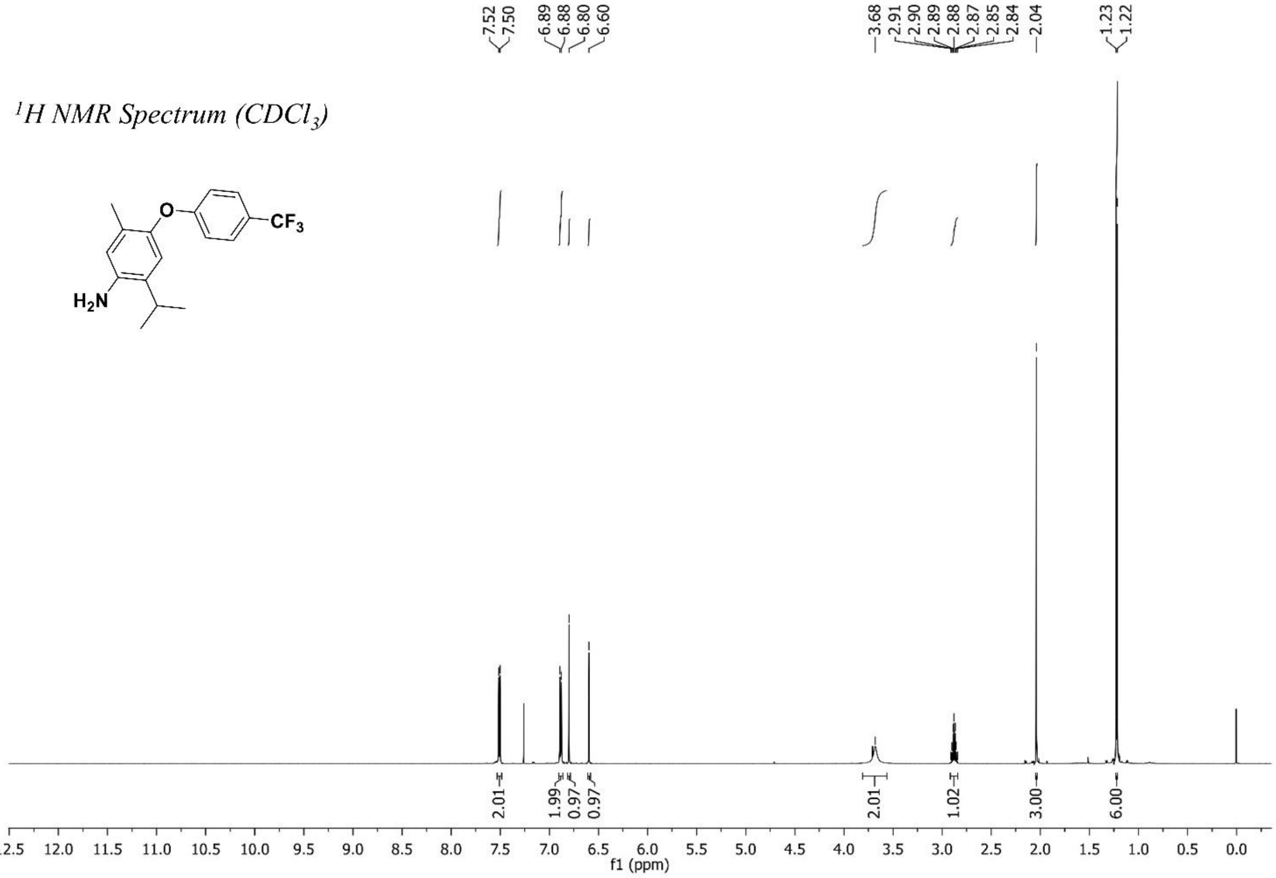

^{13}C NMR Spectrum (CDCl$_3$)

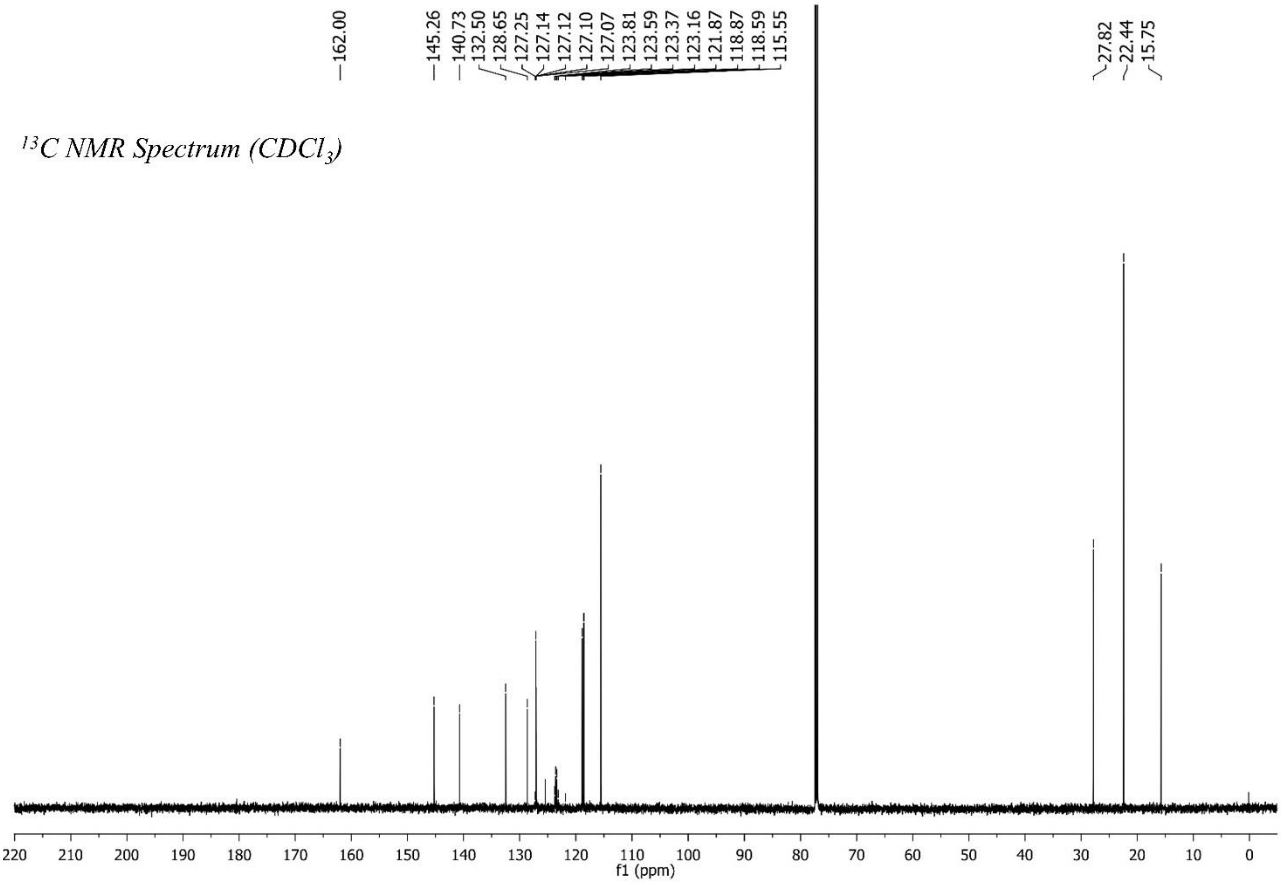

2-Isopropyl-5-methyl-4-(pyridin-4-yloxy)aniline (**55**)

^{1}H NMR Spectrum ($CDCl_3$)

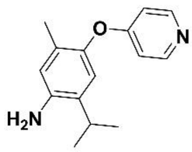

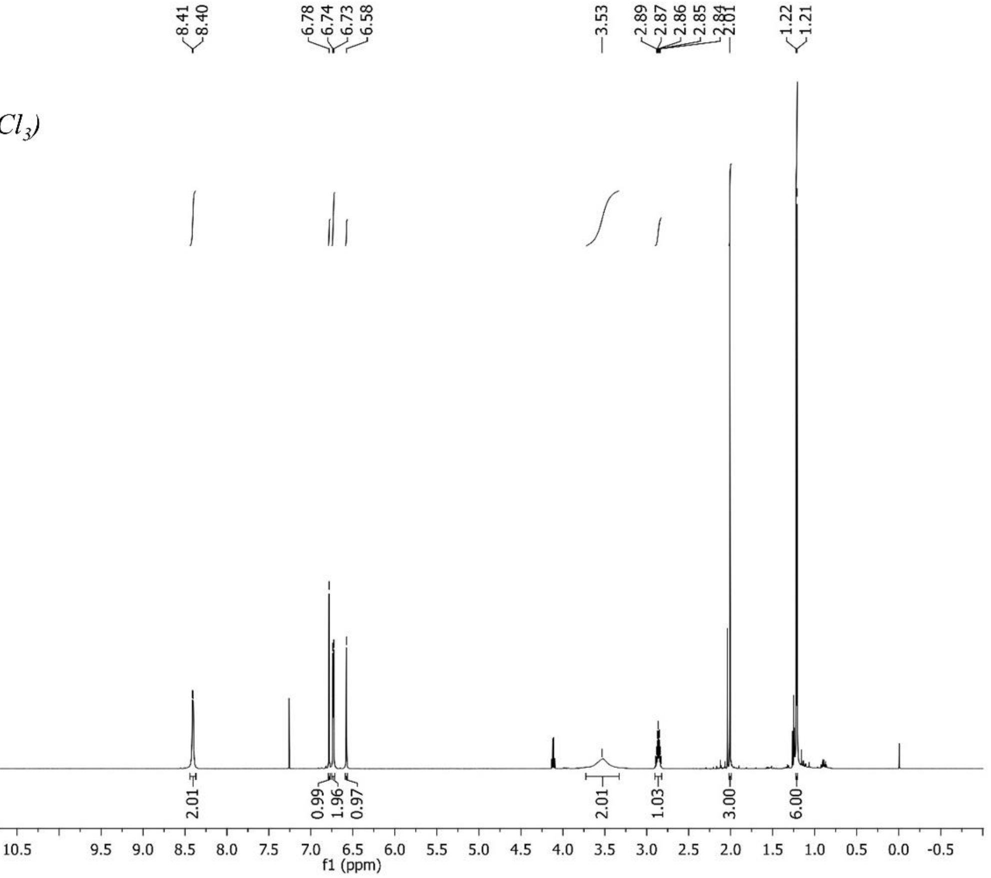

^{13}C NMR Spectrum ($CDCl_3$)

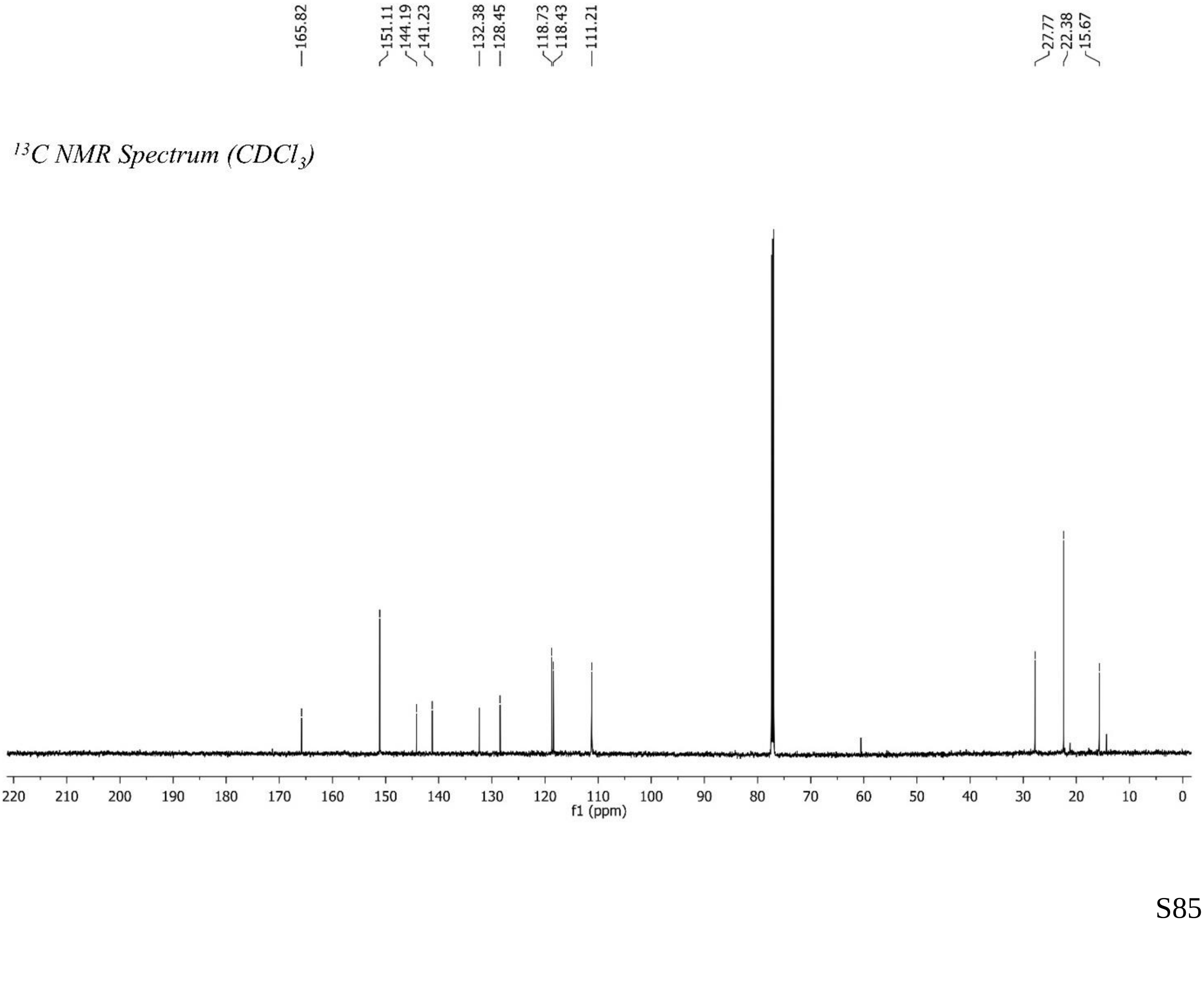

1-Chloro-5-isopropoxy-2-methyl-4-nitrobenzene (**72**)

^{1}H NMR Spectrum ($CDCl_3$)

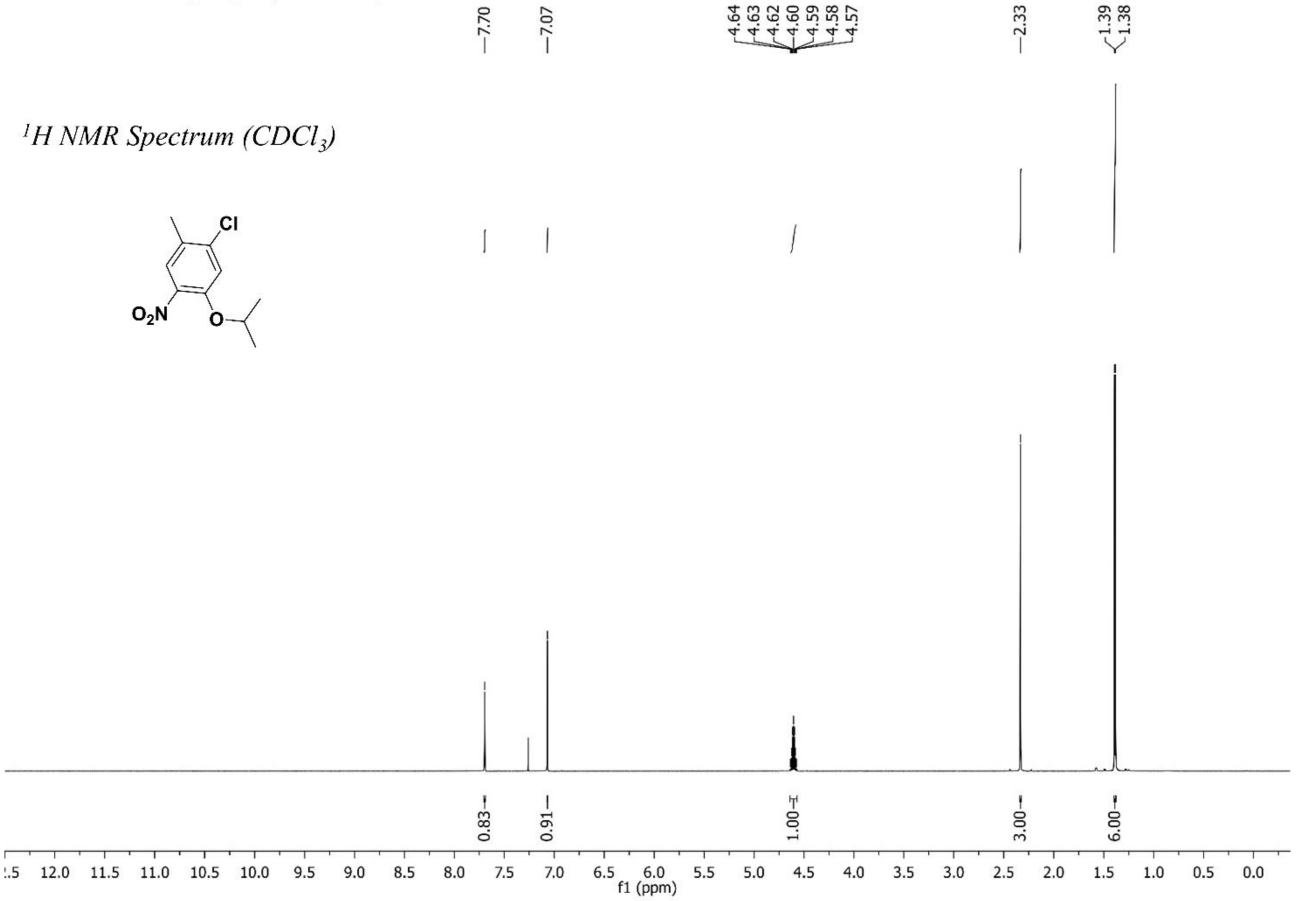

^{13}C NMR Spectrum ($CDCl_3$)

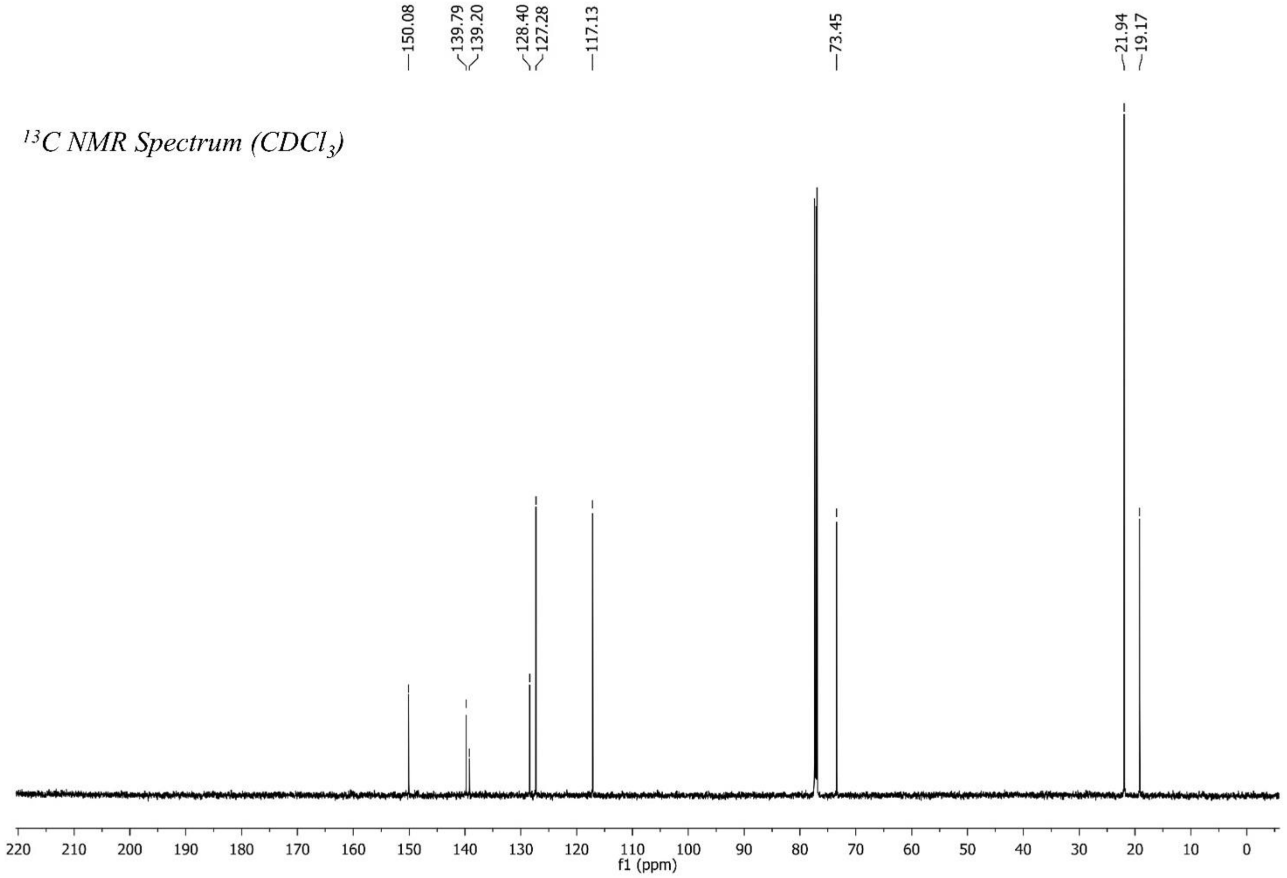

1-Chloro-5-cyclobutoxy-2-methyl-4-nitrobenzene (**73**)

^1H NMR Spectrum ($CDCl_3$)

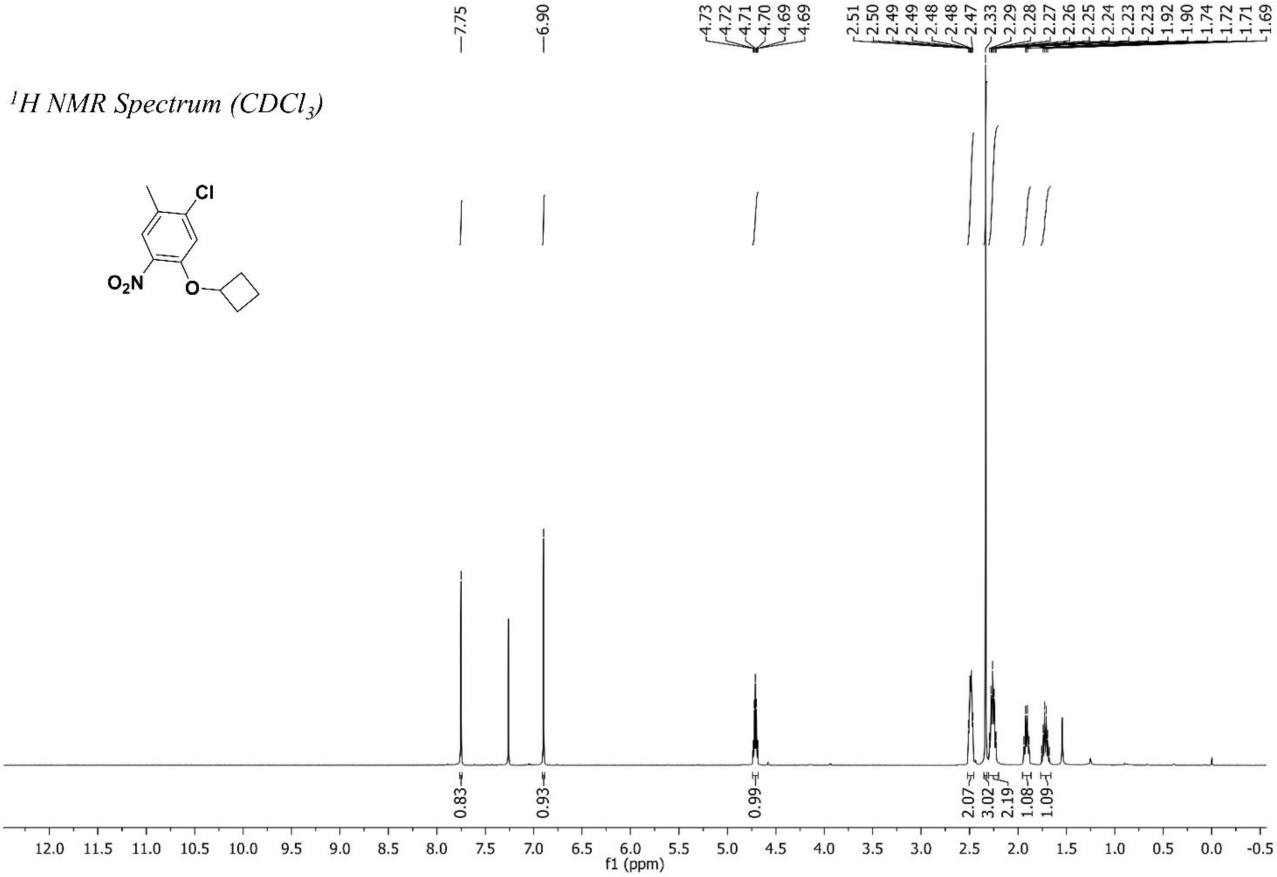

^{13}C NMR Spectrum ($CDCl_3$)

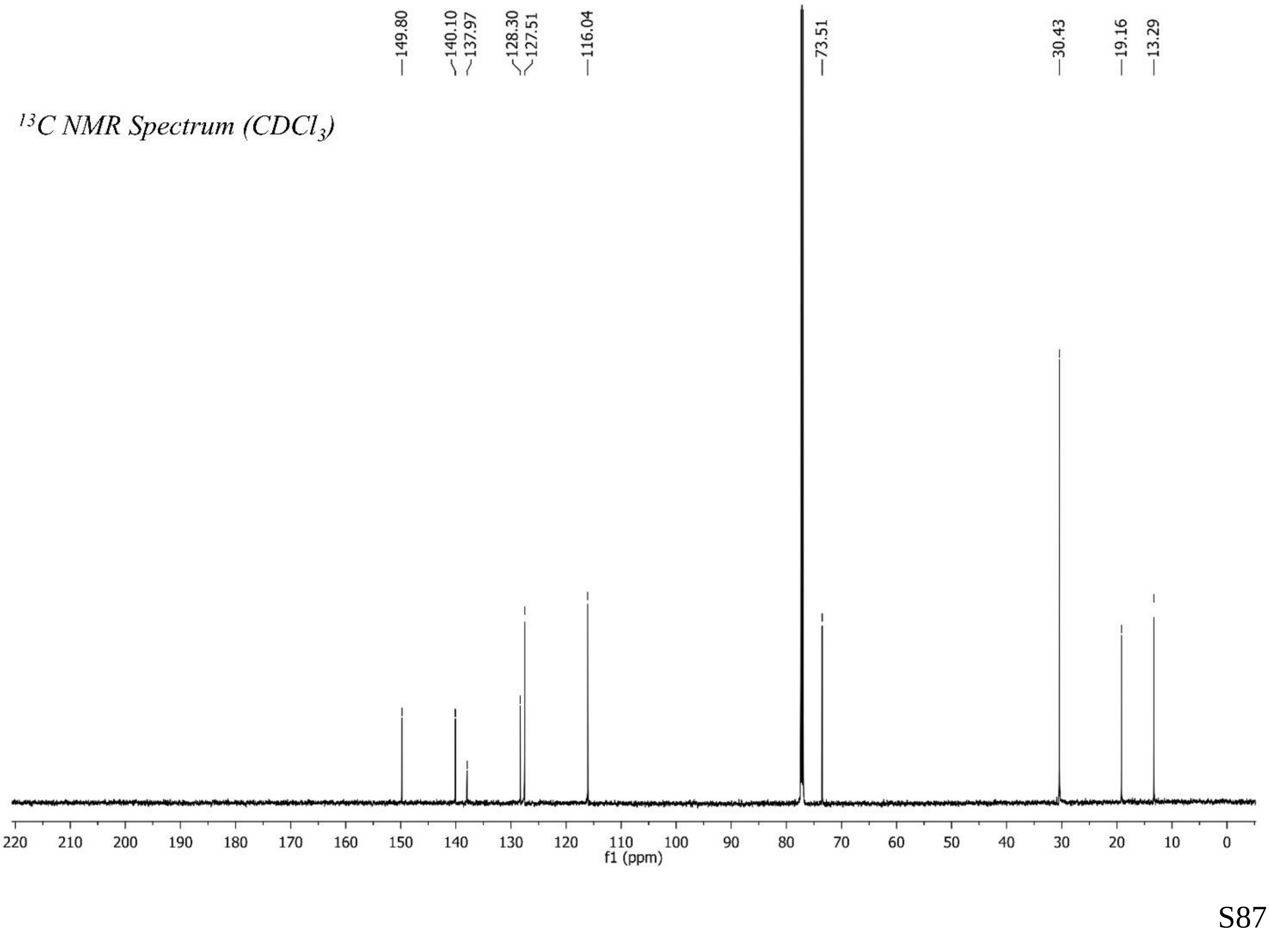

1-(sec-Butoxy)-5-chloro-4-methyl-4-nitrobenzene (**74**)

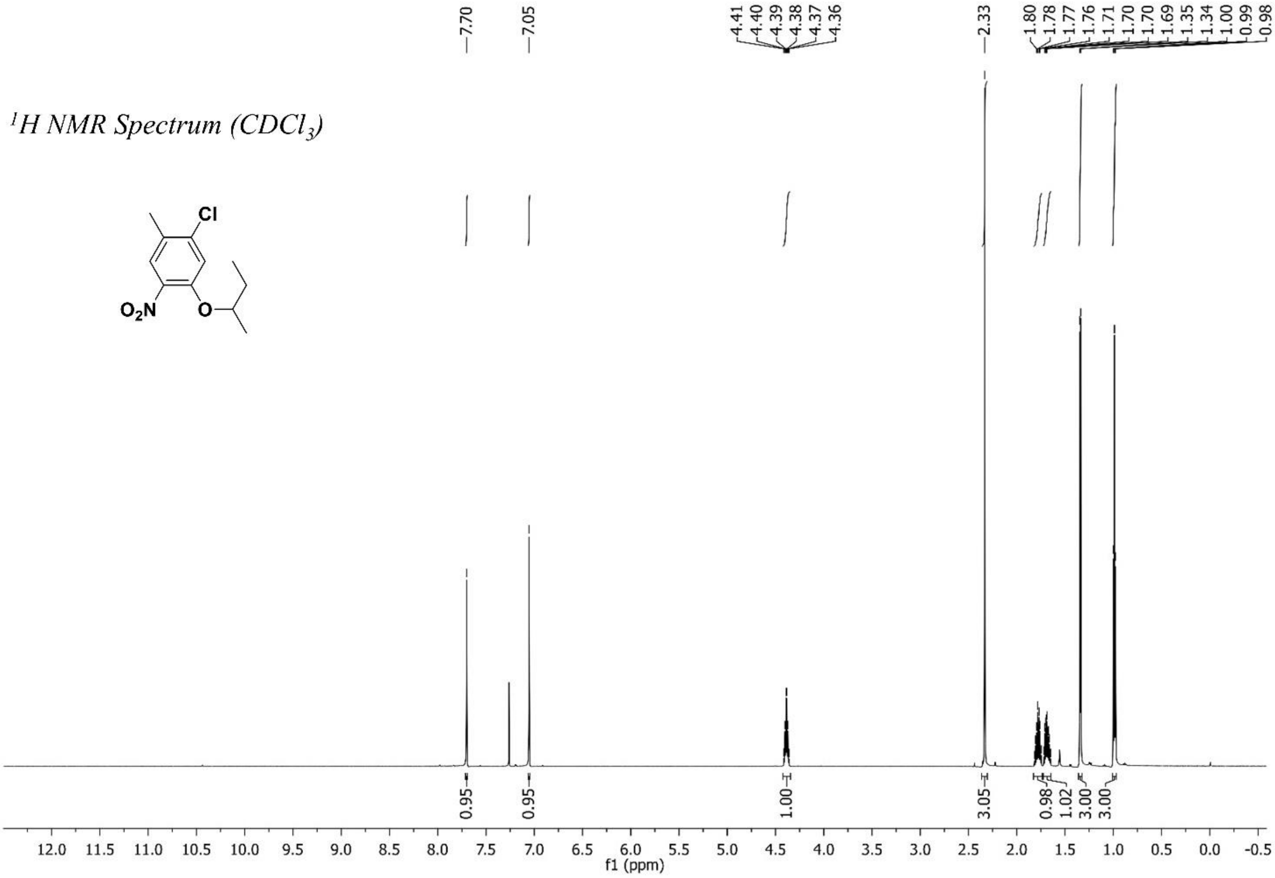

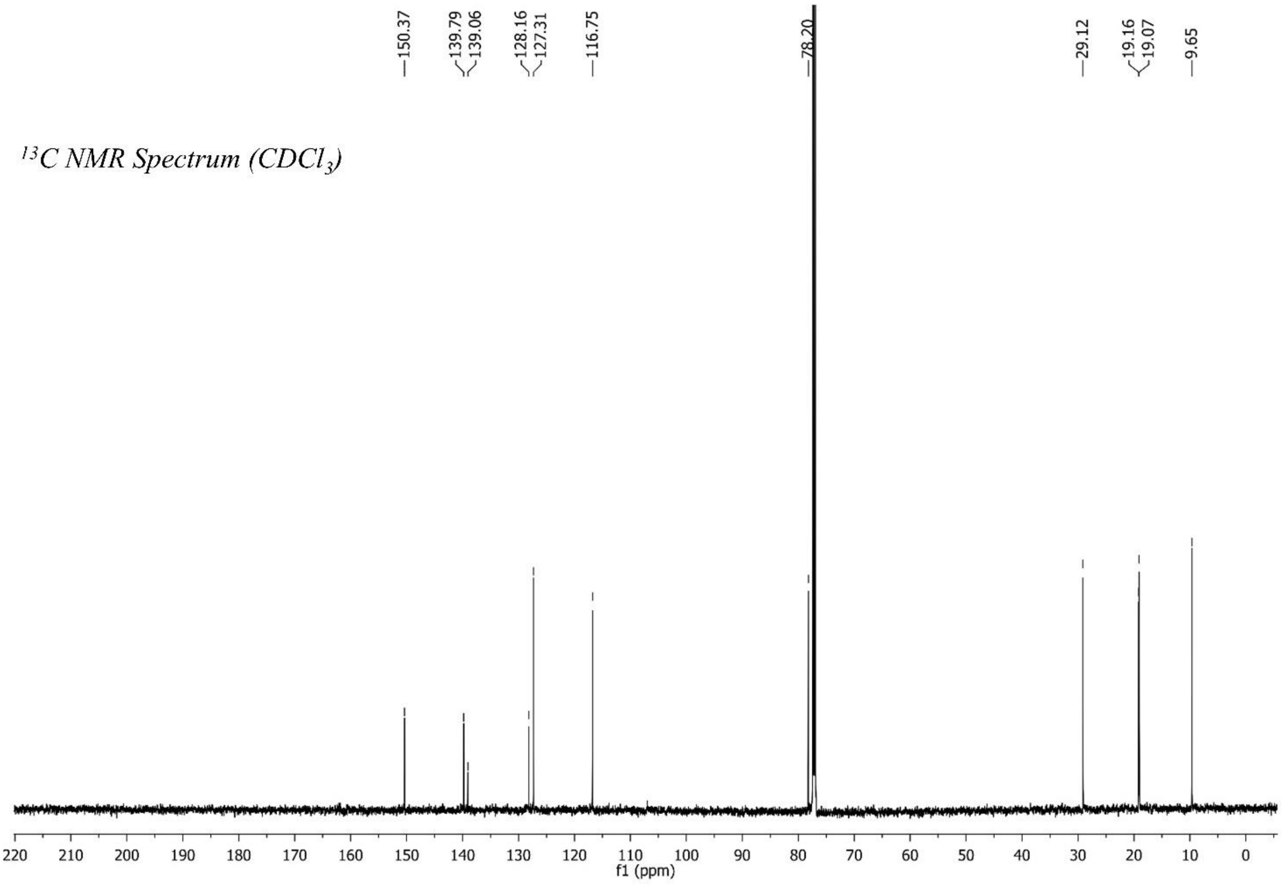

1-Chloro-2-methyl-4-nitro-5-(pentan-3-yloxy)benzene (**75**)

^{1}H NMR Spectrum (CDCl$_3$)

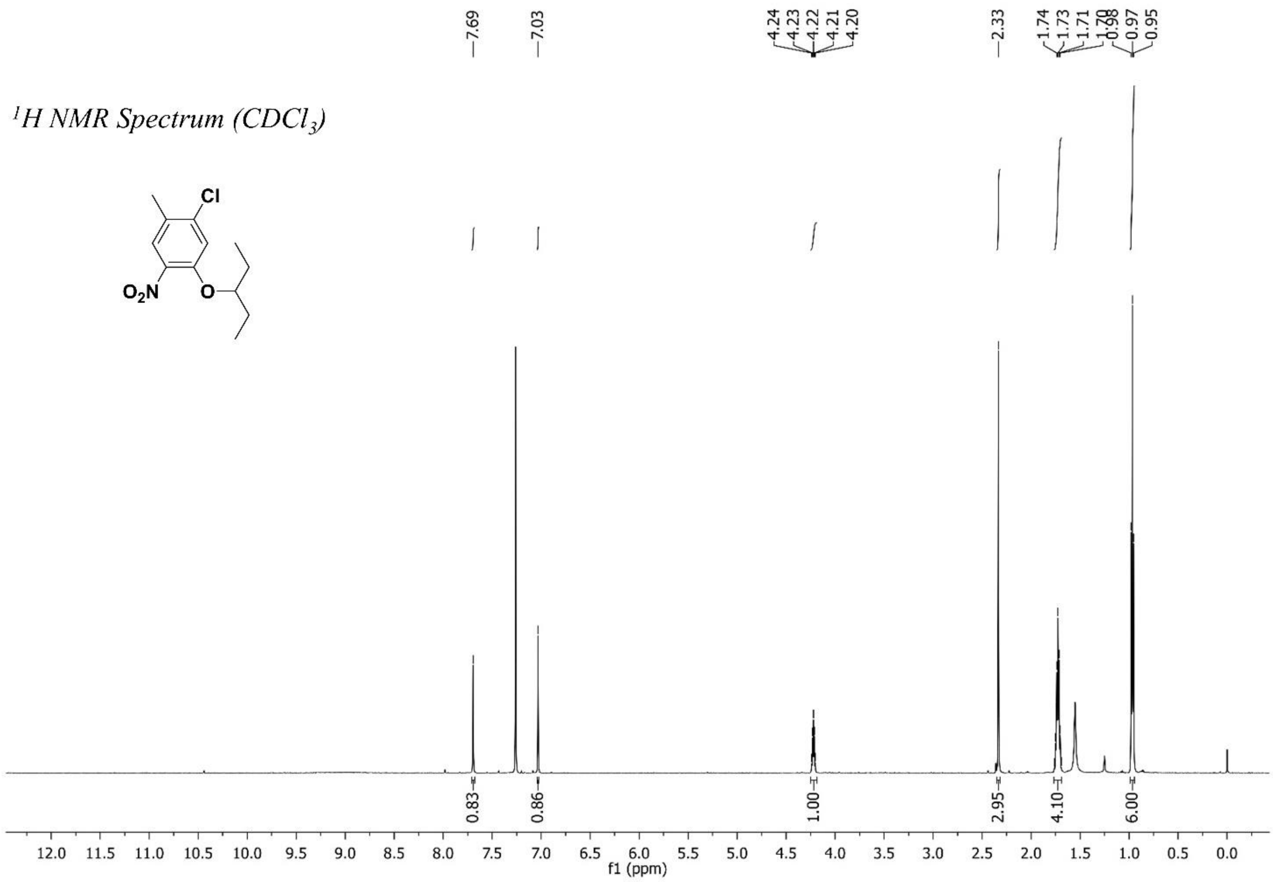

^{13}C NMR Spectrum (CDCl$_3$)

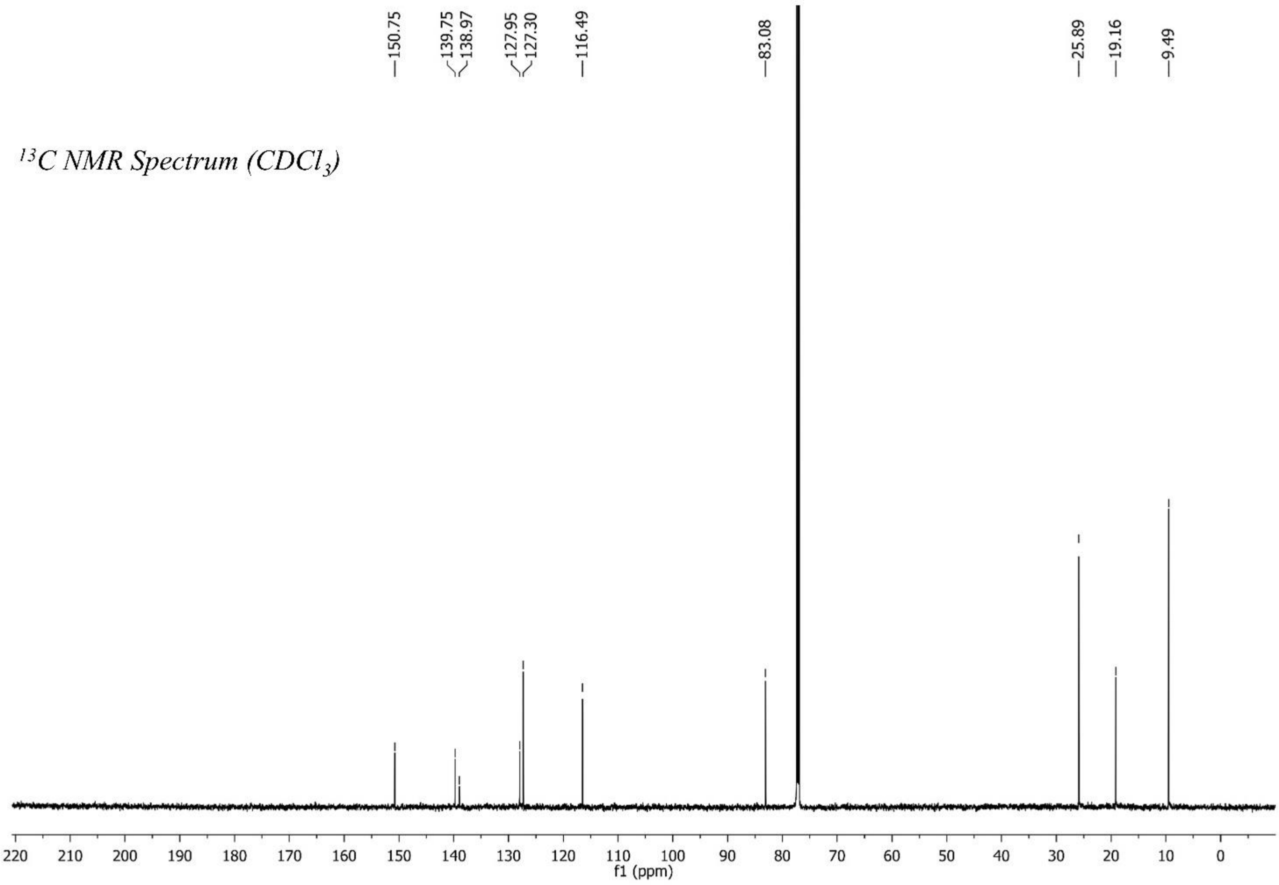

1-Chloro-2-methyl-4-nitro-5-(pentan-2-yloxy)benzene (**76**)

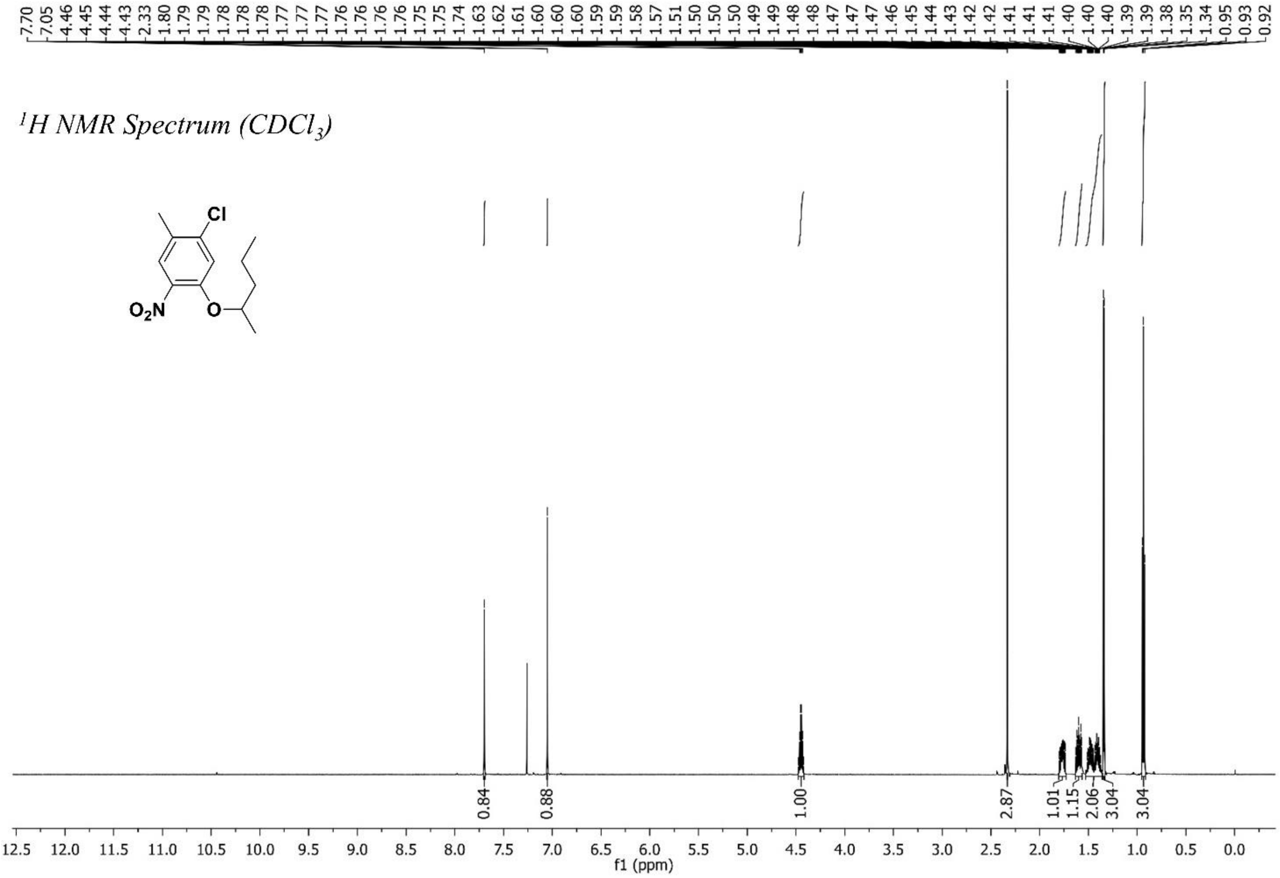

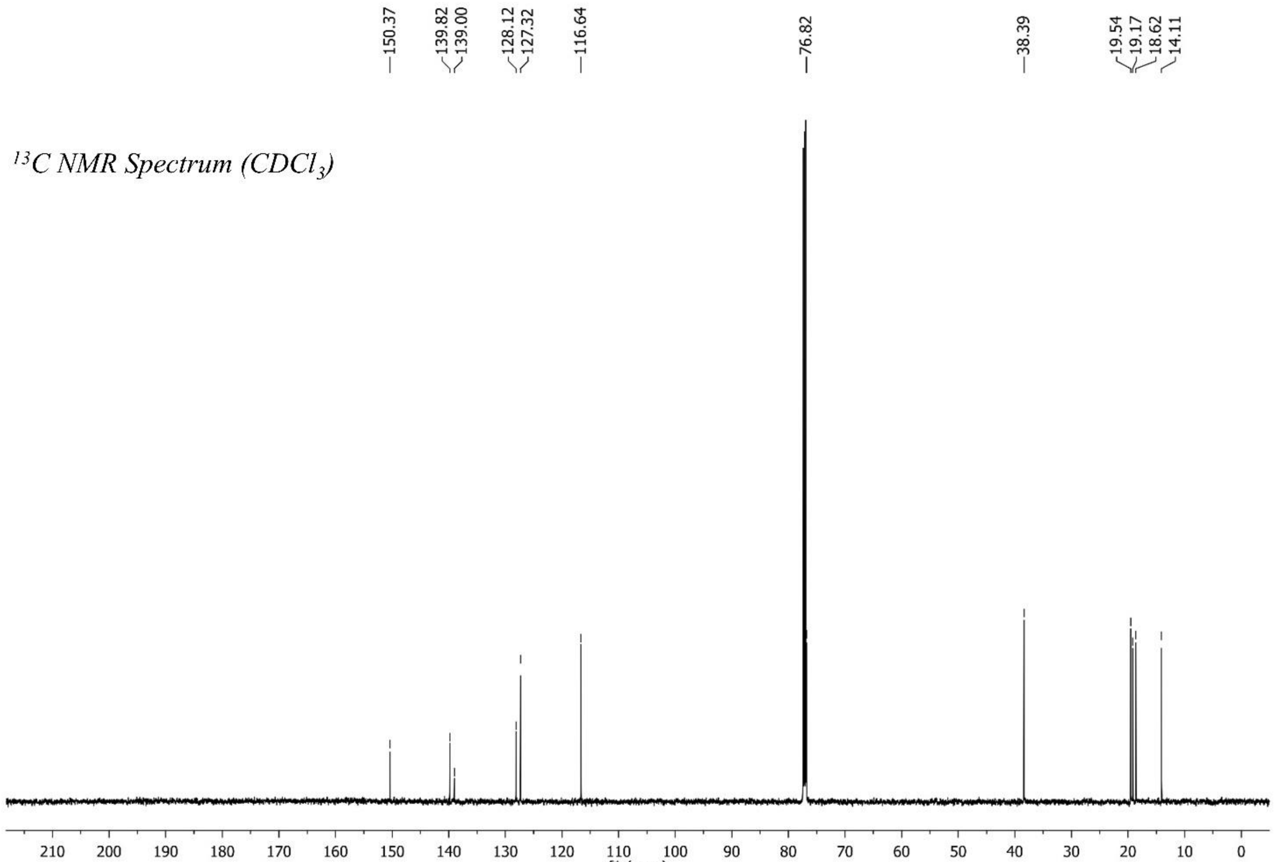

1-Isopropoxy-4-methyl-2-nitro-5-phenoxybenzene (**77**)

^{1}H NMR Spectrum (CDCl$_3$)

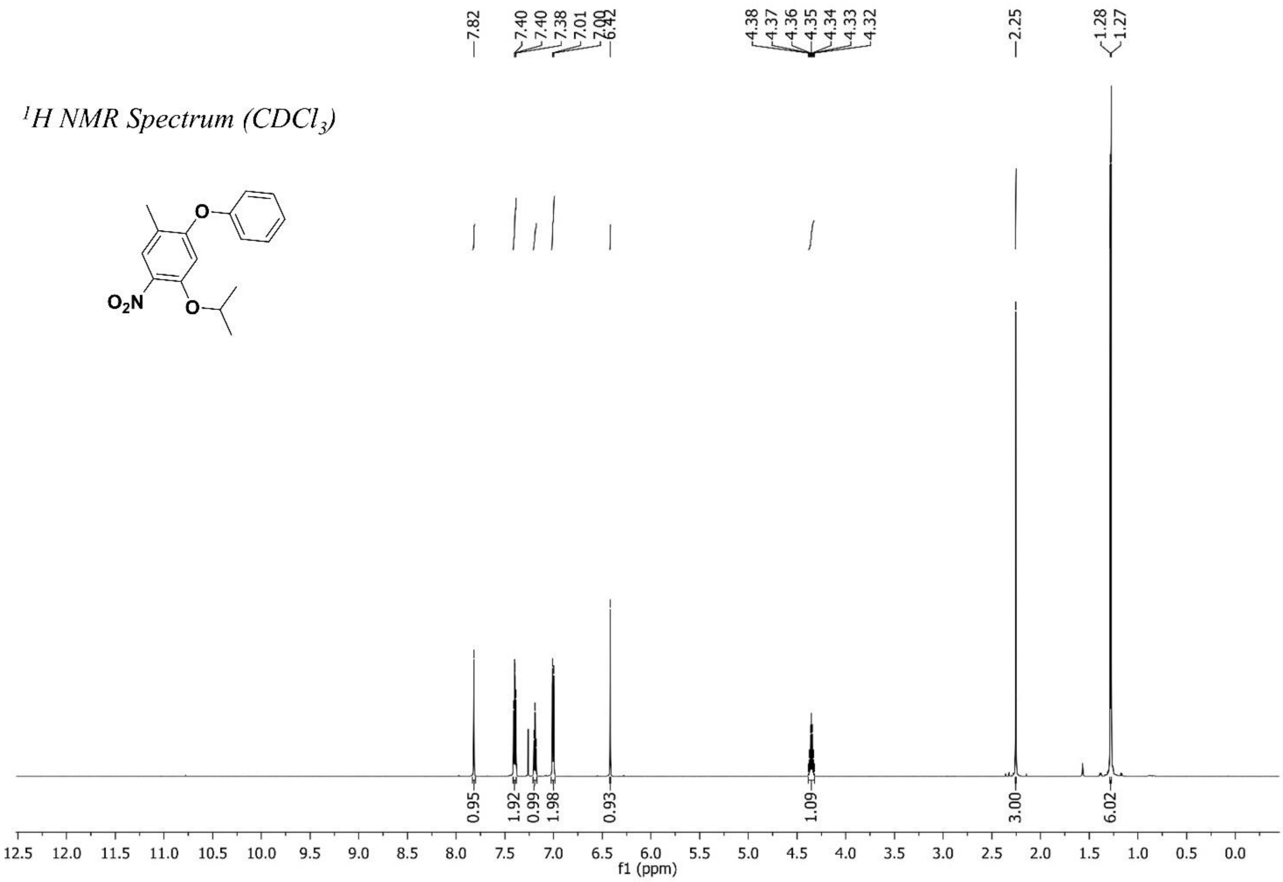

^{13}C NMR Spectrum (CDCl$_3$)

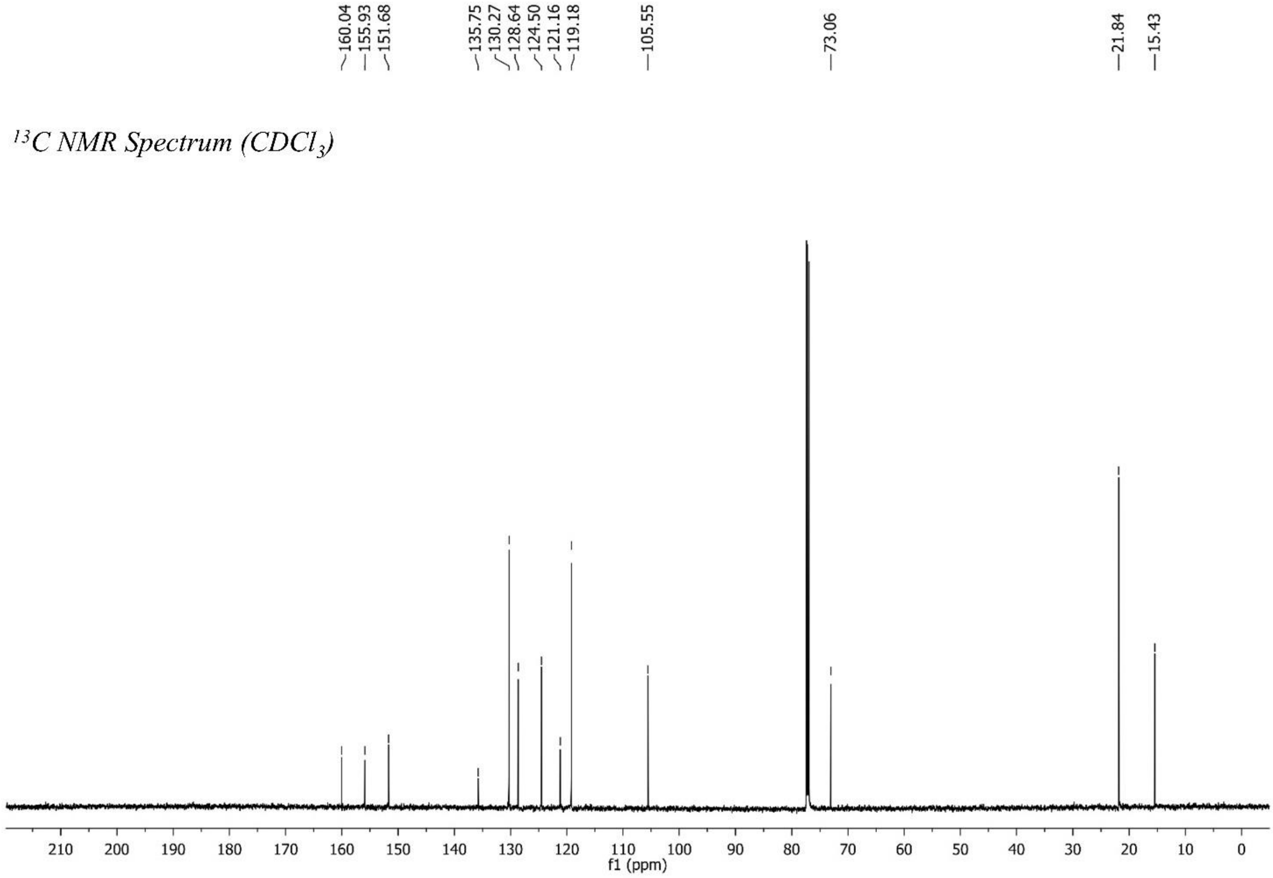

1-Cyclobutoxy-4-methyl-2-nitro-5-phenoxybenzene (**78**)

^1H NMR Spectrum ($CDCl_3$)

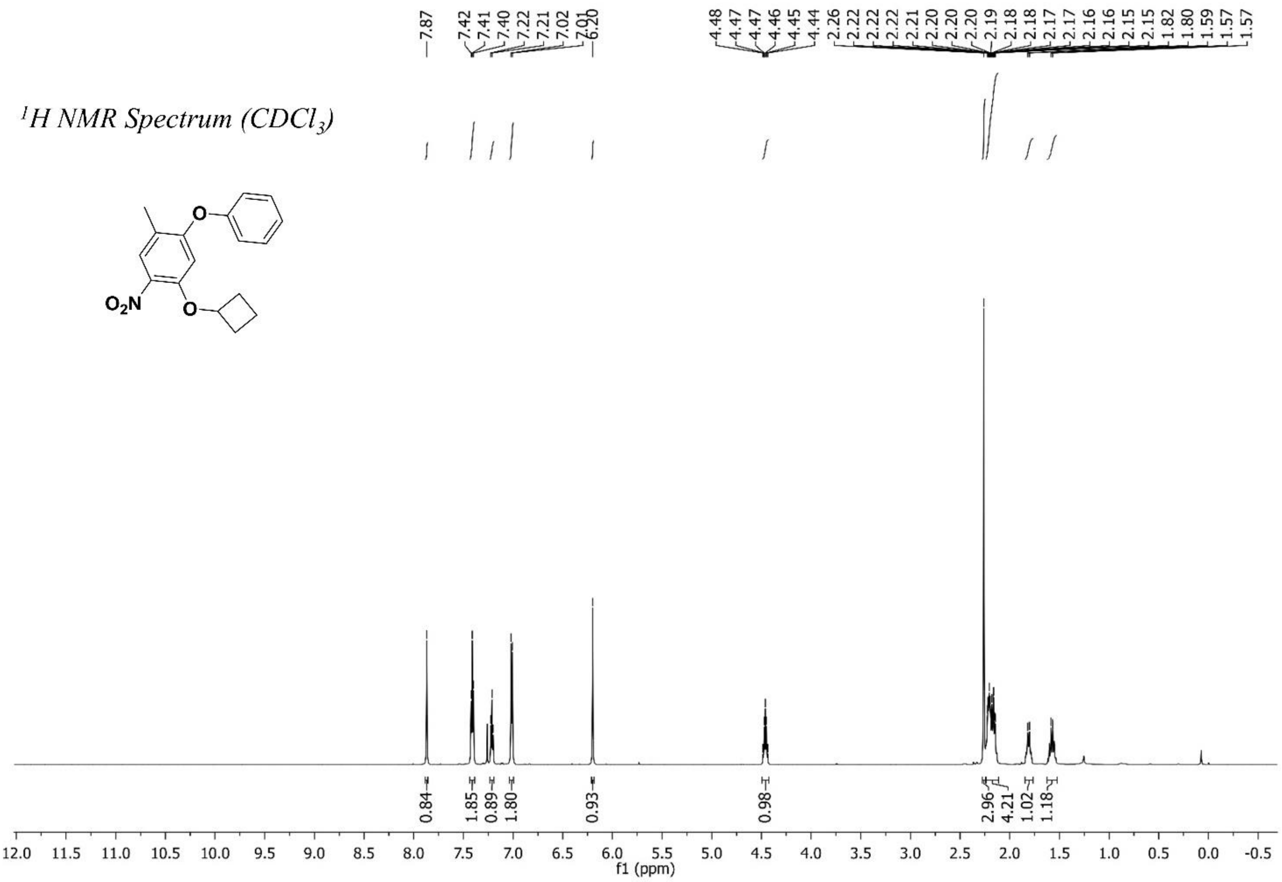

^{13}C NMR Spectrum ($CDCl_3$)

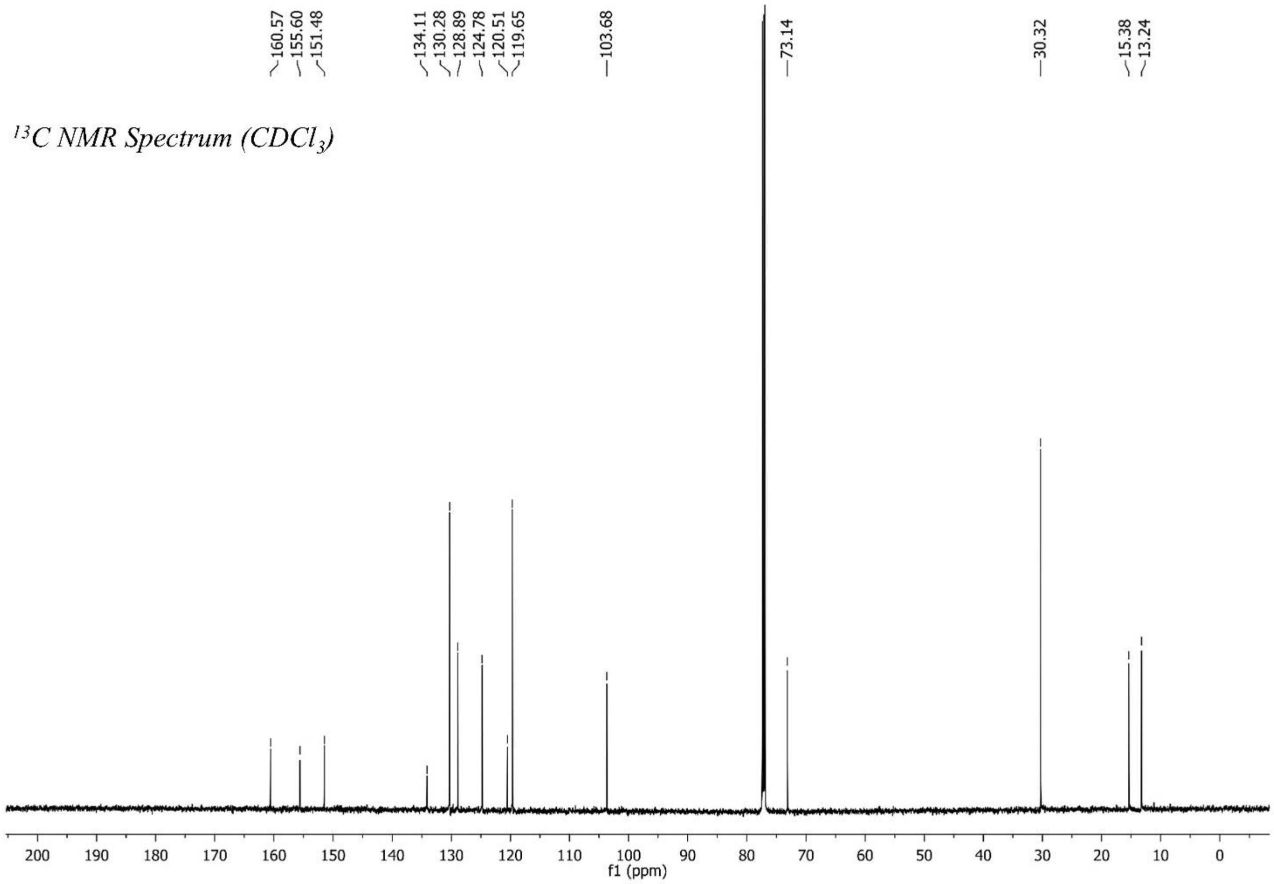

1-(Sec-butoxy)-4-methyl-2-nitro-5-phenoxybenzene (79)

^{1}H NMR Spectrum (CDCl$_3$)

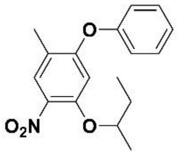

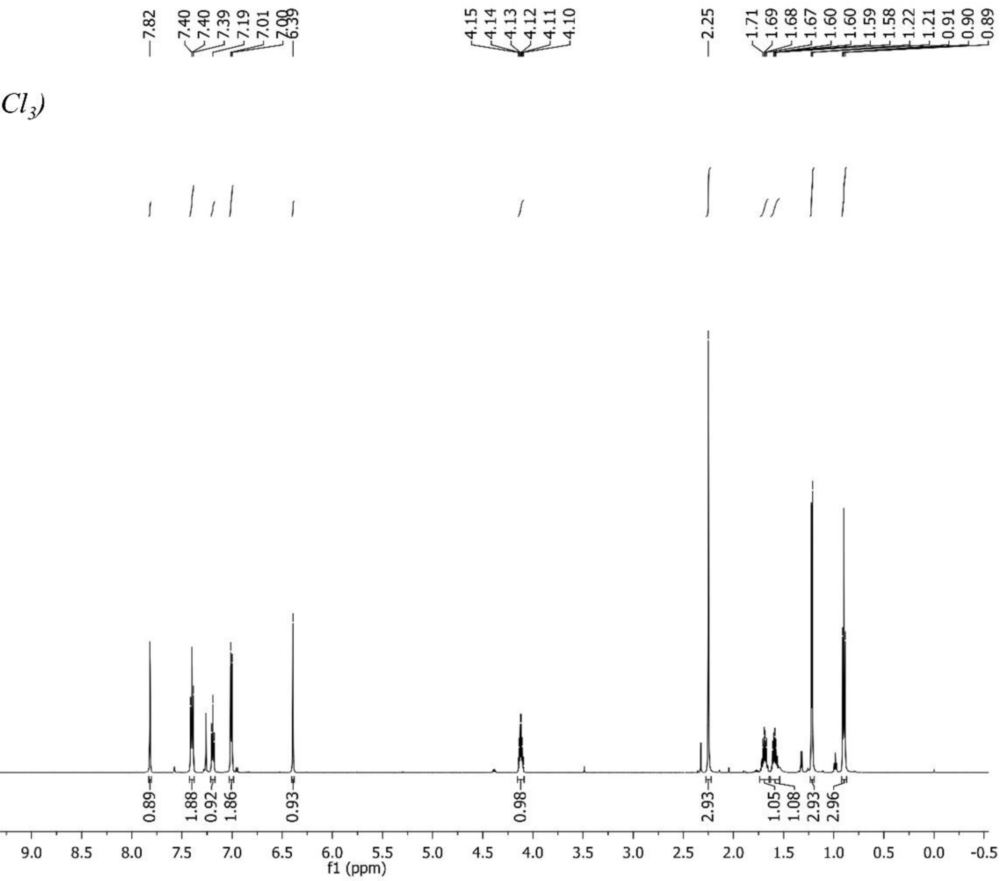

^{13}C NMR Spectrum (CDCl$_3$)

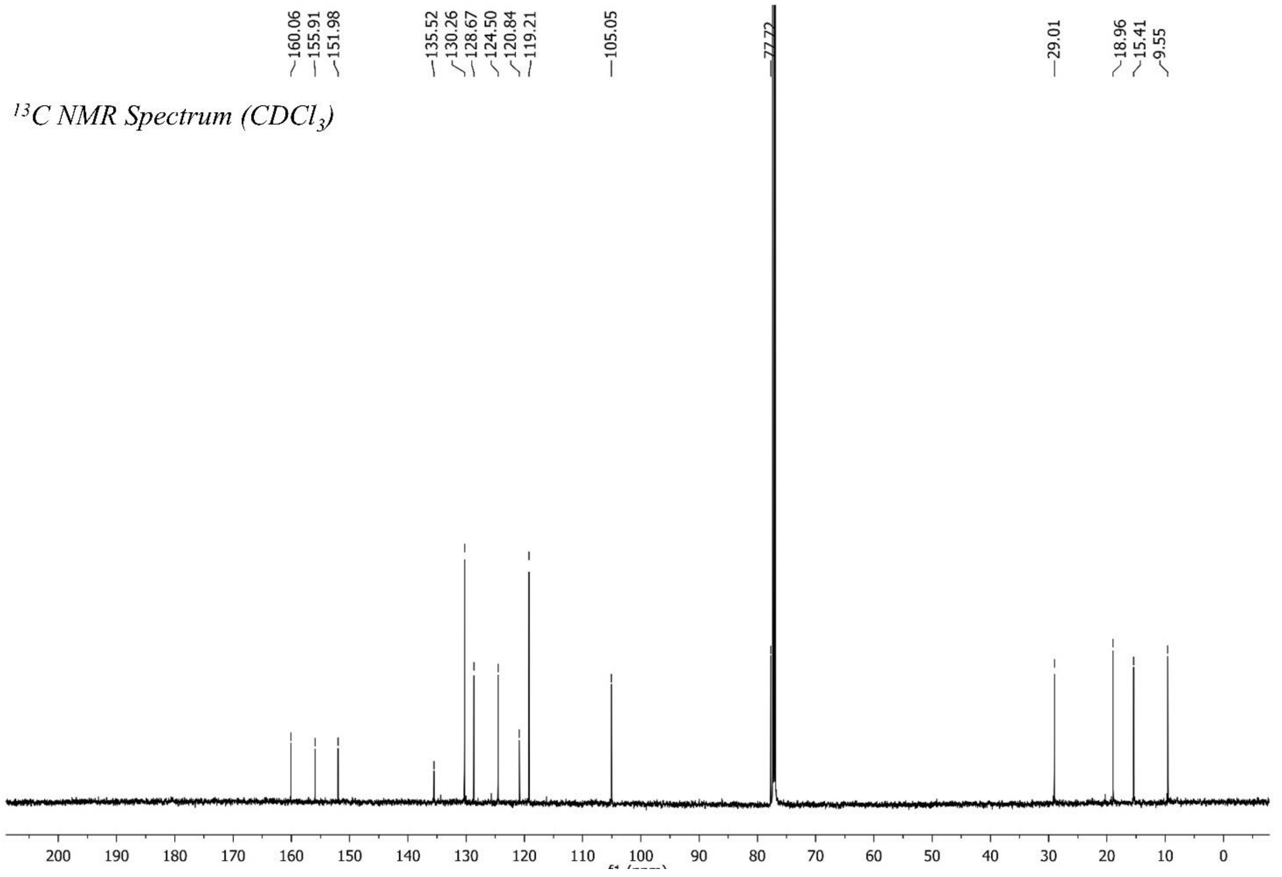

1-Methyl-5-nitro-4-(pentan-3-yloxy)-2-phenoxybenzene (**80**)

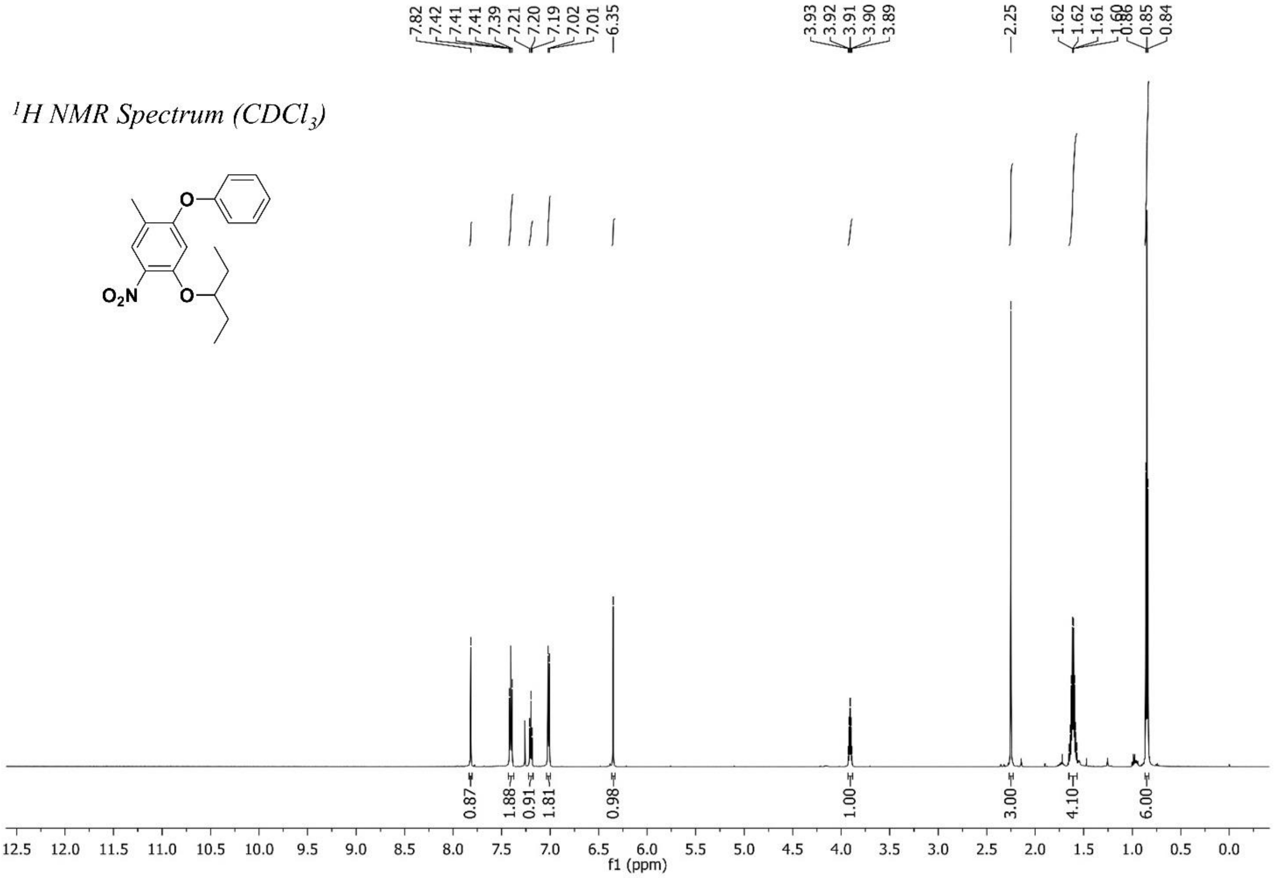

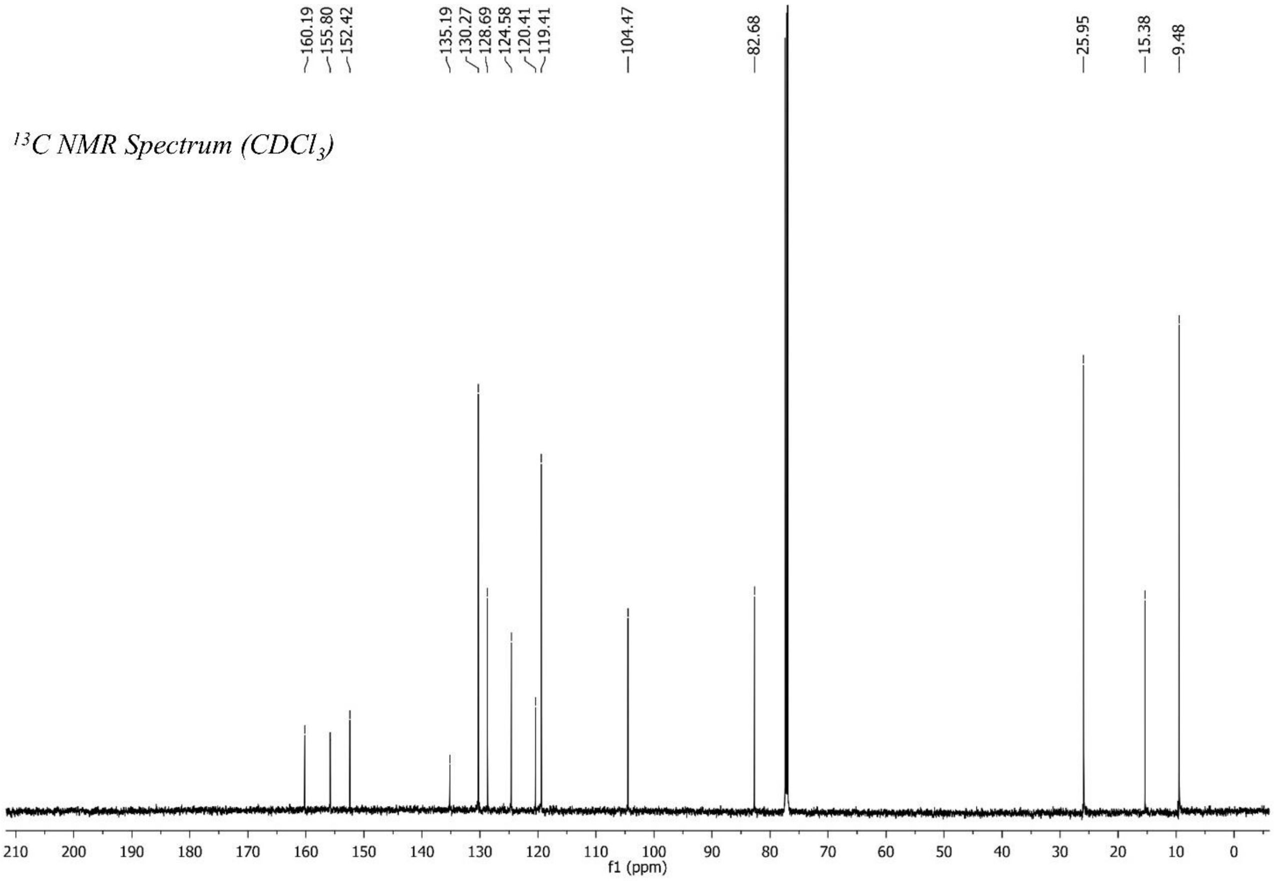

1-Methyl-5-nitro-4-(pentan-2-yloxy)-2-phenoxybenzene (**81**)

^{1}H NMR Spectrum (CDCl$_3$)

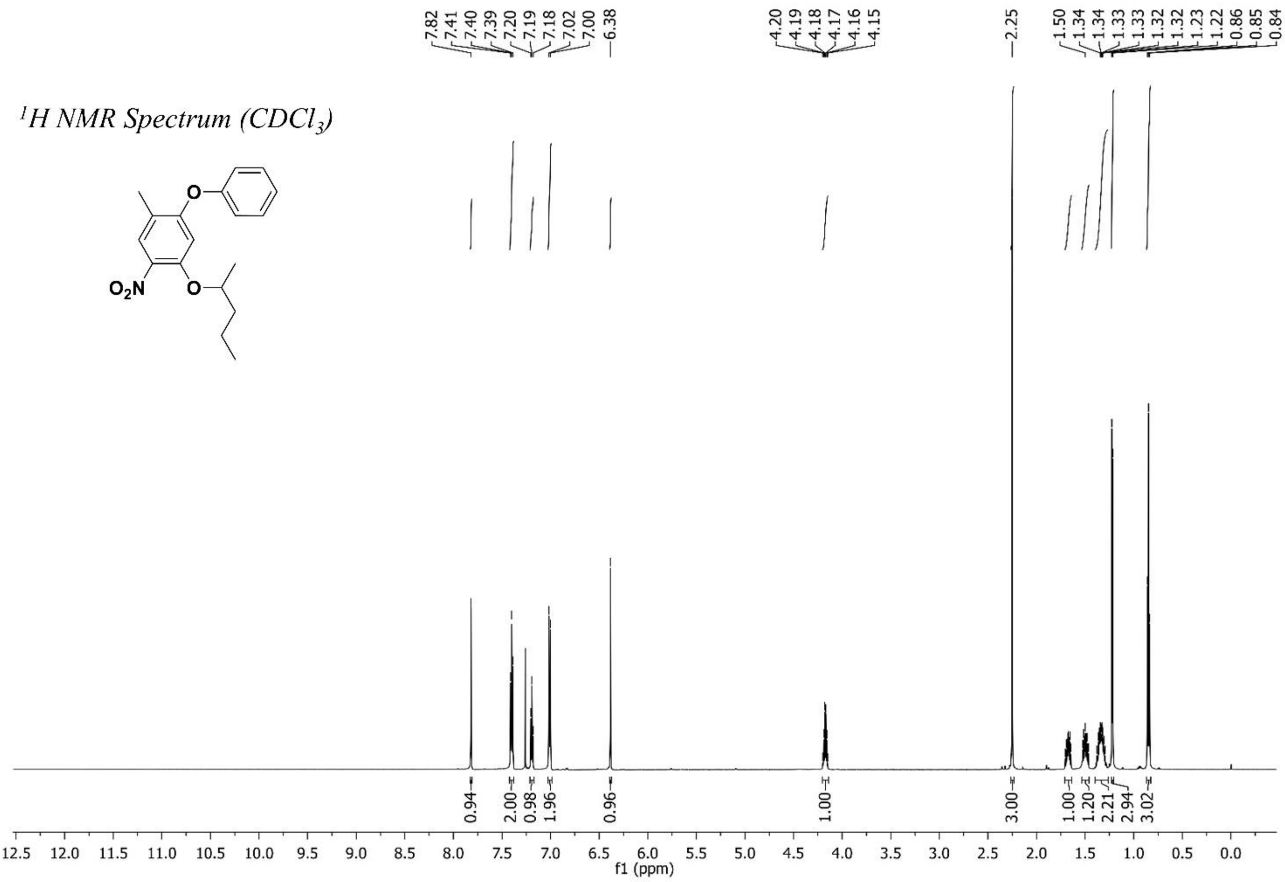

^{13}C NMR Spectrum (CDCl$_3$)

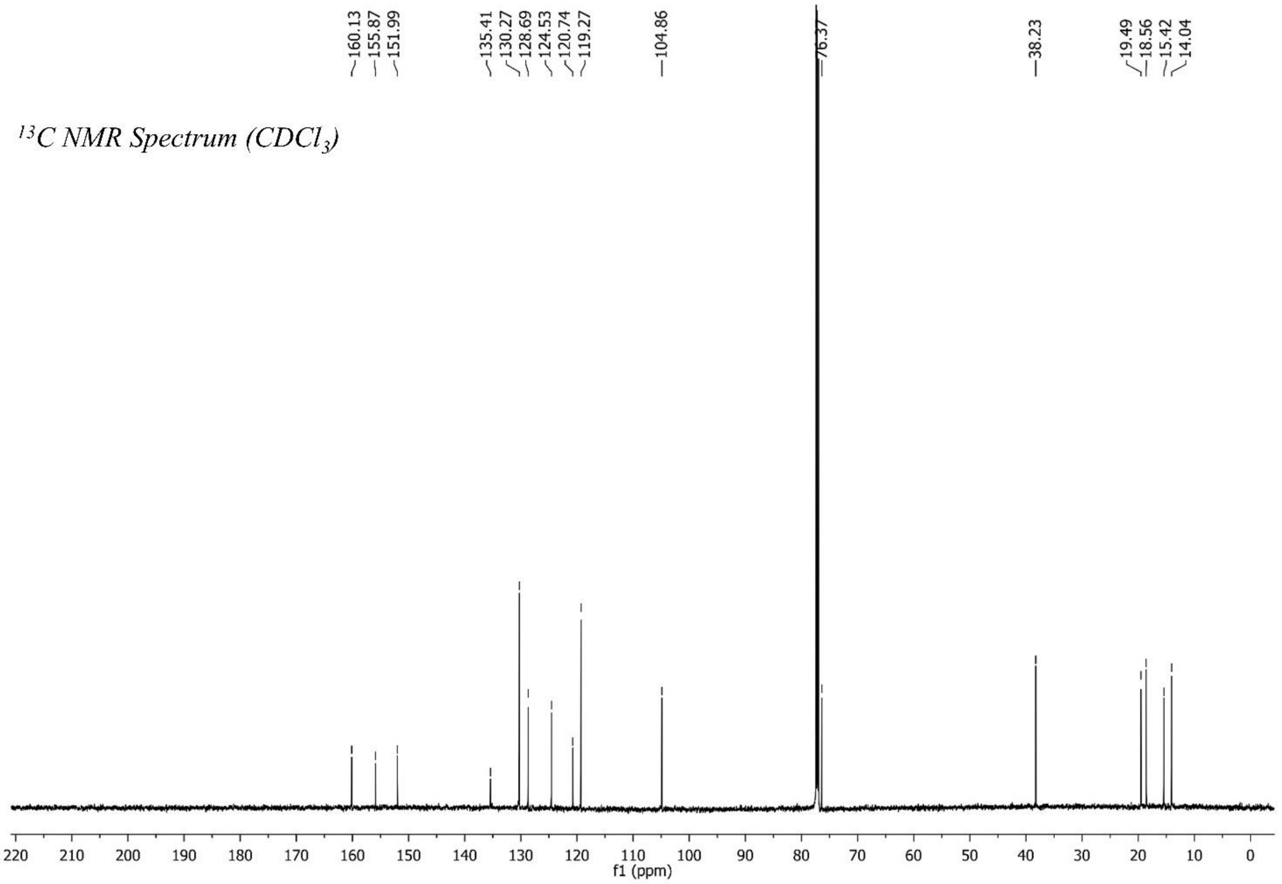

2-Isopropoxy-5-methyl-4-phenoxyaniline (**49**)

^{1}H NMR Spectrum ($CDCl_3$)

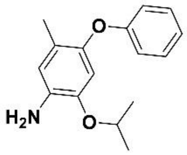

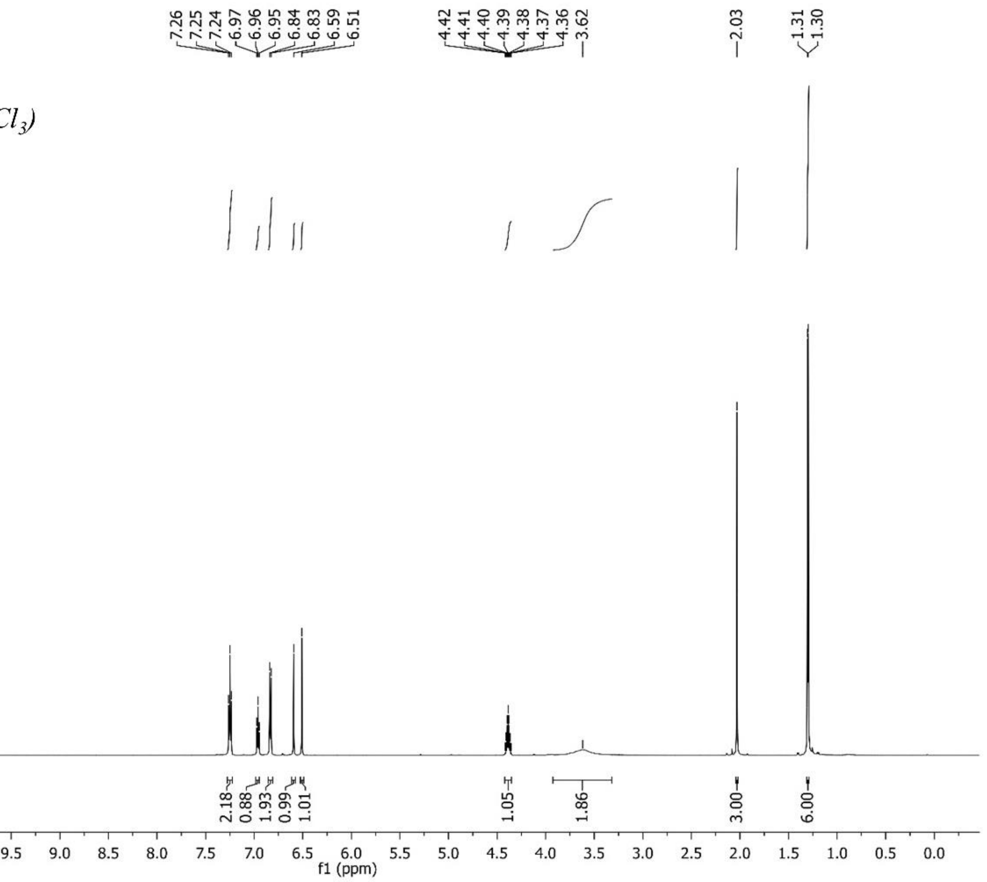

^{13}C NMR Spectrum ($CDCl_3$)

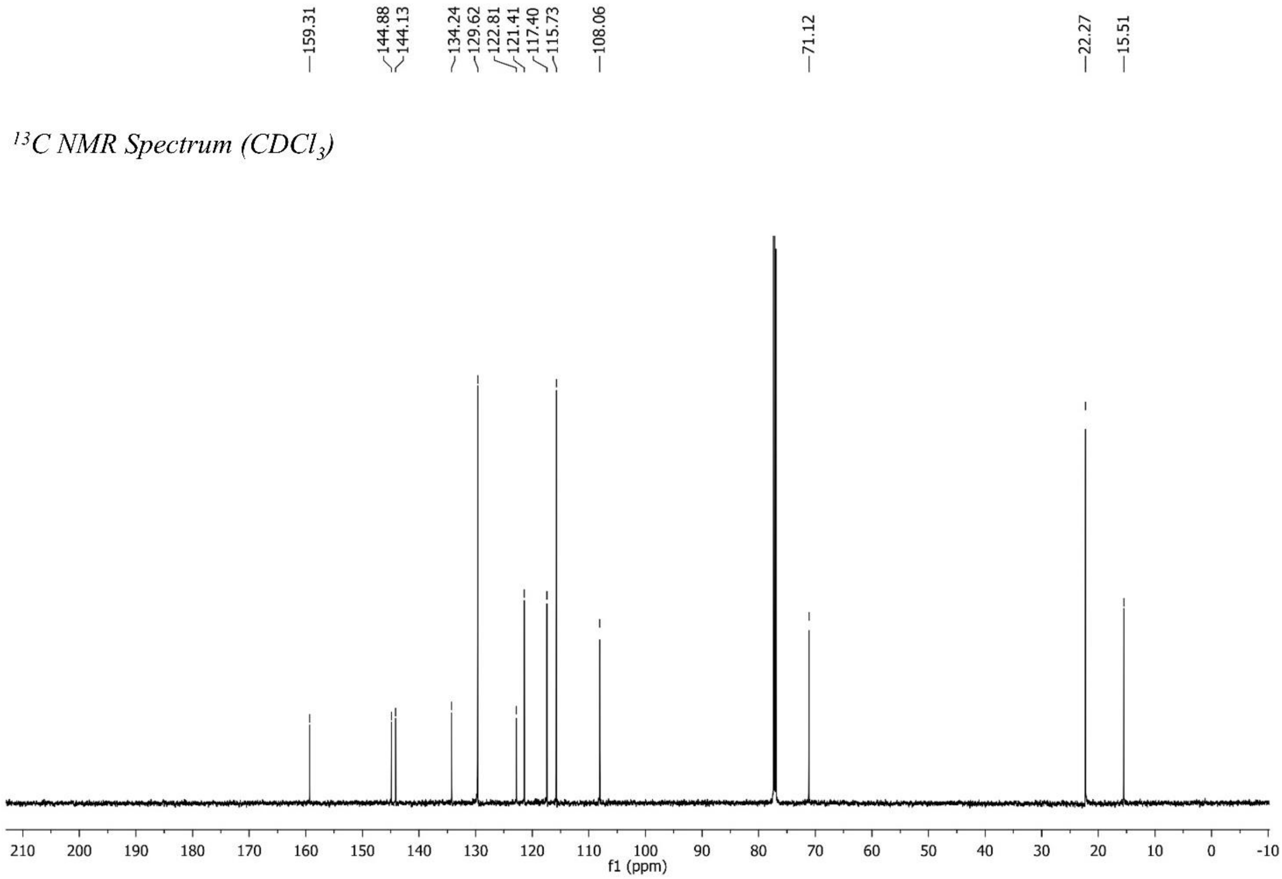

2-Cyclobutoxy-5-methyl-4-phenoxyaniline (**50**)

^{1}H NMR Spectrum ($CDCl_3$)

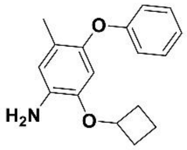

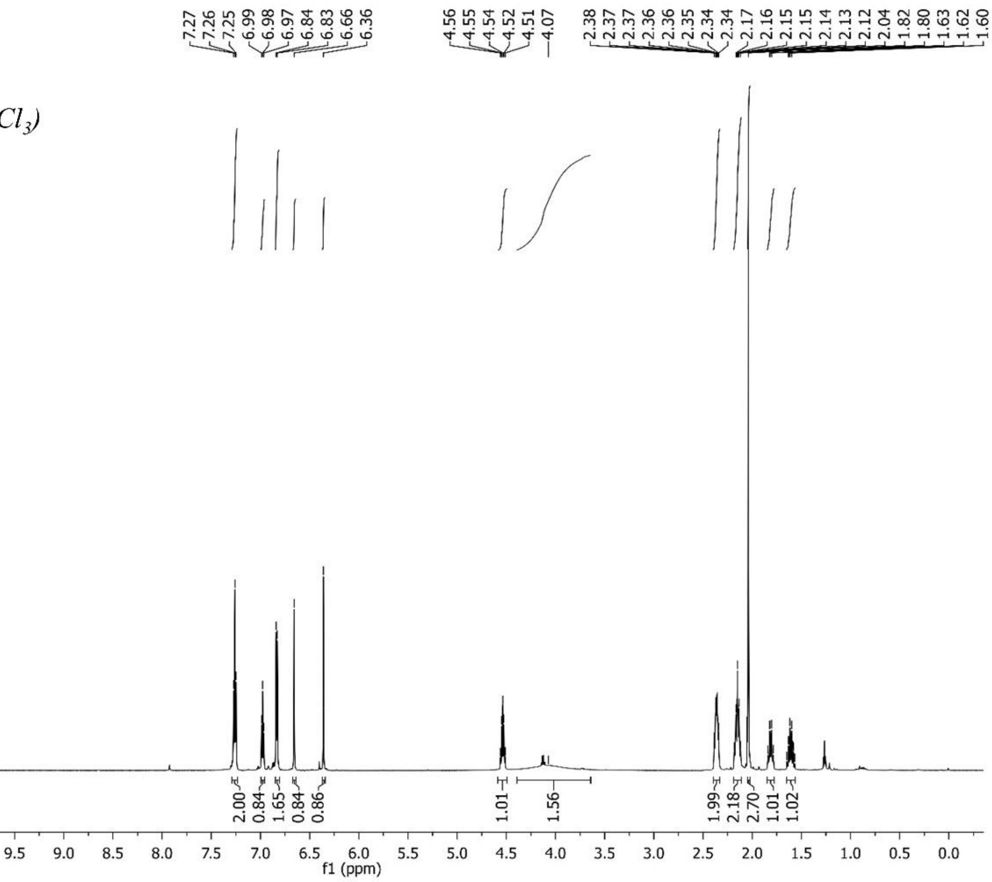

^{13}C NMR Spectrum ($CDCl_3$)

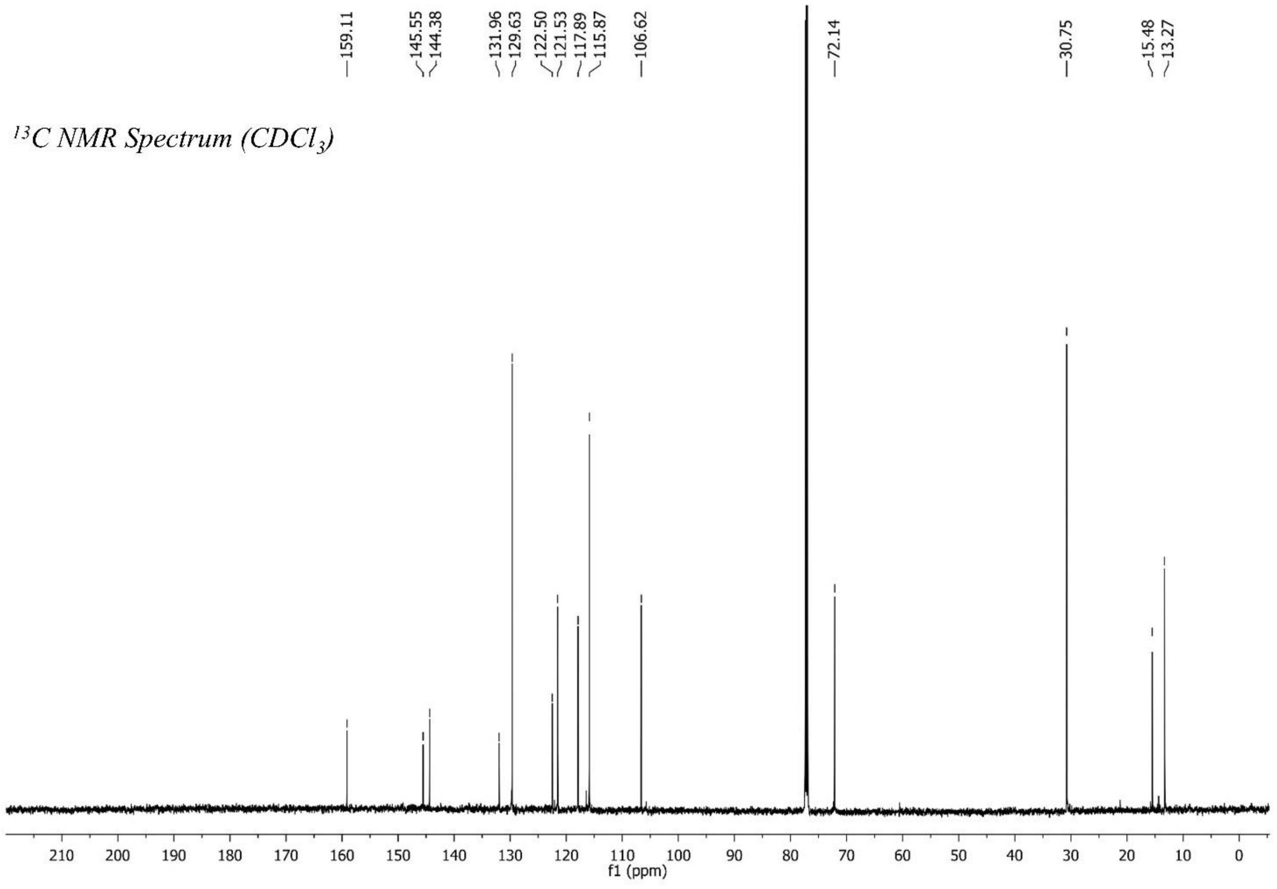

2-(Sec-butoxy)-5-methyl-4-phenoxyaniline (51)

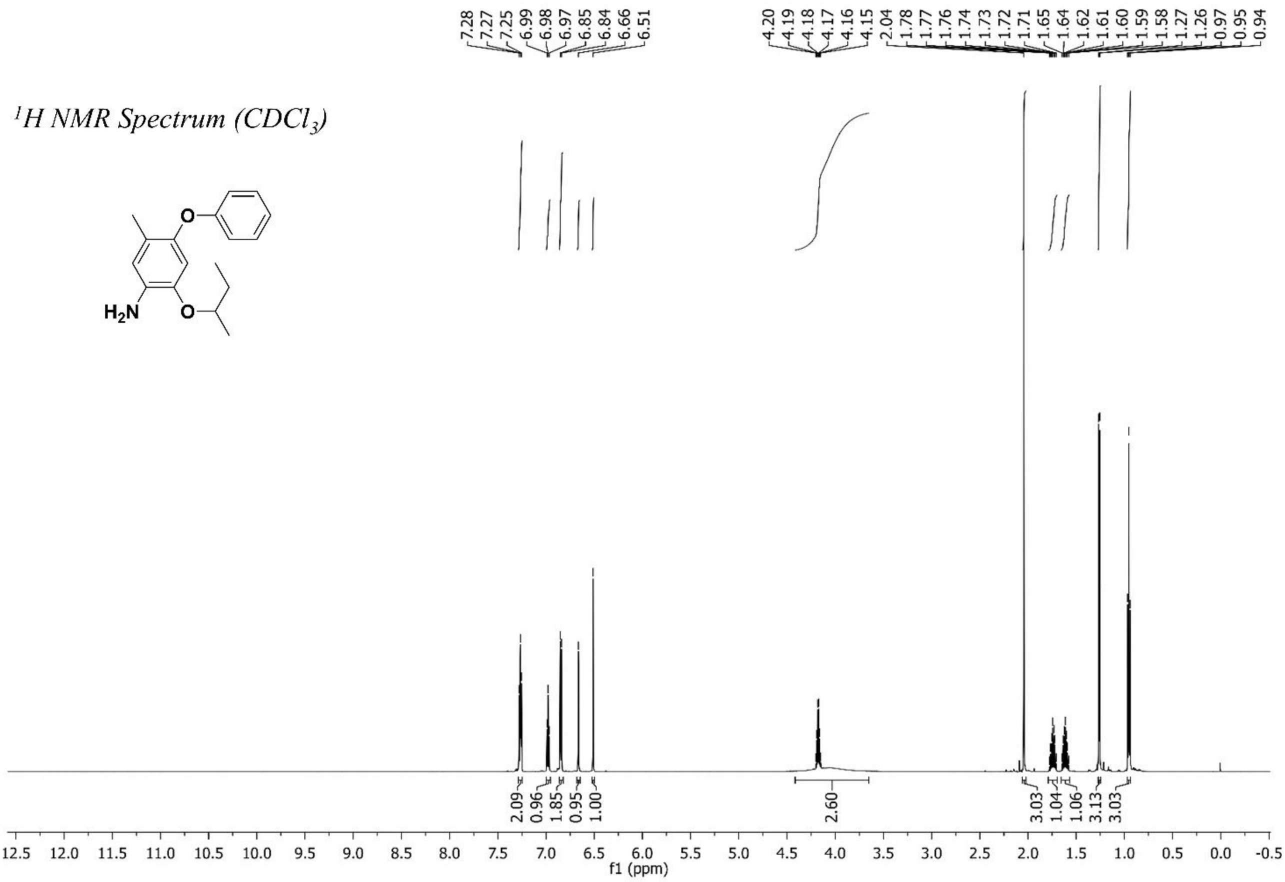

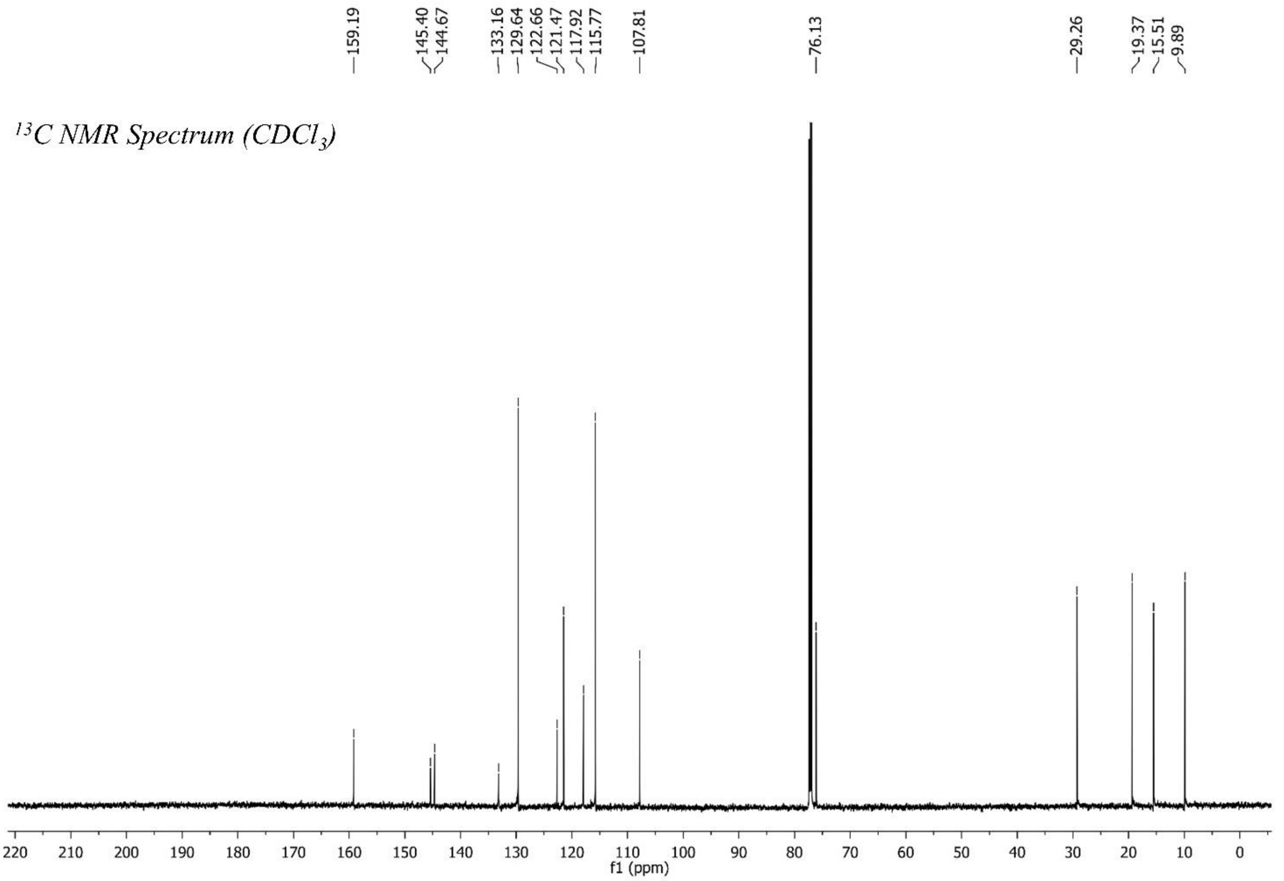

5-Methyl-2-(pentan-3-yloxy)-4-phenoxyaniline (**52**)

^{1}H NMR Spectrum (CDCl$_3$)

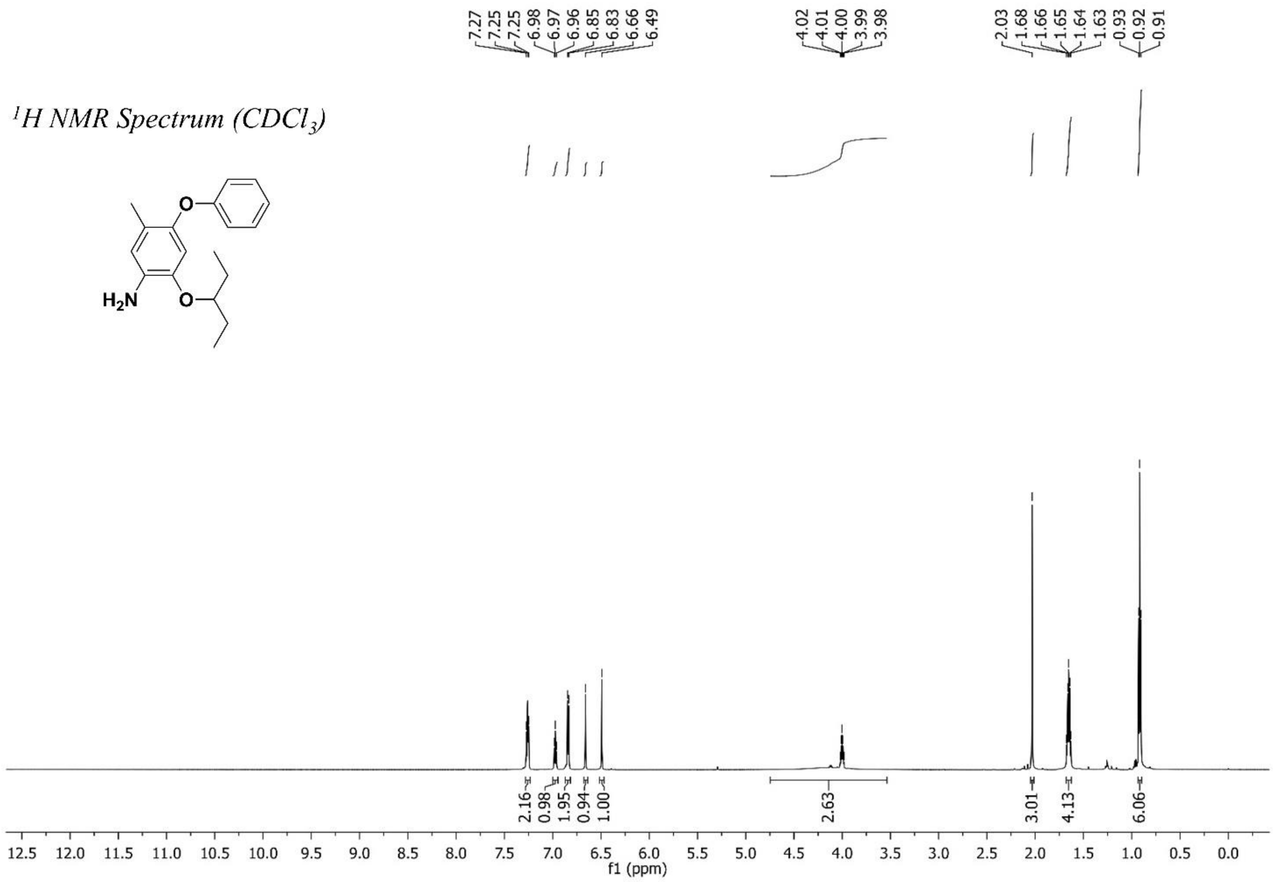

^{13}C NMR Spectrum (CDCl$_3$)

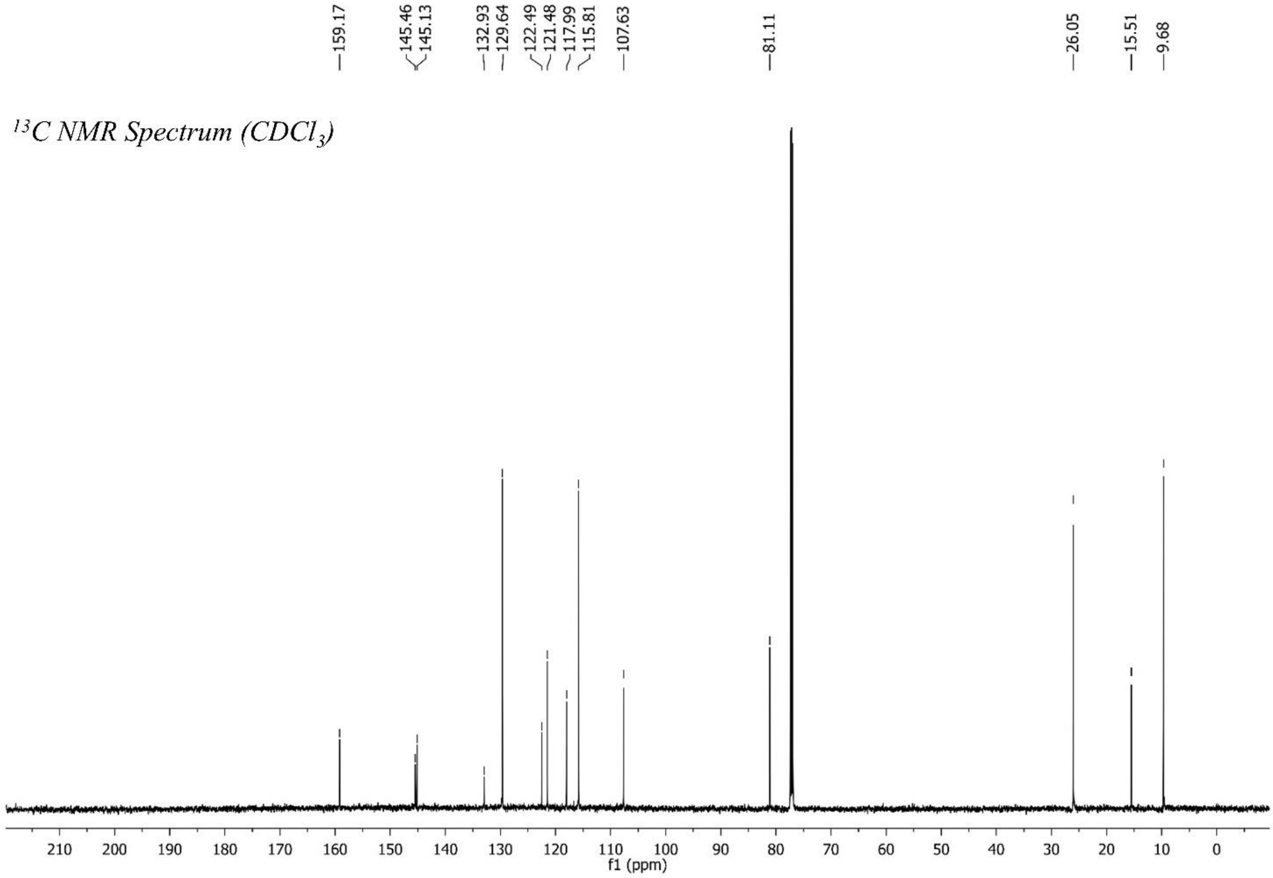

5-Methyl-2-(pentan-2-yloxy)-4-phenoxyaniline (**53**)

^{1}H NMR Spectrum (CDCl$_3$)

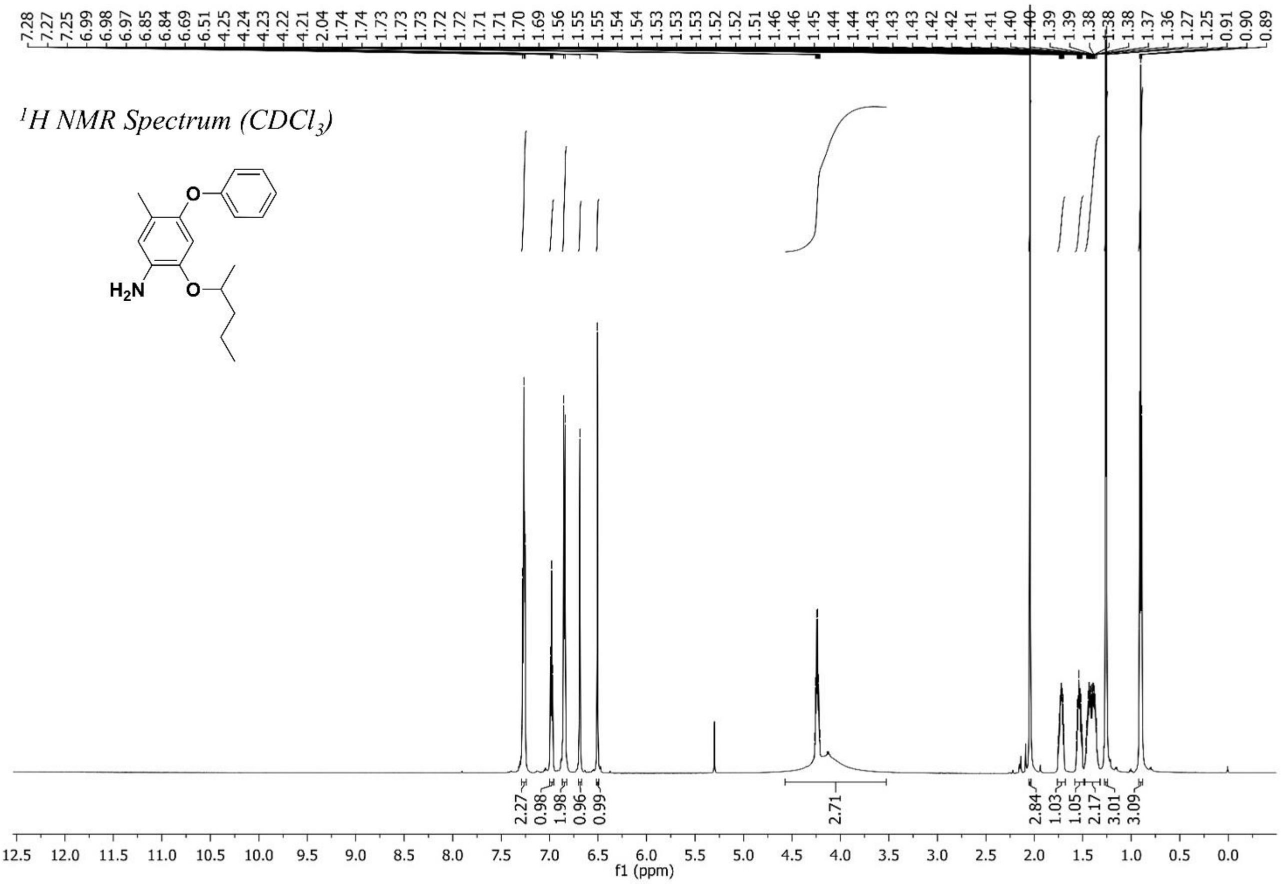

^{13}C NMR Spectrum (CDCl$_3$)

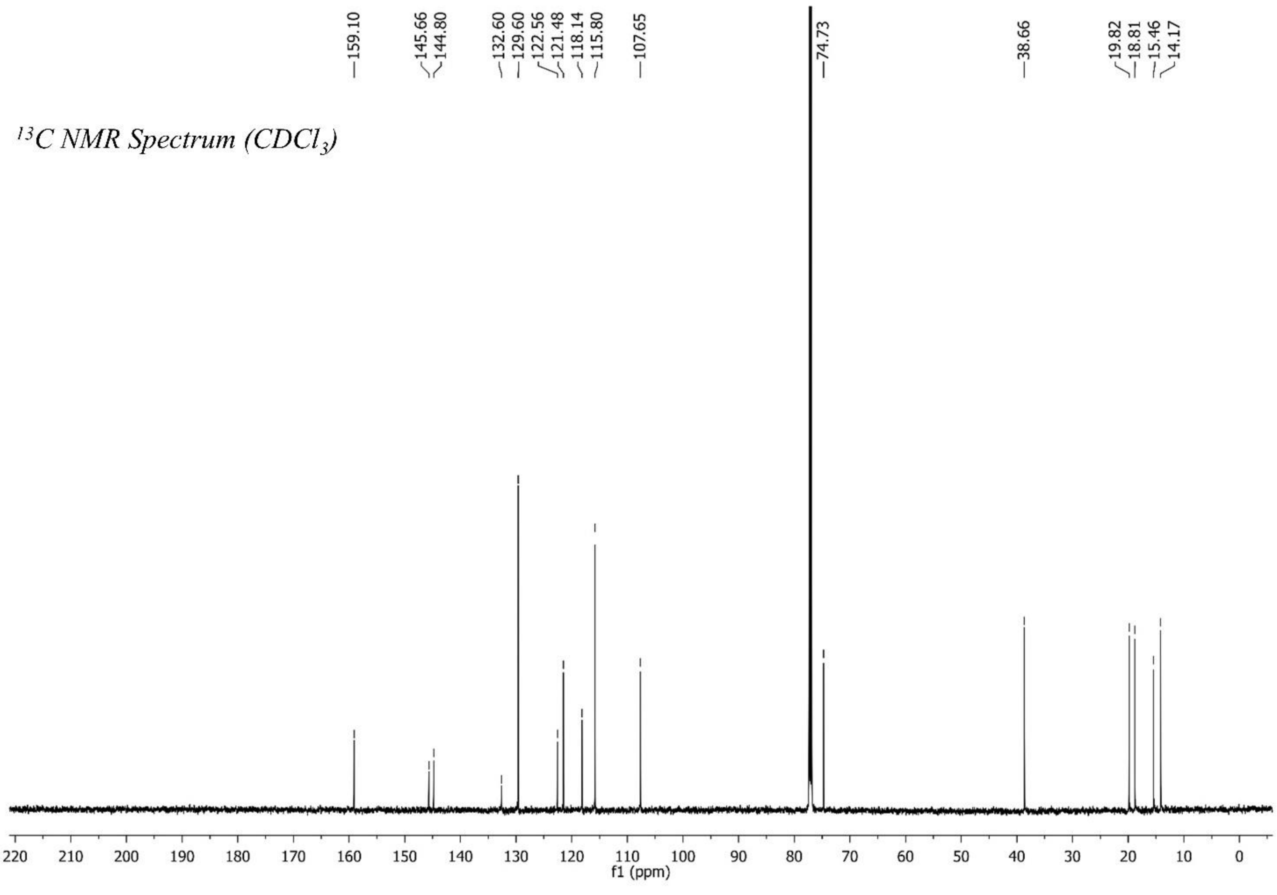

2,4-Dinitro-N-phenylaniline (**83**)

^{1}H NMR Spectrum ($CDCl_3$)

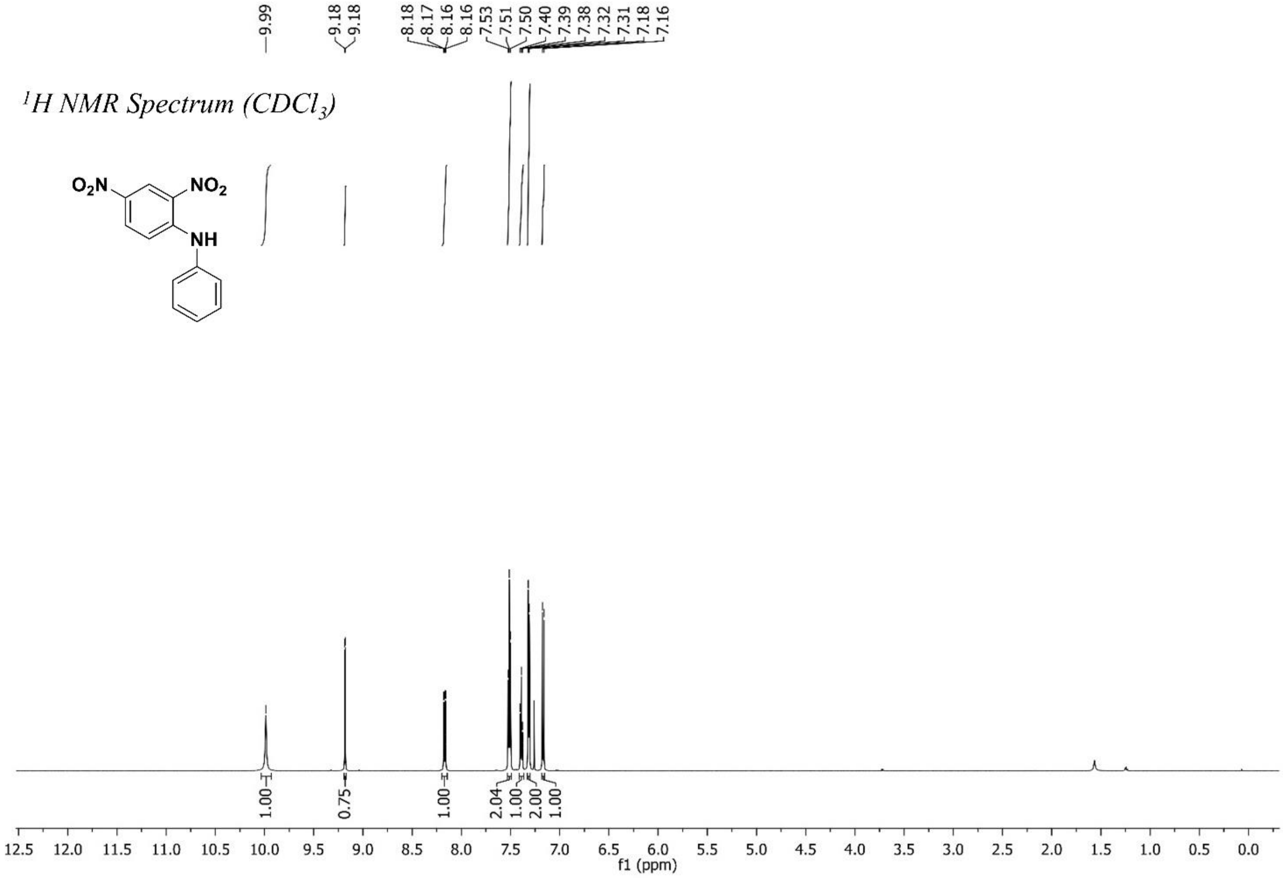

^{13}C NMR Spectrum ($CDCl_3$)

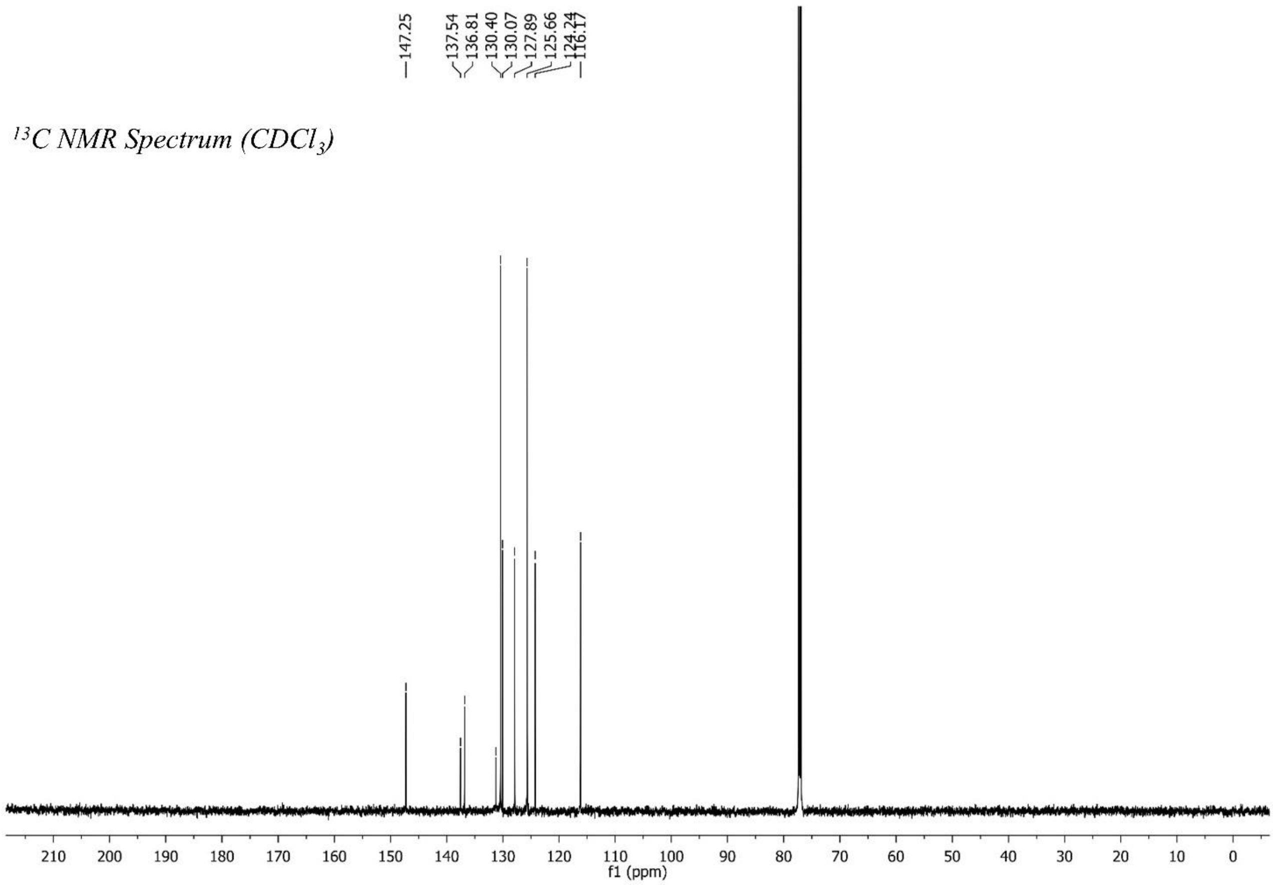

4-Nitro-N1-phenylbenzene-1,2-diamine (**84**)

^{1}H NMR Spectrum ($CDCl_3$)

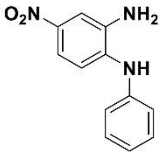

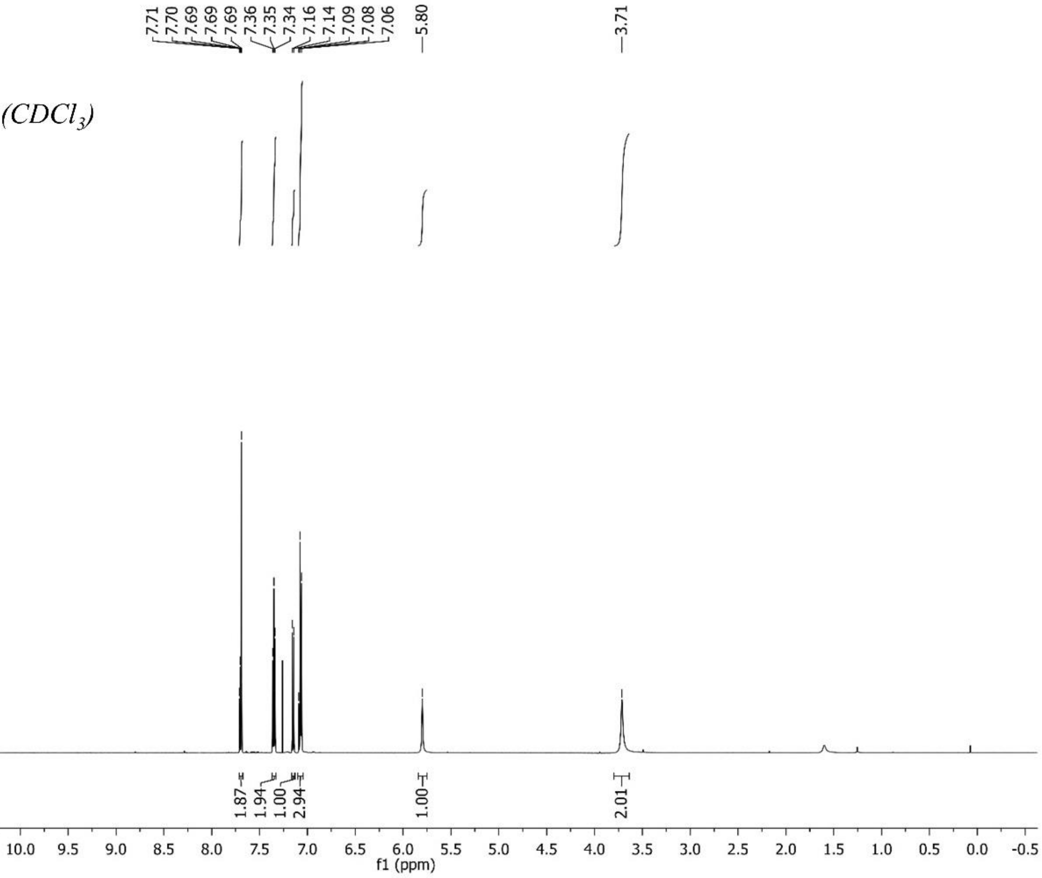

^{13}C NMR Spectrum ($CDCl_3$)

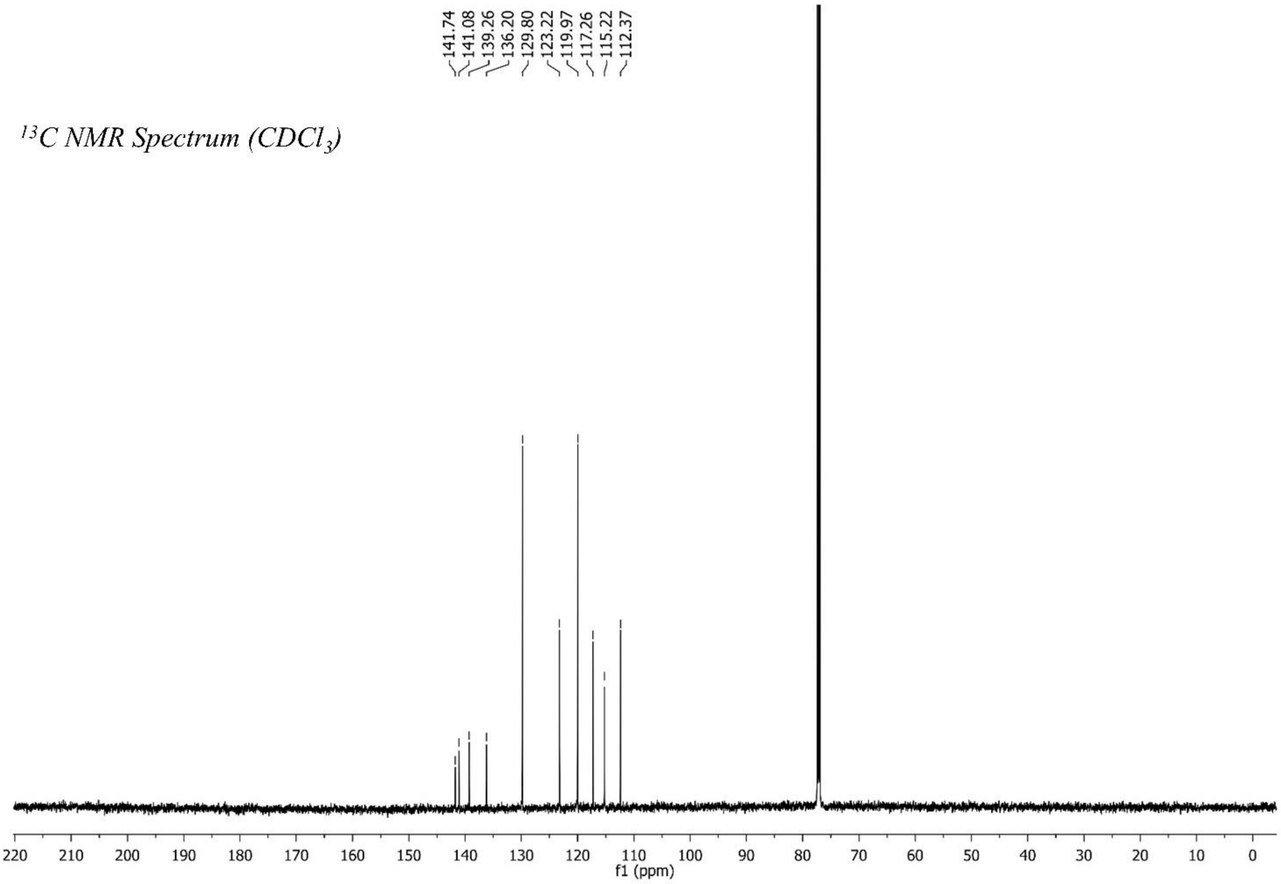

N1-Phenylbenzene-1,2,4-triamine (**85**)

^{1}H NMR Spectrum ($CDCl_3$)

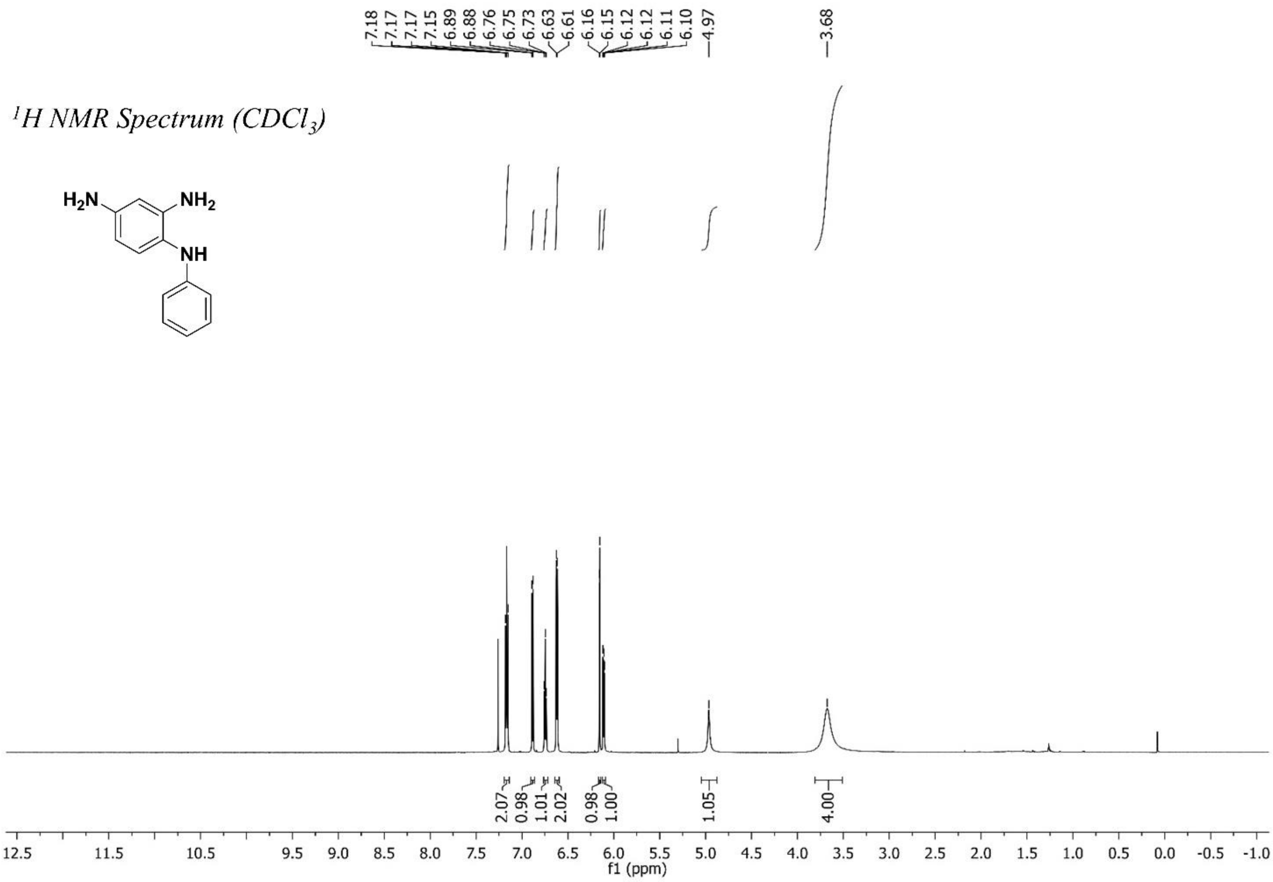

^{13}C NMR Spectrum ($CDCl_3$)

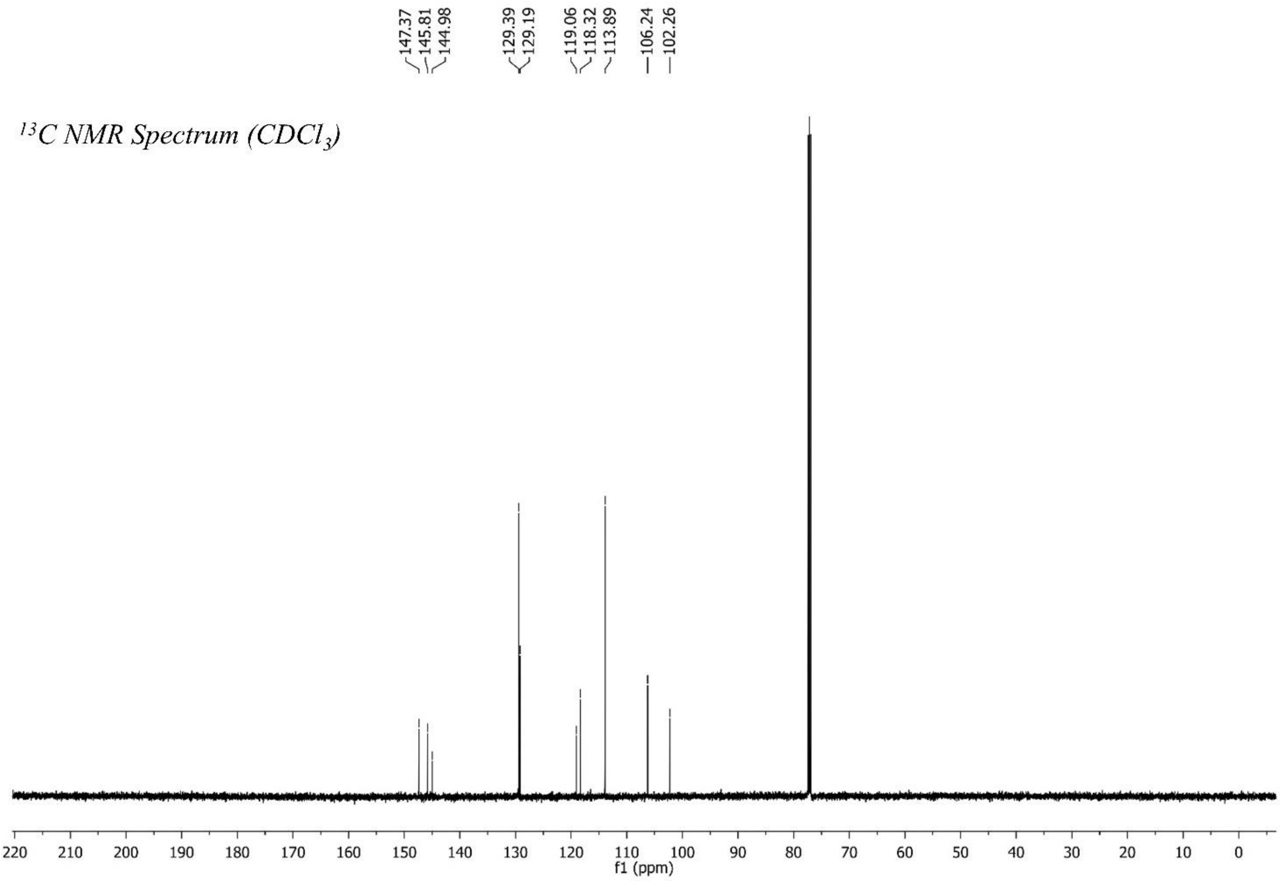

5-Nitro-1-phenyl-1H-benzo[d][1,2,3]triazole (**86**)

^{1}H NMR Spectrum ($CDCl_3$)

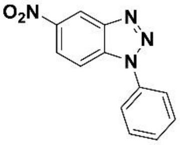

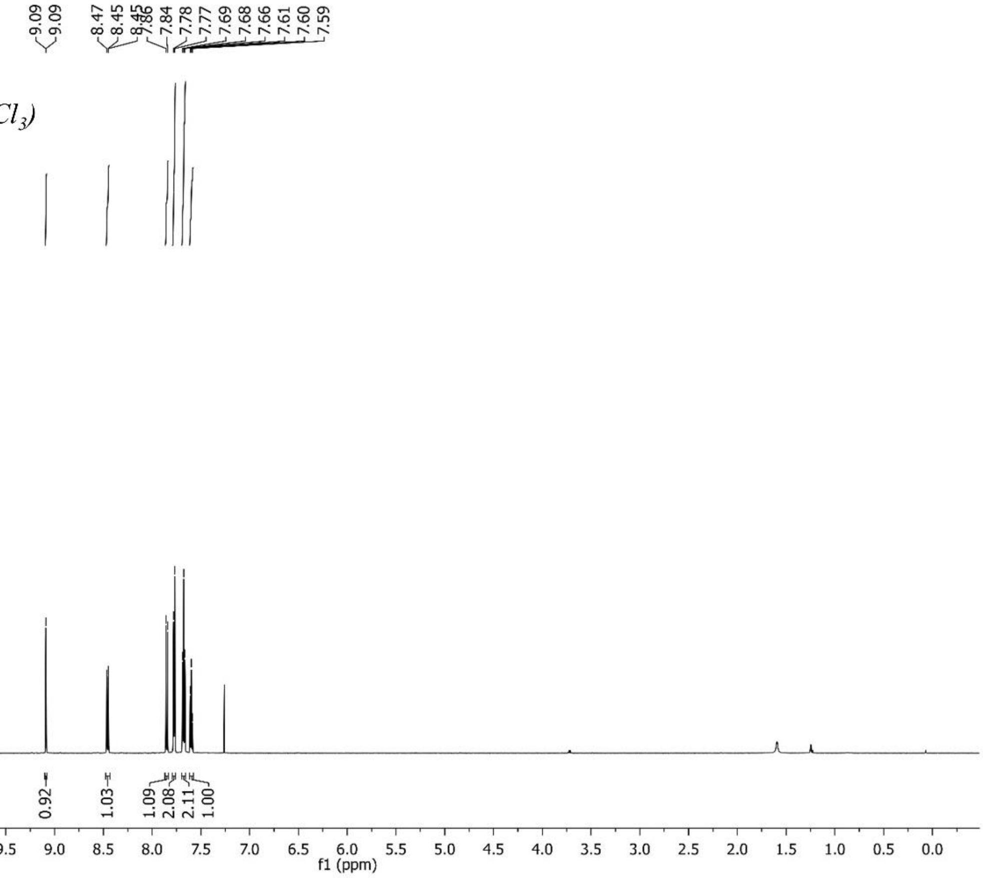

^{13}C NMR Spectrum ($CDCl_3$)

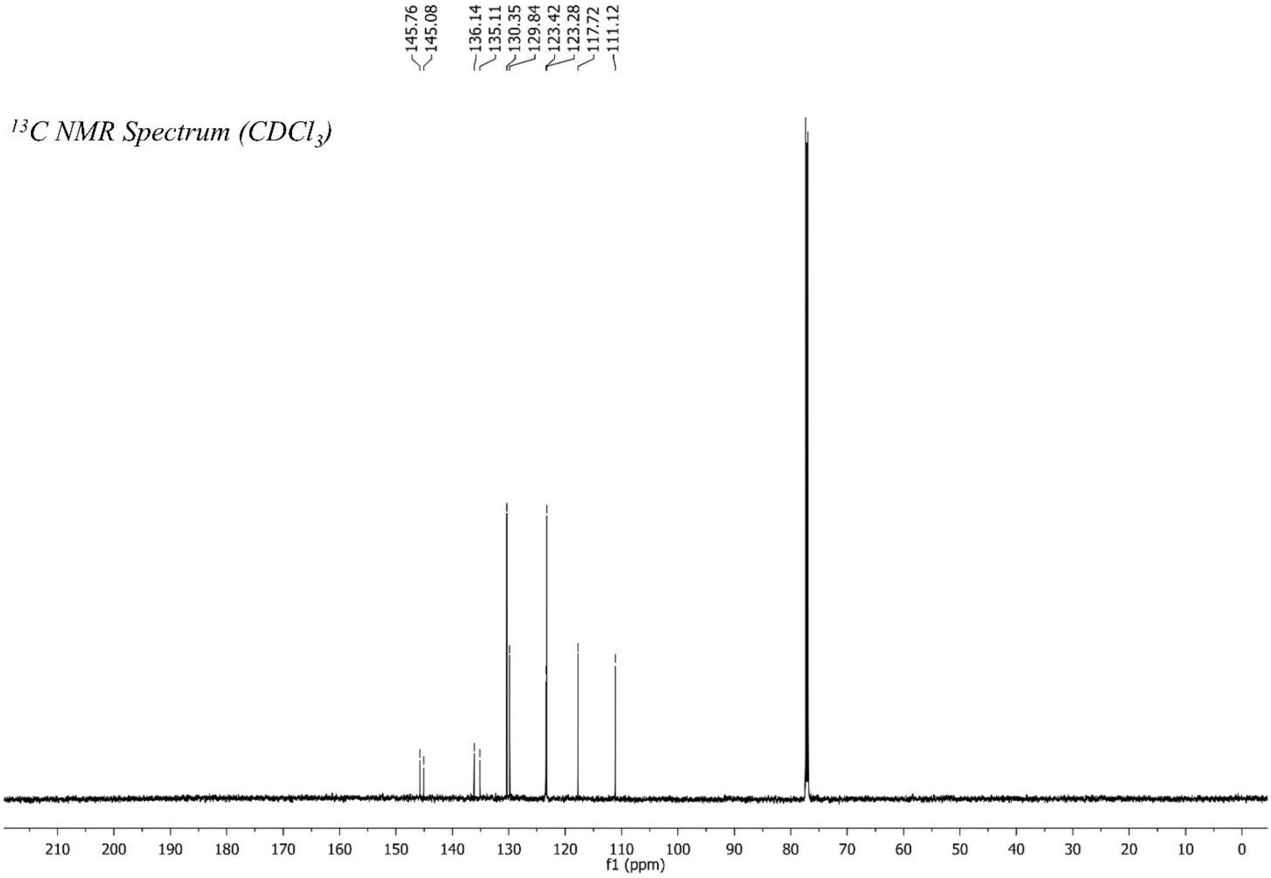

1-Phenyl-1H-benzo[d]imidazol-5-amine (**47**)

^{1}H NMR Spectrum ($CDCl_3$)

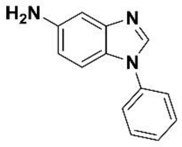

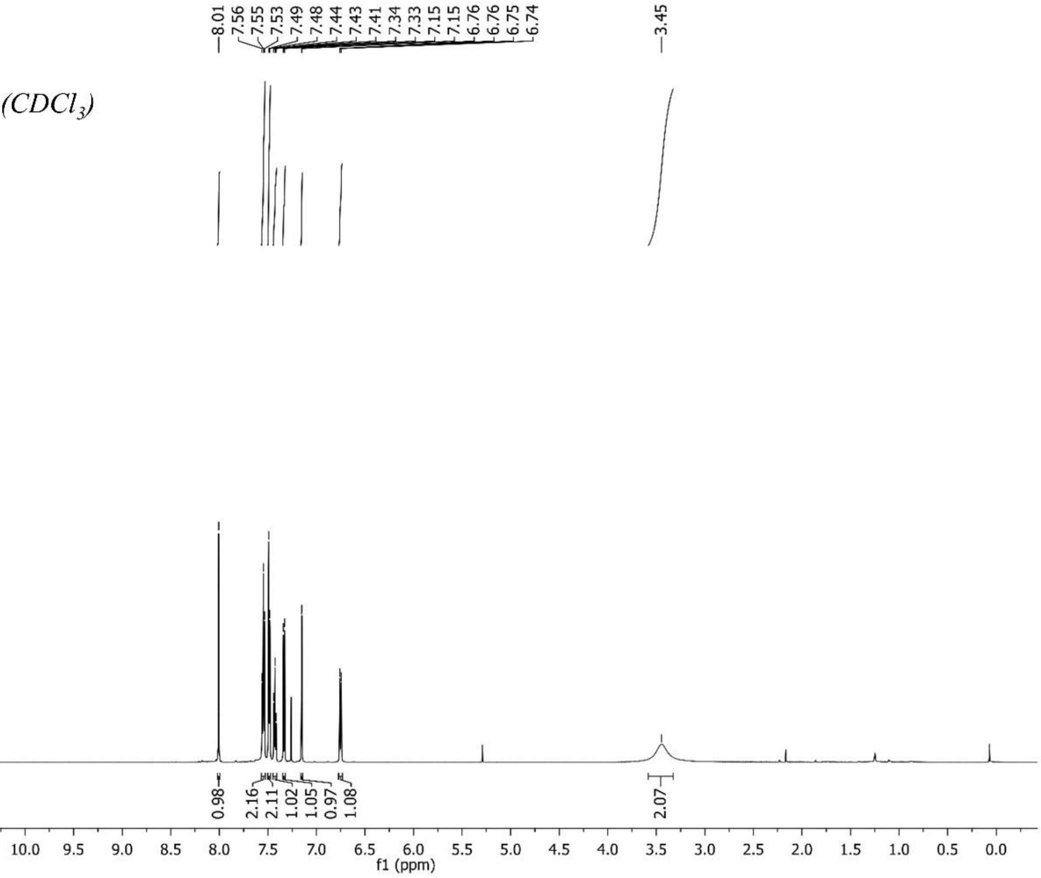

^{13}C NMR Spectrum ($CDCl_3$)

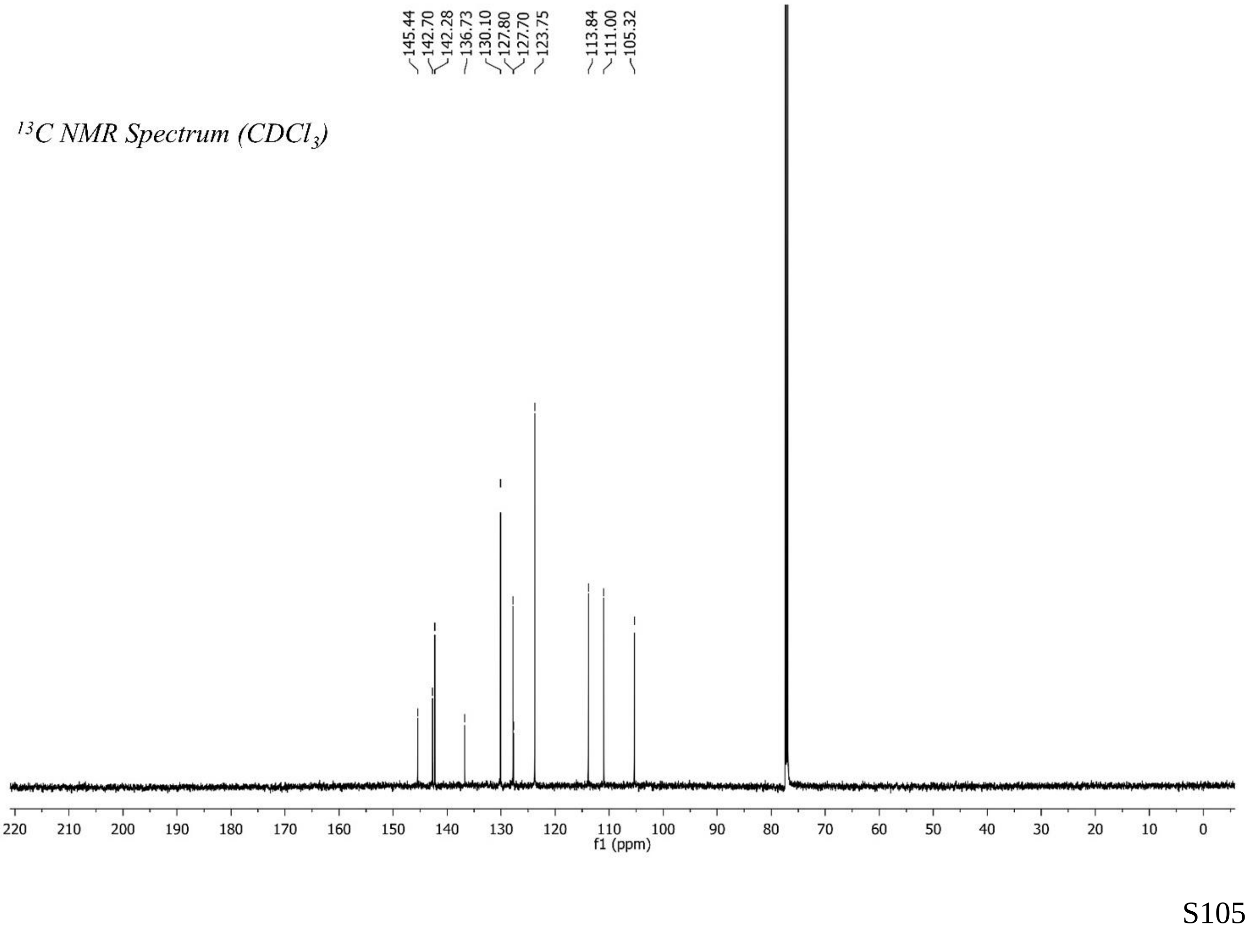

1-Phenyl-1H-benzo[d][1,2,3]triazol-5-amine (**48**)

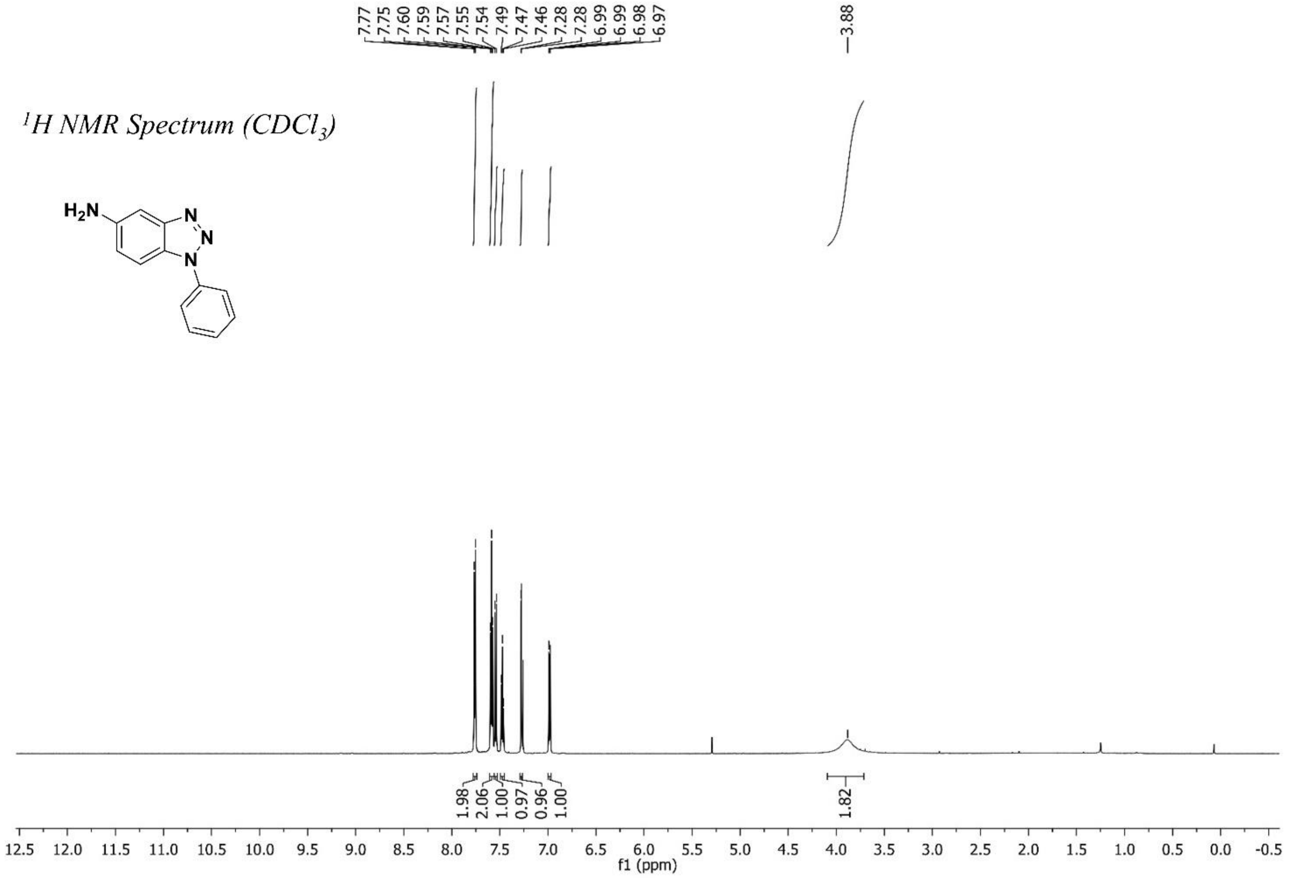

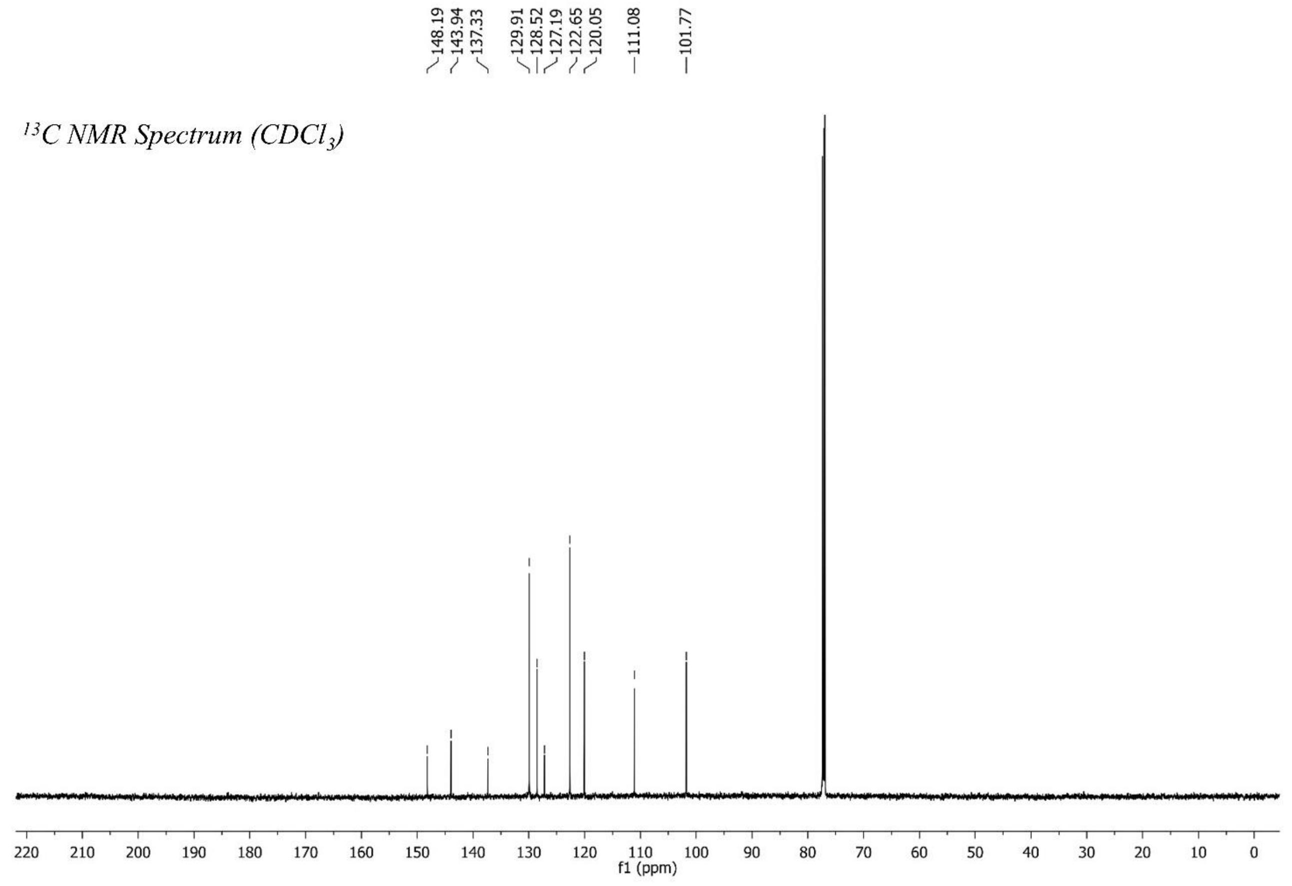

5-Nitro-1-phenyl-1H-indole (**88**)

^{1}H NMR Spectrum ($CDCl_3$)

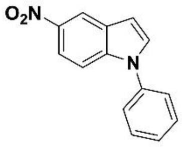

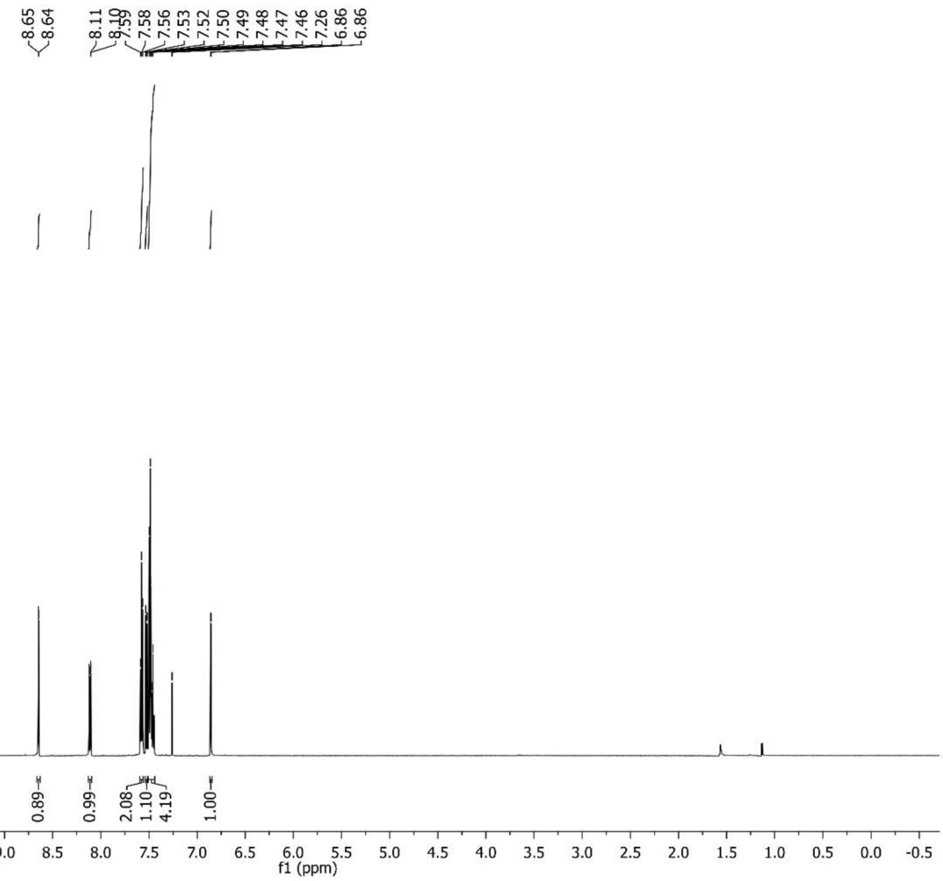

^{13}C NMR Spectrum ($CDCl_3$)

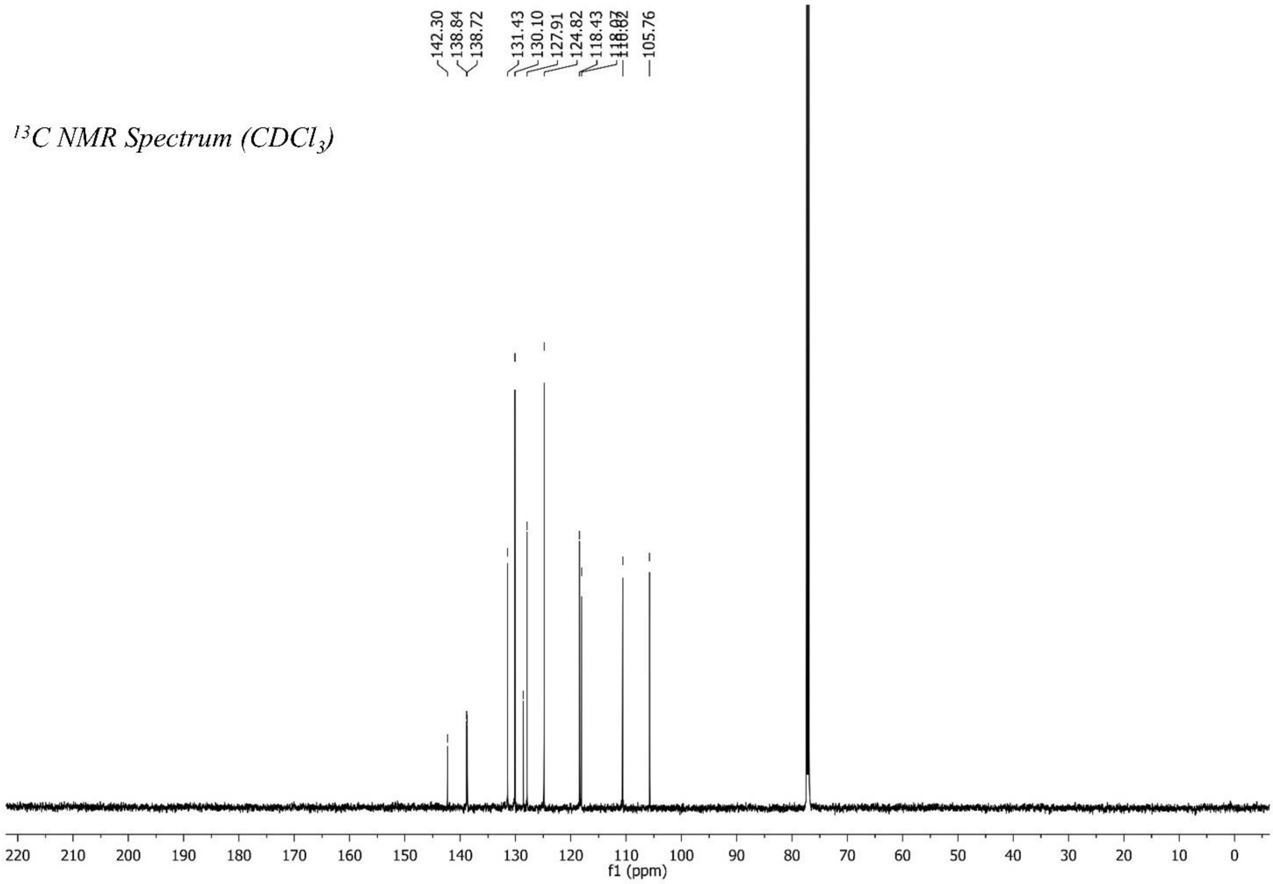

1H-Indol-5-amine (**45**)

^{1}H NMR Spectrum ($CDCl_3$)

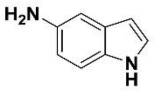

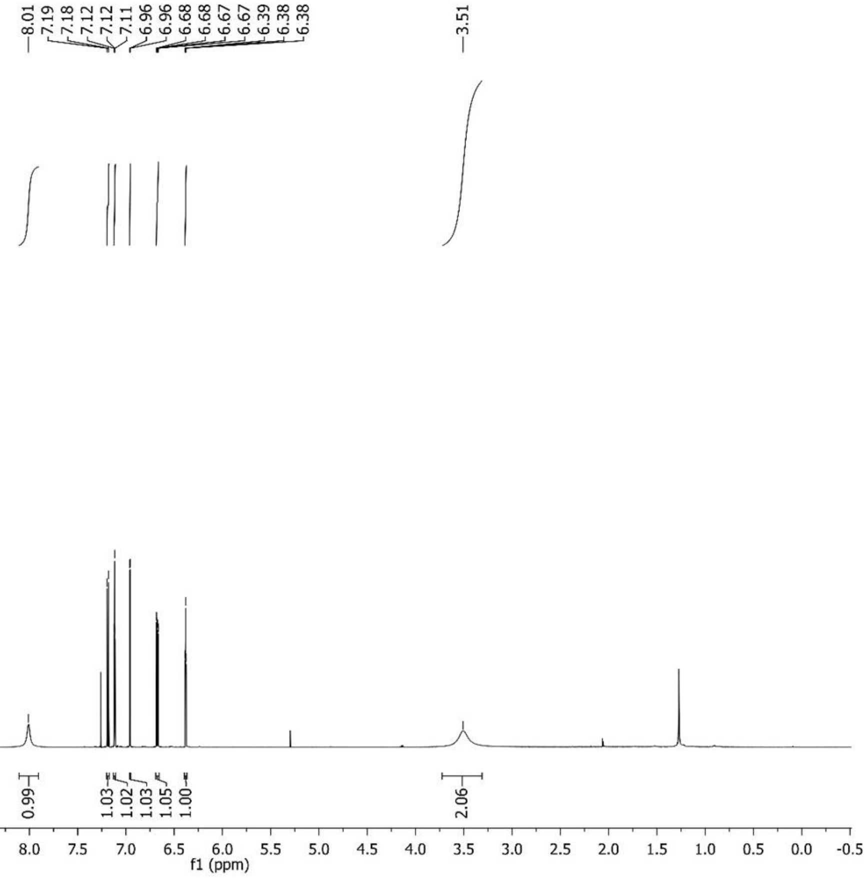

^{13}C NMR Spectrum ($CDCl_3$)

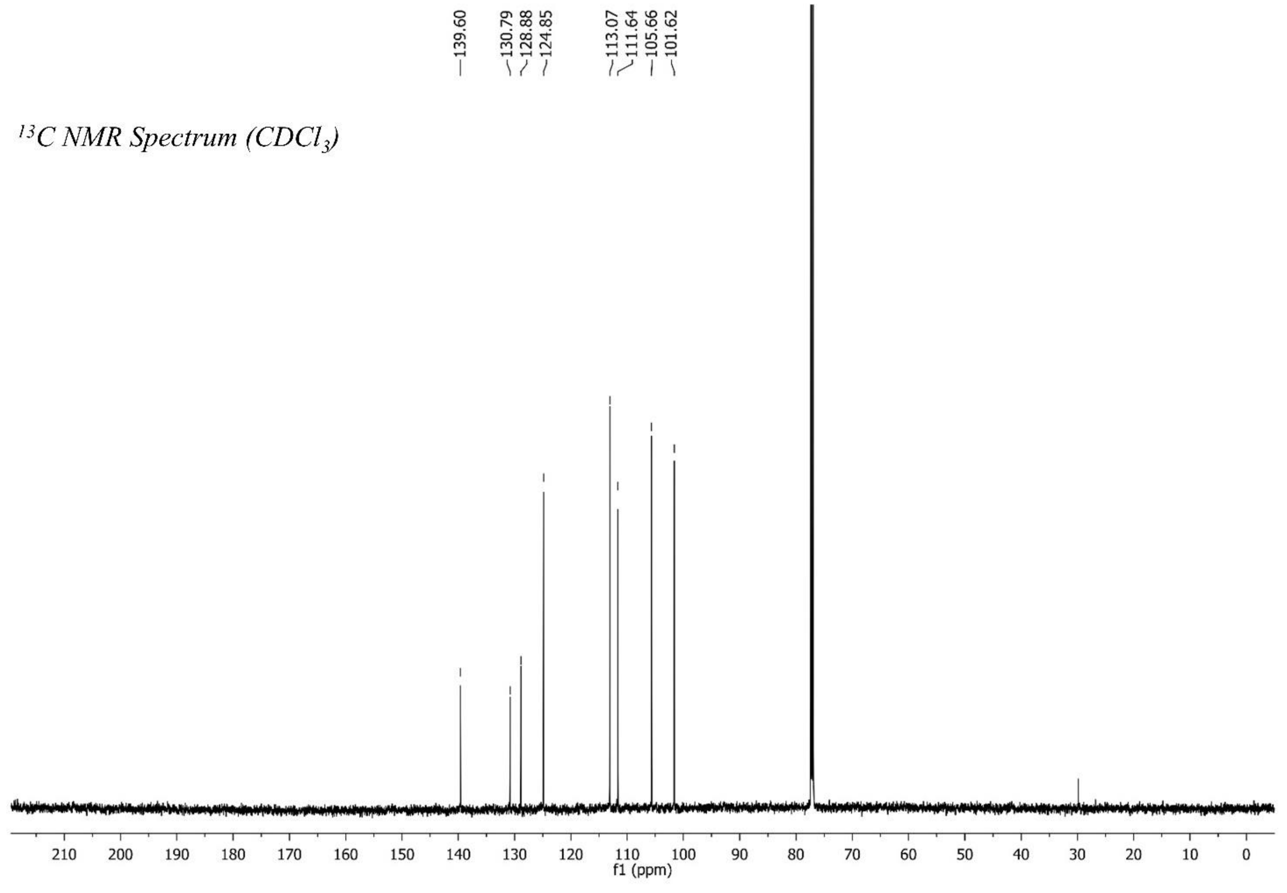

1-Phenyl-1H-indol-5-amine (**46**)

^{1}H NMR Spectrum ($CDCl_3$)

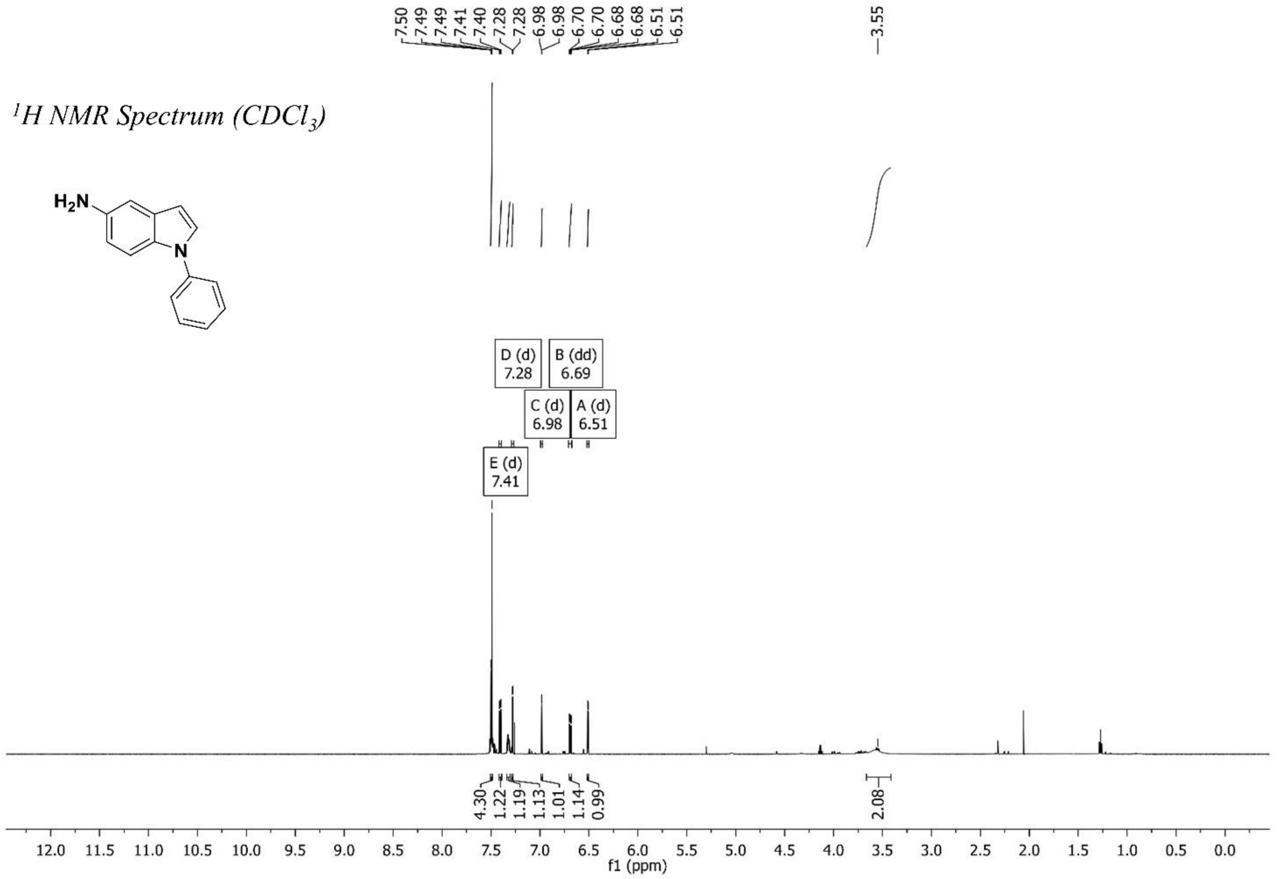

^{13}C NMR Spectrum ($CDCl_3$)

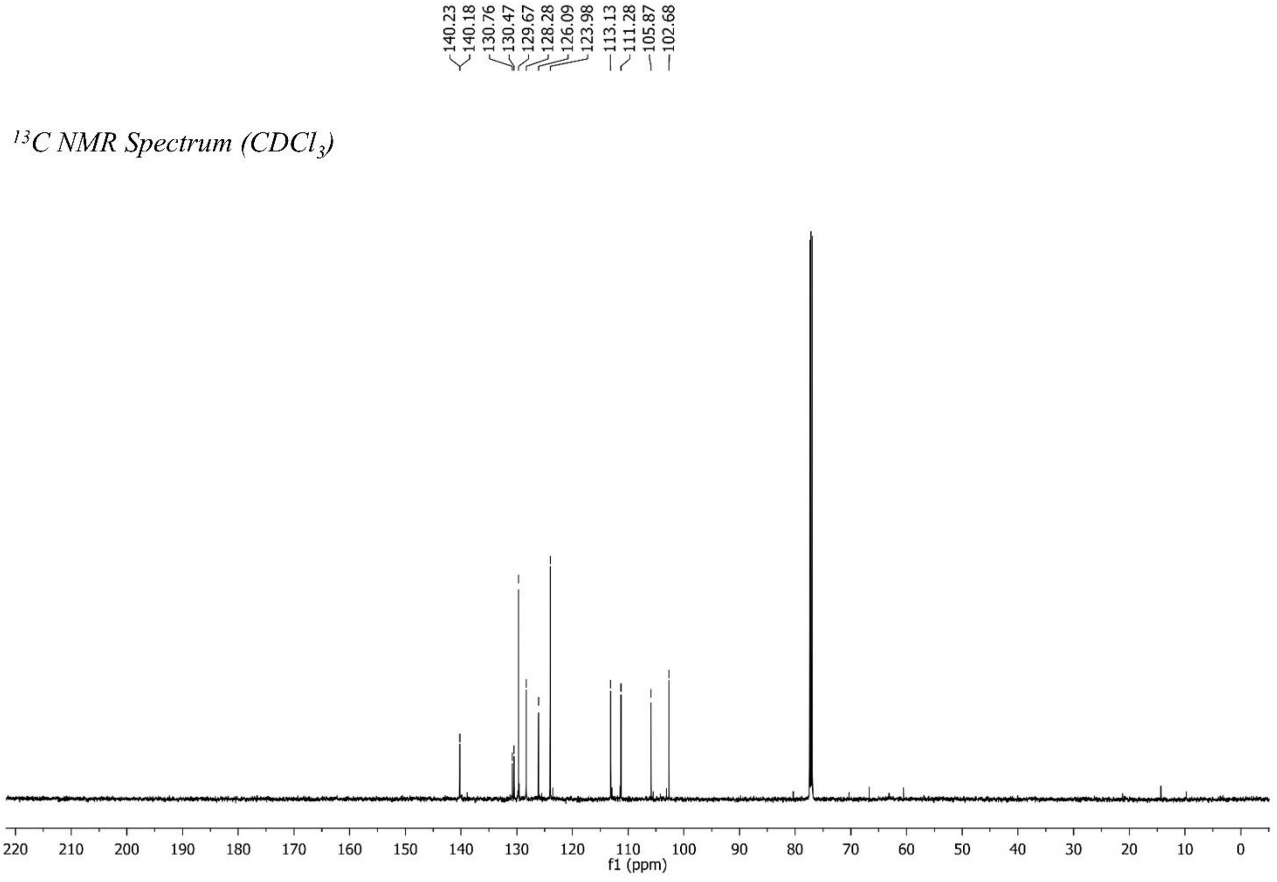

M503_1-ng-ul_inf_MS-neg 06/04/18 11:18:10

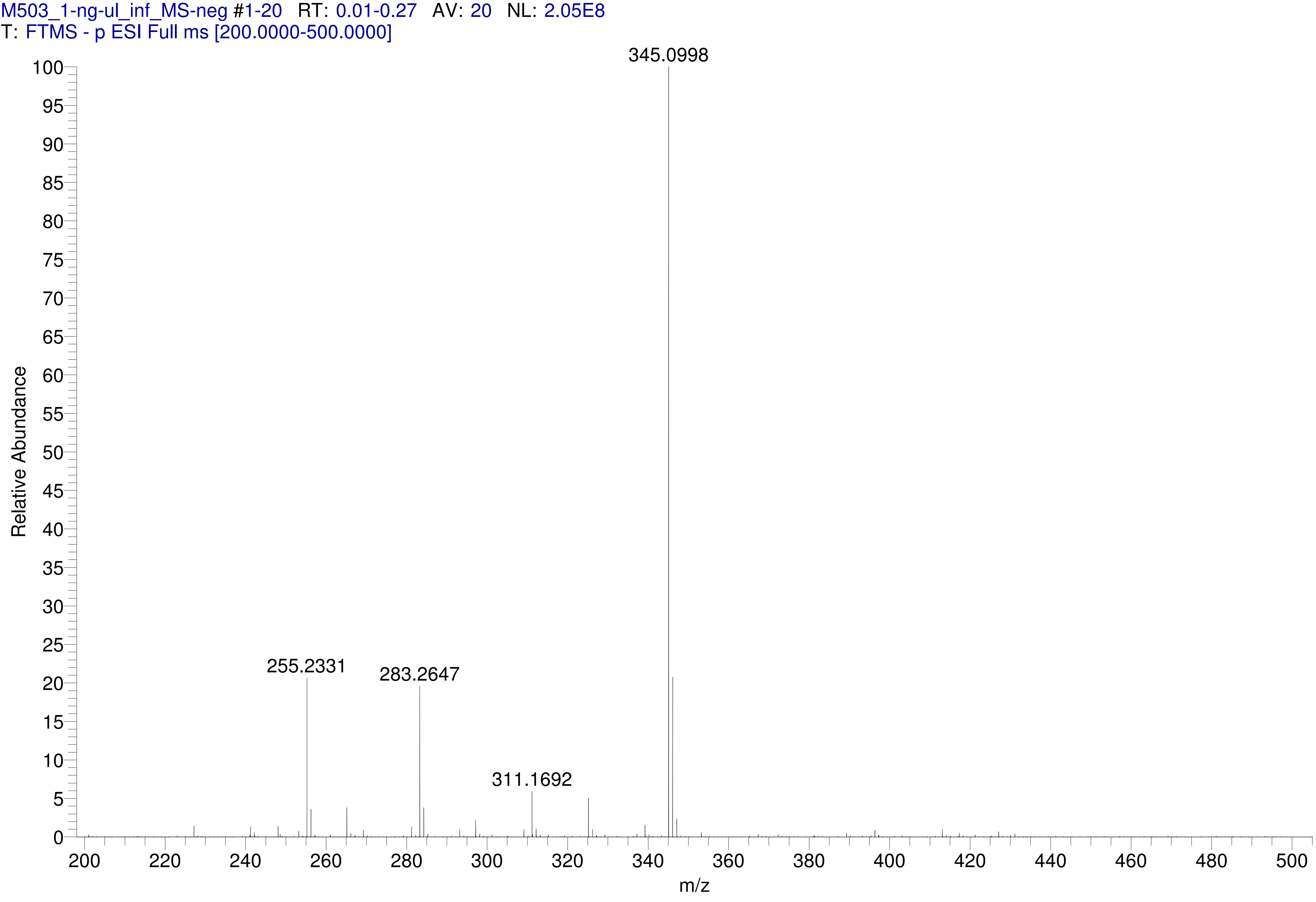

M504_1-ng-ul_inf_MS-pos 06/04/18 10:56:30

M504_1-ng-ul_inf_MS-pos #1-20 RT: 0.01-0.27 AV: 20 NL: 1.80E7
T: FTMS + p ESI Full ms [300.0000-550.0000]

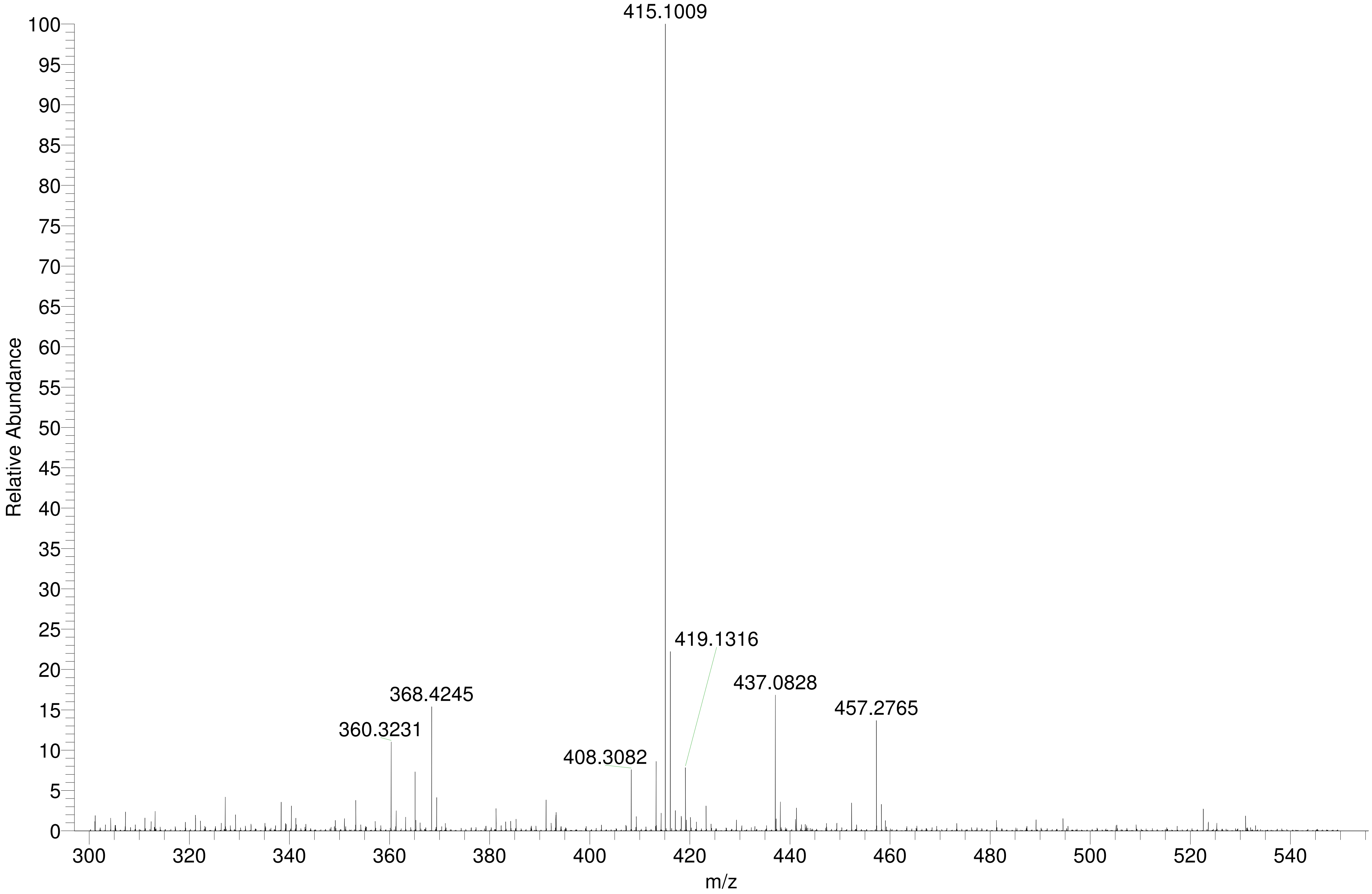

M508_1-ng-ul_inf_MS-neg #1-20 RT: 0.01-0.27 AV: 20 NL: 1.67E8
T: FTMS - p ESI Full ms [300.0000-500.0000]

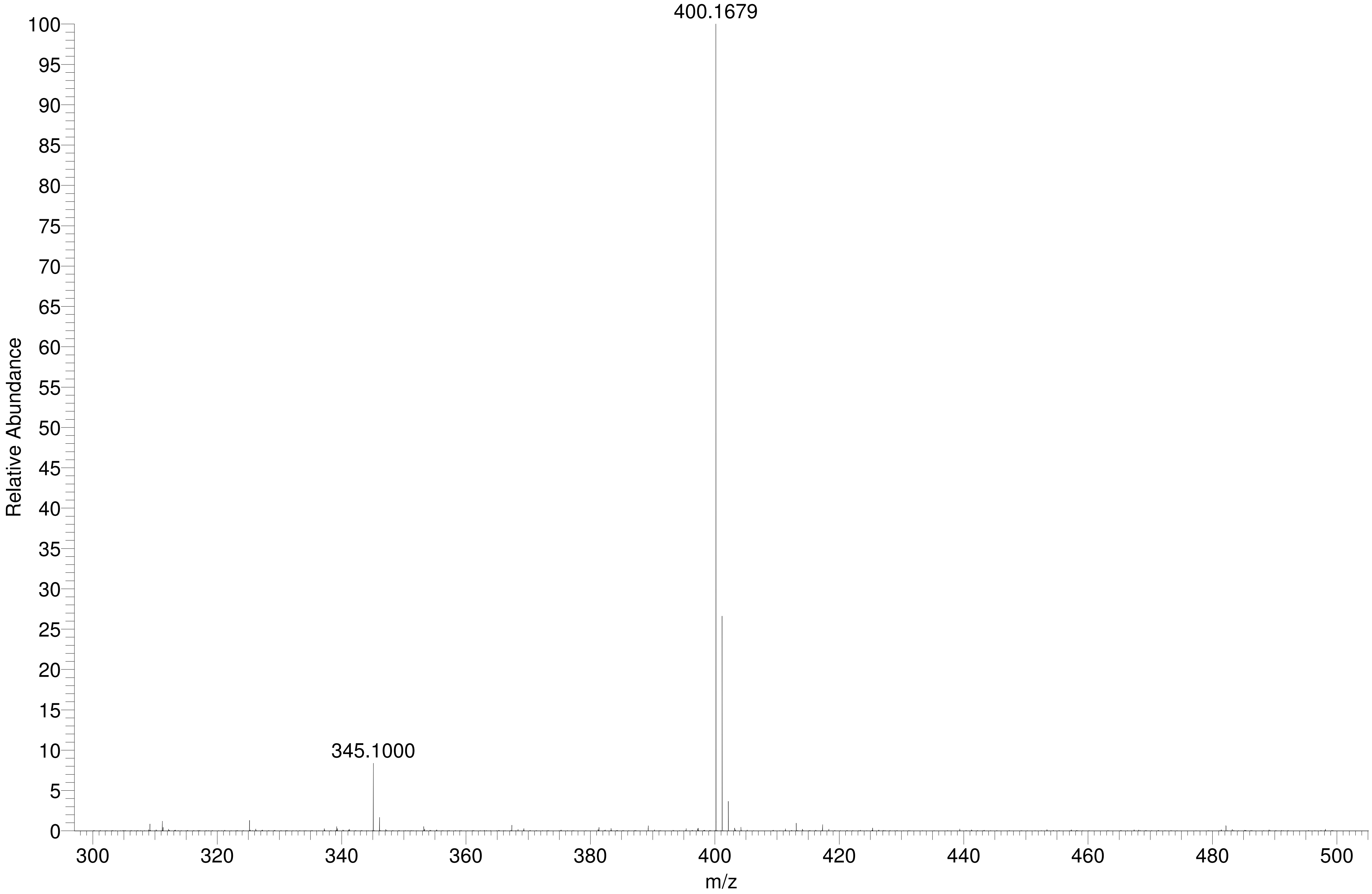

P:\Renzo\...\M548__5ng-ul__inf__MS__pos 07/14/21 10:17:57

M548__5ng-ul__inf__MS__pos #1 RT: 0.01 AV: 1 NL: 1.10E8
T: FTMS + p ESI sid=10.00 Full ms [300.0000-500.0000]

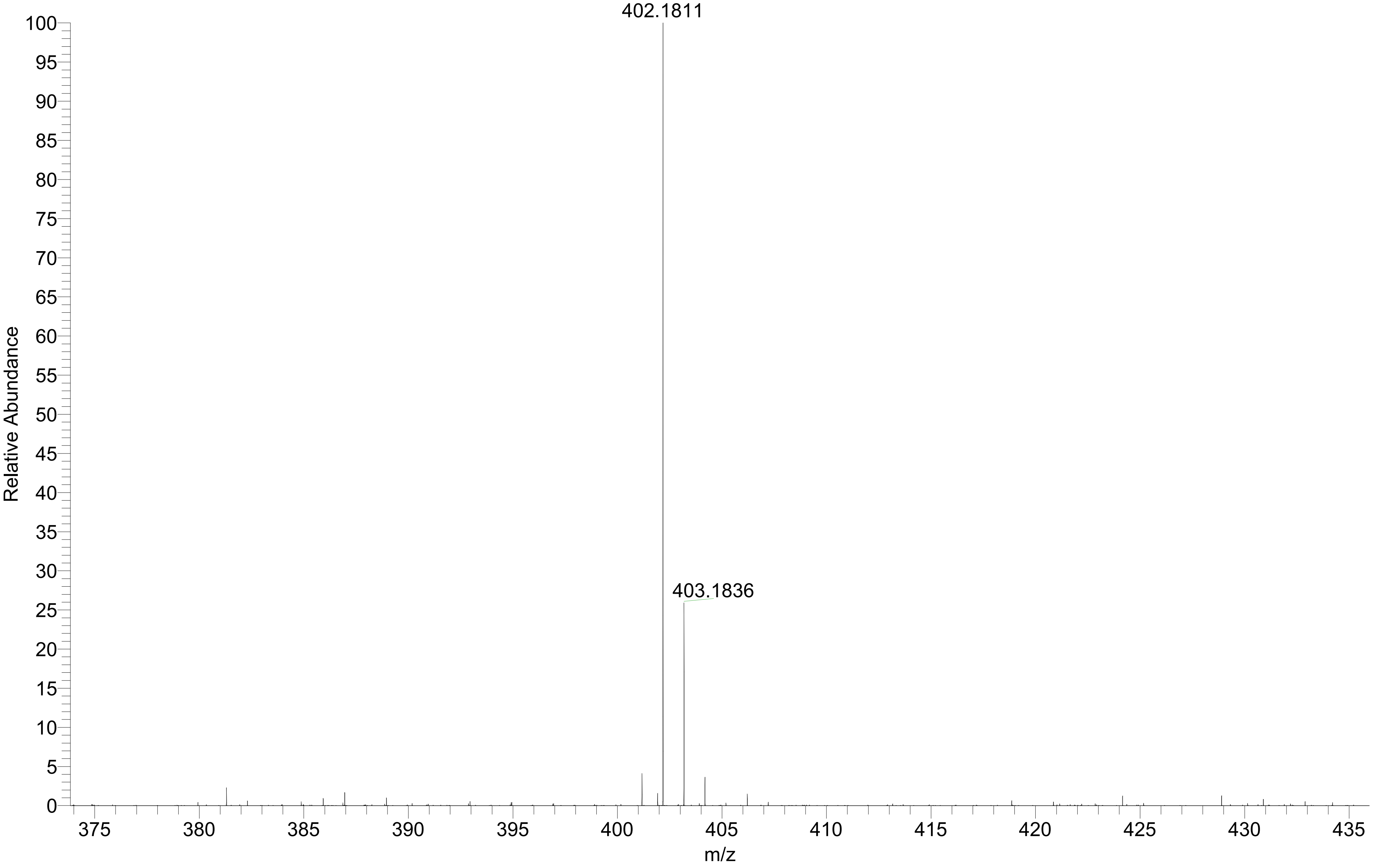

P:\Renzo\...\M549__5ng-ul__inf__MS__pos 07/14/21 10:30:32

M549__5ng-ul__inf__MS__pos #1 RT: 0.01 AV: 1 NL: 3.79E7
T: FTMS + p ESI Full ms [320.0000-420.0000]

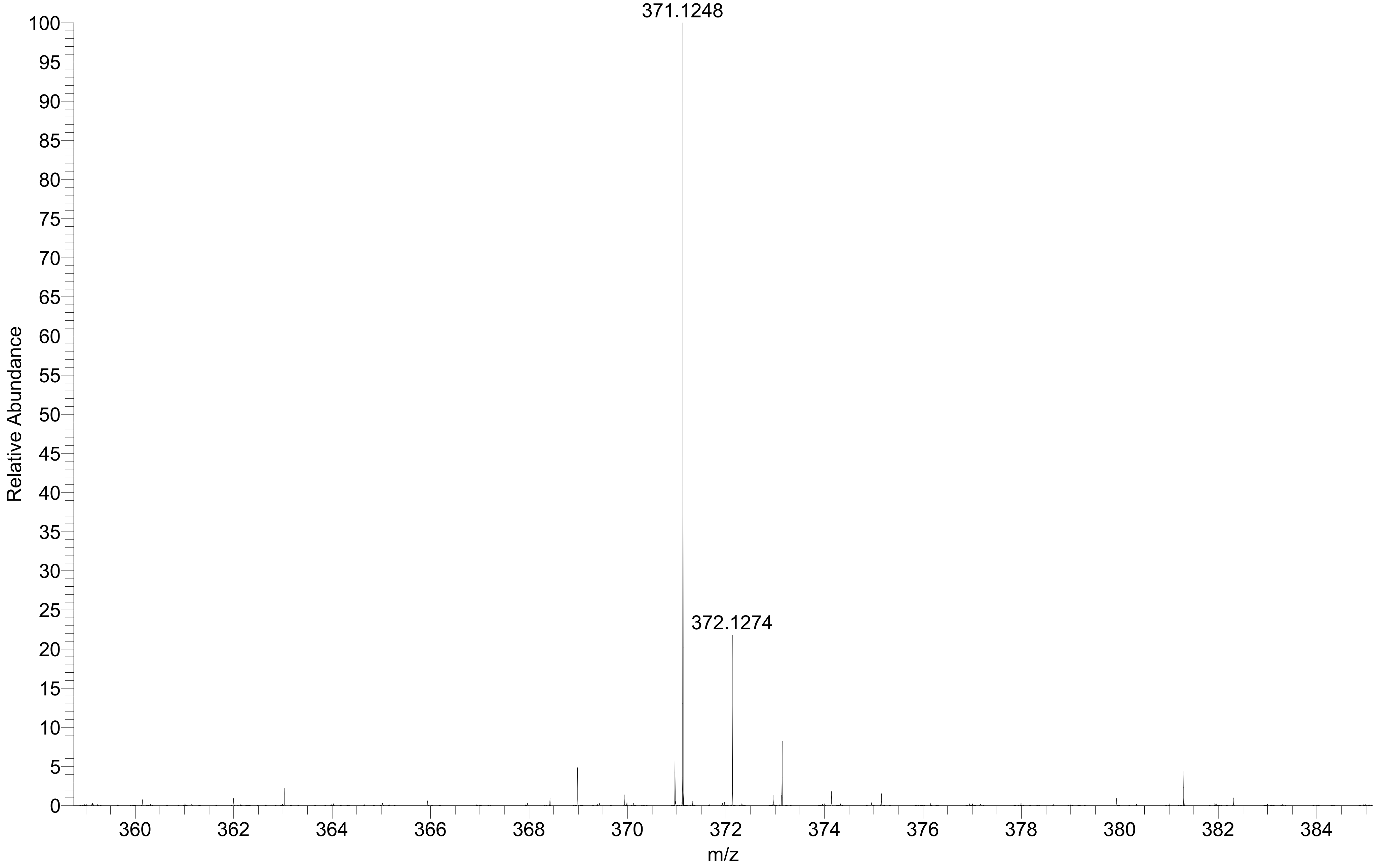

P:\Renzo\...\M550__5ng-ul__inf__MS__pos 07/14/21 10:37:52

M550__5ng-ul__inf__MS__pos #1-40 RT: 0.01-0.36 AV: 40 NL: 6.10E8
T: FTMS + p ESI Full ms [300.0000-450.0000]

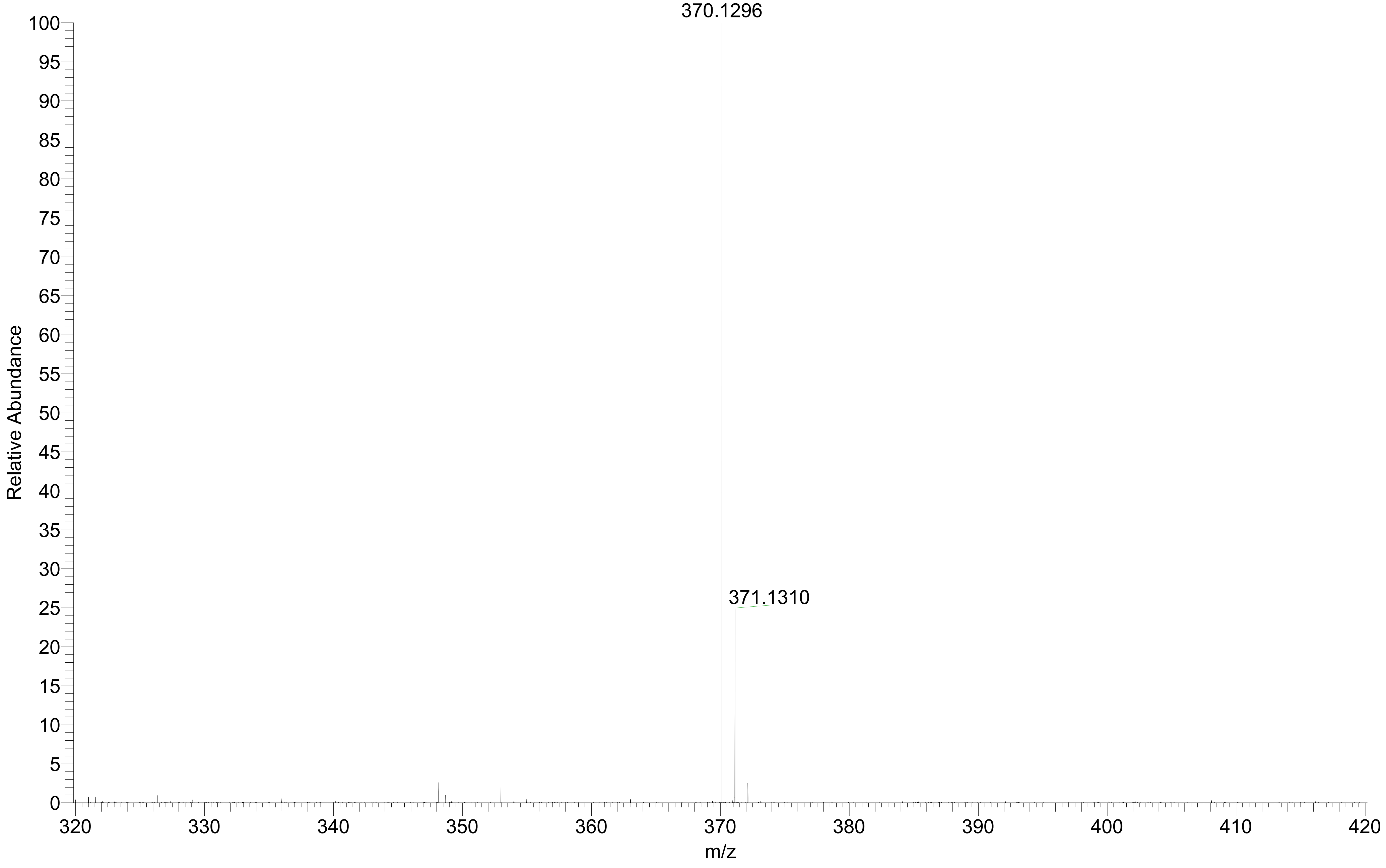

P:\Renzo\...\M551__5ng-ul__inf__MS__pos 07/14/21 13:40:47

M551__5ng-ul__inf__MS__pos #1-40 RT: 0.01-0.36 AV: 40 NL: 5.12E7
T: FTMS + p ESI Full ms [330.0000-400.0000]

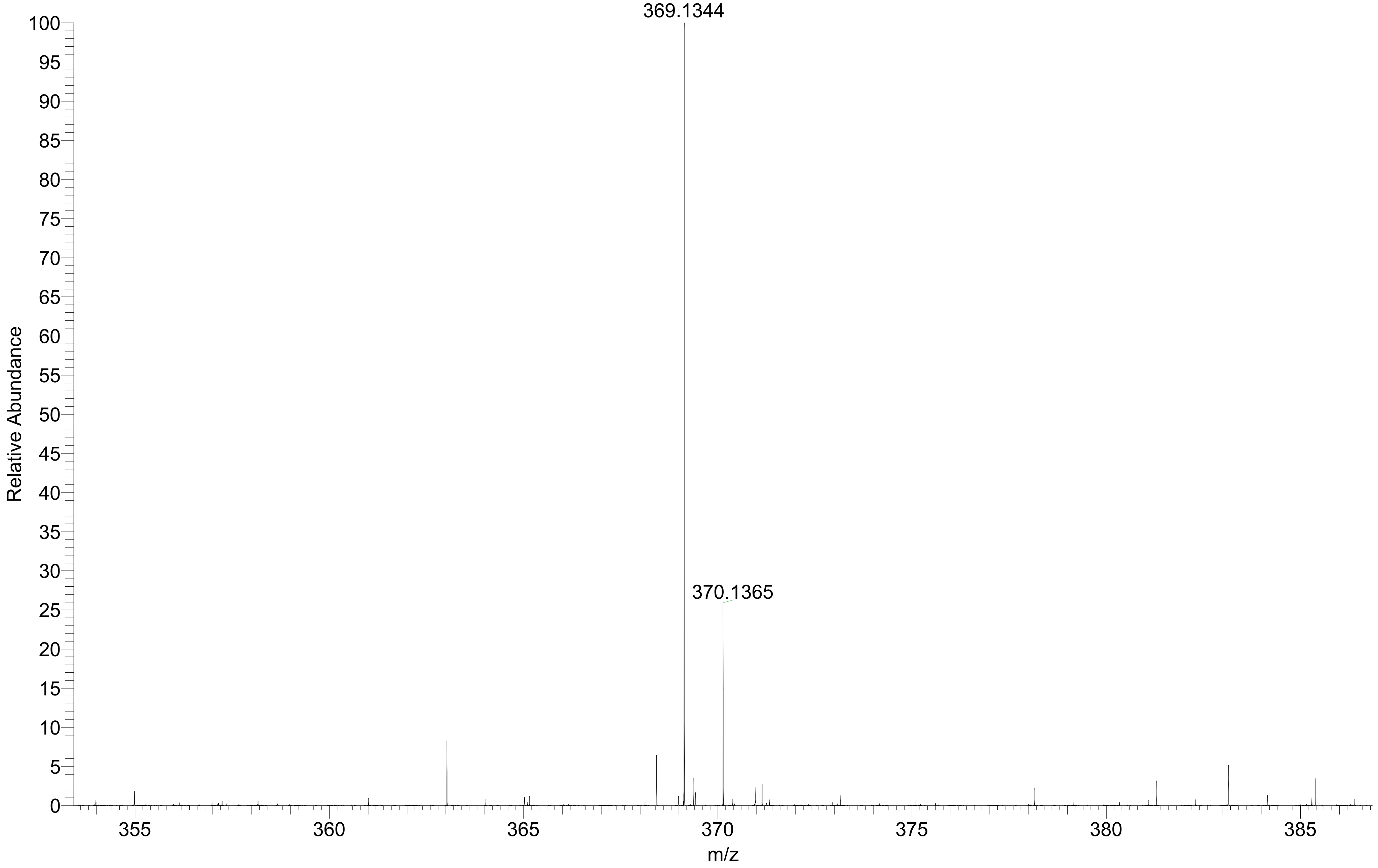

P:\Renzo\...\M552__5ng-ul__inf__MS__pos 07/14/21 11:13:05

M552__5ng-ul__inf__MS__pos #1-40 RT: 0.01-0.36 AV: 40 NL: 5.45E7
T: FTMS + p ESI SIM ms [270.0000-300.0000]

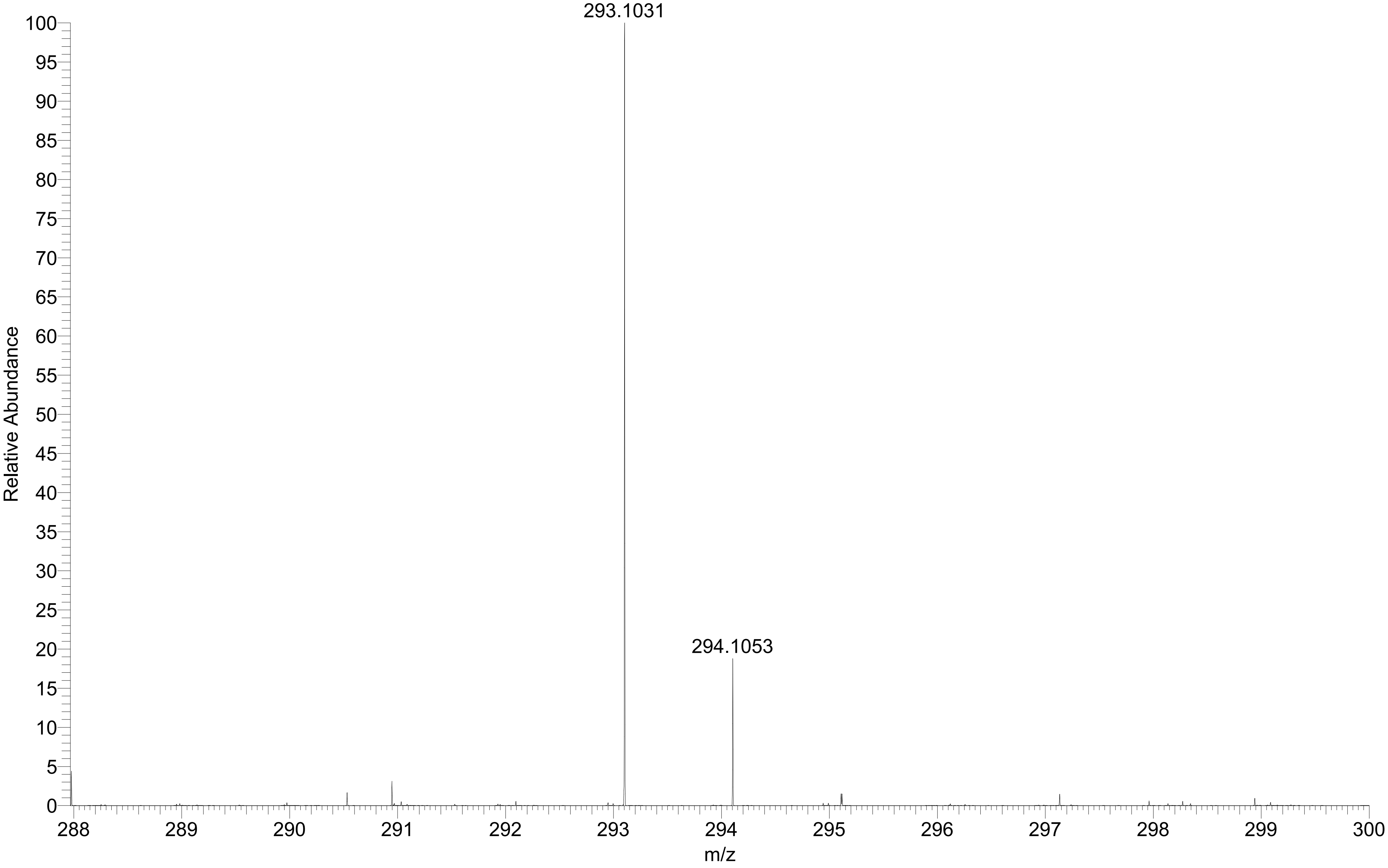

P:\Renzo\...\M553__5ng-ul__inf__MS__pos 07/14/21 11:30:05

M553__5ng-ul__inf__MS__pos #1-40 RT: 0.01-0.36 AV: 40 NL: 1.16E8
T: FTMS + p ESI SIM ms [330.0000-380.0000]

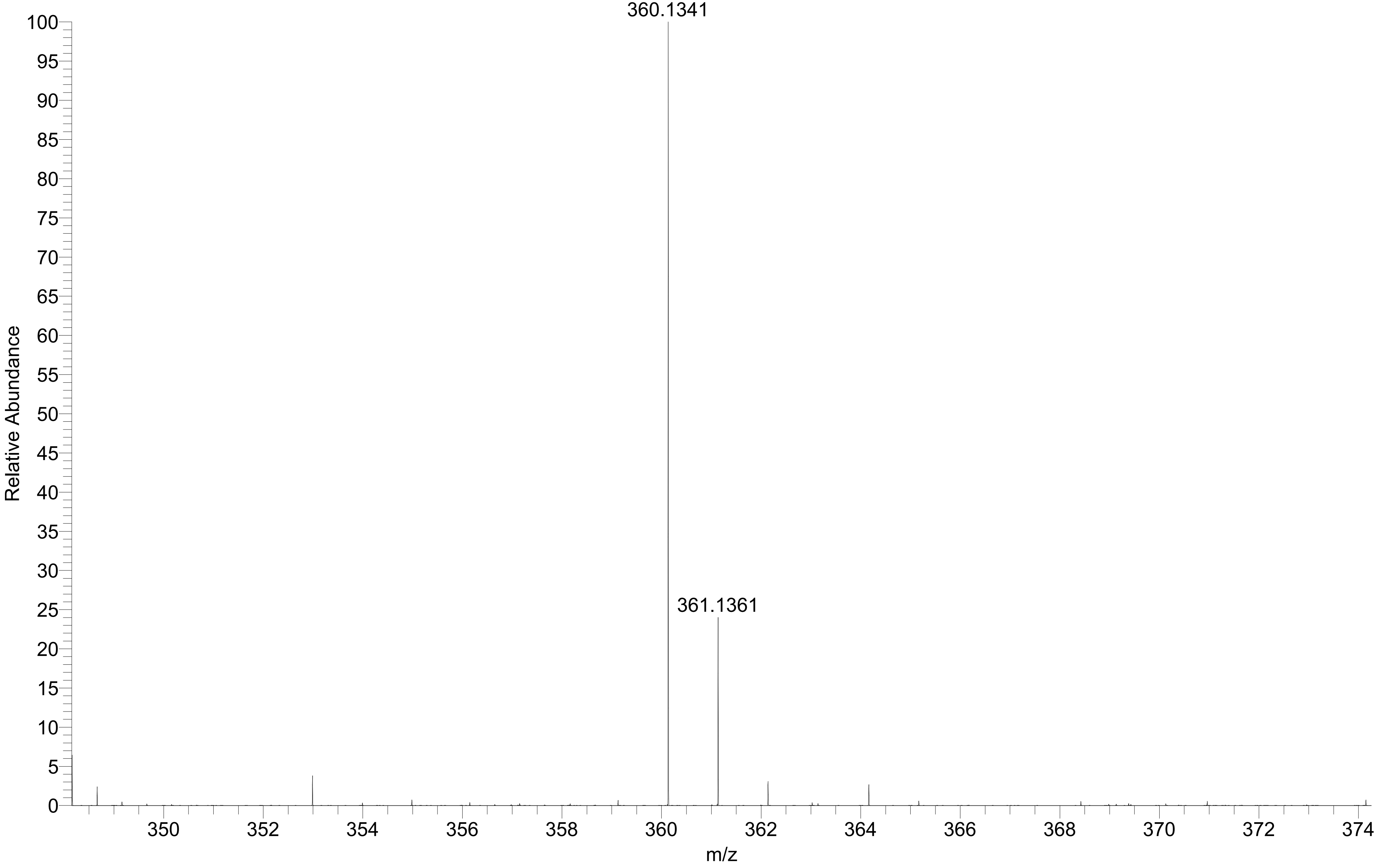

P:\Renzo\...\M554__5ng-ul__inf__MS__pos 07/14/21 11:38:58

M554__5ng-ul__inf__MS__pos #1-40 RT: 0.01-0.36 AV: 40 NL: 1.13E8
T: FTMS + p ESI SIM ms [450.0000-490.0000]

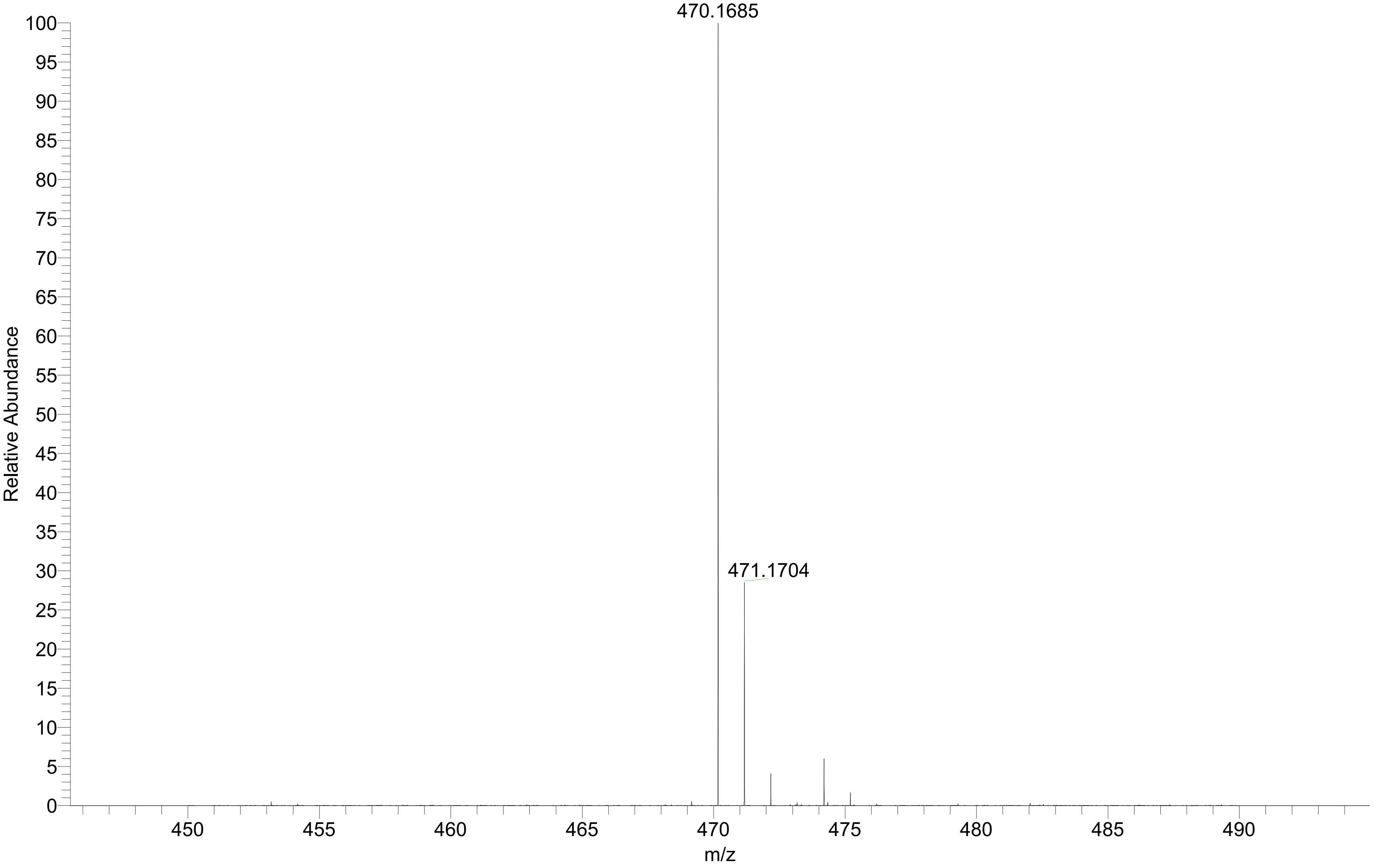

P:\Renzo\...\M605__5ng-ul__inf__MS__pos 07/14/21 11:52:43

M605__5ng-ul__inf__MS__pos #1-40 RT: 0.01-0.36 AV: 40 NL: 6.87E8
T: FTMS + p ESI Full ms [350.0000-450.0000]

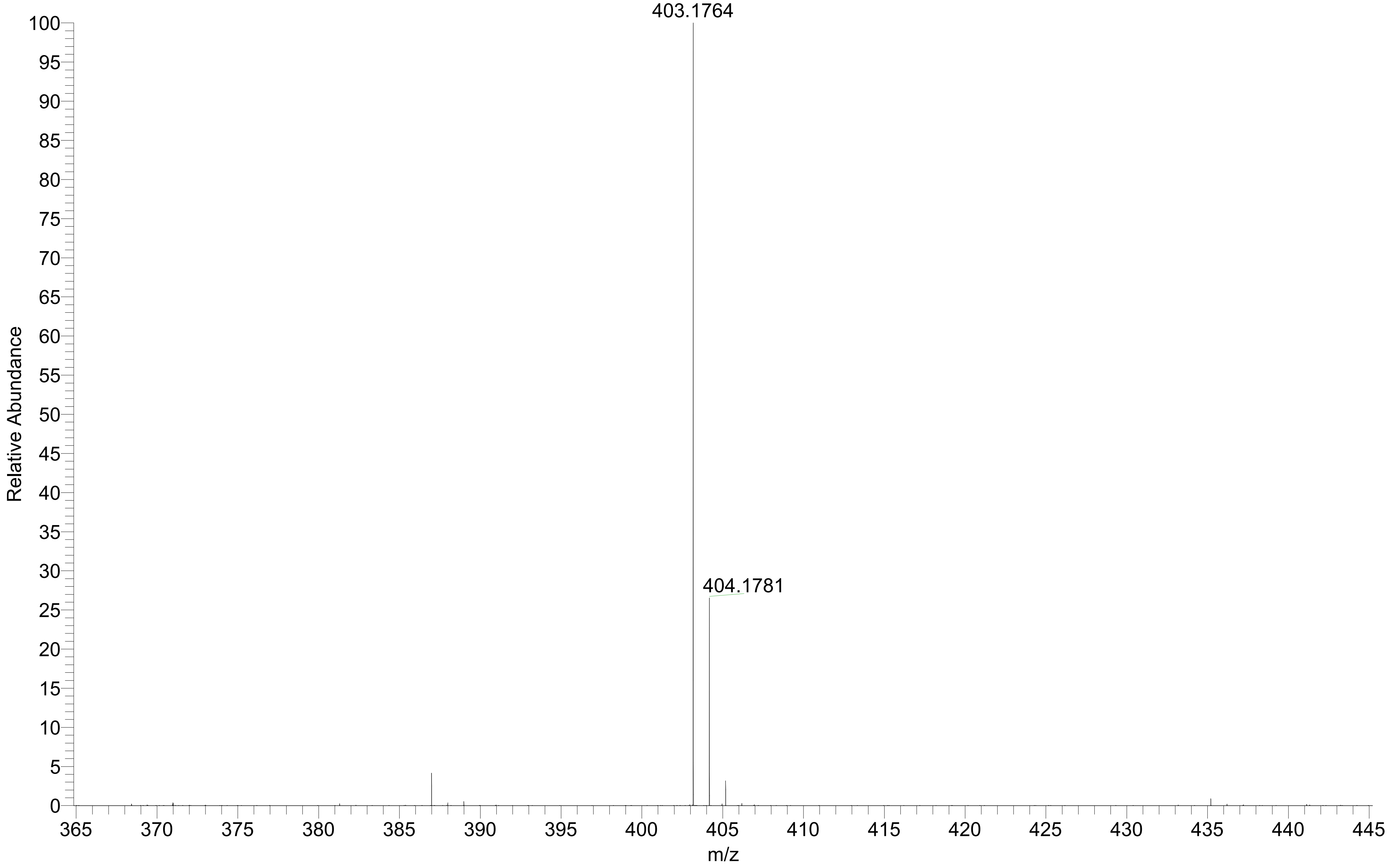

P:\Renzo\...\M612__5ng-ul__inf__MS__pos 07/14/21 13:17:51

M612__5ng-ul__inf__MS__pos #1-40 RT: 0.01-0.36 AV: 40 NL: 7.30E8
T: FTMS + p ESI SIM ms [370.0000-400.0000]

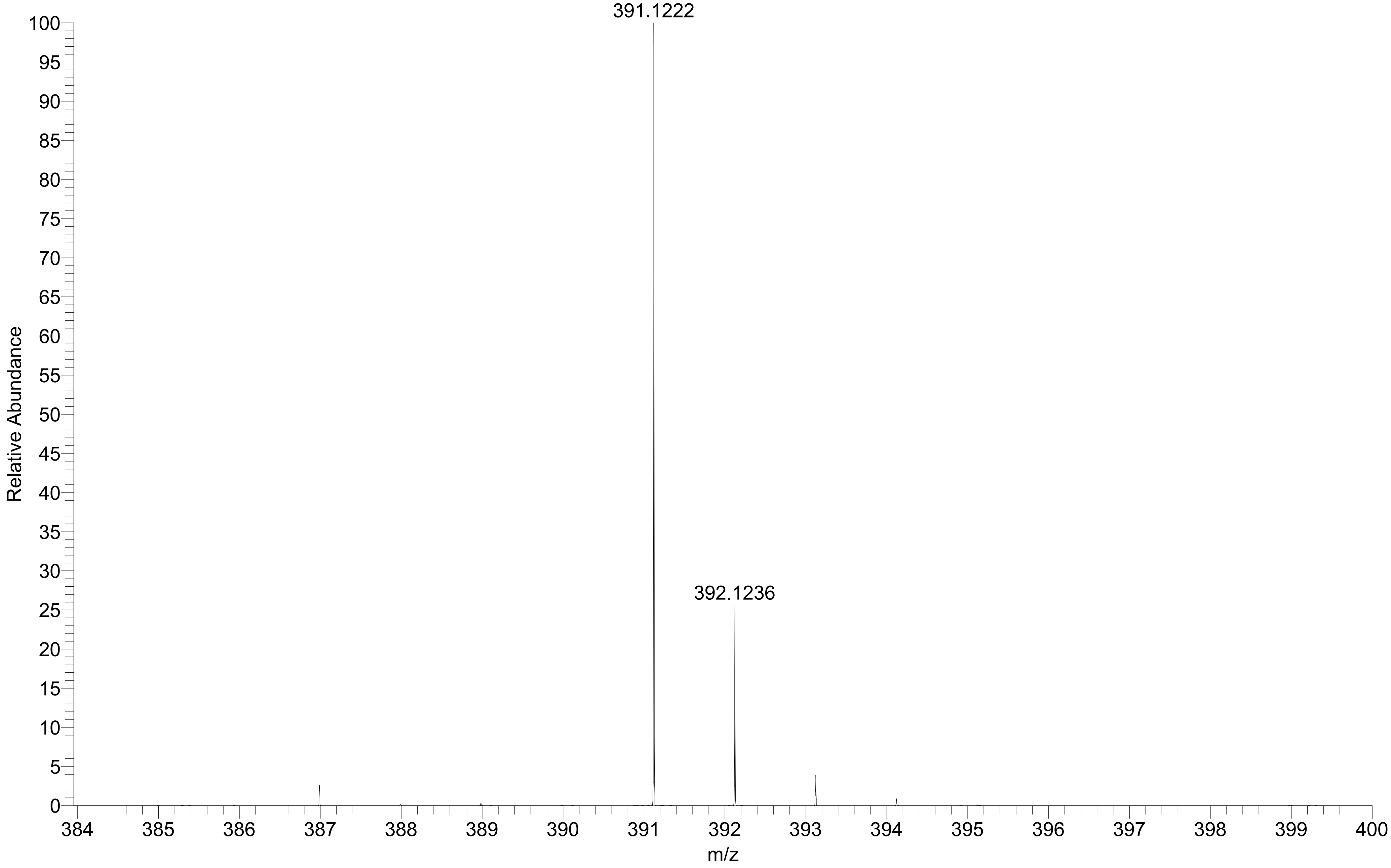

P:\Renzo\...\M623__5ng-ul__inf__MS__pos 07/14/21 13:32:43

M623__5ng-ul__inf__MS__pos #1-40 RT: 0.01-0.36 AV: 40 NL: 1.10E8
T: FTMS + p ESI Full ms [380.0000-450.0000]

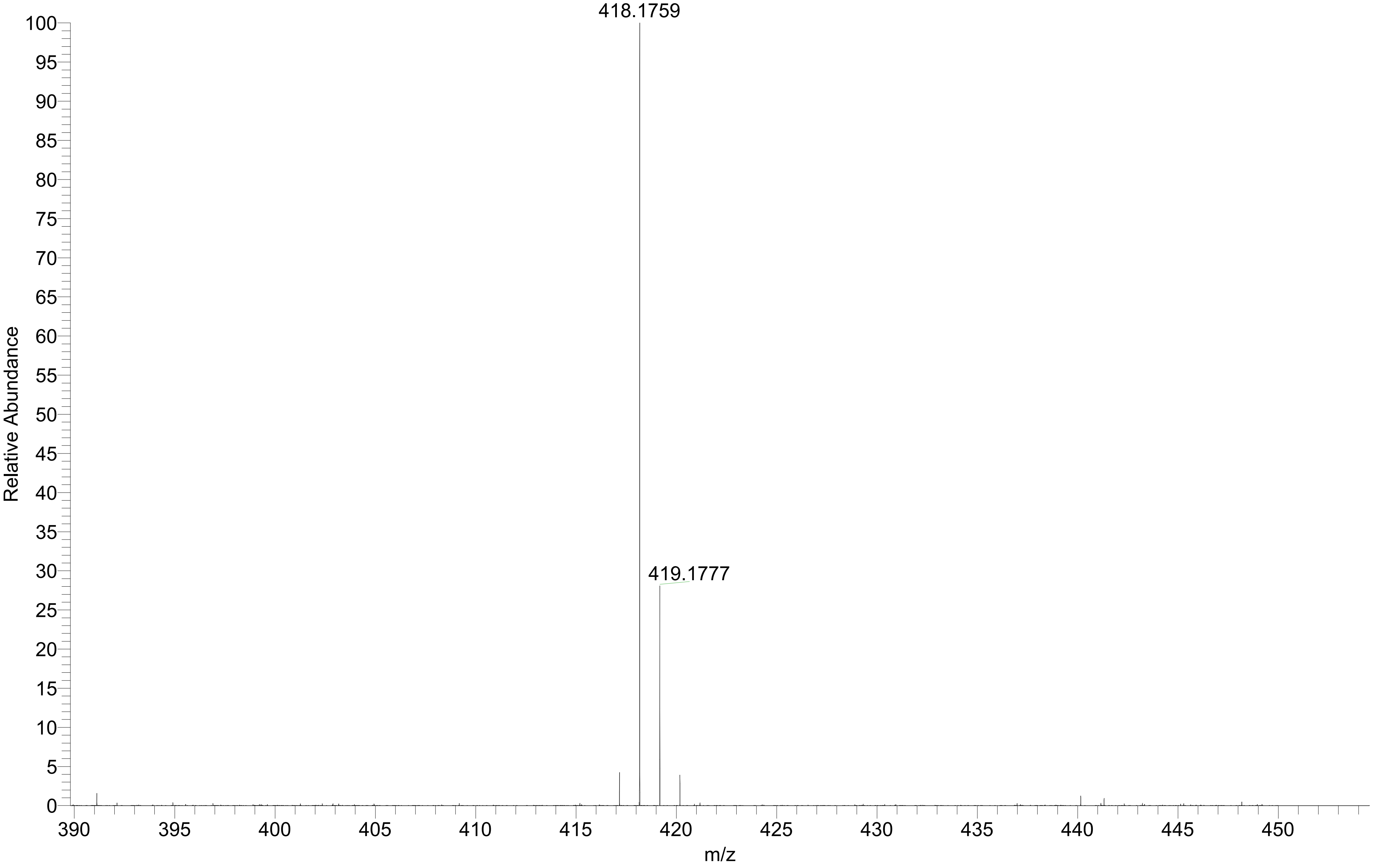

P:\Renzo\...\M626__2ng-ul__inf__MS__pos 07/20/21 10:36:57

M626__2ng-ul__inf__MS__pos #1-40 RT: 0.01-0.53 AV: 40 NL: 3.56E8
T: FTMS + p ESI sid=20.00 Full ms [350.0000-550.0000]

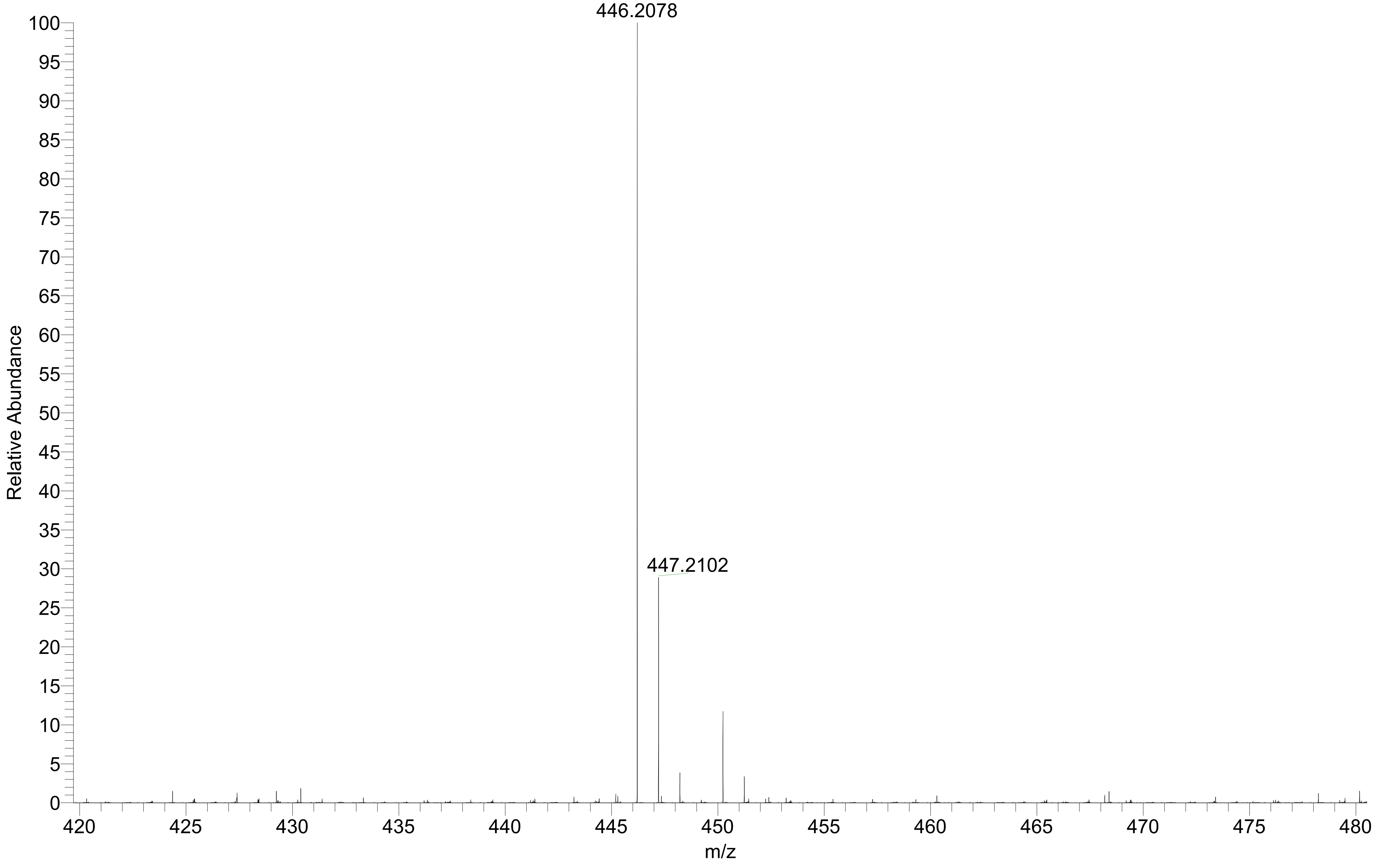

P:\Renzo\...\M627__2ng-ul__inf__MS__pos 07/20/21 10:51:42

M627__2ng-ul__inf__MS__pos #1-40 RT: 0.01-0.53 AV: 40 NL: 6.73E8
T: FTMS + p ESI sid=20.00 Full ms [350.0000-550.0000]

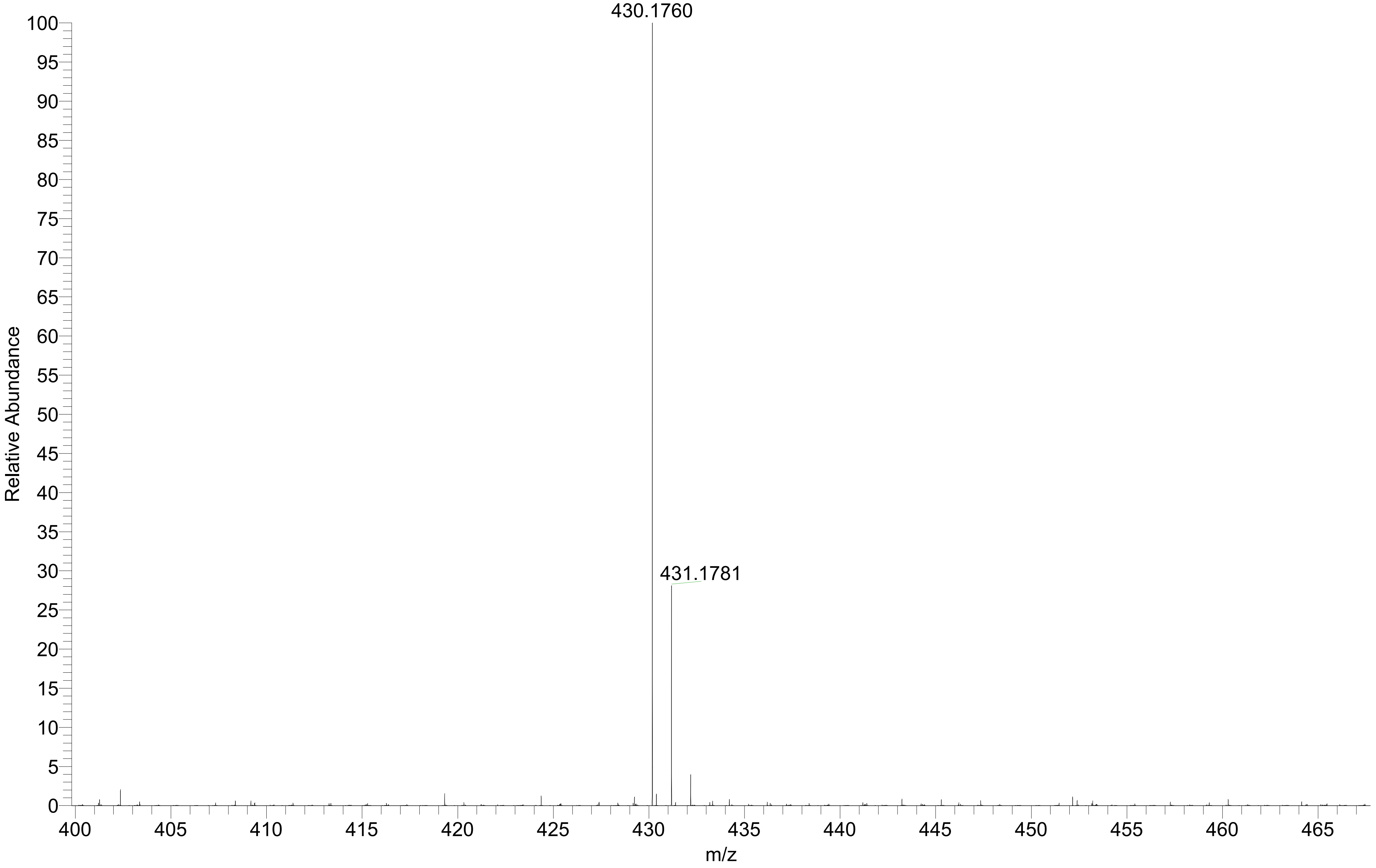

P:\Renzo\...\M628__2ng-ul__inf__MS__pos 07/20/21 10:58:28

M628__2ng-ul__inf__MS__pos #1-40 RT: 0.01-0.53 AV: 40 NL: 8.93E8
T: FTMS + p ESI sid=20.00 Full ms [350.0000-550.0000]

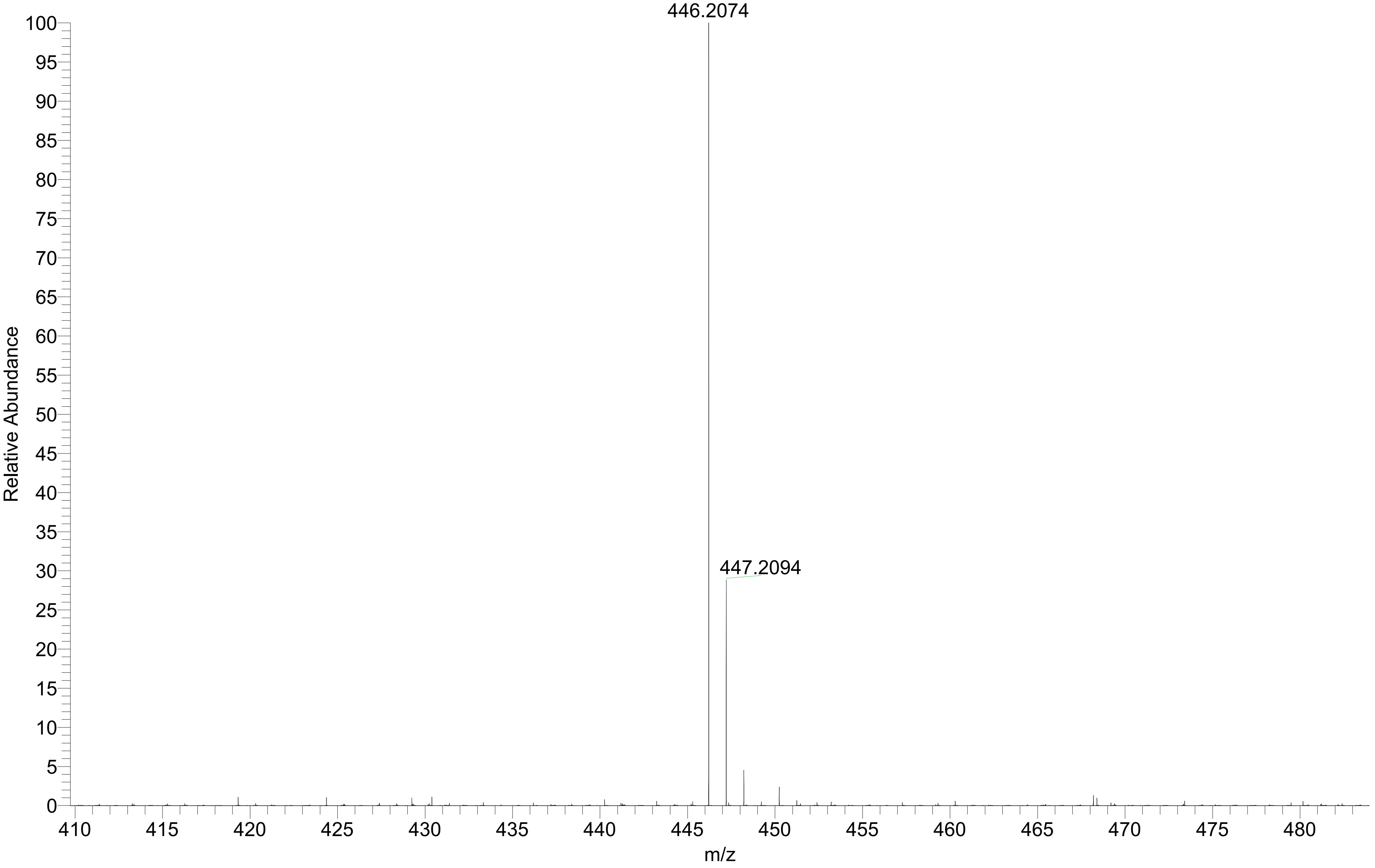

P:\Renzo\...\M629__2ng-ul__inf__MS__pos 07/20/21 11:11:39

M629__2ng-ul__inf__MS__pos #1-40 RT: 0.01-0.53 AV: 40 NL: 7.08E8
T: FTMS + p ESI sid=20.00 Full ms [350.0000-550.0000]

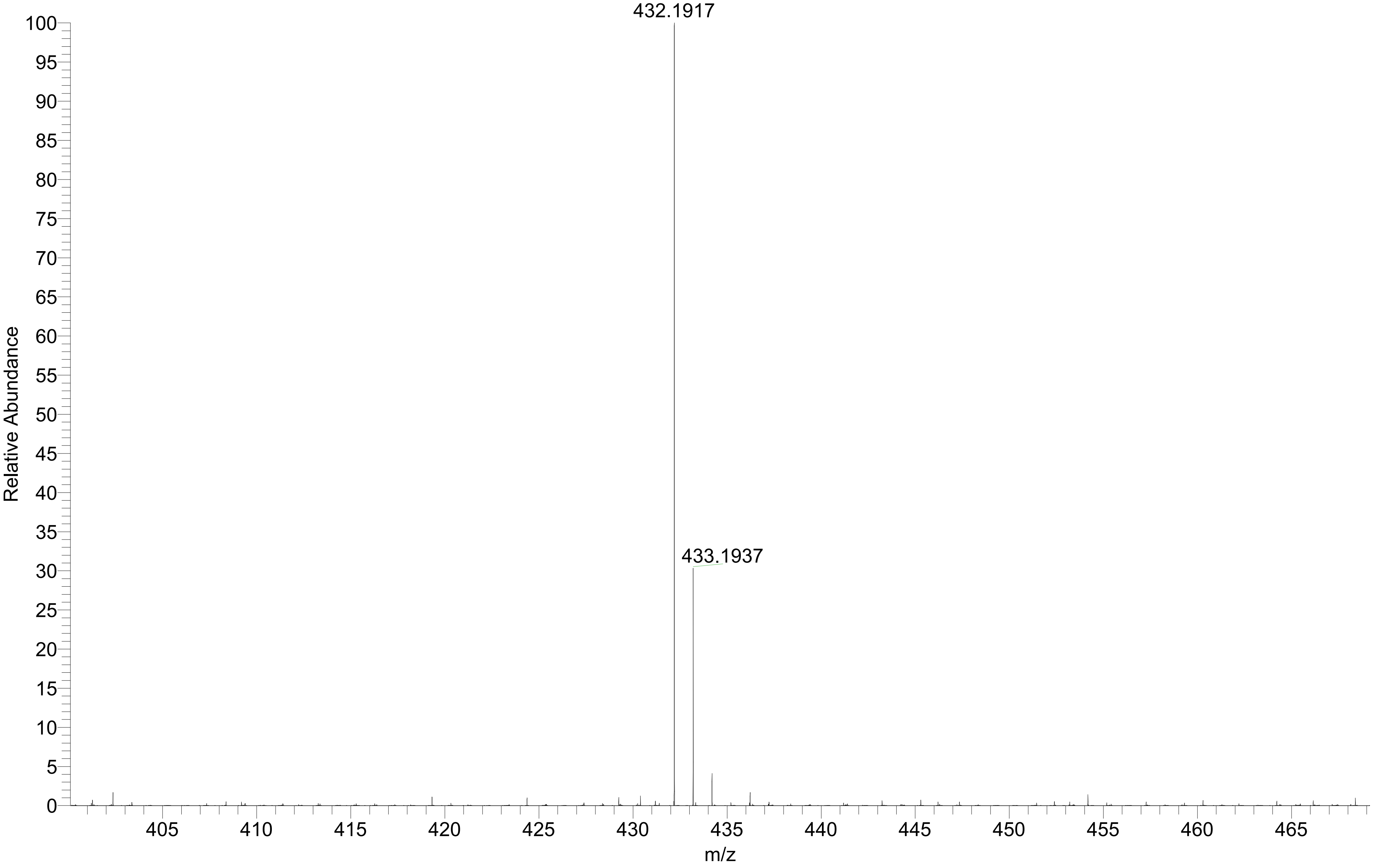

Report UHPLC

Acquisition Date/Time	7/15/2021 3:57:51 PM	Sample Name	M508-SS1020A
Acquisition Method	MS3	Injection Volume	2
Instrument Name	UHPLC		

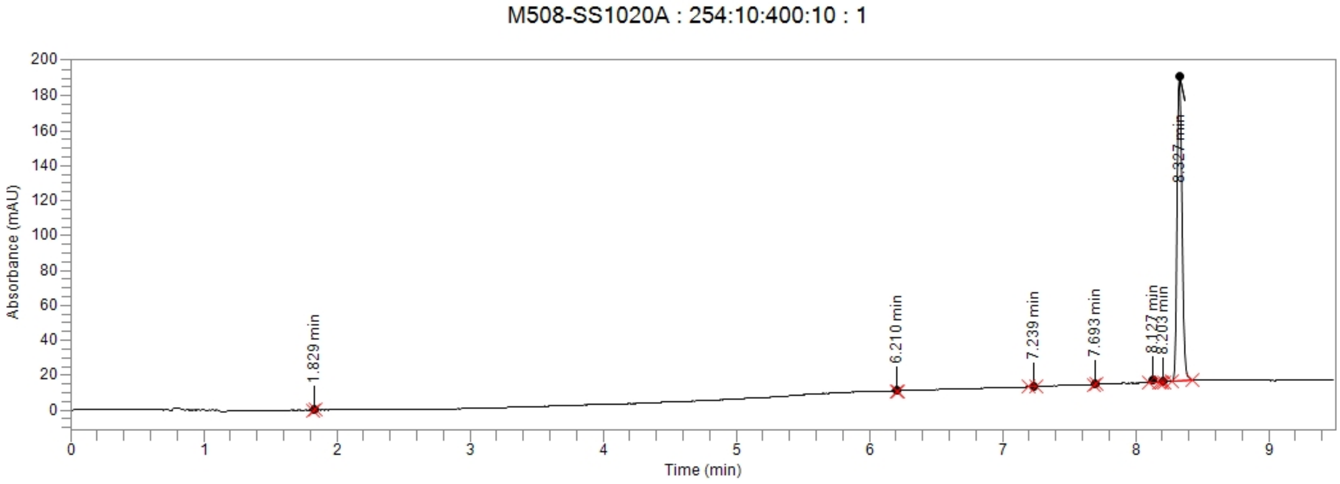

Channel Name **254:10:400:10**

Component Name	Time	Height	Area	Area %	Peak Purity	Wavelength Maximum
	1.829	132.0	53.2	0.01	1.77Fail	195
	6.210	147.3	46.2	0.01	1.24Pass	200
	7.239	180.2	447.1	0.10	1.15Pass	200
	7.693	156.3	79.0	0.02	1.14Pass	201
	8.127	1,554.1	3,695.2	0.82	1.22Pass	201
	8.203	137.2	73.7	0.02	1.11Pass	201
	8.327	174,554.0	448,281.0	99.03	1.10Pass	202
Total			452,675.3	100.00		

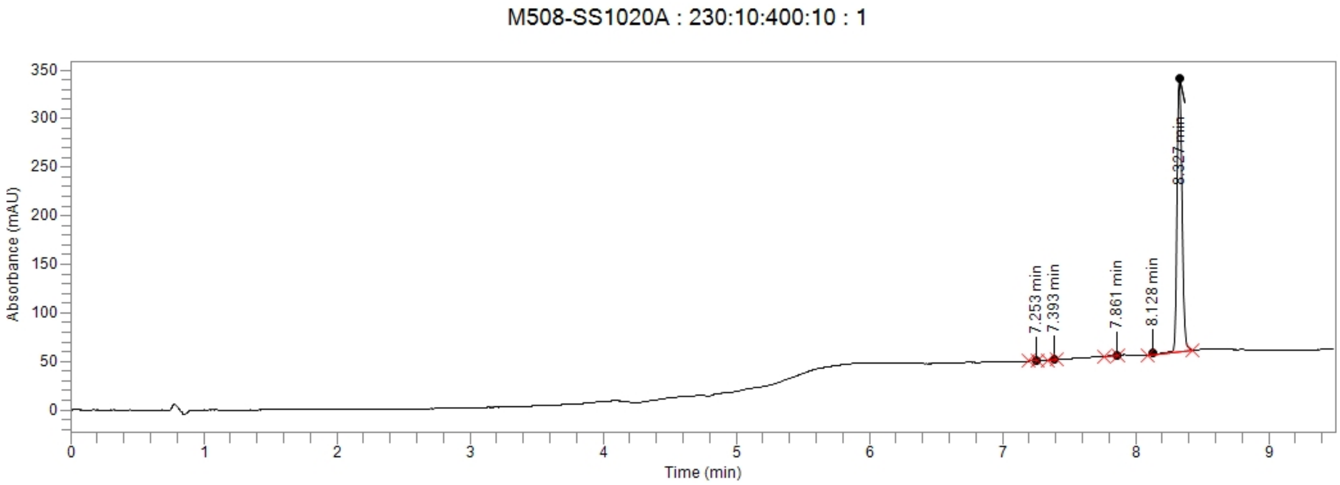

Channel Name **230:10:400:10**

Component Name	Time	Height	Area	Area %	Peak Purity	Wavelength Maximum
	7.253	210.0	851.7	0.12	1.07Pass	201
	7.393	243.0	835.1	0.11	1.14Pass	201
	7.861	237.4	1,415.9	0.19	1.12Pass	201
	8.128	2,136.7	5,293.2	0.72	1.13Pass	201
	8.327	281,656.2	727,282.9	98.86	1.11Pass	202
Total			735,678.8	100.00		

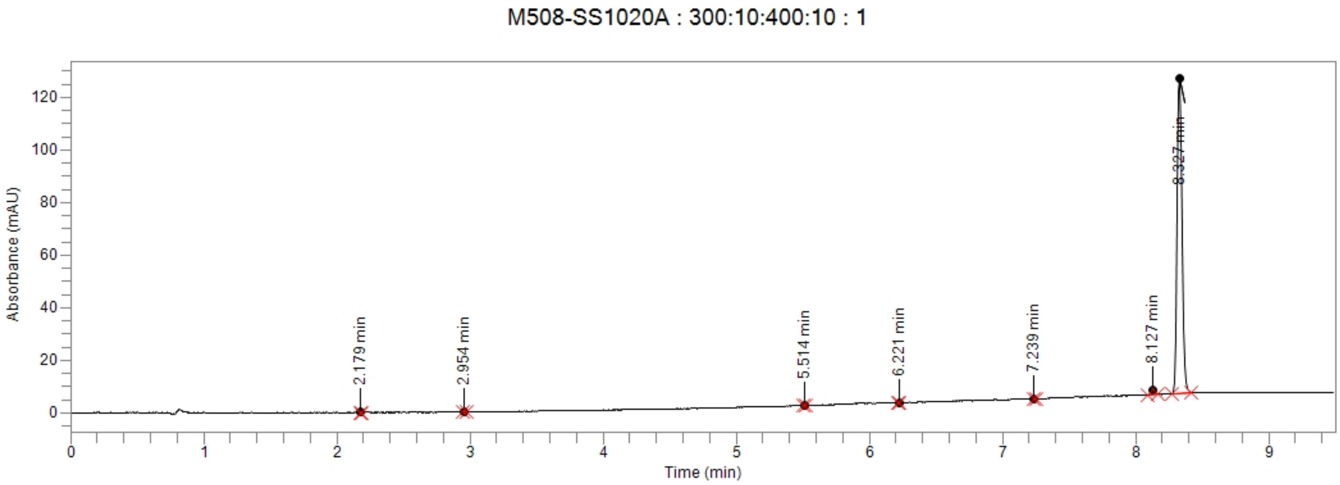

Channel Name **300:10:400:10**

Component Name	Time	Height	Area	Area %	Peak Purity	Wavelength Maximum
	2.179	146.7	51.1	0.02	7.95Fail	195
	2.954	150.3	75.8	0.02	Not Enough Valid Points	199
	5.514	135.7	55.6	0.02	1.17Pass	200
	6.221	151.6	56.9	0.02	1.14Pass	200
	7.239	163.6	79.1	0.03	1.17Pass	200
	8.127	1,535.4	3,416.5	1.10	1.22Pass	201
	8.327	119,817.7	306,949.9	98.80	1.10Pass	202
Total			310,685.0	100.00		

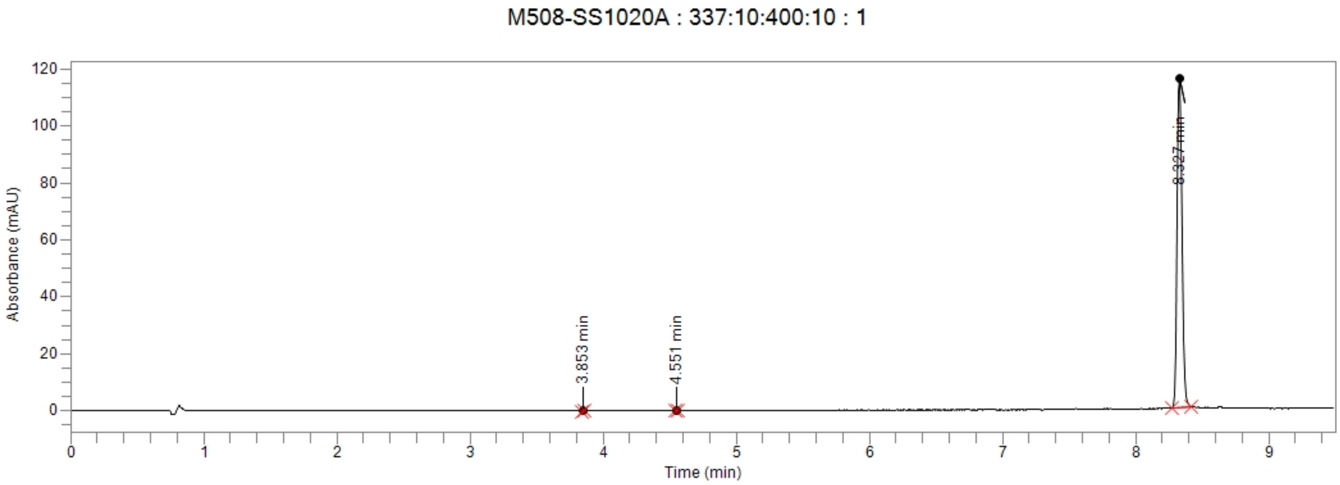

Channel Name **337:10:400:10**

Component Name	Time	Height	Area	Area %	Peak Purity	Wavelength Maximum
	3.853	183.0	152.8	0.05	Not Enough Valid Points	199
	4.551	187.1	97.0	0.03	2.14Fail	200
	8.327	115,689.7	296,627.3	99.92	1.11Pass	202
Total			296,877.1	100.00		

Report UHPLC

Acquisition Date/Time	7/15/2021 5:42:02 PM	Sample Name	M548-SS1220A
Acquisition Method	MS3	Injection Volume	2
Instrument Name	UHPLC		

M548-SS1220A : 254:10:400:10 : 1

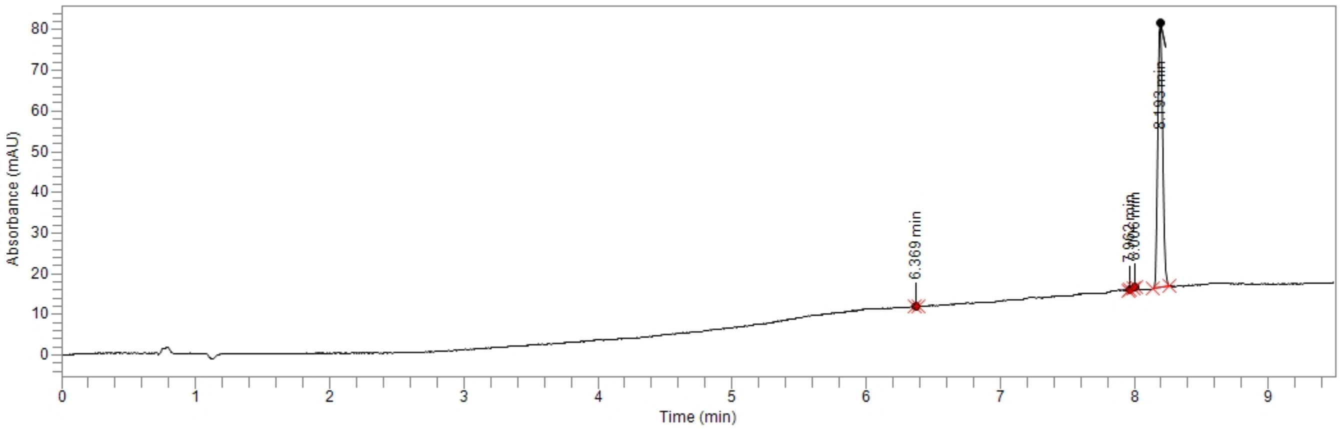

Channel Name **254:10:400:10**

Component Name	Time	Height	Area	Area %	Peak Purity	Wavelength Maximum
	6.369	165.5	106.5	0.06	1.08Pass	200
	7.962	161.0	54.4	0.03	1.05Pass	201
	8.006	215.6	159.4	0.10	1.06Pass	201
	8.193	65,195.1	163,726.8	99.80	1.10Pass	201
Total			164,047.0	100.00		

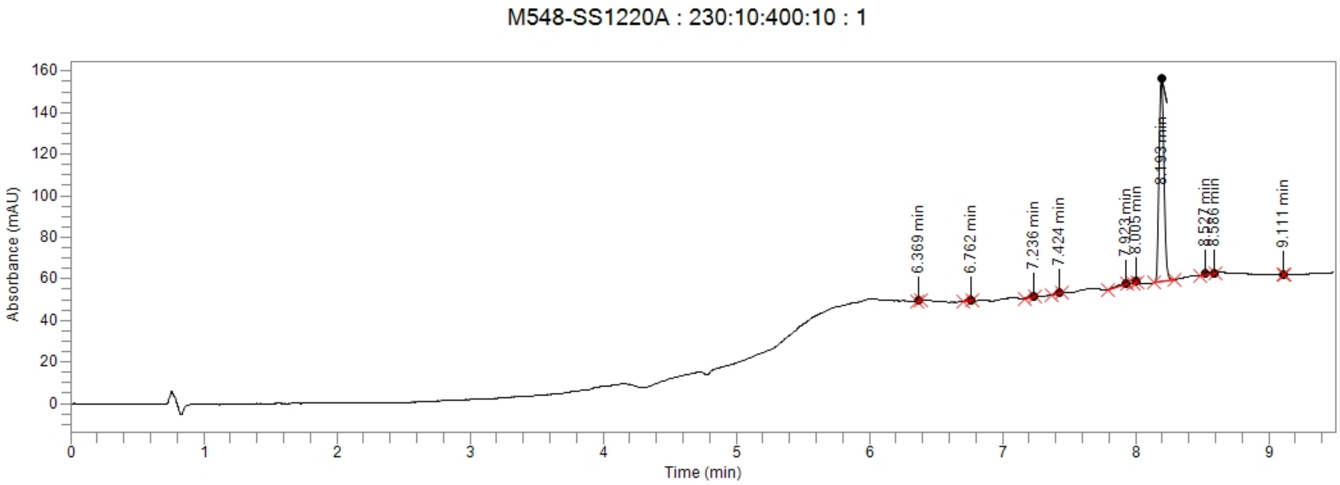

Channel Name **230:10:400:10**

Component Name	Time	Height	Area	Area %	Peak Purity	Wavelength Maximum
	6.369	162.8	104.4	0.04	1.08Pass	200
	6.762	261.3	754.8	0.30	1.13Pass	200
	7.236	177.5	1,013.2	0.40	1.10Pass	201
	7.424	201.5	789.6	0.31	1.07Pass	201
	7.923	252.6	2,975.4	1.17	1.14Pass	201
	8.005	152.4	204.1	0.08	1.14Pass	201
	8.193	98,060.7	248,150.9	97.19	1.10Pass	201
	8.527	334.7	719.7	0.28	1.13Pass	201
	8.586	176.4	572.4	0.22	1.12Pass	201
	9.111	148.9	53.1	0.02	1.22Pass	201
Total			255,337.7	100.00		

M548-SS1220A : 300:10:400:10 : 1

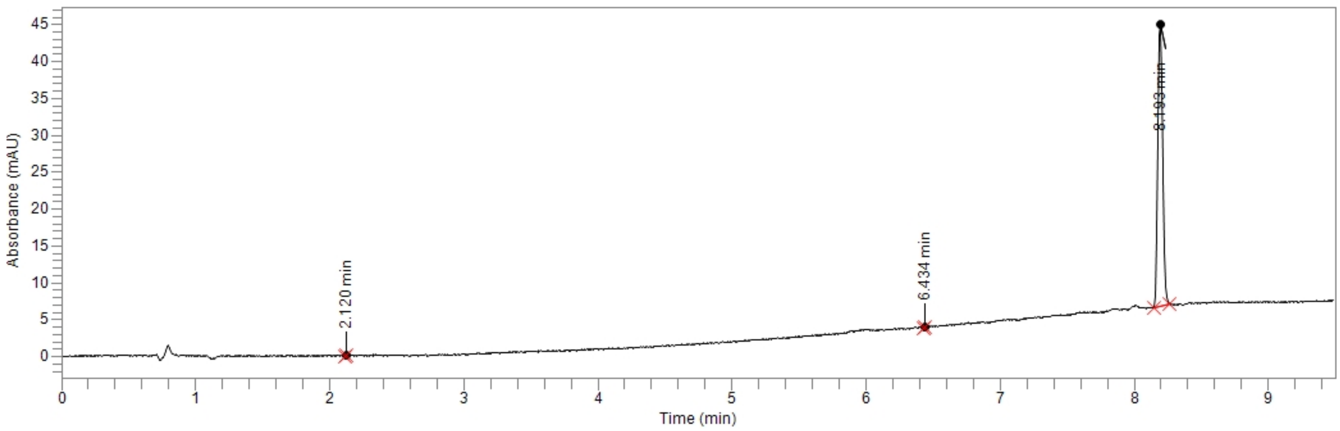

Channel Name **300:10:400:10**

Component Name	Time	Height	Area	Area %	Peak Purity	Wavelength Maximum
	2.120	121.9	38.6	0.04	4.02Fail	694
	6.434	129.0	47.3	0.05	1.32Pass	200
	8.193	38,216.2	95,851.1	99.91	1.10Pass	201
Total			95,937.0	100.00		

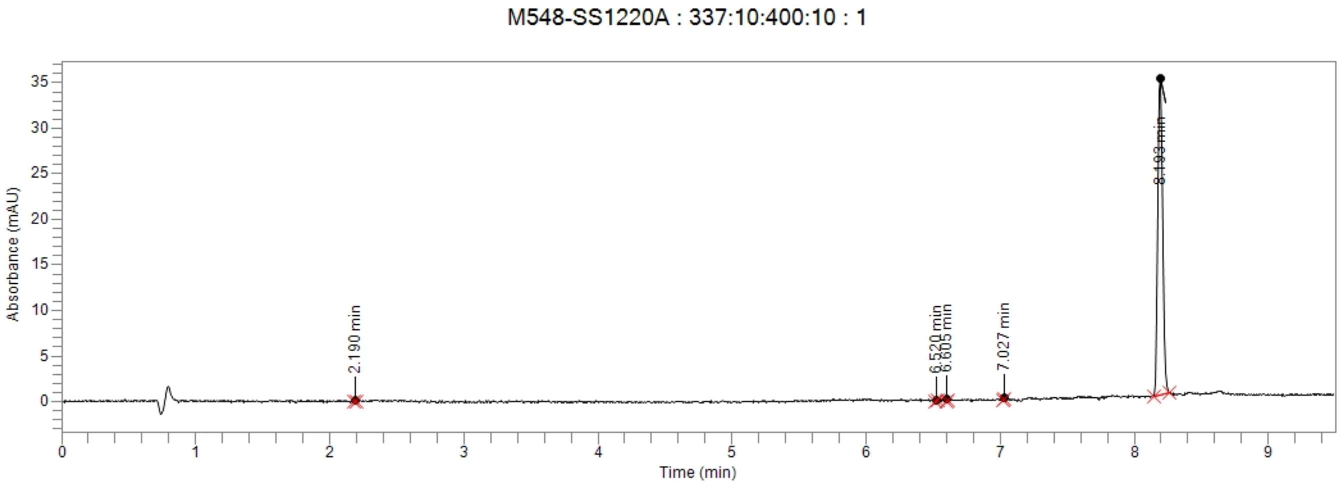

Channel Name **337:10:400:10**

Component Name	Time	Height	Area	Area %	Peak Purity	Wavelength Maximum
	2.190	175.3	86.8	0.10	7.16Fail	195
	6.520	159.3	82.1	0.09	1.21Pass	200
	6.605	133.8	45.0	0.05	1.12Pass	200
	7.027	169.1	62.6	0.07	1.13Pass	201
	8.193	34,847.3	87,401.6	99.68	1.10Pass	201
Total			87,678.0	100.00		

Report UHPLC

Acquisition Date/Time	7/15/2021 2:39:40 PM	Sample Name	M549-SS1250B
Acquisition Method	MS3	Injection Volume	2
Instrument Name	UHPLC		

M549-SS1250B : 254:10:400:10 : 1

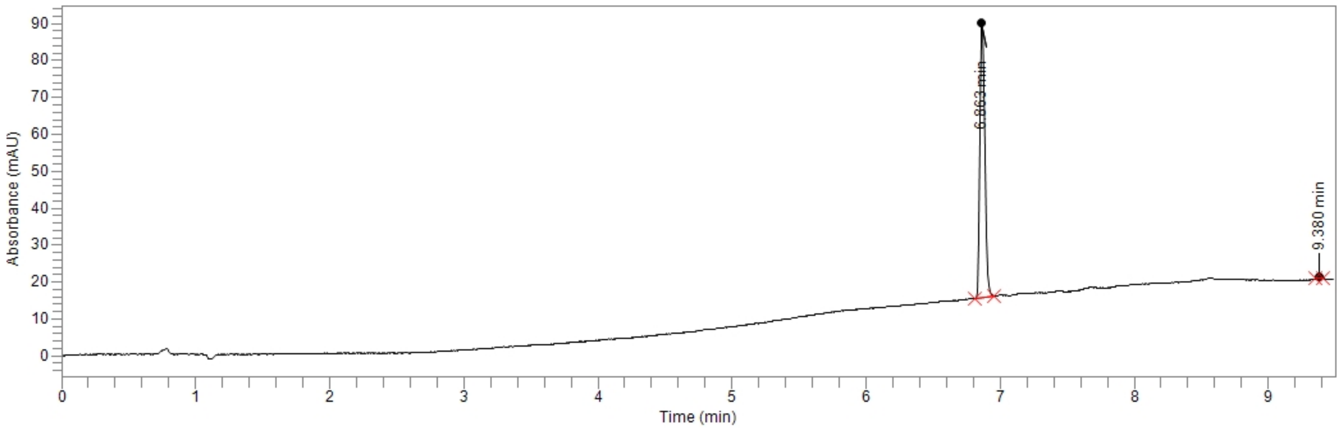

Channel Name **254:10:400:10**

Component Name	Time	Height	Area	Area %	Peak Purity	Wavelength Maximum
	6.863	74,574.7	192,764.6	99.46	1.18Pass	200
	9.380	500.6	1,037.3	0.54	1.09Pass	201
Total			193,801.9	100.00		

M549-SS1250B : 230:10:400:10 : 1

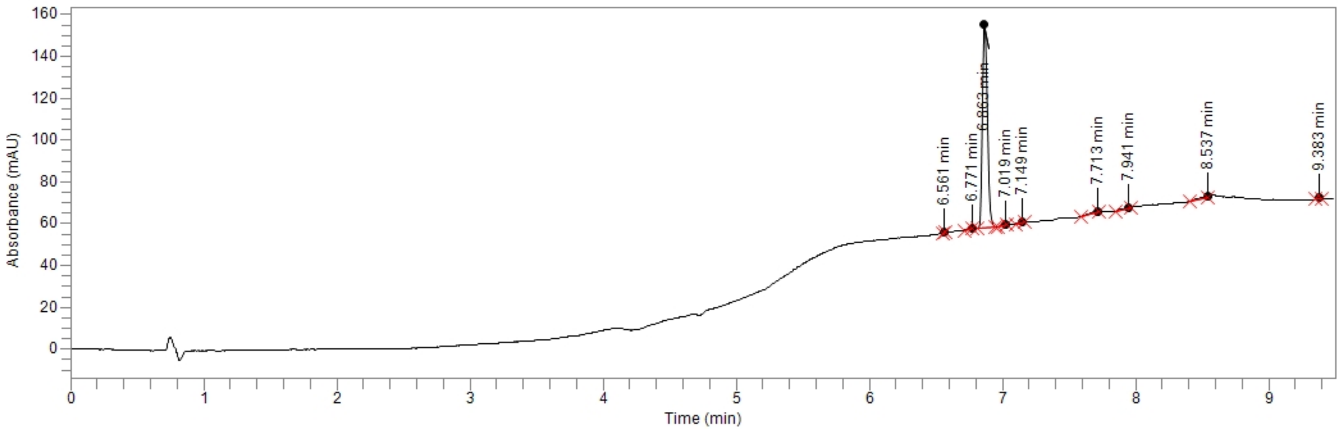

Channel Name **230:10:400:10**

Component Name	Time	Height	Area	Area %	Peak Purity	Wavelength Maximum
	6.561	171.6	71.7	0.03	1.13Pass	200
	6.771	150.8	558.2	0.21	1.17Pass	200
	6.863	97,534.5	252,011.2	96.35	1.18Pass	200
	7.019	241.5	706.9	0.27	1.21Pass	200
	7.149	187.5	426.4	0.16	1.17Pass	200
	7.713	223.0	2,770.8	1.06	1.18Pass	200
	7.941	291.4	1,317.2	0.50	1.11Pass	200
	8.537	152.0	2,865.0	1.10	1.17Pass	201
	9.383	445.0	834.5	0.32	1.09Pass	201
Total			261,561.9	100.00		

M549-SS1250B : 300:10:400:10 : 1

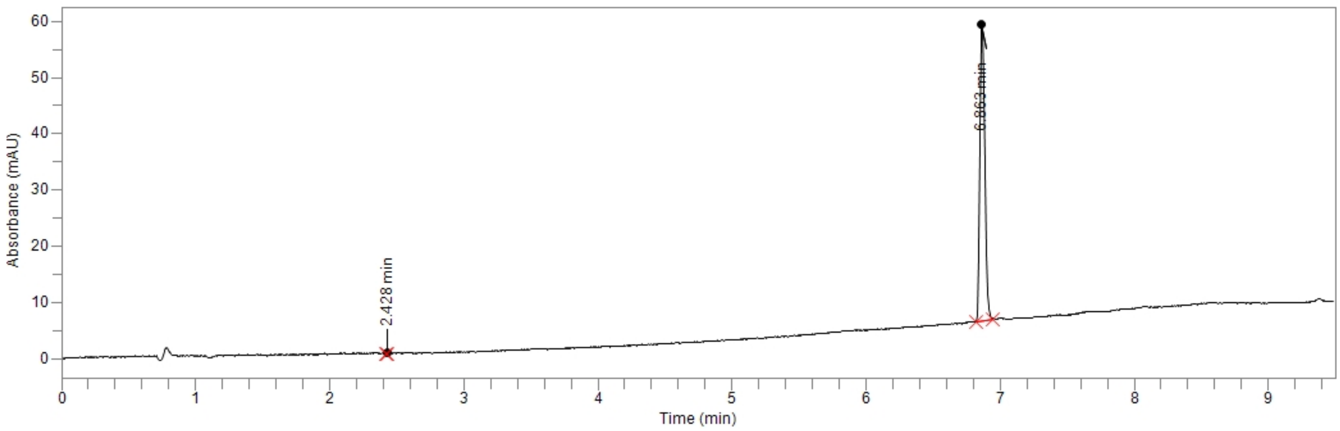

Channel Name **300:10:400:10**

Component Name	Time	Height	Area	Area %	Peak Purity	Wavelength Maximum
	2.428	172.7	61.3	0.04	4.89Fail	269
	6.863	52,816.5	136,192.3	99.96	1.18Pass	200
Total			136,253.6	100.00		

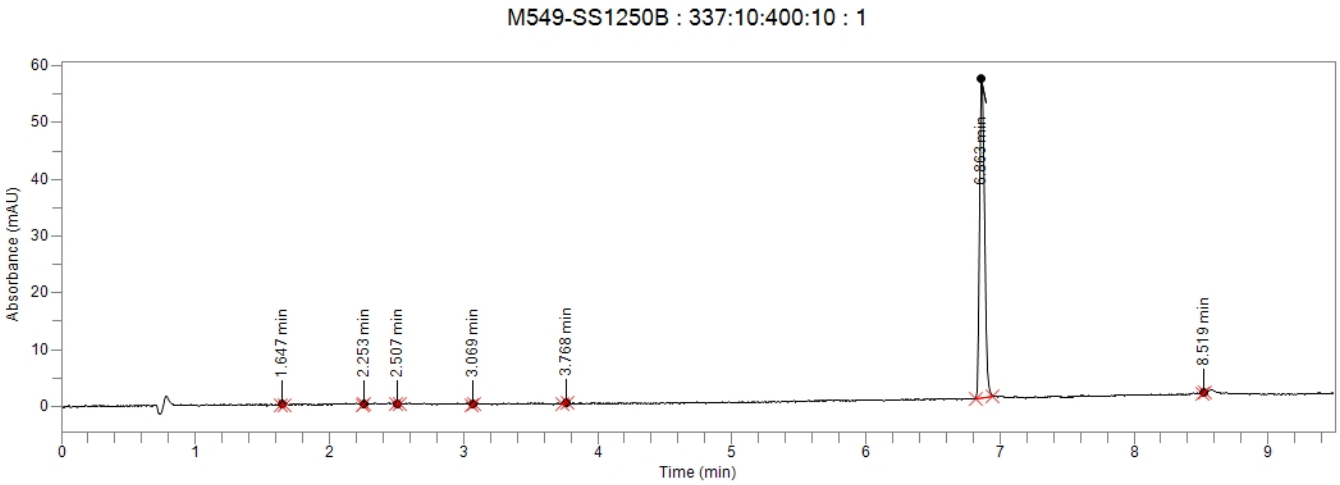

Channel Name **337:10:400:10**

Component Name	Time	Height	Area	Area %	Peak Purity	Wavelength Maximum
	1.647	192.3	102.8	0.07	2.54Fail	191
	2.253	226.7	95.5	0.07	2.32Fail	191
	2.507	141.0	138.4	0.09	2.14Fail	191
	3.069	189.6	67.5	0.05	Not Enough Valid Points	199
	3.768	96.1	197.5	0.14	1.60Fail	199
	6.863	56,255.0	145,226.5	99.55	1.18Pass	200
	8.519	145.0	54.3	0.04	1.10Pass	201
Total			145,882.6	100.00		

Report UHPLC

Acquisition Date/Time	7/15/2021 2:52:36 PM	Sample Name	M550-SS1252B
Acquisition Method	MS3	Injection Volume	2
Instrument Name	UHPLC		

M550-SS1252B : 254:10:400:10 : 1

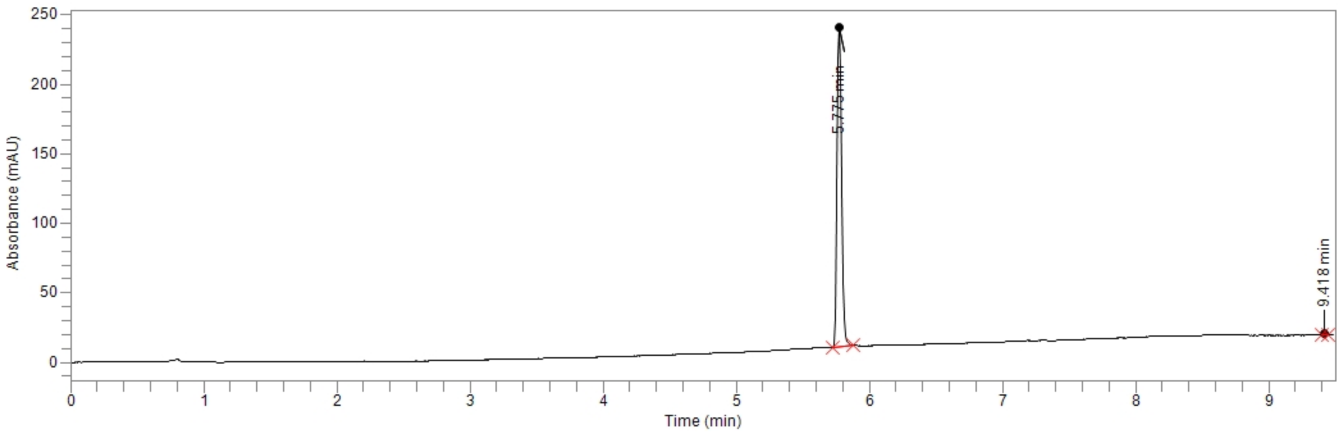

Channel Name 254:10:400:10

Component Name	Time	Height	Area	Area %	Peak Purity	Wavelength Maximum
	5.775	230,016.4	550,714.0	99.82	1.16Pass	200
	9.418	509.3	1,002.9	0.18	1.08Pass	201
Total			551,716.9	100.00		

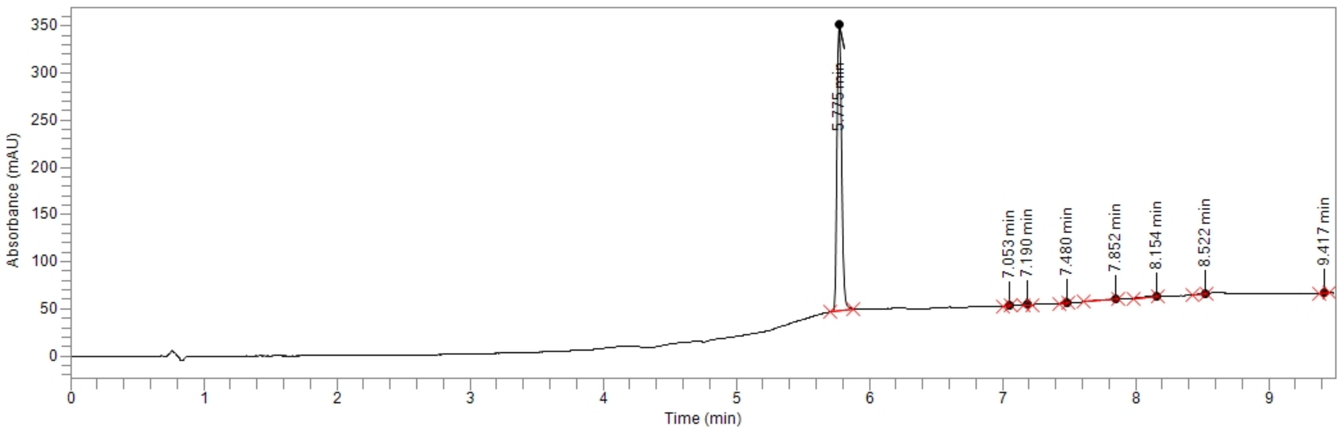

Channel Name **230:10:400:10**

Component Name	Time	Height	Area	Area %	Peak Purity	Wavelength Maximum
	5.775	304,150.1	730,977.9	98.08	1.11Pass	200
	7.053	238.9	778.4	0.10	1.22Pass	200
	7.190	533.1	1,037.2	0.14	1.11Pass	200
	7.480	226.7	809.5	0.11	1.08Pass	200
	7.852	147.5	5,050.4	0.68	1.13Pass	200
	8.154	214.3	3,066.7	0.41	1.09Pass	201
	8.522	191.7	1,334.1	0.18	1.09Pass	201
	9.417	876.9	2,202.9	0.30	1.16Pass	201
Total			745,257.1	100.00		

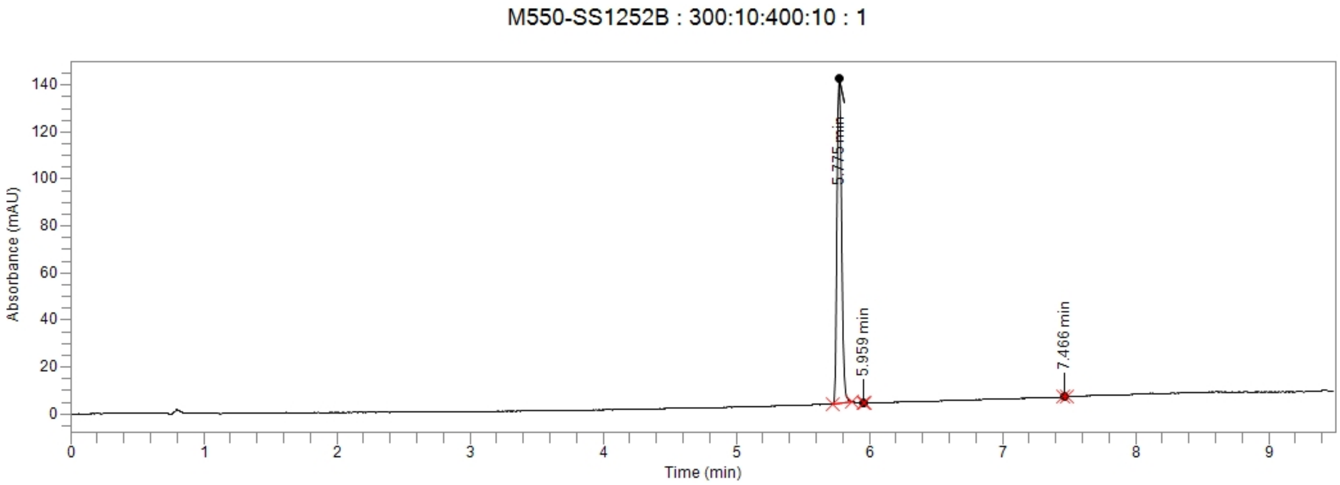

Channel Name **300:10:400:10**

Component Name	Time	Height	Area	Area %	Peak Purity	Wavelength Maximum
	5.775	138,506.2	330,956.0	99.94	1.16Pass	200
	5.959	129.5	47.6	0.01	1.10Pass	200
	7.466	212.3	144.0	0.04	1.16Pass	200
Total			331,147.6	100.00		

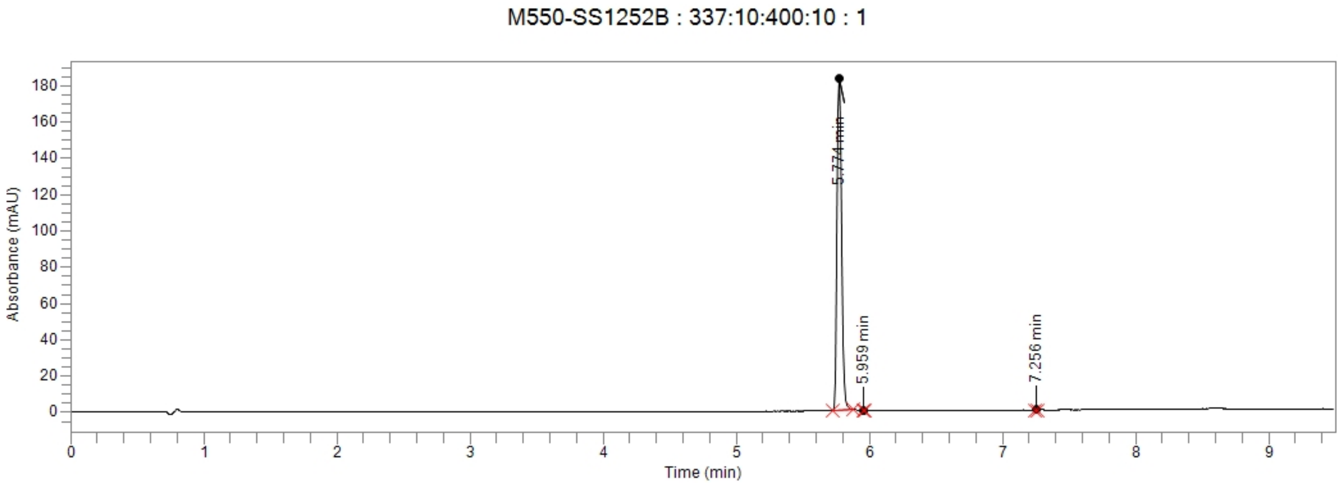

Channel Name **337:10:400:10**

Component Name	Time	Height	Area	Area %	Peak Purity	Wavelength Maximum
	5.774	183,310.4	439,577.0	99.97	1.16Pass	200
	5.959	159.1	55.8	0.01	1.11Pass	200
	7.256	171.8	56.9	0.01	1.10Pass	200
Total			439,689.7	100.00		

Report UHPLC

Acquisition Date/Time	7/15/2021 3:05:38 PM	Sample Name	M551-SS1254B
Acquisition Method	MS3	Injection Volume	2
Instrument Name	UHPLC		

M551-SS1254B : 254:10:400:10 : 1

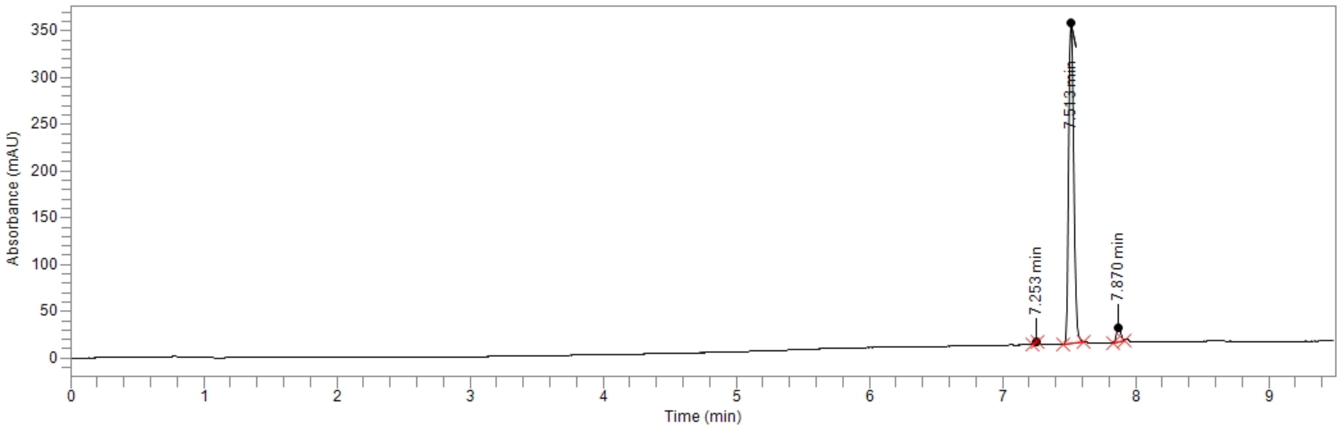

Channel Name **254:10:400:10**

Component Name	Time	Height	Area	Area %	Peak Purity	Wavelength Maximum
	7.253	557.9	900.0	0.10	1.20Pass	200
	7.513	342,902.6	908,370.3	96.16	1.15Pass	201
	7.870	14,441.6	35,348.9	3.74	1.09Pass	201
Total			944,619.2	100.00		

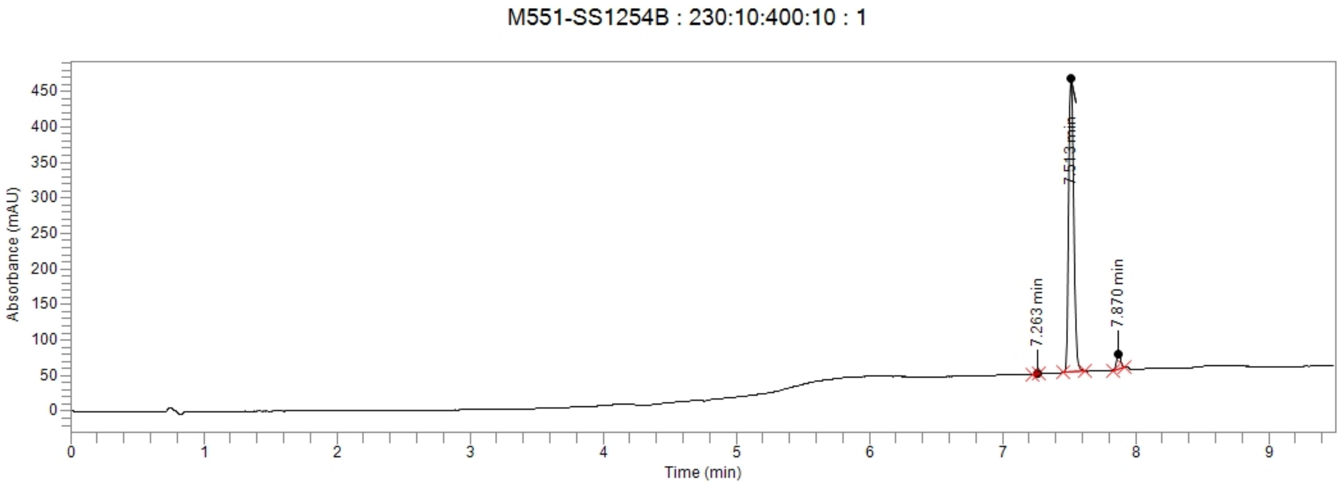

Channel Name **230:10:400:10**

Component Name	Time	Height	Area	Area %	Peak Purity	Wavelength Maximum
	7.263	680.9	1,807.3	0.16	1.07Pass	200
	7.513	413,894.3	1,097,680.5	95.54	1.15Pass	201
	7.870	20,148.1	49,483.9	4.31	1.09Pass	201
Total			1,148,971.6	100.00		

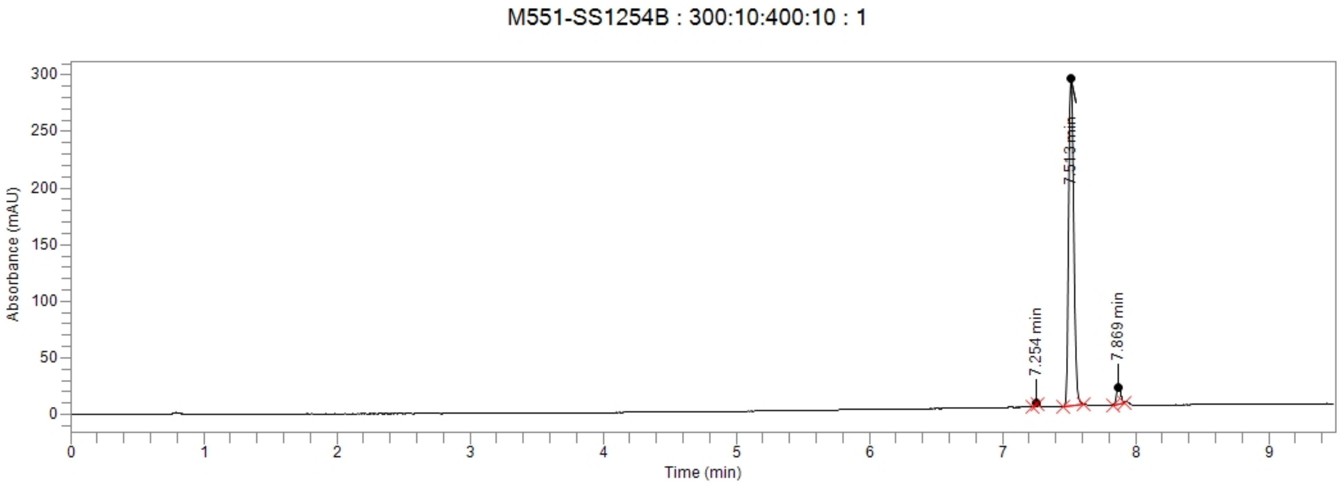

Channel Name **300:10:400:10**

Component Name	Time	Height	Area	Area %	Peak Purity	Wavelength Maximum
	7.254	852.1	1,342.2	0.17	1.16Pass	200
	7.513	289,587.6	767,091.9	95.26	1.15Pass	201
	7.869	15,074.1	36,818.6	4.57	1.25Pass	201
Total			805,252.7	100.00		

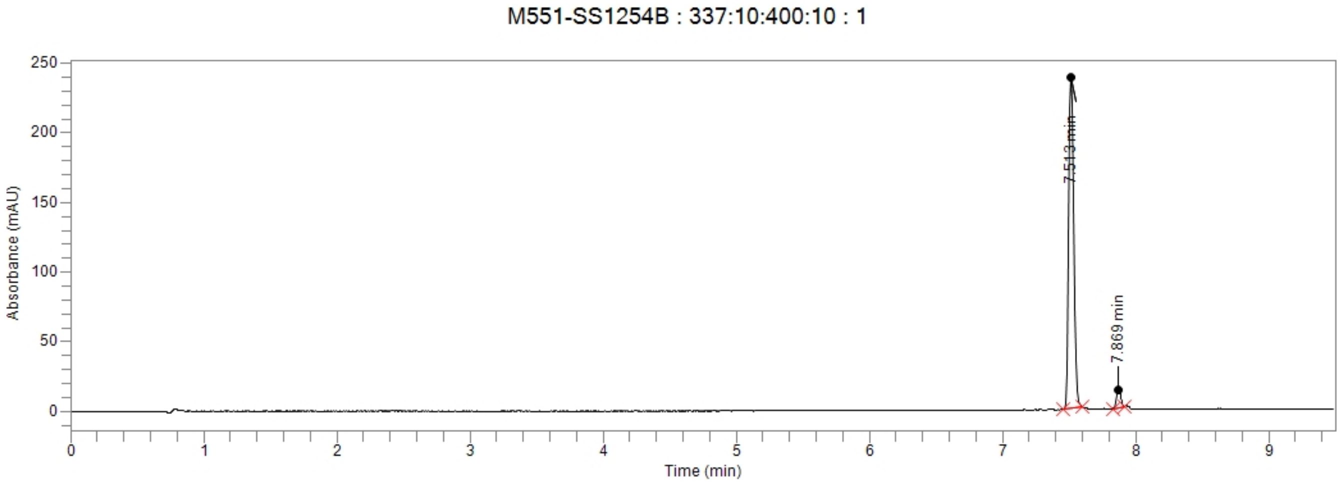

Channel Name **337:10:400:10**

Component Name	Time	Height	Area	Area %	Peak Purity	Wavelength Maximum
	7.513	238,406.5	630,659.6	95.23	1.15Pass	201
	7.869	12,918.0	31,590.4	4.77	1.09Pass	201
Total			662,250.0	100.00		

Report UHPLC

Acquisition Date/Time	7/15/2021 3:18:44 PM	Sample Name	M552-SS1256B
Acquisition Method	MS3	Injection Volume	2
Instrument Name	UHPLC		

M552-SS1256B : 254:10:400:10 : 1

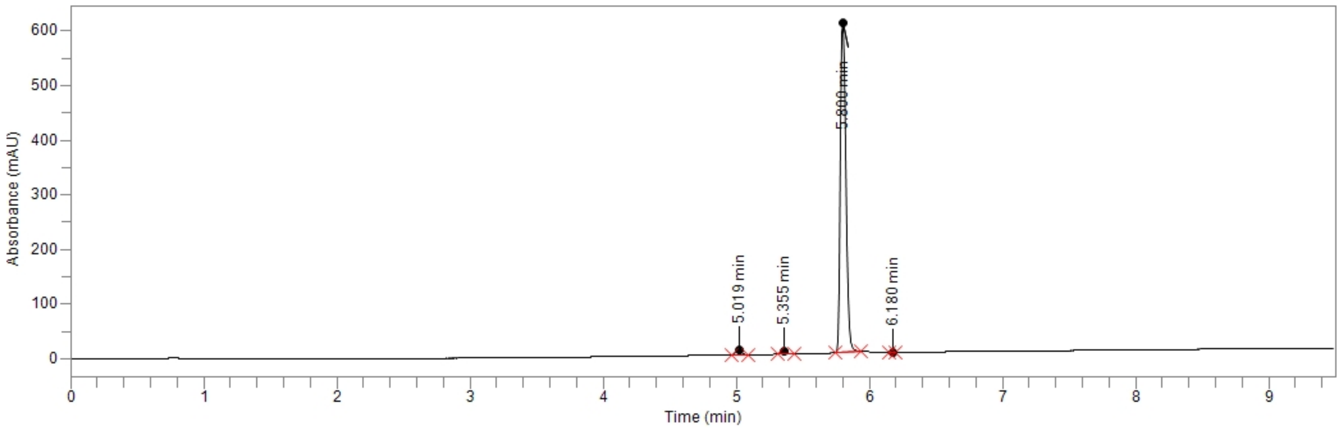

Channel Name **254:10:400:10**

Component Name	Time	Height	Area	Area %	Peak Purity	Wavelength Maximum
	5.019	10,175.5	28,264.1	1.58	1.22Pass	200
	5.355	5,717.8	15,975.4	0.89	1.24Pass	200
	5.800	604,660.5	1,749,365.4	97.50	1.14Pass	201
	6.180	384.7	691.2	0.04	1.26Pass	200
Total			1,794,296.2	100.00		

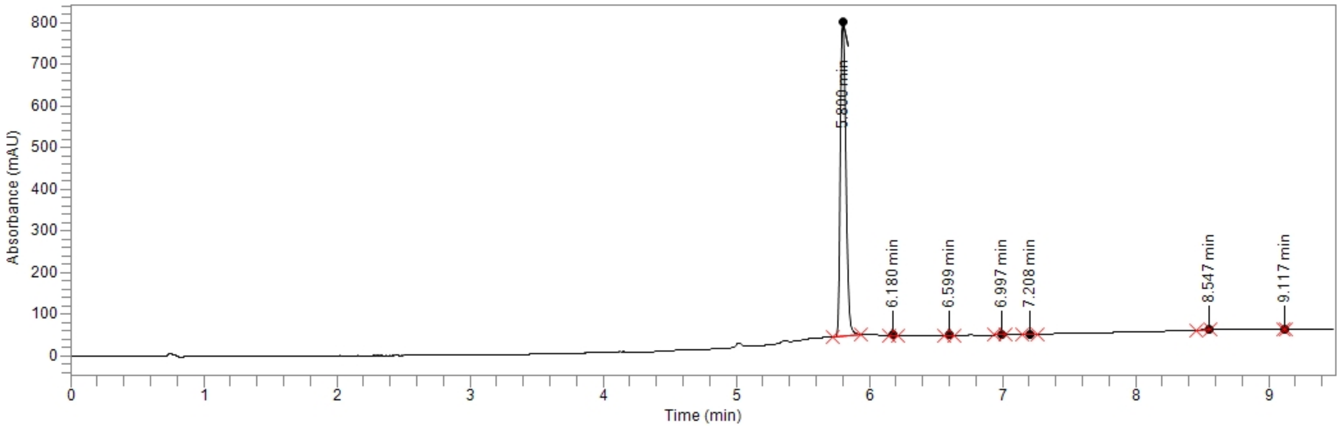

Channel Name **230:10:400:10**

Component Name	Time	Height	Area	Area %	Peak Purity	Wavelength Maximum
	5.800	755,288.3	2,190,334.1	99.40	1.14Pass	201
	6.180	906.3	2,151.2	0.10	1.49Pass	200
	6.599	888.0	2,064.4	0.09	1.36Pass	200
	6.997	545.8	1,952.9	0.09	1.12Pass	200
	7.208	1,475.5	5,067.9	0.23	1.21Pass	200
	8.547	278.7	1,828.6	0.08	1.17Pass	201
	9.117	152.9	85.1	0.00	1.25Pass	201
Total			2,203,484.3	100.00		

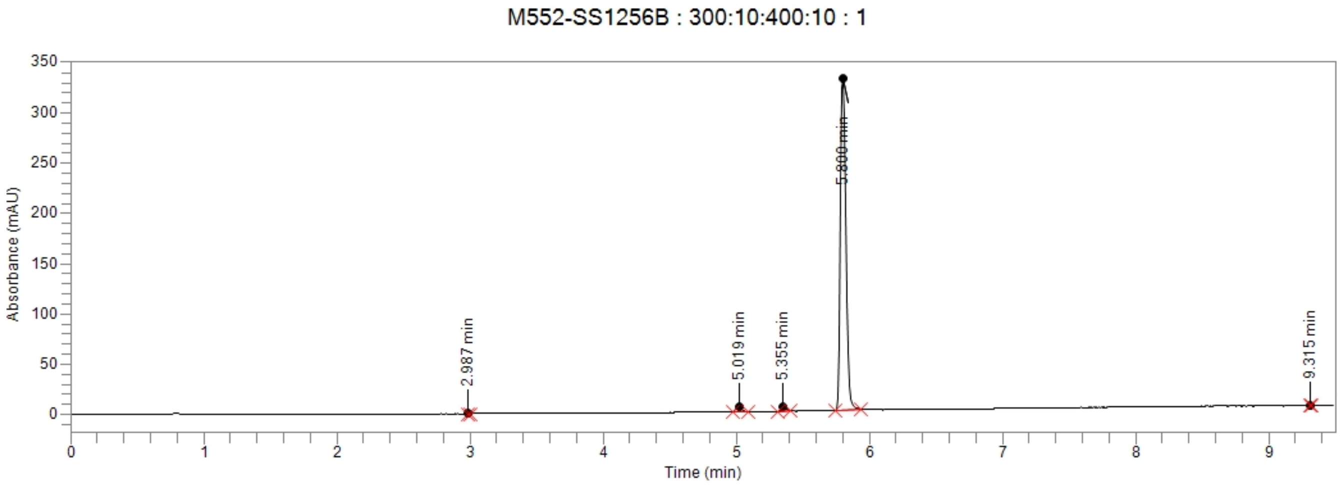

Channel Name **300:10:400:10**

Component Name	Time	Height	Area	Area %	Peak Purity	Wavelength Maximum
	2.987	158.1	122.6	0.01	Not Enough Valid Points	199
	5.019	4,929.9	13,541.1	1.38	1.22Pass	200
	5.355	4,013.9	10,706.0	1.09	1.36Pass	200
	5.800	330,577.5	956,143.4	97.51	1.14Pass	201
	9.315	154.9	56.3	0.01	1.09Pass	201
Total			980,569.3	100.00		

M552-SS1256B : 337:10:400:10 : 1

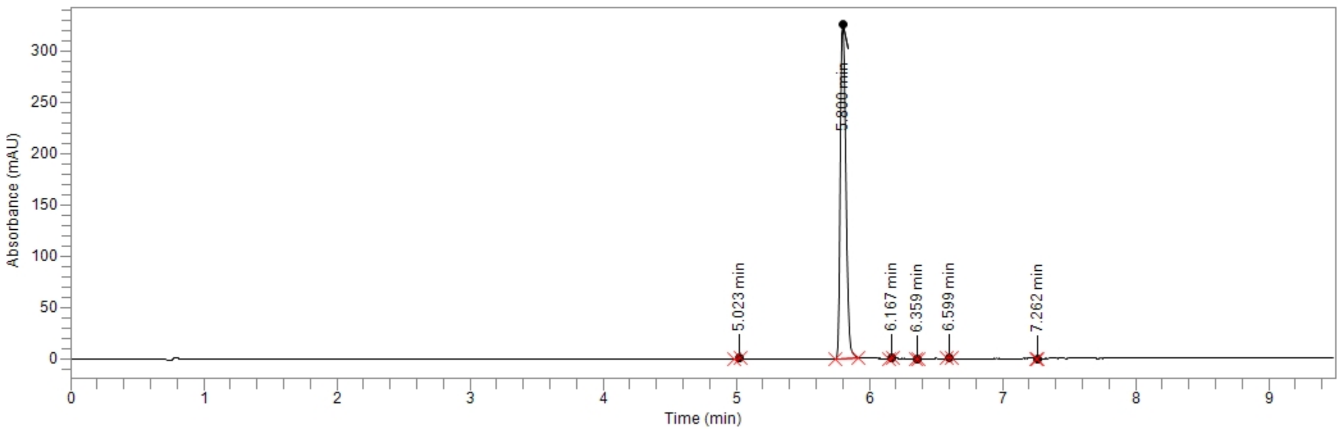

Channel Name **337:10:400:10**

Component Name	Time	Height	Area	Area %	Peak Purity	Wavelength Maximum
	5.023	438.0	912.9	0.10	1.43Pass	199
	5.800	325,705.0	941,521.7	99.84	1.14Pass	201
	6.167	253.2	186.6	0.02	1.33Pass	200
	6.359	153.4	62.9	0.01	1.14Pass	200
	6.599	232.8	275.1	0.03	1.08Pass	200
	7.262	151.0	51.4	0.01	1.18Pass	200
Total			943,010.6	100.00		

Report UHPLC

Acquisition Date/Time	7/15/2021 3:31:41 PM	Sample Name	M553-SS1312C
Acquisition Method	MS3	Injection Volume	2
Instrument Name	UHPLC		

M553-SS1312C : 254:10:400:10 : 1

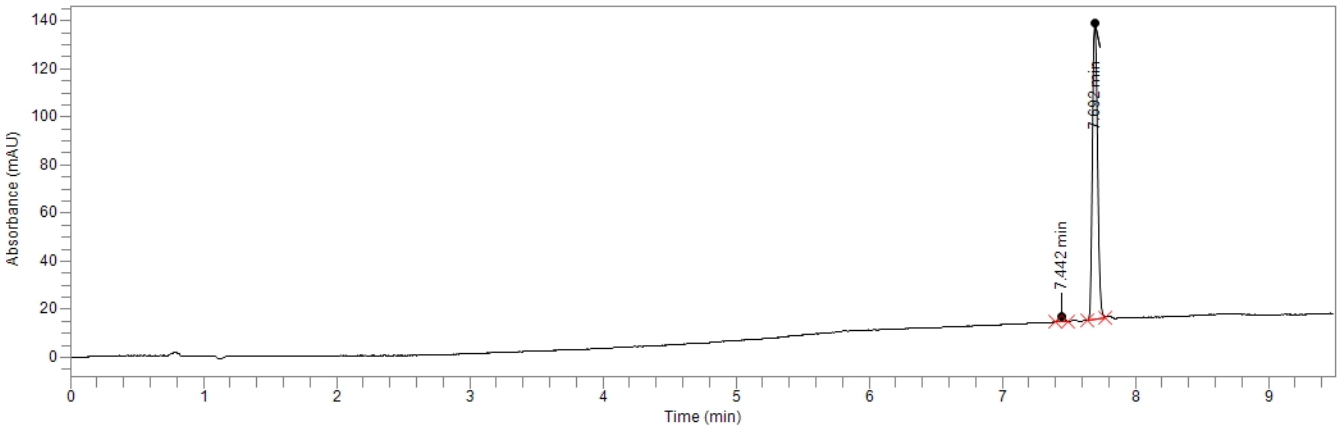

Channel Name 254:10:400:10

Component Name	Time	Height	Area	Area %	Peak Purity	Wavelength Maximum
	7.442	2,183.1	5,921.6	1.80	1.15Pass	200
	7.692	123,244.2	322,148.1	98.20	1.24Pass	201
Total			328,069.7	100.00		

M553-SS1312C : 230:10:400:10 : 1

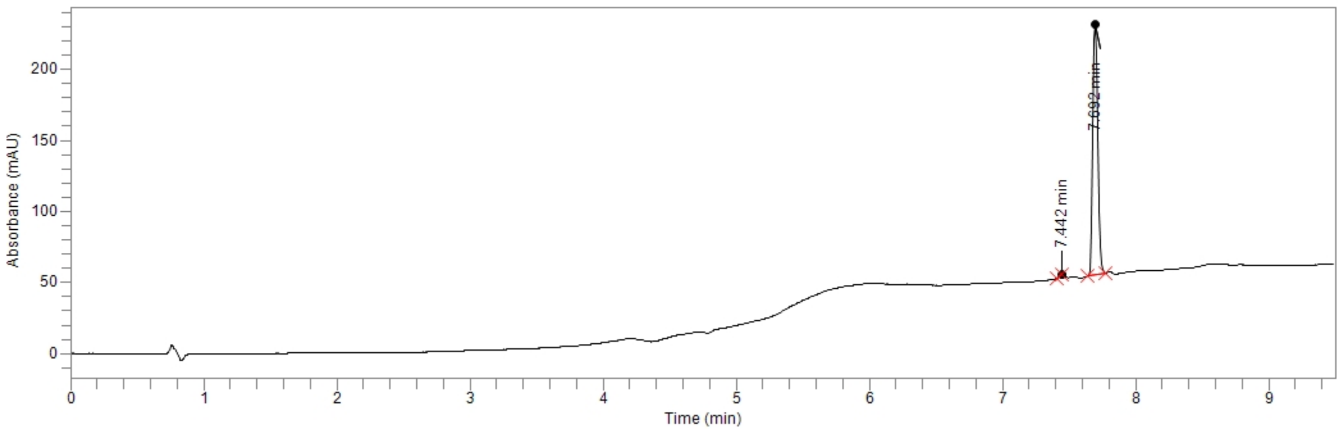

Channel Name **230:10:400:10**

Component Name	Time	Height	Area	Area %	Peak Purity	Wavelength Maximum
	7.442	592.7	1,138.3	0.25	1.10Pass	200
	7.692	176,566.9	461,042.1	99.75	1.24Pass	201
Total			462,180.4	100.00		

M553-SS1312C : 300:10:400:10 : 1

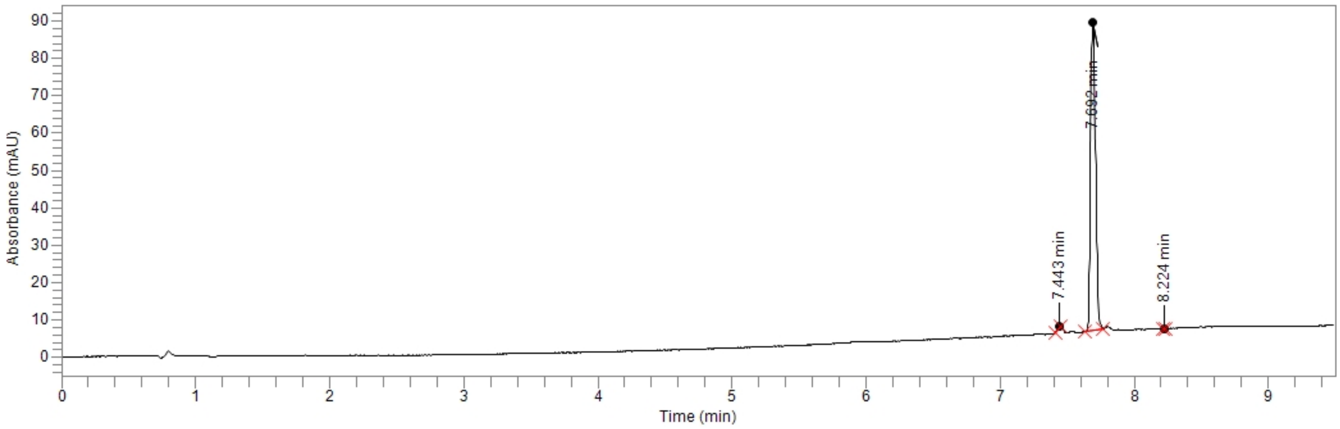

Channel Name **300:10:400:10**

Component Name	Time	Height	Area	Area %	Peak Purity	Wavelength Maximum
	7.443	413.5	631.5	0.29	1.28Pass	200
	7.692	82,487.9	214,911.1	99.67	1.24Pass	201
	8.224	184.7	77.0	0.04	1.09Pass	201
Total			215,619.5	100.00		

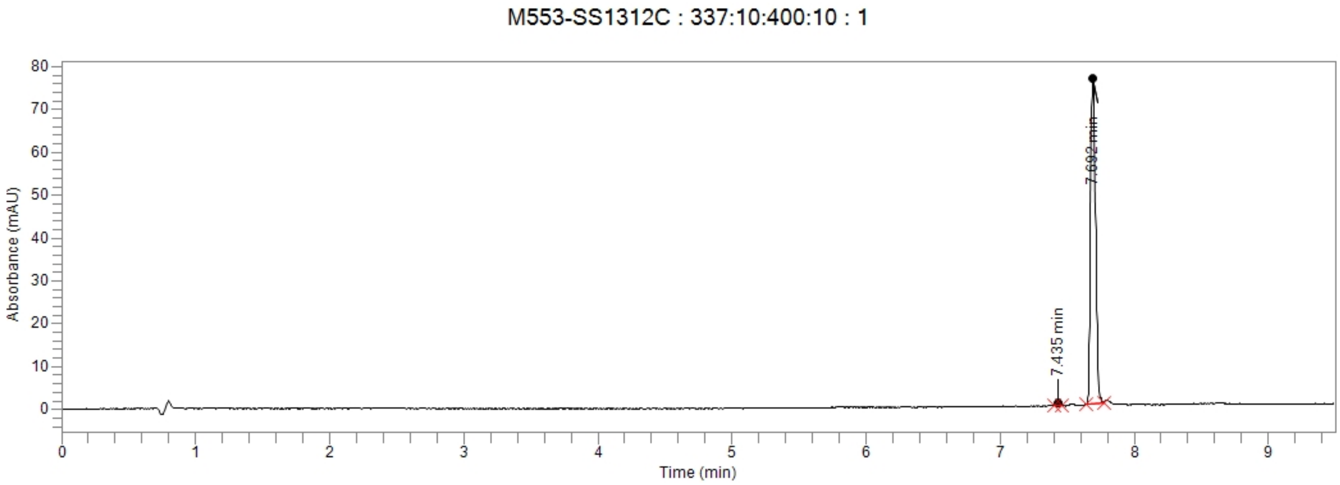

Channel Name **337:10:400:10**

Component Name	Time	Height	Area	Area %	Peak Purity	Wavelength Maximum
	7.435	686.2	1,663.3	0.83	1.15Pass	200
	7.692	76,003.9	197,954.6	99.17	1.24Pass	201
Total			199,617.9	100.00		

Report UHPLC

Acquisition Date/Time	7/15/2021 3:44:39 PM	Sample Name	M554-SS1318B
Acquisition Method	MS3	Injection Volume	2
Instrument Name	UHPLC		

M554-SS1318B : 254:10:400:10 : 1

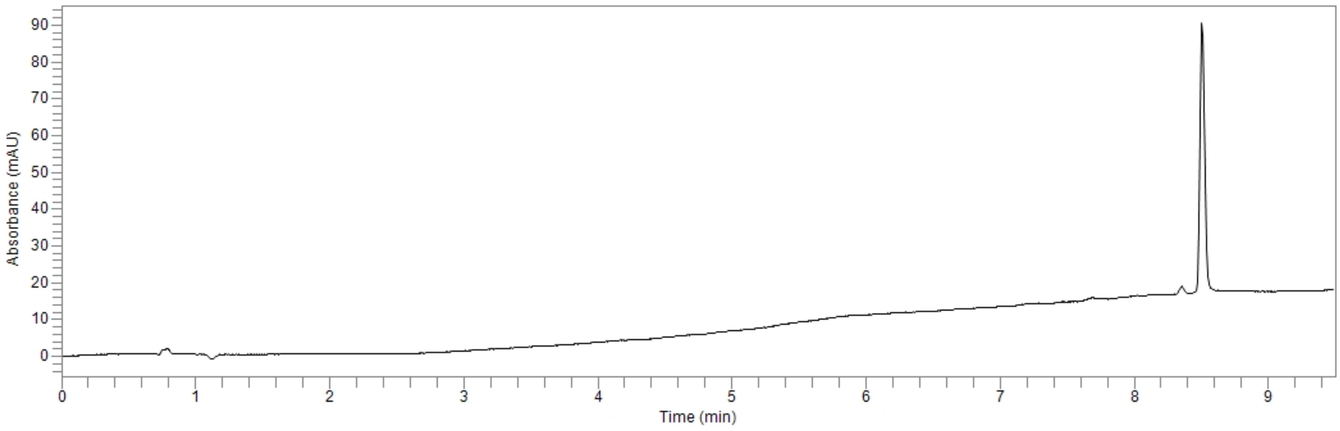

Channel Name **254:10:400:10**

Component Name	Time	Height	Area	Area %	Peak Purity	Wavelength Maximum
Total						

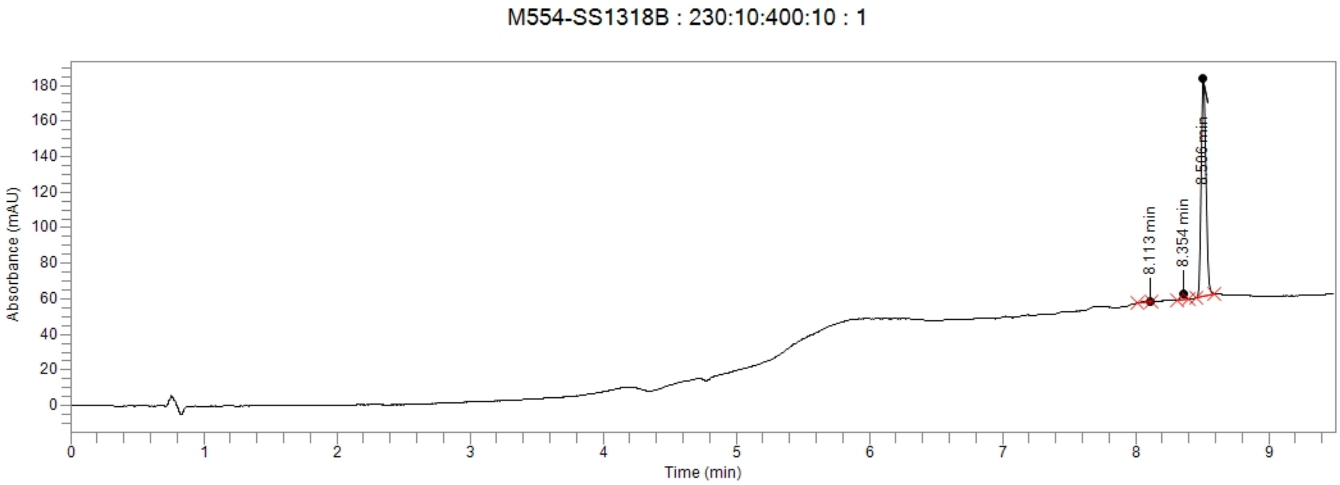

Channel Name **230:10:400:10**

Component Name	Time	Height	Area	Area %	Peak Purity	Wavelength Maximum
	8.113	154.5	953.7	0.31	1.16Pass	201
	8.354	2,967.4	7,037.3	2.27	1.14Pass	201
	8.506	122,815.4	301,602.2	97.42	1.09Pass	201
Total			309,593.3	100.00		

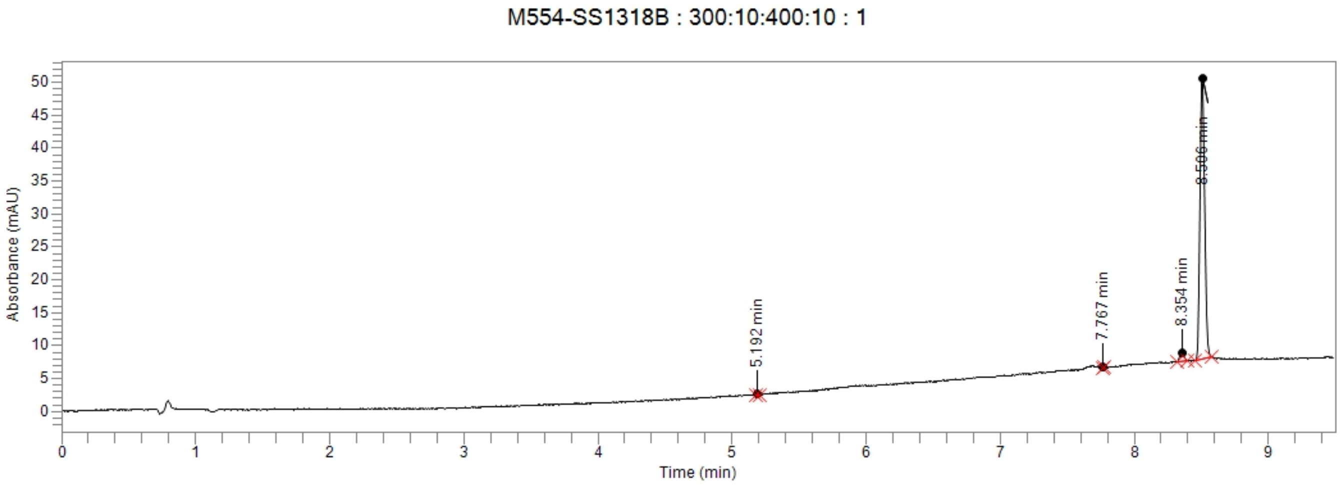

Channel Name **300:10:400:10**

Component Name	Time	Height	Area	Area %	Peak Purity	Wavelength Maximum
	5.192	163.4	194.8	0.18	1.12Pass	200
	7.767	117.1	39.6	0.04	1.07Pass	201
	8.354	1,233.1	2,732.3	2.56	1.13Pass	201
	8.506	42,642.5	103,841.1	97.22	1.09Pass	201
Total			106,807.7	100.00		

M554-SS1318B : 337:10:400:10 : 1

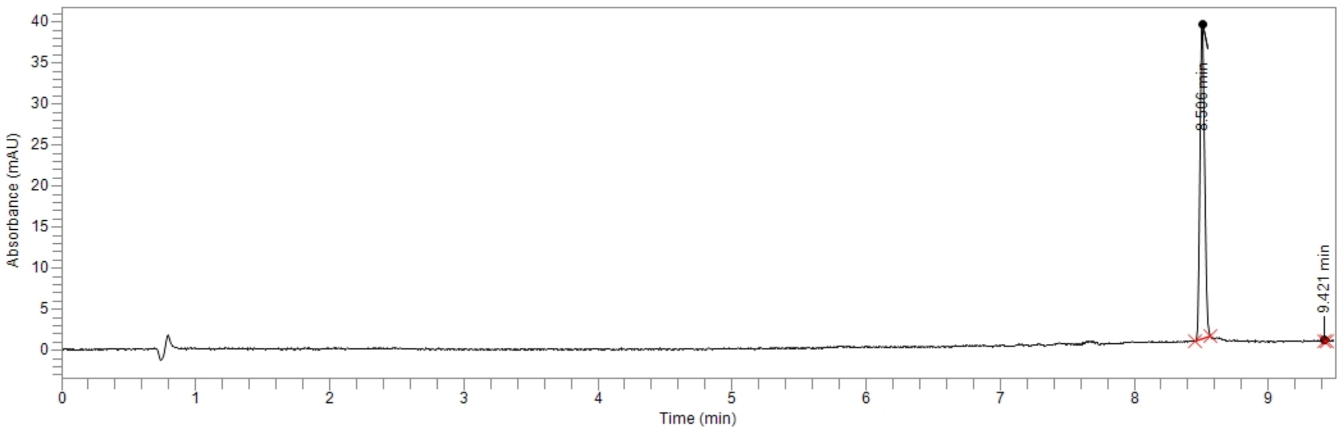

Channel Name **337:10:400:10**

Component Name	Time	Height	Area	Area %	Peak Purity	Wavelength Maximum
	8.506	38,348.8	92,898.7	99.92	1.09Pass	201
	9.421	136.1	73.8	0.08	1.14Pass	201
Total			92,972.5	100.00		

Report UHPLC

Acquisition Date/Time	7/15/2021 4:10:58 PM	Sample Name	M605-SS1684A
Acquisition Method	MS3	Injection Volume	2
Instrument Name	UHPLC		

M605-SS1684A : 254:10:400:10 : 1

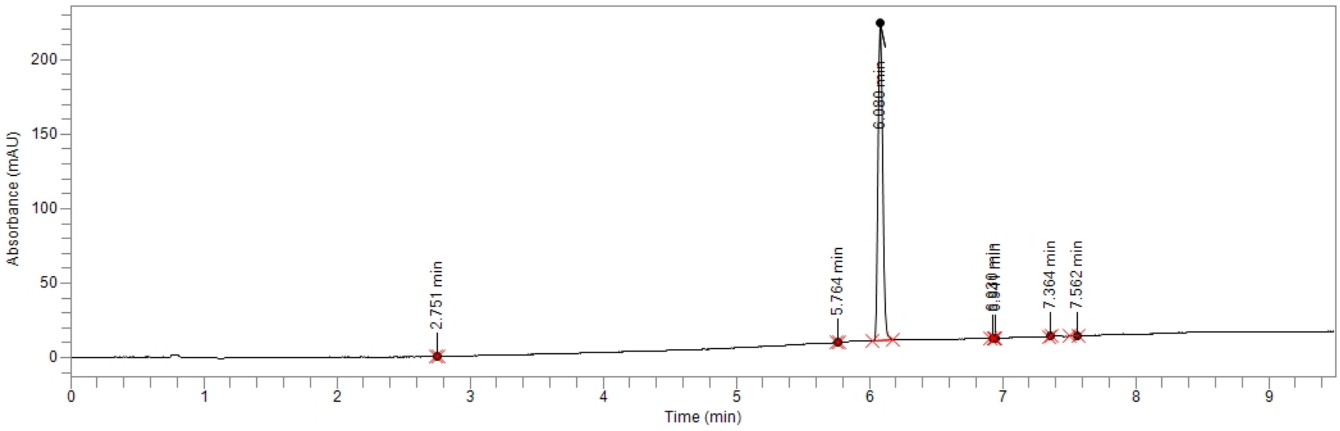

Channel Name 254:10:400:10

Component Name	Time	Height	Area	Area %	Peak Purity	Wavelength Maximum
	2.751	138.8	83.0	0.02	Not Enough Valid Points	198
	5.764	137.2	59.3	0.01	1.13Pass	200
	6.080	213,522.3	519,132.8	99.82	1.36Pass	201
	6.930	136.9	131.5	0.03	1.27Pass	200
	6.941	148.3	51.0	0.01	1.14Pass	200
	7.364	114.4	148.7	0.03	1.18Pass	201
	7.562	152.1	447.1	0.09	1.18Pass	201
Total			520,053.4	100.00		

M605-SS1684A : 230:10:400:10 : 1

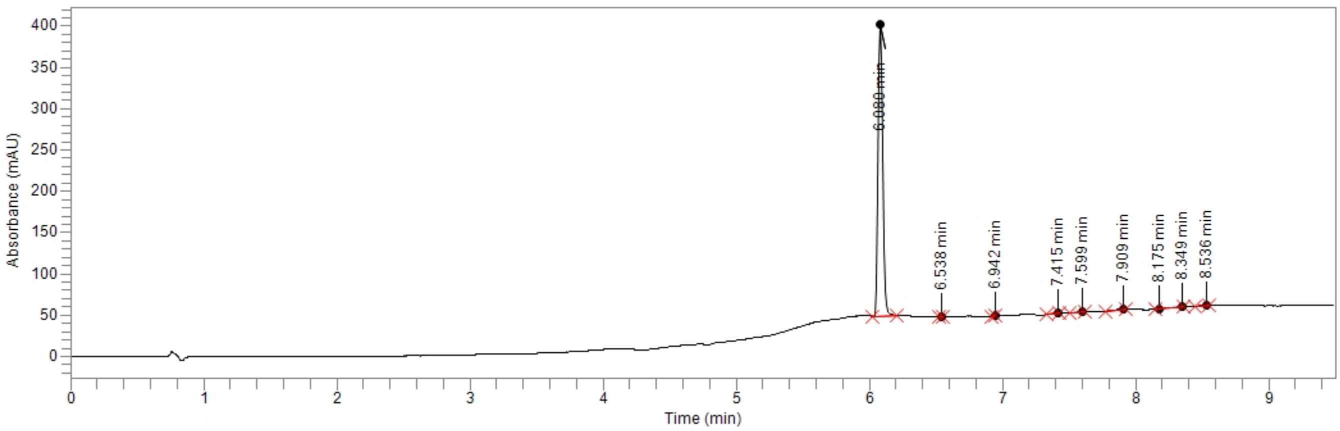

Channel Name **230:10:400:10**

Component Name	Time	Height	Area	Area %	Peak Purity	Wavelength Maximum
	6.080	353,740.3	862,557.2	98.70	1.36Pass	201
	6.538	162.7	90.0	0.01	1.15Pass	200
	6.942	166.1	210.2	0.02	1.26Pass	200
	7.415	310.4	1,956.9	0.22	1.15Pass	200
	7.599	259.9	1,526.9	0.17	1.18Pass	201
	7.909	410.7	3,181.5	0.36	1.15Pass	201
	8.175	150.1	218.0	0.02	1.11Pass	201
	8.349	126.8	2,760.2	0.32	1.06Pass	201
	8.536	321.5	1,388.2	0.16	1.16Pass	201
Total			873,889.1	100.00		

M605-SS1684A : 300:10:400:10 : 1

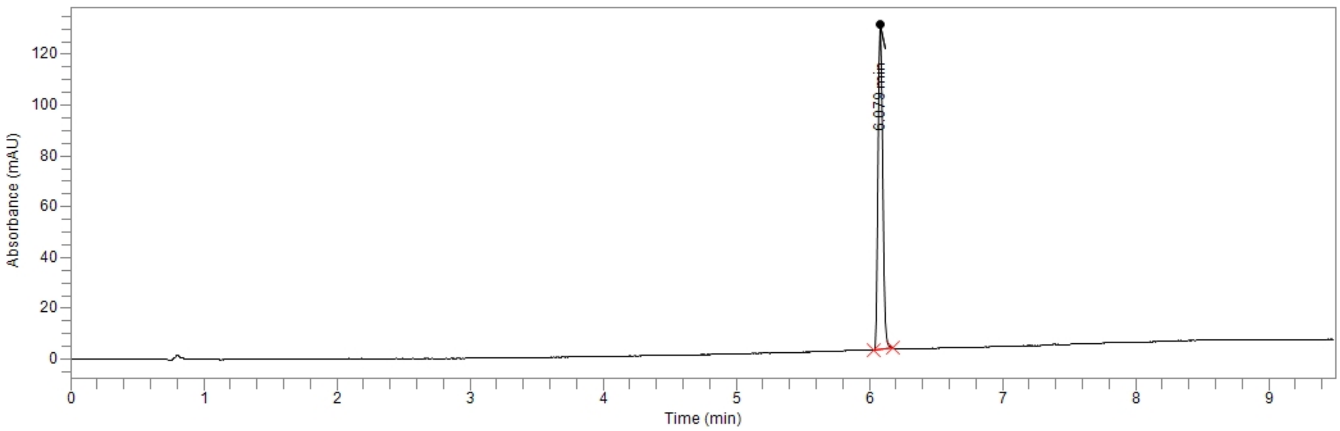

Channel Name **300:10:400:10**

Component Name	Time	Height	Area	Area %	Peak Purity	Wavelength Maximum
	6.079	128,198.8	311,660.5	100.00	1.10Pass	201
Total			311,660.5	100.00		

M605-SS1684A : 337:10:400:10 : 1

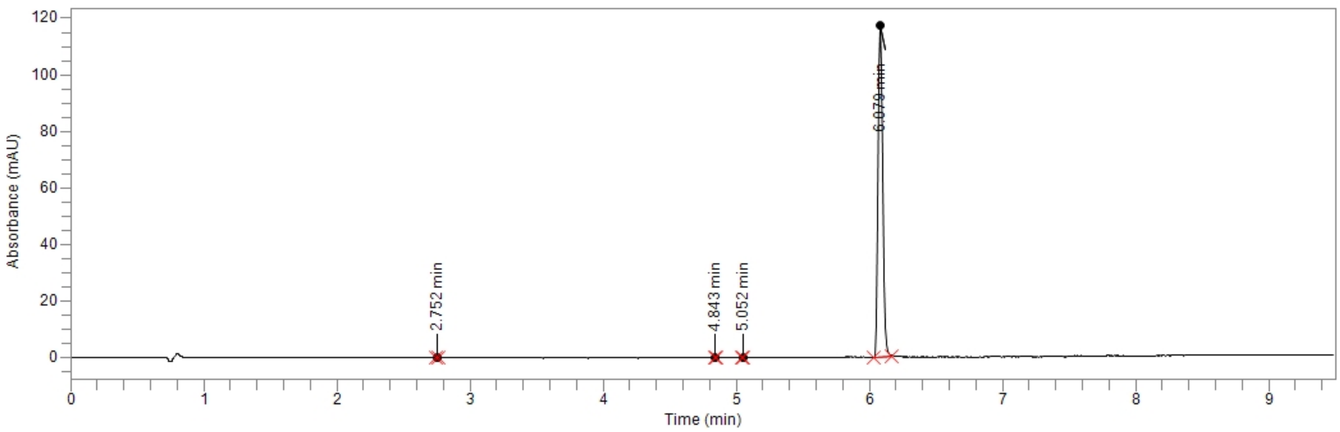

Channel Name **337:10:400:10**

Component Name	Time	Height	Area	Area %	Peak Purity	Wavelength Maximum
	2.752	146.3	78.3	0.03	Not Enough Valid Points	198
	4.843	172.3	62.2	0.02	1.27Pass	200
	5.052	230.7	84.5	0.03	1.13Pass	200
	6.079	117,215.3	285,605.1	99.92	1.13Pass	201
Total			285,830.1	100.00		

Report UHPLC

Acquisition Date/Time	7/15/2021 4:23:51 PM	Sample Name	M612-SS1774A
Acquisition Method	MS3	Injection Volume	2
Instrument Name	UHPLC		

M612-SS1774A : 254:10:400:10 : 1

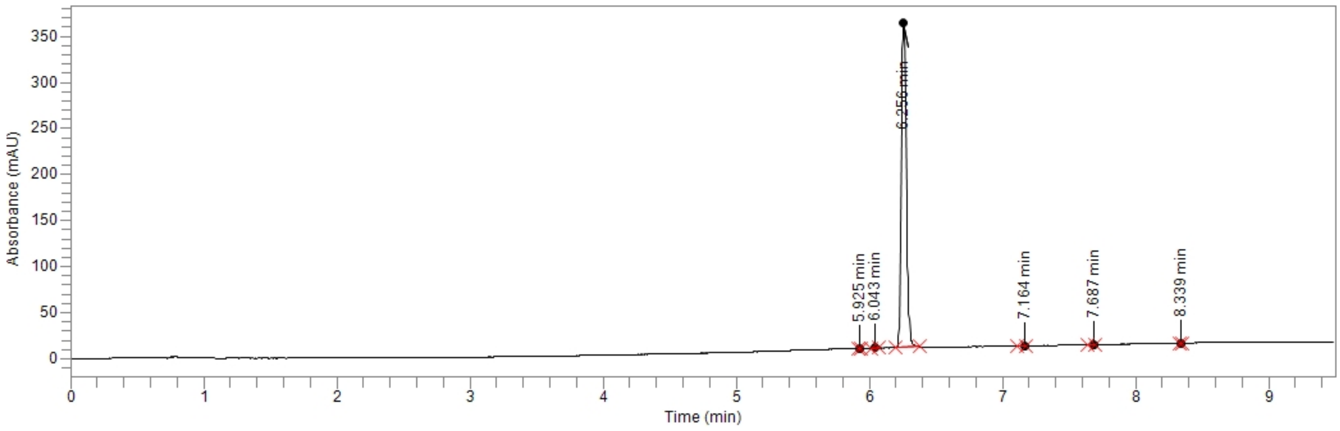

Channel Name **254:10:400:10**

Component Name	Time	Height	Area	Area %	Peak Purity	Wavelength Maximum
	5.925	123.3	45.9	0.00	1.07Pass	200
	6.043	551.5	1,124.4	0.12	1.19Pass	200
	6.256	353,467.4	930,434.0	99.74	1.34Pass	202
	7.164	181.8	577.9	0.06	1.11Pass	201
	7.687	164.4	583.3	0.06	1.18Pass	201
	8.339	119.6	96.3	0.01	1.07Pass	201
Total			932,861.9	100.00		

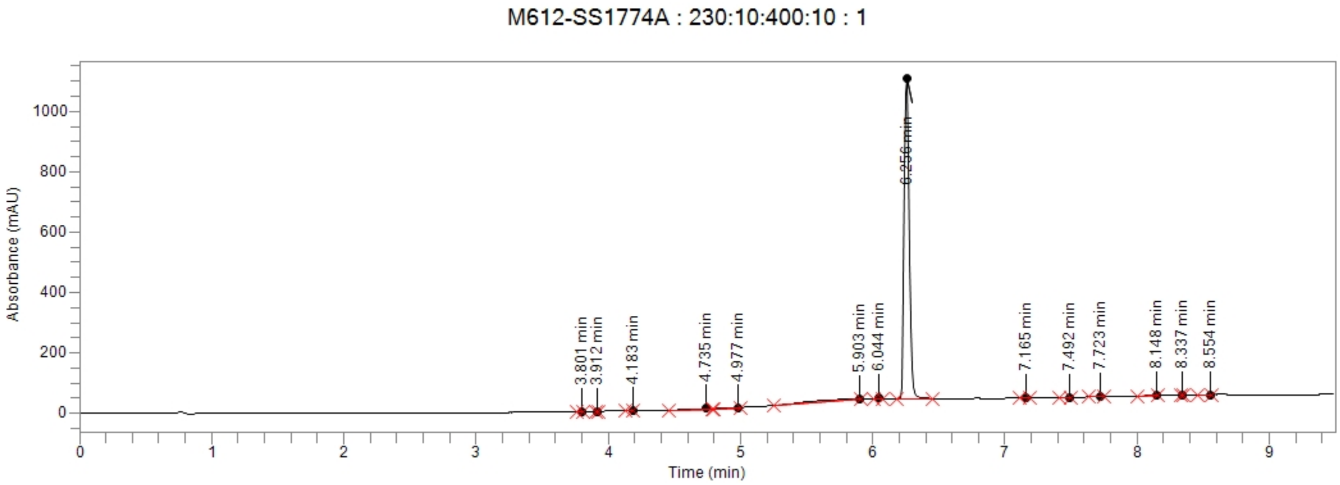

Channel Name **230:10:400:10**

Component Name	Time	Height	Area	Area %	Peak Purity	Wavelength Maximum
	3.801	138.6	260.9	0.01	Not Enough Valid Points	199
	3.912	178.4	109.2	0.00	Not Enough Valid Points	199
	4.183	165.1	272.7	0.01	Not Enough Valid Points	199
	4.735	1,994.7	26,182.9	0.88	Not Enough Valid Points	199
	4.977	211.9	9,025.5	0.30	Not Enough Valid Points	200
	5.903	318.2	86,634.6	2.91	1.69Fail	200
	6.044	1,007.6	2,296.6	0.08	1.07Pass	200
	6.256	1,062,979.6	2,835,351.0	95.40	1.34Pass	202
	7.165	511.5	1,578.1	0.05	1.13Pass	201
	7.492	325.2	2,109.4	0.07	1.14Pass	201
	7.723	539.6	3,137.0	0.11	1.14Pass	201
	8.148	222.9	3,079.1	0.10	1.11Pass	201
	8.337	160.5	116.4	0.00	1.07Pass	201
	8.554	237.5	1,947.5	0.07	1.06Pass	201
Total			2,972,100.9	100.00		

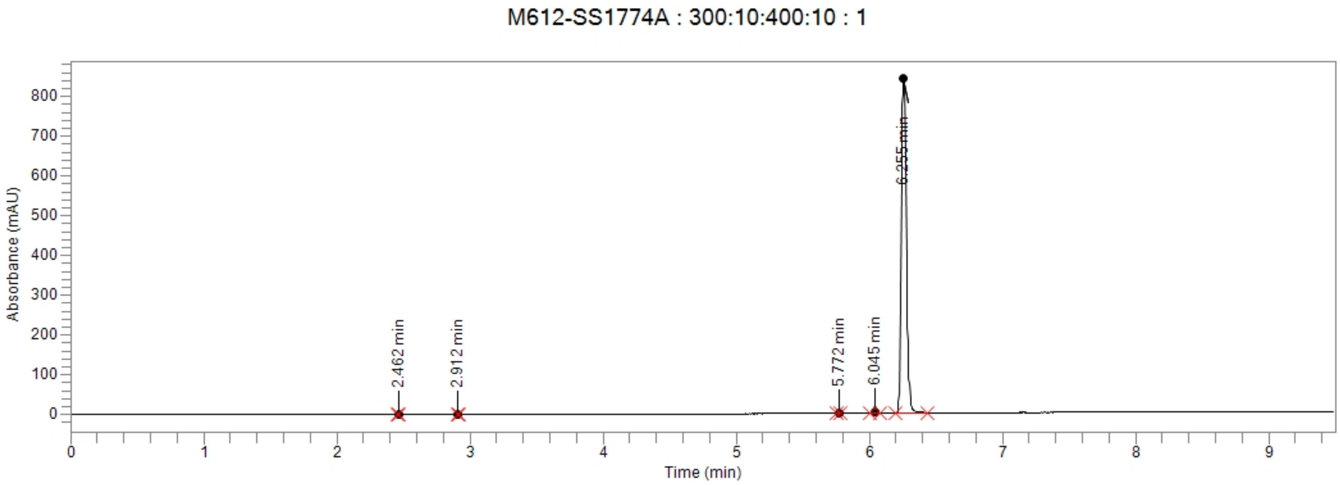

Channel Name **300:10:400:10**

Component Name	Time	Height	Area	Area %	Peak Purity	Wavelength Maximum
	2.462	159.4	55.8	0.00	Not Enough Valid Points	199
	2.912	165.7	62.7	0.00	Not Enough Valid Points	199
	5.772	144.0	151.2	0.01	1.16Pass	200
	6.045	1,516.8	3,415.5	0.15	1.20Pass	200
	6.255	843,923.9	2,235,436.8	99.84	1.34Pass	202
Total			2,239,122.0	100.00		

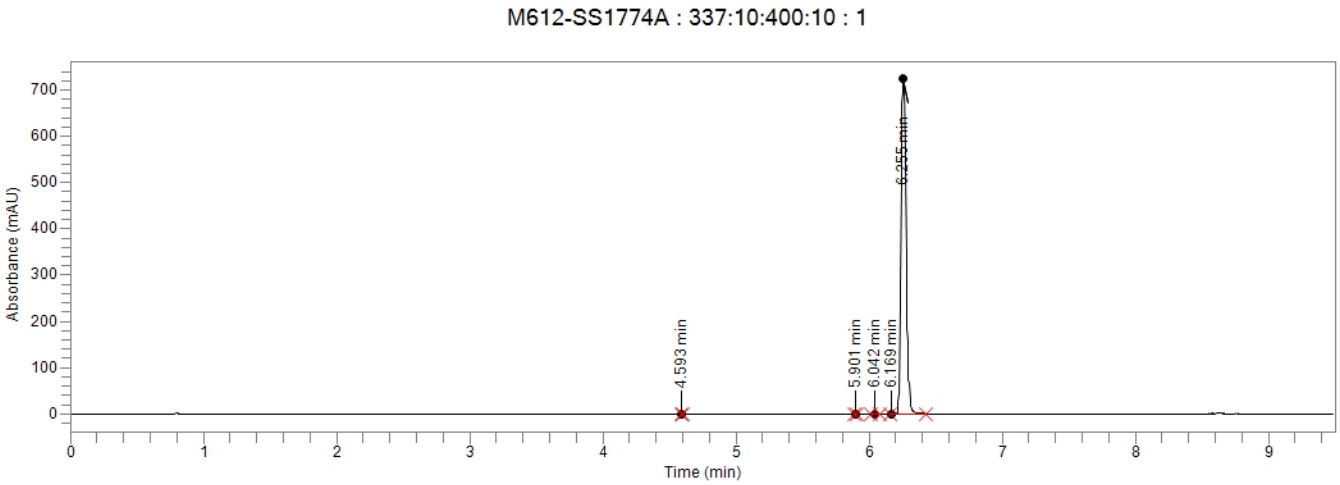

Channel Name **337:10:400:10**

Component Name	Time	Height	Area	Area %	Peak Purity	Wavelength Maximum
	4.593	120.3	40.3	0.00	Not Enough Valid Points	200
	5.901	170.6	152.5	0.01	1.11Pass	200
	6.042	538.5	1,092.9	0.06	1.31Pass	200
	6.169	208.1	186.8	0.01	1.09Pass	200
	6.255	726,275.4	1,938,641.7	99.92	1.34Pass	202
Total			1,940,114.3	100.00		

Report UHPLC

Acquisition Date/Time	7/15/2021 4:36:50 PM	Sample Name	M623-SS2008A
Acquisition Method	MS3	Injection Volume	2
Instrument Name	UHPLC		

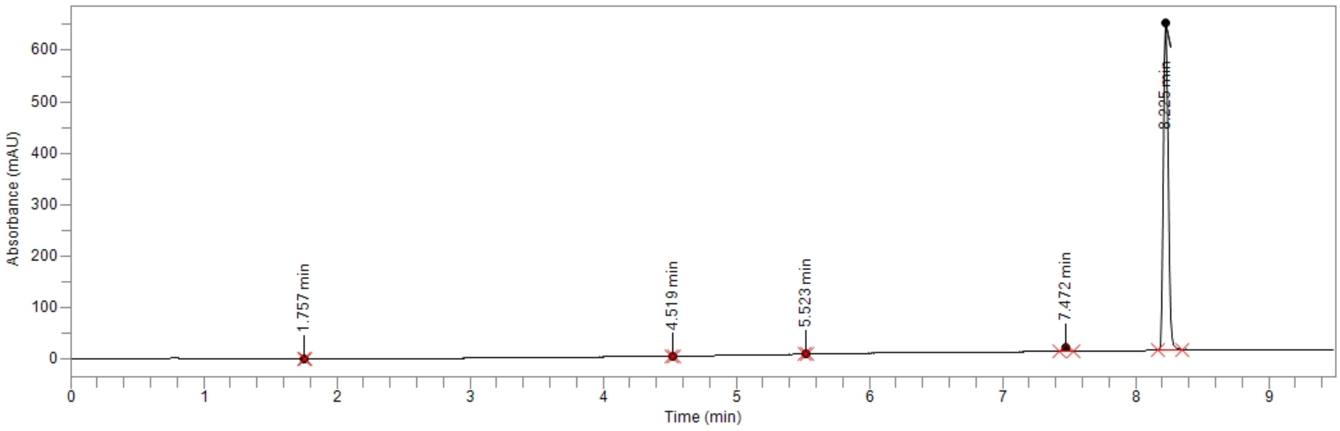

Channel Name **254:10:400:10**

Component Name	Time	Height	Area	Area %	Peak Purity	Wavelength Maximum
	1.757	148.0	50.2	0.00	2.64Fail	196
	4.519	144.5	71.5	0.00	Not Enough Valid Points	200
	5.523	91.9	65.4	0.00	1.31Pass	200
	7.472	6,724.7	17,184.1	1.05	1.28Pass	201
	8.225	637,412.5	1,615,976.3	98.94	1.25Pass	223
Total			1,633,347.5	100.00		

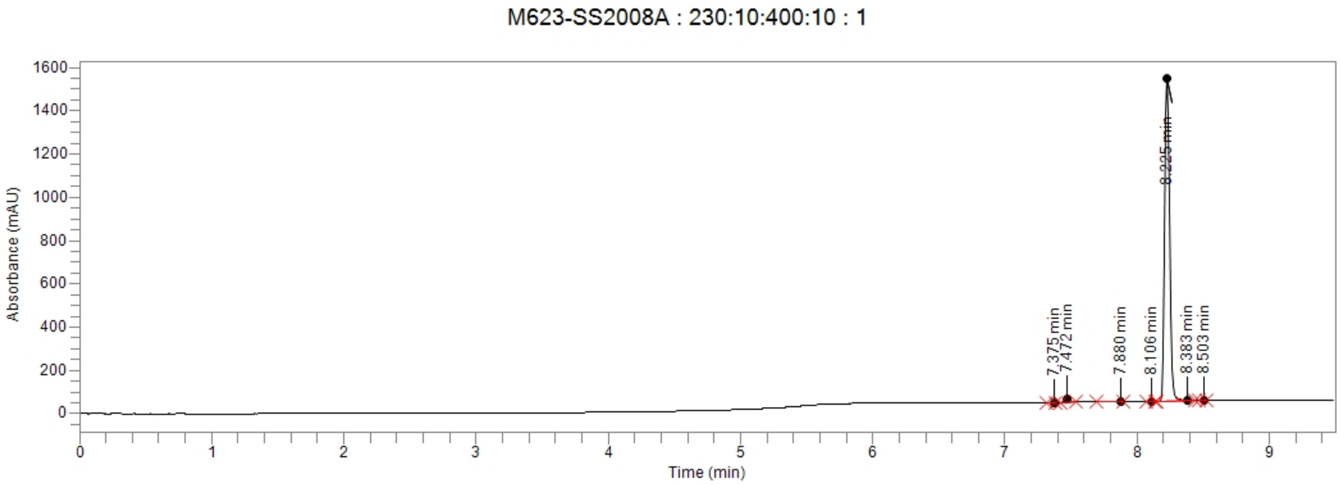

Channel Name **230:10:400:10**

Component Name	Time	Height	Area	Area %	Peak Purity	Wavelength Maximum
	7.375	251.0	975.2	0.02	1.27Pass	201
	7.472	14,270.7	36,746.0	0.94	1.28Pass	201
	7.880	131.6	4,654.4	0.12	1.21Pass	201
	8.106	576.3	1,289.2	0.03	1.12Pass	201
	8.225	1,493,775.0	3,864,902.7	98.46	1.19Pass	223
	8.383	3,228.8	16,352.2	0.42	1.14Pass	201
	8.503	209.7	395.1	0.01	1.06Pass	201
Total			3,925,314.8	100.00		

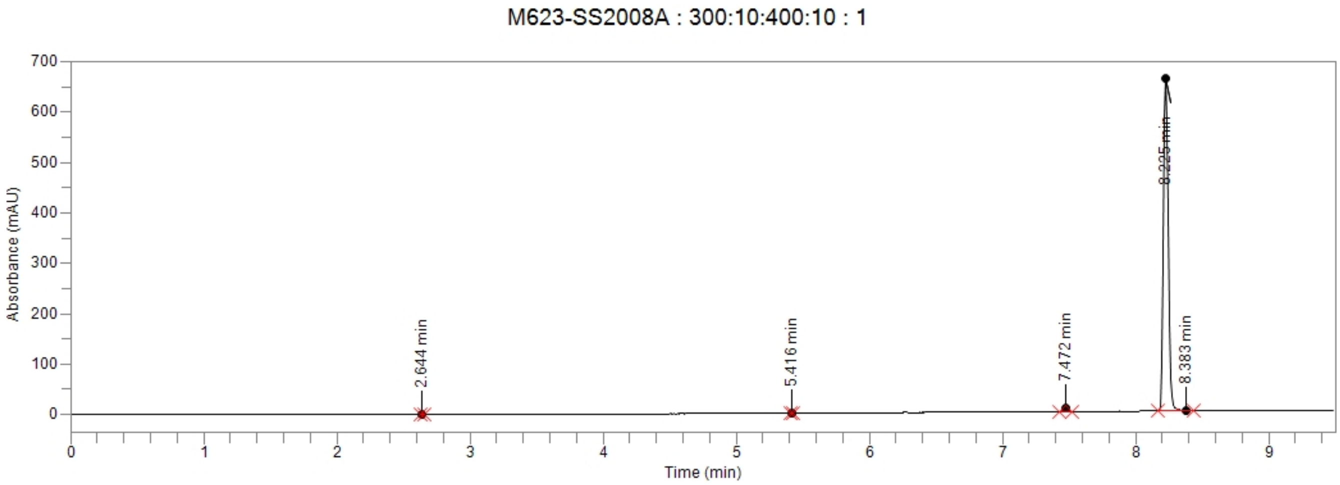

Channel Name **300:10:400:10**

Component Name	Time	Height	Area	Area %	Peak Purity	Wavelength Maximum
	2.644	126.9	135.6	0.01	Not Enough Valid Points	199
	5.416	145.1	61.7	0.00	1.25Pass	200
	7.472	5,972.1	14,969.2	0.88	1.28Pass	201
	8.225	660,542.0	1,677,950.4	98.80	1.19Pass	223
	8.383	1,270.5	5,171.4	0.30	1.18Pass	201
Total			1,698,288.4	100.00		

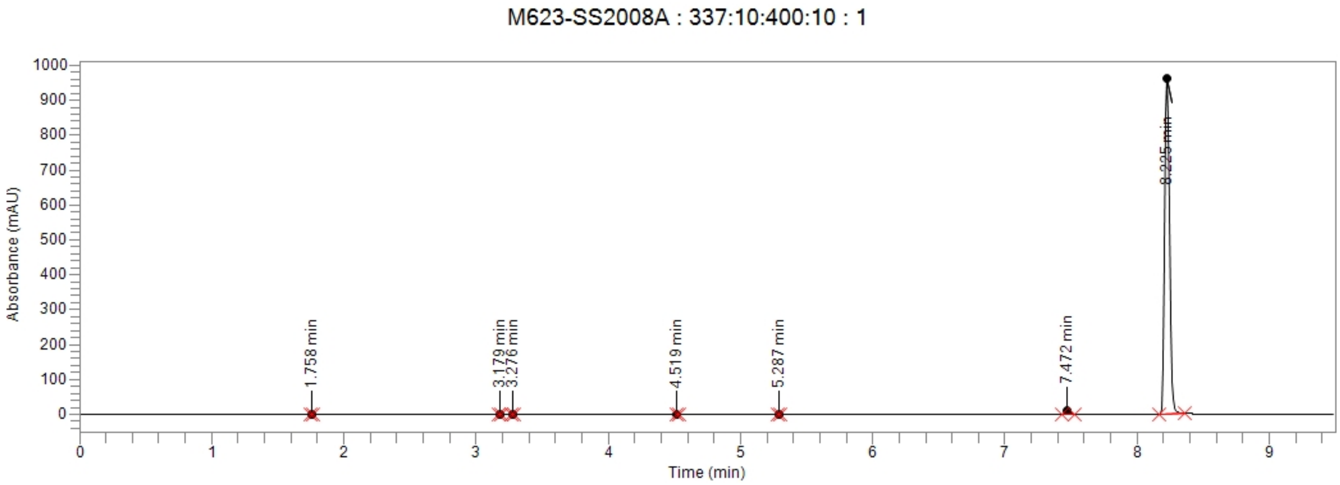

Channel Name **337:10:400:10**

Component Name	Time	Height	Area	Area %	Peak Purity	Wavelength Maximum
	1.758	193.0	92.8	0.00	4.16Fail	195
	3.179	187.5	110.3	0.00	Not Enough Valid Points	199
	3.276	161.8	54.7	0.00	Not Enough Valid Points	199
	4.519	178.3	130.8	0.01	Not Enough Valid Points	200
	5.287	176.0	71.1	0.00	1.14Pass	200
	7.472	9,303.7	23,928.1	0.96	1.28Pass	201
	8.225	962,737.0	2,480,497.5	99.03	1.19Pass	223
Total			2,504,885.1	100.00		

Report UHPLC

Acquisition Date/Time	7/15/2021 4:49:50 PM	Sample Name	M626-SS2174A
Acquisition Method	MS3	Injection Volume	2
Instrument Name	UHPLC		

M626-SS2174A : 254:10:400:10 : 1

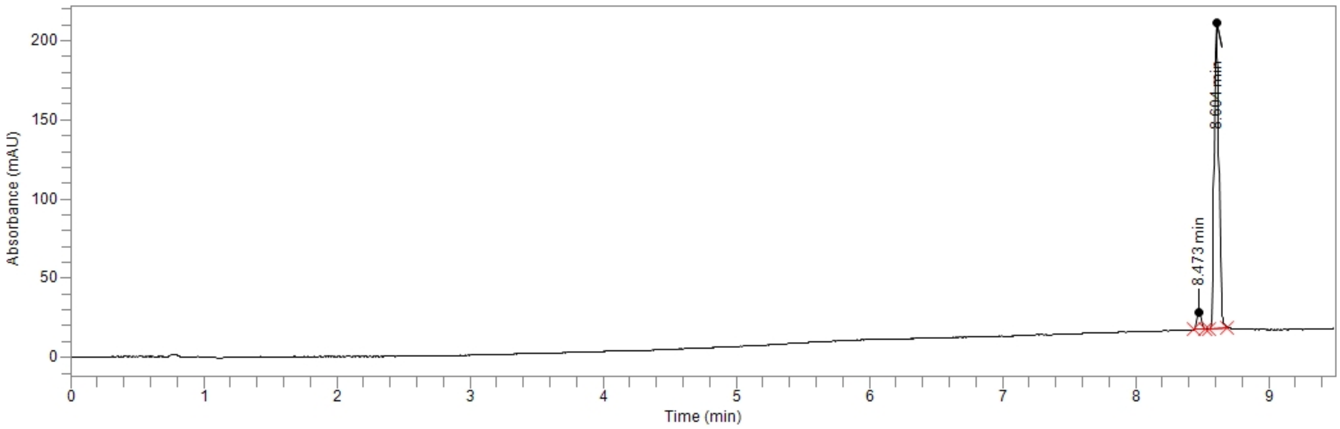

Channel Name **254:10:400:10**

Component Name	Time	Height	Area	Area %	Peak Purity	Wavelength Maximum
	8.473	10,621.1	25,331.2	4.98	1.08Pass	201
	8.604	193,359.2	483,714.0	95.02	1.14Pass	202
Total			509,045.2	100.00		

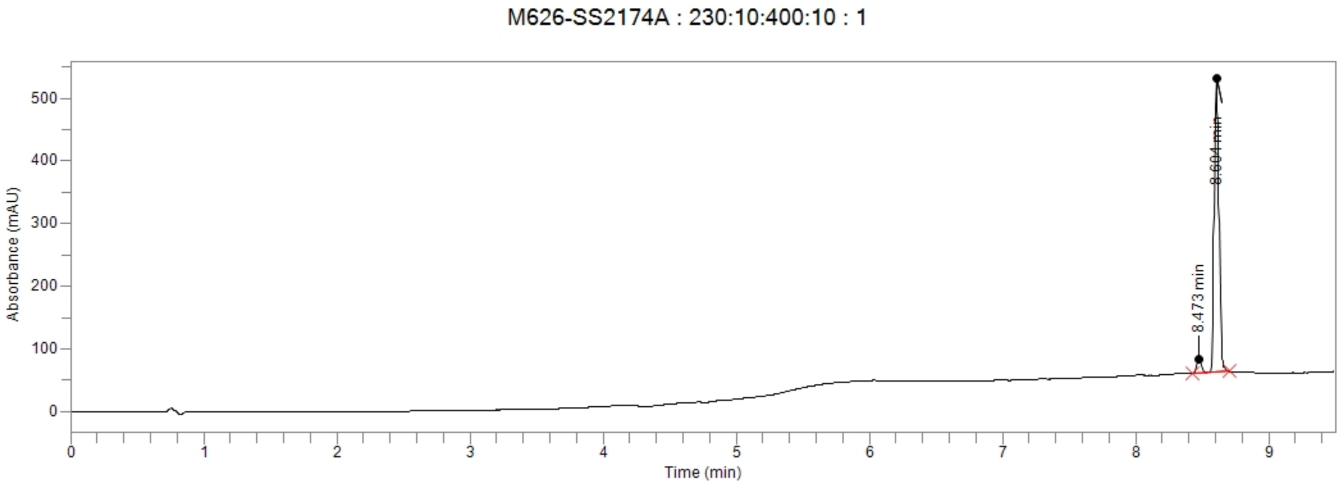

Channel Name **230:10:400:10**

Component Name	Time	Height	Area	Area %	Peak Purity	Wavelength Maximum
	8.473	22,784.9	56,885.6	4.58	1.10Pass	201
	8.604	469,973.6	1,184,979.4	95.42	1.14Pass	202
Total			1,241,865.0	100.00		

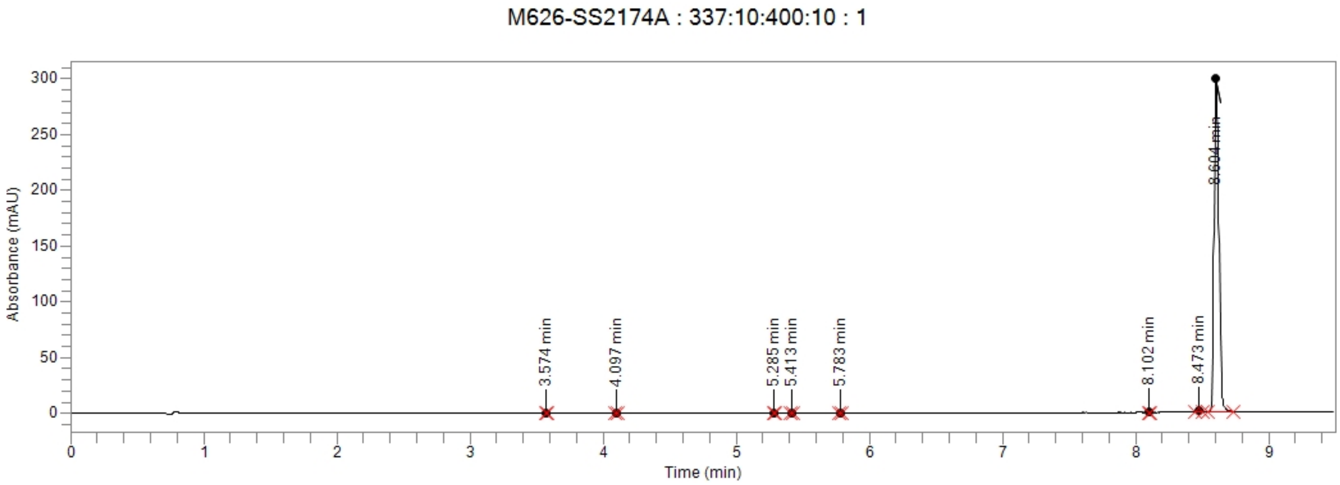

Channel Name **337:10:400:10**

Component Name	Time	Height	Area	Area %	Peak Purity	Wavelength Maximum
	3.574	201.8	73.1	0.01	Not Enough Valid Points	199
	4.097	189.4	97.1	0.01	Not Enough Valid Points	199
	5.285	166.6	73.6	0.01	1.15Pass	200
	5.413	176.6	134.7	0.02	1.17Pass	200
	5.783	160.2	55.7	0.01	1.21Pass	200
	8.102	213.4	91.5	0.01	1.20Pass	201
	8.473	735.5	1,365.3	0.18	1.22Pass	201
	8.604	299,650.4	757,739.1	99.75	1.14Pass	202
Total			759,630.1	100.00		

Report UHPLC

Acquisition Date/Time	7/15/2021 5:03:06 PM	Sample Name	M627-SS2184B
Acquisition Method	MS3	Injection Volume	2
Instrument Name	UHPLC		

M627-SS2184B : 254:10:400:10 : 1

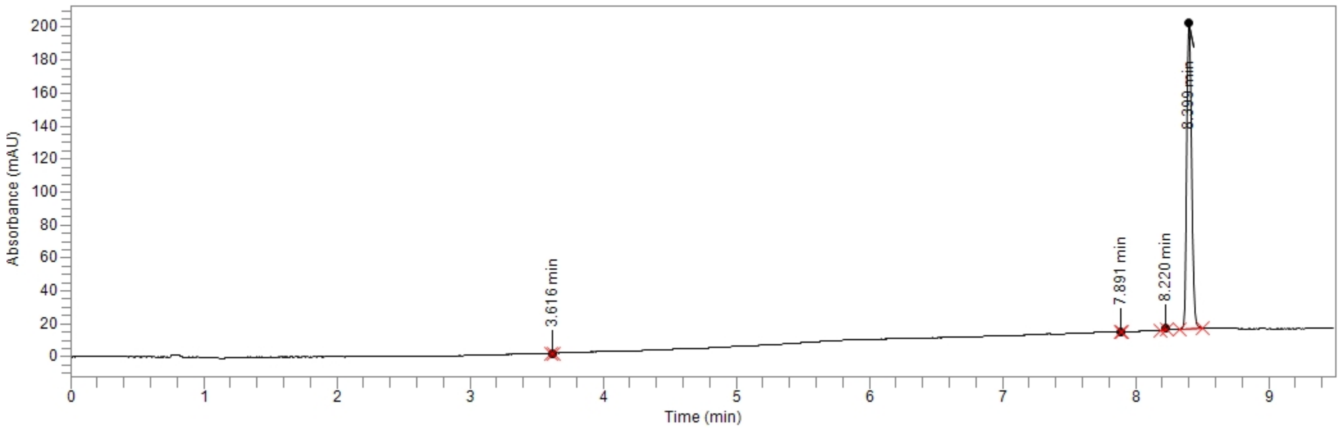

Channel Name **254:10:400:10**

Component Name	Time	Height	Area	Area %	Peak Purity	Wavelength Maximum
	3.616	130.6	54.1	0.01	Not Enough Valid Points	199
	7.891	189.4	69.8	0.01	1.22Pass	201
	8.220	292.0	416.8	0.09	1.10Pass	201
	8.399	186,021.9	474,249.3	99.89	1.10Pass	202
Total			474,790.0	100.00		

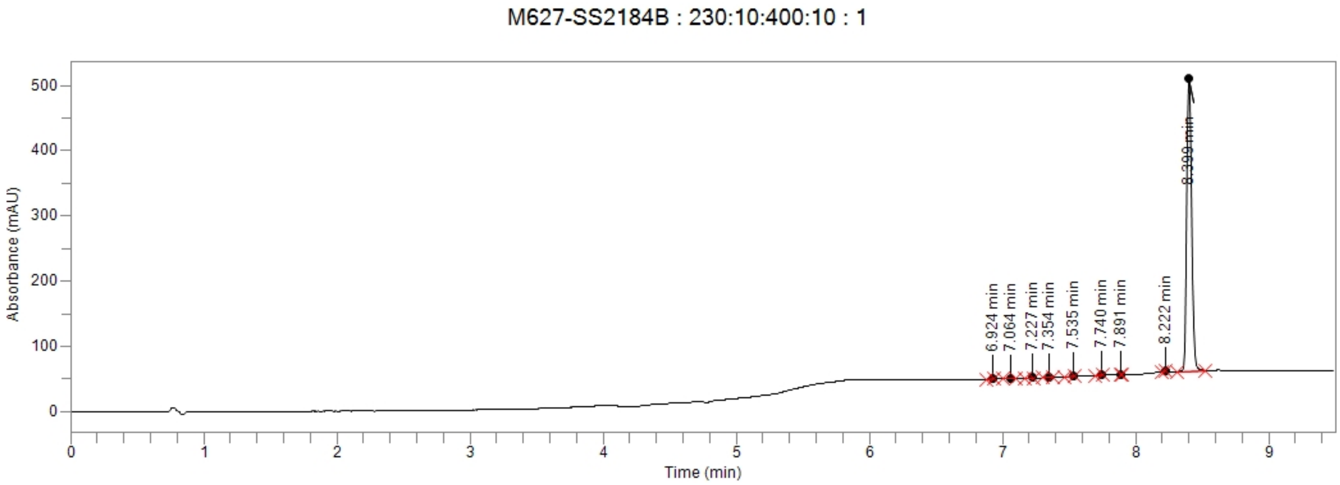

Channel Name **230:10:400:10**

Component Name	Time	Height	Area	Area %	Peak Purity	Wavelength Maximum
	6.924	298.1	743.3	0.06	1.11Pass	200
	7.064	716.7	2,397.6	0.21	1.22Pass	201
	7.227	344.6	1,285.5	0.11	1.10Pass	201
	7.354	440.5	1,255.7	0.11	1.11Pass	201
	7.535	256.1	1,456.1	0.13	1.08Pass	201
	7.740	194.4	667.8	0.06	1.10Pass	201
	7.891	163.4	59.5	0.01	1.22Pass	201
	8.222	440.4	578.6	0.05	1.28Pass	201
	8.399	450,231.6	1,148,488.1	99.27	1.10Pass	202
Total			1,156,932.3	100.00		

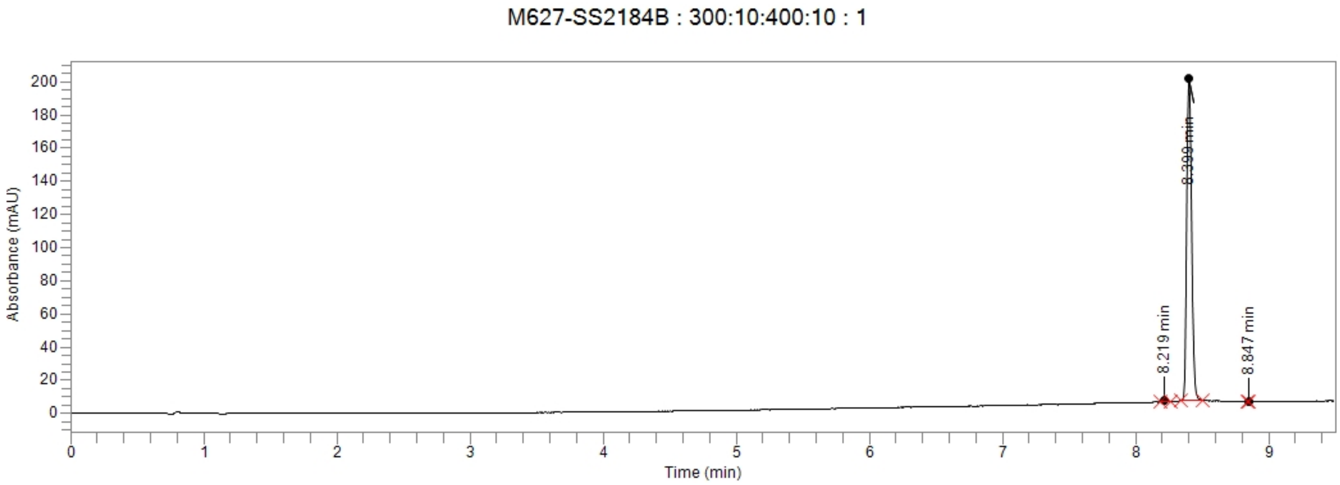

Channel Name **300:10:400:10**

Component Name	Time	Height	Area	Area %	Peak Purity	Wavelength Maximum
	8.219	1,295.2	3,077.0	0.62	1.12Pass	201
	8.399	194,691.3	495,607.0	99.37	1.10Pass	202
	8.847	175.6	50.1	0.01	1.13Pass	201
Total			498,734.1	100.00		

M627-SS2184B : 337:10:400:10 : 1

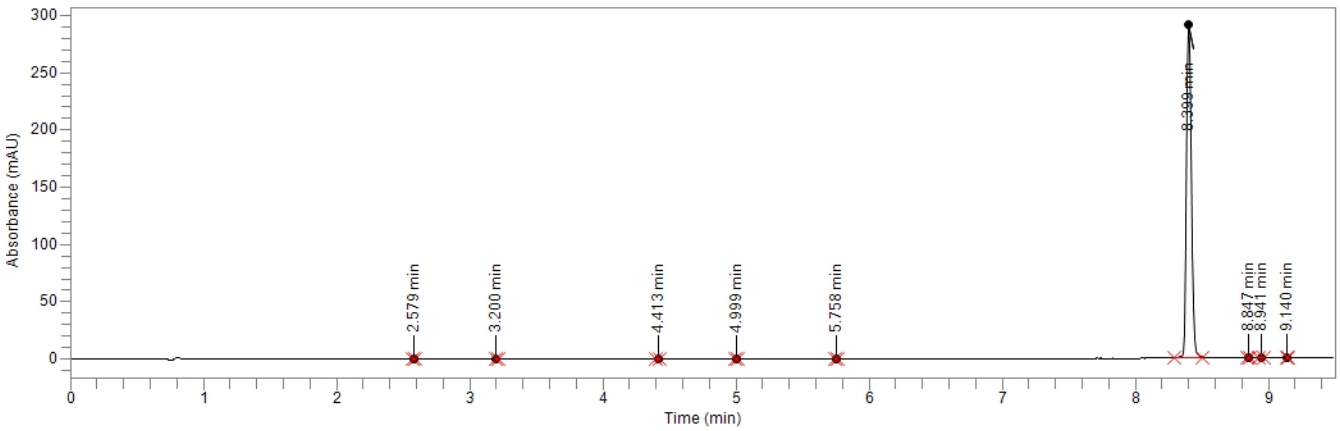

Channel Name **337:10:400:10**

Component Name	Time	Height	Area	Area %	Peak Purity	Wavelength Maximum
	2.579	216.4	133.5	0.02	3.62Fail	199
	3.200	163.7	111.6	0.01	Not Enough Valid Points	199
	4.413	131.3	147.4	0.02	Not Enough Valid Points	199
	4.999	171.7	119.0	0.02	1.19Pass	200
	5.758	203.6	188.2	0.03	1.13Pass	200
	8.399	291,470.3	746,786.4	99.84	1.10Pass	202
	8.847	198.1	86.9	0.01	1.18Pass	201
	8.941	249.7	382.8	0.05	1.11Pass	201
	9.140	117.2	42.0	0.01	1.12Pass	201
Total			747,997.9	100.00		

Report UHPLC

Acquisition Date/Time	7/15/2021 5:16:02 PM	Sample Name	M628-SS2180B
Acquisition Method	MS3	Injection Volume	2
Instrument Name	UHPLC		

M628-SS2180B : 254:10:400:10 : 1

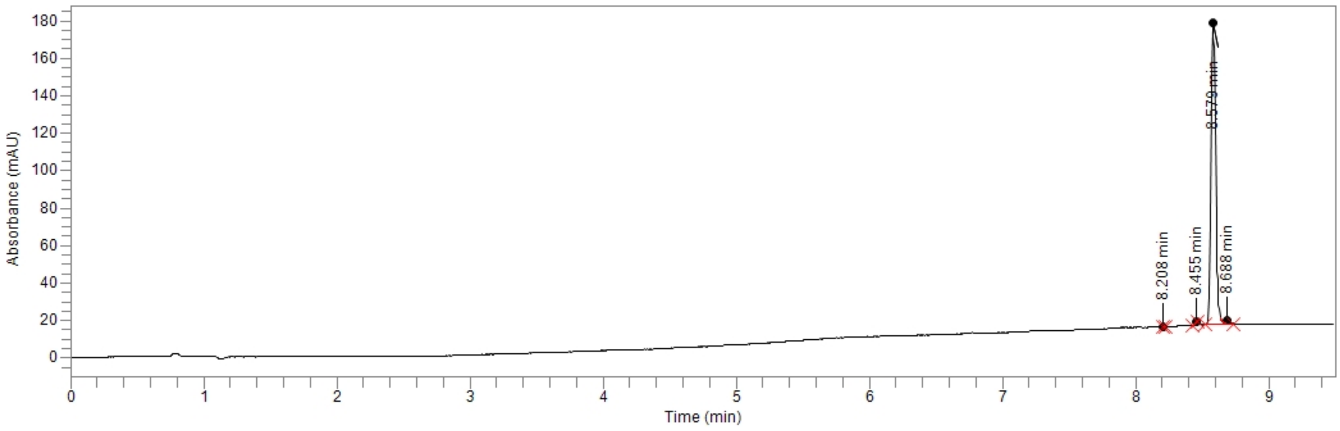

Channel Name 254:10:400:10

Component Name	Time	Height	Area	Area %	Peak Purity	Wavelength Maximum
	8.208	122.8	66.3	0.02	1.21Pass	201
	8.455	387.8	523.2	0.13	1.22Pass	201
	8.579	161,421.4	409,738.6	98.41	1.19Pass	202
	8.688	2,099.2	6,045.2	1.45	1.12Pass	201
Total			416,373.3	100.00		

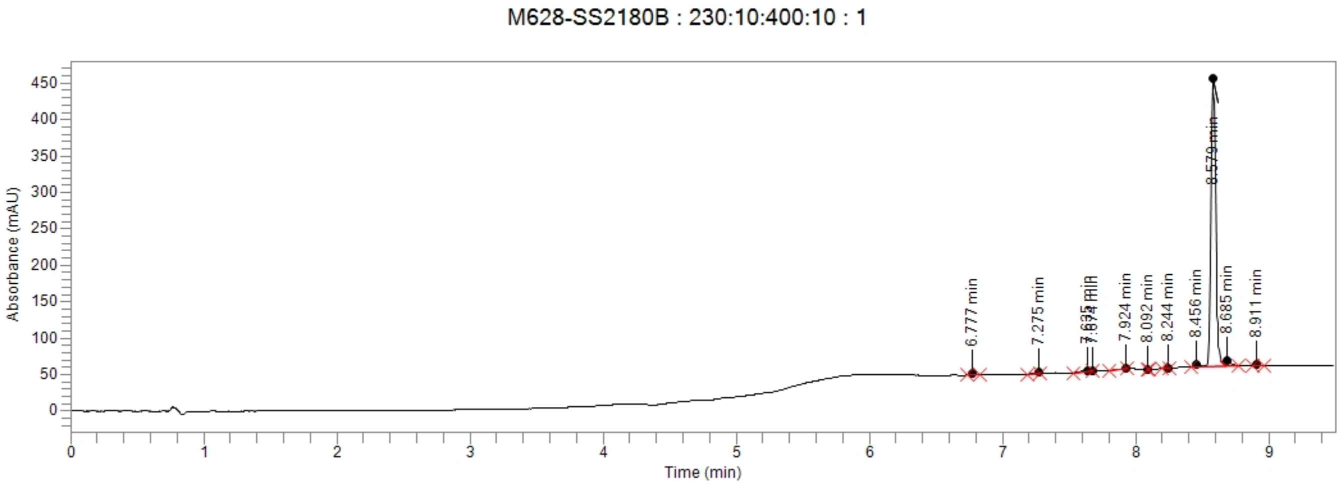

Channel Name **230:10:400:10**

Component Name	Time	Height	Area	Area %	Peak Purity	Wavelength Maximum
	6.777	3,050.2	7,696.0	0.72	1.29Pass	200
	7.275	322.4	2,348.2	0.22	1.08Pass	201
	7.635	538.1	3,777.8	0.36	1.07Pass	201
	7.674	218.5	734.1	0.07	1.15Pass	201
	7.924	243.1	2,070.0	0.19	1.11Pass	201
	8.092	165.5	69.4	0.01	1.11Pass	201
	8.244	358.4	837.5	0.08	1.12Pass	201
	8.456	3,466.5	8,508.7	0.80	1.20Pass	201
	8.579	395,576.7	1,011,161.0	95.07	1.19Pass	202
	8.685	7,347.2	23,449.2	2.20	1.12Pass	201
	8.911	1,180.5	2,953.0	0.28	1.09Pass	201
Total			1,063,605.1	100.00		

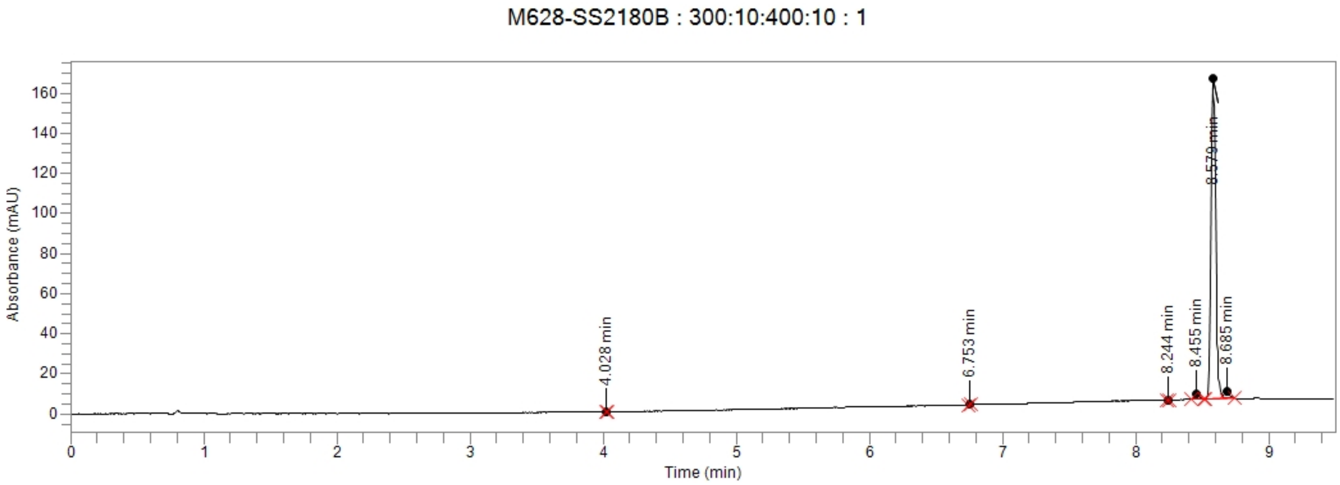

Channel Name **300:10:400:10**

Component Name	Time	Height	Area	Area %	Peak Purity	Wavelength Maximum
	4.028	167.4	68.1	0.02	Not Enough Valid Points	199
	6.753	161.8	56.5	0.01	1.12Pass	200
	8.244	192.7	78.6	0.02	1.15Pass	201
	8.455	2,490.0	5,862.1	1.39	1.16Pass	201
	8.579	159,890.4	405,478.5	96.23	1.19Pass	202
	8.685	3,576.6	9,806.7	2.33	1.13Pass	201
Total			421,350.5	100.00		

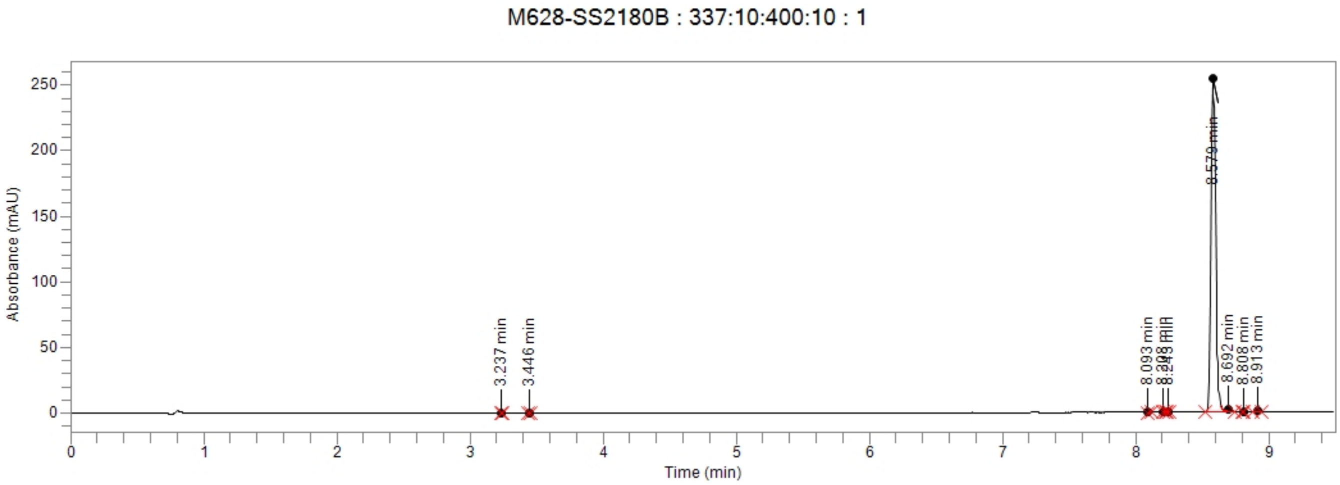

Channel Name **337:10:400:10**

Component Name	Time	Height	Area	Area %	Peak Purity	Wavelength Maximum
	3.237	130.3	43.3	0.01	Not Enough Valid Points	199
	3.446	163.9	84.7	0.01	Not Enough Valid Points	199
	8.093	244.5	221.7	0.03	1.08Pass	201
	8.208	218.2	132.5	0.02	1.08Pass	201
	8.243	179.7	71.0	0.01	1.10Pass	201
	8.579	254,278.9	646,010.2	98.71	1.19Pass	202
	8.692	1,913.5	6,004.5	0.92	1.14Pass	201
	8.808	178.7	56.4	0.01	1.11Pass	201
	8.913	864.5	1,827.1	0.28	1.09Pass	201
Total			654,451.4	100.00		

Report UHPLC

Acquisition Date/Time	7/15/2021 5:28:58 PM	Sample Name	M629-SS2182B
Acquisition Method	MS3	Injection Volume	2
Instrument Name	UHPLC		

M629-SS2182B : 254:10:400:10 : 1

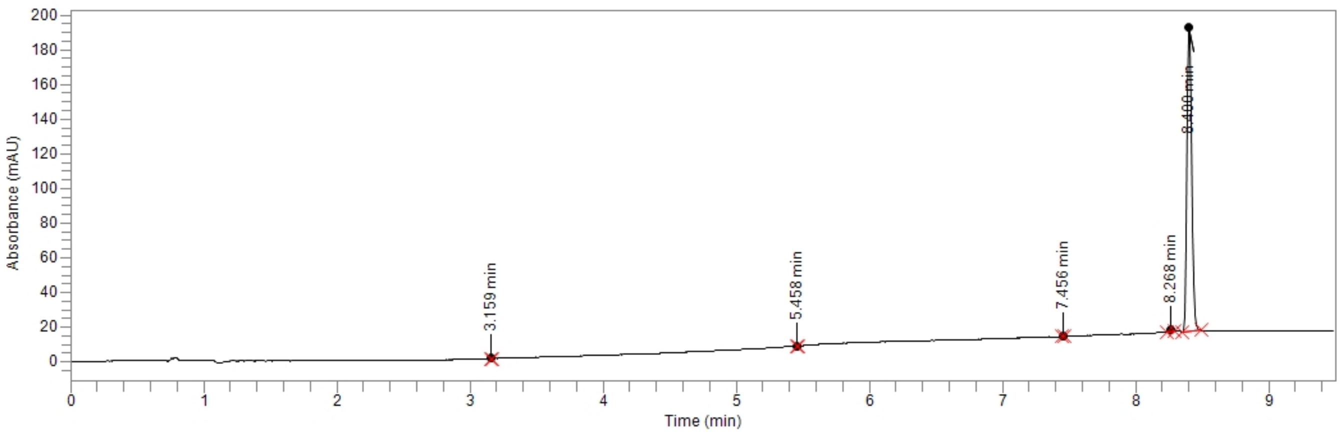

Channel Name **254:10:400:10**

Component Name	Time	Height	Area	Area %	Peak Purity	Wavelength Maximum
	3.159	202.8	70.4	0.02	Not Enough Valid Points	199
	5.458	158.2	49.6	0.01	1.21Pass	200
	7.456	184.7	89.4	0.02	1.21Pass	201
	8.268	935.9	1,820.8	0.42	1.17Pass	201
	8.400	175,530.7	436,207.1	99.54	1.13Pass	202
Total			438,237.2	100.00		

M629-SS2182B : 230:10:400:10 : 1

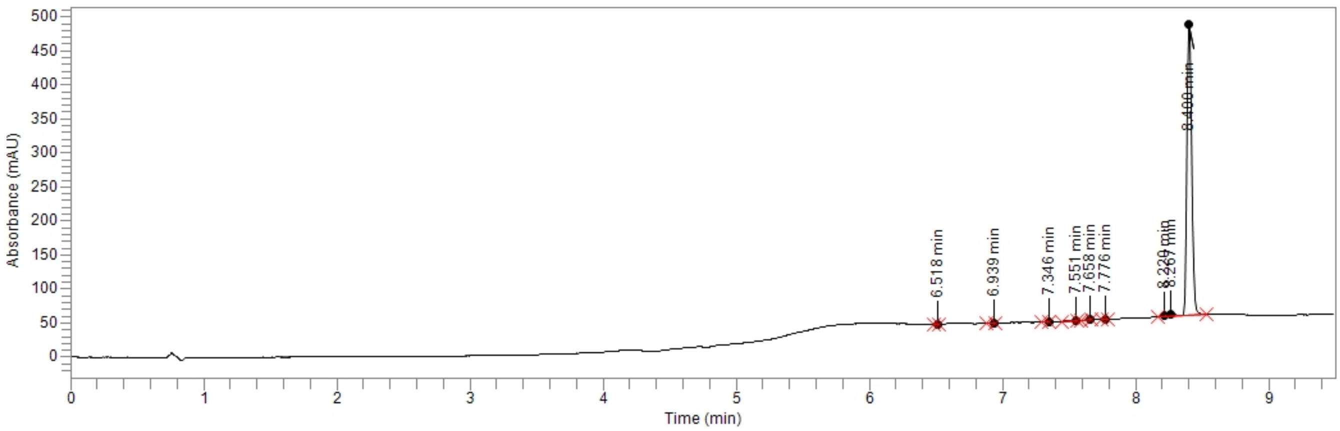

Channel Name **230:10:400:10**

Component Name	Time	Height	Area	Area %	Peak Purity	Wavelength Maximum
	6.518	173.5	232.1	0.02	1.22Pass	200
	6.939	320.5	1,174.5	0.11	1.18Pass	200
	7.346	159.7	733.6	0.07	1.14Pass	201
	7.551	155.7	1,701.6	0.16	1.16Pass	201
	7.658	124.8	650.6	0.06	1.19Pass	201
	7.776	407.0	658.5	0.06	1.15Pass	201
	8.220	1,095.9	2,336.4	0.21	1.07Pass	201
	8.267	3,448.8	12,982.9	1.19	1.05Pass	201
	8.400	428,155.0	1,069,091.9	98.12	1.13Pass	202
Total			1,089,562.1	100.00		

M629-SS2182B : 300:10:400:10 : 1

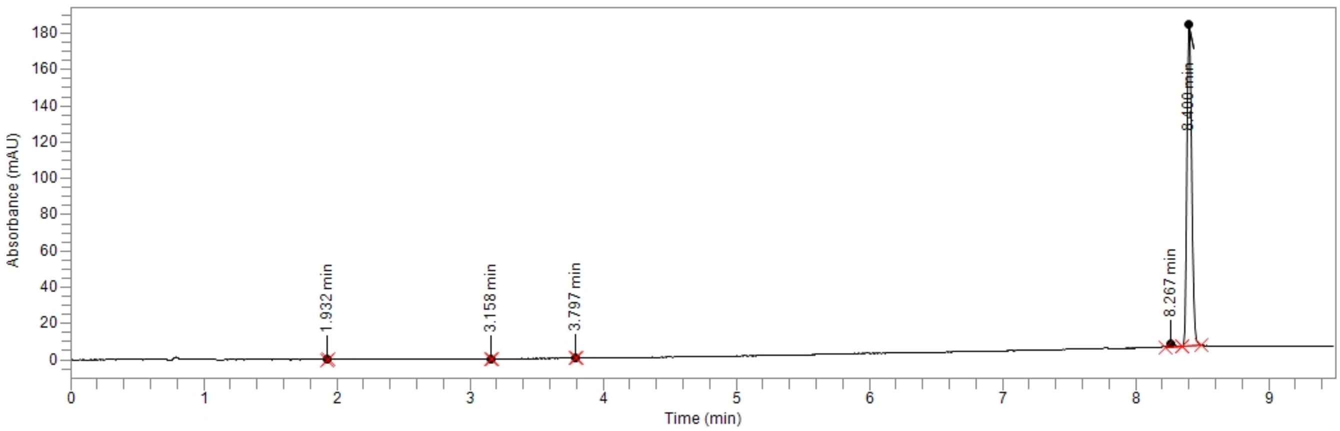

Channel Name **300:10:400:10**

Component Name	Time	Height	Area	Area %	Peak Purity	Wavelength Maximum
	1.932	175.0	68.7	0.02	1.96Fail	196
	3.158	180.9	60.7	0.01	Not Enough Valid Points	199
	3.797	131.8	42.0	0.01	Not Enough Valid Points	199
	8.267	2,013.8	5,434.3	1.22	1.05Pass	201
	8.400	177,604.8	441,385.9	98.75	1.13Pass	202
Total			446,991.6	100.00		

M629-SS2182B : 337:10:400:10 : 1

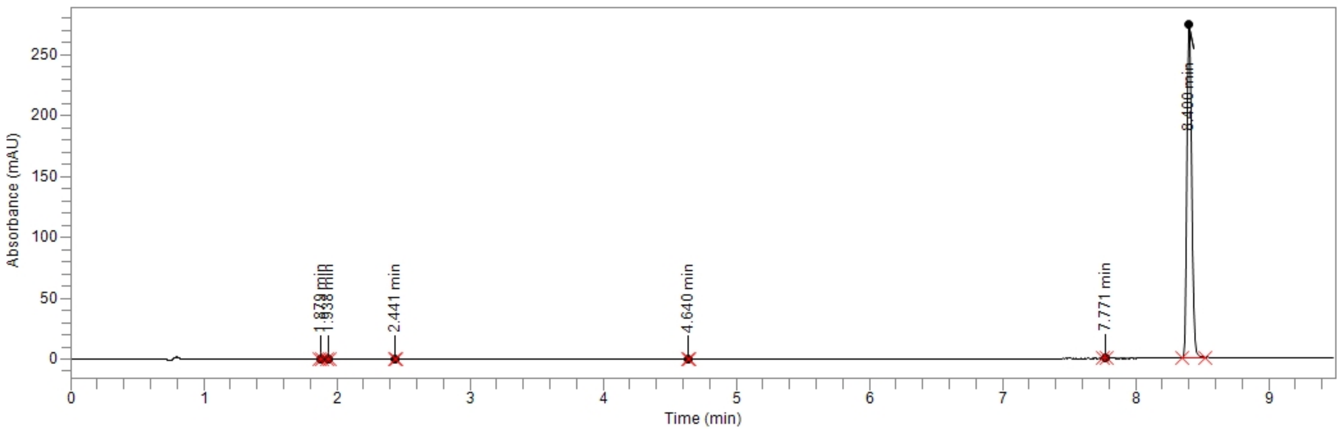

Channel Name **337:10:400:10**

Component Name	Time	Height	Area	Area %	Peak Purity	Wavelength Maximum
	1.879	158.4	54.6	0.01	2.53Fail	199
	1.938	177.3	137.6	0.02	3.48Fail	199
	2.441	137.7	56.9	0.01	Not Enough Valid Points	198
	4.640	152.9	58.6	0.01	Not Enough Valid Points	200
	7.771	220.5	283.8	0.04	1.14Pass	201
	8.400	274,370.6	686,148.4	99.91	1.13Pass	202
Total			686,739.9	100.00		

Report UHPLC

Acquisition Date/Time	7/15/2021 2:26:35 PM	Sample Name	Methanol
Acquisition Method	MS3	Injection Volume	2
Instrument Name	UHPLC		

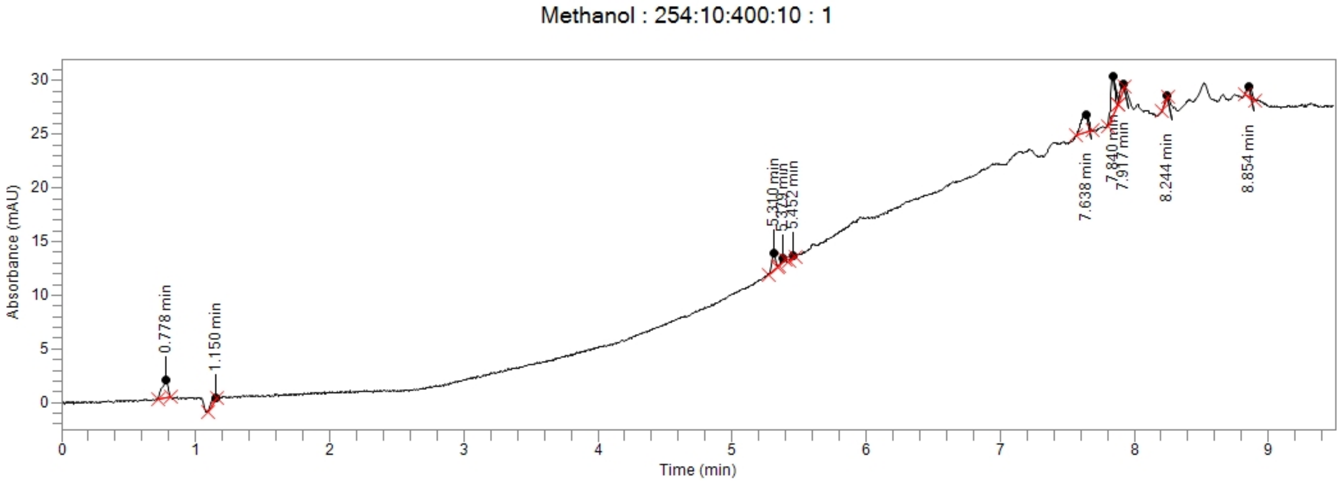

Channel Name **254:10:400:10**

Component Name	Time	Height	Area	Area %	Peak Purity	Wavelength Maximum
	0.778	1,638.4	4,981.4	17.15	Not Enough Valid Points	198
	1.150	223.4	1,246.4	4.29	2.42Fail	191
	5.310	1,560.0	2,953.7	10.17	1.31Pass	199
	5.379	313.0	626.4	2.16	1.19Pass	199
	5.452	291.1	475.3	1.64	1.23Pass	199
	7.638	1,666.2	6,778.4	23.34	1.03Pass	200
	7.840	3,681.4	7,545.3	25.98	1.10Pass	200
	7.917	630.2	1,350.3	4.65	1.06Pass	200
	8.244	410.4	673.0	2.32	1.15Pass	201
	8.854	985.5	2,413.8	8.31	1.06Pass	201
Total			29,043.9	100.00		

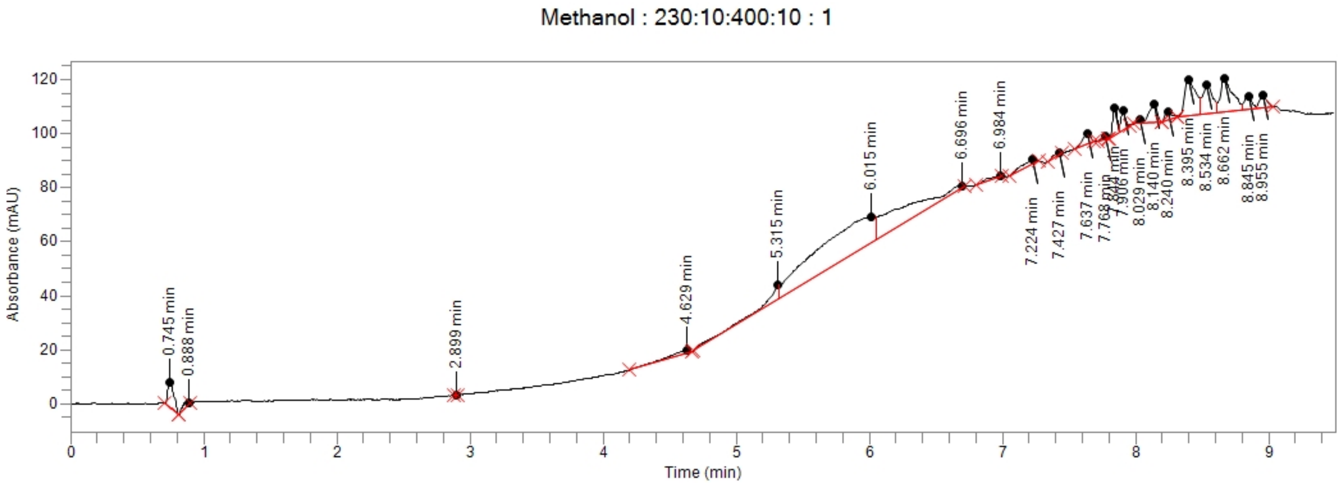

Channel Name **230:10:400:10**

Component Name	Time	Height	Area	Area %	Peak Purity	Wavelength Maximum
	0.745	9,363.2	30,636.4	3.00	Not Enough Valid Points	199
	0.888	475.3	5,376.7	0.53	4.79Fail	194
	2.899	143.4	115.6	0.01	1.22Pass	199
	4.629	1,223.2	16,169.8	1.58	1.50Pass	199
	5.315	5,435.9	38,226.9	3.74	1.55Fail	199
	6.015	9,390.6	401,623.9	39.31	1.24Pass	200
	6.696	589.9	166,003.6	16.25	1.15Pass	200
	6.984	227.6	5,724.3	0.56	1.12Pass	200
	7.224	1,558.1	13,232.1	1.30	1.11Pass	200
	7.427	682.5	3,648.5	0.36	1.13Pass	200
	7.637	3,581.1	15,713.2	1.54	1.15Pass	200
	7.768	987.7	2,273.7	0.22	1.05Pass	200
	7.844	9,753.0	23,292.4	2.28	1.08Pass	200
	7.906	6,703.3	16,410.1	1.61	1.09Pass	200
	8.029	1,384.2	3,938.0	0.39	1.07Pass	200
	8.140	6,885.2	26,977.0	2.64	1.06Pass	201
	8.240	3,135.0	9,478.1	0.93	1.07Pass	201
	8.395	13,224.4	74,979.8	7.34	1.09Pass	201
	8.534	10,576.9	55,241.4	5.41	1.13Pass	201
	8.662	12,428.2	77,244.0	7.56	1.07Pass	201
	8.845	4,657.9	18,355.8	1.80	1.12Pass	201
	8.955	4,408.2	16,894.5	1.65	1.35Pass	201
Total			1,021,555.8	100.00		

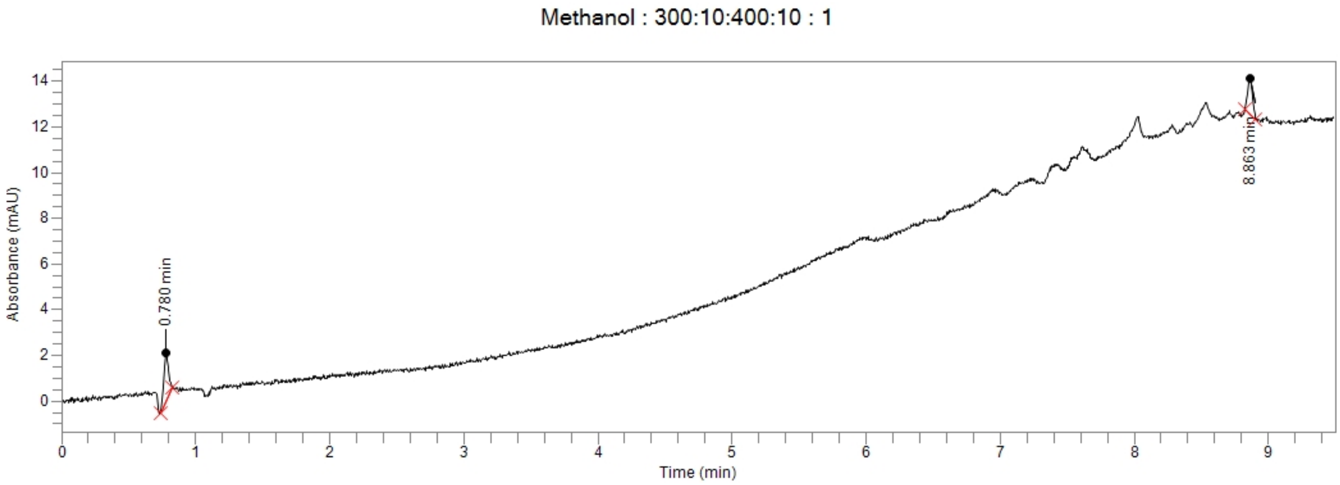

Channel Name **300:10:400:10**

Component Name	Time	Height	Area	Area %	Peak Purity	Wavelength Maximum
	0.780	2,088.5	5,091.2	56.63	Not Enough Valid Points	198
	8.863	1,574.6	3,899.7	43.37	1.09Pass	201
Total			8,990.8	100.00		

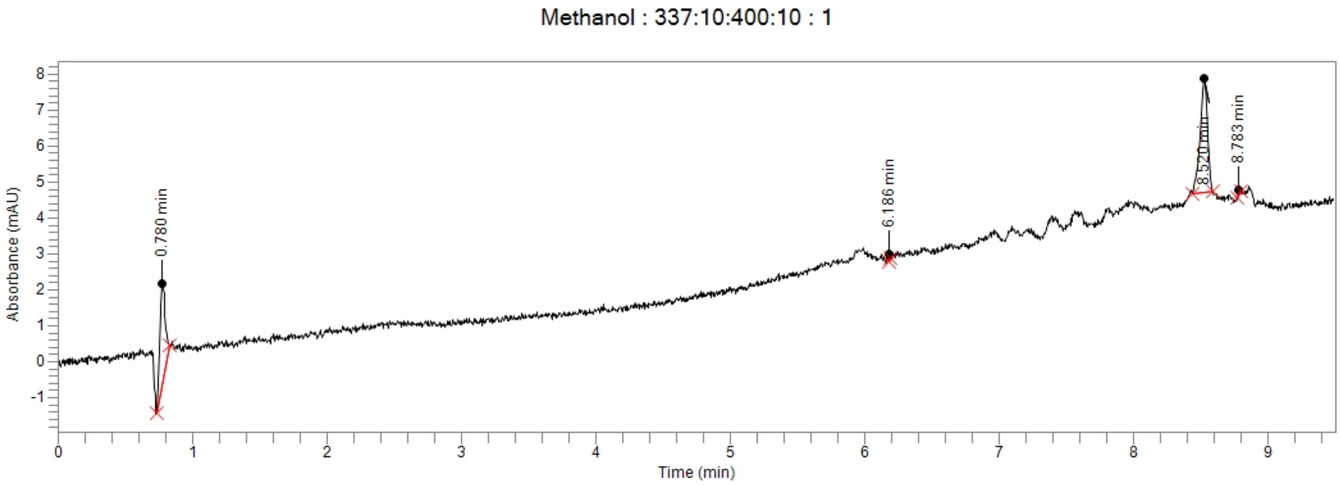

Channel Name **337:10:400:10**

Component Name	Time	Height	Area	Area %	Peak Purity	Wavelength Maximum
	0.780	2,722.8	7,059.7	36.42	Not Enough Valid Points	198
	6.186	162.7	62.6	0.32	1.11Pass	200
	8.520	3,179.9	12,160.1	62.72	1.09Pass	201
	8.783	159.9	104.2	0.54	1.10Pass	201
Total			19,386.7	100.00		
